# Kinetics and Mechanism of Azole n−π*-Catalyzed Amine Acylation

**DOI:** 10.1021/jacs.3c06258

**Published:** 2023-08-01

**Authors:** Harvey J. A. Dale, George R. Hodges, Guy C. Lloyd-Jones

**Affiliations:** † EaStChem, University of Edinburgh, Joseph Black Building, David Brewster Road, Edinburgh EH9 3FJ, U.K.; ‡ Jealott’s Hill International Research Centre, Syngenta, Bracknell, Berkshire RG42 6EY, U.K.

## Abstract

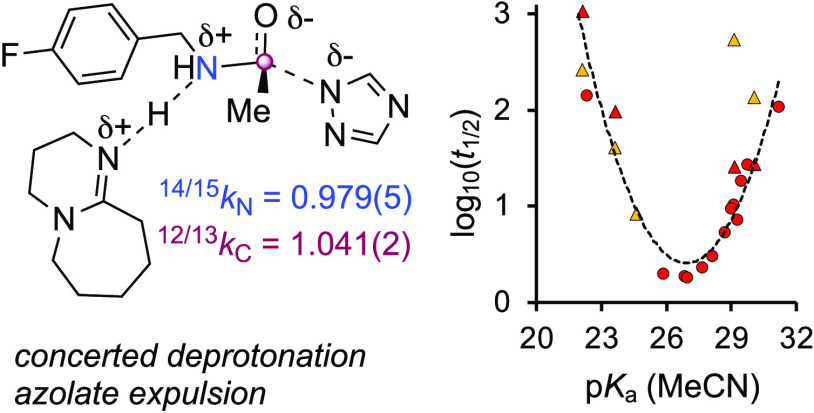

Azole anions are highly competent in the activation of weak acyl donors, but, unlike neutral (aprotic) Lewis bases, are not yet widely applied as acylation catalysts. Using a combination of *in situ* and stopped-flow ^1^H/^19^F NMR spectroscopy, kinetics, isotopic labeling, ^1^H DOSY, and electronic structure calculations, we have investigated azole-catalyzed aminolysis of *p*-fluorophenyl acetate. The global kinetics have been elucidated under four sets of conditions, and the key elementary steps underpinning catalysis deconvoluted using a range of intermediates and transition state probes. While all evidence points to an overarching mechanism involving n−π* catalysis *via N*-acylated azole intermediates, a diverse array of kinetic regimes emerges from this framework. Even seemingly minor changes to the solvent, auxiliary base, or azole catalyst can elicit profound changes in the temporal evolution, thermal sensitivity, and progressive inhibition of catalysis. These observations can only be rationalized by taking a holistic view of the mechanism and a set of limiting regimes for the kinetics. Overall, the analysis of 18 azole catalysts spanning nearly 10 orders of magnitude in acidity highlights the pitfall of pursuing ever more nucleophilic catalysts without regard for catalyst speciation.

## 1 Introduction

**Scheme 1:**
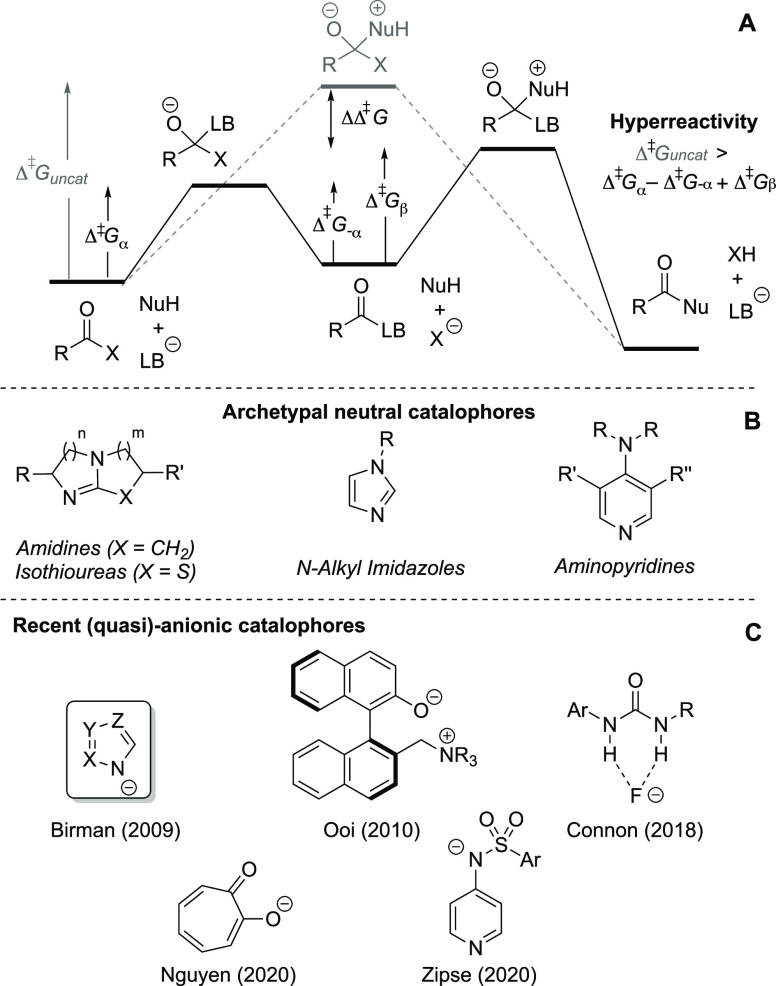
Generic Anionic Lewis Base n−π*-Catalyzed Acylation Satisfying Kemp’s Criterion (A),^2^ and Selected Neutral (B) and Anionic (C) Catalophores

Acyl group transfers can be efficiently accelerated by Lewis base n−π* catalysis.^1^ In these reactions, the base acts as a nucleophile toward the acyl donor and then as a nucleofuge from a hyperreactive^2^ acylated intermediate, Scheme 1A. High catalytic activity is essential for efficient enantioselective acylation unless the background reaction can be actively impeded, e.g., by redox.^3^ While numerous catalysts have been designed for enantioselective acylation,^1b,3h,4^ most are neutral, aprotic, nitrogen-centered π-conjugated Lewis bases, in which the “catalophore”^4b,5^ is an amidine,^5c,6^ isothiourea,^5c,7^*N*-alkylated imidazole,^8^ or *N*′,*N*′-dialkylaminopyridine^5a,8d,9^ (e.g., DMAP^10a^ and PPY^10b^), Scheme 1B. However, a pre-occupation with enantioselectivity has long overshadowed the development of the underlying activity of these catalysts.^5b^ Indeed, even the most recent examples employ the same class of acyl donors that were used with DMAP itself, i.e., acid anhydrides and acyl halides.^10^ Acylative catalysis using less reactive donors is largely absent with aprotic Lewis bases, yet the successful realization of this may be key to solving a range of issues. For example, the use of a weaker acyl donor could suppress the competing background racemic reaction that undermines the enantioselective acylation of unprotected primary amines.^11^

Major advances have been made by Zipse,^10c,10d,12a−12e^ Mayr,^12d−12f^ Namba,^10e^ Dyker,^10f^ and Han,^12g,12h^ through investigation of the features that control the nucleophilicity of the aminopyridine core of DMAP, and then tuning this by annulation,^10c,12b,12d^ ionization,^10d^ and conjugation.^12c^

Initially, these modifications resulted in substantial improvements in catalytic activity;^12d^ however, the development of ever more nucleophilic aminopyridines did not.^10c,12f^ This phenomenon was ascribed to the formation of overstabilized acylated intermediates, but compelling evidence for either a fundamental switch in catalyst speciation, or loss of hyperreactivity,^2^ has proved elusive. Other aprotic catalysts^13^ including aminopyridine *N*-oxides,^13f−13j^ pyridazines,^13k^ amidines and isothioureas,^1b,4b,5b,5c,7a,13j^ as well as those based on (quasi)-anionic catalophores,^14^ including betaines,^14a^ ion-paired fluoride,^14b,14c^ tropolonate anions,^14d^ and pyridinyl amides,^10d^Scheme 1C, have also been developed, but none have elicited transformative activity. Indeed, other than the work of Birman,^15a^*vide infra*, the challenge of activating weak acyl donors using simple organic catalysts has been almost completely unmet.

## 2 Results and Discussion

### 2.1 Azole-Catalyzed Acylation

In 2009, Birman reported that the 1,2,4-triazole anion, generated *in situ* or added as a pre-formed salt, is a potent catalyst for the aminolysis and transesterification of unactivated carboxylic esters.^15a^ Various other azoles were also active, albeit less so than the 1,2,4-triazole, whereas a range of other protic catalysts (e.g., HOBt) and aprotic Lewis bases (e.g., DMAP, NMI), proved essentially inactive under the same conditions. However, attempts to apply the 1,2,4-triazole anion core as a “promising activator”^5b^ in enantioselective catalysis^3h,15b,15c^ have apparently been without success. Intrigued by this, and by the primary kinetic data in Birman’s original report,^15a^ we investigated the mechanism of azole-catalyzed acylation in a systematic and quantitative manner.

Herein, we report the outcome of this study, including the results of *in situ* monitoring by conventional and variable-ratio stopped-flow (VR-SF) ^1^H/^19^F NMR spectroscopy,^16^ numerical and graphical kinetic analyses, synthesis and reactivity of intermediates, activation parameters (Δ^‡^*H*, Δ^‡^*S*), DOSY analysis, ^12,13^C and ^14,15^N kinetic isotope effects (KIEs), and electronic structure calculations. Our results confirm, both by kinetic implication^17^ and by *in situ* spectroscopic detection, the general intermediacy of *N*-acylated azoles under Birman’s conditions,^15a^ and rationalize, *inter alia*, (i) the effect of the solvent on the kinetics; (ii) the significance of the auxiliary base; and (iii) the previously intractable relationship between catalytic activity and azole acidity.^5b,15a^

### 2.2 Preliminary Investigations

We began with single-point analyses (^1^H/^19^F NMR spectroscopy) of the aminolysis of various carboxylic esters under Birman’s conditions.^15a^ The reaction of *p*-fluorophenyl acetate (*p*-F-PhOAc, **1**) with *p*-fluorobenzyl amine (*p*-F-BnNH_2_, **2**), using 1,8-diazabicyclo(5.4.0)undec-7-ene (DBU, **3**) as auxiliary base and 1,2,4-triazole (**4a**_**H**_; 10 mol %) as the catalyst, in MeCN at 20 °C was prime for detailed study. Birman’s data on isosteric substrates (PhOAc, BnNH_2_)^15a^ allowed cross-validation, and the structure of **1** was amenable to isotopic labeling and substituent modification, *vide infra*. Background hydrolysis and aminolysis of **1** in the absence of either catalyst (**4a**_**H**_) or DBU (**3**) was negligible over the timescale of the catalyzed reaction.

**Figure 1:**
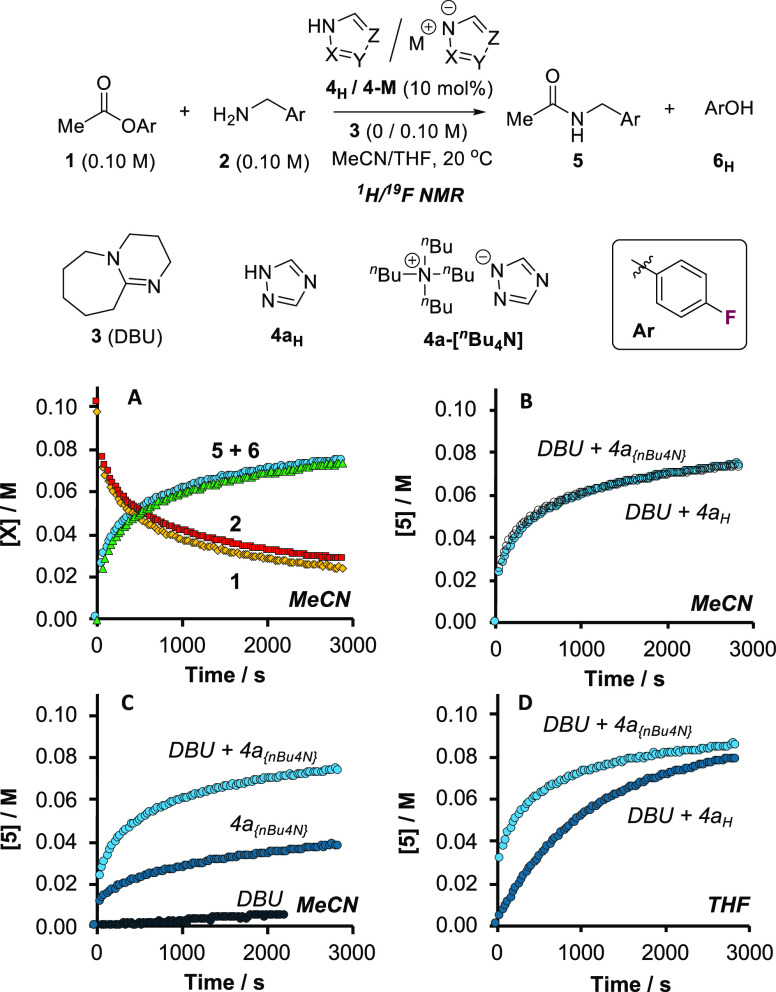
Exploratory ^19^F NMR spectroscopic monitoring of the reaction of **1** (0.10 M) with **2** (0.10 M), using 1-fluoronaphthalene (0.050 M) as internal standard. Conditions: (**A**) DBU (**3**, 0.10 M) and **4a**_**H**_ (10 mol %) in MeCN. (**B**) **3** (0.10 M) and **4a**_**H**_ versus [**4a**][^*n*^Bu_4_N] (10 mol %) in MeCN. (**C**) **3** (0.10 M) versus **3** (0.10 M) and [**4a**][^*n*^Bu_4_N] (10 mol %) versus [**4a**][^*n*^Bu_4_N] (10 mol %) in MeCN. (D) **4a**_**H**_ versus [**4a**][^*n*^Bu_4_N] (10 mol %) in tetrahydrofuran (THF).

Exploratory *in situ*^19^F NMR monitoring experiments conducted under more dilute conditions confirmed that, as reported by Birman,^15a^ the aminolysis in MeCN is initially rapid but soon slows. For example, with 10 mol % **4a**_**H**_ as a catalyst, Figure 1A, 25% conversion of **1** was achieved within 60 s, while 50% conversion required 470 s. For practical reasons, the *in situ* monitoring was generally terminated prior to full conversion (<70%), but subsequent end-point analysis confirmed near-quantitative conversion of **1** and **2** to amide **5** and phenol **6**_**H**_. Provided that modest precautions were taken to exclude adventitious moisture, see Section S3.1 in the Supporting Information (SI), the reaction profiles were highly reproducible: between runs, stock solutions, and batches of **1**, **2**, and **3**, and by NMR method (^1^H NMR in MeCN-*d*_3_ versus ^19^F NMR in MeCN), the latter discounting any significant solvent kinetic isotope effect.

The analysis in MeCN established several salient features. The products (**5** and **6**_**H**_) form concurrently throughout the reaction, without any detectable accumulation of discrete intermediate species, and the total concentrations of 1,2,4-triazole ([**4a**_**H**_]_**T**_) and DBU ([**3**]_**T**_) remain invariant. In the absence of DBU (**3**), i.e., just using 10 mol % **4a**_**H**_, there is no detectable aminolysis over the same period.

Exchanging **4a**_**H**_ (10 mol %) for tetra-*n*-butylammonium 1,2,4-triazolate [**4a**][^*n*^Bu_4_N] (10 mol %) has no discernible impact on the kinetics, Figure 1B. Catalysis by triazolate [**4a**][^*n*^Bu_4_N] (10 mol %) in the absence of DBU (**3**) is initially rapid, liberating stoichiometric (10 mol %) of **5** and **6**, but is followed by a progressively inhibited evolution for the remainder of the reaction, Figure 1C. In the absence of triazole, with just DBU **3** (100 mol %), the aminolysis is very slow indeed.

### 2.3 Identification of Kinetic Regimes I-IV

Further *in situ*^19^F NMR monitoring revealed many nuances. For example, changing the solvent from MeCN to THF (Figure 1D) resulted in distinctly lower-order kinetics and slower initial rates of aminolysis, again with **5** and **6** liberated in parallel. Using the triazolate [**4a**][^*n*^Bu_4_N] instead of the azole **4a**_**H**_ restored the high-order kinetic behavior. Changing the pre-catalyst from triazole **4a**_**H**_ to pyrazole **4b**_**H**_, in addition to affording lower-order kinetics and slower initial rates, resulted in an asynchronous product evolution with **5** lagging behind **6** throughout the course of monitoring*, vide infra*.

**Figure 2:**
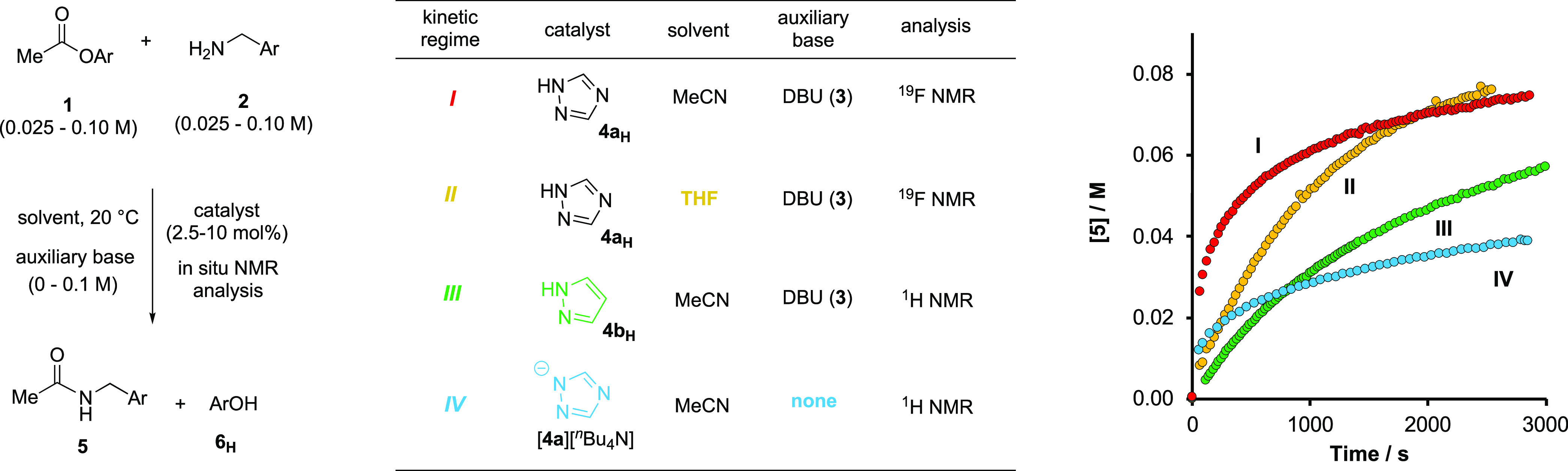
Four distinct kinetic regimes in the azole-catalyzed aminolysis of **1** with **2**. Ar = *p*-F-C_6_H_4_. Each regime was explored systematically by varying the initial concentration of single components relative to reference conditions shown (I, II, III: [**1**]_0_ = [**2**]_0_ = [**3**]_0_ = 0.10 M, [**4a/b**_**H**_]_0_ = 0.010 M; IV: [**1**]_0_ = [**2**]_0_ = 0.10 M, [**4a**][^*n*^Bu_4_N]_0_ = 0.010 M, 10 mol %) using *in situ*^1^H (III) or ^19^F (I, II, IV) NMR spectroscopy in MeCN (I, IV), MeCN-*d*_3_ (III), or THF (II), with 1-fluoronaphthalene (0.050 M; ^19^F NMR) or 1,3,5-trimethoxybenzene (0.033 M; ^1^H NMR) as internal integration standards.

Further evaluation established four distinct regimes (I–IV, Figure 2) for analysis. Regimes I and II involve catalysis by triazole **4a**_**H**_, and differ only by solvent (MeCN, I, versus THF, II). Regime III uses pyrazole **4b**_**H**_ as catalyst, and regime IV uses triazolate [**4a**][^*n*^Bu_4_N] but without auxiliary base, **3**. Both II and IV are in MeCN. The global kinetics under regimes I to IV were then studied in detail using conventional *in situ*^1^H/^19^F NMR spectroscopy,^16^ see Section S3.8 in the Supporting Information. Empirical rate equations for regimes I and II were explored by graphical methods,^18^ with the kinetic order of each component discerned by systematically varying its initial concentration, with all others remaining constant. Numerical methods^16^ were required for analysis of the kinetics under regimes III and IV.

### 2.4 Kinetics under Regime I

The rate of 1,2,4-triazole (**4a**_**H**_) catalyzed aminolysis in MeCN was cleanly first order with respect to the acyl donor [**1**] and the total catalyst [**4a**]_T_, and approximately first order (0.9–1.0) in the amine [**2**]. Exogenous amide **5** had no discernible influence upon the rate of aminolysis. However, assessing the temporal concentration of DBU [**3**] (p*K*_aH_(MeCN) = 24.3)^19^ proved difficult because of the progressive liberation of phenol **6**_**H**_ (p*K*_a_(MeCN) ≈ 27.2), and the complexities associated with acid-base equilibria in aprotic organic media. In accordance with the work of Coetzee,^20a^ Kolthoff,^20b^ Chmurzynski,^20c^ and, more recently, Leito,^20d−20g^^1^H/^19^F NMR titrations (MeCN/MeCN-*d*_3_, 20 °C) of phenol **6**_**H**_ with DBU (**3**), indicated that up to ∼0.5 equiv. of **3** deprotonates ∼0.5 equiv **6**_**H**_, but there is no significant further deprotonation detected beyond this point. Much stronger organic bases, such as the phosphazene superbase Et-P_2_(dma)_5_ (p*K*_aH_(MeCN) = 32.9), were required to liberate the free phenoxide, **6**^**–**^. Internally calibrated and referenced^16^ diffusion-ordered ^1^H NMR spectroscopy (DOSY) confirmed the formation of a highly stable first-order homoconjugate, {**6-6**_**H**_}^**-**^,^21^ from excess **3** and **6**_**H**_ (0.050 M, MeCN), see Section S5 in the Supporting Information for full discussion.

**Figure 3:**
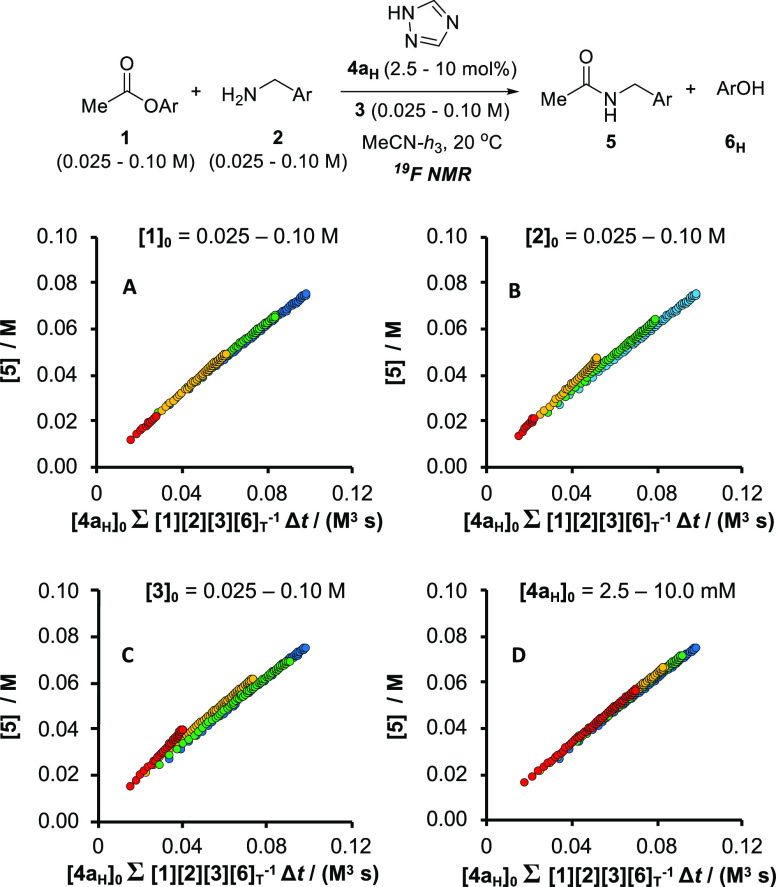
Fully normalized product (**5**) evolution profiles (**A**–**D**) obtained by *in situ*^19^F NMR monitoring of the reaction of **1** with **2**, catalyzed by **4a**_**H**_ in MeCN at 20 °C with DBU (**3**) as an auxiliary base (Regime I). *Within the concentration ranges analyzed*, when [**6**]_T_ > 0.01 M, d[**5**]/d*t* ≈ *k*_obs_^(I)^*[***1**][**2**][**3**][**4a**]_T_/[**6**]_T_, where [**3**] ≈ [**3**]_0_ – [**6**]_T_/2, and *k*_obs_^(I)^ ≈ 0.74 M^–2^ s^–1^.

**Scheme 2:**
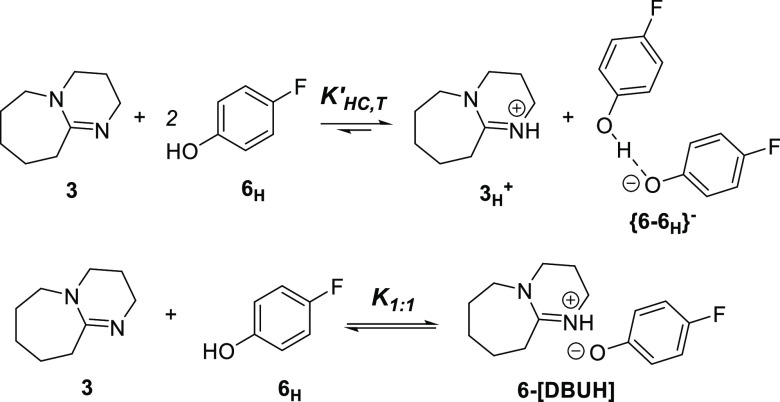
Acid–Base Equilibrium Models for **3** and **6**_**H**_

Nonlinear regression of the ^19^F NMR isotherm to a telescoped equilibrium model, Scheme 2, afforded a phenomenological equilibrium constant of *K*′_HC,T_ = 745 M^–1^, see Section S6.4 in the Supporting Information for a full discussion. Approximating the concentration of free DBU (**3**) at low to moderate conversions as [**3**] ≈ [**3**]_0_ – [**6**]_T_/2 (eq 1) in turn revealed a clear first-order dependence of the aminolysis kinetics on the auxiliary base, [**3**]. An approximately inverse first-order dependence on the total concentration of liberated *p*-fluorophenol [**6**_**H**_]_T_ was then elucidated by full normalization, Figure 3, see Section S3.8.2 in the Supporting Information.
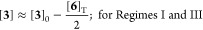
1

### 2.5 Kinetics under Regime II

**Figure 4:**
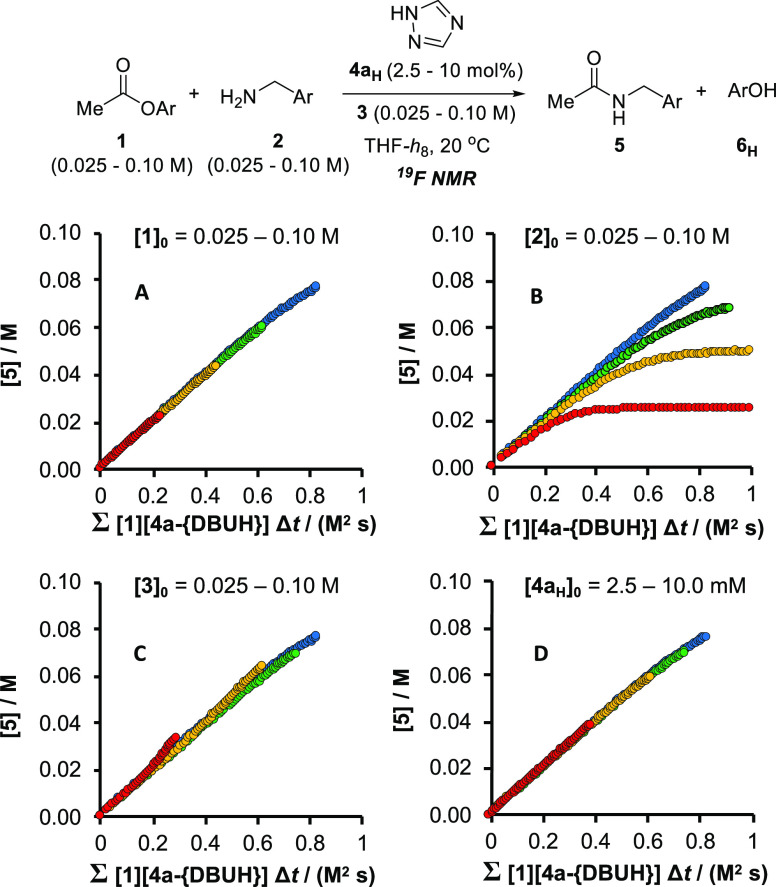
Fully normalized product (**5**) evolution profiles (**A**, **C**, **D**) obtained by *in situ*^19^F NMR monitoring of the reaction of **1** with **2**, catalyzed by **4a**_**H**_ in THF at 20 °C with DBU (**3**) as an auxiliary base (regime II). See text for a discussion of the non-normalization when [**2**] is varied (graph **B**), and the determination of [**4a**_**{DBUH}**_]_*t*_. *Within the concentration ranges analyzed*, when [**1**]_0_ < [**2**]_0_, then d[**5**]/d*t* ≈ *k*_obs_^(II)^[**1**]([**4a**][^*n*^Bu_4_N]), where *k*_obs_^(II)^ ≈ 0.09 M^–1^ s^–1^.

1,2,4-Triazole (**4a**_**H**_) catalyzed aminolysis in THF (regime II) proceeded with distinctly different kinetics (Figure 4) to regime I. While first-order dependencies on the acyl donor [**1**] and catalyst [**4a**]_T_ were still observed, the initial rate (*v*_0_) of aminolysis was approximately independent of amine [**2**]_0_, and only moderately dependent upon DBU [**3**]_0_. With **2** in excess over **1** (i.e., [**1**]_0_/[**2**]_0_ < 1), aminolysis followed a cleanly pseudo-first-order evolution (*v* ≈ *k*′_obs_[**1**]) throughout the full course of monitoring. In contrast, when **2** was limiting (i.e., [**1**]_0_/[**2**]_0_ > 1), systematic deviations in the graphical analysis arose from fractional orders in **2** (*ca* 0.1–0.2) at high conversions (see Figure 4B). Initial rate analysis of the effect of DBU **3**, indicated a fractional order: *v*_0_ ∝ [**3**]_0_^*x*^, where *x* ≈ 0.4–0.5. Graphical normalization of the full reaction profile, assuming [**3**] ≈ [**3**]_0_ – [**6**]_T_/2 (*vide supra)*, afforded analogous partial orders.^22^

Temporal concentrations of the ionized catalyst **4a**_**{DBUH+}**_ and DBU **3** under regime II were not amenable to direct measurement, or to simple analytical approximation. Instead they were estimated by numerical methods simulations using *K*_1:1_(**4a**_**H**_), *K*_1:1_(**6**_**H**_), [**4a**_**H**_]_0_, and [**6**]_T_. This then enabled full graphical normalization for regime II, provided that [**1**]_0_/[**2**]_0_ < 1, Figure 4, see Section S3.8.3 in the Supporting Information.

Titrations of **6**_**H**_ (0.050 M) with **3** in THF suggested negligible homoconjugation with the isotherm satisfactorily simulated by a simple associative (1:1) equilibrium model (*K*_1:1_(**6**_**H**_) = 237 M^–1^, Scheme 2). Analogous isotherms were obtained in the titration of **4a**_**H**_ (0.050 M) with **3** in THF-*d*_8_ (20 °C), with nonlinear regression affording *K*_1:1_(**4a**_**H**_) = 42 M^–1^, see Section S6.4 in the Supporting Information. The approximately zero-order dependence on [**2**] (when [**1**]_0_/[**2**]_0_ < 1) and apparent absence of product inhibition by phenol **6**_**H**_ distinguish the kinetics from regime I, and collectively account for the starkly different reaction profiles in MeCN (regime I) versus THF (regime II) under otherwise identical conditions.^15a^

### 2.6 Kinetic under Regimes III and IV

Systematic analysis of regimes III and IV revealed yet further kinetic intricacies. Aminolysis under regime III in MeCN evolved with approximate first-order dependencies on both the pyrazole catalyst **4b**_**H**_, and the auxiliary base ([**3**] ≈ [**3**]_0_ – [**6**]_T_/2), with complex fractional orders in both [**1**] and [**2**], and no significant product inhibition by **6**_**H**_, see Section S3.8.4 in the Supporting Information.

Catalysis by pre-formed 1,2,4-triazolate [**4a**][^*n*^Bu_4_N] in the absence of DBU (regime IV), proceeded with first-order dependencies on [**1**] and [**2**] and product inhibition by **6**_**H**_, However, in contrast to regimes I, II and III, which all employ DBU **3** as an auxiliary base, regime IV proceeded with second-order dependence on total azole [**4a**]_T_, see Section S3.8.5 in the Supporting Information. For both regime III and regime IV, the global normalization of the kinetic profiles proved intractable due to nuanced fractional orders in substrate (regime III), and complex product inhibition (regime IV) by liberated **6**_**H**_.^23^ Detailed analysis was however achieved by consideration of steady-state kinetic approximations and application of numerical methods, *vide infra*.

### 2.7 Steady-State Approximation and Limiting Conditions for Regimes I, II, and III

**Figure 5:**
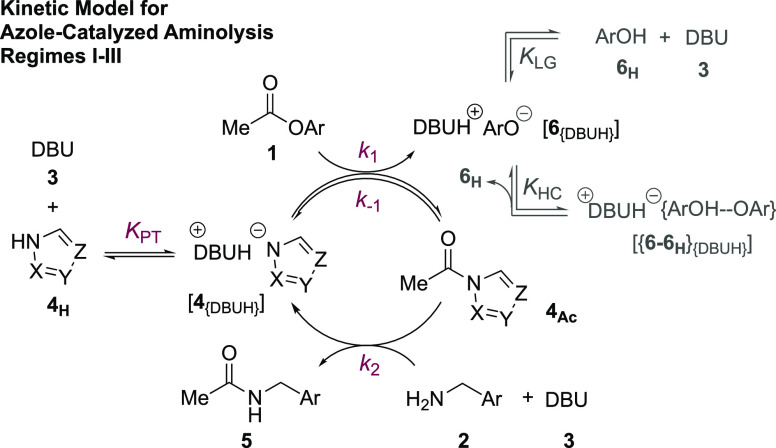
Model employed for derivation of steady-state approximations for product (**5**) evolution from **1** and **2** in terms of their concentrations, plus the total catalyst [**4**]_T_, free DBU [**3**], and phenolate salt [**6**_**{DBUH}**_]. The latter depends on the total concentration of phenol(ate) [**6**]_T_, the acidity of **6**_**H**_ (*K*_LG_), and the propensity for homoconjugation (*K*_HC_).

The diversity in the general kinetic behaviors outlined in Sections 2.2–2.5 might initially suggest the existence of multiple mechanisms for the aminolysis. However, regimes I–III which all employ an auxiliary base, DBU **3**, can be reconciled using a single overarching Lewis base n−π* catalysis mechanism, Figure 5, in which the evolution of amide **5** is described by the steady-state approximation shown in eq 2. Several simplifications to eq 2 can be made by considering limiting regimes. For example, if *N*-acylated intermediate, **4**_**Ac**_ does not significantly accumulate, as found in Regimes I and II, then the steady-state evolution of amide **5** simplifies to eq 3. For sufficiently acidic azoles (*K*_PT_) and when the *N*-acylated intermediate is in rapid pre-equilibrium (*K*_1_), the steady-state evolution of amide **5** further reduces (eq 4) to a form analogous to the empirical rate law of regime I.^24^
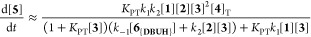
2
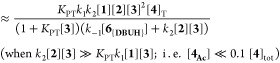
3
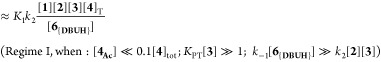
4
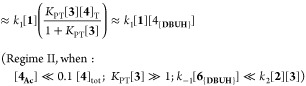
5
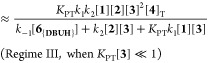
6Alternatively, for less acidic azoles (*K*_PT_) where the *N*-acylated intermediate is efficiently trapped by aminolysis (*k*_2_), the steady-state evolution of amide **5** reduces to a different form (eq 5), analogous to the empirical rate law of regime II. Apparent fractional orders in [**1**] and [**2**], observed under regime III, are only consistent with the mechanism in Figure 5, if the corresponding *N*-acylated intermediate, **4b**_**Ac**_, accumulates significantly during turnover. With less acidic azoles (*K*_PT_), this leads to eq 6.

In eqs 2, 3, 4, and 6, the concentration of the phenolate salt, [**6**_**{DBUH}**_], is dictated by the overall concentration of liberated phenol, [**6**]_T_ = [**1**]_0_ – [**1**], the relative acidities of **3**_**H**_^**+**^ and **6**_**H**_ (*K*_LG_), and the tendency of **6**_**H**_ to undergo homoconjugation (*K*_HC_). For simplicity, the phenolates [**6**_**{DBUH}**_] and {**6-6**_**H**_}_**{DBUH}**_, were evaluated as ion-paired species, eq 7. Analytical or numerical solutions to cubic eq 8 allow estimation of the phenomenological equilibrium constants *K*_LG_ and *K*_HC_ by ^19^F NMR titration of **6**_**H**_ with DBU (**3**); see model 6 in Section S6.3 in the Supporting Information for a full discussion.
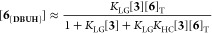
7
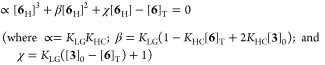
8

### 2.8 Analysis of Acyl Intermediates (**4**_**Ac**_)

**Figure 6:**
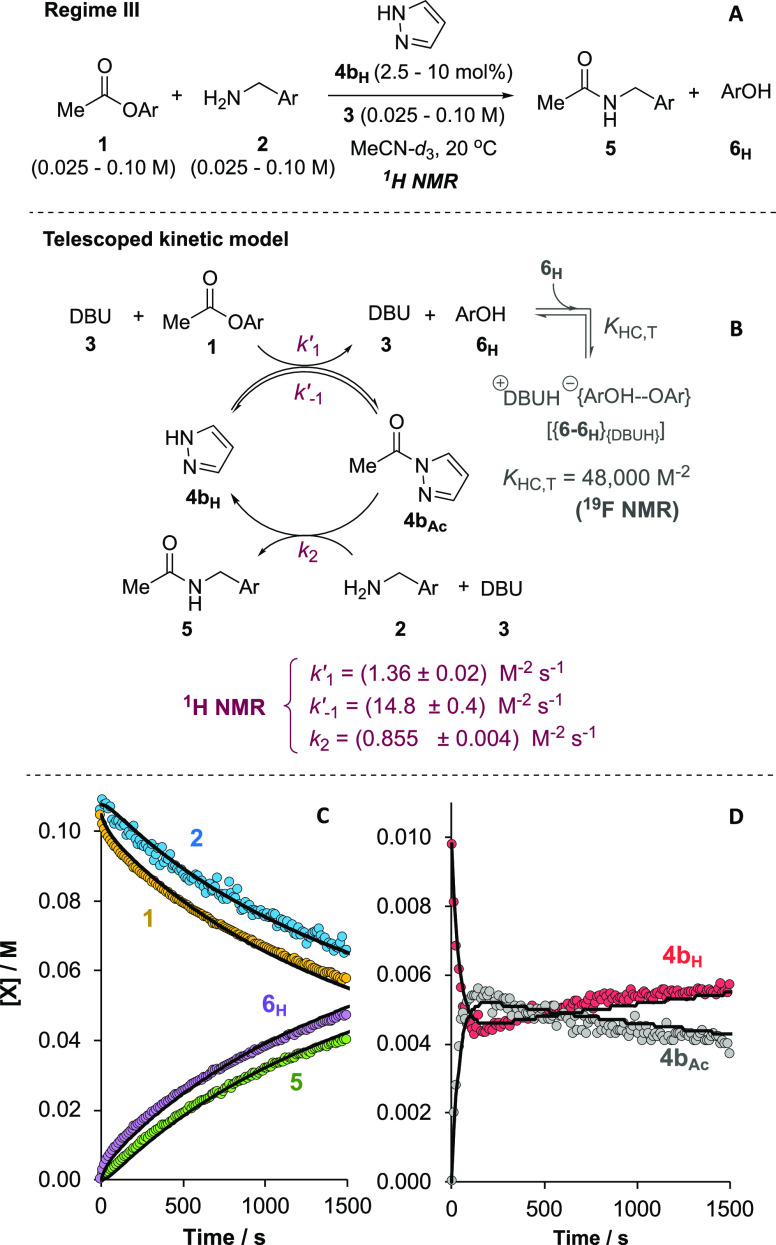
(A) Regime III (**4b**_**H**_-catalysis in MeCN) initiated with different initial concentrations of **1**, **2**, **3**, and **4b**_**H**_. (B) Model employed for holistic numerical methods fitting of data. (C) Example temporal concentration profile of [**1**], [**2**], [**5**], and [**6**]_**T**_ obtained by VR-SF-^1^H NMR, MeCN-*d*_3_, 20 °C; [**1**]_0_, [**2**]_0_, [**3**]_0_ = 0.10 M; [**4b**_**H**_]_0_, 10 mol %. Solid lines are the profiles predicted using the holistic numerical methods fitting from all 15 runs. (D) Temporal concentration profiles of [**4b**_**Ac**_] and [**4b**_**H**_] and fitting, from the same run as (C).

Quantitative ^1^H and ^19^F NMR monitoring of the aminolysis of **1** under regime III (catalysis by **4b**_**H**_ in MeCN, Figure 6A) revealed that an intermediate *N*-acylated species **4b**_**Ac**_, Figure 6B, (δ_**H**_ (MeCN) CH_3_CO = 2.67 ppm) is generated *in situ* at steady state from **1** + **4b**_**H**_, together with a trace of acetate anion. The identity of **4b**_**Ac**_ was confirmed by independent synthesis, see Section S2.4 in the Supporting Information. Under standard conditions, conventional *in situ*^1^H NMR monitoring, Figure 6C, was just able to capture the onset of steady state, with **4b**_**Ac**_ attaining a maximal fractional population of *f*_Ac_ = [**4b**_**Ac**_]/[**4b**]_0_ ≈ 53% after around 160 s and then slowly decaying thereafter, Figure 6D.

Modulating [**3**]_0_ (0.025–0.10 M) under otherwise standard conditions had no significant effect on *f*_Ac_ (max) other than the time taken to reach steady state. With ^1^H NMR spectroscopic analysis providing both the temporal evolutions of [**1**], [**2**], and [**5**], Figure 6C, and the catalyst speciation ([**4b**_**Ac**_]/[**4b**]_T_), Figure 6D, a full complement of kinetic data were acquired under regime III by varying [**1**]_**0**_, [**2**]_**0**_, [**3**]_**0**_ and [**4b**_**H**_]_0_. With standard graphical analysis intractable due to the significant accumulation of **4b**_**Ac**_, the resulting data were globally fitted to a telescoped kinetic model, Figure 6B. Satisfactory correlations were obtained across a total of 15 data sets, with the model capturing the kinetic significance of all key components, as well as independent values for *k*′_1_, *k*′_–1_ and *k*_2_; see Section S3.8.4 in the Supporting Information for the full sets of fitted data.

Stoichiometric aminolysis of 1:1 **4a**_**Ac**_ + α-[D_3_]-**4a**_**Ac**_, and **4a**_**H**_-catalyzed (10 mol %) aminolysis of 1:1 **1** + α-[D_3_]-**1** (0.05: 0.05 M), both gave **5** + α-[D_3_]-**5** with negligible H/D exchange. Thus, the enolization of **4a**_**Ac**_ by DBU (**3**) is kinetically insignificant under the standard catalytic conditions, and neither aminolysis step proceed *via* ketene (CH_2_=CO) elimination from **1** or **4a**_**Ac**_.^25^

### 2.9 Role of the Auxiliary Base

**Figure 7:**
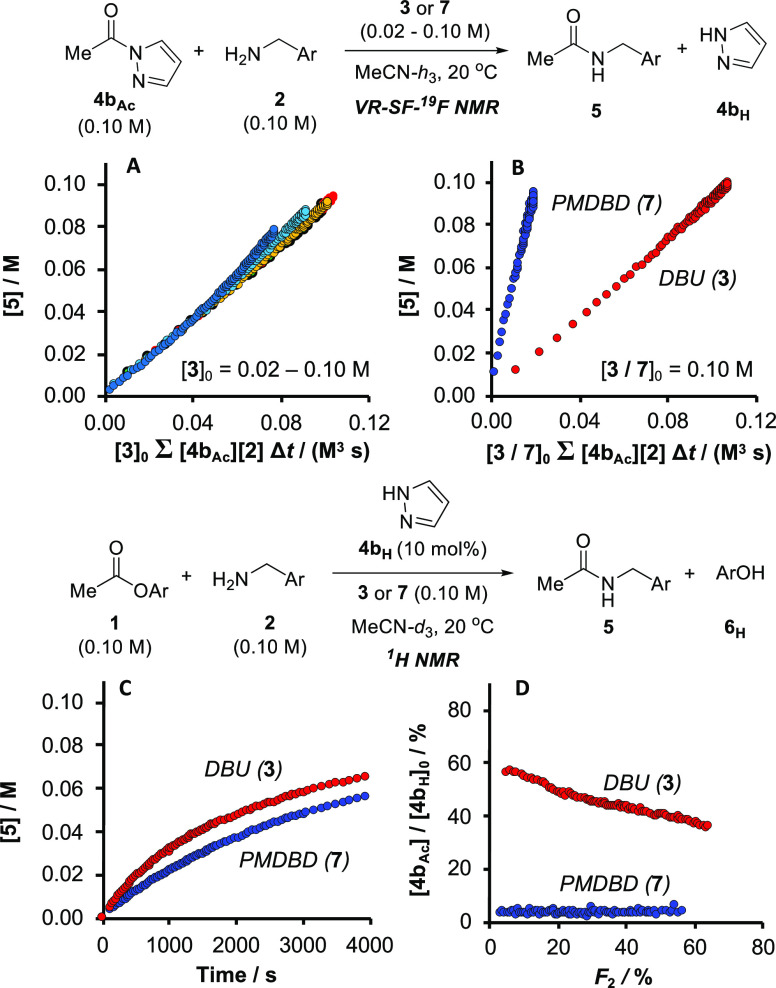
(A) Fully normalized kinetic profiles (VR-SF-^19^F NMR) for the stoichiometric aminolysis of *N*-acetyl pyrazole **4b**_**Ac**_ with **2** at variable initial concentrations of auxiliary DBU 3 (0.02–0.10 M) in MeCN (20 °C); (B) Comparison of fully normalized kinetic profiles for the aminolysis of **4b**_**Ac**_ and **2** with *either***3** or PMDBD (3,3,6,9,9-pentamethyl-2,10-diazabicyclo[4.4.0]dec-1-ene) **7** (0.10 M). (C) Comparison of amide (**5**) evolution under regime III (**4b**_**H**_, 10 mol %; ^1^H NMR, MeCN-*d*_3_, 20 °C) with either **3** or **7** as the auxiliary base. (D) Catalyst speciation ([**4b**_**Ac**_]/[**4b**_**H**_]_0_) from the same run as (C). *F*_2_ is the fractional conversion (%) of amine **2**, i.e., ([**2**]_0_-[**2**]_*t*_)/0.01[**2**]_0_.

Previous assessments of the role of the auxiliary base, DBU **3**, in azole-catalyzed acylations focused solely on the deprotonation (*K*_PT_) of **4**_**H**_.^5b,15a^ In contrast, the overarching mechanism shown in Figure 5 includes two additional roles for DBU: homoconjugation of liberated phenol **6**_**H**_ (*K*_LG_; *K*_HC_) and catalysis of the aminolysis of **4**_**Ac**_ (*k*_2_). To probe the latter in more detail, the reaction of independently synthesized *N*-acetyl pyrazole **4b**_**Ac**_ (0.10 M) with amine **2** (0.10 M) was analyzed using VR-SF-^19^F NMR (MeCN, 20 °C) across a series of DBU concentrations (**3**, 0.02–0.10 M), Figure 7A; see Section S3.8.6 in the Supporting Information.

Component-specific and subsequent global graphical normalization of the resulting kinetic data confirmed a formal termolecular rate law of the form *k*_obs_[**4b**_**Ac**_][**2**][**3**], and thus an explicit role for the auxiliary base (**3**) in the aminolysis of the *N*-acyl intermediates (**4**_**Ac**_) under catalytic conditions. The termolecular rate law, *k*_obs_[**4b**_**Ac**_][**2**][**3**], does not distinguish whether DBU accelerates aminolysis of **4b**_**Ac**_ by acting as general Brønsted base, or by the generation of a second, more reactive, *N*-acetylated intermediate, [DBU-**3**_**Ac**_]^**+**^. An identical set of VR-SF-^19^F NMR analyses of the stoichiometric aminolysis of **4b**_**Ac**_ by amine **2**, but replacing DBU **3** with 3,3,6,9,9-pentamethyl-2,10-diazabicyclo[4.4.0]dec-1-ene (PMDBD, **7**), evolved with formal termolecular kinetics, but at an approximately five-fold greater rate (Figure 7B). PMDBD (**7**) is less basic (p*K*_aH_(MeCN) = 22.6) and significantly more sterically hindered than DBU **3** (p*K*_aH_(MeCN) = 24.3), features that in the absence of other factors, are expected to attenuate the aminolysis of **4b**_**Ac**_, by either mechanism. However, unlike DBU **3**, the PMDBD (**7**) can engage in tautomeric (bifunctional) catalysis involving simultaneous donation (NH) and acceptance (N) of a proton. This phenomenon can reasonably account for the greater efficiency of PMDBD **7**, relative to DBU **3**, in catalyzing the aminolysis of **4b**_**Ac**_, and suggests both proceed via Brønsted base effects, rather than Lewis base n−π* catalysis.

In contrast to the stoichiometric reactions, the **4b**_**H**_-catalyzed acylation of amine **2** using PMDBD **7** as auxiliary base proceeded marginally slower than with DBU **3** (Figure 7C) and with a very much lower steady-state population of the acyl intermediate **4b**_**Ac**_ (Figure 7D). Overall, this is the combined outcome of more efficient consumption of **4b**_**Ac**_ by amine **2** and its much less efficient regeneration from **1**. The latter is likely due to the lower basicity of PMDBD **7** and/or attenuation of the nucleophilicity of the pyrazolate anion toward **1** by charge-reinforced hydrogen bonding in the azolate **4b**_**{PMDBDH}**_.

### 2.10 Comparison of Activation Parameters under Regimes I, II, and III

If the kinetics of regimes I–III are interpreted as limiting manifestations of the mechanism in Figure 5, then catalysis proceeds *via* two overarching sequential aminolyses: ester **1** by azolate [**4**]^−^ to form acyl intermediate **4**_**Ac**_, and then **4**_**Ac**_ by amine **2** to form amide **5**. The global kinetics indicate that the rate-determining transition state of regime I (**4a**_**H**_ in MeCN) is the second aminolysis, while for regime II (**4a**_**H**_ in THF) it is the first. Conversely, regime III (**4b**_**H**_ in MeCN) is kinetically nuanced, with (at least) two energetically near-degenerate transition states exerting collective control over the rate of product formation: one leading to intermediate **4b**_**Ac**_, the other to its consumption.

Comparison of activation parameters for the aminolysis steps across the three regimes allowed further testing of these conclusions. Catalysis under regime III, in which the *N*-acylated intermediate **4b**_**Ac**_ could be detected by ^1^H NMR spectroscopy, provided the benchmark for these comparisons. Two independent rate coefficients (*k*′_1_, *k*_2_) were determined by simultaneous numerical methods fitting of temporal concentration data for [**1**], [**2**], [**5**], and [**4b**_**Ac**_], obtained by VT-SF-^1^H NMR, to the telescoped kinetic model shown in Figure 6B.^26^ Activation parameters for regime III (Table 1, entry 1) were estimated from standard reciprocal temperature plots. The activation parameters were also corroborated in stoichiometric experiments that generated (Table 1, entry 2) and consumed (entries 3 and 4) intermediate **4b**_**Ac**_, see Section S3.8.7 in the Supporting Information. The difference in activation parameters for aminolysis of **4b**_**Ac**_ catalyzed by auxiliary base **3** versus **7** (entries 3 versus 4) suggests that the relative stabilization of the rate-determining transition state with **7** is almost exclusively enthalpic in origin, a key hallmark of tautomeric catalysis.^27^

**Table 1:** Activation Parameters (Δ^‡^*H*/kJ mol^–1^; Δ^‡^*S*/J K^–1^mol^–1^) for Regimes I, II, and III^a^

entry	regime/solv./T	components	Δ^‡^*H*′_1_ (Δ^‡^*H*^(I)^)	Δ^‡^*S*′_1_ (Δ^‡^*S*^(I)^)	Δ^‡^*H*′_2_ (Δ^‡^*H*^(II)^)	Δ^‡^*S*′_2_ (Δ^‡^*S*^(II)^)
1^b^	III/MeCN/10–40 °C	**1**, **2**, **3**, **4b**_**H**_ (cat.)	19	–178	4	–233
2^c^	III/MeCN/10–40 °C	**1**, **3**, **4b**_**H**_ (stoich.)	23	–164	–	–
3^d^	III/MeCN/10–40 °C	**2**, **3**, **4b**_**Ac**_ (stoich.)	–	–	3	–237
4^d^	III/MeCN/10–40 °C	**2**, **7**, **4b**_**Ac**_ (stoich.)	–	–	0	–233
5^e^	I/MeCN/20–50°C	**1**, **2**, **3**, **4a**_**H**_ (cat.)	–f	–f	(10)^f^	(−214)^f^
6^g^	I/MeCN/10–40 °C	**2**, **4a**_**Ac**_ (stoich.)^h^	–	–	–2	–219
7^i^	II/THF/20–40 °C	**1**, **2**, **3**, **4a**_**H**_ (cat.)	(23)^f^	(−189)^f^	–f	–f

a Activation parameters estimated from standard plots of ln(*k*/*T*) versus (1/*T*) using rate coefficients (*k*′_1_, *k*_2_, *k*^(I)^, *k*^(II)^) extracted from kinetic data obtained by VR-SF-^1^H NMR, under catalytic (cat.) and stoichiometric (stoich.) conditions; see Section S3.8.7 in the Supporting Information.

b [**1**]_0_, [**2**]_0_, [**3**]_0_ = 0.10 M, [**4b**_**H**_]_0_, 0.01 M, *k*′_1_, *k*_2_, by numerical fitting.

c [**1**]_0_, [**3**]_0_ = 0.10 M, [**4b**_**H**_]_0_ = 0.020 M, *k*′_1_ by graphical normalization.

d [**4b**_**Ac**_]_0_, [**2**]_0_, [**3,7**]_0_ = 0.10 M, *k*_2_ by graphical normalization.

e [**1**]_0_, [**2**]_0_, [**3**]_0_ = 0.10 M, [**4a**_**H**_]_0_ = 0.01 M.

f Acyl intermediate does not accumulate: the activation parameters are based on a single phenomenological rate coefficient, *k*^(I)^ or *k*^(II)^, by graphical normalization.

g [**2**]_0_, [**4a**_**Ac**_]_0_ = 0.1 M; autocatalytic in **4a**_**H**_, *v* = *k*_obs_[**4a**_**Ac**_][**2**][**4**_**aH**_].

h Reaction complete in under 0.2 s in the presence of **3**, 0.1 M.

i [**1**]_0_, [**2**]_0_, [**3**]_0_ = 0.10 M, [**4a**_**H**_]_0_ = 0.005 M, graphically normalized at low conversion (<20%) to enable the approximations [**4a**_**{DBUH}**_] ≈ [**4a**_**H**_]_0_ and *v* ≈ *k*^(II)^[**1**][**4a**_**H**_]_0_.

**Figure 8:**
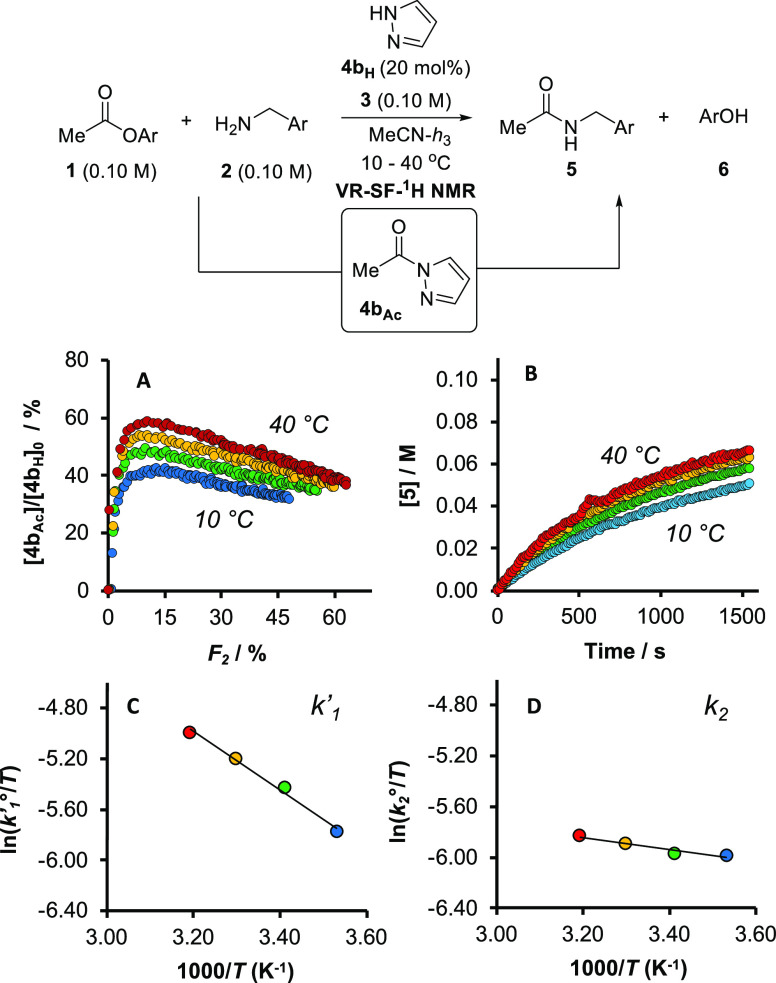
VT-SF-^1^H NMR analysis of the aminolysis under regime III ([**1**]_0_, [**2**]_0_, [**3**]_0_ = 0.10 M; [**4b**_**H**_]_0_ = 0.02 M) 20 mol %; MeCN, 10 to 40 °C; *k*′_1_(*T*) and *k*′_2_(*T*) were determined at each temperature by numerical methods fitting of the profiles of [**1**], [**2**], [**5**], and [**4b**_**Ac**_] to the kinetic model in Figure 6B. (A) Acyl-speciation of catalyst. (B) Temporal evolution of amide **5**. (C) Eyring analysis of *k*′_1_(*T*) (*k*′_1_° = *k*′_1_.*c°*^2^; *c°* = 1 M). (D) Eyring analysis of *k*_2_(*T*) (*k*_2_° = *k*_2_.*c°*^2^; *c°* = 1 M).

The rate of aminolysis of intermediate **4b**_**Ac**_ is nearly temperature-independent, in the range studied, see Section S3.8.7 in the Supporting Information. As evident from the comparison of the activation parameters in Table 1, entries 1–4, increasing the reaction temperature for the catalytic process results in a higher speciation of the *N*-acyl intermediate **4b**_**Ac**_, Figure 8A, but only a very modest increase in the rate of amide (**5**) formation Figure 8B. Although both aminolyses (*k*′_1_, Figure 8C, and *k*_2_, Figure 8D) are formally termolecular, the opposing differentials in activation enthalpy and entropy suggest they proceed by microscopically distinct mechanisms.

Activation parameters were then estimated for **4a**_**H**_-catalyzed aminolysis under Regimes I and II. Since the *N*-acyl intermediate **4a**_**Ac**_ does not detectably accumulate during turnover under either regime, a single phenomenological rate coefficient was determined (*k*^(I)^, *k*^(II)^) at each temperature using eqs 4 and 5. There is a lower enthalpic and larger entropic barrier to overall turnover in regime I (MeCN, Table 1 entries 5 and 6) compared to regime II (THF, entry 7), resulting in a significantly lower temperature dependence of the turnover rate under regime I, see Section S3.8.7 in the Supporting Information.

Direct quantitative comparison of activation parameters for the two individual aminolysis steps of regime III (Table 1, entries 1–3) with regimes I and II is precluded by differences in ground state (III/I)^28^ and solvent (III/II). Nonetheless, the overall activation parameters for turnover rate-limiting generation of the acyl intermediate (regime II) are similar to those of the first aminolysis (*k*′_1_) in regime III (entries 1 and 7) and analogously, the activation parameters for regime I, in which acyl intermediate consumption is turnover rate-limiting, are similar to the second aminolysis (*k*_2_) in regime III (entries 1 and 5).

We attempted to refine the comparison of Regimes I and III by determining the kinetics of the stoichiometric aminolysis of independently synthesized **4a**_**Ac**_ with **2** and auxiliary base DBU (**3**) in MeCN. However, the reaction was too rapid to monitor by VR-SF-^19^F NMR.^29^ Nonetheless, in the absence DBU (**3**), the aminolysis slowed sufficiently to permit the acquisition of variable-temperature kinetic data, see Section S3.8.7 in the Supporting Information. The evolution of **5** under these auxiliary base-free conditions was found to be of first order in **4a**_**Ac**_ and in **2**, with an additional and dominant first-order autocatalytic dependence on **4a**_**H**_.^30^ The first-order autocatalysis by **4a**_**H**_ (p*K*_a_(MeCN) 9.4) instead of a second-order dependence on the far more basic amine **2** (p*K*_aH_(MeCN) = 16.9) suggests tautomeric catalysis^27^ by **4a**_**H**_. The rate of this formally termolecular process was temperature-independent in the range studied (10–40 °C) with a weakly negative enthalpic barrier and a substantial negative entropic contribution to the free energy of activation, Table 1, entry 6.

Overall, the general correspondence between Δ^‡^*H*^(II)^/Δ^‡^*S*^(II)^ (THF, II, entry 7) and Δ^‡^*H*′_1_/Δ^‡^*S*′_1_ (MeCN, III, entry 1), and between Δ^‡^*H*^(II)^/Δ^‡^*S*^(II)^ (MeCN, I, entry 5) and Δ^‡^*H*′_2_/Δ^‡^*S*′_2_ (MeCN, III, entry 1) suggests that the structures of the rate-determining transition states traversed in the generation and consumption of **4**_**Ac**_ are similar in all three regimes,^31^ despite differences in catalyst structure (**4a**/**4b**) and solvent ionizing strength (MeCN/THF). This conclusion is supported by the similar Hammett reaction constants (ρ^(I)^ = −0.50, MeCN; ρ^(II)^ = −0.34, THF) for commitment of the amine substrate (**2**) in the second stage (*k*_2_) of regimes I and II, as determined by the intermolecular competition of a series of *p*-substituted benzylamines, see Section S4.1 in the Supporting Information.

### 2.11 Structure–Activity Relationships

A key point noted in Birman’s original report,^15a^ and in a subsequent review,^5b^ was the apparent absence of a tractable relationship between the azole acidity (p*K*_a_, DMSO) and the catalytic efficiency, based on the first half-life of the acyl donor (PhOAc).^15a^

**Figure 9:**
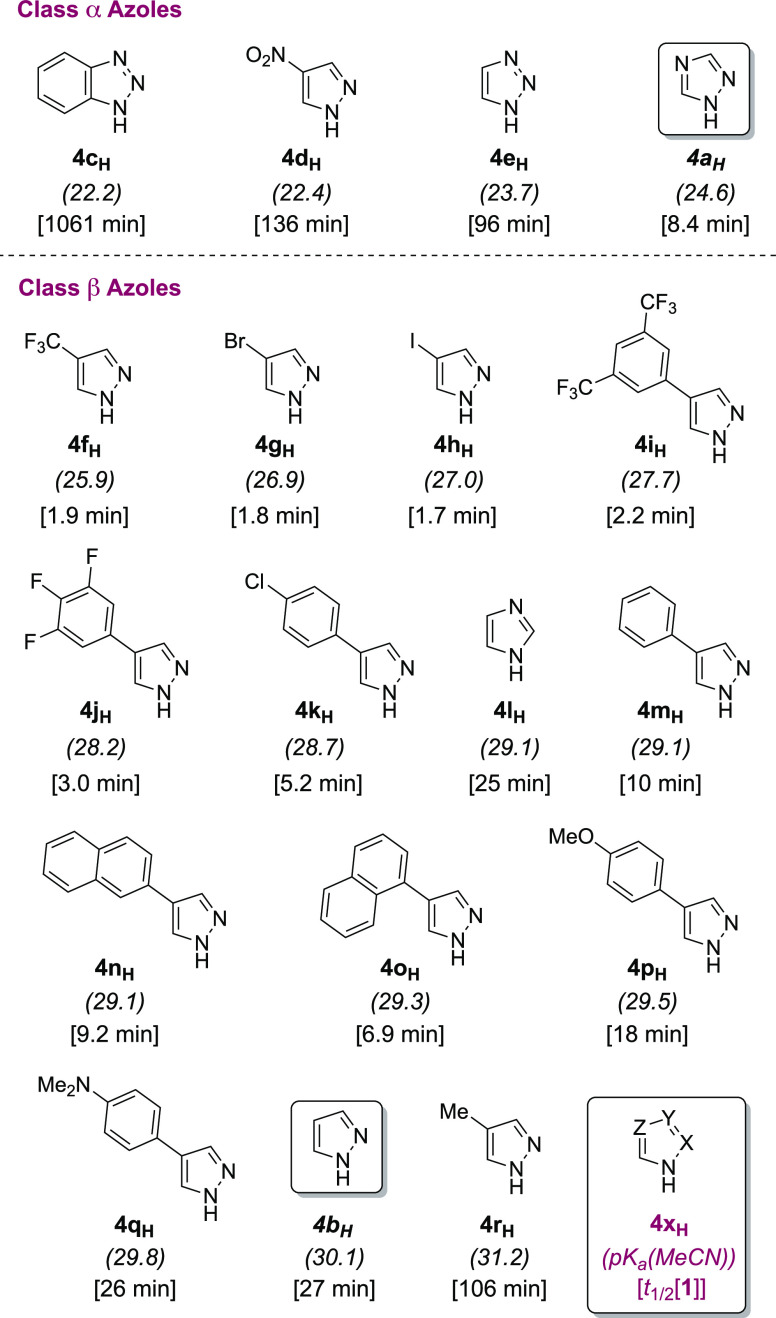
Classification (α/β) of the 18 azoles compared as aminolysis catalysts, see Figure 10, based on p*K*_a_(MeCN). Values in parentheses are experimental or calculated thermodynamic acidities, p*K*_a_(MeCN). Values in brackets are the first half-life of **1** (*t*_1/2_[**1**], min), conditions as Figure 10, see Section S3.8.8 in the Supporting Information. For class β azoles, the *N*-acetylated intermediate **4**_**Ac**_ accumulates sufficiently to be detected by *in situ*^1^H NMR spectroscopy during turnover. Half-lives for catalysis by **4c**_**H**_, **4d**_**H**_, and **4r**_**H**_ determined by numerical methods fitting and extrapolation to [**1**]/[**1**]_0_ = 0.5.

Of a wide range of azoles tested, by far the most efficient was 1,2,4-triazole **4a**_**H**_, which in the presence of the auxilliary base DBU (**3**) was concluded to generate the triazolate [**4a**]^−^ as the active species. 1,2,4-Triazole **4a**_**H**_ remains the most effective simple Lewis base catalyst reported to date for the direct aminolysis and transesterification of weakly activated esters.^5b,15a,15b^ To better understand the mechanistic origins of these observations, we tested a series of azoles (**4a-r**_**H**_; Figure 9) that were selected to provide acidities spanning nearly 10 orders of magnitude. Eight 4-aryl-substituted pyrazoles were synthesized by Suzuki–Miyaura arylation of unprotected or *N*-benzyl protected 4-bromopyrazole,^32^ see Section S2.2 in the Supporting Information, the remaining 10 azoles were obtained from commercial sources. Thermodynamic acidities of azoles **4a-r**_**H**_ p*K*_a_(MeCN) = 22.1–31.2 were determined using the experimental acidity of imidazole (p*K*_a_(MeCN)= 29.1)^20g,33^ as an anchor and parameters determined from the linear regression of computed (KS-DFT/DLPNO-CCSD(T)) and experimental acidities for seven substituted indoles (p*K*_a_(MeCN) = 23.6–32.6, RMSE = 0.30),^20g^ covering a comparable range of acidities, see Section S7.3 in the Supporting Information.

**Figure 10:**
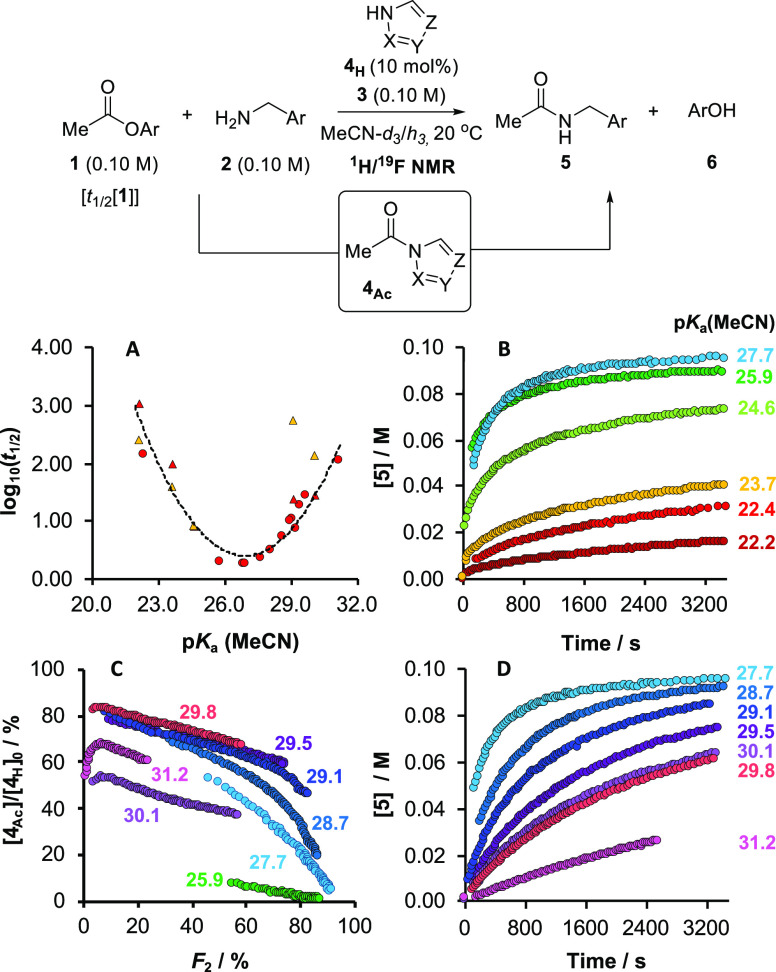
Kinetics of aminolysis ([**1**]_0_, [**2**]_0_, [**3**]_0_ = 0.10 M) catalyzed by azoles (**4**_**H**_; 10 mol % at 20 °C) analyzed by *in situ*^1^H or ^19^F NMR spectroscopy, in MeCN-*d*_3_ or MeCN, respectively. (A) Empirical catalytic efficiency, as quantified by the first half-life of **1** (*t*_1/2_[**1**], min), compared to azole acidity, p*K*_a_(MeCN). Red data points this work; yellow are data reported by Birman^15a^ for an analogous system (PhOAc, ^*i*^PrNH_2_, DBU, azole; CDCl_3_); triangular data points denote azoles included in both studies. (B) Example amide evolution profiles using azoles p*K*_a_(MeCN) ≤ 27.7. (C) Catalyst speciation profiles [**4**_**Ac**_]/[**4**_**H**_]_0_ versus fractional conversion, *F*_2_, of **2** for selected azoles p*K*_a_(MeCN) ≥27.7. (D) Selected amide evolution profiles using azoles p*K*_a_(MeCN) ≥27.7.

The efficiency of each of the 18 azole catalysts (**4a**_**H**_ to **4r**_**H**_) was then compared by aminolysis of ester **1** monitored *in situ* by either ^1^H NMR (MeCN-*d*_3_) or ^19^F NMR (MeCN) under identical conditions Figure 10. In Figure 10A, the red data points are the first half-life of **1** (log_10_ *t*_1/2_[**1**], *y*-axis) as a function of azole acidity, p*K*_a_(MeCN), *x*-axis. The half-lives range from 18 h to 1.7 min. Qualitative comparison is also provided by the subset of five azole catalyst activities reported by Birman,^15a^ see yellow data points, albeit for the reaction of isopropylamine with PhOAc in CDCl_3_ with DBU (**3**). The catalytic activity increases as the azole acidity is raised from benzotriazole **4c**_**H**_ (p*K*_a_(MeCN) = 22.2) reaching a maximum in the range (25 < p*K*_a_(MeCN) < 28).

The trend then inverts, with the catalytic activity reducing as the azole acidity is further raised, to reach 4-methylpyrazole **4r**_**H**_ (p*K*_a_(MeCN) = 31.2). Thus, under the conditions employed in this work, 4-iodopyrazole **4h**_**H**_ (p*K*_a_(MeCN) = 27.0; *t*_1/2_ ≈ 1.7 min) not 1,2,4-triazole **4a**_**H**_ (p*K*_a_(MeCN) = 24.6; *t*_1/2_ ≈ 8 min) is the most efficient catalyst. To investigate why the most efficient catalysis is observed for azoles of intermediate acidity (25 < p*K*_a_(MeCN) < 28), the temporal concentration profiles for each catalyst (Figure 10B–D) were analyzed in more detail. In qualitative terms, two classes of kinetic profile were apparent across the full series of azoles, with the transition occurring at p*K*_a_(MeCN) ≈ 26. For class α azoles (p*K*_a_(MeCN) < 25; **4a**_**H**_, **4c**_**H**_, **4d**_**H**_, **4e**_**H**_, Figure 9) the amide (**5**) evolution profiles are characterized by a substantial initial rate with progressive inhibition by co-evolved **6**_**H**_. For these class α azoles, the acetylated intermediate **4**_**Ac**_ was not detected at any point during the *in situ* NMR spectroscopic monitoring, and **5** and **6**_**H**_ are liberated in concert.

**Figure 11:**
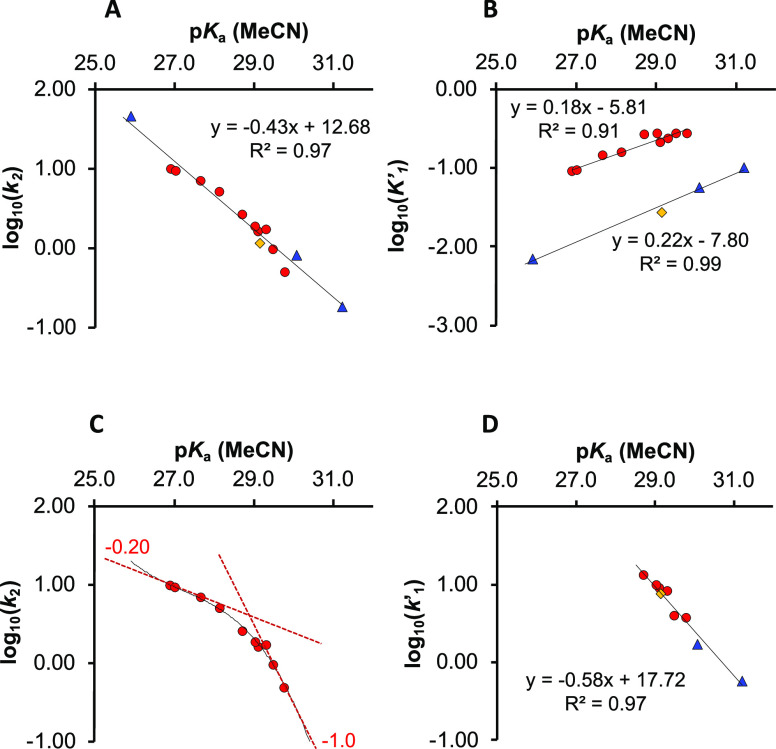
Structure–reactivity relationships between acidity (p*K*_a_(MeCN)) and key kinetic parameters for the aminolysis of **1** with **2** + **3** in MeCN-*d*_3_ (20 °C) catalyzed by class β azoles 10 mol % (p*K*_a_(MeCN) > 25). Correlations shown are of *k*_2_ (A), *K*′_1_ (B), *k*_2_ for 4-substituted pyrazoles with π-donating substituents (C), and *k*′_1_ (D). Parameters were determined by numerical fitting of kinetic profiles of **1**, **2**, and **5** in each run to the telescoped kinetic model in Figure 6, with homoconjugation of **6**_**H**_ assumed to be unaffected by azole identity; see Section S3.8.7 in the Supporting Information for full details. Red circles: 4-substituted pyrazoles with π-donating substituents (**4g**_**H**_–**4k**_**H**_; **4m**_**H**_–**4q**_**H**_). Blue triangles: pyrazoles without π-donating substituents (**4b**_**H**_, **4f**_**H**_, **4r**_**H**_). Yellow diamond: imidazole (**4l**_**H**_).

In contrast, for class β azoles (p*K*_a_(MeCN) > 25; **4b**_**H**_; **4f**_**H**_–**4r**_**H**_, Figure 9) there is no significant inhibition by co-evolved **6**_**H**_ and the *N*-acetylated intermediates (**4**_**Ac**_) accumulate sufficiently to be quantified by *in situ*^1^H NMR spectroscopy (Figure 10C). This then allows kinetic deconvolution of the catalysis by class β azoles and construction of multiple structure–activity relationships, Figure 11A–D. Rate and equilibrium coefficients for each class β azole were determined by numerical fitting of the temporal concentration profiles of **1**, **2**, and **5** to the telescoped kinetic model, see Section S3.8.7 in the Supporting Information. For azoles of intermediate acidity (**4f**_**H**_–**4j**_**H**_; p*K*_a_(MeCN) = 25.9–28.2), independent values for *k*′_1_ and *k*′_–1_ could not be obtained by this method. Instead, values for *K*′_1_ and *k*_2_ were obtained by imposing the assumption of a rapid pre-equilibrium. For weakly acidic azoles (**4k**_**H**_–**4r**_**H**_; p*K*_a_(MeCN) > 28.2), however, numerical fitting led to independent values for *k*′_1_, *k*′_–1_, and *k*_2_. Imposing constraints of either a rapid pre-equilibrium (*k*′_–1_ ≫ *k*_2_) or irreversibility (*k*′_–1_ ≪ *k*_2_) for these azoles led to significantly poorer fits, suggesting that all three processes are kinetically significant.

Linear correlations (*R*^2^ > 0.91) between the stability of the *N-*acyl intermediate, log_10_(*K*′_1_), and the Brønsted acidity of the azole, p*K*_a_(MeCN), reveals two subsets of the class β azoles, Figure 11B. Pyrazoles **4b**_**H**_, **4f**_**H**_, and **4r**_**H**_, and imidazole **4l**_**H**_ all have systematically smaller equilibrium constants, *K*′_1_, than pyrazoles of comparable acidity that bear a π-donating substituent at the 4-position. This effect is analogous, albeit smaller, to the impact of π-donating substituents in acetic anhydride hydrolysis catalyzed by 4-substituted pyridines, and likely reflects resonance stabilization of the *N*-acyl intermediate.^34a^ Partitioning the class β azoles into the two subsets aids in the interpretation of the correlation between azole acidity, p*K*_a_(MeCN), and the kinetics of aminolysis of the *N*-acetylated intermediate, log_10_(*k*_2_). For azoles with π-donating substituents, the correlation displays a distinct curvature, with limiting slopes of approximately −1.0 and −0.2 at the least (**4q**_**H**_) and most acidic (**4g**_**H**_) ends of the scale, respectively, Figure 11C.

Jencks analyzed the kinetics of general base-catalyzed aminolyses of *N*-acetyl imidazole **4l**_**Ac**_ and 1-acetyl-1,2,4-triazole **4a**_**Ac**_, in buffered aqueous solution.^34b,34c^ In both reactions, there were inflections in correlations between log_10_(*k*_cat_) and the Brønsted basicity of the general base catalyst, with the slope tending toward +1.0 for the weakest bases and plateauing at about +0.2 for the strongest. The inflections were interpreted as arising from changes in the identity of the rate-determining transition state. It was proposed that elimination, or concerted deprotonation-elimination of a tetrahedral anion, was rate-determining for the weakest bases, whereas diffusive encounters between a tetrahedral zwitterion and the general base catalyst were rate-limiting for the strongest bases.

**Scheme 3:**
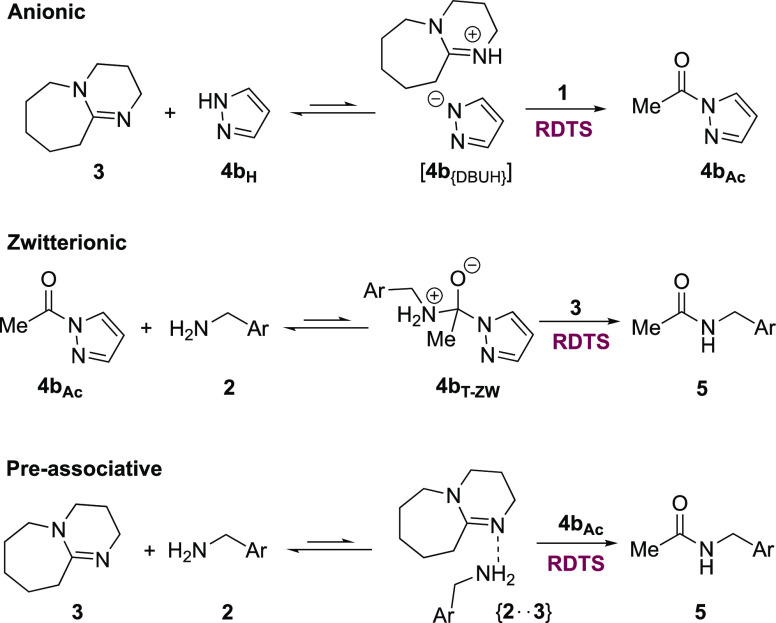
Selected Rate-Determining Transition States (RDTS) Considered for **4b**-Catalyzed Aminolysis

An inverted but otherwise analogous switch in the kinetic regime may underpin the data in Figure 11C. The limiting slope of −1.0 for *N*-acetylated adducts derived from weakly acidic azoles (e.g., **4b**_**H**_) would then correspond to fully rate-determining azolate expulsion from a tetrahedral anion, or to concerted deprotonation-elimination from the preceding tetrahedral zwitterion (**4**_T-ZW_, Scheme 3).

In either case, the near-unity correlation requires a well-advanced (product-like) rate-determining transition state with significant C–N_azole_ cleavage. The rate-determining transition state for azoles of lower p*K*_a_(MeCN), where the lifetime of the tetrahedral zwitterion **4**_**T-ZW**_ in MeCN would be expected to be considerably shorter than the timescale for diffusion, is less clear. Within the mechanistic framework outlined by Jencks,^34^ the limiting slope of approximately −0.2 may arise from rate-determining mass transport, or proton transfer, or a nuanced regime in which these processes are competitive with elimination. Moreover, the data do not preclude for example pre-associative addition, of {**2**··**3**} to **4**_**Ac**_, being rate-determining, Scheme 3.

Overall, the empirical relationship in Figure 10A is readily rationalized by changes in the catalyst speciation that arise from the modulation of the acidity of the azole. The deprotonation of highly acidic azoles (left-hand end of *x-*axis in Figure 10A) affords weakly Lewis-basic azolate anions. These generate low concentrations of highly reactive *N*-acetylated intermediate **4**_**Ac**_, with the dominant catalyst speciation being the azolate anion, and the catalytic efficiency low. Reducing the acidity of the azole stabilizes the *N*-acylated intermediate, increasing its steady-state population, and for azoles of intermediate acidity (central section of *x-*axis in Figure 10A) the highest catalytic efficiency is attained. As the acidity of the azole is further reduced (right-hand end of *x-*axis in Figure 10A) so is the catalytic efficiency due to increased off-cycle speciation as **4**_**H**_ and the reduced reactivity of the on-cycle intermediate **4**_**Ac**_.

### 2.12 Structural Insight from Heavy Atom KIEs

To add structural texture to the relationships in Figure 11, selected heavy-atom kinetic isotope effects (KIEs) in the **4a**_**H**_**-** and **4b**_**H**_-catalyzed aminolysis of **1** with **2** and **3** in MeCN (regimes I and III), were measured by intermolecular competition. These azoles were chosen because their catalytic kinetics were well characterized and because the difference in their acidities (Δp*K*_a_(MeCN) = 5.5) is such that the rate-determining transition states for the aminolysis of the corresponding *N*-acetylated adducts were expected, on the basis of Figure 11, to differ significantly in structure.

The carbonyl ^12^C/^13^C KIE for regime I was determined by *in situ*^1^H NMR spectroscopic analysis of a mixture of [^13^CO]-**1**/[^13^CH_3_]-**1** under standard conditions,^35^ see Section S4.2 in the Supporting Information. The relative isotope effect ^12/13^*k*_CO_ ≈ *k*_13CH3_/*k*_13CO_ = 1.041(2) was extracted by nonlinear regression of the isotopomer ratio^36^*R* = [^13^CO-**1**]/[^13^CH_3_–**1**]. The amine ^14^N/^15^N KIE was determined by *in situ*^19^F NMR spectroscopic analysis of [^15^N]-**2**/*meta*-deuterated **2** ([Ar-*d*_1_]-**2**).^37^ The relative inverse isotope effect *k*_14N_/*k*_15N_ = 0.979(5) was extracted by nonlinear regression of the isotopomer ratio *R*, and then normalized for the independently determined aryl ^1^H/^2^H KIE, see Section S4.3 in the Supporting Information.

The closely balanced rates of formation (*k*′_1_), phenolysis (*k*′_–1_), and aminolysis (*k*_2_) of **4b**_**Ac**_ under regime III result in a weighted and thus conversion-dependent ^12^C/^13^C KIE. Consequently, only the ^14^N/^15^N KIE was measured for catalysis by pyrazole **4b**_**H**_ under regime III, affording a normalized value of ^14/15^*k*_NH2_ = 0.976(3). The direct determination of KIEs from the stoichiometric aminolyses of **4a**_**Ac**_ and **4b**_**Ac**_ proved impractical due to their high reactivity.

To the best of our knowledge, heavy-atom KIEs for acyl transfer have only been determined in protic media.^38^ and we thus evaluated theoretical ^14/15^*k*_N_ and ^12/13^*k*_C_ KIEs for a broad range of saddle-point structures, see Section S7.2 in the Supporting Information. For catalysis by **4a**_**H**_, these were initially located on the PBE0+GD3BJ/6–311+G(d,p)/IEFPCM(MeCN) surface. Theoretical KIEs were then computed for each relative to [^13^CO-**1**]/[^13^CH_3_-**1**] and [^15^N]-**2**/**2**, using the Bigeleisen-Mayer equation (*T* = 293.15 K; 20 °C), linearly scaled harmonic frequencies (λ_zpve_ = 0.98), and Bell’s one-dimensional quantum-mechanical tunneling correction.^39^ Equilibrium isotope effects for zwitterion generation (**4a**_**T-ZW**_; analogous to **4b-**_**T-ZW**_ in Scheme 3) were computed analogously, without tunneling correction. For all saddle-point structures both KIEs were computed using a further seven KS-DFT methodologies, spanning different basis sets (6-31+G(d,p), 6-311++G(2d,p), cc-pVTZ, def2-TZVP), solvation models (SMD(MeCN)), and exchange–correlation functionals (ωB97X-D, M06-2X).^40^ The full set of results from these calculations are shown in Section S7.2 in the Supporting Information. Analogous saddle-point structures (plus the tetrahedral zwitterion intermediate **4b**_**T-ZW**_) for **4b**_**H**_-catalyzed aminolysis (SI) were obtained on the PBE0+GD3BJ/6-311+G(d,p)/IEFPCM(MeCN) surface alone, and theoretical ^14^N/^15^N KIEs calculated in the standard manner (see SI).

All of the conventional transition state models located for **4a**_**H**_-catalyzed aminolysis afforded theoretical KIEs that were essentially invariant across the eight methods, allowing a nuanced evaluation. The average KIEs, with the uncertainty in each KIE estimated from the corresponding standard deviation, were compared with the experiment. While some individual transition state models provide a good description of either the ^12^C/^13^C or the ^14^N/^15^N KIE measured under regime I (catalysis by **4a**_**H**_), no single^41^ transition state provided a description fully consistent with both, see Section S7.2 in the Supporting Information.

**Figure 12:**
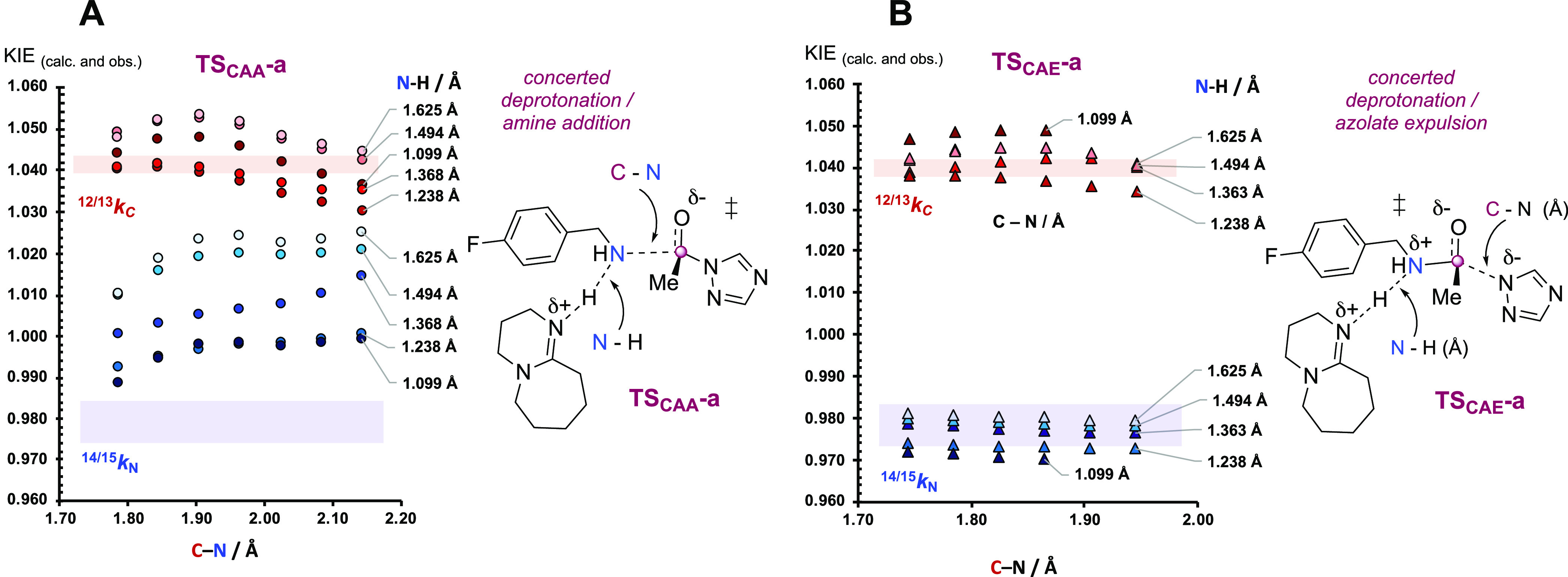
KIEs (^14/15^*k*_NH2_; ^13/12^*k*_CO_) calculated by Schramm-type analysis of (A) amine attack (**TS**_**CAA**_**-a**) versus (B) azolate expulsion (**TS**_**CAE**_**-a**) for catalysis by **4a** in MeCN (regime I, Figure 2). The shaded bands indicate the experimental KIE values within ranges of estimated errors (^14/15^*k*_NH2_ = 0.979(5)) and ^12/13^*k*_CO_ = 1.041(2). See Section S7.2 in the Supporting Information for the KIEs (^14/15^*k*_NH2_; ^13/12^*k*_CO_) calculated for eight other transition states involving **4a** and **4b**, plus EIEs (^14/15^*K*_NH2_; ^13/12^*K*_CO_) for the generation of zwitterions **4a**_**T-ZW**_ and **4b**_**T-ZW**_.

Nonetheless, the calculations identified that: (i) the large inverse ^14^N/^15^N KIE (^14/15^*k*_NH2_ = 0.979(5)) rules out transition states in which the C–N (amine) bond is not already fully formed, suggesting azole elimination and/or proton transfer is rate-determining; and (ii) the large normal ^12^C/^13^C KIE (^12/13^*k*_CO_ = 1.041(2)) suggests there is C–N (azole) bond cleavage in the rate-determining transition state, albeit without defining the extent of this. These features are captured in Schramm-type^42^ analyses of a continuum of constrained transition state models emulating amine attack (**TS**_**CAA**_**-a**, Figure 12A) and azolate expulsion (**TS**_**CAE**_**-a**, Figure 12B), both with accompanying concerted proton transfer. Of these, only the concerted, general base-catalyzed decomposition of **4a**_**T-ZW**_ by DBU (**3**), i.e., **TS**_**CAE**_**-a**Figure 12B, afforded a range of models giving KIEs in agreement with experimental values determined under regime I.

For regime III, the slightly more inverse amine KIE (^14/15^*k*_NH2_ = 0.976(3)) and lower nucleofugacity of the azolate (p*K*_a_(MeCN), **4b**_**H**_ = 30.1) suggests that proton transfer from the zwitterion (**4a**_**T-ZW**_) to DBU (**3**) is complete, and elimination may proceed *via* an *O*-coordinated tetrahedral anion, see Section S7.2 in the Supporting Information for further discussion.

## 3 Conclusions

The aminolysis of *p*-fluorophenyl acetate **1** by *p*-fluorobenzyl amine **2**, with DBU (**3**) as an auxiliary base, has been used to explore the kinetics and mechanism of acyl transfer catalysis by protic azoles (**4**_**H**_).^3h,5b,15^*In situ* and variable-ratio stopped-flow ^1^H and ^19^F NMR spectroscopy provided compelling data for anionic Lewis base n−π* catalysis via *N*-acylated azole intermediates (**4**_**Ac**_). While all evidence points to a single overarching mechanism, Figure 5, a strikingly diverse array of limiting kinetic regimes emerges from remarkably similar conditions. Indeed, the identity of the auxiliary base, the solvent, and the azole, all strongly influence the evolution of catalysis.

Three limiting regimes (I, II, III) have been identified for catalysis by protic azoles (**4**_**H**_), Figure 5. The regimes are distinguished not only by their kinetics but also by their very different sensitivities to changes in reaction temperature. This feature arises from the steps that generate and then consume the *N*-acylated azole intermediate (**4**_**Ac**_) proceeding *via* microscopically, but not necessarily kinetically, distinct mechanisms. The diversity of the kinetics of azole-catalyzed aminolysis has previously resulted in several mechanistic aspects remaining ambiguous or being overlooked altogether. A number of these features have been identified and can now be rationalized.

First, distinct changes in the reaction profile between different solvents do not necessarily solely reflect differences in the extent of product inhibition. These changes can also arise from catalysis involving different rate-determining transition states, see for example regimes I (MeCN) and II (THF), eqs 4 and 5. Second, a sufficiently strong auxiliary base, e.g., DBU (**3**) is required for turnover, and it serves two roles. It ionizes the protic azole pre-catalyst (**4**_**H**_)^15a^*and* promotes the aminolysis of the *N*-acylated azole intermediate (**4**_**Ac**_) by general Brønsted base catalysis. Although increasing base strength will not necessarily lead to an increase in catalytic efficiency, bifunctional bases, e.g., PMDBD (3,3,6,9,9-pentamethyl-2,10-diazabicyclo[4.4.0]dec-1-ene, **7**), can accelerate the aminolysis of **4**_**Ac**_ by tautomeric catalysis, leading to significant changes in catalyst speciation, Figure 7D. Thirdly, under otherwise constant conditions, there is a qualitatively parabolic relationship between azole acidity and empirical catalytic activity, Figure 10A. This is a natural consequence of the approximate correlation between Lewis and Brønsted basicity across a comparable series of azolate anions, and associated changes in catalyst speciation. However, when only sparsely sampled (see e.g., the yellow symbols in Figure 10A) the underlying relationship between azole p*K*_a_ and catalytic efficiency is intractable.^15a^ Similar bell–curve relationships have been suggested for *N,N*-dialkylaminopyridine catalysts in the acylation of alcohols by carboxylic acid anhydrides,^10c^ albeit without explicit evidence for a fundamental shift in catalyst speciation, or for the expected onset of kinetic saturation in the acyl donor.

The general features noted above lead to important practical implications for the development of azole anion Lewis base n−π* catalysis. A simple but fundamental point is that no single azole catalyst will be optimal for acyl group transfer in general: the position of the maximum activity (lowest log_10_ *t*_1/2_) in Figure 10A will vary with the nature of both the acyl donor and acyl acceptor, as well as the auxiliary base. For the direct aminolysis of weakly activated esters such as acetate (**1**) studied herein, the analysis has identified that 4-iodo-pyrazole **4h**_**H**_ (Figure 9) is around five times more active than 1,2,4-triazole **4a**_**H**_, the previously most effective simple Lewis base catalyst.^5b,15a,15b^

Moreover, apparently minor changes in catalyst acidity, auxiliary base structure, and the reaction medium can induce significant changes in catalyst speciation, kinetic regime, and susceptibility to product inhibition. This is significant because common empirical measures of catalytic activity may not be directly comparable, between systems, or at different conversions. The two key steps in azole-catalyzed aminolysis are the formation and then consumption of the *N*-acyl intermediate **4**_**Ac**_. These steps have significantly different activation parameters, Figure 8, Table 1, and thus, depending upon the kinetic regime, the efficiency of catalytic acyl transfer may be quite sensitive to temperature, or not at all. Consequently, the best optimization strategy for outcompeting unselective uncatalyzed background reactions may differ from azole to azole, from solvent to solvent, and from base to base.

## Supporting Information Available

Additional discussion, experimental procedures, characterization data, kinetic simulations using numerical methods, DOSY analyses, titration models and data, Hammett correlations, KIEs, NMR spectra, computational methods, and calculations (PDF)
