# Supplementary material for: Kinetics and Mechanism of Azole n−π*-Catalyzed Amine Acylation
Source: J Am Chem Soc. 2023 Aug 1;145(32):18126–40. doi: 10.1021/jacs.3c06258 (PMC10436283; doi:10.1021/jacs.3c06258)
Supplement: Supplementary file 1 — ja3c06258_si_001.pdf [file ja3c06258_si_001.pdf]

Supporting Information

Kinetics and Mechanism of Azole n-π* Catalyzed Amine Acylation

Harvey J. A. Dale,† George R. Hodges,‡ and Guy C. Lloyd-Jones†*

† EaStChem, University of Edinburgh, Joseph Black Building, David Brewster Road, Edinburgh, EH9 3FJ, U.K.

‡ Jealott's Hill International Research Centre, Syngenta, Bracknell, Berkshire RG42 6EY, U.K.

Email: *guy.lloyd-jones@ed.ac.uk

S1. General Experimental Methods 4
S1.1. NMR characterisation *4*
S1.2. Chromatography *4*
S1.3. Reagents and solvents *5*
S1.4. Infrared spectroscopy *5*
S1.5. Mass spectrometry *5*
S1.6. Melting points *6*
S1.7. Air-sensitive reactions *6*
S1.8. Miscellaneous *6*
S2. Synthetic Procedures 7
S2.1. Salts *7*
S2.2. 4-Aryl-pyrazoles *11*
S2.3. Isotopically labelled substrates *22*
S2.4. N-Acetylated azoles *32*
S3. Reaction Monitoring Details 34
S3.1. General considerations *34*
S3.2. Manually assembled reactions *35*
S3.3. Variable Ratio Stopped-flow NMR (VR-SF-NMR) monitoring *37*
S3.4. Longitudinal relaxation times (T_1) *46*
S3.5. Numerical kinetic analysis *52*
S3.6. Graphical kinetic analysis *52*
S3.7. Steady-state kinetic analysis *53*
S3.8. Reaction Monitoring and Kinetic Analyses *59*
S3.8.1 ^{1}H and ^{19}F NMR monitoring: example spectra *59*
S3.8.2 Regime I Kinetics *60*
S3.8.3 Regime II Kinetics *75*
S3.8.4 Regime III Kinetics *78*
S3.8.5 Regime IV Kinetics *95*
S3.8.6 Isothermal kinetic studies of N-acetylated azoles *97*
S3.8.7 Variable-temperature kinetic studies *101*

S3.8.8 Structure-activity relationships: kinetic data *107*

S4. Intermolecular Competitions 128

S4.1. Competitive Hammett relationships *128*

S4.2. $^{12}C/^{13}C$ KIE *134*

S4.3. $^{14}N/^{15}N$ KIE *135*

S5. ^{1}H DOSY NMR 139

S5.1. Overview *139*

S5.2. Data acquisition *140*

S5.3. Relevant theory *141*

S5.4. Data analysis *142*

S5.5. Data analysis - validation *142*

S5.6. Discussion and figures *149*

S6. $^{1}H/^{19}F$ NMR Titrations 154

S6.1. Overview *154*

S6.2. Typical titration *154*

S6.3. Equilibrium models *155*

S6.4. Fitted isotherms *164*

S7. Computations 166

S7.1. General considerations *166*

S7.2. Heavy atom kinetic isotope effects *167*

S7.3. pK_a calculations *190*

S8. NMR spectra 194

References 253

S1. General Experimental Methods

S1.1. NMR characterisation

Unless otherwise stated, all ^{1}H, ^{2}H, $^{13}C\{^{1}H\}$, ^{15}N, ^{19}F, and $^{29}Si\{^{1}H\}$-INEPT NMR spectra obtained for characterisation were acquired on a Bruker Ascend 400 MHz NMR spectrometer fitted with a broadband direct-detect Cryoprobe Prodigy unit at 300 K (27 °C) (^{1}H 400 MHz; ^{2}H 61 MHz; $^{13}C\{^{1}H\}$ 101 MHz; ^{15}N 41 MHz; ^{19}F 377 MHz; ^{29}Si 80 MHz). All ^{1}H chemical shifts are quoted in parts per million (ppm) relative to the residual solvent peak: MeCN-d_2 (δ_H = 1.97 ppm), DMSO-d_5 (δ_H = 2.50 ppm), $CHCl_3$ (δ_H = 7.26 ppm). All ^{13}C chemical shifts are quoted in parts per million (ppm) relative to the deuterated solvent peak: MeCN-d_3 (δ_C = 117.3 ppm), DMSO-d_6 (δ_C = 39.5 ppm), $CDCl_3$ (δ_C = 77.2 ppm). All $^{13}C\{^{1}H\}$ resonances are assumed to be singlets unless otherwise stated.

All coupling constants, *J*, are quoted in Hz; coupling constants that did not match as a result of digitisation are reported as rounded averages. Multiplicities arising from coupling to spin ½ nuclei are indicated as s (singlet), brs (broad singlet), d (doublet, 1:1), brd (broad doublet, 1:1), t (triplet, 1:2:1), q (quartet, 1:3:3:1), quint. (quintet, 1:4:6:4:1), sext. (sextet, 1:5:10:10:5:1), sept. (septet, 1:6:15:20:15:6:1), m (multiplet), or combinations thereof. Multiplicities arising from coupling to quadrupolar nuclei are indicated explicitly by the splitting and relative intensities of the individual peaks, e.g. 1:1:1 t (triplet, I = 1), 1:1:1:1 q (quartet, I = 3/2). Where necessary, ^{1}H and $^{13}C\{^{1}H\}$ NMR assignments were confirmed using a combination of 2D ^{1}H correlated spectroscopy (COSY), 2D ^{1}H–^{13}C heteronuclear multiple-bond correlation spectroscopy (HMBC), 2D ^{1}H–^{13}C heteronuclear single quantum coherence (HSQC) and computational prediction (KS-DFT; GIAO/PBE0+GD3BJ/6-311+G(2d,p)/IEFPCM//PBE0+GD3BJ/6-311+G(d,p)/IEFPCM). All NMR spectra were analysed with Mestrenova (Version 11).

S1.2. Chromatography

Analytical thin-layer chromatography was performed on precoated aluminium-backed plates (Silica Gel 60 F254; Merck), and visualisation was achieved using ultraviolet light (254 nm) and/or staining with either aqueous basic potassium permanganate ($KMnO_4$) solution or ethanolic phosphomolybdic acid followed by heating. Column chromatography was performed using Geduran® Silica Gel 60 (40-63 µm; Merck).

S1.3. Reagents and solvents

Unless stated otherwise, reagents were purchased from commercial sources (Sigma Aldrich, Alfa Aesar, Acros Organics or Fluorochem) at the highest available grade, and were used without purification. All internal standards used for kinetic monitoring experiments and other quantitative purposes were of > 99% purity.

Anhydrous ethereal solvents (THF, Et_2O) were obtained by passing solvent through a column of anhydrous alumina using an Anhydrous Engineering Grubbs-type system and storing over 3 Å molecular sieves. Anhydrous non-ethereal organic solvents (MeCN, MeCN-d_3, CH_2Cl_2, $CHCl_3$, $CDCl_3$, DMSO, DMSO-d_6) were obtained by drying over activated 3 Å molecular sieves for > 48 h. Molecular sieves were activated by heating *in vacuo* (220 °C, 0.8 mbar) for > 16 h. All aqueous solutions were prepared with deionised H_2O. The removal of organic solvents *in vacuo* was typially achieved using rotary evaporator with a water bath set to 40 °C; higher temperatures (up to 60 °C) were applied only for less volatile solvents (e.g., PhMe, H_2O).

All NMR solvents were dried over activated 3Å molecular sieves, and stock solutions were typically used within several hours, but all reactions were assembled under ambient conditions. Product evolution profiles were found to be highly reproducible between individual kinetic runs, between different stock solutions, and even between different batches of **1**, **3** and/or **2**, all provided modest precautions (i.e., moderately anhydrous but otherwise standard purity solvents) were taken towards the exclusion of adventitious moisture; hydrolysis of **1** was generally minor (< 3 %) under standard catalytic conditions, without the use of a glovebox for solution preparation, and without the use of J. Young valve NMR tubes for monitoring. Kinetic profiles for the obtained independently by ^{1}H NMR monitoring (MeCN-d_3) were found to be essentially identical to those obtained by ^{19}F NMR (MeCN-h_3), discounting the existence of a significant solvent kinetic isotope effect. In principle, the measurably asynchronous evolution of **5** and **6** could arise from an initially rapid hydrolytic release of **6$_H$** from **1** – but no such significant pre-emptive hydrolysis was observed with **4a$_H$** under otherwise identical conditions (including equivalent handling of substrates and solvents).

S1.4. Infrared spectroscopy

Infrared (IR) spectra of neat compounds were recorded over the range 4000-400 cm^{-1} using a Bruker ALPHA™ ATR-FTIR spectrometer; peaks are reported in cm^{-1}. Only characteristic peaks are reported.

S1.5. Mass spectrometry

Electron impact (EI^+) spectra were recorded on a ThermoElectron MAT 900 mass spectrometer using a double focusing sector field mass analyser. Electrospray ionisation (ESI^+) spectra were recorded on a Bruker ESI Micro-Tof mass spectrometer using a time-of-flight mass analyser. Data are reported in the form of m/z.

S1.6. Melting points

Melting points (mp) were determined on a Griffin capillary apparatus in capillary melting point tubes and are uncorrected.

S1.7. Air-sensitive reactions

Air-sensitive synthetic procedures were performed under an atmosphere of anhydrous nitrogen gas (N_2) using standard Schlenk techniques, on a vacuum line attached to a double manifold equipped with an oil pump (0.8 mbar). Glassware used for such procedures was oven dried (220 °C) for at least 16 h, and immediately (< 20 s) placed under high vacuum to cool once removed from the oven. Degassed organic solvents were prepared in Strauss flasks by standard freeze-pump-thaw procedures (> 3 cycles); degassed H_2O was prepared in a Schlenk flask by purging thoroughly with a continuous stream of N_2 for > 24 h.

S1.8. Miscellaneous

Room temperature (rt) refers to 20 $\pm$ 2 °C. Temperatures of –20 °C to 0 °C for overnight reactions were obtained using an immersion cooler (LabPlant AP100). Reactions involving heating (> 22 °C) were performed using DrySyn blocks and a contact thermocouple (vials) or a silicone oil bath (Schlenk flasks, round-bottomed flasks).

All glass microsyringes were cleaned (internal barrel, needle and plunger) as soon as possible after use, using a sequence of HCl (2 M), H_2O, MeOH and Me_2CO, and dried *in vacuo* overnight; all volumetric glassware was cleaned using the same sequence of washes, but dried at ambient temperature. Schott reagents bottles for stopped-flow NMR experiments were cleaned similarly and oven-dried (220 °C, > 24 h). NMR tubes containing exclusively organic samples were cleaned using a sequence of H_2O, MeOH and Me_2CO, and dried in *vacuo*. NMR tubes contaminated with solid residues that proved unsusceptible to this routine were subject to preliminary cleaning with aqua regia (3:1 HCl: HNO_3), followed by the standard protocol.

Schlenk flasks used for Pd-catalysed Suzuki-Miyaura couplings were cleaned with aqueous HNO_3 (3 M), KOH (1 M) in ^{i}PrOH:H_2O (9:1), H_2O, Me_2CO, and then oven dried (220 °C, > 24 h); magnetic stirrers were cleaned with HNO_3 (3 M), H_2O, Me_2CO and oven-dried (220 °C, > 24 h). All NMR tubes used to analyse crude products generated by Pd-catalysed Suzuki-Miyaura couplings were subject to preliminary cleaning with aqua regia, irrespective of their physical appearance.

S2. Synthetic Procedures

S2.1. Salts

Tetra-n-butylammonium 1,2,4-triazolate 4a$_{\{nBu4\ N\}}$

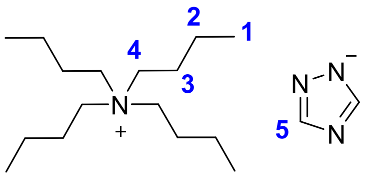

1,2,4-Triazole **4a$_H$** (0.691 g, 10.0 mmol) and tetra-n-butylammonium hydroxide 30-hydrate ($^nBu_4N(OH).30H_2O$; 8.00 g, 10.0 mmol) were dissolved in MeOH (50 mL), and the resulting solution was stirred under ambient conditions for 15 min. After this time the solvent was removed under reduced pressure to afford a viscous, colourless oil; the oily residue was suspended in PhMe (150 mL) and dried azeotropically using a standard Dean-Stark apparatus. The PhMe solvent was then removed under reduced pressure (20 mbar, 50 °C) to afford a viscous oil, which crystallised spontaneously upon cooling to room temperature to afford a pale yellow solid. The salt was dried vigorously *in vacuo* for 72 h (0.8 mbar, 50 °C) and then transferred to a nitrogen-filled glovebox for storage and handling. The ^{1}H and ^{13}C and NMR spectra are consistent with the literature.

^{1}H NMR (400 MHz, CD_3CN) δ_H / ppm: 0.95 (12H, t, $^3J_{H1\text{-}H2}$ = 7.4 Hz, [C1]H_3), 1.33 (8H , sext., $^3J_{H1\text{-}H2}$ = $^3J_{H2\text{-}H3}$ = 7.4 Hz, [C2]H_2), 1.52 – 1.64 (8H, m, [C3]H_2), 3.04 – 3.17 (8H, m, [C4]H_2), 7.65 (2H, s, [C5]-*H*).

^{13}C NMR (101 MHz, CD_3CN) δ_C / ppm: 13.8 (C1), 20.3 (1:1:1 t, $^3J_{C\text{-}N}$ = 1.6 Hz, C2), 24.3 (C3), 59.3 (1:1:1 t, $^1J_{C\text{-}N}$ = 2.9 Hz, C4), 150.5 (C5).

Tetra-n-butylammonium 4-F-phenol-4-F-phenolate {6-6$_H$}$_{\{nBu4N\}}$

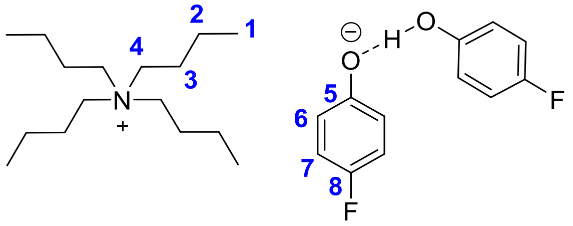

Under ambient conditions, tetra-n-butylammonium hydroxide 30 hydrate ([^{n}Bu$_4$N][OH].30H_2O; 3.57 g, 4.46 mmol, 1.00 equiv.) and 4-fluorophenol **6$_H$** (1.00 g, 8.92 mmol, 2.00 equiv.) were assembled in MeOH (20 mL), with the resulting solution stirred for 5 min. The MeOH was then removed under reduced pressure to afford a yellow solid, which was dried azeotropically using PhMe (3 x 30 mL, 50 °C) and subsequently *in vacuo* (0.8 mbar, 20 °C, 72 h). A crystalline sample suitable for X-ray diffraction was prepared by vapour diffusion from MeCN/Et$_2$O.

^{1}H NMR (400 MHz, CD$_3$CN) δ_H / ppm: 0.95 (12H, t, $^3J_{H1-H2}$ = 7.4 Hz, [C1]H_3), 1.33 (8H , sext., $^3J_{H1-H2}$ = $^3J_{H2-H3}$ = 7.4 Hz, [C2]H_2), 1.50 – 1.64 (8H, m, [C3]H_2), 3.00 – 3.11(8H, m, [C4]H_2), 6.49 – 6.56 (4H, m, [C6]H), 6.67 – 6.75 (4H, m, [C7]H).

^{13}C NMR (101 MHz, CD$_3$CN) δ_C / ppm: 13.8 (C1), 20.3 (1:1:1 t, $^3J_{C-N}$ = 1.5 Hz, C2), 24.3 (C3), 59.3 (1:1:1 t, $^1J_{C-N}$ = 2.5 Hz, C4), 115.7 (d, $^2J_{C-F}$ = 21.9 Hz, C7), 117.9 (d, $^3J_{C-F}$ = 7.5 Hz, C6), 154.1 (d, $^1J_{C-F}$ = 227 Hz, C8), 162.7 (s, C5).

^{19}F NMR (377 MHz, CD$_3$CN) δ_F / ppm: -134.98 (tt, $^4J_{H-F}$ = 4.9 Hz, $^3J_{H-F}$ = 8.9 Hz, Ar-F).

[DBUH$^+$][PF$_6^-$]

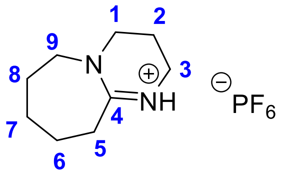

1,8-Diazabicyclo[5.4.0]undec-7-ene **3** (DBU; 1.0 equiv.) was dissolved in CH_2Cl_2 (50 mL) and the resulting solution was cooled to 0 °C; once cooled, ethereal hydrochloric acid ($HCl_{(Et2O)}$; 2.0 M, 1.2 equiv.) was added. The solution was stirred for 5 min and then combined with an aqueous solution (30 mL) of sodium hexafluorophosphate ($NaPF_6$; 3.0 g, 1.5 equiv.) to afford a biphasic mixture, which was stirred vigorously at ambient temperature under an inert atmosphere of N_2. After 16 h the organic layer was isolated and the aqueous phase extracted with CH_2Cl_2 (3 x 10 mL); the organic extracts were washed with deionised water (10 mL) then combined and the solvent removed under reduced pressure to afford a pale yellow solid, which was dried vigorously *in vacuo* for 48 h (0.8 mbar, 20 °C). The stoichiometry of the salt (1.0:1.0, DBUH$^+$:PF$_6^-$) was confirmed by multinuclear (^{1}H and ^{19}F) NMR spectroscopy, using 1-fluoronaphthalene as an internal standard and an extended relaxation delay (d1 = 10 s).

^{1}H NMR (400 MHz, CD_3CN) δ_H / ppm: 1.61 – 1.78 (6H, m, [C6]H_2 + [C7]H_2 + [C8]H_2), 1.97 (2H, quint., $^3J_{H1\text{-}H2}$ = $^3J_{H2\text{-}H3}$ = 5.9 Hz, [C2]H_2), 2.54 – 2.60 (2H, m, [C5]H_2), 3.22 – 3.27 (2H, m, [C3]H_2), 3.46 (2H, t, $^3J_{H1\text{-}H2}$ = 5.9 Hz, [C1]H_2), 3.49 – 3.56 (2H, m, [C9]H_2), 7.43 (1H, s, NH).

^{13}C NMR (101 MHz, CD_3CN) δ_C / ppm: 19.8 (C2), 24.3 (C6), 26.8 (C7), 29.4 (C8), 34.0 (C5), 39.2 (C3), 49.4 (C1), 55.3 (C9), 167.1 (C4).

^{19}F NMR (377 MHz, CD_3CN) δ_F / ppm: -72.9 (d, $^1J_{P\text{-}F}$ = 707 Hz, PF_6).

^{31}P NMR (162 MHz, CD_3CN) δ_P / ppm: -144.6 (sept., $^1J_{P\text{-}F}$ = 707 Hz, PF$_6$).

[DBUH$^+$][BPh$_4^-$]

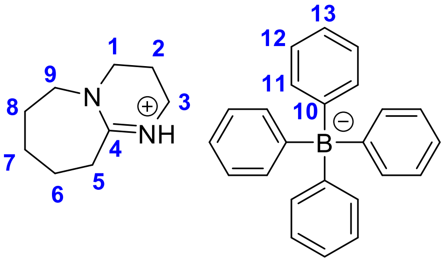

1,8-Diazabicyclo[5.4.0]undec-7-ene **3** (DBU; 0.50 mL, 3.4 mmol) was dissolved in 2.0 M aqueous hydrochloric acid ($HCl_{(aq)}$, 15 mL), and the resulting solution was vigorously stirred. An aqueous solution (15 mL) of sodium tetraphenylborate ($NaBPh_4$; 1.75 g, 5.1 mmol) was added gradually, leading instantaneously to the precipitation of a white solid. Once addition was complete, the suspension was stirred vigorously at ambient temperature under an insert atmosphere of N_2; after 1 h the precipitate was isolated by filtration and washed extensively with deionised H_2O. The crude solid was suspended in refluxing $CHCl_3$ and dissolved by gradual addition of CH_3CN; cooling induced the formation of a white crystalline solid (needles), which was subsequently isolated by vacuum filtration and dried vigorously *in vacuo* (0.8 mbar, 20 ^{o}C) for 48 h (1.19 g, 74 %).

^{1}H NMR (400 MHz, CD_3CN) δ_H / ppm: 1.61 – 1.78 (6H, m, [C6]H_2 + [C7]H_2 + [C8]H_2), 1.97 (2H, quint., $^3J_{H1-H2}$ = $^3J_{H2-H3}$ = 5.9 Hz, [C2]H_2), 2.54 – 2.60 (2H, m, [C5]H_2), 3.19 – 3.24 (2H, m, [C3]H_2), 3.41 (2H, t, $^3J_{H1-H2}$ = 5.9 Hz, [C1]H_2), 3.45 – 3.51 (2H, m, [C9]H_2), 6.85 (4H, t, $^3J_{H12-H13}$ = 7.3 Hz, [C13]H), 7.00 (8H, t, $^3J_{H11-H12}$ = 7.3 Hz, [C11]H), 7.25 – 7.31 (8H, m, [C12]H).

^{13}C NMR (101 MHz, CD_3CN) δ_C / ppm: 19.8 (C2), 24.3 (C6), 26.9 (C7), 29.4 (C8), 34.0 (C5), 39.2 (C3), 49.4 (C1), 55.3 (C9), 122.8 (s, C13), 126.6 (1:1:1:1 q, $^2J_{C-B}$ = 2.8 Hz, C11), 136.7 (1:1:1:1 q, $^3J_{C-B}$ = 1.3 Hz, C12), 164.8 (1:1:1:1 q, $^1J_{C-B}$ = 42.9 Hz, C10), 167.1 (C4).

^{11}B NMR (126 MHz, CD_3CN) δ_B / ppm: -6.6 Hz (s, *B*Ph$_4$).

S2.2. 4-Aryl-pyrazoles

General procedure I

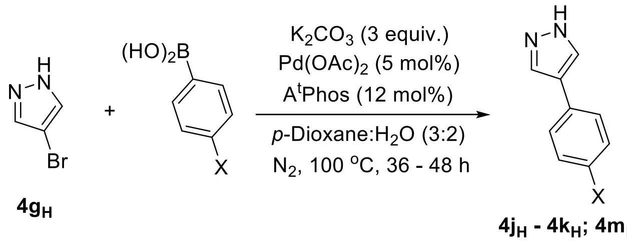

$4g_H$ **$4j_H$ - $4k_H$; $4m_H$ - $4q_H$**

An oven-dried Schlenk flask containing a PTFE-coated magnetic stirrer bar was charged under air with 4-bromopyrazole **$4g_H$** (0.176 g, 1.2 mmol, 1.0 equiv.), the appropriately 4-substituted aryl boronic acid ($^{4\text{-}X}$Ar-B(OH)$_2$; **8_X**, 2.4 mmol, 2.0 equiv.), anhydrous potassium carbonate (0.497 g, 3.6 mmol, 3.0 equiv.), (4-(N,N-dimethylamino)phenyl)di-tert-butyl phosphine (A^{t}Phos; 0.0381 g, 0.144 mmol, 12 mol %) and palladium(II) acetate ($Pd(OAc)_2$; 0.0134 g, 0.060 mmol, 5 mol%,). Following the addition of all solid components, the flask was carefully evacuated under high vacuum (0.8 mbar, 20 °C) for 15 min, and subsequently refilled with N_2; this sequence was repeated twice over. Under a dynamic flow of N_2, degassed *p*-dioxane (3.6 mL) was then dispensed to the flask, with stirring, followed by degassed, deionised H_2O (2.4 mL). The flask was sealed under N_2, and the reaction mixture (3:2 *p*-dioxane:H_2O) stirred at room temperature. After 15 min, the sealed flask was submerged in a pre-heated silicone oil bath (100 °C), and the reaction mixture stirred (500 rpm) for 36 – 48 h. The reaction mixture was subsequently cooled to ambient temperature, exposed to air, diluted with EtOAc (20 mL), and filtered through a celite pad. Solid residues in the flask were rinsed with further EtOAc (2 x 20 mL) and filtered. The combined filtrate was washed once with aqueous NaOH (1.0 M) and once with saturated aqueous NaCl, and dried over anhydrous $MgSO_4$. The drying agent was removed by gravity filtration and the solvent removed under reduced pressure (40°C). The crude solid was purified either by recrystallisation (THF/hexanes), flash column chromatography (silica, Et_2O or EtOAc), and/or acid-base extraction (HCl/NaOH).

General procedure II

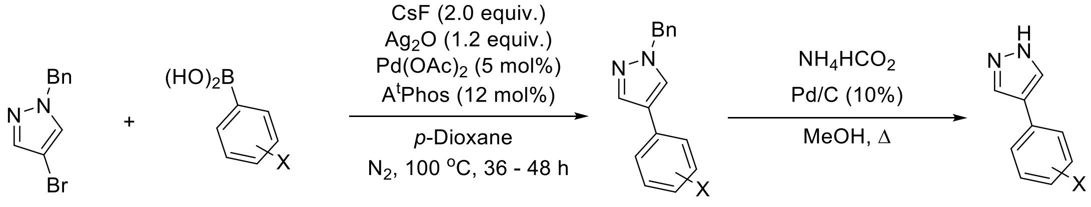

$4g_{Bn}$ **$4x_{Bn}$** **$4x_H$**

IIa (coupling): An oven-dried Schlenk flask containing a PTFE-coated magnetic stirrer bar was charged under air with N-benzyl-4-bromopyrazole **$4g_{Bn}$** (0.142 g, 0.60 mmol, 1.0 equiv.), the appropriately substituted aryl boronic acid (^{X}Ar-B(OH)$_2$; **8_X**, 1.2 mmol, 2.0 equiv.), anhydrous cesium(**I**) fluoride (0.182 g, 1.2 mmol, 2.0 equiv.), silver(**I**) oxide (0.167 g , 0.72 mmol, 1.2 equiv.), (4-(N,N-dimethylamino)phenyl)di-tert-butyl phosphine (A^{t}Phos; 0.0191 g, 0.072 mmol, 12 mol%) and palladium(**II**) acetate (Pd(OAc)$_2$; 0.0067 g, 0.030 mmol, 5 mol%). Following the addition of all solid components, the flask was carefully evacuated under high vacuum (0.8 mbar, 20 °C) for 15 min, and subsequently refilled with N_2; this sequence was repeated twice over. Under a dynamic flow of N_2, degassed, anhydrous *p*-dioxane (6.0 mL) was then dispensed to the flask, which was then sealed under N_2. After stirring at ambient temperature for 15 min, the sealed flask was submerged in a pre-heated silicone oil bath (100 °C), and the reaction mixture stirred rapidly (500 rpm) for 36 – 48 h. The reaction mixture was subsequently cooled to ambient temperature, exposed to air, diluted with EtOAc (20 mL), and filtered through a celite pad. Solid residues in the flask were rinsed with further EtOAc (2 x 20 mL), and the suspension filtered. The combined filtrate was washed once with aqueous NaOH (1.0 M) and once with saturated aqueous NaCl, and dried over anhydrous $MgSO_4$. The drying agent was removed by gravity filtration and the solvent was removed reduced pressure at 40°C. The crude solid **$4x_{Bn}$** was purified by flash column chromatography (silica, Petrol: Et_2O). Similar coupling efficiencies were observed with N-(*p*-methoxybenzyl)-4-bromopyrazole; N-tert-butyloxycarbonyl (N-Boc) protection, in contrast, proved ineffectual.

IIb (deprotection): Debenzylation of the coupled pyrazole **$4x_{Bn}$** was facilitated by catalytic transfer hydrogenation, using ammonium formate (NH_4HCO_2) as a solid, pH-neutral surrogate for H_2. A two-necked round-bottomed flask was charged with stoichiometric Pd/C (10% w/w, 0.54 mmol, 1.0 equiv.) and the appropriate 4-aryl-N-benzyl-pyrazole (0.54 mmol, 1.0 equiv.). The flask volume was evacuated under high vacuum (0.8 mbar, 20 °C) for 15 min, and subsequently refilled with N_2; this sequence was repeated twice over, and followed by the addition of anhydrous MeOH (10 mL) to form a suspension. Under a dynamic flow of N_2, excess NH_4HCO_2 (2.7 mmol, 5.0 equiv.) was added to the stirred suspension, which was subsequently heated to reflux under N_2. Reaction progress was carefully monitored by TLC (Petrol:Et_2O), with further portions of NH_4HCO_2 / Pd/C added where necessary to achieve full deprotection. Following full consumption of the substrate, the reaction mixture was cooled to room temperature and exposed to air; the Pd/C was removed by vacuum filtration through a pad of celite (EtOAc), the flask was rinsed with further EtOAc, and the washings filtered. The filtrate was then evaporated under reduced pressure to afford the corresponding 4-aryl-1H-pyrazole **$4x_H$**.

N-Benzyl-4-[(3,5-bis-trifluoromethyl)phenyl]pyrazole 4i$_{Bn}$

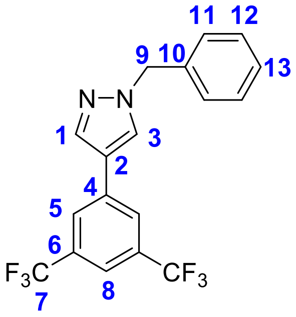

Prepared according to General Procedure II with 3,5-bis(trifluoromethyl)phenylboronic acid (0.309 g, 1.2 mmol, 2.0 equiv.). Product obtained as a yellow solid following purification by flash column chromatography (silica, 1:2 Et_2O: Petrol, Rf = 0.33; 0.199 g, 0.54 mmol, 90 %, m.p. 121 – 124 °C, [M] = $C_{18}H_{12}F_6N_2$).

^{1}H NMR (400 MHz, CD_3CN) δ_H / ppm: 5.37 (2H, s, C(9)H2), 7.32 – 7.44 (5H, m, C(11)H + C(12)H + C(13)H), 7.82 (1H, m, C(8)H), 7.99 (1H, d, $^4J_{H-H}$ = 0.8 Hz, C(1)H), 8.11 (2H, m, C(5)H), 8.17 (1H, d, $^4J_{H-H}$ = 0.8 Hz, C(3)H).

^{13}C{^{1}H} NMR (101 MHz, CD_3CN) δ_C / ppm: 55.8 (C9), 119.3 (sept, $^3J_{C-F}$ = 3.9 Hz, C8), 120.3 (C2), 123.7 (q, $^1J_{C-F}$ = 272 Hz, C7), 125.4 (m, C5), 127.9 (C11), 128.0 (C13), 128.2 (C3), 128.7 (C12), 131.5 (q, $^2J_{C-F}$ = 33.1 Hz, C6), 135.4 (C4), 137.0 (C10), 137.2 (C1).

^{19}F NMR (377 MHz, CD_3CN) δ_F / ppm: -63.50 (s, CF_3)

HRMS (ESI$^+$): [MH]+ Found 371.09710; Required 371.09774 (-0.64 ppm).

IR υ_{film} / cm^{-1}: 421, 497, 570, 606, 681, 698, 714, 776, 797, 843, 881, 902, 997, 1080, 1109, 1126, 1157, 1277, 1335, 1403, 1565, 1621.

4-Phenyl-1H-pyrazole 4m$_H$

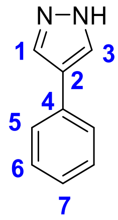

Prepared according to General Procedure I with phenyl boronic acid (0.293 g, 2.4 mmol, 2.0 equiv.). Product obtained as a white solid following recrystallisation from THF/hexane (0.082 g, 0.57 mmol, 47 %, m.p. 230 – 235 °C, [M] = $C_9H_8N_2$).

^{1}H NMR (400 MHz, CD_3CN) δ_H / ppm: 7.25 (1H, m, [C7]*H*), 7.40 (2H, m, [C6]*H*), 7.60 (2H, m, [C5]*H*), 7.92 (2H, s, [C1]H + [C3]*H*), 11.15 (1H, brs, N*H*). NH exchange leads to single time-averaged resonance for [C1]*H* and [C3]*H*.

^{13}C{^{1}H} NMR (101 MHz, CD_3CN) δ_C / ppm: 121.9 (C2), 125.4 (C5), 126.2 (C7), 128.8 (C6), 132.9 (C4). Resonances for C1 and C3 not observed in CD_3CN as a result of substantial line broadening arising from NH exchange.

HRMS (ESI$^+$): [MH]+ Found 145.07780; Required 145.07602 (1.78 ppm).

IR υ_{film} / cm^{-1}: 436, 507, 624, 692, 755, 827, 853, 878, 904, 948, 1034, 1153, 1377, 1605, 2896 (br), 3111 (br).

4-(4-Chlorophenyl)-1H-pyrazole 4k$_H$

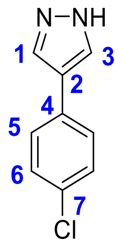

Prepared according to General Procedure I with 4-chlorophenylboronic acid (0.375 g, 2.4 mmol, 2.0 equiv.). Product obtained as a white solid following recrystallisation from THF/hexane (0.066 g, 0.37 mmol, 30 %, m.p. 180 – 182 °C, [M] = $C_9H_7ClN_2$).

^{1}H NMR (400 MHz, CD_3CN) δ_H / ppm: 7.41 (2H, m, [C6]*H*), 7.58 (2H, m, [C5]*H*), 7.92 (2H, s, [C1]*H* + [C3]*H*), 11.22 (1H, brs, NH). NH exchange leads to single time-averaged resonance for [C1]*H* and [C3]*H*.

^{13}C{^{1}H} NMR (101 MHz, CD_3CN) δ_C / ppm: 120.7 (C2), 126.9 (C5), 128.8 (C6), 131.2 (C4), 131.8 (C7). Resonances for C1 and C3 not observed in CD_3CN as a result of substantial line broadening arising from NH exchange.

HRMS (ESI$^+$): [MH]+ Found 179.03830; Required 179.03705 (1.25 ppm).

IR υ_{film} / cm^{-1}: 446, 505, 517, 628, 662, 735, 807, 833, 865, 946, 959, 1012, 1035, 1091, 1102, 1109, 1159, 1186, 1295, 1322, 1345, 1356, 1370, 1486, 1509, 2917 (br), 3110 (br).

4-(4-Methoxyphenyl)-1H-pyrazole 4p$_H$

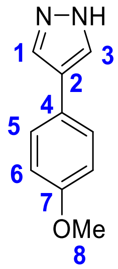

Prepared according to General Procedure I with 4-methoxyphenylboronic acid (0.365 g, 2.4 mmol, 2.0 equiv.). Product obtained as a white solid following recrystallisation from THF/hexane (0.056 g, 0.32 mmol, 27 %, m.p. 230 – 233 °C, [M] = $C_{10}H_{10}N_2O$).

^{1}H NMR (400 MHz, CD_3CN) δ_H / ppm: 3.82 (3H, s, [C8]*H*3), 6.97 (2H, m, [C6]*H*), 7.51 (2H, m, [C5]*H*), 7.84 (2H, s, [C1]*H* + [C3]*H*), 11.07 (1H, brs, N*H*). NH exchange leads to single time-averaged resonance for [C1]H and [C3]H.

^{13}C{^{1}H} NMR (101 MHz, CD_3CN) δ_C / ppm: 54.9 (C8), 114.2 (C6), 121.6 (C2), 125.5 (C4), 126.6 (C5), 158.3 (C7). Resonances for C1 and C3 not observed in CD_3CN as a result of substantial line broadening arising from NH exchange.

HRMS (ESI$^+$): [MH]+ Found 175.0880; Required 175.08659 (1.41 ppm).

IR υ_{film} / cm^{-1}: 494, 520, 528, 605, 626, 635, 659, 719, 793, 819, 863, 930, 948, 962, 1011, 1027, 1109, 1150, 1177, 1244, 1277, 1355, 1371, 1442, 1463, 1498, 1522, 1568, 2954 (br), 3133 (br).

4-[(3,5-Bis-trifluoromethyl)phenyl]-1H-pyrazole 4i$_H$

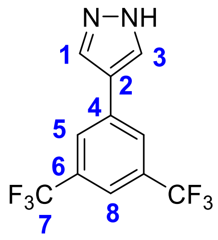

Prepared according to General Procedure IIb with N-benzyl-4-[(3,5-bis-trifluoromethyl)phenyl]pyrazole (0.200 g, 0.54 mmol, 1.0 equiv.), and used without further purification (yellow solid, 0.122 g, 0.44 mmol, 81 %, m.p. 177 – 183 °C, [M] = $C_{11}H_6F_6N_2$).

^{1}H NMR (400 MHz, CD_3CN) δ_H / ppm: 7.84 (1H, sept, $^4J_{H\text{-}F}$ = 0.8 Hz, [C8]*H*), 8.11 (2H, s, [C1]*H* + [C3]*H*), 8.16 (2H, m, [C5]*H*), 11.36 (1H, brs, N*H*). NH exchange leads to single time-averaged resonance for [C1]*H* and [C3]*H* in CD_3CN.

^{1}H NMR (400 MHz, DMSO-d$_6$) δ_H / ppm: 7.82 (1H, s, [C8]*H*), 8.24 (1H, brs, [C1]*H*/[C3]*H*), 8.30 (2H, s, [C5]*H*), 8.57 (1H, brs, [C1]*H*/[C3]*H*), 13.19 (1H, brs, N*H*). NH exchange seemingly slower in DMSO-d$_6$ than CD_3CN, leading to broad but resolved resonances for [C1]H and [C3]H.

^{13}C{^{1}H} NMR (101 MHz, DMSO-d$_6$) δ_C / ppm: 119.1 (C2), 119.1 (sept., $^3J_{C\text{-}F}$ = 3.8 Hz, C8), 123.9 (q, $^1J_{C\text{-}F}$ = 272.9 Hz, CF_3), 125.7 (m, C5), 127.7 (brs, C1/C3), 131.3 (q, $^2J_{C\text{-}F}$ = 32.6 Hz, C6), 136.4 (C4), 137.5 (brs, C1/C3). ^{13}C resonances from C2 and C8 partially overlap, but $^3J_{C\text{-}F}$ coupling in latter can be resolved. NH exchange seemingly slower in DMSO-d$_6$ than CD_3CN, leading to broad but resolved resonances for C1 and C3.

^{19}F NMR (377 MHz, DMSO-d$_6$) δ_F / ppm: -61.36 (s, CF_3)

HRMS (EI$^+$): [M]+ Found 280.04361; Required 280.04297 (0.64 ppm).

IR υ_{film} / cm^{-1}: 423, 484, 523, 602, 636, 672, 681, 699, 789, 816, 844, 855, 889, 906, 914, 948, 982, 999, 1043, 1105, 1116, 1142, 1161, 1181, 1275, 1312, 1325, 1338, 1381, 1398, 1535, 1580, 1620, 2941 (br), 3176 (br).

4-(4-(Dimethylamino)phenyl)-1H-pyrazole 4q$_H$

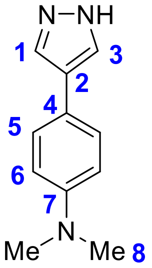

Prepared according to General Procedure **I** with 4-(dimethylamino)phenylboronic acid (0.396 g, 2.4 mmol, 2.0 equiv.), using a modified purification protocol. Following the evaporation of EtOAc, the crude product was suspended in Et_2O and acidified with ethereal HCl (2.0 M in Et_2O) to form a brown precipitate, which was isolated by vacuum filtration, washed liberally with further Et_2O, and then suspended in EtOAc. Aqueous NaOH (1.0 M) was added dropwise to the vigorously stirred suspension until the precipitate had fully dissolved, after which the organic layer was separated and the aqueous phase extracted with further EtOAc. The combined organic extracts were dried over anhydrous $MgSO_4$ and evaporated under reduced pressure to afford a brown solid, which was subsequently purified by recrystallisation from THF/hexane (0.068 g, 0.36 mmol, 30 %, m.p. 247 – 250 °C, [M] = $C_{11}H_{13}N_3$).

^{1}H NMR (400 MHz, CD_3CN) δ_H / ppm: 2.95 (6H, s, [C8]H_3), 6.80 (2H, m, [C6]*H*), 7.43 (2H, m, [C5]*H*), 7.79 (2H, s, [C1]*H* + [C3]*H*), 11.00 (1H, brs, NH). NH exchange leads to single time-averaged resonance for [C1]*H* and [C3]*H*.

^{13}C{^{1}H} NMR (101 MHz, CD_3CN) δ_C / ppm: 39.9 (C8), 113.0 (C6), 121.2 (C4), 122.2 (C2), 126.2 (C5), 149.6 (C7). Resonances for C1 and C3 not observed in CD_3CN as a result of substantial line broadening arising from NH exchange.

HRMS (ESI$^+$): [MH]+ Found 188.11990; Required 188.11822 (1.68 ppm).

IR υ_{film} / cm^{-1}: 410, 424, 489, 528, 586, 621, 660, 721, 755, 808, 861, 942, 953, 1005, 1031, 1060, 1122, 1150, 1205, 1223, 1276, 1349, 1443, 1477, 1500, 1518, 1573, 1614, 2918 (br), 3111 (br).

4-(3,4,5-Trifluorophenyl)-1H-pyrazole 4j$_H$

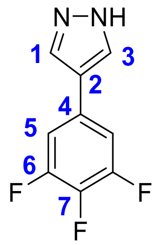

Prepared according to General Procedure I with 3,4,5-trifluorophenylboronic acid (0.422 g, 2.4 mmol, 2.0 equiv.). Product obtained as a white solid following recrystallisation from THF/hexane and further purification by flash column chromatography (silica, EtOAc, R_f = 0.54; 0.040 g, 0.20 mmol, 17 %, m.p. 257 – 261 °C, [M] = $C_9H_5F_3N_2$). NMR spectra acquired in both MeCN-*d*$_3$ and DMSO-*d*$_6$ for comparison.

^{1}H NMR (400 MHz, CD_3CN) δ_H / ppm: 7.35 (2H, m, [C5]*H*), 7.92 (2H, s, [C1]*H* + [C3]*H*), 11.24 (1H, brs, N*H*). NH exchange leads to single time-averaged resonance for [C1]H and [C3]H.

^{1}H NMR (400 MHz, DMSO-d$_6$) δ_H / ppm: 7.62 (2H, m, [C5]*H*), 8.02 (1H, s, [C1]*H*/[C3]*H*), 8.31 (1H, s, [C1]*H*/[C3]*H*), 13.06 (1H, brs, N*H*). NH exchange seemingly slower in DMSO-*d*$_6$ than CD_3CN, leading to broad but resolved resonances for [C1]*H* and [C3]*H*.

^{13}C{^{1}H} NMR (101 MHz, CD_3CN) δ_C / ppm: 109.5 (m, C5), 119.4 (m, C2), 129.9 (td, $^4J_{C-F}$ = 4.5 Hz, $^3J_{C-F}$ = 8.9 Hz, C4), 137.9 (dt, $^2J_{C-F}$ = 15.6 Hz, $^1J_{C-F}$ = 246.8 Hz, C7), 151.4 (ddd, $^3J_{C-F}$ = 4.4 Hz, $^2J_{C-F}$ = 10.0 Hz, $^1J_{C-F}$ = 245.9 Hz, C6). Resonances for C1 and C3 not observed in CD_3CN as a result of substantial line broadening arising from NH exchange.

^{13}C{^{1}H} NMR (101 MHz, DMSO-d$_6$) δ_C / ppm: 109.7 (m, C5), 119.3 (m, C2), 127.0 (brs, C1/C3), 130.7 (td, $^4J_{C-F}$ = 4.6 Hz, $^3J_{C-F}$ = 9.3 Hz, C4), 137.2 (brs, C1/C3), 137.2 (dt, $^2J_{C-F}$ = 15.6 Hz, $^1J_{C-F}$ = 246.8 Hz, C7), 151.2 (ddd, $^3J_{C-F}$ = 4.4 Hz, $^2J_{C-F}$ = 9.7 Hz, $^1J_{C-F}$ = 245.3 Hz, C6). NH exchange seemingly slower in DMSO-d$_6$ than CD_3CN, leading to broad but resolved resonances for C1 and C3.

^{19}F NMR (377 MHz, CD_3CN) δ_F / ppm: -137.2 (2F, m, [C6]F), -166.8 (1F, tt, $^4J_{H-F}$ = 6.6 Hz, $^3J_{F-F}$ = 20.0 Hz, [C7]F).

^{19}F NMR (377 MHz, DMSO-d$_6$) δ_F / ppm: -135.8 (2F, m, [C6]F), -166.0 (1F, tt, $^4J_{H-F}$ = 6.8 Hz, $^3J_{F-F}$ = 21.8 Hz, [C7]F).

HRMS (ESI$^+$): [MH]+ Found 199.04900; Required 199.04776 (1.24 ppm).

IR υ_{film} / cm^{-1}: 508, 527, 600, 629, 670, 695, 738, 816, 848, 882, 947, 999, 1033, 1044, 1162, 1241, 1276, 1290, 1332, 1377, 1393, 1449, 1465, 1517, 1543, 1585, 1613, 1631, 2901 (br), 3176 (br).

4-(1-Napthylenyl)-1H-pyrazole 4o$_H$

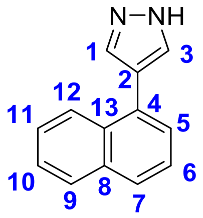

Prepared according to General Procedure I with 1-napthylenylboronic acid (0.412 g, 2.4 mmol, 2.0 equiv.). Product obtained as a white solid following recrystallisation from THF/hexane and further purification by flash column chromatography (silica, Et_2O, R_f = 0.45; 0.088 g, 0.45 mmol, 38 %, m.p. 98 – 101 °C, [M] = $C_{13}H_{10}N_2$).

^{1}H NMR (400 MHz, DMSO-d$_6$) δ_H / ppm: 7.49 – 7.57 (4H, m, [C5]*H* + [C6]*H* + [C10]*H* + [C11]*H*), 7.81 (1H, brs, [C1]*H*/[C3]*H*), 7.86 (1H, m, [C7]*H*), 7.97 (1H, m, [C9]*H*), 8.10 (1H, brs, [C1]*H*/[C3]*H*), 8.15 (1H, m, [C12]*H*), 13.13 (1H, brs, N*H*). NH exchange seemingly slower in DMSO-d$_6$ than CD_3CN, leading to broad but resolved resonances for [C1]*H* and [C3]*H*.

^{13}C{^{1}H} NMR (101 MHz, DMSO-d$_6$) δ_C / ppm: 119.6 (C2), 125.7 (C12), 126.1 (C5), 126.3 (C10), 126.7 (C11), 127.0 (C6), 127.3 (C7), 128.3 (brs, C1/C3), 128.8 (C9), 131.4 (C8+C13), 134.0 (C4), 139.2 (brs, C1/C3). NH exchange seemingly slower in DMSO-d$_6$ than CD_3CN, leading to broad but resolved resonances for C1 and C3.

HRMS (ESI$^+$): [MH]+ Found 195.09290; Required 195.09168 (1.22 ppm).

IR υ_{film} / cm^{-1}: 429, 448, 564, 629, 655, 676, 775, 799, 850, 932, 1011, 1022, 1151, 1350, 1506, 1594, 2899 (br), 3093 (br).

4-(2-Napthylenyl)-1H-pyrazole 4n$_H$

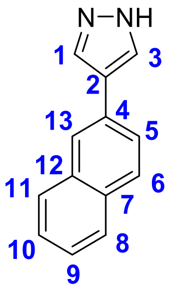

Prepared according to General Procedure I with 2-napthylenylboronic acid (0.396 g, 2.4 mmol, 2.0 equiv.), using a modified purification protocol. Following the evaporation of EtOAc, the crude product was suspended in Et_2O and acidified with ethereal HCl (2.0 M in Et_2O) to form a white precipitate, which was isolated by vacuum filtration, washed liberally with further Et_2O, and then suspended in EtOAc. Aqueous NaOH (1.0 M) was added dropwise to the vigorously stirred suspension until the precipitate had fully dissolved, after which the organic layer was separated and the aqueous phase extracted with further EtOAc. The combined organic extracts were dried over anhydrous $MgSO_4$ and evaporated under reduced pressure to afford a white solid, which was subsequently purified by recrystallisation from THF/hexane (0.111 g, 0.57 mmol, 48 %, m.p. 260 – 265 °C, [M] = $C_{11}H_{13}N_3$).

^{1}H NMR (400 MHz, DMSO-d$_6$) δ_H / ppm: 7.44 (1H, ddd, $^4J_{H9\text{-}H11}$ = 1.4 Hz, $^3J_{H9\text{-}H10}$ = 6.9 Hz, $^3J_{H8\text{-}H9}$ = 7.9 Hz, [C9]*H*), 7.50 (1H, ddd, $^4J_{H8\text{-}H10}$ = 1.4 Hz, $^3J_{H9\text{-}H10}$ = 6.9 Hz, $^3J_{H10\text{-}H11}$ = 8.3 Hz, [C10]*H*), 7.81 (1H, m, [C5]*H*), 7.88 (3H, m, [C6]*H* + [C8]*H* + [C11]*H*), 8.08 (1H, brs, [C1]*H*/[C3]*H*), 8.13 (1H, s, [C13]*H*), 8.31 (1H, brs, [C1]*H*/[C3]*H*), 13.00 (1H, brs, N*H*). NH exchange seemingly slower in DMSO-d$_6$ than CD_3CN, leading to broad but resolved resonances for [C1]*H* and [C3]*H*.

^{13}C{^{1}H} NMR (101 MHz, DMSO-d$_6$) δ_C / ppm: 121.6 (C2), 122.9 (C13), 125.0 (C5), 125.6 (C9), 126.4 (brs, C1/C3), 126.7 (C10), 127.9 (C11), 128.0 (C8), 128.7 (C6), 131.0 (C4), 132.0 (C7), 134.0 (C12), 137.0 (brs, C1/C3). NH exchange seemingly slower in DMSO-d$_6$ than CD_3CN, leading to broad but resolved resonances for C1 and C3.

HRMS (ESI$^+$): [MH]+ Found 195.09270; Required 195.09168 (1.02 ppm).

IR υ_{film} / cm^{-1}: 471, 483, 620, 667, 742, 811, 850, 888, 934, 961, 1032, 1132, 1357, 1599, 2917 (br), 3051, 3130 (br).

S2.3. Isotopically labelled substrates

***p*-Fluorophenyl acetate 1**

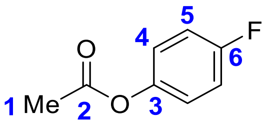

p-Fluorophenol **6$_H$** (7.3 g, 64.9 mmol, 1.0 equiv.) and triethylamine (NEt_3; 9.9 mL, 71.4 mmol, 1.1 equiv.) were dissolved in anhydrous CH_2Cl_2 (200 mL), and the resulting solution was cooled to 0 °C under a nitrogen atmosphere. Acetyl chloride (AcCl; 6.1 mL, 84.4 mmol, 1.2 equiv.) was added gradually over 5 min to the cooled solution; after addition was complete, the solution was stirred for 30 min at 0 °C and for a further 1 h at ambient temperature. The reaction was quenched with the addition of aqueous HCl (2.0 M, 30 mL), and the aqueous phase then extracted with CH_2Cl_2 (3 x 50 mL). The combined organic extracts were washed with saturated aqueous $NaHCO_3$ (2 x 50 mL) and saturated aqueous NaCl (50 mL), and then dried over anhydrous $MgSO_4$. The solvent was removed under reduced pressure to afford a pale yellow oil, which was subsequently purified by flash column chromatography (silica, 1:2 Et_2O:Petrol, R_f = 0.62) and distillation under reduced pressure (5 mbar, 75 °C) to afford a colourless liquid (8.2 g, 53.2 mmol, 82 %). The ^{1}H, ^{13}C and ^{19}F NMR spectra are consistent with the literature.[S1]

^{1}H NMR (400 MHz, CD_3CN) δ_H / ppm: 2.24 (3H, s, [C1]H_3), 7.07 – 7.18 (4H, m, [C4]*H* + [C5]*H*).

^{13}C{^{1}H} NMR (101 MHz, CD_3CN) δ_C / ppm: 21.2 (C1), 116.9 (d, $^2J_{C\text{-}F}$ = 23.6 Hz, C5), 124.5 (d, $^3J_{C\text{-}F}$ = 8.6 Hz, C4), 148.0 (d, $^4J_{C\text{-}F}$ = 2.8 Hz, C3), 161.1 (d, $^1J_{C\text{-}F}$ = 242 Hz, C6), 170.6 (C2).

^{19}F NMR (377 MHz, CD_3CN) δ_F / ppm: -119.1 (tt, $^4J_{H\text{-}F}$ = 4.9 Hz, $^3J_{H\text{-}F}$ = 8.2 Hz).

***p*-Fluorophenyl [^{13}CO]-acetate [^{13}CO]-1**

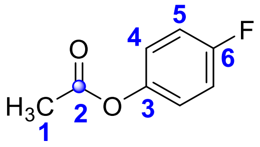

Prepared analogously to unlabelled *p*-fluorophenyl acetate **1**, from [^{13}CO]-acetyl chloride (0.36 mL, 5.0 mmol, 1.0 equiv.), 4-fluorophenol **6$_H$** (0.620 g, 5.5 mmol, 1.1 equiv.) and NEt_3 (0.84 mL, 6.0 mmol, 1.2 equiv.) in CH_2Cl_2 (25 mL). Isolated as a colourless oil following purification by flash column chromatography on silica, as described above (0.410 g, 2.6 mmol, 53 %, > 99.5 % ^{13}C). The ^{1}H, ^{13}C and ^{19}F NMR spectra are consistent with the unlabelled (^{12}C) isotopologue.[S1]

^{1}H NMR (400 MHz, CD_3CN) δ_H / ppm: 2.25 (3H, d, $^{2}J_{C2-H}$ = 7.1 Hz, [C1]H_3), 7.07 – 7.18 (4H, m, [C4]H + [C5]H).

^{13}C{^{1}H} NMR (101 MHz, CD_3CN) δ_C / ppm: 21.2 (d, $^{1}J_{C1-C2}$ = 60.5 Hz, C1), 116.9 (d, $^{2}J_{C-F}$ = 23.6 Hz, C5), 124.5 (dd, $^{3}J_{C2-C4}$ = 1.5 Hz, $^{3}J_{C-F}$ = 8.6 Hz, C4), 148.0 (dd, $^{4}J_{C-F}$ = 2.8 Hz, $^{2}J_{C2-C3}$ = 3.6 Hz, C3), 161.1 (d, $^{1}J_{C-F}$ = 242 Hz, C6), 170.6 (^{13}C2).

^{19}F NMR (377 MHz, CD_3CN) δ_F / ppm: -119.1 (ttd, $^{6}J_{C-F}$ = 0.9 Hz, $^{4}J_{H-F}$ = 4.9 Hz, $^{3}J_{H-F}$ = 8.2 Hz).

***p*-Fluorophenyl [$^{13}CH_3$]-acetate [$^{13}CH_3$]-1**

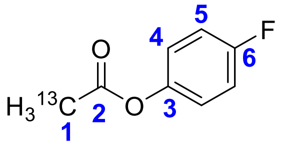

p-Fluorophenyl [$^{13}CH_3$]-acetate was prepared according to a different procedure from the unlabelled isotopologue, starting from [$^{13}CH_3$]-acetic acid ([$^{13}CH_3$]-AcOH) instead of the corresponding acetyl chloride. Under an inert atmosphere of N_2, anhydrous THF (16 mL), NEt_3 (1.1 mL, 7.8 mmol, 2.5 equiv.) and [$^{13}CH_3$]-AcOH (0.19 mL, 3.3 mmol, 1.05 equiv.) were dispensed to an oven-dried, two-necked flask, and the resulting solution was cooled to 0 °C. Pivaloyl chloride (PivCl; 0.38 mL, 3.1 mmol, 1.0 equiv.) was then added dropwise over a period of 1 min, leading to the evolution of a white precipitate. The suspension was stirred rapidly for 10 min at 0 °C, removed from the ice bath, and stirred for a further 15 min, after which time a solution of 4-fluorophenol (0.39 g, 3.4 mmol, 1.1 equiv.) and catalytic 4-(dimethylamino)pyridine (DMAP; 0.076 g, 0.62 mmol, 20 mo%) in anhydrous THF (5 mL) was added. The fully assembled reaction was subsequently stirred at room temperature, under N_2, for 18 h, then quenched with aqueous HCl (5 mL, 2.0 M). The solvent was removed under reduced pressure, and the crude redissolved in CH_2Cl_2. The organic phase was separated, and the aqueous phase extracted with further CH_2Cl_2 (3 x 10 mL). The combined organic extracts were washed with saturated $NaHCO_{3(aq)}$ and saturated $NaCl_{(aq)}$, dried over anhydrous $MgSO_4$, and evaporated under reduced pressure to afford a yellow liquid, which was purified by flash column chromatography as described above to afford a colourless liquid (0.481 g, 3.1 mmol, 70 %, > 98.5 % ^{13}C). The ^{1}H, ^{13}C and ^{19}F NMR spectra are consistent with the unlabelled (^{12}C) isotopologue.[S1]

^{1}H NMR (400 MHz, CD_3CN) δ_H / ppm: 2.25 (3H, d, $^{1}J_{C1\text{-}H}$ = 130.1 Hz, [$^{13}C1$]H_3), 7.07 – 7.18 (4H, m, [C4]H + [C5]H)

$^{13}C\{^{1}H\}$ NMR (101 MHz, CD_3CN) δ_C / ppm: 21.2 ($^{13}C1$), 116.9 (d, $^{2}J_{C\text{-}F}$ = 23.6 Hz, C5), 124.5 (d, $^{3}J_{C\text{-}F}$ = 8.6 Hz, C4), 148.0 (d, $^{4}J_{C\text{-}F}$ = 2.8 Hz, C3), 161.1 (d, $^{1}J_{C\text{-}F}$ = 242 Hz, C6), 170.6 (d, $^{1}J_{C1\text{-}C2}$ = 60.5 Hz, C2)

^{19}F NMR (377 MHz, CD_3CN) δ_F / ppm: -119.1 (tt, $^{4}J_{H\text{-}F}$ = 4.9 Hz, $^{3}J_{H\text{-}F}$ = 8.2 Hz)

***p*-Fluorophenyl acetate-*d*$_3$ [CD$_3$]-1**

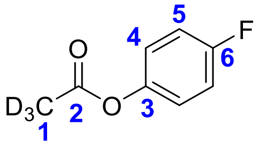

Prepared analogously to unlabelled 4-fluorophenyl acetate **1**, from acetyl chloride-*d*$_3$ (AcCl-*d*$_3$; 0.44 mL, 6.1 mmol, 1.1 equiv.), 4-fluorophenol **6**$_{\mathbf{H}}$ (0.653 g, 5.3 mmol, 1.0 equiv.) and NEt_3 (0.94 mL, 6.7 mmol, 1.2 equiv.) in CH_2Cl_2 (25 mL). Reaction quenched with aqueous 35% DCl (0.30 mL) and D_2O (5 mL) instead of the corresponding protiated species. Isolated as a colourless oil following purification by flash column chromatography on silica, as described above (0.473 g, 3.0 mmol, 49 %; 86.3 % [CD_3], 13.5 % [CD_2H], 0.2 % [CDH_2]). The ^{1}H, ^{13}C and ^{19}F NMR spectra are consistent with the protiated (CH_3) isotopologue.[S1]

^{1}H NMR (400 MHz, CD$_3$CN) δ_H / ppm: 7.07 – 7.18 (4H, m, [C4]*H* + [C5]*H*).

^{2}H NMR (61 MHz, CD$_3$CN) δ_D / ppm: 2.20 (s).

^{13}C{^{1}H} NMR (101 MHz, CD$_3$CN) δ_C / ppm: 20.5 (m, C1), 116.9 (d, $^2J_{C\text{-}F}$ = 23.6 Hz, C5), 124.5 (d, $^3J_{C\text{-}F}$ = 8.6 Hz, C4), 148.0 (d, $^4J_{C\text{-}F}$ = 2.8 Hz, C3), 161.1 (d, $^1J_{C\text{-}F}$ = 242 Hz, C6), 170.6 (m, C2).

^{19}F NMR (377 MHz, CD$_3$CN) δ_F / ppm: -119.1 (tt, $^4J_{H\text{-}F}$ = 4.9 Hz, $^3J_{H\text{-}F}$ = 8.2 Hz).

Triisopropyl(3-bromo-4-fluorophenoxy)silane 9

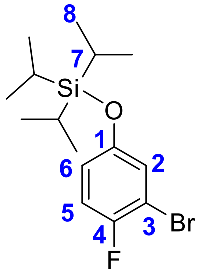

3-Bromo-4-fluorophenol (6.30 g, 33 mmol, 1.0 equiv.) and imidazole (5.61 g, 82 mmol, 2.5 equiv.) were dissolved in anhydrous CH_2Cl_2 (110 mL) to afford a yellow solution, to which triisopropylsilyl chloride (TIPSCl; 8.5 mL, 40.0 mmol, 1.2 equiv.) was added gradually over a period of 5 min. The combined solution was subsequently stirred for 16 h at ambient temperature under a protective atmosphere of N_2. After this time the reaction was quenched with saturated $NH_4Cl_{(aq)}$ and the aqueous phase extracted with CH_2Cl_2 (3 x 30 mL). The combined organic extracts were washed with saturated $NaCl_{(aq)}$, dried over anhydrous $MgSO_4$ and evaporated under reduced pressure to afford the TIPS-protected phenol as a pale yellow oil, which was subsequently purified by elution through a silica plug (9:1 petrol:Et_2O; 10.3 g, 90 %, 1.21 g mL^{-1}).

^{1}H NMR (400 MHz, $CDCl_3$) δ_H / ppm: 1.12 (18H, d, $^3J_{H7\text{-}H8}$ = 7.3 Hz, [C8]H_3), 1.27 (3H, m, [C7]*H*), 6.78 (1H, ddd, $^4J_{H\text{-}H}$ = 2.9 Hz, $^4J_{H6\text{-}F}$ = 3.9 Hz, $^3J_{H5\text{-}H6}$ = 8.9 Hz, [C6]*H*), 6.98 (1H, dd, $^3J_{H\text{-}F}$ = 8.2 Hz, $^3J_{H5\text{-}H6}$ = 8.9 Hz, [C5]*H*), 7.08 (1H, dd, $^4J_{H\text{-}H}$ = 2.9 Hz, $^4J_{H2\text{-}F}$ = 5.7 Hz, [C2]*H*).

^{13}C{^{1}H} NMR (101 MHz, $CDCl_3$) δ_C / ppm: 12.5 (C7), 17.8 (C8), 108.6 (d, $^2J_{C3\text{-}F}$ = 22.4 Hz, C3), 116.3 (d, $^2J_{C5\text{-}F}$ = 23.6 Hz, C5), 119.7 (d, $^3J_{C6\text{-}F}$ = 6.7 Hz, C6), 124.2 (C2), 152.5 (d, $^4J_{C\text{-}F}$ = 2.7 Hz, C1), 153.9 (d, $^1J_{C\text{-}F}$ = 242.2 Hz, C4).

^{19}F NMR (377 MHz, $CDCl_3$) δ_F / ppm: -118.3 (ddd, $^4J_{H6\text{-}F}$ = 3.9 Hz, $^4J_{H2\text{-}F}$ = 5.7 Hz, $^3J_{H\text{-}F}$ = 8.2 Hz)

^{29}Si NMR (80 MHz, $CDCl_3$) δ_{Si} / ppm: 17.0 (s)

HRMS (EI$^+$): [M]+ Found 346.07736; Required 346.07583 (1.52 ppm).

3-Deutero-4-fluorophenol [Ar-d_1]-6_H

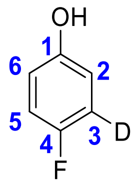

Under an inert atmosphere of N_2, triisopropyl(3-bromo-4-fluorophenoxy)silane **9** (9.0 g, 26 mmol, 1.0 equiv.) was dissolved in anhydrous Et_2O (90 mL), and the resulting solution cooled to –78 °C. With stirring, ^nBuLi (13 mL, 33 mmol, 1.3 equiv.; 2.5 M in hexanes) was added gradually over a period of 5 min, leading to the instantaneous formation of a yellow solution. Stirring was continued for a further 10 min at –78 °C, after which time the reaction was quenched rapidly with MeOD (2.6 mL, 65 mmol, 2.5 equiv.). The quenched solution was stirred for 10 min at –78 °C, and then warmed to room temperature over a period of 30 min. Tetra-n-butylammonium fluoride (TBAF; 34 mmol, 1.3 equiv.; 1.0 M in THF) was then added to the warmed solution, which was subsequently stirred for another 2 h at ambient temperature and then quenched with aqueous HCl (2.0 M). The organic phase was isolated and the aqueous phase extracted with both EtOAc (2 x 20 mL) and CH_2Cl_2 (2 x 20 mL); the combined organic extracts were then washed with saturated $NaCl_{(aq)}$, dried over anhydrous $MgSO_4$, and evaporated under reduced pressure to afford a crude oil. The crude was purified by flash column chromatography (silica, 7:1 petrol:EtOAc) and Kugelrohr distillation to afford a white crystalline solid (1.86 g, 63 %, m.p. 41 - 43°C; 98 % [d_1], 2 % [d_0]). The ^1H, $^{13}C\{^1H\}$ and ^{19}F NMR spectra are consistent with the unlabelled (protiated, **6_H**) isotopologue.

^1H NMR (400 MHz, $CDCl_3$) δ_H / ppm: 4.65 (1H, s, O*H*), 6.74 – 6.80 (2H, m, [C2]*H* + [C6]*H*), 6.89 - 6.96 (1H, m, [C5]*H*).

^2H NMR (61 MHz, $CHCl_3$) δ_D / ppm: 6.91 (m).

$^{13}C\{^1H\}$ NMR (101 MHz, $CDCl_3$) δ_C / ppm: 115.8 (m, $^2J_{C\text{-}F}$ = 23.2 Hz, $^1J_{C\text{-}D}$ = 25.0 Hz, C3) 116.0 (d, $^2J_{C\text{-}F}$ = 23.1 Hz, C5), 116.2 (*app* t, $^2J_{C\text{-}F}$ = 7.6 Hz, C2+C6), 151.5 (m, C1), 156.9 (d, $^1J_{C\text{-}F}$ = 238 Hz, C4). Apparent triplet (*app* t) at δ_C = 116.2 ppm arises from two overlapped doublets, with coincidental degeneracy of $^3J_{C\text{-}F}$ = $^2\Delta C(D)$.

^{19}F NMR (377 MHz, $CDCl_3$) δ_F / ppm: -124.47 (m, $^3J_{D\text{-}F}$ = 1.1 Hz, $^4J_{H\text{-}F}$ = 4.4 Hz, $^3J_{H\text{-}F}$ = 8.3 Hz).

HRMS (EI$^+$): [M]+ Found 113.03929; Required 113.03817 (1.12 ppm).

3-Deutero-4-fluorobenzonitrile [Ar-d_1]-10

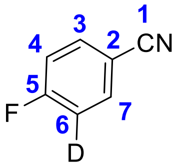

Under an inert atmosphere of N_2, anhydrous THF (125 mL) was dispensed to an oven-dried, three-necked flask fitted with a condenser, septum, and pressure-equalising addition funnel. The flask was cooled to -78°C ($CO_{2(s)}$/Me_2CO), and *n*-butyl lithium (^nBuLi, 1.6 M in THF; 17.2 mL, 27.5 mmol, 1.10 equiv.) was added, with stirring, over a period of 5 min. The resulting solution was stirred at -78°C for a further 10 min to ensure thermal equilibration. 3-Bromo-4-fluorobenzonitrile (5.0 g, 25.0 mmol, 1.00 equiv.) in anhydrous THF (25 mL, 1.0 M) was then added dropwise, *via* the funnel, to the cooled ^nBuLi solution (reverse addition) over a period of 20 min, affording an orange solution. Once addition of the benzonitrile was complete, the fully assembled reaction was stirred at -78°C for a further 15 min, and then *rapidly* quenched, in succession, with MeOD (5.1 mL, 125 mmol, 5.0 equiv.) and D_2O (2.5 mL, 125 mmol, 5.0 equiv.), with vigorous stirring. The quenched reaction mixture was stirred vigorously at -78°C for a further 10 min, after which time the flask was removed from the $CO_{2(s)}$/Me_2CO bath and stirred for a further 30 min. The reaction mixture was then exposed to air, quenched with HCl (2.0 M, 10 mL), and diluted with Et_2O; the organic phase separated; and the aqueous phase extracted with further Et_2O (3 x 20 mL). The combined organic extracts were washed with saturated NaCl(aq), dried over anhydrous $MgSO_4$, and evaporated under reduced pressure to afford the deuterated product as a pale yellow crystalline solid (needles). 2.98 g. 24.4 mmol. 98%. No further purification was required. >99 % D incorporation (**[Ar-d_1]-10**). The ^1H, $^{13}C\{^1H\}$ and ^{19}F NMR spectra are consistent with the unlabelled (protiated; **10**) isotopologue.

^1H NMR (400 MHz, $CDCl_3$) δ_H / ppm: 7.20 (1H, m, [C4]H), 7.71 (2H, m, [C3]H + [C7]H).

^2H NMR (61 MHz, $CHCl_3$) δ_D / ppm: 7.21 (m).

$^{13}C\{^1H\}$ NMR (101 MHz, $CDCl_3$) δ_C / ppm: 108.6 (m, $^3J_{D\text{-}C}$ = 1.3 Hz, $^4J_{C\text{-}F}$ = 3.7 Hz, C2), 116.6 (m, $^2J_{C6\text{-}F}$ = 22.7 Hz, $^1J_{C\text{-}D}$ = 25.5 Hz, C6), 116.9 (d, $^2J_{C4\text{-}F}$ = 22.7 Hz, C4), 118.0 (C1), 134.6 (*app* t, $^3J_{C\text{-}F}$ = 9.1 Hz, C3+C7), 165.0 (d, $^1J_{C\text{-}F}$ = 256.7 Hz, C5). Apparent triplet (*app* t) at δ_C = 134.6 ppm arises from two overlapped doublets, with coincidental degeneracy of $^3J_{C\text{-}F}$ = $^2\Delta C(D)$.

^{19}F NMR (377 MHz, $CDCl_3$) δ_F / ppm: -102.7 (m, $^3J_{D\text{-}F}$ = 1.1 Hz, $^4J_{H\text{-}F}$ = 5.1 Hz, $^3J_{H\text{-}F}$ = 8.2 Hz).

3-Deutero-4-fluorobenzylamine [Ar-*d*$_1$]-2

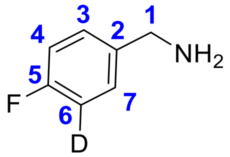

Under an inert atmosphere of N_2, lithium aluminium hydride ($LiAlH_4$, 1.0 M in THF; 16.4 mL, 16.4 mmol, 2.5 equiv.) was dispensed to an oven-dried, three-necked flask fitted with a condenser, septum, and pressure-equalising addition funnel. The $LiAlH_4$ solution was diluted with additional anhydrous THF (10 mL), and cooled to 0°C. 3-Deutero-4-fluorobenzonitrile **[Ar-*d*$_1$]-10** (0.80 g, 6.6 mmol, 1.00 equiv.) in anhydrous THF (10 mL, 0.66 M) was added dropwise to the cooled solution, *via* the funnel, over a period of 10 min, after which the fully assembled reaction was stirred for a further 30 min at 0°C, and then heated to reflux under N_2. After 16 h at reflux, the reaction solution was cooled to 0°C, diluted with THF (15 mL), stirred vigorously, and then quenched carefully under N_2 *via* the dropwise addition of aqueous NaOH (10% w/v, 5 mL), followed by H_2O (10 mL). The quenched reaction mixture was, in succession, warmed to room temperature, stirred vigorously for a further 30 min, diluted with Et_2O (10 mL) and saturated aqueous NaCl (5 mL), and decanted.The aqueous phase was extracted with further Et_2O (3 x 20 mL), and the combined organic extracts dried over anhydrous $MgSO_4$ and evaporated under reduced pressure to afford a pale yellow liquid, which was subsequently purified by Kugelrohr distillation (3 mbar, 50 °C - 80 °C) to afford 3-deutero-4-fluorobenzylamine **[Ar-*d*$_1$]-2** as a colourless liquid (0.402 g, 3.2 mmol, 49%). The ^{1}H, ^{13}C{^{1}H} and ^{19}F NMR spectra are consistent with the unlabelled (protiated; **2**) isotopologue.

^{1}H NMR (400 MHz, CD_3CN) δ_H / ppm: 1.49 (2H, brs, N*H*), 3.79 (2H, s, [C1]H_2), 7.07 (1H, m, [C4]*H*), 7.36 (2H, m, [C3]*H* + [C7]*H*).

^{2}H NMR (61 MHz, CD_3CN) δ_D / ppm: 7.08 (m).

^{13}C{^{1}H} NMR (101 MHz, CD_3CN) δ_C / ppm: 45.2 (C1), 114.5 (m, $^2J_{C\text{-}F}$ = 21.2 Hz, $^1J_{C\text{-}D}$ = 25.0 Hz, C6), 114.7 (d, $^2J_{C\text{-}F}$ = 21.2 Hz, C4), 128.7 (*app* t, $^3J_{C\text{-}F}$ = 9.0 Hz, C3+C7), 140.5 (m, C2), 161.4 (d, $^1J_{C\text{-}F}$ = 241.4 Hz, C5). Apparent triplet (*app* t) at δ_C = 128.7 ppm arises from two overlapped doublets, with coincidental degeneracy of $^3J_{C\text{-}F}$ = $^2\Delta$C(D).

^{19}F NMR (377 MHz, CD_3CN) δ_F / ppm: -119.2 (m, $^3J_{D\text{-}F}$ = 1.3 Hz, $^4J_{H\text{-}F}$ = 5.4 Hz, $^3J_{H\text{-}F}$ = 9.1 Hz).

[^{15}N]-4-fluorobenzamide [^{15}N]-11

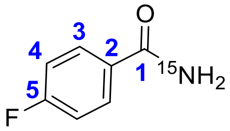

A round-bottomed flask was charged under air with $^{15}NH_4Cl$ (0.455 g, 8.3 mmol, 1.0 equiv.), deionised H_2O (4 mL) and Et_2O (4 mL). The resulting biphasic mixture was cooled to 0°C, stirred vigorously (1400 rpm) for 2 min, and 4-fluorobenzoyl chloride (2.0 mL, 16.9 mmol, 2.0 equiv.) was added. Aqueous NaOH (4 mL, 7.4 M, 29.6 mmol, 3.5 equiv.) was then added dropwise to the rapidly stirred mixture, still at 0°C, leading to the instantaneous evolution of a white precipitate. The resulting suspension was stirred vigorously at 0°C for a further 10 min, and then warmed to room temperature over a period of 15 min with continuous stirring. The precipitate was isolated by vacuum filtration, washed liberally with H_2O and then sparingly with chilled Et_2O (0°C), and dried *in vacuo* to afford [^{15}N]-4-fluorobenzamide as a white solid (0.881 g, 6.3 mmol, 76 %). The ^{1}H, ^{13}C{^{1}H} and ^{19}F NMR spectra are consistent with the unlabelled (**11**) isotopologue.

^{1}H NMR (400 MHz, CD_3CN) δ_H / ppm: 6.04 (1H, brd, $^1J_{H\text{-}N}$ = 88.5 Hz, N*H*), 6.77 (1H, brd, $^1J_{H\text{-}N}$ = 89.2 Hz, N*H*), 7.21 (2H, m, [C4]*H*), 7.90 (2H, m, [C3]*H*).

^{13}C{^{1}H} NMR (101 MHz, CD_3CN) δ_C / ppm: 115.2 (d, $^2J_{C\text{-}F}$ = 22.0 Hz, C4), 130.0 (d, $^3J_{C\text{-}F}$ = 9.1 Hz, C3), 130.4 (dd, $^4J_{C\text{-}F}$ = 3.0 Hz, $^2J_{C\text{-}N}$ = 8.6 Hz, C2), 164.7 (d, $^1J_{C\text{-}F}$ = 249.1 Hz, C5), 167.5 (d, $^1J_{C\text{-}N}$ = 16.1 Hz, C1).

^{19}F NMR (377 MHz, CD_3CN) δ_F / ppm: -110.7 (tt, $^4J_{H\text{-}F}$ = 5.5 Hz, $^3J_{H\text{-}F}$ = 8.9 Hz).

^{15}N{^{1}H} NMR (41 MHz, CD_3CN) δ_F / ppm: 96.8 (s)

[^{15}N]-4-fluorobenzylamine [^{15}N]-2

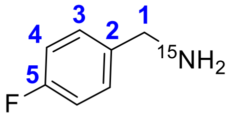

Under an inert atmosphere of N_2, lithium aluminium hydride ($LiAlH_4$, 1.0 M in THF; 16.0 mL, 16.0 mmol, 2.5 equiv.) was dispensed to an oven-dried, three-necked flask fitted with a condenser, septum, and pressure-equalising addition funnel. The $LiAlH_4$ solution was diluted with additional anhydrous THF (10 mL), and cooled to 0°C. [^{15}N]-4-fluorobenzamide **[^{15}N]-11** (0.88 g, 6.4 mmol, 1.00 equiv.) in anhydrous THF (10 mL, 0.64 M) was added dropwise to the cooled solution, *via* the funnel, over a period of 10 min, after which the fully assembled reaction was stirred for a further 30 min at 0°C, and then heated to reflux under N_2. After 16 h at reflux, the reaction solution was cooled to 0°C, diluted with THF (15 mL), stirred vigorously, and then quenched carefully under N_2 *via* the dropwise addition of aqueous NaOH (10% w/v, 5 mL), followed by H_2O (10 mL). The quenched reaction mixture was, in succession, warmed to room temperature, stirred vigorously for a further 30 min, diluted with Et_2O (10 mL) and saturated aqueous NaCl (5 mL), and decanted.The aqueous phase was extracted with further Et_2O (3 x 20 mL), and the combined organic extracts dried over anhydrous $MgSO_4$ and evaporated under reduced pressure to afford a pale yellow liquid, which was subsequently purified by Kugelrohr distillation (3 mbar, 50 °C - 80 °C) to afford the [^{15}N]-4-fluorobenzylamine **[^{15}N]-2** as a colourless liquid (0.495 g, 3.9 mmol, 61%). The ^{1}H, ^{13}C{^{1}H} and ^{19}F NMR spectra are consistent with the unlabelled (**2**) isotopologue.

^{1}H NMR (400 MHz, CD_3CN) δ_H / ppm: 1.46 (2H, brs, N*H*), 3.79 (2H, s, [C1]H_2), 7.07 (2H, m, [C4]*H*), 7.36 (2H, m, [C3]*H*).

^{13}C{^{1}H} NMR (101 MHz, CD_3CN) δ_C / ppm: 45.2 (d, $^{1}J_{C\text{-}N}$ = 4.1 Hz, C1), 114.7 (d, $^{2}J_{C\text{-}F}$ = 21.3 Hz, C4), 128.8 (d, $^{3}J_{C\text{-}F}$ = 8.0 Hz, C3), 140.5 (d, $^{2}J_{C\text{-}N}$ = 3.1 Hz, C2), 161.5 (d, $^{1}J_{C\text{-}F}$ = 241.3 Hz, C5).

^{19}F NMR (377 MHz, CD_3CN) δ_F / ppm: -119.0 (tt, $^{4}J_{H\text{-}F}$ = 5.5 Hz, $^{3}J_{H\text{-}F}$ = 9.2 Hz).

^{15}N{^{1}H} NMR (41 MHz, CD_3CN) δ_F / ppm: 23.6 (s).

S2.4. N-Acetylated azoles

1-Acetyl-1,2,4-triazole $4a_{Ac}$

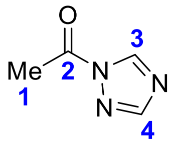

1,2,4-Triazole **$4a_H$** (5.0 g, 72.4 mmol, 1.0 equiv.) and triethylamine (NEt_3; 11 mL, 78.9 mmol, 1.1 equiv.) were dissolved in anhydrous THF (200 mL), and the resulting solution was cooled to 0 °C under a nitrogen atmosphere. Acetyl chloride (6.2 mL, 86.9 mmol, 1.2 equiv.) was added gradually over 5 min, affording a white precipitate; once addition was complete, the suspension was stirred vigorously for 15 min at 0 °C. The suspension was subsequently warmed to ambient temperature, diluted with Et_2O (50 mL) and stirred vigorously for a further 30 min. The white precipitate was then removed by vacuum filtration, and the solvent removed under reduced pressure (> 50 mbar, 40 °C) to afford a colourless oil, which crystallised spontaneously upon cooling to 4 °C. The crude product was subsequently sublimed *in vacuo* (1 mbar, 40 – 60 °C) to afford a white crystalline solid (6.51 g, 58.6 mmol, 81 %), which was swiftly transferred to a nitrogen-filled glovebox for storage and handling. The ^{1}H and ^{13}C and NMR spectra are consistent with the literature.[S2-S3]

^{1}H NMR (400 MHz, CD_3CN) δ_H / ppm: 2.68 (3H, s, [C1]H_3), 8.07 (1H, s, [C4]*H*), 8.95 (1H, s, [C3]*H*).

^{13}C{^{1}H} NMR (101 MHz, CD_3CN) δ_C / ppm: 22.7 (C1), 144.7 (C3), 154.0 (C4), 169.4 (C2).

1-(Acetyl-*d*$_3$)-1,2,4-triazole [CD_3]-$4a_{Ac}$

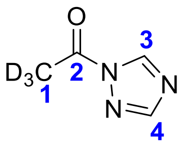

Prepared and purified analogously to the unlabelled isotopologue **$4a_{Ac}$**, from 1,2,4-triazole **$4a_H$** (0.77 g, 11.2 mmol, 1.0 equiv.), acetyl chloride-*d_3* (AcCl-*d_3;* 0.91 mL, 12.3 mmol,1.1 equiv.) and NEt_3 (1.62 mL, 11.6 mmol, 1.05 equiv.) and anhydrous THF (40 mL). Product isolated as a white crystalline solid following purification by sublimation (1.01 g, 8.9 mmol, 79 %; 97% [CD_3], 3 % [CD_2H]). The ^{1}H and ^{13}C and NMR spectra are consistent with the unlabelled isotopologue.[S2-S3]

^{1}H NMR (400 MHz, CD_3CN) δ_H / ppm: 8.07 (1H, s, [C4]*H*), 8.95 (1H, s, [C3]*H*).

^{2}H NMR (61 MHz, CD_3CN) δ_D / ppm: 2.61 (s).

^{13}C{^{1}H} NMR (101 MHz, CD_3CN) δ_C / ppm: 22.0 (m, $^1J_{D\text{-}C}$ = 19.9 Hz, C1), 144.7 (C3), 154.0 (C4), 169.4 (C2).

1-Acetyl-pyrazole 4b$_{Ac}$

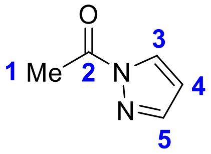

Pyrazole **4b$_{Ac}$** (3.0 g, 44.1 mmol, 1.0 equiv.) and triethylamine (NEt_3; 6.7 mL, 48.5 mmol, 1.10 equiv.) were dissolved in anhydrous THF (150 mL), and the resulting solution was cooled to 0 °C under a nitrogen atmosphere. Acetyl chloride (3.8 mL, 52.9 mmol, 1.2 equiv.) was added gradually over 5 min, affording a white precipitate; once addition was complete, the suspension was stirred vigorously for 15 min at 0 °C. The suspension was subsequently warmed to ambient temperature, and stirred vigorously for a further 30 min. The white precipitate was removed by filtration, and the solvent removed under reduced pressure (> 100 mbar, 40 °C) to afford a pale yellow oil. The crude product was subsequently distilled under reduced pressure (25 mbar, 65 °C), affording a pure, colourless oil (3.66 g, 33.2 mmol, 75 %), which was swiftly transferred to a nitrogen-filled glovebox for storage and handling. The ^{1}H and ^{13}C and NMR spectra are consistent with the literature.[S4]

^{1}H NMR (400 MHz, CD_3CN) δ_H / ppm: 2.66 (3H, s, [C1]H_3), 6.52 (1H, dd, $^{3}J_{H4\text{-}H5}$ = 1.5 Hz, $^{3}J_{H3\text{-}H4}$ = 2.9 Hz, [C4]*H*), 7.76 (1H, m, [C5]*H*), 8.28 (1H, dd, $^{4}J_{H3\text{-}H5}$ = 0.7 Hz, $^{3}J_{H3\text{-}H4}$ = 2.9 Hz, [C3]*H*).

^{13}C{^{1}H} NMR (101 MHz, CD_3CN) δ_C / ppm: 21.1 (C1), 109.7 (C4), 128.0 (C3), 143.9 (C5), 169.5 (C2).

S3. Reaction Monitoring Details

S3.1. General considerations

Instrumentation

Unless otherwise stated (e.g., for Eyring analysis), all reaction monitoring experiments were performed at 293.1 K (T_{NMR} = 20.0 °C) using a Bruker Ascend 400 MHz NMR spectrometer, fitted with a broadband direct-detect CryoProbe Prodigy unit and located in a thermostatted instrument room. The ambient temperature of the instrument room was controlled to within ±2 °C of the specified probe temperature (T_{IRM} = 20.0 °C ± 2.0 °C), and a default nitrogen gas flow rate of 450 L h^{-1} was set on the spectrometer to minimise the temperature gradient across the sample once in the probe.

Stock solutions

Solid compounds were weighed using a high precision analytical balance (resolution = 0.01 mg) and liquids were dispensed with gas-tight, glass microsyringes. All stock solutions were prepared in volumetric glassware, under ambient conditions, and typically used on the day of their preparation; all solutions stored for longer periods (< 3 days) were flushed with N_2, sealed, kept in the fridge (4 °C), and their stability/composition assessed by ^{1}H NMR analysis prior to use in monitoring experiments. Internal integration standards, irrespective of their physical state, were either weighed directly into volumetric flasks, or weighed into a vial and subsequently transferred to a volumetric flask with extensive washing.

For each series of kinetics experiments, a parent stock solution of an internal integration standard, of concentration $[IS]_0$, was freshly prepared in the required solvent, using either 1-fluoronaphthalene (1-F-Nap; >99 %, ^{1}H/^{19}F NMR, $[IS]_0$ = 0.0500 ± 0.0010 M) or 1,3,5-trimethoxybenzene (1,3,5-TMB; > 99 %, ^{1}H NMR, $[IS]_0$ = 0.0333 ± 0.00067 M). Moderately anhydrous solvents, dried for > 48 h over activated 3 Å molecular sieves, were used to prepare this stock solution, but no attempts were made to rigorously exclude air or moisture during solution preparation (no evidence was found to suggest modest concentrations of adventitious water were overly problematic). All other stock solutions were prepared from this parent solution, ensuring the concentration of the internal integration standard remained approximately constant irrespective of the ratios in which the various stock solutions were mixed during reaction assembly (stock solutions were typically < 0.30 M in any given substrate/reagent).

NMR processing

All NMR spectra were processed using standard MestReNova software (Version 11), with phasing (zeroth- and first-order) and baseline (Whittaker Smoother or low order Bernstein polynomial) corrections optimised manually. All spectra acquired in a kinetic run were processed identically as a stack, with exponential line broadening (typically, ^{1}H: 0.30 Hz; ^{19}F: 1.0 Hz) applied prior to phasing and baseline corrections unless precluded by spectral congestion/overlap. Pseudo-2D data (from SF-NMR)

was subjected to preliminary processing with TopSpin 3.6 (xf2; split2D), and then loaded into MestReNova as a stack of 1D spectra for further processing.

Solute concentrations $[x]_t$ were determined by normalisation against an inert integration standard of known concentration $[IS]_0$ (Eqn S1). $I_{t,x}^{(i)}$ and $I_{t,IS}^{(j)}$ denote the integrals of the i th and j th resonances of the solute (x) and internal standard (IS), respectively, and $N_x^{(i)}$, $N_{IS}^{(j)}$ denote the respective number of nuclei contributing to each resonance.

$$[x]_t = \left(\frac{I_{t,x}^{(i)}}{I_{t,IS}^{(j)}}\right).\left(\frac{N_{IS}^{(j)}}{N_x^{(i)}}\right).[\mathrm{IS}]_0 \qquad (S1)$$

S3.2. Manually assembled reactions

General concerns

All conventional (non-SF) kinetic studies were performed with *in situ* reaction monitoring by 1D ^{1}H or ^{19}F NMR spectroscopy (single excitation pulse), using anhydrous MeCN-d_3 (^{1}H) / MeCN-h_3 (^{1}H/^{19}F) or THF-d_8 (^{1}H) / THF-h_8 (^{1}H/^{19}F) as solvents, and 1-fluoronaphthalene (> 99 %, ^{1}H/^{19}F, c_{IS} = 0.0500 $\pm$ 0.0010 M) or 1,3,5-trimethoxybenzene 1,3,5-TMB (> 99 %, ^{1}H, c_{IS} = 0.0333 $\pm$ 0.00067 M) as internal integration standards. Reactions were initiated by manual assembly, under ambient conditions (T_{IRM} = 20.0 °C $\pm$ 2.0 °C), with samples subsequently loaded into the spectrometer *via* a Bruker SampleXpress sample changer. All reactions were assembled in Norell 5mm borosilicate NMR tubes, with sample rotation disabled for all kinetic monitoring experiments.

All reactions monitored *in situ* were analysed using a standardised set of NMR acquisition parameters, with the sample remaining in the probe throughout the course of the reaction. In each kinetic run, the composition of the reaction mixture was analysed periodically using the standard multi_zgvd program in TopSpin 3.6, with each time point characterised by a ^{1}H or ^{19}F spectrum acquired using: (i) a single transient (ns = 1); (ii) a 30° excitation pulse (zg30; ^{1}H/^{19}F); (iii) a relaxation delay of t_{D1} = 10.0 s (^{1}H/^{19}F); (iv) an acquisition time of t_{AQ} = 3.98 s (^{1}H) or 3.47 s (^{19}F); and (v) a pre-set delay of *at least* t_{MD} = 5.0 s between consecutive spectra (multi_zgvd delay), affording *minimum* total recycle times ($t_R = t_{AQ} + t_{D1} + t_{MD}$) of t_R = 19.0 s and t_R = 18.5 s for ^{1}H and ^{19}F NMR monitoring, respectively.

Typical reaction assembly

All conventional kinetics experiments (non-SF) were carried out according to a standard routine; a typical reaction assembly is outlined here (Scheme S1).

In a standard kinetics experiment, all mutually unreactive components – typically some combination of an acyl acceptor (e.g., **2**), base (e.g., **3**), and/or *N*-heterocycle catalyst (e.g., **4a$_H$**) – were first assembled together in the NMR tube to afford a stable sample with a volume of > 600 uL (Solution 1); this sample was typically prepared < 2 h prior to reaction initiation. This pre-reactive sample (Solution 1) was loaded

into the NMR probe, automatically tuned to ^{1}H and matched, locked to the deuterium (^{2}H) signal of the solvent if applicable (MeCN-d_3/THF-d_8), and subjected to gradient shimming (^{2}H for MeCN-d_3/THF-d_8; lockoff ^{1}H for MeCN-h_3/THF-h_8). For samples prepared using deuterated solvents (MeCN-d_3/THF-d_8), the ^{2}H lock level was then further optimised by refining the Z-X-Y-XZ-YZ-Z shims, using the automated tune routine in TopShim. The receiver gain was optimised automatically, and a ^{1}H spectrum acquired using standard acquisition parameters (*vide supra*); for monitoring by ^{19}F NMR, the probe was then tuned and matched to ^{19}F, the receiver gain optimised, and a standard ^{19}F NMR spectrum acquired.

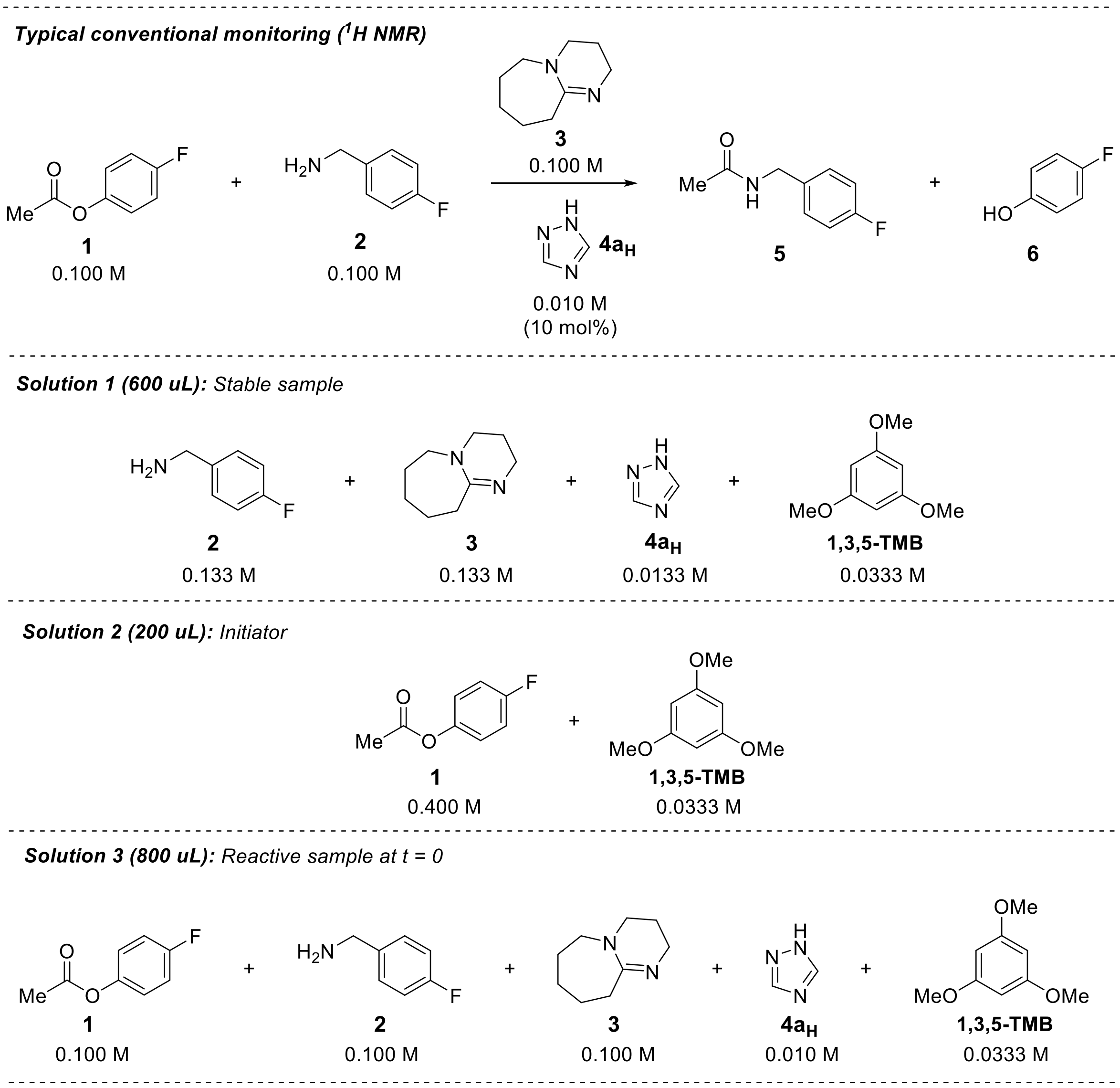

Scheme S1: Typical reaction assembly for *in situ* NMR monitoring of manually assembled reactions, in this case for monitoring the **4a**$_{\mathbf{H}}$-catalyzed aminolysis of **1** by **2** in the presence of **3** by ^{1}H NMR spectroscopy. Concentrations shown correspond to standard catalytic conditions (equimolar 0.10 M **1**, **2**, base; 10 mol% azole). Reactions monitored in either MeCN-d_3/THF-d_8 (^{1}H NMR), or MeCN-h_3/THF-h_8 (^{19}F NMR). 1,3,5-Trimethoxybenzene 1,3,5-TMB (0.0333 M) was typically used as an internal standard for ^{1}H NMR monitoring experiments; 1-F-naphthalene 1-F-Nap (0.0500 M) was used for all ^{19}F NMR experiments (and occasionally ^{1}H NMR experiments).

The pre-reactive sample (Solution 1) was subsequently ejected from the spectrometer and transferred to a proximate fumehood (T_{IRM} = 20.0 °C ± 2.0 °C), whereupon it was uncapped and the reaction initiated by injection of the appropriate reagent/substrate *via* a glass microsyringe (typically an electrophilic acyl donor, e.g. **1**; Solution 2). The fully assembled reaction (Solution 3) was rapidly mixed by inverting the capped sample (three times) and shaking vigorously (for > 5 s), and then swiftly transferred back to the spectrometer, loaded into the probe, locked (^{1}H monitoring only) and subjected to gradient shimming (^{1}H monitoring only), after which monitoring was commenced immediately. The total time elapsed between reaction initiation and acquisition of the first spectrum – the dead time, t_{Dead} – was recorded manually; the time point t_i corresponding to the ith spectrum (i = 0 for Solution 1; i = 1,2,3.. for Solution 3) was calculated according to: $t_i = t_{Dead} + (i-1).t_R$.

For regime **II**, in which variable temperature kinetic data was obtained by conventional in situ ^{19}F NMR spectroscopy, the pre-reactive sample was heated to the required temperature in the NMR probe whilst the initiating solution was subject to external thermal equilibration in an oil bath; the full reaction was assembled immediately (< 20 s) after ejection of the pre-reactive sample from the spectrometer, and returned similarly quickly. This approach was deemed sufficiently robust for **4a$_H$**-catalysed aminolysis in THF (regime **II**) only, as the initial rate of product evolution in MeCN (regime **I**) was exceptionally rapid. VR-SF-^{1}H NMR was used for **4b$_H$**-catalysed aminolysis (regime **III**) in order to capture the pre-steady state accumulation of **4b$_{Ac}$**. For variable-temperature studies of aminolysis in THF (regime **II**), a reduced catalyst loading ([**4a$_H$**]$_0$ = 0.005 M, 5 mol%) was used in all runs and graphical normalization of the resulting kinetics was conducted using low conversion data (F_1 < 20 %) only, to enable the approximations [**4a$_{\{DBUH\}}$**] ≈ [**4a$_H$**]$_0$ and $v \approx k^{(II)}$[**1**][**4a$_H$**]$_0$.

S3.3. Variable Ratio Stopped-flow NMR (VR-SF-NMR) monitoring

General concerns

Kinetic studies using variable ratio stopped-flow NMR (VR-SF-NMR) were typically performed, unless otherwise stated, at 293.1 K (20.0 °C), using ^{1}H or ^{19}F NMR spectroscopy, anhydrous MeCN *or* THF as solvents, and 1-fluoronaphthalene (>99 %, ^{1}H/^{19}F) or 1,3,5-trimethoxybenzene 1,3,5-TMB (> 99%,^{1}H) as internal integration standards. All reactions were assembled using a custom-built variable-ratio VR-SF NMR system, with the temperature of the system regulated collectively by both the NMR spectrometer (N_2) and an external recirculating heater-chiller. Prior to the loading of any stock solutions, the entire SF-NMR system (*vide infra*) was flushed with copious anhydrous solvent (MeCN or THF as required) and left to stand for > 1 h. Throughout operation, all three syringes (A, B, C) of the variable ratio syringe drive (*vide infra*) were loaded with solutions, even if only two were required for reaction assembly; in such a case the third was loaded with the appropriate solvent, rather than being left empty.

The VR-SF-NMR unit used to assemble all reactions reported in this work was reserved exclusively for anhydrous reactions using aprotic solvents only (e.g. MeCN, THF, DCM); to avoid inadvertent exposure to excessive moisture, all reactions in aqueous solution or other protic media (e.g., MeOH) were assembled using a second, identical, unit.

All variable temperature reaction monitoring (10°C – 50°C) was performed by SF-NMR to ensure: (i) close thermal regulation of both the active reaction volume and the pre-magnetised but otherwise unmixed reactants; and (ii) access to a large dynamic range of reaction rates, especially at higher temperatures. For all kinetic runs the temperature of the NMR probe (T_{NMR}) and external chiller (T_{Ch}) were set identically, and production-quality runs were only initiated once *both* temperatures had equilibrated to within $\pm$ 0.2 °C of the target temperature. Kinetics experiments conducted at multiple different temperatures – e.g., for Eyring analysis – were performed in order of increasing temperature, with the probe reshimmed on a typical reaction mixture following each change in temperature; to save time, gradient shimming (^{1}H) was performed prior to full thermal equilibration, when temperature of the chiller (T_{Ch}) had attained a temperature within $\pm$ 1.0 °C of the target.

Instrumentation details

Full details of VR-SF-NMR instrumentation have been reported previously.[S5] In brief, it consists of the following key elements: (i) a *triple syringe-drive unit*, consisting of three independent glass syringes (A, B, C; 2500 µL each) driven by stepper motors, each connected to a separate *reagent line*; (ii) a *thermostatted umbilical*, encapsulating the outlet reagent lines from all three main syringes, that can be inserted directly into the NMR spectrometer; (iii) a *mixing cell*, also enclosed within the umbilical and located in the *spinner adaptor*, at which all three reagent lines converge; (iv) a *glass flow cell* (300 uL, 3mm e.d.), held in the NMR probe head, which captures the output solution from the mixing cell and houses the active volume for the NMR measurement; and (v) a *waste line*, leading from the flow cell to an external waste bottle. The mixing cell is additionally fed by a fourth inlet line (D), connected to an auxiliary syringe unit (syringe D; 1.0 mL); this is used to flush the mixing and flow cells with clean solvent between kinetic runs, leaving the reagent lines unaffected.

Immediately before the mixing cell, the three reagent lines (A, B, C) are packed concentrically in helical coils, so as to pre-magnestise a reservoir of each stock solution (~ 500 – 700 µL each; volumes slightly different for A, B and C lines); this provides the basis for accurate quantitation during reaction monitoring. Thermostatting is achieved by virtue of an external recirculating heater-chiller (Huber Grande Fleur), which continuously pumps a heat-transfer medium (aqueous ethylene glycol) through the umbilical and thereby directly over the reactant lines and mixing cell. The process temperature reported by the chiller (T_{Ch}) is monitored by virtue of a Pt 100 resistance probe, located in close proximity to the mixing cell.

The SF unit and NMR spectrometer communicate through a trigger signal, which instigates, after a specified trigger delay, the desired pulse sequence once the reaction has been assembled (i.e., the syringes have ceased moving).

Typical solution preparation

The follow sections outline details of solution preparation (**Scheme S2**), instrument setup and reaction assembly for a model reaction: the aminolysis of 1-acetyl-1,2,4-triazole **4a**$_{\mathbf{Ac}}$ with **2**. Under the various

conditions reported in this thesis, this reaction is typically complete in < 1 min – and often much less in the presence of a strong auxiliary base, e.g. DBU **3**.

A standard stock solution (250 mL, 0.0500 ± 0.0010 M) of 1-fluoronaphthalene (> 99%) in anhydrous MeCN was prepared using volumetric glassware (Solution V). The 1-fluoronaphthalene was dispensed directly to a volumetric flask using a gas-tight glass syringe, and the flask weighed *in situ* using a high precision analytical balance (0.01 mg resolution) to accurately determine the final concentration. All subsequent stock solutions were prepared using this internal standard solution.

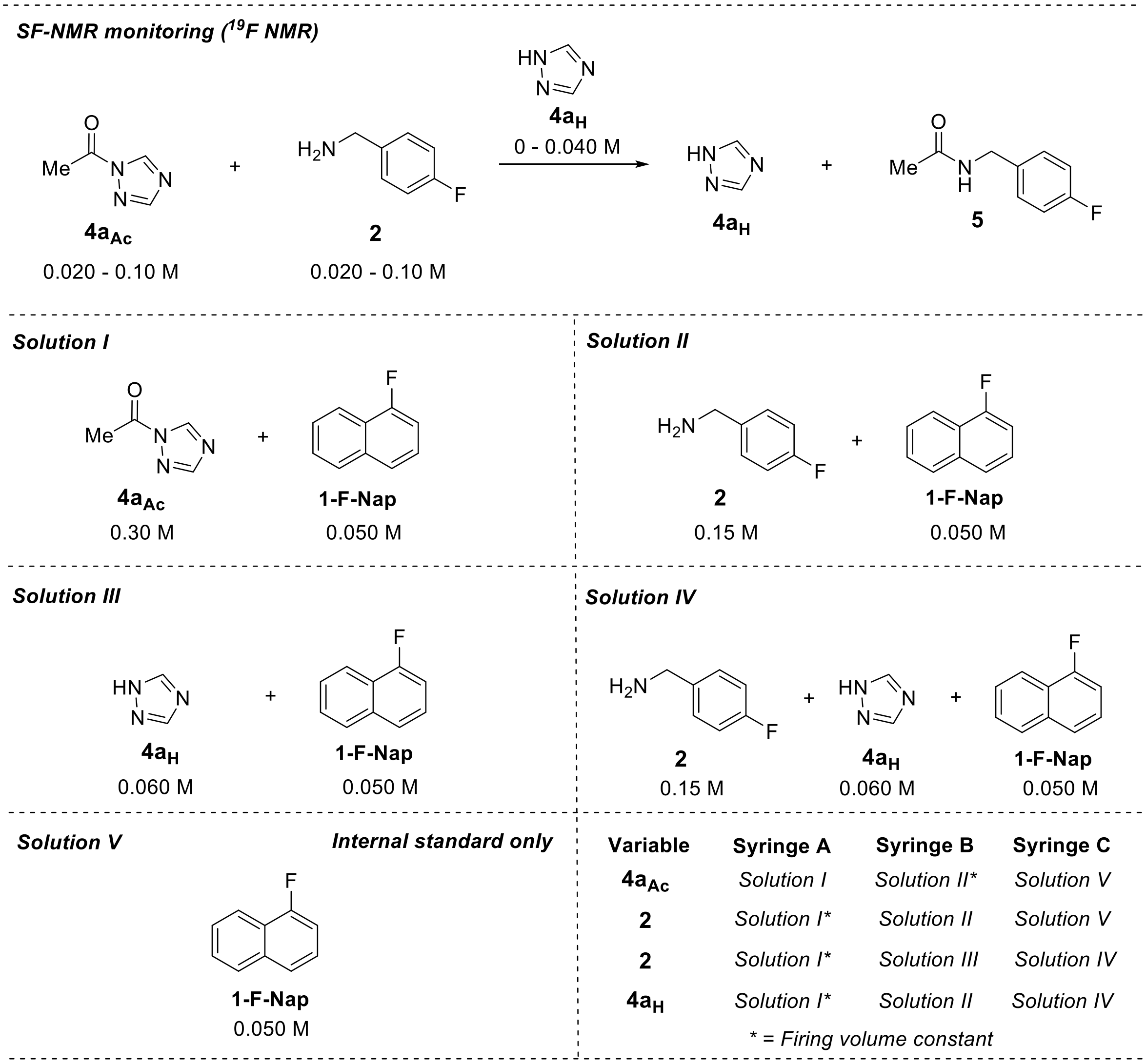

Variable	Syringe A	Syringe B	Syringe C
$4a_{Ac}$	*Solution I*	*Solution II**	*Solution V*
2	*Solution I**	*Solution II*	*Solution V*
2	*Solution I**	*Solution III*	*Solution IV*
$4a_{H}$	*Solution I**	*Solution II*	*Solution IV*

** = Firing volume constant*

Scheme S2: Typical solution preparation for variable ratio SF-^{19}F NMR monitoring, in this case for monitoring the (auto-catalysed) aminolysis 1-acetyl-1,2,4-triazole **$4a_{Ac}$** by *p*-F-BnNH$_2$ **2**. Concentrations shown reflect typical conditions. Reactions monitored in either MeCN or THF. Final section shows the nature of syringe loading that would be required to determine the kinetic order independently for each component, using solutions I-V. In each case, the firing volume of one syringe, and the total volume, would be kept constant, whilst the firing volumes of the other two syringes would be varied. With this particular combination of solutions, the kinetic order in **2** can be analysed in either the absence (solutions I, II, V) or presence (I, III, IV) of exogeneous 1,2,4-triazole **$4a_{H}$**.

Using the parent stock solution of 1-fluoronaphthalene (0.0500 M; solution V), smaller stock solutions (50 mL) of (I) 1-acetyl-1,2,4-triazole **4a$_{Ac}$** (0.30 M) + 1-fluoronaphthalene (0.050 M); (II) 4-fluorobenzyl amine **2** (0.15 M) + 1-fluoronaphthalene (0.050 M); and (III) 1-2-4-triazole **4a$_H$** (0.060 M) + 1-fluoronaphthalene (0.050 M) were prepared in volumetric glassware. 1-Acetyl-1,2,4-triazole **4a$_{Ac}$** was stored and weighed in a N_2-filled glovebox, but its solution was prepared (quickly) under ambient conditions; 4-fluorobenzyl amine **2** was dispensed under ambient conditions using a gas-tight glass microsyringe, and 1,2,4-triazole **4a$_H$** was weighed under ambient conditions. A fourth solution of (IV) 4-fluorobenzyl amine **2** (0.15 M) + 1,2,4-triazole **4a$_H$** (0.060 M) + 1-fluoronaphthalene (0.050 M) was subsequently prepared (25 mL) from solution (II).

All four solutions (I) – (IV) were subsequently transferred to oven-dried Schott reagent bottles (50 mL), which were subsequently sealed using three valve caps with threaded ports (Diba Labware: QSeries cap, GL32, 3 ports + valves). A fifth Schott reagent bottle (50 mL) was filled with the standard solution of (v) 1-fluoronaphthalene (0.050 M). A final, larger Schott reagent bottle (250 mL; 4 ports + valves) was filled with anhydrous MeCN only, without any internal standard.

^{19}F NMR spectra, alongside ^{1}H NMR spectra with and without solvent suppression, were then acquired for all five stock solutions (I) – (V), using standard acquisition parameters (*vide supra*), in order to confirm their purity and determine the actual stock concentrations of the various components. Samples were taken directly from the Schott reagent bottles, after the stock solutions had been transferred from their original volumetric flasks. All stock solutions were subsequently analysed by ^{1}H and ^{19}F NMR spectroscopy later in the day, after all kinetic runs had been completed, to assess their stability under ambient conditions.

In general, none of the stock solutions (I) – (V) displayed any discernible degradation over durations as long as 8 h when stored under ambient conditions, and even when connected to the VR-SF-NMR syringe drive. Moreover, for a given set of solutions the reaction kinetics remained reproducible over the same period, provided care was taken to ensure there was no significant ingress of moisture into any of the reagent lines. The same checks were undertaken for all reactions monitored by VR-SF-NMR.

Varying trace concentrations of residual acetic acid in *different* stock solutions of 1-acetyl-1,2,4-triazole **4a$_{Ac}$** led to minor variations in reaction kinetics *between* experiments – i.e., between monitoring runs conducted on different days – but generally the effect on the reaction evolution was minor, and in any case only apparent in the absence of a strong exogeneous base such as DBU (acetic acid is a competent tautomeric catalyst in the neutral charge state only). Fortunately, tautomeric autocatalysis by 1,2,4-triazole **4a$_H$**, liberated *in situ* by aminolysis, largely outcompeted this effect, provided the concentration of acetic acid was kept sufficiently low. To minimise the residual concentration of acetic acid in 1-acetyl-1,2,4-triazole **4a$_{Ac}$**, the crude product was *doubly* sublimed *in vacuo* following its initial preparation, stored in the freezer of an N_2-glovebox, and re-sublimed again if left for more than 1 month. To the same end, N-acetyl pyrazole **4b$_{Ac}$** was doubly distilled after synthesis and stored in the fridge in a sealed vial.

Typical instrument setup

Prior to the loading of stock solutions, the external heater-chiller and NMR probe were both set to the same temperature (293.1 K; 20.0°C), with the umbilical remaining outside the spectrometer. Whilst waiting for the temperatures of both instruments to equilibrate, the 5 V trigger output from the syringe drive unit was connected to the spectrometer console via a BNC/SMA adaptor (acquisition rack, T-controller, input 'V', pin 1; SMA connector); the syringe drive unit was also connected to a PC installed with KinetaDrive (TgK) software, from which all subsequent commands (syringe firing, reloading, system flushing) were issued.

The sealed bottle of MeCN was then connected to all three reagent inlet lines (A, B, C) of the syringe drive unit *via* standard HPLC adaptors, and all four valves on the bottle cap were opened. Using KinetaDrive software, the three syringes were loaded automatically with MeCN and then fired in their entirety in one shot [total shot volume V_T = 7500 µL; syringe ratios f_A = 0.333 (2500 µL), f_B = 0.333 (2500 µL), f_C = 0.333 (2500 µL)] to remove hexane from the reagent lines, mixing cell, and flow cell (the system was stored under hexane whilst out of use). The reagent syringes were refilled with MeCN and fired a further four times (4 x 7500 µL total), using the same protocol. The three reagent lines were then disconnected from the Schott bottle of MeCN and the flush inlet line (D) connected; the flush syringe (1000 µL) was then filled with MeCN and fired, with this flushing cycle repeated six times (6 x 1000 µL).

After ensuring there were no leaks / compromised connections, the syringe drive unit was manoeuvred into position, and the umbilical lowered through the sample transit tube (STT) of the NMR spectrometer until the flow cell was positioned appropriately in the NMR probe head. With the umbilical docked, the probe was tuned to ^{1}H, matched, and shimmed (^{1}H) *without* a deuterium lock, the receiver gain optimised automatically, and a ^{1}H NMR spectrum recorded using the conventional (*vide supra*) acquisition parameters to assess the spectral resolution and lineshape, and purity of sample in the flow cell (to check for contamination from, e.g., internal leaks of H_2O/ethylene glycol).

Next, stock solution (I) was connected to the inlet line of syringe A and two valves on the bottle cap were opened, including the valve at the connection to the inlet line and one other; stock solutions (II) and (IV) were similarly connected to the inlet lines of syringes B and C, respectively. Stock solutions (II) and (V) were reserved for later kinetic runs. Using the KinetaDrive software, all three reagent syringes (A, B, and C) were automatically loaded with their respective solutions, and then fired simultaneously, in their entirety, in one shot [total shot volume V_T = 7500 µL; syringe ratios f_A = 0.333 (2500 µL), f_B = 0.333 (2500 µL), f_C = 0.333 (2500 µL)]; all three syringes were then reloaded and fired a further two times, using the same protocol. After the third firing, the sample was subjected to further gradient shimming (^{1}H) without a deuterium lock, and a second ^{1}H NMR spectrum was recorded.

For ^{19}F monitoring experiments, the probe was then tuned/matched accordingly, the receiver gain optimised automatically, and a conventional ^{19}F NMR spectrum recorded. Finally, all three syringes were fully reloaded, and the mixing and flow cells flushed with MeCN-h_3 via syringe D (3 x 1 mL).

Typical kinetic runs

All kinetic runs were initiated using a pseudo2D triggered pulse sequence, with a total shot volume (V_T = 600 μL) of *twice* the flow cell volume (V_{FC} = 300 uL) and a flow rate of 1.0 mL s^{-1} used for all runs. To ensure pre-magnetisation of the reactants, no single syringe was used to fire less than 40 μL, or more than 400 μL, in a single shot (all three inlet lines hold a reservoir of ≥ 500 μL pre-magnetised solution). Production-quality kinetic runs were only initiated once the temperature of the NMR probe (T_{NMR}) and external chiller (T_{Ch}) had both equilibrated to within ± 0.2 °C of the target temperature, typically but not invariably 293.1 K (20.0 °C).

For each run the total volume V_T was assembled from the individual volumes (V_A, V_B, V_C) fired from each of the syringes (A, B, C); in practise, the composition of each shot was controlled by manipulating the fractional contributions of each syringe (f_A, f_B, f_C) using KinetaDrive software, whereby:

$$V_T = V_A + V_B + V_C = (f_A + f_B + f_C)V_T \qquad (S2)$$

In this way, the initial concentration of any component *x* in the fully assembled reaction, $[x]_0$, was equal to a linear combination of the stock concentrations of that component in each of the syringes, $[x]_s$ (s = A, B, C).

$$[x]_0 = f_A.[x]_A + f_B.[x]_B + f_C.[x]_C \qquad (S3)$$

Once all three syringes were loaded with the appropriate stock solutions (*vide supra*), the fractional contributions of the three syringes (f_A, f_B, f_C) were configured to achieve the desired reaction composition (see Tables S1-S4), the mixing and flow cells flushed with anhydrous MeCN, and the triggered pulse sequence primed (*via* the zg command).

When ready, the syringes were fired to generate a shot of total volume 600 uL; once the syringes had ceased moving (t_{Sy} ≈ 0.140 s), and following additional trigger (t_{D20} = 0.040 s) and data writing (t_{DW} = 30 ms) delays, reaction monitoring was triggered automatically. With monitoring triggered, ^1H or ^{19}F spectra were acquired continuously for the duration of the experiment, with each spectrum acquired as a single transient using: (i) a 10° excitation pulse; (ii) a relaxation delay of t_{D1} = 0.10 s; and (iii) an acquisition time of t_{AQ} = 1.0 s. Successive spectra were acquired back to back, with a total recycle time of $t_R = t_{AQ} + t_{D1} + t_{DW}$ = 1.13 s and an approximate dead time of $t_{Dead} = t_{Sy} + t_{D20} + t_{DW} \approx 0.21$ s; this includes a standard data writing time (t_{DW} = 0.030 s). The time point corresponding to the nth spectrum was calculated according to $t_n = t_{Dead} + (n-1).t_R$ (n = 1, 2, 3..); unlike conventional reaction monitoring, no t_0 spectrum (n = 0) was acquired.

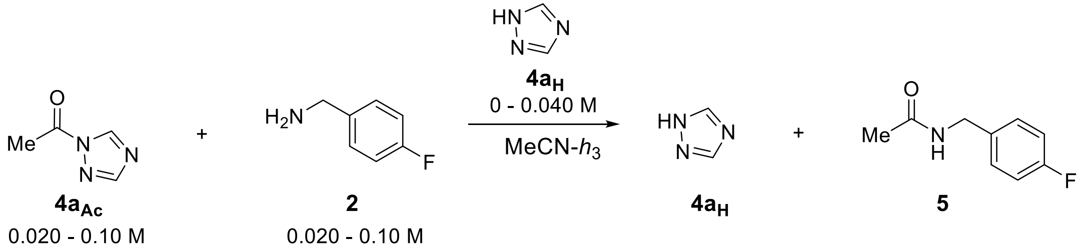

V_T / μL	f_A	V_A / μL	f_B	V_B / μL	f_C	V_c / μL	[**4a$_{Ac}$**]$_0$ / (M)	[**2**]$_0$ / (M)	[**4a$_H$**]$_0$ / (M)
600	0.333	200	0.000	0	0.667	400	0.100	0.100	***0.040***
600	0.333	200	0.133	80	0.533	320	0.100	0.100	***0.032***
600	0.333	200	0.267	160	0.400	240	0.100	0.100	***0.024***
600	0.333	200	0.467	280	0.200	120	0.100	0.100	***0.012***
600	0.333	200	0.533	320	0.133	80	0.100	0.100	***0.008***
600	0.333	200	0.600	360	0.067	40	0.100	0.100	***0.004***

Table S1: Fractional syringe contributions (f_i), syringe firing volumes (V_i / μL) and initial reactant concentrations $[i]_0$ for SF-NMR monitoring of the aminolysis of 1-acetyl-1,2,4-triazole **4a$_{Ac}$** (0.10 M) with **2** (0.10 M) and variable concentrations of exogeneous 1,2,4-triazole **4a$_H$** (0 – 0.040 M). Syringe A filled with solution I; syringe B with solution II; syringe C with solution (IV). [**4a$_{Ac}$**]$_A$ = 0.300 M; [**2**]$_B$ = [**2**]$_C$ = 0.150 M; [**4a$_H$**]$_C$ = 0.060 M. [**1**-F-Nap]$_A$ = [1-F-Nap]$_B$ =[1-F-Nap]$_C$ = 0.050 M.

V_T / μL	f_A	V_A / μL	f_B	V_B / μL	f_C	V_c / μL	[**4a$_{Ac}$**]$_0$ / (M)	[**2**]$_0$ / (M)
600	0.333	200	0.667	400	0.000	0	0.100	***0.100***
600	0.333	200	0.533	320	0.133	80	0.100	***0.080***
600	0.333	200	0.400	240	0.267	160	0.100	***0.060***
600	0.333	200	0.267	160	0.400	240	0.100	***0.040***
600	0.333	200	0.133	80	0.533	320	0.100	***0.020***

Table S2: Fractional syringe contributions (f_i), syringe firing volumes (V_i / μL) and initial reactant concentrations $[i]_0$ for SF-NMR monitoring of the aminolysis of 1-acetyl-1,2,4-triazole **4a$_{Ac}$** (0.10 M) with variable concentrations of *p*-F-BnNH$_2$ **2** (0.020 – 0.10 M) but no exogenous 1,2,4-triazole **4a$_H$**. Syringe A filled with solution I; syringe B with solution II; syringe C with solution (V). [**4a$_{Ac}$**]$_A$ = 0.300 M; [**2**]$_B$ = 0.150 M. [1-F-Nap]$_A$ = [1-F-Nap]$_B$ =[1-F-Nap]$_C$ = 0.050 M

V_T / uL	f_A	V_A / uL	f_B	V_B / uL	f_C	V_c / uL	[**4a$_{Ac}$**]$_0$ / (M)	[**2**]$_0$ / (M)
600	0.333	200	0.667	400	0.000	0	***0.100***	0.100
600	0.267	160	0.667	400	0.067	40	***0.080***	0.100
600	0.200	120	0.667	400	0.133	80	***0.060***	0.100
600	0.133	80	0.667	400	0.200	120	***0.040***	0.100
600	0.067	40	0.667	400	0.267	160	***0.020***	0.100

Table S3: Fractional syringe contributions (f_i), syringe firing volumes (V_i / µL) and initial reactant concentrations $[i]_0$ for SF-NMR monitoring of the aminolysis of variable 1-acetyl-1,2,4-triazole **4a$_{Ac}$** (0.020 - 0.10 M) with *p*-F-BnNH$_2$ **2** (0.10 M) but no exogenous 1,2,4-triazole **4a$_H$**. Syringe A filled with solution I; syringe B with solution II; syringe C with solution (V). [**4a$_{Ac}$**]$_A$ = 0.300 M; [**2**]$_B$ = 0.150 M. [1-F-Nap]$_A$ = [1-F-Nap]$_B$ =[1-F-Nap]$_C$ = 0.050 M.

V_T / µL	f_A	V_A / µL	f_B	V_B / µL	f_C	V_c / µL	[**4a$_{Ac}$**]$_0$ / (M)	[**2**]$_0$ / (M)	[**4a$_H$**]$_0$ / (M)
600	0.333	200	0.667	400	0.000	0	0.100	***0.100***	0.040
600	0.333	200	0.533	320	0.133	80	0.100	***0.080***	0.040
600	0.333	200	0.400	240	0.267	160	0.100	***0.060***	0.040
600	0.333	200	0.267	160	0.400	240	0.100	***0.040***	0.040
600	0.333	200	0.133	80	0.533	320	0.100	***0.020***	0.040

Table S4: Fractional syringe contributions (f_i), syringe firing volumes (V_i / µL) and initial reactant concentrations $[i]_0$ for SF-NMR monitoring of the aminolysis of 1-acetyl-1,2,4-triazole **4a$_{Ac}$** (0.10 M) with variable concentrations of **2** (0.020 – 0.10 M) in the presence of exogenous 1,2,4-triazole **4a$_H$** (0.040 M). Syringe A filled with solution I; syringe B with solution III; syringe C with solution (IV). [**4a$_{Ac}$**]$_A$ = 0.300 M; [**2**]$_B$ = 0.150 M; [**4a$_H$**]$_B$ = [**4a$_H$**]$_C$ = 0.060 M. [1-F-Nap]$_A$ = [1-F-Nap]$_B$ =[1-F-Nap]$_C$ = 0.050 M.

Acquisition parameters

Some reactions monitored by SF-NMR remained incomplete after > 30 min, whilst others – such as those above – attained full conversion in < 30 s. Accordingly, acquisition parameters for VR-SF-NMR experiments (i.e., excitation flip angle, t_{D1}, t_{AQ}) were generally optimised on a case-by-case basis, in an attempt to balance high signal-to-noise with accurate quantitation. Optimised acquisition parameters, once identified, were held constant *within* series of kinetic runs, e.g., for the determination of a kinetic orders under a specific set of conditions, to ensure some degree of systematic error cancellation.

In general, acquisition parameters were optimised within the following ranges: (i) excitation pulses with flip angles of 10° - 30°; (ii) relaxation delays of t_{D1} = 0.10 – 10.0 s; and (iii) acquisition times of t_{AQ} = 1.0 – 2.0 s. The very fastest reactions (*vide supra*) were monitored only by SF-^{19}F NMR, using a 10° excitation pulse, a relaxation delay of t_{D1} = 0.10 s, and an acquisition time of t_{AQ} = 1.0 s (*vide infra*); the slowest were monitored by VR-SF-^{1}H or VR-SF-^{19}F NMR, using a 30° excitation pulse, a relaxation delay of t_{D1} = 10.0 s and an acquisition time of t_{AQ} = 2.0 s. Others were monitored using intermediate acquisition parameters.

In all VR-SF-NMR experiments, especially those involving particularly rapid reactions and notably short recycle times (i.e., $t_R \approx 1.1$ s), the summed concentrations of related components (e.g., [**2**] + [**5**] ; or [**1**] + [**6**]$_T$) were monitored carefully to ensure that integral quantitation was not significantly compromised by differential rates of longitudinal relaxation in substrate, product, or standard nuclei. Independent measurements of longitudinal relaxation time constants (T_1) were undertaken to corroborate these assessments (*vide infra).*

S3.4. Longitudinal relaxation times (T_1)

General comments

To guide the optimisation of acquisition parameters for NMR reaction monitoring (*vide supra*), longitudinal relaxation time constants (T_1) were measured explicitly for key nuclei in a range of major reaction components. Relaxation time constants for ^{19}F and/or ^{1}H nuclei in each compound were measured by conventional inversion-recovery experiments {t_{D1}--π--τ--π/2--t_{AQ}}, using pure (single-component) samples prepared in MeCN-d_3. In each experiment, the recovery of longitudinal magnetisation was monitored by repeating the pulse sequence {t_{D1}--π--τ--π/2--t_{AQ}} for a standardised list of 14 recovery delays (τ = 0.01 s, 0.1 s, 0.5 s, 1.0 s, 1.5 s, 2.0 s, 2.5 s, 4.0 s, 6.0 s, 8.0 s, 10 s, 15 s, 20 s, 40 s), acquiring an integer multiple of four transients per spectrum (ns = 4 for ^{1}H; ns = 8 for ^{19}F), and imposing an extended relaxation delay of t_{D1} = 40 s between successive transients.

All samples were prepared and analysed analogously, using the same component concentration (0.100 M), solvent source, and NMR spectrometer (Bruker Ascend 400 MHz). All samples were assembled in the same manner as monitored reactions, prepared at pertinent concentrations, and analysed at the same temperature (T_{NMR} = 293.1 K). Both ^{1}H and ^{19}F inversion-recovery experiments were undertaken for *p*-F-PhAc **1**, *p*-F-BnNH$_2$ **2**, *p*-F-BnNHAc **5**, *p*-F-PhOH **6$_H$**, and 1-F-naphthalene 1-F-Nap; ^{1}H experiments only were conducted for 1,2,4-triazole **4a$_H$**, pyrazole **4b$_H$**, N-acetyl pyrazole **4b$_{Ac}$** and 1,3,5-trimethoxybenzene 1,3,5-TMB, in addition to an equimolar mixture of 1,2,4-triazole **4a$_H$** and DBU **3** (0.10 M : 0.10 M) (Table S5).

Relaxation time constants were extracted from plots of resonance intensities ($I_τ$) *vs* τ by non-linear regression (Figure S1); to account for imperfect inversions from the initial 180° (π) pulse, a three-parameter model (I_∞, k, T_1) was used in all cases (Equation S4).

$$I_\tau = I_\infty\left(1 - ke^{-\frac{\tau}{T_1}}\right) \qquad (S4)$$

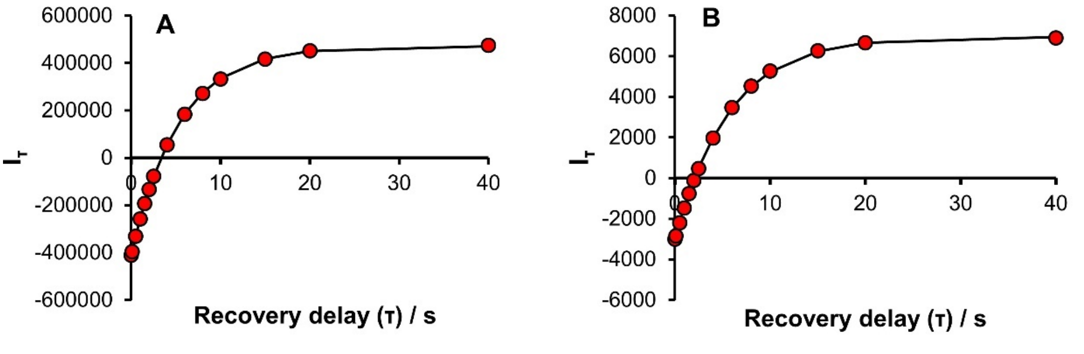

Figure S1: Typical inversion recovery profiles for: (A) 1,3,5-trimethoxybenzene 1,3,5-TMB (T_1(ArH) = 5.3 s; k = 1.87, I_∞ = 471794), measured by ^{1}H NMR; and (B) **5** (T_1(F) = 5.7 s, k = 1.44, I_∞ = 6695), measured by ^{19}F NMR spectroscopy. All T_1 constants measured at T_{NMR} = 293.1 K in pure (single-component) MeCN-d_3 solutions.

Species	δ_H / ppm	T_1 (^{1}H) / s	δ_F / ppm	T_1 (^{19}F) / s
p-F-PhAc **1**	2.26 (C*H*$_3$)	4.2	-118.87	5.3
	7.15 (Ar-*H*)	6.2	-	-
p-F-BnNH$_2$ **2**	3.79 (C*H*$_2$)	4.3	-118.76	5.6
	7.07 (Ar-*H*)	6.3	-	-
	7.36 (Ar-*H*)	6.3	-	-
p-F-BnNHAc **5**	1.92 (C*H*$_3$)	4.3	-117.70	5.7
	4.31 (C*H*$_2$)	3.7	-	-
	7.08 (Ar-*H*)	6.2	-	-
	7.31 (Ar-*H*)	6.2	-	-
p-F-PhOH **6$_H$**	6.80 (Ar-*H*)	7.2	-127.08	6.3
	6.97 (Ar-*H*)	7.2	-	-
1-F-Naphthalene **1-F-Nap**	7.25 (Ar-*H*)	7.3	-125.02	4.8
	7.50 (Ar-*H*)	7.1	-	-
	7.63 (Ar-*H*)	7.1	-	-
	7.75 (Ar-*H*)	7.0	-	-
	7.98 (Ar-*H*)	7.1	-	-
	8.12 (Ar-*H*)	7.1	-	-
1,3,5-Trimethoxybenzene **1,3,5-TMB**	3.78 (C*H*$_3$)	2.7	-	-
	6.12 (Ar-*H*)	5.3	-	-
Pyrazole **4b$_H$**	6.32 (Ar-*H*)	8.4	-	-
	7.59 (Ar-*H*)	8.6	-	-
N-Acetyl pyrazole **4b$_{Ac}$**	2.67 (C*H*$_3$)	5.8	-	-
	6.53 (Ar-*H*)	7.7	-	-
	7.76 (Ar-*H*)	8.2	-	-
	8.28 (Ar-*H*)	8.2	-	-
1,2,4-Triazole **4a$_H$**	8.15 (Ar-*H*)	8.1	-	-
**4a$_H$** (+ DBU **3**, 1:1)	7.95 (Ar-*H*)	7.5	-	-

Table S5: Longitudinal relaxation (T_1) constants for a range of key species pertinent to the main text. All T_1 constants were measured independently by standard inversion recovery experiments (^{1}H or ^{19}F NMR) at T_{NMR} = 293.1 K, using pure (single-component) MeCN-d_3 solutions. A relaxation delay of t_{D1} = 40 s was used in all inversion recovery experiments (> 4 T_1 of the slowest relaxing nucleus).

^{19}F NMR

Inversion-recovery experiments were conducted using eight transients (ns = 8) per spectrum, a relaxation delay of t_{D1} = 40 s between successive transients, and an acquisition time of t_{AQ} = 1.5 s. All 14 spectra were processed as a stack, with baseline (Whittaker smoother) and phasing (zeroth and first order) corrections optimised manually.

Unsurprisingly, longitudinal relaxation rates for the ^{19}F nuclei in *p*-F-PhAc **1**, *p*-F-BnNH$_2$ **2**, *p*-F-BnNHAc **5**, *p*-F-PhOH **6$_H$**, and 1-F-naphthalene 1-F-Nap were found to be almost identical to one another in MeCN-d_3, with the largest difference being that between **6$_H$** (T_1(^{19}F) = 6.3 s) and 1-F-Nap (T_1(^{19}F) = 4.8 s).

^{1}H NMR

Inversion-recovery experiments were conducted using four transients (ns = 4) per spectrum, a relaxation delay of t_{D1} = 40 s between successive transients, and an acquisition time of t_{AQ} = 1.0 s. All 14 spectra were processed as a stack, with baseline (Whittaker smoother) and phasing (zeroth and first order) corrections optimised manually.

Compared to the T_1(^{19}F) constants, the range of T_1 constants observed amongst the various ^{1}H nuclei was larger, with the methyl protons in 1,3,5-trimethoxybenzene 1,3,5-TMB (T_1(^{1}H, CH_3) = 2.7 s) and doubly degenerate aryl protons in pyrazole **4b$_H$** (T_1(^{1}H, Ar-*H*) = 8.6 s) representing limiting cases amongst the compounds analysed. The addition of equimolar DBU **3** to 1,2,4-triazole **4a$_H$** lead only to a modest increase in the rate of longitudinal relaxation of these aryl protons, affording T_1(^{1}H, Ar-*H*) = 7.5 s compared to T_1(^{1}H, Ar-*H*) = 8.1 s under neutral conditions.

Quantitation accuracy

All reactions monitored in this work (conventional and VR-SF-NMR) were monitored *in situ*, without ejection from the NMR probehead, using a continuous loop of the pulse-acquire sequence: {*p1*--t_{AQ}--t_{D1}--t_{MD}}. This sequence consists of: (i) a single, non-selective excitation pulse (p1); (ii) an acquisition time (t_{AQ}); and (iii) a relaxation delay t_{D1} + t_{MD} (t_{MD} = 0 for VR-SF-NMR monitoring; t_{MD} > 5.0 s for conventional monitoring), and overall corresponds to a regular train of excitation pulses separated by a constant recycle time of $t_R = t_{AQ} + t_{D1} + t_{MD}$, i.e., {*p1*--$t_R$}$_n$ (*n* = 1, 2, 3..). In almost all cases, temporal concentration profiles ([x]$_t$ vs *t*) were assembled by acquiring a spectrum with a *single transient* at each time point, $t_n = t_{Dead} + (n\text{-}1).t_R$ (*n* = 1, 2, 3..), with concentrations in turn determined by integration and normalisation against an inert internal standard (*vide supra*).

Notwithstanding random errors arising from processing deficiencies or imperfect signal-to-noise, the accuracy of any given temporal concentration profile acquired in this way will be governed primarily by the *relative magnetisations* of the nuclei in the analyte and internal standard at each time point. For recycle times that are of a similar magnitude to, or shorter than, the longer longitudinal relaxation time constant (i.e., for t_R < 10 T_1 for the *slower* relaxing nucleus), the relative magnetisations of any two nuclei will in general be non-unity and time-dependent, with its value contingent upon the duration of the excitation pulse (the flip angle), recycle time, and any difference in the rate of longitudinal relaxation time constants between the two nuclei. Essentially all reactions monitored in this work fall under this regime (*vide supra*).

The underlying pulse sequence and sample treatment of this monitoring setup are equivalent to those employed in the FLIPS method.[S6] FLIPS was originally devised as an efficient methodology for measuring unknown T_1 constants; however, when used in conjunction with independently measured T_1 constants – determined by, e.g., inversion-recovery – it can also be used to assess the temporal evolution of the bulk magnetisation for any nucleus subjected to a continuous, regular train of excitation pulses (i.e., during reaction monitoring when spectra are acquired using a single transient).

Specifically, the underlying mathematics of FLIPS allows one to calculate the *xy-projection* M_{xy} of the bulk magnetisation vector M_0 for an arbitrary nucleus immediately after excitation, which is in turn proportional to the intensity *I* of the corresponding signal observed in the Fourier-transformed spectrum. Assuming all nuclei are fully magnetised – i.e., at thermal equilibrium – prior to the first excitation pulse ($n = 1$), the *xy*-projection of the bulk magnetisation, $M_{xy,n}^{(\alpha)}$, for a given nucleus α, immediately after the nth excitation pulse, i.e., at $t'_n = (n-1).t_R$ ($n =$ 1, 2, 3…), is given by:

$$M_{xy,n}^{(\alpha)} = \begin{Bmatrix} M_0^{(\alpha)} \sin(\theta), & n = 1 \\ M_0^{(\alpha)} \sin(\theta) \left[1 - \sum_{i=1}^{n-1} \cos^{(i-1)}(\theta)\,(1-\cos(\theta))\, e^{-\frac{i.t_R}{T_{1,\alpha}}} \right], & n \geq 2 \end{Bmatrix} \quad (S5)$$

The xy-magnetisation after the nth excitation pulse, $M_{xy,n}^{(\alpha)}$, can alternatively be derived iteratively:

$$M_{xy,n}^{(\alpha)} = M_0 \sin(\theta) - \left[M_0 \sin(\theta) - M_{xy,n-1}^{(\alpha)} \cos(\theta) \right] e^{-\frac{t_R}{T_{1,\alpha}}}, \qquad n \geq 2 \quad (S6)$$

For recycle times that are of a similar magnitude to, or shorter than, the longitudinal relaxation time constant (i.e., for $t_R < 10\ T_1$), the detected signal will decrease with each excitation pulse, with the system eventually evolving towards a steady state. In this regime, the *xy*-magnetisation $M_{xy,SS}^{(\alpha)}$ remains invariant between successive time points, such that $M_{xy,SS}^{(\alpha)} = M_{xy,n}^{(\alpha)} = M_{xy,n-1}^{(\alpha)}$, giving:

$$M_{xy,SS}^{(\alpha)} = M_0 \sin(\theta) \left(\frac{1 - e^{\frac{t_R}{T_{1,\alpha}}}}{\cos(\theta) - e^{\frac{t_R}{T_{1,\alpha}}}} \right) \quad (S7)$$

To assess the effect of these nuances on monitoring quantitation, the *relative* magnetisation $M_n^{(\alpha/\beta)}$, and corresponding errors $\varepsilon_n^{(\alpha/\beta)}$, of various pairwise combinations of nuclei (α, β) from Table S5 were simulated under a range of real monitoring conditions (*vide supra*). Simulations were conducted for four "worst-case" (i.e., *least* quantitative) scenarios, using pairwise combinations of analyte and standard nuclei with the *most disparate* T_1 constants, and the *minimum recycle times* typically employed for reaction monitoring (conventional NMR: θ = 30°, $t_R(^1H) > 19$ s, $t_R(^{19}F) > 18.5$ s; VR-SF-NMR: θ = 10°, $t_R(^1H, ^{19}F) > 1.1$ s).

Given the underlying proportionality, the relative magnetisation $M_n^{(\alpha/\beta)}$ translates to the relative intensities, $I_n^{(\alpha/\beta)}$, of the signals from nuclei α and β observed in the nth spectrum, such that:

$$M_n^{\left(\frac{\alpha}{\beta}\right)} = I_n^{\left(\frac{\alpha}{\beta}\right)} = \frac{M_{xy,n}^{(\alpha)}}{M_{xy,n}^{(IS)}} = \frac{I_{xy,n}^{(\alpha)}}{I_{xy,n}^{(IS)}} \qquad (S8)$$

$$\varepsilon_n^{\left(\frac{\alpha}{\beta}\right)} = 100\left(I_n^{\left(\frac{\alpha}{\beta}\right)} - 1\right)\% \qquad (S9)$$

As expected, quantitation errors in the relative magnetisation $M_n^{(\alpha/\beta)}$ were found to increase over time, tending towards a maximum in the steady state regime. Under typical conditions for conventional NMR monitoring (30 deg excitation; Figures S2A-B), a steady state is realised within the first few time points; for VR-SF-NMR monitoring (10 deg excitation; Figures S2C-D); of the very fastest reactions – i.e., those that reach full conversion in < 60 s – the relative magnetisation typically evolves throughout the bulk of the reaction, reaching a steady state only in the final stages, if at all.

Crucially, the simulations in Figure S2 show that the *maximum* quantitation error encountered in conventional *in situ* ^{1}H and ^{19}F monitoring, and VR-SF-^{19}F NMR monitoring, ought to be < 2 % for all reactions reported in this work. Even under the most demanding conditions, i.e., VR-SF-^{1}H NMR monitoring of the very fastest reactions (t_R = 1.1 s, θ = 10°), these simulations suggest that the *maximum* quantitation error ought to be < 8 %. In general, quantitation errors arising from differential rates of longitudinal relaxation are much smaller for VR-SF-^{19}F NMR than VR-SF-^{1}H NMR monitoring, given the apparent insensitivity of T_1(^{19}F) constants in aryl fluorides to various forms of aryl substitution; for this reason, VR-SF-^{19}F NMR monitoring was favoured over VR-SF-^{1}H NMR for monitoring the very fastest reactions in this work (i.e, θ = 10°, t_{AQ} = 1.0 s, t_{D1} = 0.1 s). For very fast reactions, VR-SF-^{1}H NMR was only used if absolutely necessary – to extract, e.g., additional information/species not observable by ^{19}F NMR – with corroboration provided in each case by independent VR-SF-^{19}F NMR measurements of the overall reaction evolution.

These simulations are only an indicative guide of the likely quantitation errors encountered during reaction monitoring, primarily because the T_1 constants of compounds measured in single-component solutions are likely to differ to some extent from the true relaxation rates when that component is present alongside many others in a complex mixture. Fortunately, in this respect the simulated quantitation errors above likely constitute *upper bounds* on quantitation errors encountered during reaction monitoring: amongst *small organic molecules* there exists a well-characterised, inverse relationship between longitudinal relaxation time (T_1) constants and solution viscosity, and in general fully assembled reactions in organic solvents are likely to be more viscous than pure solutions of the individual, neutral compounds at comparable concentrations. The formation of salts under standard catalytic conditions would presumably lead to pronounced increases in viscosity, and in turn to materially diminished T_1 constants during reaction monitoring, especially in reactions involving strong organic bases (e.g. DBU **3**).

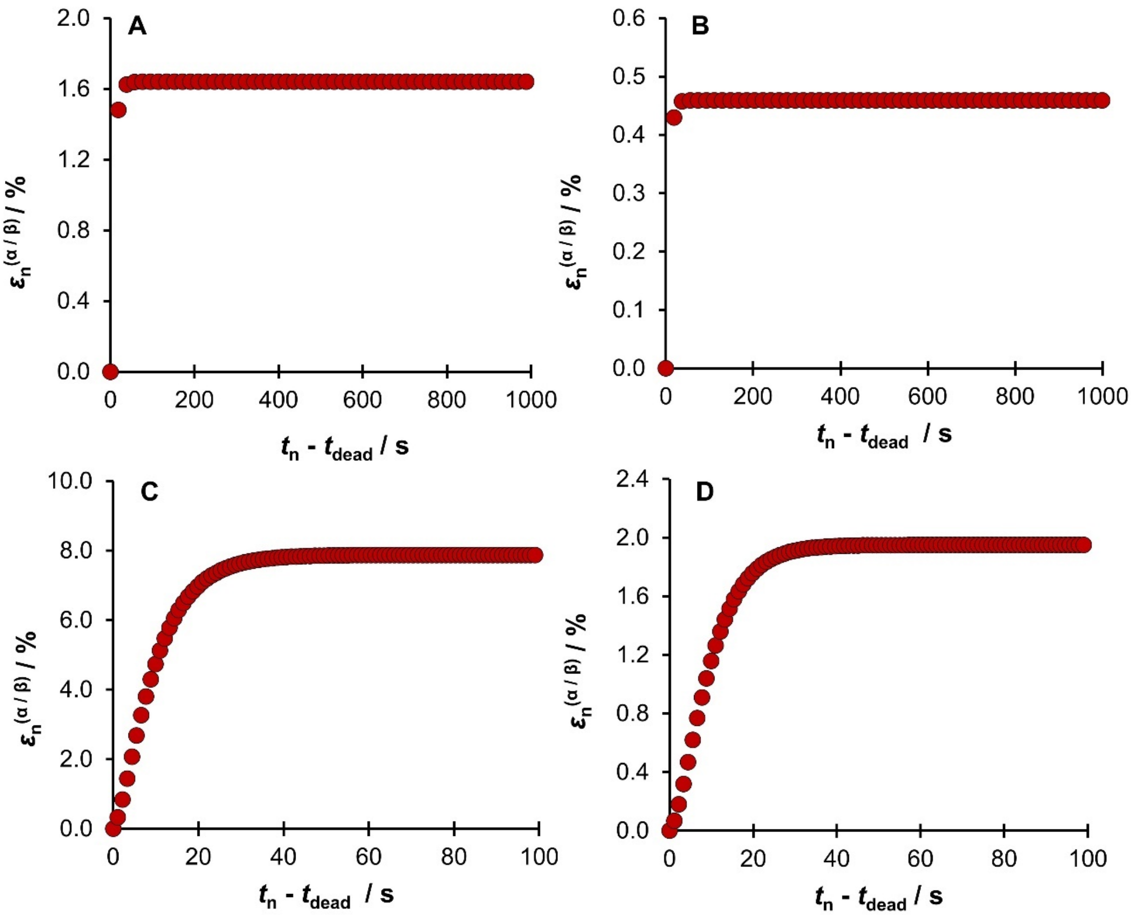

Figure S2: Simulated integration errors, arising from differential rates of longitudinal relaxation, for various limiting NMR reaction monitoring regimes used in this work. Errors pertain to deviations of relative integrals from unity for a given pair of nuclei (α, β) subjected to a continuous pulse-acquire sequence (recycle time t_R, flip angle θ). (A) Errors in relative integrals arising from nuclei with T_1 constants of 2.7 s (i.e., 1,3,5-TMB, C*H*$_3$) and 8.6 s (i.e., pyrazole **4b$_H$**, Ar*H*) (θ = 30°; t_R = 19.0 s; conventional ^{1}H NMR monitoring); (B) Errors in relative integrals arising from nuclei with T_1 constants of 4.8 s (i.e., 1-F-Nap, Ar*F*) and 6.3 s (i.e., **6$_H$**, Ar*F*) (θ = 30°; t_R = 19.0 s; conventional ^{19}F NMR monitoring); (C) Errors in relative integrals arising from nuclei with T_1 constants of 2.7 s (i.e, 1,3,5-TMB, C*H*$_3$) and 8.6 s (i.e., pyrazole **4b$_H$**, Ar*H*) (θ = 10°; t_R = 1.1 s; SF-^{1}H NMR monitoring); (D) Errors in relative integrals arising from nuclei with T_1 constants of 4.8 s (i.e., 1-F-Nap, Ar*F*) and 6.3 s (**6$_H$**, Ar*F*) (θ = 10°; t_R = 1.1 s; SF-^{19}F NMR monitoring). α corresponds to faster relaxing nucleus in each case (i.e., ε > 0).

S3.5. Numerical kinetic analysis

Numerically fitted rate coefficients were generally determined via a least-squares fitting routine implemented using the Levenberg-Marquardt algorithm (tolerance 10^{-4}) for non-linear fitting and the Rosenbrock integration method (accuracy 10^{-3}) for iteratively solving the underlying systems of differential equations. Kinetically minimal process models were used in each case, with telescoped termolecular terms where appropriate, to fit experimental kinetic data. As rate coefficients were often determined by simultaneously fitting the concentration-time profiles of multiple species to a single kinetic model (e.g., **1**, **2**, **5**, $\mathbf{4b_{Ac}}$; Figure 6, main text) – some present at significantly lower concentrations (i.e., $\mathbf{4b_{Ac}}$) than others throughout a given reaction course – all fitted rate constants were determined by minimising the weighted sum of squares (weighted SSQ) in each run.

References in the main text to *global* numerical fitting (e.g., Figure 6, main text) pertain specifically to instances where rate constants were determined by fitting a single set of kinetic parameters to concentration-time data obtained from *multiple reactions*, conducted under identical conditions but differing in the initial conditions of one or more components.

Uncertainties in individual rate constants determined by such numerical fitting were estimated from the calculated 95 % confidence interval, and for rate constants quoted in the main text relative errors were generally < ± 5 %. Higher uncertainties, up to < ± 15 %, were obtained in a limited number of cases.

S3.6. Graphical kinetic analysis

For reactions evolving in accordance with simple rate laws, graphical kinetic analysis was undertaken using the variable time normalization analysis (VTNA) method described by Bures.[S7-S8] Normalization was generally conducted with respect to product ([**5**] or $[\mathbf{6_H}]_T$) formation, with the kinetic effect of each component removed from product evolution profiles by numerical integration.

The kinetic orders of each component were generally determined *independently* of each other, using experimental data obtained from at least four different kinetic runs initiated, with different initial concentrations of that component, but all other aspects remaining constant. Global normalization, and the determination of phenomenological rate coefficients – i.e., k_{obs} – was only undertaken after all – or all but one – of the individual component orders had been determined, or else when component orders in directly comparable systems had already been determined.

For brevity, the x-axes of normalized kinetic plots in which only one component is varied are expressed as $\Sigma\ [A]^{\alpha}\ \Delta t$, where α denotes the order of the given component A.

In such plots, the x-value of each data point i (x_i, [**5**]$_i$), measured at a time t_i after reaction initiation, was calculated in accordance with the trapezium rule:

$$x_i = \sum [\mathrm{A}]^{\alpha}\,\Delta t = \sum_{m=0}^{m=i}\left[\left(\frac{[\mathrm{A}]_i + [\mathrm{A}]_{i-1}}{2}\right)^{\alpha}(t_i - t_{i-1})\right] \qquad (S10)$$

The normalization of kinetic profiles with respect to multiple components was conducted in analogous manner, with the x-axes of such plots generally denoted by $\Sigma\,[\mathrm{A}]^{\alpha}[\mathrm{B}]^{\beta}[\mathrm{C}]^{\gamma}\ldots\,\Delta t$ and the x-values of each data point calculated according to:

$$x_i = \sum [\mathrm{A}]^{\alpha}[\mathrm{B}]^{\beta}[\mathrm{C}]^{\gamma}\,\Delta t = \sum_{i=0}^{n}\left[\left(\frac{[\mathrm{A}]_i + [\mathrm{A}]_{i-1}}{2}\right)^{\alpha}\left(\frac{[\mathrm{B}]_i + [\mathrm{B}]_{i-1}}{2}\right)^{\beta}\left(\frac{[\mathrm{C}]_i + [\mathrm{C}]_{i-1}}{2}\right)^{\gamma}(t_i - t_{i-1})\right] \qquad (S11)$$

S3.7. Steady-state kinetic analysis

Product evolution

Under a steady state regime, and neglecting the effect that populating the acetylated intermediate $\mathbf{4_{Ac}}$ has on [**1**] and [$\mathbf{6_{\{DBUH\}}}$], the rate of product (**5**) evolution according to mechanism in Figure S3 (and Figure 6, main text) may be approximated [S9-S10] by:

$$\frac{d[\mathbf{5}]}{dt} \approx \frac{K_{PT}k_1k_2[\mathbf{1}][\mathbf{2}][\mathbf{3}]^2[\mathbf{4_H}]_T}{(1 + K_{PT}[\mathbf{3}])\left(k_{-1}\left[\mathbf{6_{\{DBUH\}}}\right] + k_2[\mathbf{2}][\mathbf{3}]\right) + K_{PT}k_1[\mathbf{1}][\mathbf{3}]} \qquad (S12)$$

This was derived for the kinetically minimal mechanism in Figure S3, as follows. The fractional populations of $\mathbf{4_H}$, $\mathbf{4_{\{DBUH\}}}$ and $\mathbf{4_{Ac}}$, at steady-state, are given by:

$$\frac{\left[\mathbf{4_{\{DBUH\}}}\right]}{[\mathbf{4_H}]_T} \approx \frac{k_{PT}[\mathbf{3}]\left(k_{-1}\left[\mathbf{6_{\{DBUH\}}}\right] + k_2[\mathbf{2}][\mathbf{3}]\right)}{\Delta} \qquad (S13)$$

$$\frac{[\mathbf{4_H}]}{[\mathbf{4_H}]_T} \approx \frac{k_{-PT}\left(k_{-1}\left[\mathbf{6_{\{DBUH\}}}\right] + k_2[\mathbf{2}][\mathbf{3}]\right)}{\Delta} \qquad (S14)$$

$$\frac{[\mathbf{4_{Ac}}]}{[\mathbf{4_H}]_T} \approx \frac{k_{PT}k_1[\mathbf{1}][\mathbf{3}]}{\Delta} \qquad (S15)$$

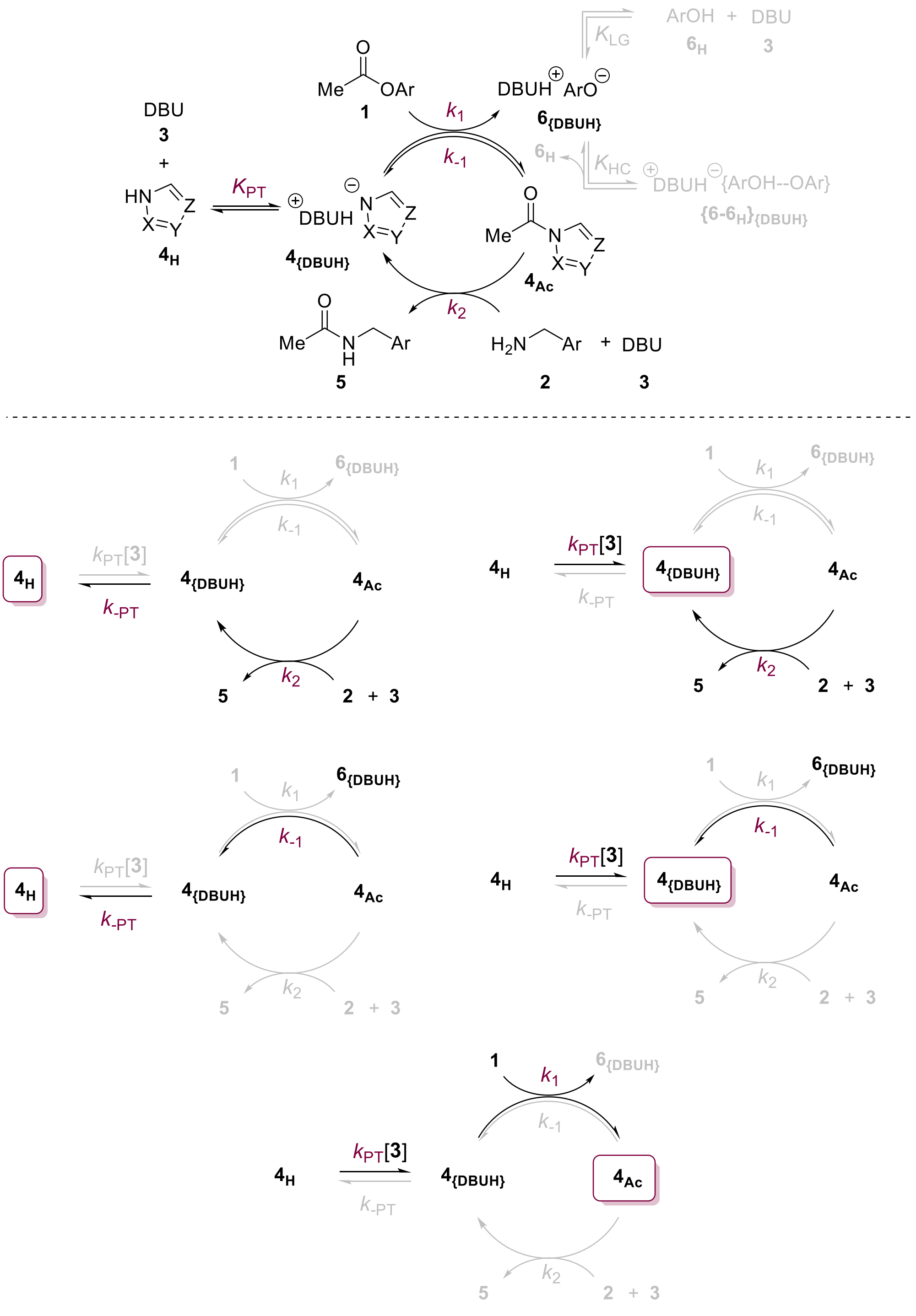

Figure S3: General mechanism for the azole-catalysed aminolysis of phenyl esters under basic conditions, and the five King-Altman patterns used to derive the steady-state evolution of amide **5**.

The kinetic denominator, Δ, is given by the sum of the numerators across these three equations, such that:

$$\Delta = k_{PT}[\mathbf{3}]\left(k_{-1}\left[\mathbf{6}_{\{DBUH\}}\right] + k_2[\mathbf{2}][\mathbf{3}]\right) + k_{-PT}\left(k_{-1}\left[\mathbf{6}_{\{DBUH\}}\right] + k_2[\mathbf{2}][\mathbf{3}]\right) + k_{PT}k_1[\mathbf{1}][\mathbf{3}] \qquad (S16)$$

$$\Delta = (k_{-PT} + k_{PT}[\mathbf{3}])\left(k_{-1}\left[\mathbf{6}_{\{DBUH\}}\right] + k_2[\mathbf{2}][\mathbf{3}]\right) + k_{PT}k_1[\mathbf{1}][\mathbf{3}] \qquad (S17)$$

Simple substitution, and division of both the numerator and denominator by $k_{\text{-PT}}$, in turn affords the rate of product (**5**) evolution, as shown in the main text:

$$\frac{d[\mathbf{5}]}{dt} \approx k_2[\mathbf{2}][\mathbf{3}][\mathbf{4}_{Ac}] \approx \frac{k_{PT}k_1k_2[\mathbf{1}][\mathbf{2}][\mathbf{3}]^2[\mathbf{4}_H]_T}{(k_{-PT} + k_{PT}[\mathbf{3}])\left(k_{-1}\left[\mathbf{6}_{\{DBUH\}}\right] + k_2[\mathbf{2}][\mathbf{3}]\right) + k_{PT}k_1[\mathbf{1}][\mathbf{3}]} \qquad (S18)$$

$$\frac{d[\mathbf{5}]}{dt} \approx \frac{K_{PT}k_1k_2[\mathbf{1}][\mathbf{2}][\mathbf{3}]^2[\mathbf{4}_H]_T}{(1 + K_{PT}[\mathbf{3}])\left(k_{-1}\left[\mathbf{6}_{\{DBUH\}}\right] + k_2[\mathbf{2}][\mathbf{3}]\right) + K_{PT}k_1[\mathbf{1}][\mathbf{3}]} \qquad (S19)$$

Speciation of 6

In the rate equations above, it is the phenolate salt concentration, [$\mathbf{6}_{\{DBUH\}}$], that features in the denominator. The manner in which this term is related to the *total concentration* of evolved phenol, [**6**]$_T$, depends upon the how one models the base-induced homoconjugation of $\mathbf{6}_H$.

If the first-order homoconjugate is modelled as an *ion-paired* species, $\mathbf{\{6\text{-}6_H\}_{\{DBUH\}}}$, the equilibrium constant K_{HC} describes the *association* of $\mathbf{6}_H$ *to* the phenolate salt $\mathbf{6}_{\{DBUH\}}$**.** The speciation of **6** under this regime, excluding the (relatively minor) effect of catalyst ionisation on [**3**], is described by the equations in Figure S4.

Alternatively, if the first-order homoconjugate is modelled as a *free anionic* species, $\mathbf{\{6\text{-}6_H\}^-}$**,** the equilibrium constant K'_{HC} describes the *substitution* of $\mathbf{3}_H^+$ by $\mathbf{6}_H$ *from* the phenolate salt $\mathbf{6}_{\{DBUH\}}$**.** The speciation of **6** under this regime, excluding the (relatively minor) effect of catalyst ionisation on [**3**], is described by the equations in Figure S5.

Speciation of 6 - ion-paired homoconjugate

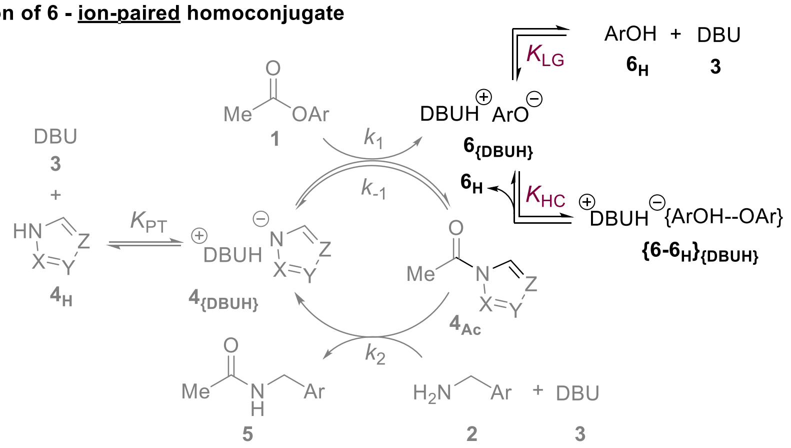

Equilibria

$$K_{LG} = \frac{[\mathbf{6}_{\{DBUH\}}]}{[\mathbf{3}][\mathbf{6}_H]} \qquad K_{HC} = \frac{[\{\mathbf{6}\text{-}\mathbf{6}_H\}_{\{DBUH\}}]}{[\mathbf{6}_{\{DBUH\}}][\mathbf{6}_H]} = \frac{[\{\mathbf{6}\text{-}\mathbf{6}_H\}_{\{DBUH\}}]}{K_{LG}[\mathbf{3}][\mathbf{6}_H]^2}$$

Free phenol

$$\alpha[\mathbf{6}_H]^3 + \beta[\mathbf{6}_H]^2 + \chi[\mathbf{6}_H] + \delta = 0$$

$$\alpha = K_{LG}K_{HC} \qquad \beta = K_{LG}(1 - K_{HC}[\mathbf{6}]_T + 2K_{HC}[\mathbf{3}]_T) \qquad \chi = K_{LG}([\mathbf{3}]_T - [\mathbf{6}]_T) + 1 \qquad \delta = -[\mathbf{6}]_T$$

Homoconjugate

$$[\{\mathbf{6}\text{-}\mathbf{6}_H\}_{\{DBUH\}}] = \frac{K_{LG}K_{HC}[\mathbf{6}_H]^2([\mathbf{3}]_T - [\mathbf{6}]_T + [\mathbf{6}_H])}{1 - K_{LG}K_{HC}[\mathbf{6}_H]^2}$$

Phenolate salt

$$[\mathbf{6}_{\{DBUH\}}] = [\mathbf{6}]_T - [\mathbf{6}_H] - 2[\{\mathbf{6}\text{-}\mathbf{6}_H\}_{\{DBUH\}}]$$

Figure S4: Equations describing the speciation of **6,** under the assumption of universally strong ion-pairing, in the azole-catalysed (**4**$_H$) aminolysis of **1** with **2** and **3** in MeCN (or any solvent in which phenol homoconjugation may be significant). [**6**]$_T$ = [**1**]$_0$ – [**1**] corresponds to the total concentration of evolved **6** at any point in time, and [**3**]$_T$ to the initial base concentration. Equations assume that the catalyst loading is negligible relative to [**3**]$_T$, which is a reasonable but imperfect approximation for the typical loadings in this work.

Speciation of 6 - free homoconjugate

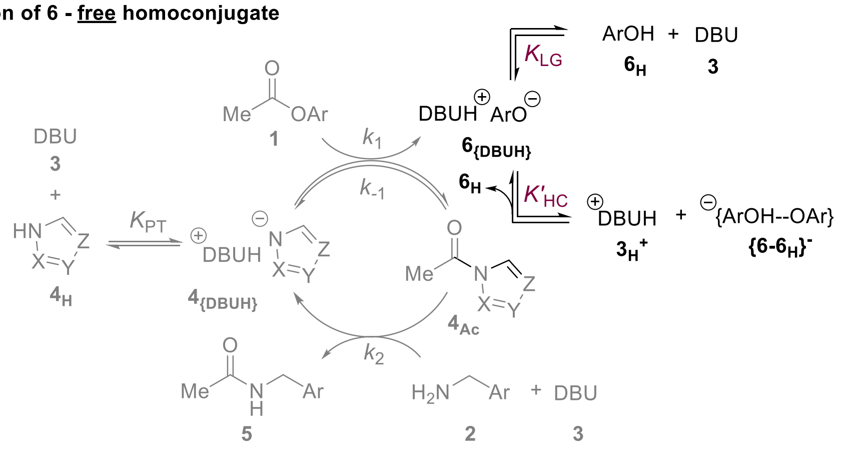

Equilibria

$$K_{LG} = \frac{[\mathbf{6}_{\{DBUH\}}]}{[\mathbf{3}][\mathbf{6}_H]} \qquad K'_{HC} = \frac{[\{\mathbf{6}\text{-}\mathbf{6}_H\}][\mathbf{3}_H^+]}{[\mathbf{6}_{\{DBUH\}}][\mathbf{6}_H]} = \frac{[\{\mathbf{6}\text{-}\mathbf{6}_H\}][\mathbf{3}_H^+]}{K_{LG}[\mathbf{3}][\mathbf{6}_H]^2}$$

Free phenol

$$\alpha[\mathbf{6}_H]^4 + \beta[\mathbf{6}_H]^3 + \chi[\mathbf{6}_H]^2 + \delta[\mathbf{6}_H] + \varepsilon = 0$$

$$\alpha = K^2_{LG}(1 - K'_{HC}) \qquad \beta = K_{LG}\{2 + 2K_{LG}([\mathbf{3}]_T - [\mathbf{6}]_T) + K_{LG}K'_{HC}([\mathbf{6}]_T - 2[\mathbf{3}]_T) - 2K'_{HC}\}$$

$$\chi = K^2_{LG}([\mathbf{6}]^2_T - 2[\mathbf{3}]_T[\mathbf{6}]_T + [\mathbf{3}]^2_T) + 2K_{LG}([\mathbf{3}]_T - 2[\mathbf{6}]_T) + 2K_{LG}K'_{HC}([\mathbf{6}]_T - 2[\mathbf{3}]_T) + 1$$

$$\delta = 2[\mathbf{6}]_T(K_{LG}[\mathbf{6}]_T - K_{LG}[\mathbf{3}]_T - 1) \qquad \varepsilon = [\mathbf{6}]^2_T$$

Homoconjugate

$$[\{\mathbf{6}\text{-}\mathbf{6}_H\}] = \frac{[\mathbf{6}]_T - [\mathbf{6}_H] + K_{LG}[\mathbf{6}_H]([\mathbf{6}]_T - [\mathbf{3}]_T - [\mathbf{6}_H])}{2 + K_{LG}[\mathbf{6}_H]}$$

Phenolate salt

$$[\mathbf{6}_{\{DBUH\}}] = [\mathbf{6}]_T - [\mathbf{6}_H] - 2[\{\mathbf{6}\text{-}\mathbf{6}_H\}]$$

Figure S5: Equations describing the speciation of **6,** under the assumption of *moderate* ion-pairing, in the azole-catalysed ($\mathbf{4}_H$) aminolysis of **1** with **2** and **3** in MeCN (or any solvent in which phenol homoconjugation may be significant). $[\mathbf{6}]_T = [\mathbf{1}]_0 – [\mathbf{1}]$ corresponds to the total concentration of evolved **6** at any point in time, and $[\mathbf{3}]_T$ to the initial base concentration. Equations assume that the catalyst loading is negligible relative to $[\mathbf{3}]_T$, which is a reasonable but imperfect approximation for the typical loadings in this work.

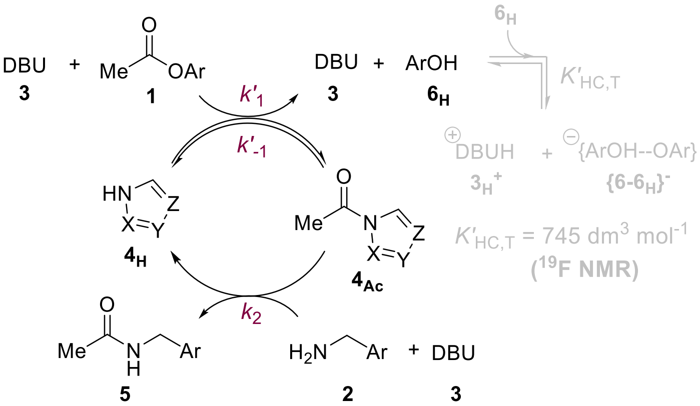

Scheme S3: Telescoped kinetic model used for fitting the experimental kinetics of the catalytic aminolysis of **1** with **2**, **3**, and class β azoles $\mathbf{4_H}$ (pK_a(MeCN) > 25) in MeCN. The speciation of **6** was telescoped by neglecting the concentration of the phenolate salt, [$6_{\{DBUH\}}$], and treating homoconjugation as a global equilibrium, with $[\mathbf{6}]_T \approx [\mathbf{6_H}] + [\mathbf{\{6\text{-}6_H\}_{\{DBUH\}}}]$; the reverse phenolysis of $\mathbf{4b_{Ac}}$ was then modelled as a formally termolecular process with a rate $k'_{-1}[\mathbf{6_H}][\mathbf{3}][\mathbf{4b_{Ac}}]$. $K_{HC,T}$ was estimated independently by ^{19}F NMR titration and non-linear regression of the resulting isotherm to an ion-paired and non-ion paired homoconjugation models; both gave near-identical results in the kinetic fitting.

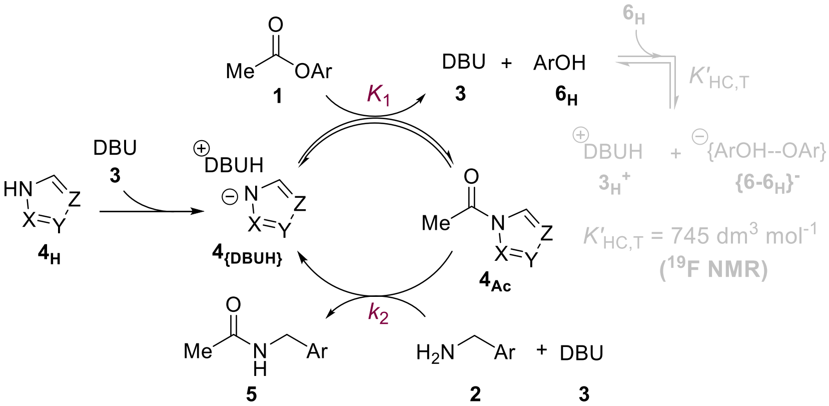

Scheme S4: Telescoped kinetic model used for fitting the experimental kinetics of the catalytic aminolysis of **1** with **2**, **3**, and class α azoles $\mathbf{4_H}$ (pK_a(MeCN) < 25) in MeCN.

S3.8. Reaction Monitoring and Kinetic Analyses

S3.8.1 ¹H and ¹⁹F NMR monitoring: example spectra

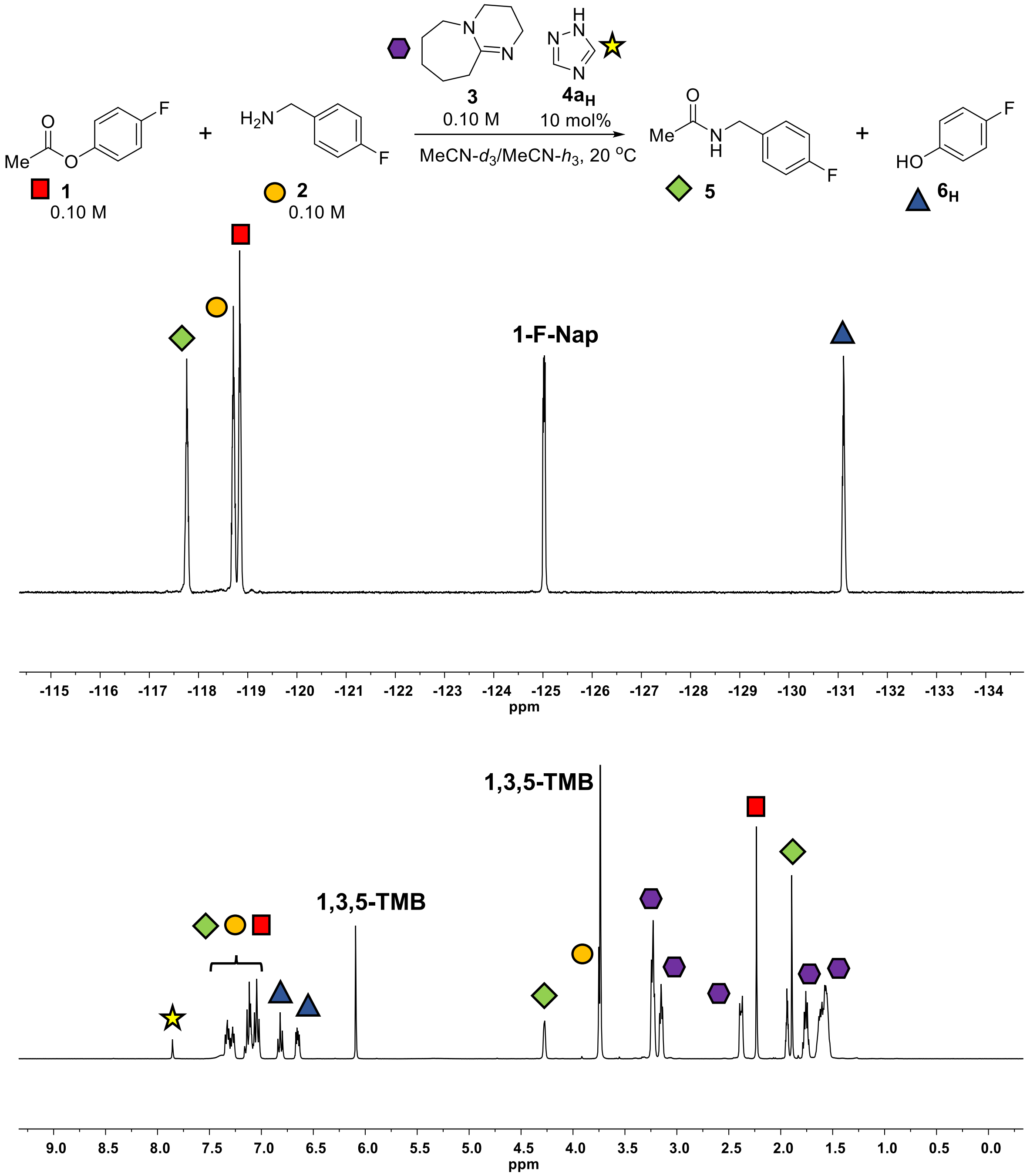

Figure S6: Example ^{19}F and ^{1}H NMR spectra obtained during *in situ* monitoring of the aminolysis of *p*-F-PhAc **1** (0.10 M) with *p*-F-BnNH$_2$ **2** (0.10M), DBU **3** (0.10 M) and 1,2,4-triazole **4a$_H$** (0.010 M) in MeCN-h_3 (^{19}F) and MeCN-d_3 (^{1}H). 1-F-Nap = 1-F-naphthalene (0.050 M). 1,3,5-TMB = 1,3,5-trimethoxybenzene (0.033 M).

S3.8.2 Regime I Kinetics

Raw product evolution profiles

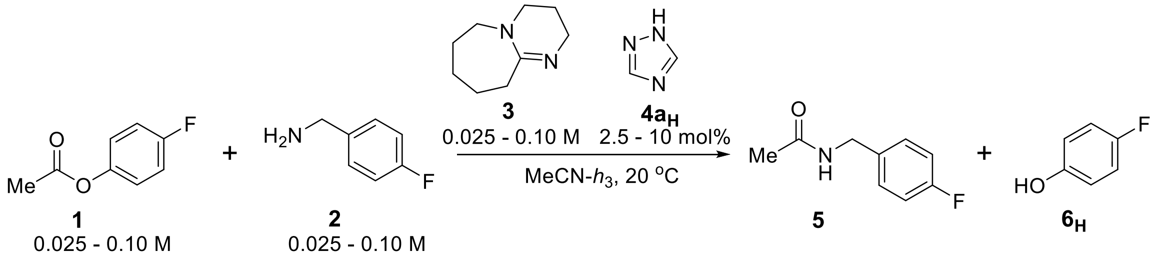

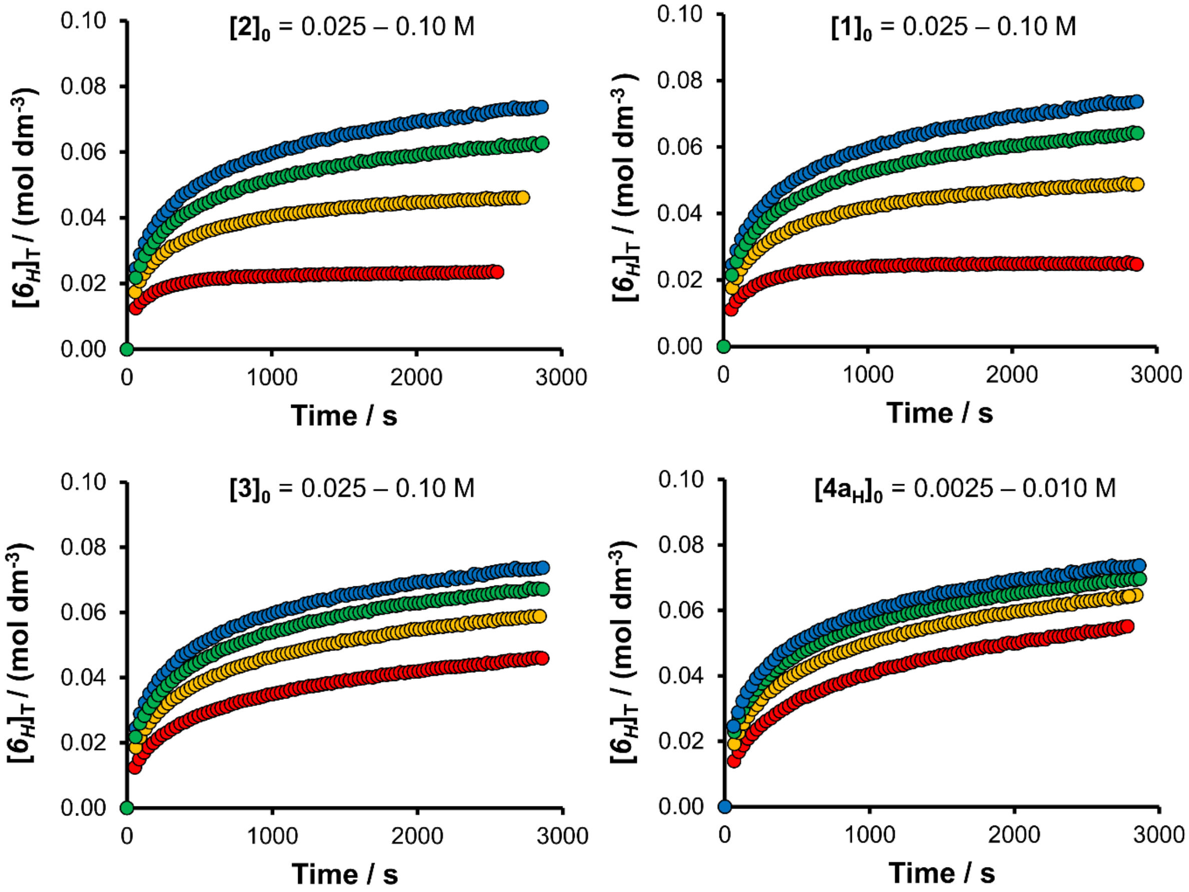

Figure S7: Product evolution profiles for the aminolysis of *p*-F-PhAc **1** with *p*-F-BnNH$_2$ **2** in MeCN at 20 °C, obtained by *in situ* ^{19}F NMR spectroscopy. Product evolution quantified by the *total* concentration of liberated *p*-F-PhOH [**6**$_H$]$_T$ in each kinetic run. (A) Aminolysis of **1** (0.10 M) with variable **2** (0.025 – 0.10 M), DBU **3** (0.10 M) and 1,2,4-triazole **4a**$_H$ (10 mol%); (B) Aminolysis of variable **1** (0.025 - 0.10 M) with **2** (0.10 M), **3** (0.10 M) and **4a**$_H$ (10 mol%); (C) Aminolysis of **1** (0.10 M) with **2** (0.10 M), variable **3** (0.025 - 0.10 M) and **4a**$_H$ (10 mol%); (D) Aminolysis of **1** (0.10 M) with **2** (0.10 M), **3** (0.10 M) and variable **4a**$_H$ (2.5 - 10 mol%).

Normalized product evolution profiles

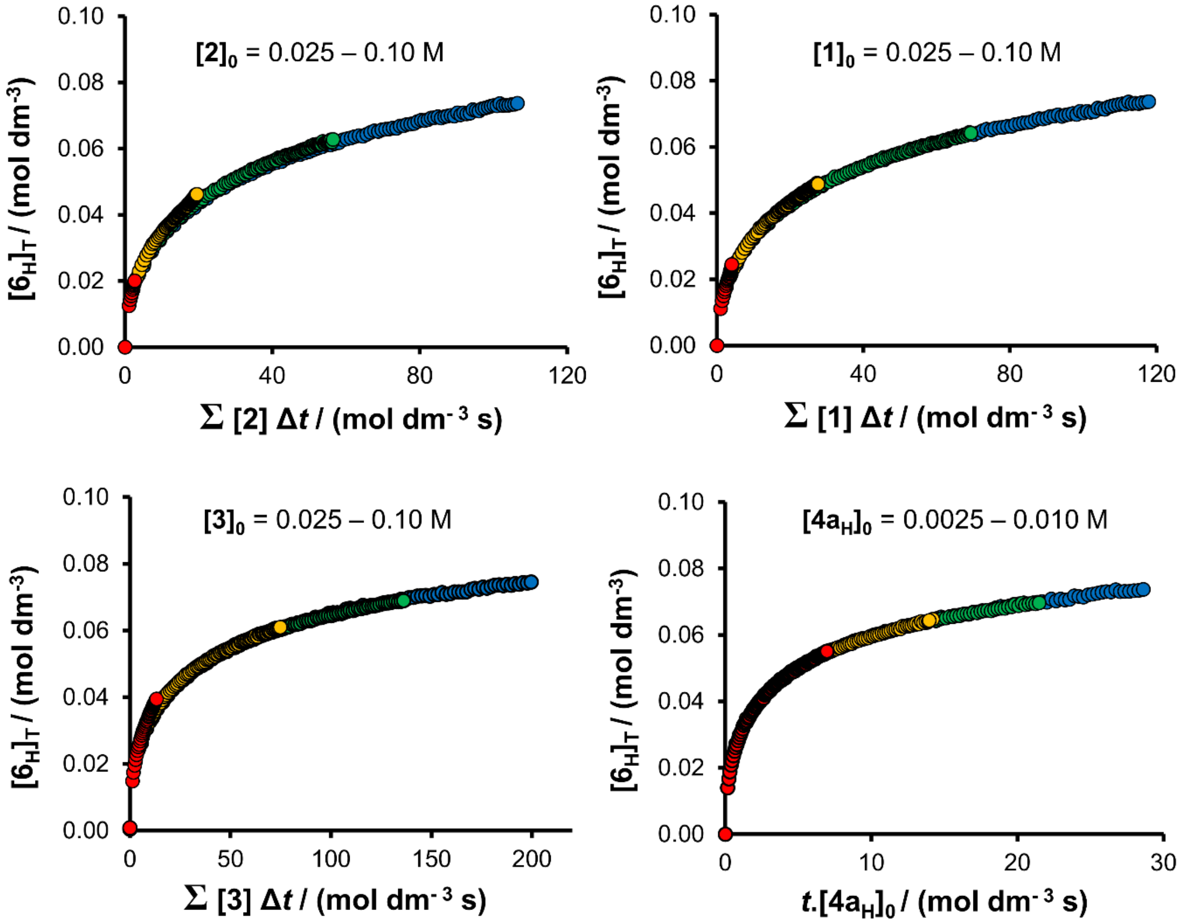

Figure S8: Single-component normalised product evolution profiles for each of the four components involved in the aminolysis of *p*-F-PhAc **1** with *p*-F-BnNH$_2$ **2**, DBU **3** and 1,2,4-triazole **4a**$_H$ in MeCN at 20 °C (raw kinetics: **Figure S7**). Catalyst deactivation is apparently minimal under standard catalytic conditions, such that [**4a**$_{\{DBUH\}}$] ≈ [**4a**$_H$]$_0$. In accordance with detailed equilibrium studies (*vide infra*), the concentration of unionised DBU at each time-point was approximated by [**3**] = [**3**]$_0$ – 0.5.[**6**$_H$]$_T$.

For the global numerical fitting parameters used in Runs 1 to 13 below, see Table S9.

Global numerical fitting: Run 1

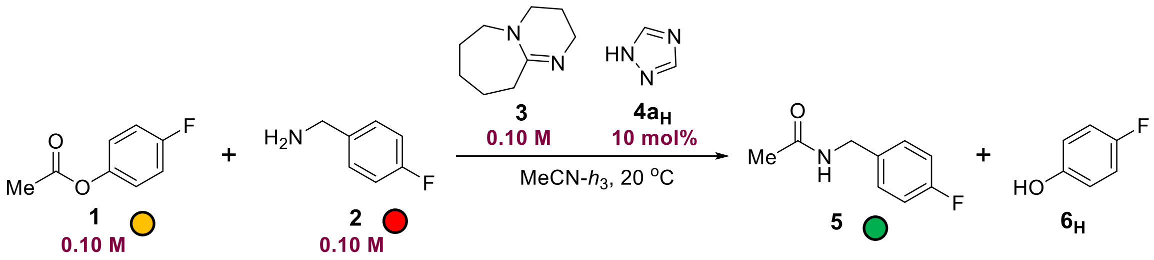

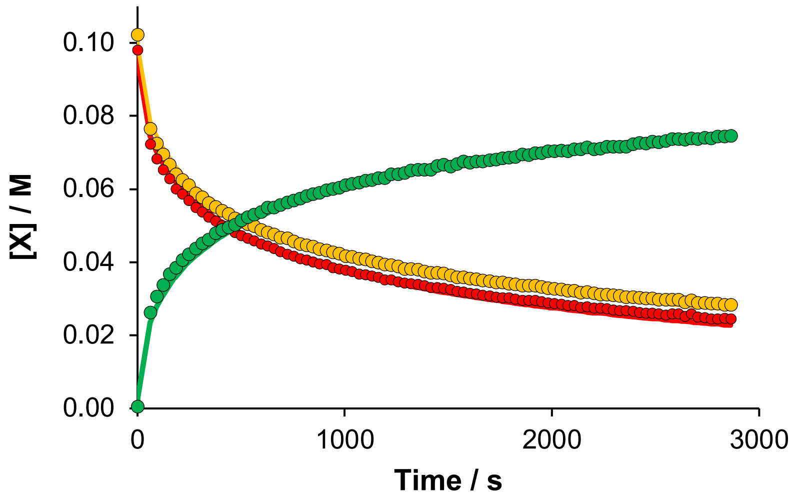

Figure S9: Experimental concentration-time profiles (points) for **1**, **2**, and **5** under regime I (*run 1:* [**1**]$_0$ = 0.10 M, [**2**]$_0$ = 0.10 M, [**3**]$_0$ = 0.10 M, [**4a$_H$**]$_0$ = 0.010 M; MeCN, 20 °C), and simulated profiles (lines) calculated from kinetic parameters obtained by global numerical fitting of all profiles – and those from the other 12 runs – to the telescoped kinetic model shown in Scheme S4. Profiles obtained by *in situ* ^{19}F NMR monitoring.

Global numerical fitting: Run 2

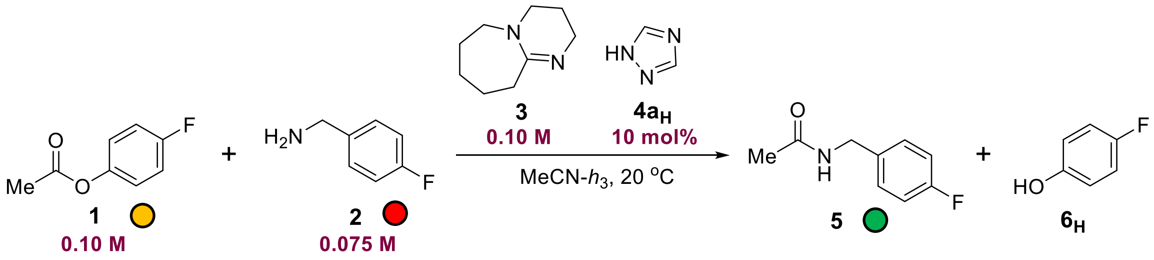

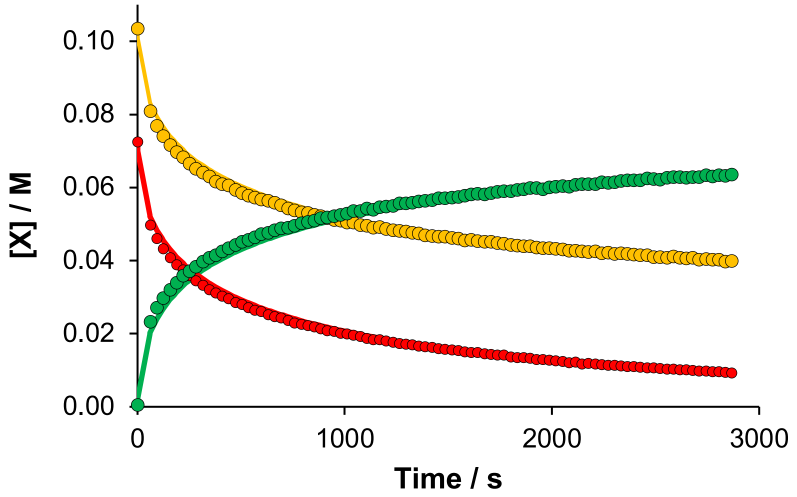

Figure S10: Experimental concentration-time profiles (points) for **1**, **2**, and **5** under regime I (*run 2:* [**1**]$_0$ = 0.10 M, [**2**]$_0$ = 0.075 M, [**3**]$_0$ = 0.10 M, [**4a$_H$**]$_0$ = 0.010 M; MeCN-h_3, 20 °C), and simulated profiles (lines) calculated from kinetic parameters obtained by global numerical fitting of all profiles – and those from the other 12 runs – to the telescoped kinetic model shown in Scheme S4. Profiles obtained by *in situ* ^{19}F NMR monitoring.

Global numerical fitting: Run 3

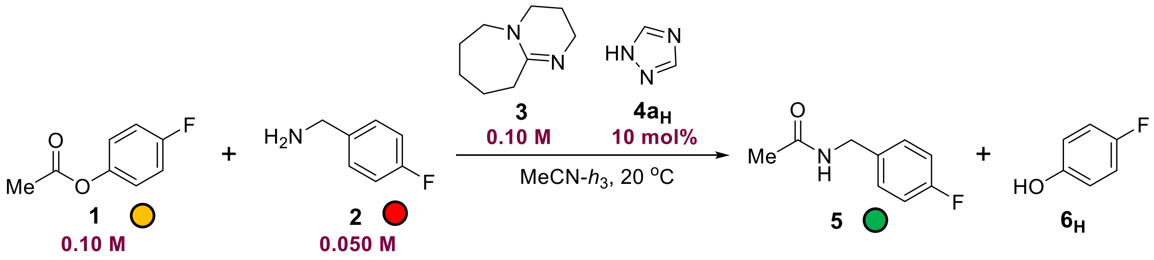

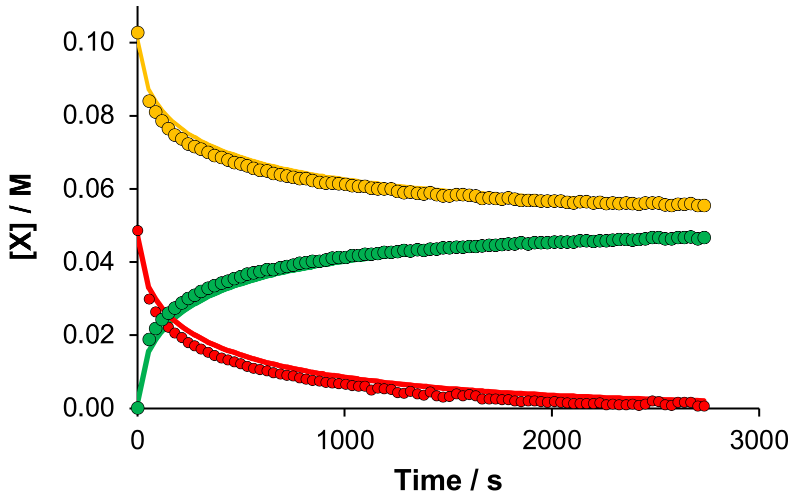

Figure S11: Experimental concentration-time profiles (points) for **1**, **2**, and **5** under regime I (*run 3:* [**1**]$_0$ = 0.10 M, [**2**]$_0$ = 0.050 M, [**3**]$_0$ = 0.10 M, [**4a$_H$**]$_0$ = 0.010 M; MeCN, 20 °C), and simulated profiles (lines) calculated from kinetic parameters obtained by global numerical fitting of all profiles – and those from the other 12 runs – to the telescoped kinetic model shown in Scheme S4. Profiles obtained by *in situ* ^{19}F NMR monitoring.

Global numerical fitting: Run 4

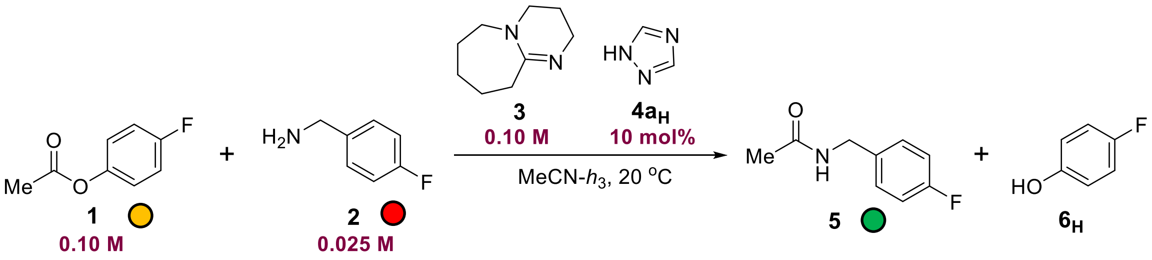

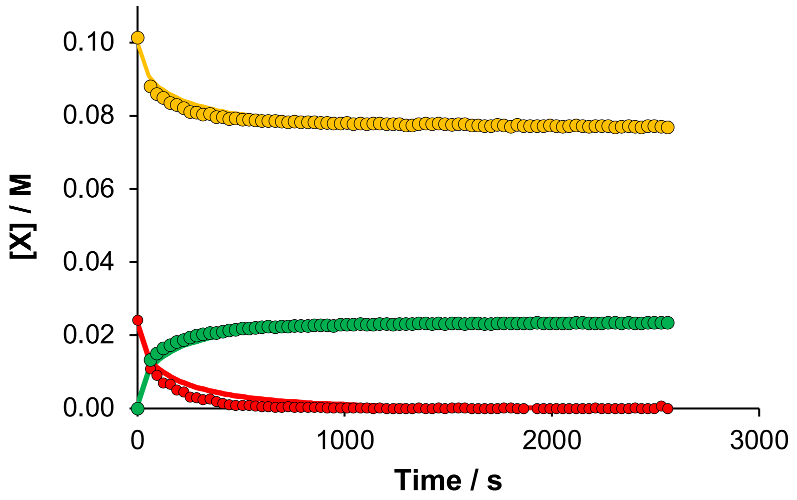

Figure S12: Experimental concentration-time profiles (points) for **1**, **2**, and **5** under regime I (*run 4:* [**1**]$_0$ = 0.10 M, [**2**]$_0$ = 0.025 M, [**3**]$_0$ = 0.10 M, [**4a$_H$**]$_0$ = 0.010 M; MeCN, 20 °C), and simulated profiles (lines) calculated from kinetic parameters obtained by global numerical fitting of all profiles – and those from the other 12 runs – to the telescoped kinetic model shown in Scheme S4. Profiles obtained by *in situ* ^{19}F NMR monitoring.

Global numerical fitting: Run 5

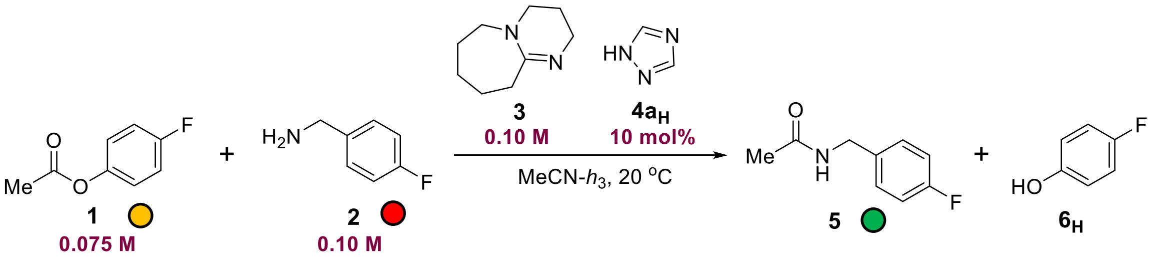

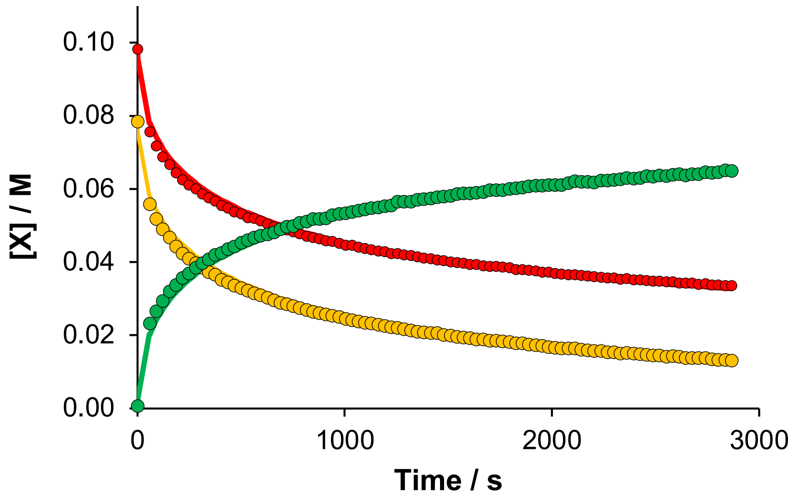

Figure S13: Experimental concentration-time profiles (points) for **1**, **2**, and **5** under regime I (*run 5:* [**1**]$_0$ = 0.075 M, [**2**]$_0$ = 0.10 M, [**3**]$_0$ = 0.10 M, [**4a$_H$**]$_0$ = 0.010 M; MeCN, 20 °C), and simulated profiles (lines) calculated from kinetic parameters obtained by global numerical fitting of all profiles – and those from the other 12 runs – to the telescoped kinetic model shown in Scheme S4. Profiles obtained by *in situ* ^{19}F NMR monitoring.

Global numerical fitting: Run 6

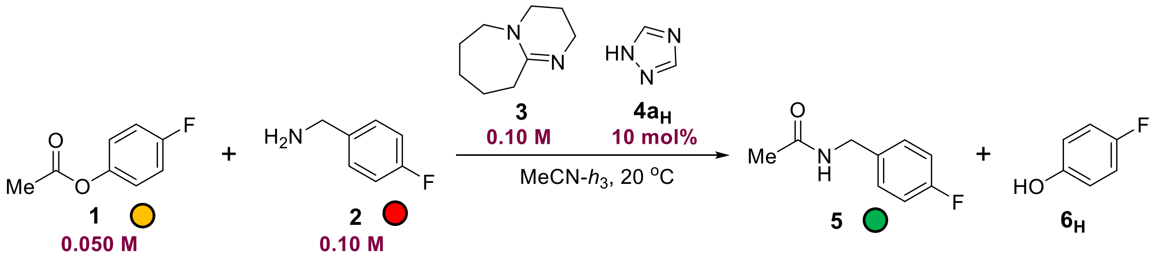

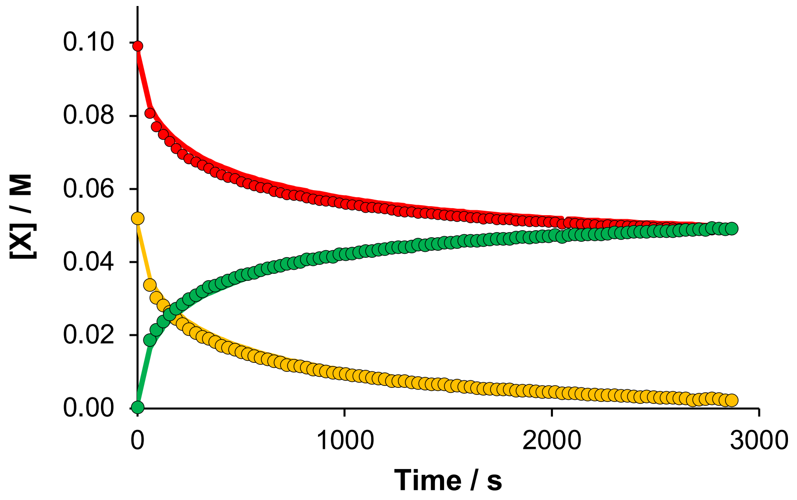

Figure S14: Experimental concentration-time profiles (points) for **1**, **2**, and **5** under regime I (*run 6:* [**1**]$_0$ = 0.050 M, [**2**]$_0$ = 0.10 M, [**3**]$_0$ = 0.10 M, [**4a$_H$**]$_0$ = 0.010 M; MeCN, 20 °C), and simulated profiles (lines) calculated from kinetic parameters obtained by global numerical fitting of all profiles – and those from the other 12 runs – to the telescoped kinetic model shown in Scheme S4. Profiles obtained by *in situ* ^{19}F NMR monitoring.

Global numerical fitting: Run 7

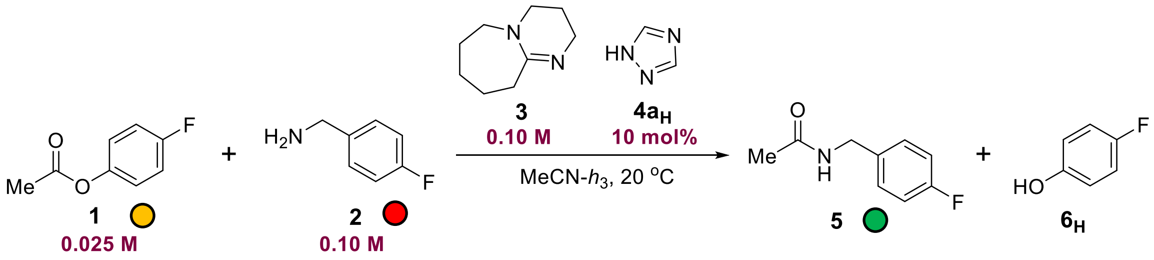

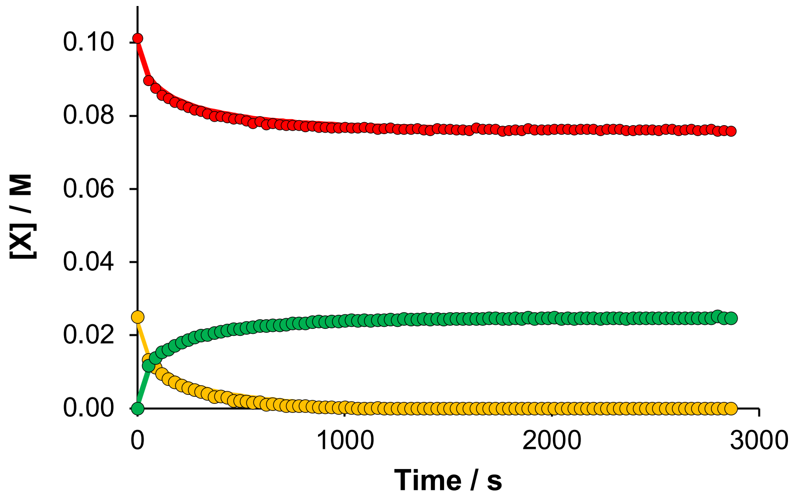

Figure S15: Experimental concentration-time profiles (points) for **1**, **2**, and **5** under regime I (*run 7:* [**1**]$_0$ = 0.025 M, [**2**]$_0$ = 0.10 M, [**3**]$_0$ = 0.10 M, [**4a$_H$**]$_0$ = 0.010 M; MeCN, 20 °C), and simulated profiles (lines) calculated from kinetic parameters obtained by global numerical fitting of all profiles – and those from the other 12 runs – to the telescoped kinetic model shown in Scheme S4. Profiles obtained by *in situ* ^{19}F NMR monitoring.

Global numerical fitting: Run 8

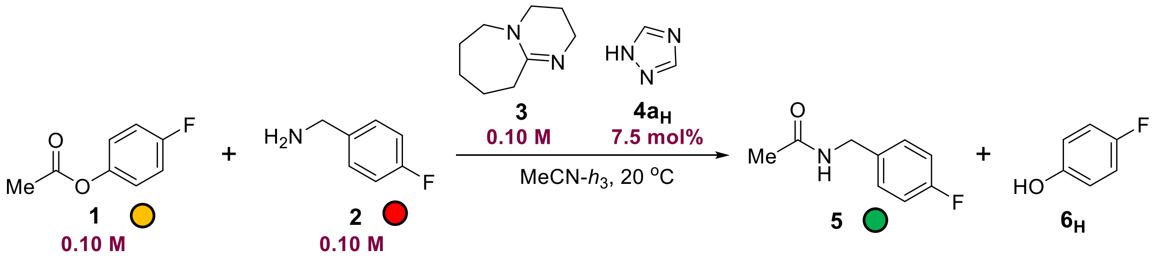

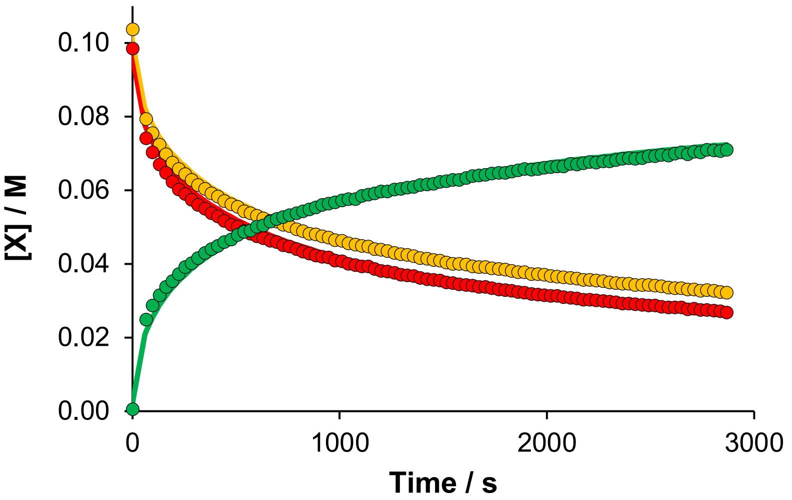

Figure S16: Experimental concentration-time profiles (points) for **1**, **2**, and **5** under regime I (*run 8:* [**1**]$_0$ = 0.10 M, [**2**]$_0$ = 0.10 M, [**3**]$_0$ = 0.10 M, [**4a$_H$**]$_0$ = 0.0075 M; MeCN, 20 °C), and simulated profiles (lines) calculated from kinetic parameters obtained by global numerical fitting of all profiles – and those from the other 12 runs – to the telescoped kinetic model shown in Scheme S4. Profiles obtained by *in situ* ^{19}F NMR monitoring.

Global numerical fitting: Run 9

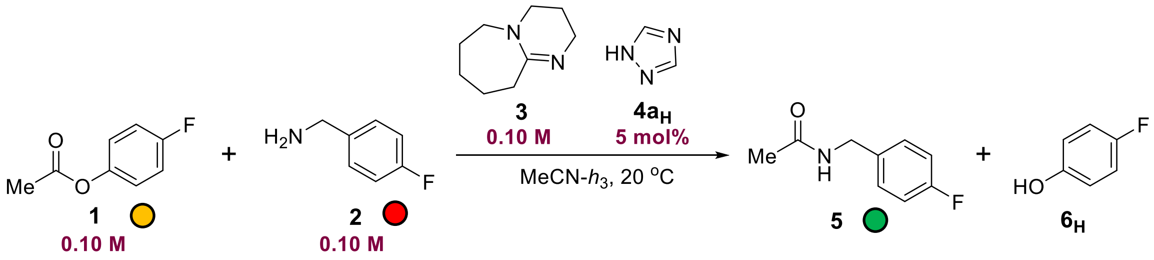

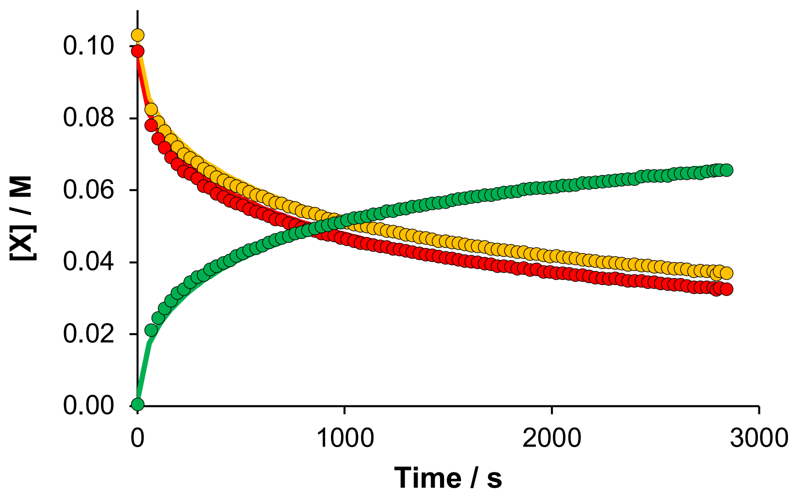

Figure S17: Experimental concentration-time profiles (points) for **1**, **2**, and **5** under regime I (*run 9:* [**1**]$_0$ = 0.10 M, [**2**]$_0$ = 0.10 M, [**3**]$_0$ = 0.10 M, [**4a$_H$**]$_0$ = 0.0050 M; MeCN, 20 °C), and simulated profiles (lines) calculated from kinetic parameters obtained by global numerical fitting of all profiles – and those from the other 12 runs – to the telescoped kinetic model shown in Scheme S4. Profiles obtained by *in situ* ^{19}F NMR monitoring.

Global numerical fitting: Run 10

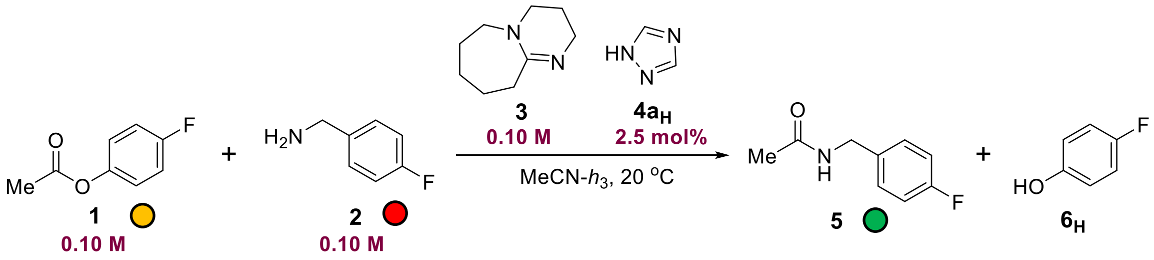

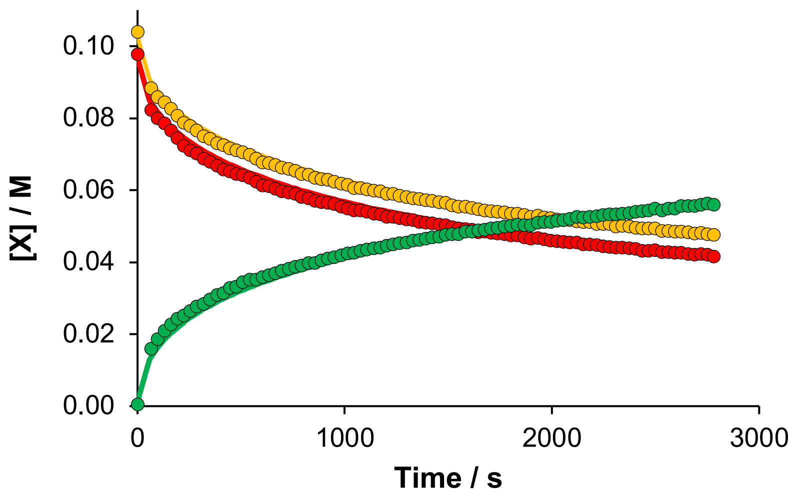

Figure S18: Experimental concentration-time profiles (points) for **1**, **2**, and **5** under regime I (*run 10:* [**1**]$_0$ = 0.10 M, [**2**]$_0$ = 0.10 M, [**3**]$_0$ = 0.10 M, [**4a$_H$**]$_0$ = 0.0025 M; MeCN, 20 °C), and simulated profiles (lines) calculated from kinetic parameters obtained by global numerical fitting of all profiles – and those from the other 12 runs – to the telescoped kinetic model shown in Scheme S4. Profiles obtained by *in situ* ^{19}F NMR monitoring.

Global numerical fitting: Run 11

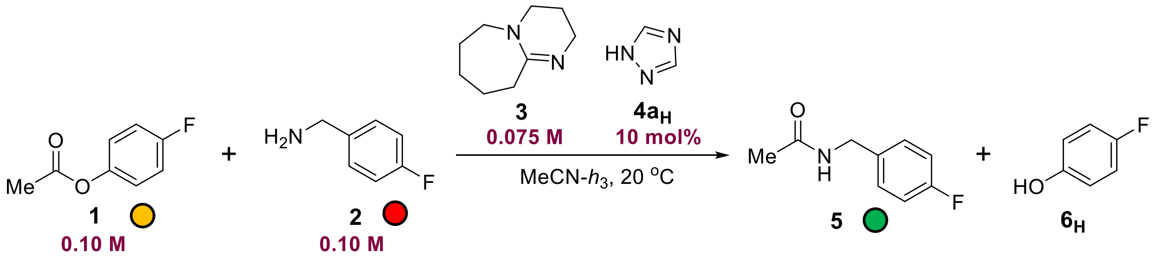

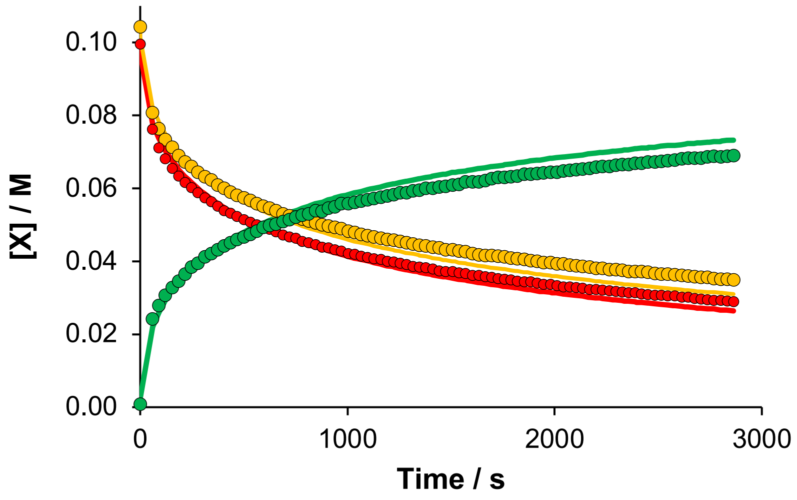

Figure S19: Experimental concentration-time profiles (points) for **1**, **2**, and **5** under regime I (*run 11:* [**1**]$_0$ = 0.10 M, [**2**]$_0$ = 0.10 M, [**3**]$_0$ = 0.075 M, [**4a$_H$**]$_0$ = 0.010 M; MeCN, 20 °C), and simulated profiles (lines) calculated from kinetic parameters obtained by global numerical fitting of all profiles – and those from the other 12 runs – to the telescoped kinetic model shown in Scheme S4. Profiles obtained by *in situ* ^{19}F NMR monitoring.

Global numerical fitting: Run 12

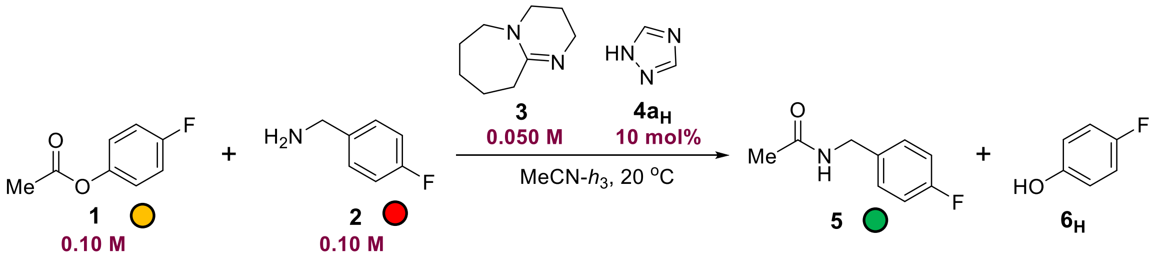

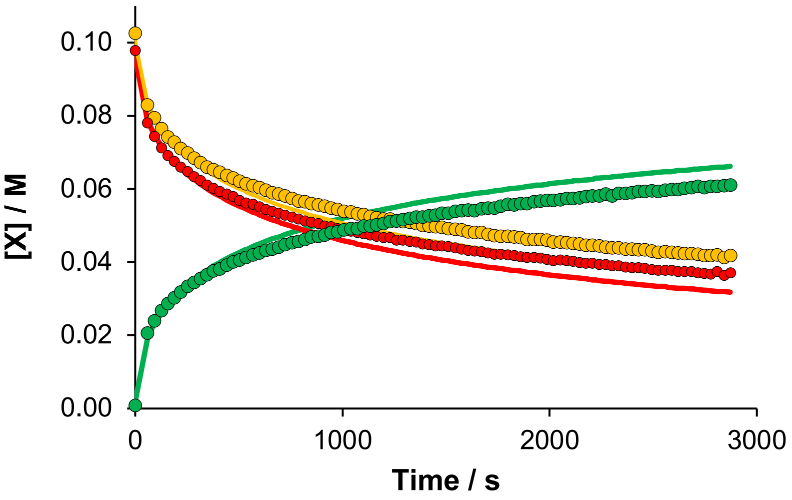

Figure S20: Experimental concentration-time profiles (points) for **1**, **2**, and **5** under regime I (*run 12:* [**1**]$_0$ = 0.10 M, [**2**]$_0$ = 0.10 M, [**3**]$_0$ = 0.050 M, [**4a$_H$**]$_0$ = 0.010 M; MeCN, 20 °C), and simulated profiles (lines) calculated from kinetic parameters obtained by global numerical fitting of all profiles – and those from the other 12 runs – to the telescoped kinetic model shown in Scheme S4. Profiles obtained by *in situ* ^{19}F NMR monitoring.

Global numerical fitting: Run 13

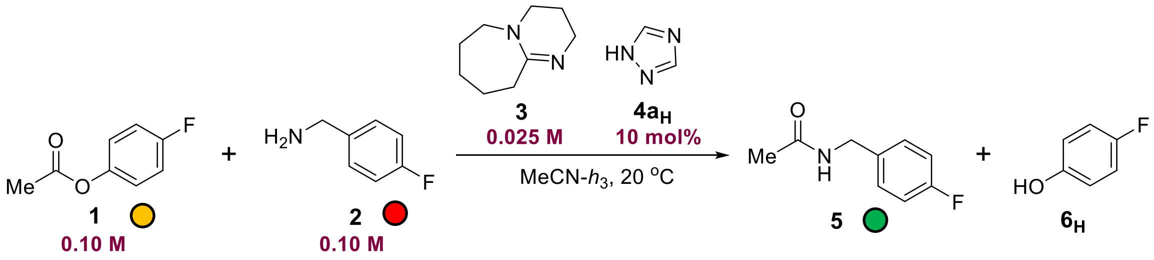

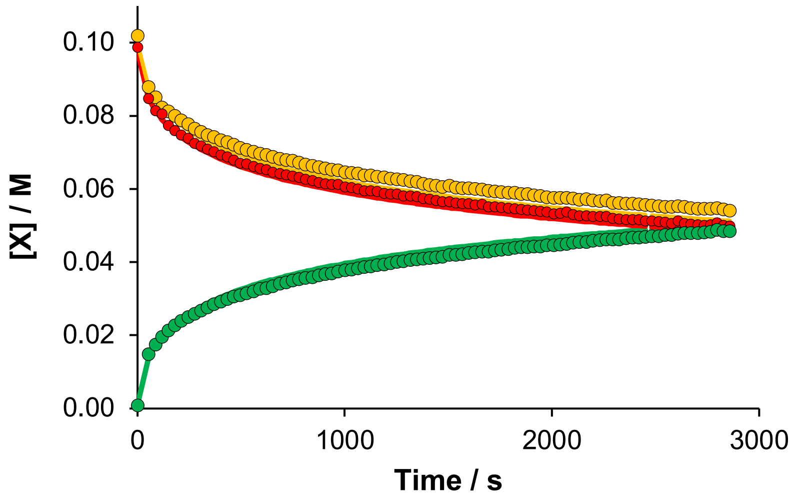

Figure S21: Experimental concentration-time profiles (points) for **1**, **2**, and **5** under regime I (*run 13:* [**1**]$_0$ = 0.10 M, [**2**]$_0$ = 0.10 M, [**3**]$_0$ = 0.025 M, [**4a$_H$**]$_0$ = 0.010 M; MeCN, 20 °C), and simulated profiles (lines) calculated from kinetic parameters obtained by global numerical fitting of all profiles – and those from the other 12 runs – to the telescoped kinetic model shown in Scheme S4. Profiles obtained by *in situ* ^{19}F NMR monitoring.

S3.8.3 Regime II Kinetics

Raw product evolution profiles

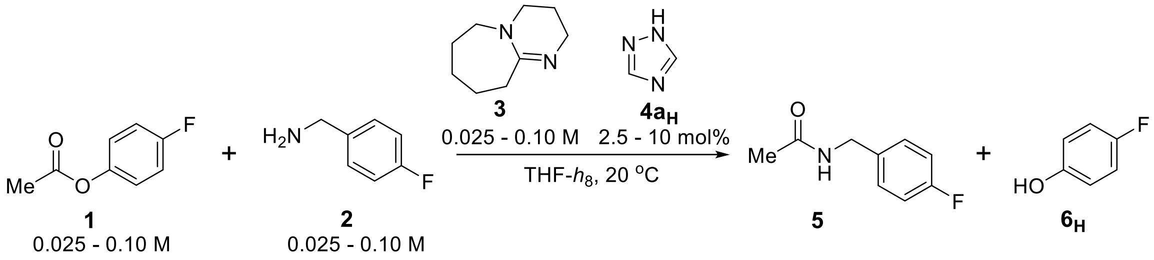

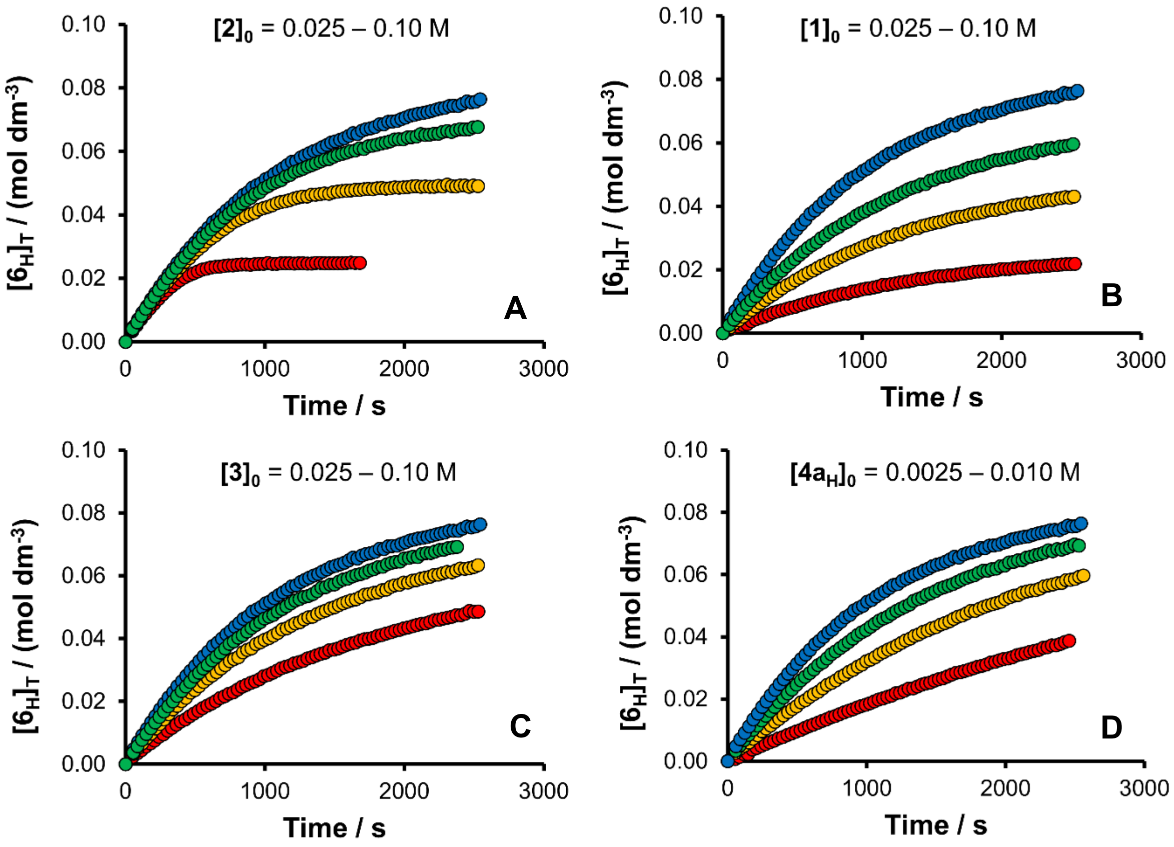

Figure S22: Product evolution profiles for the aminolysis of *p*-F-PhAc **1** with *p*-F-BnNH$_2$ **2** in THF at 20 °C, obtained by *in situ* ^{19}F NMR spectroscopy. Product evolution quantified by the *total* concentration of liberated *p*-F-PhOH, **[6$_H$]$_T$,** in each kinetic run. (A) Aminolysis of **1** (0.10 M) with variable **2** (0.025 – 0.10 M), DBU **3** (0.10 M) and 1,2,4-triazole **4a$_H$**(10 mol%); (B) Aminolysis of variable **1** (0.025 - 0.10 M) with **2** (0.10 M), **3** (0.10 M) and **4a$_H$** (10 mol%); (C) Aminolysis of **1** (0.10 M) with **2** (0.10 M), variable **3** (0.025 - 0.10 M) and **4a$_H$** (10 mol%); (D) Aminolysis of **1** (0.10 M) with **2** (0.10 M), **3** (0.10 M) and variable **4a$_H$** (2.5 - 10 mol%).

Normalized product evolution profiles

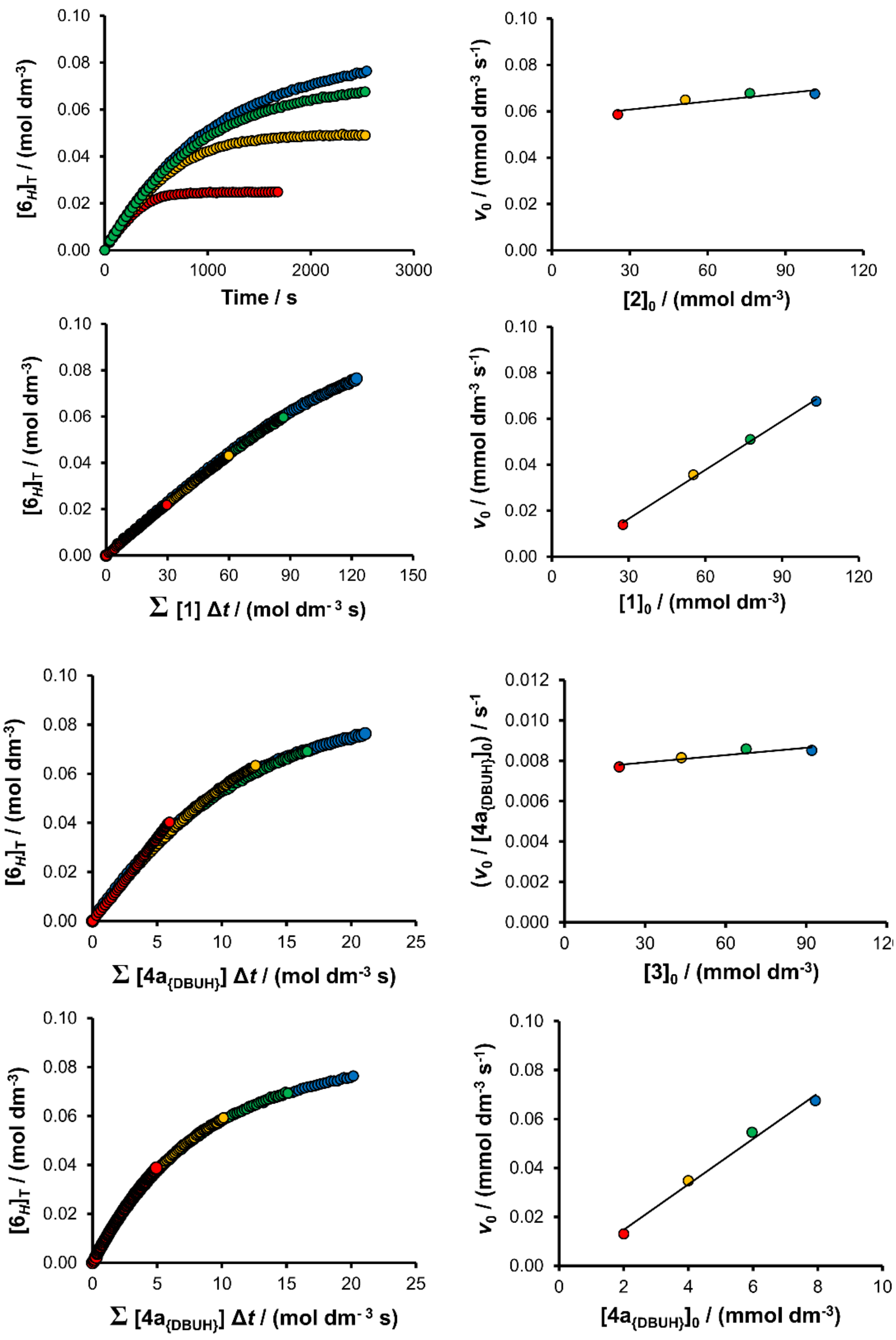

Figure S23: Single-component normalised product evolution profiles and initial rate analysis for each of the four components involved in the aminolysis of *p*-F-PhAc **1** with *p*-F-BnNH$_2$ **2**, DBU **3** and 1,2,4-triazole **4a$_H$** in THF at 20 °C (regime II; raw kinetics: Figure S22). Normalisation conducted with respect to 1,2,4-triazolate **4a$_{\{DBUH\}}$** and *un-ionised* DBU **3**, the concentrations of which ([**4a$_{\{DBUH\}}$**], [**3**]) were determined at each time point by numerical simulation. Numerical simulations used values of $K_{1:1}$(**4a$_H$**) = 41 M^{-1} for **4a$_H$** + **3** ↔ **4a$_{\{DBUH\}}$** and $K_{1:1}$(**6$_H$**) = 237 M^{-1} for **6$_H$** + **3** ↔ **6$_{\{DBUH\}}$**, as determined by ^{1}H and ^{19}F NMR titrations in THF-d_8/h_8 at [**4a$_H$**]$_T$ = 0.050 M and [**6$_H$**]$_T$ = 0.050 M, respectively.

*Initial rate analysis for variable [**3**]₀*

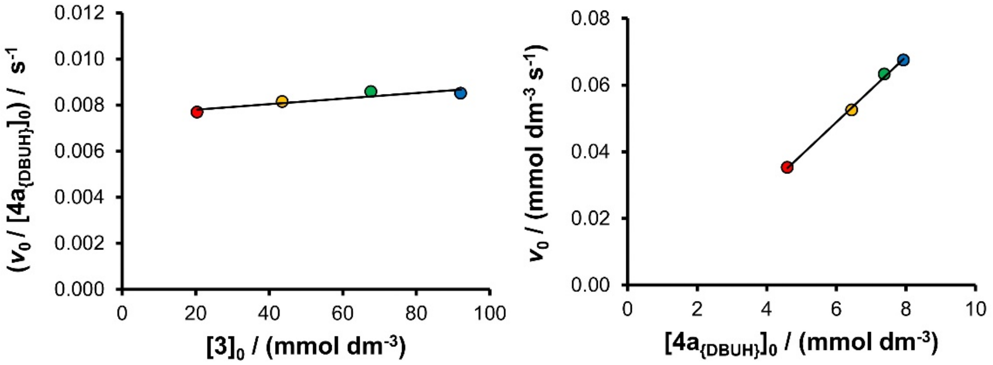

Figure S24: Initial rate analysis for the aminolysis of *p*-F-PhAc **1** (0.10 M) with *p*-F-BnNH$_2$ **2** (0.10 M), variable **3** DBU (0.025 - 0.10 M) and 1,2,4-triazole **4a$_H$** (10 mol%) in THF at 20 °C (from Figure S22). Initial concentrations of un-ionised DBU **3** ([**3**]$_0$) and 1,2,4-triazolate (**4a$_{\{DBUH\}}$**) were determined analytically on the basis of an equilibrium constant $K_{1:1}$(**4a$_H$**) = 41 M^{-1} for **4a$_H$** + **3** ↔ **4a$_{\{DBUH\}}$**, as determined by ^{1}H NMR titration in THF-d_8 at [**4a$_H$**]$_T$ = 0.050 M.

S3.8.4 Regime III Kinetics

Raw product evolution profiles

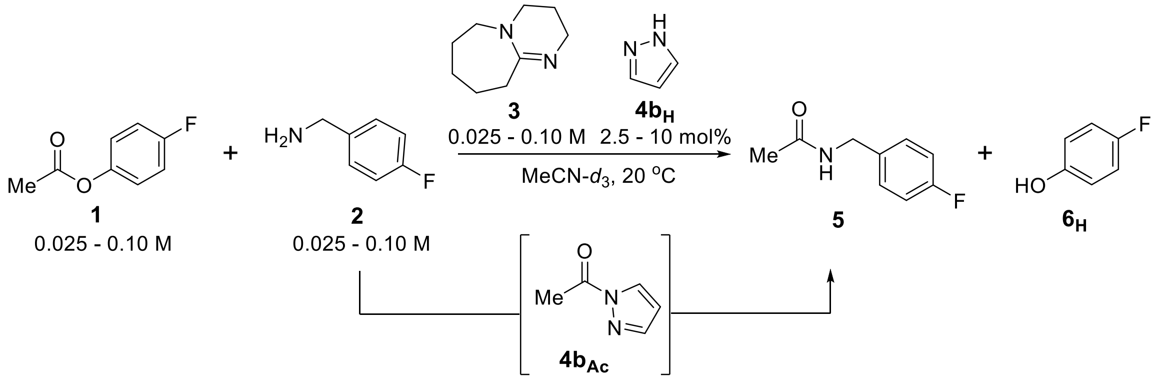

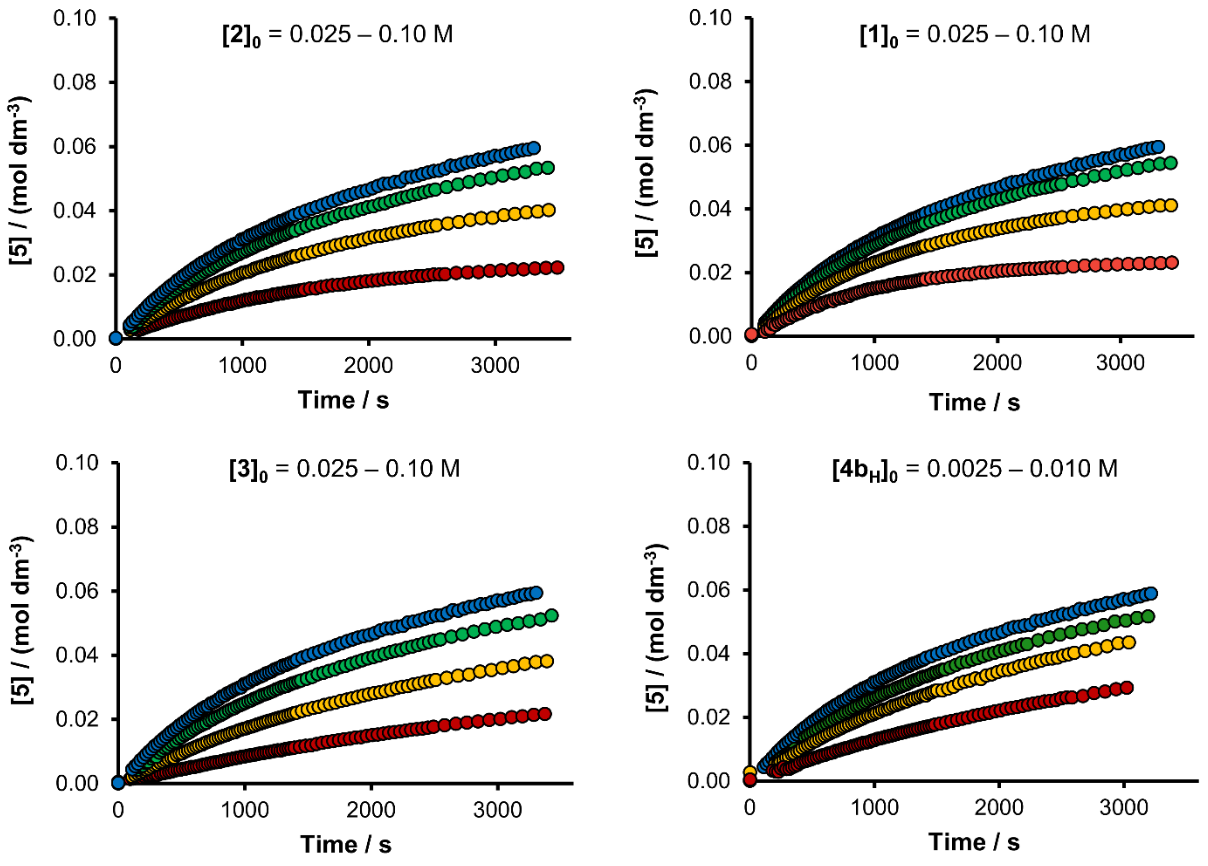

Figure S25: Product evolution profiles for the aminolysis of *p*-F-PhAc **1** with *p*-F-BnNH$_2$ **2**, DBU **3** and pyrazole **4b$_H$** in MeCN-*d*$_3$ at 20 °C, obtained by *in situ* ^{1}H NMR spectroscopy. Product evolution quantified by the total concentration of liberated *p*-F-BnNHAc **5** in each kinetic run. (A) Aminolysis of **1** (0.10 M) with variable **2** (0.025 – 0.10 M), **3** (0.10 M) and **4b$_H$** (10 mol%); (B) Aminolysis of variable **1** (0.025 - 0.10 M) with **2** (0.10 M), **3** (0.10 M) and **4b$_H$** (10 mol%); (C) Aminolysis of **1** (0.10 M) with **2** (0.10 M), variable **3** (0.025 - 0.10 M) and **4b$_H$** (10 mol%); (C) Aminolysis of **1** (0.10 M) with **2** (0.10 M), **3** (0.10 M) and variable **4b$_H$** (2.5 - 10 mol%).

Catalyst speciation profiles

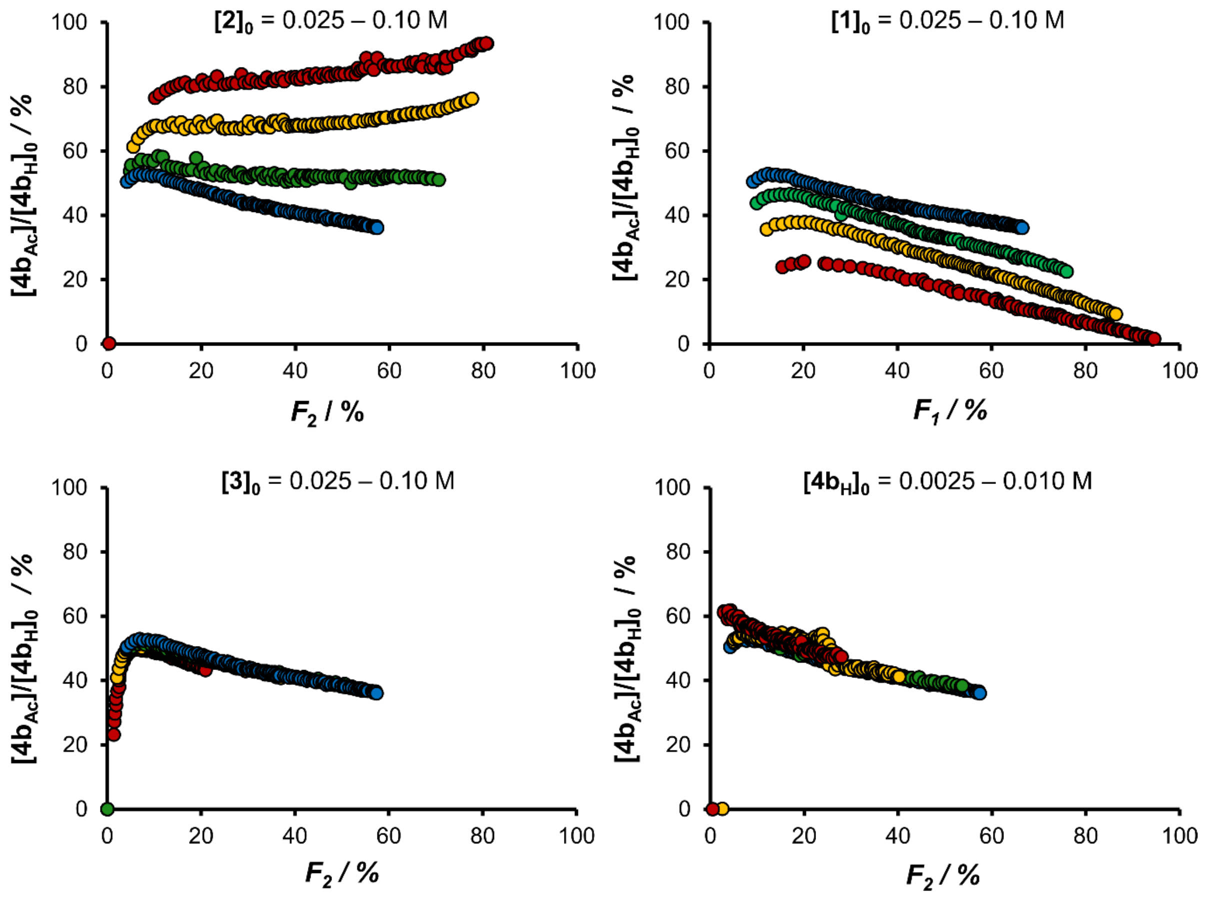

Figure S26: Catalyst speciation ([**4b**$_{Ac}$]/[**4b**$_{H}$]$_0$) profiles for the aminolysis of *p*-F-PhAc **1** with *p*-F-BnNH$_2$ **2**, DBU **3** and pyrazole **4b**$_{H}$ in MeCN-d_3 at 20 °C, obtained by *in situ* ^{1}H NMR spectroscopy; conversion pertains to limiting substrate in each case (F_1 or F_2). (A) Aminolysis of **1** (0.10 M) with variable **2** (0.025 – 0.10 M), **3** (0.10 M) and **4b**$_{H}$ (10 mol%); (B) Aminolysis of variable **1** (0.025 - 0.10 M) with **2** (0.10 M), **3** (0.10 M) and **4b**$_{H}$ (10 mol%); (C) Aminolysis of **1** (0.10 M) with **2** (0.10 M), variable **3** (0.025 - 0.10 M) and **4b**$_{H}$ (10 mol%); (D) Aminolysis of **1** (0.10 M) with **2** (0.10 M), **3** (0.10 M) and variable **4b**$_{H}$ (2.5 - 10 mol%).

For the global fitting parameters used in Runs 1 to 15 below, see Table S7.

Global numerical fitting: Run 1

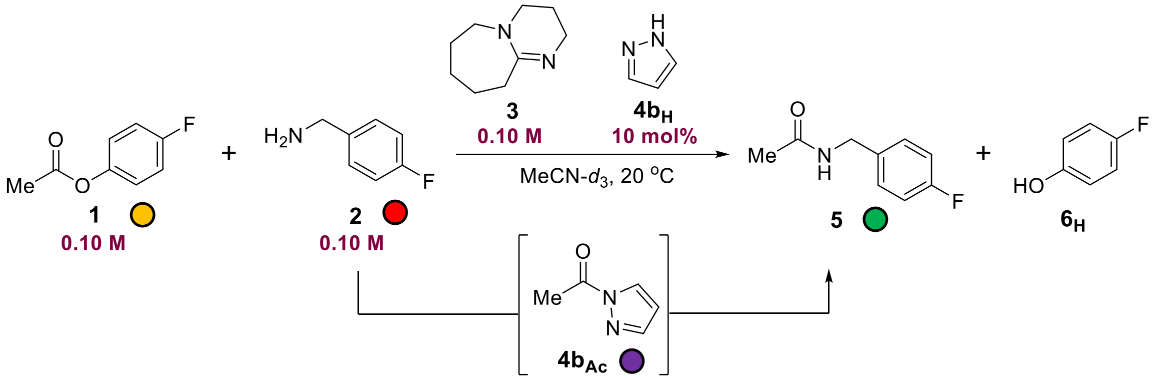

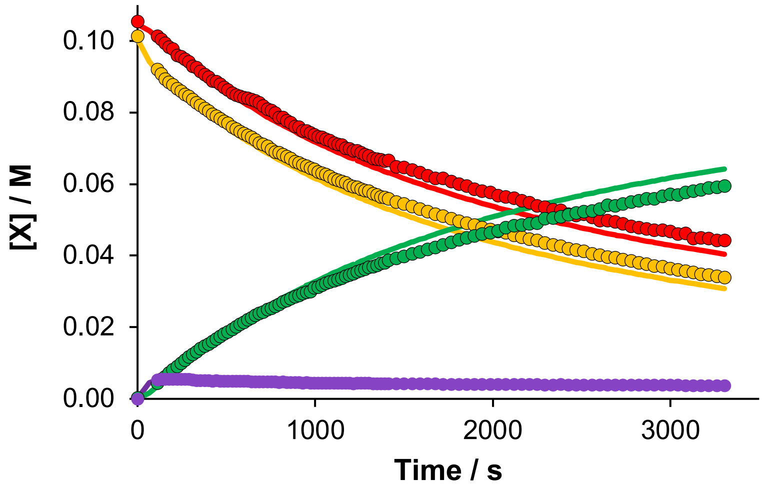

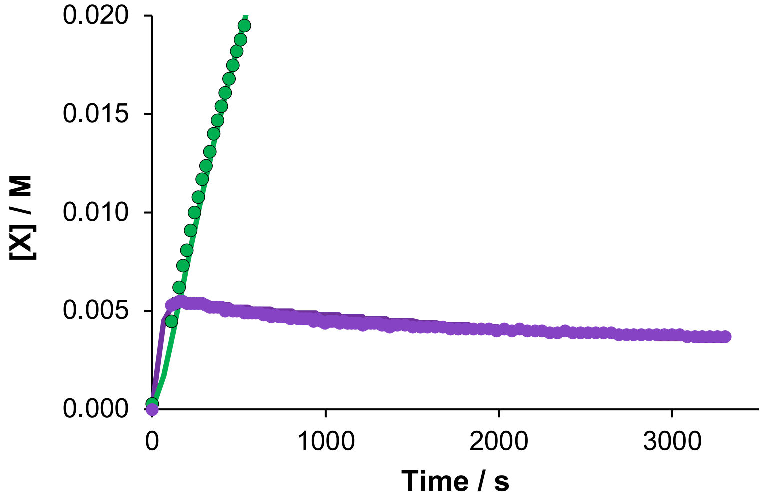

Figure S27: Experimental concentration-time profiles (points) for **1**, **2**, **5** and **4b$_{Ac}$** under regime III (*run 1*: [**1**]$_0$ = 0.10 M, [**2**]$_0$ = 0.10 M, [**3**]$_0$ = 0.10 M, [**4b$_H$**]$_0$ = 0.010 M; MeCN-d_3, 20 °C), and simulated profiles (lines) calculated from kinetic parameters obtained by global numerical fitting of all profiles – and those from the other 14 runs – to the telescoped kinetic model shown in Figure 6 and Scheme S3. Profiles obtained by *in situ* ^{1}H NMR monitoring.

Global numerical fitting: Run 2

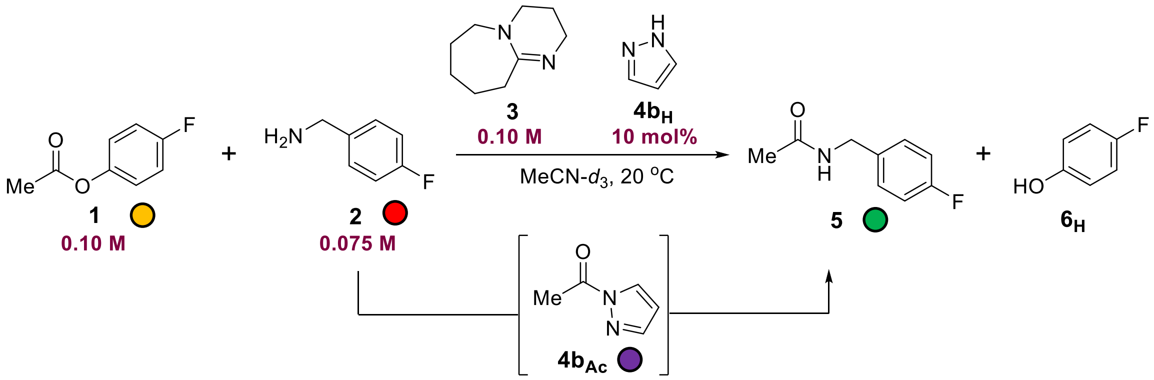

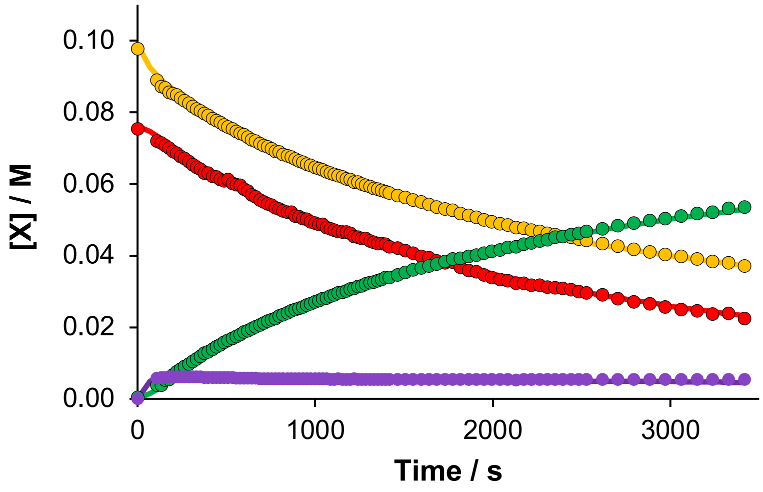

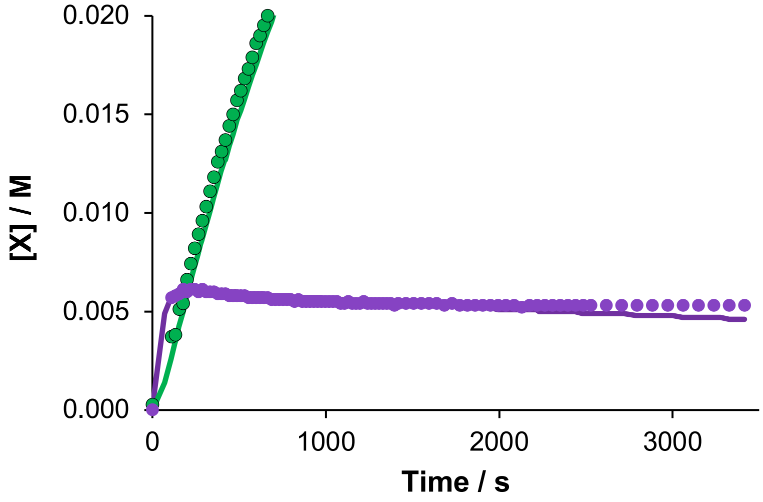

Figure S28: Experimental concentration-time profiles (points) for **1**, **2**, **5** and **4b$_{Ac}$** under regime III (*run 2*: [**1**]$_0$ = 0.10 M, [**2**]$_0$ = 0.075 M, [**3**]$_0$ = 0.10 M, [**4b$_H$**]$_0$ = 0.010 M; MeCN-d_3, 20 °C), and simulated profiles (lines) calculated from kinetic parameters obtained by global numerical fitting of all profiles – and those from the other 14 runs – to the telescoped kinetic model shown in Figure 6 and Scheme S3. Profiles obtained by *in situ* ^{1}H NMR monitoring.

Global numerical fitting: Run 3

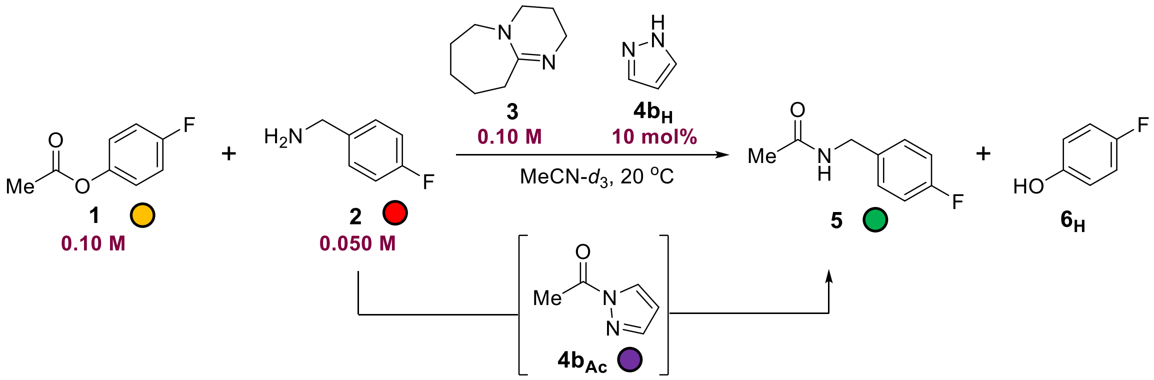

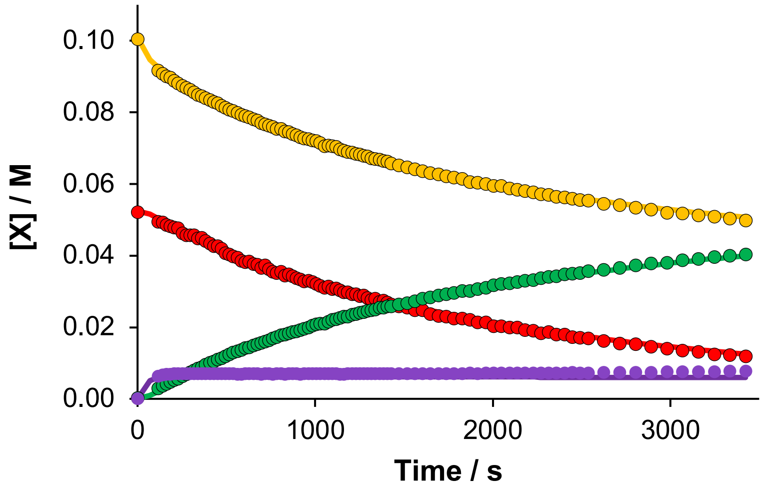

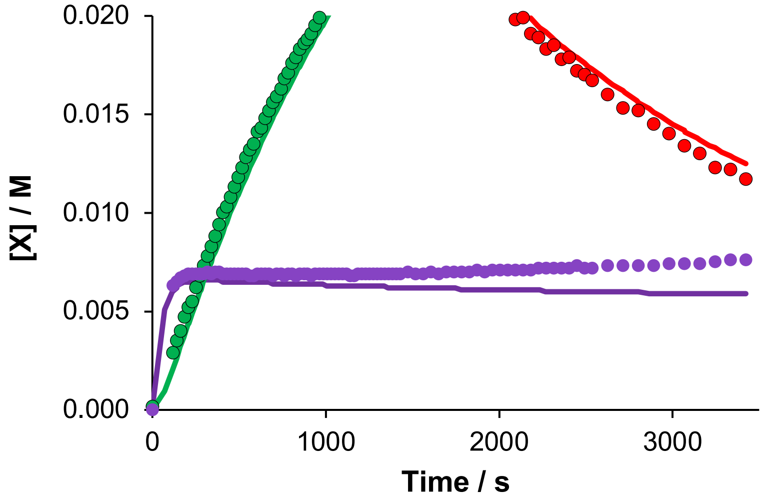

Figure S29: Experimental concentration-time profiles (points) for **1**, **2**, **5** and **4b$_{Ac}$** under regime III (*run 3*: [**1**]$_0$ = 0.10 M, [**2**]$_0$ = 0.050 M, [**3**]$_0$ = 0.10 M, [**4b$_H$**]$_0$ = 0.010 M; MeCN-d_3, 20 °C), and simulated profiles (lines) calculated from kinetic parameters obtained by global numerical fitting of all profiles – and those from the other 14 runs – to the telescoped kinetic model shown in Figure 6 and Scheme S3. Profiles obtained by *in situ* ^{1}H NMR monitoring.

Global numerical fitting: Run 4

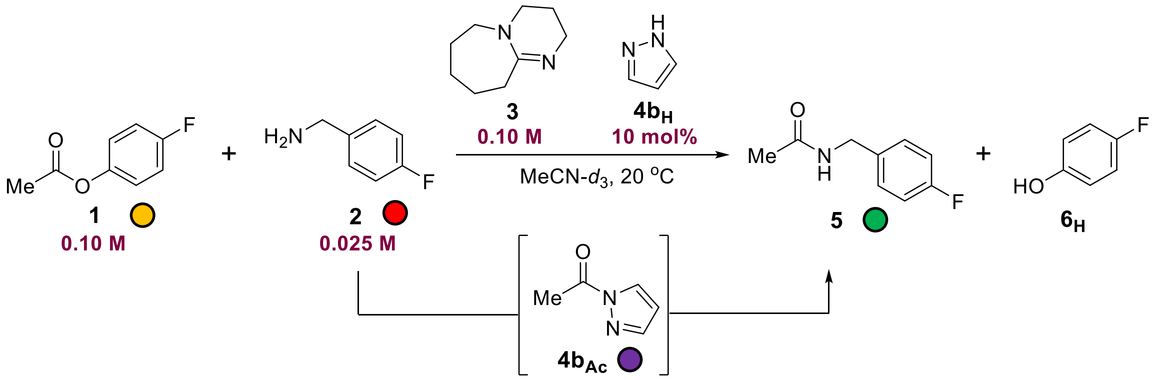

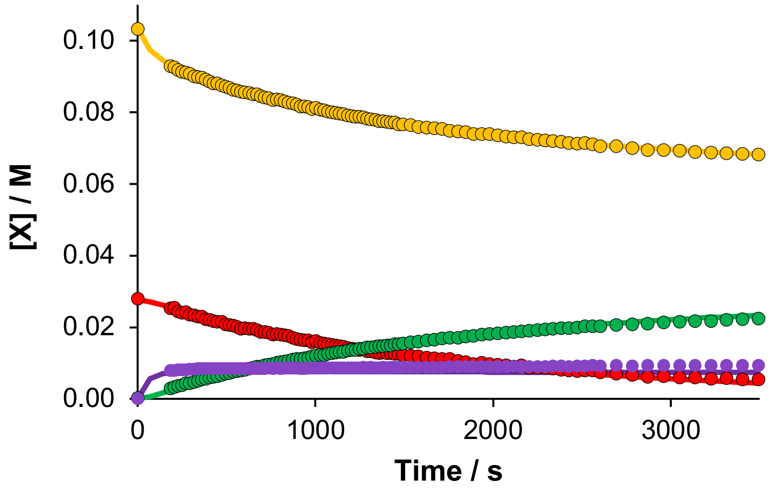

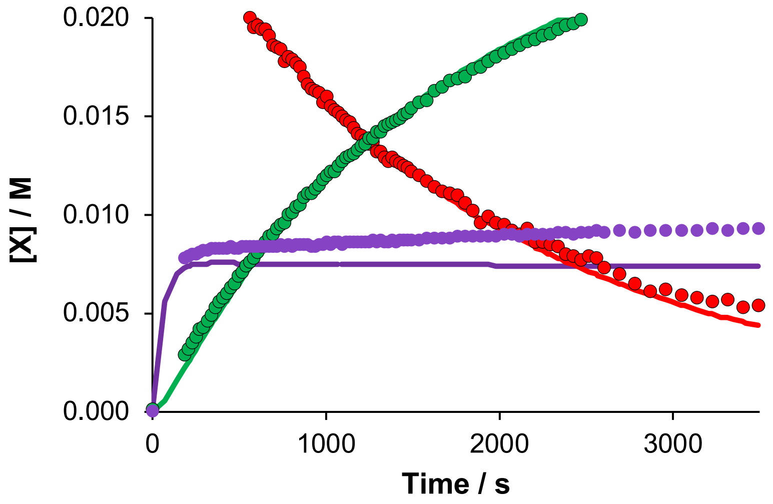

Figure S30: Experimental concentration-time profiles (points) for **1**, **2**, **5** and **4b$_{Ac}$** under regime III (*run 4*: [**1**]$_0$ = 0.10 M, [**2**]$_0$ = 0.025 M, [**3**]$_0$ = 0.10 M, [**4b$_H$**]$_0$ = 0.010 M; MeCN-d_3, 20 °C), and simulated profiles (lines) calculated from kinetic parameters obtained by global numerical fitting of all profiles – and those from the other 14 runs – to the telescoped kinetic model shown in Figure 6 and Scheme S3. Profiles obtained by *in situ* ^{1}H NMR monitoring.

Global numerical fitting: Run 5

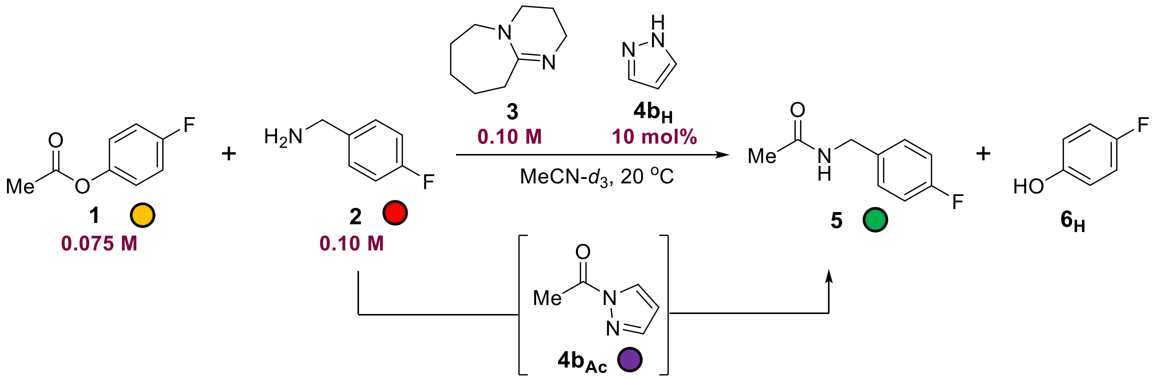

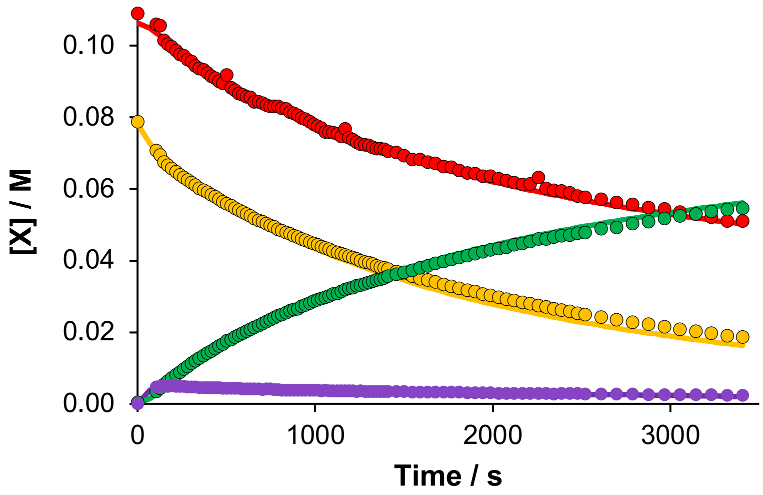

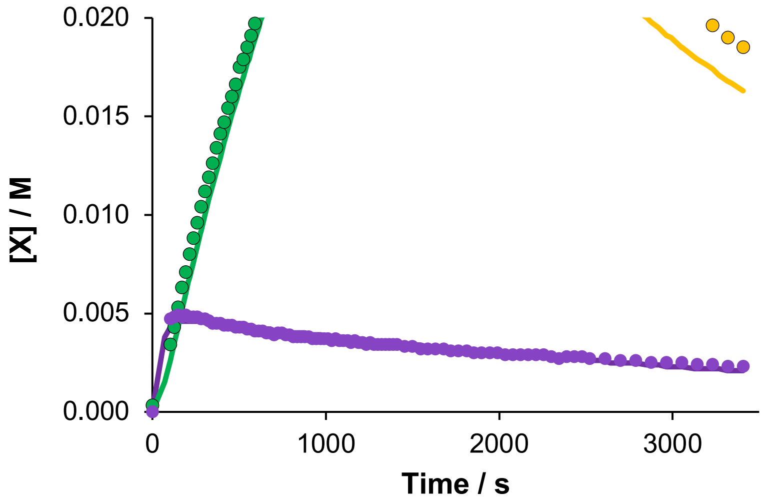

Figure S31: Experimental concentration-time profiles (points) for **1**, **2**, **5** and **4b$_{Ac}$** under regime III (*run 5*: [**1**]$_0$ = 0.075 M, [**2**]$_0$ = 0.10 M, [**3**]$_0$ = 0.10 M, [**4b$_H$**]$_0$ = 0.010 M; MeCN-d_3, 20 °C), and simulated profiles (lines) calculated from kinetic parameters obtained by global numerical fitting of all profiles – and those from the other 14 runs – to the telescoped kinetic model shown in Figure 6 and Scheme S3. Profiles obtained by *in situ* ^{1}H NMR monitoring.

Global numerical fitting: Run 6

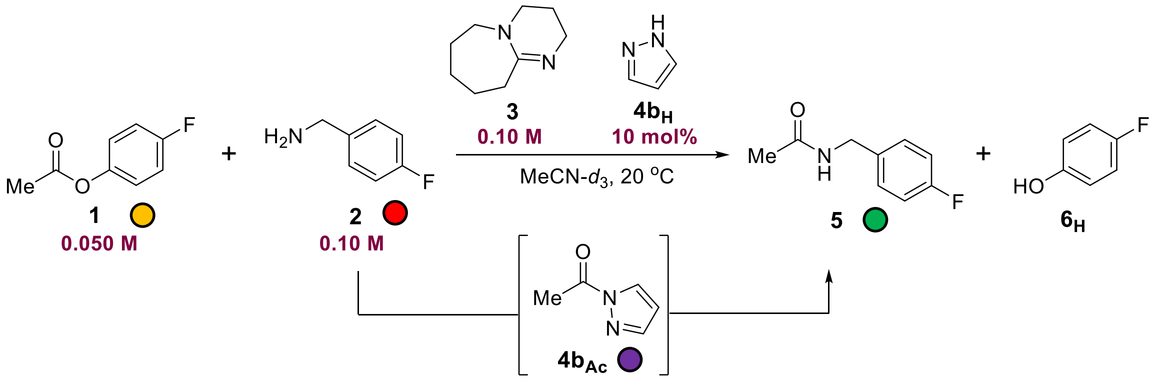

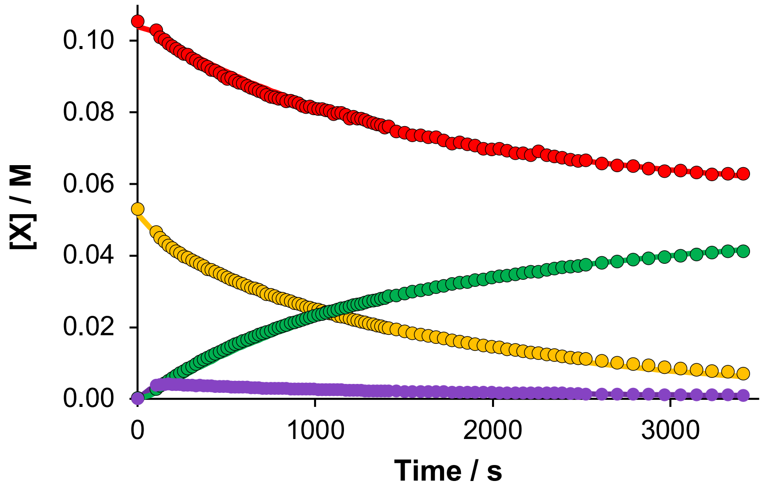

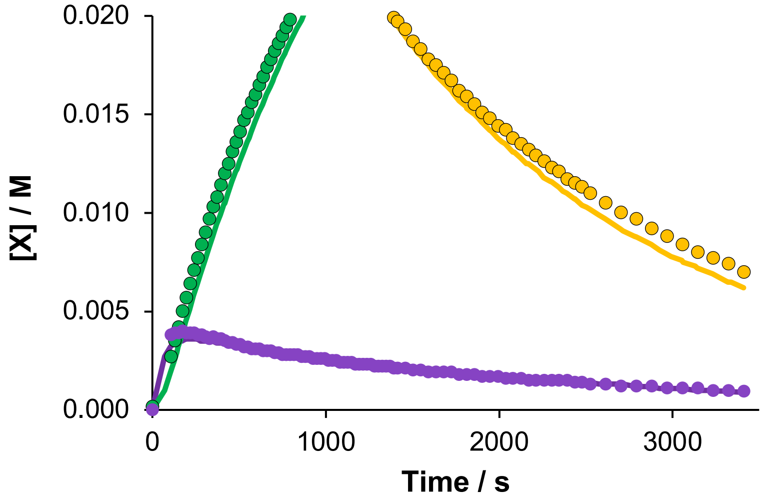

Figure S32: Experimental concentration-time profiles (points) for **1**, **2**, **5** and **4b$_{Ac}$** under regime III (*run 6*: [**1**]$_0$ = 0.050 M, [**2**]$_0$ = 0.10 M, [**3**]$_0$ = 0.10 M, [**4b$_H$**]$_0$ = 0.010 M; MeCN-d_3, 20 °C), and simulated profiles (lines) calculated from kinetic parameters obtained by global numerical fitting of all profiles – and those from the other 14 runs – to the telescoped kinetic model shown in Figure 6 and Scheme S3. Profiles obtained by *in situ* ^{1}H NMR monitoring.

Global numerical fitting: Run 7

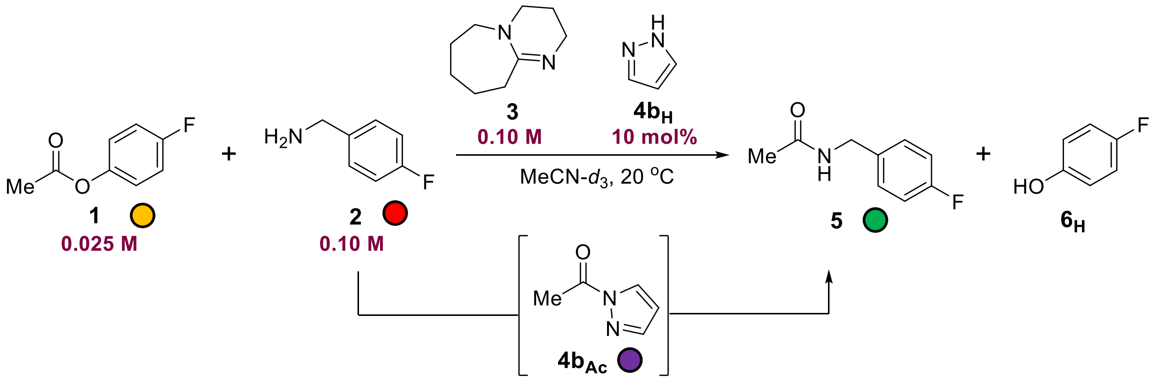

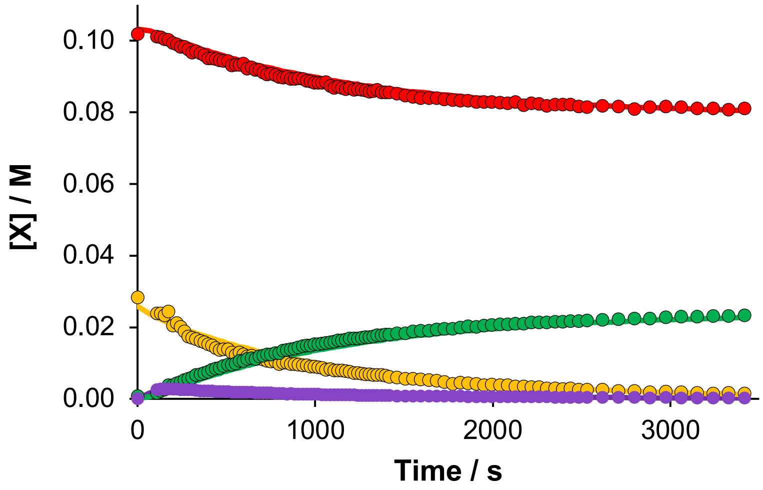

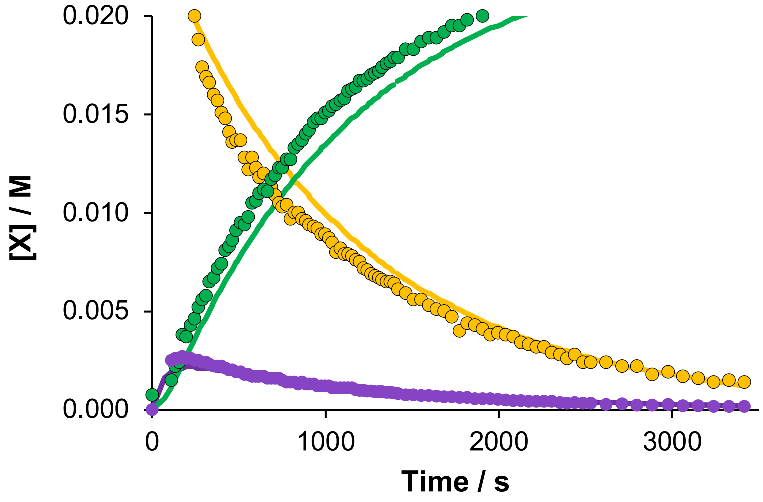

Figure S33: Experimental concentration-time profiles (points) for **1**, **2**, **5** and **4b$_{Ac}$** under regime III (*run 7*: [**1**]$_0$ = 0.025 M, [**2**]$_0$ = 0.10 M, [**3**]$_0$ = 0.10 M, [**4b$_H$**]$_0$ = 0.010 M; MeCN-d_3, 20 °C), and simulated profiles (lines) calculated from kinetic parameters obtained by global numerical fitting of all profiles – and those from the other 14 runs – to the telescoped kinetic model shown in Figure 6 and Scheme S3. Profiles obtained by *in situ* ^{1}H NMR monitoring.

Global numerical fitting: Run 8

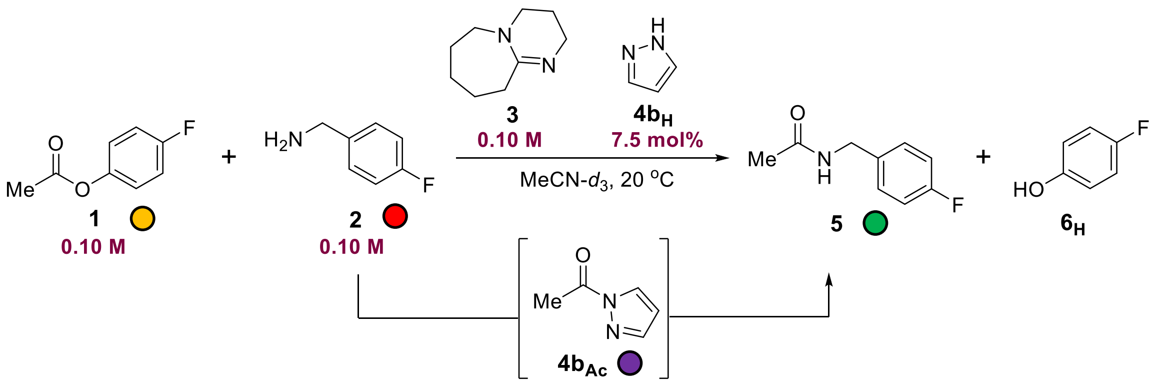

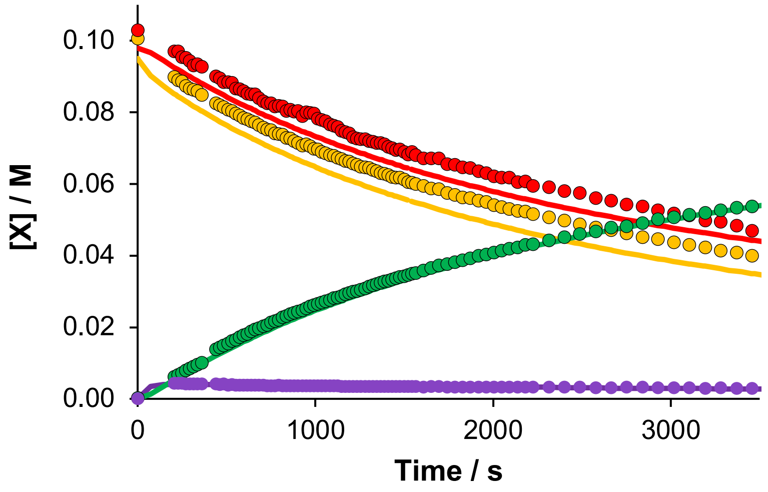

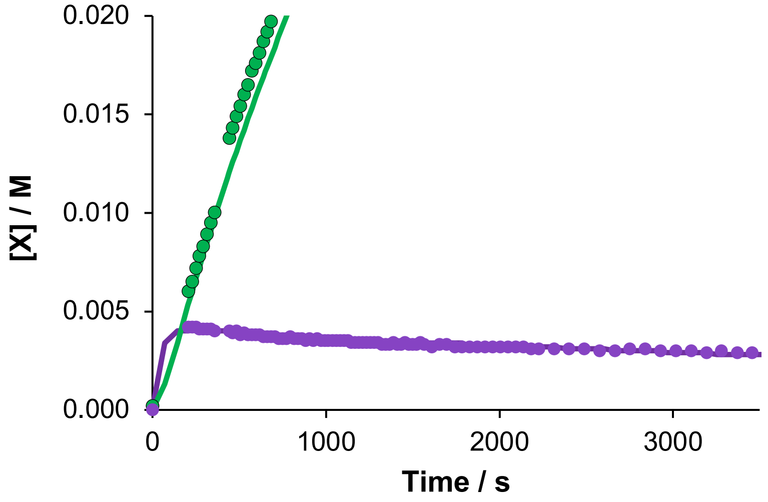

Figure S34: Experimental concentration-time profiles (points) for **1**, **2**, **5** and **4b$_{Ac}$** under regime III (*run 8*: [**1**]$_0$ = 0.10 M, [**2**]$_0$ = 0.10 M, [**3**]$_0$ = 0.10 M, [**4b$_H$**]$_0$ = 0.0075 M; MeCN-d_3, 20 °C), and simulated profiles (lines) calculated from kinetic parameters obtained by global numerical fitting of all profiles – and those from the other 14 runs – to the telescoped kinetic model shown in Figure 6 and Scheme S3. Profiles obtained by *in situ* ^{1}H NMR monitoring.

Global numerical fitting: Run 9

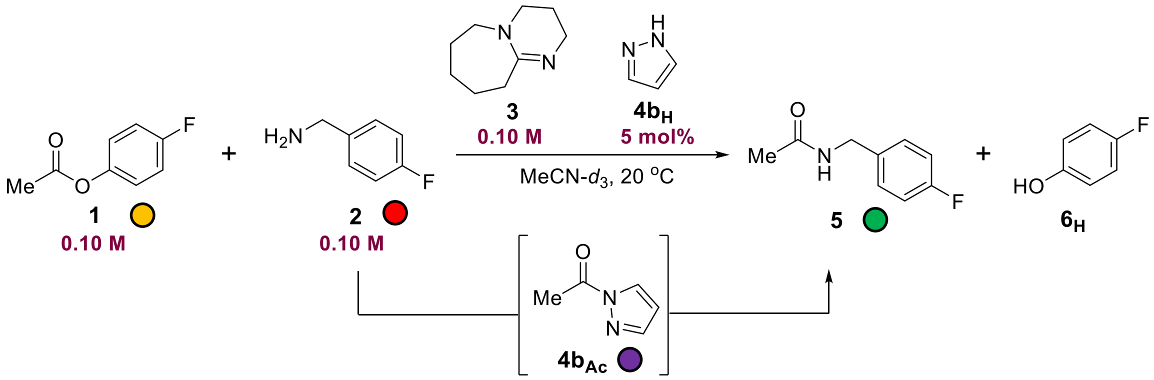

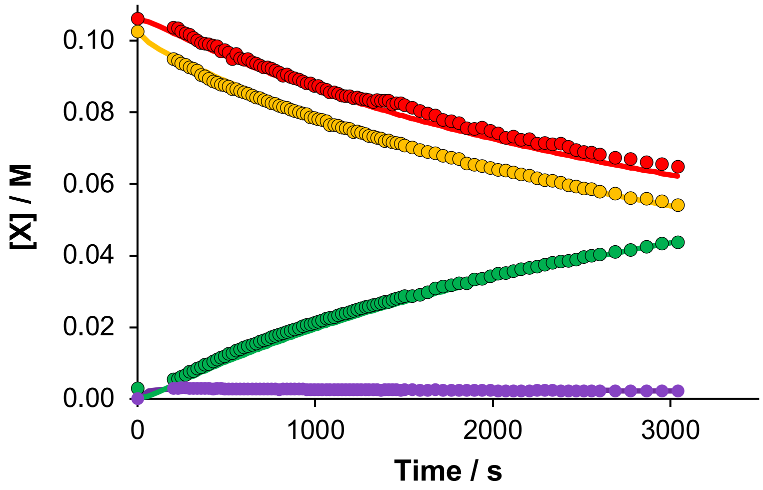

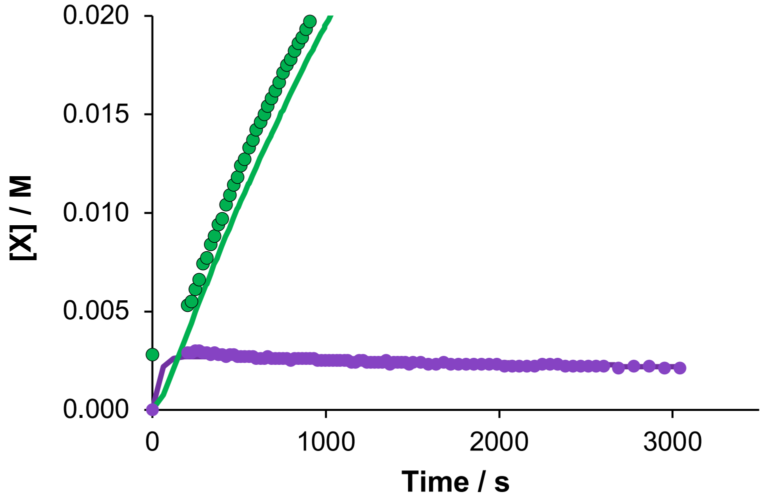

Figure S35: Experimental concentration-time profiles (points) for **1**, **2**, **5** and **4b$_{Ac}$** under regime III (*run 9*: [**1**]$_0$ = 0.10 M, [**2**]$_0$ = 0.10 M, [**3**]$_0$ = 0.10 M, [**4b$_H$**]$_0$ = 0.0050 M; MeCN-d_3, 20 °C), and simulated profiles (lines) calculated from kinetic parameters obtained by global numerical fitting of all profiles – and those from the other 14 runs – to the telescoped kinetic model shown in Figure 6 and Scheme S3. Profiles obtained by *in situ* ^{1}H NMR monitoring.

Global numerical fitting: Run 10

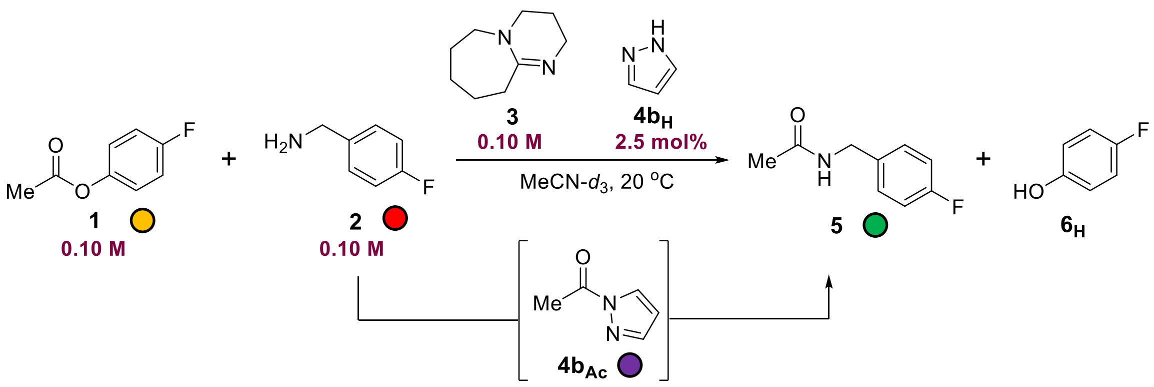

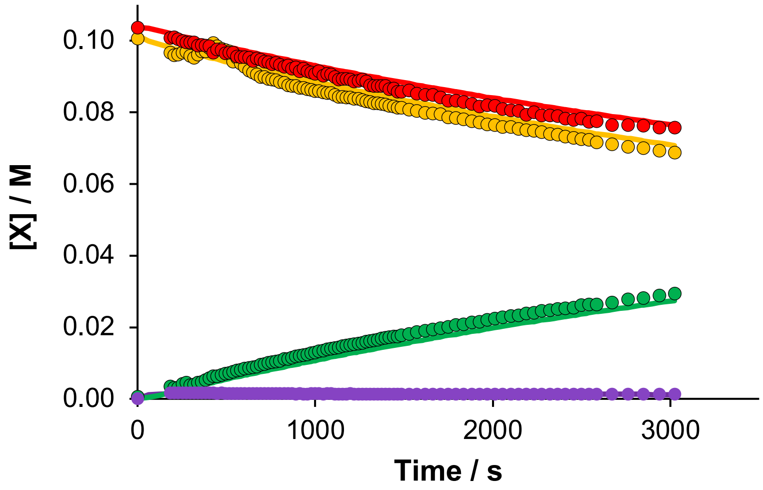

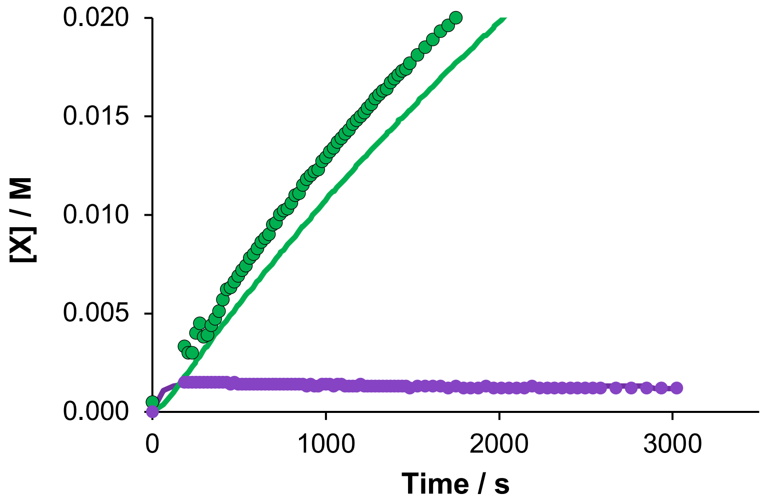

Figure S36: Experimental concentration-time profiles (points) for **1**, **2**, **5** and **4b$_{Ac}$** under regime III (*run 10*: [**1**]$_0$ = 0.10 M, [**2**]$_0$ = 0.10 M, [**3**]$_0$ = 0.10 M, [**4b$_H$**]$_0$ = 0.0025 M; MeCN-d_3, 20 °C), and simulated profiles (lines) calculated from kinetic parameters obtained by global numerical fitting of all profiles – and those from the other 14 runs – to the telescoped kinetic model shown in Figure 6 and Scheme S3. Profiles obtained by *in situ* ^{1}H NMR monitoring.

Global numerical fitting: Run 11

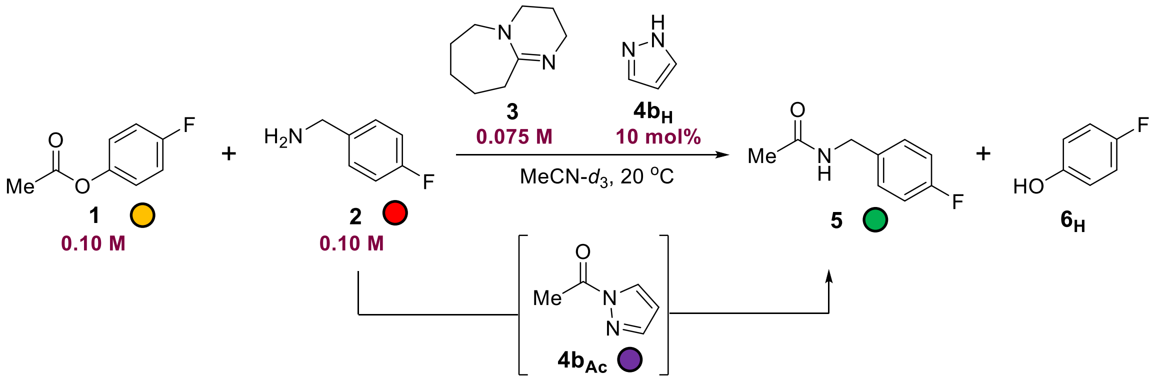

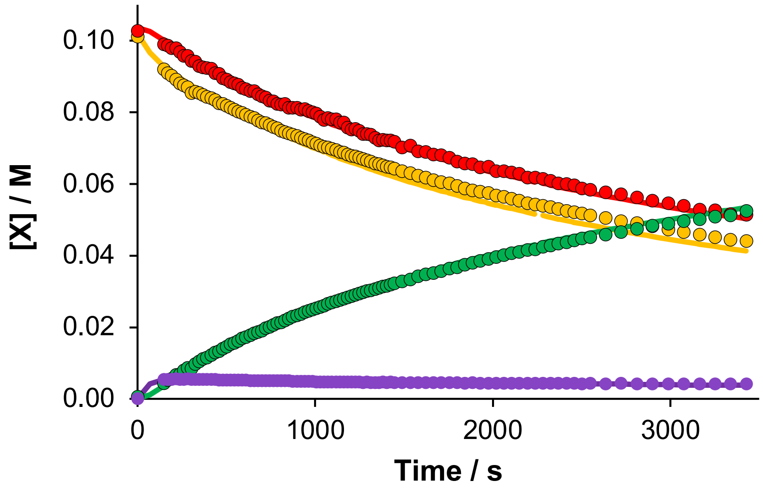

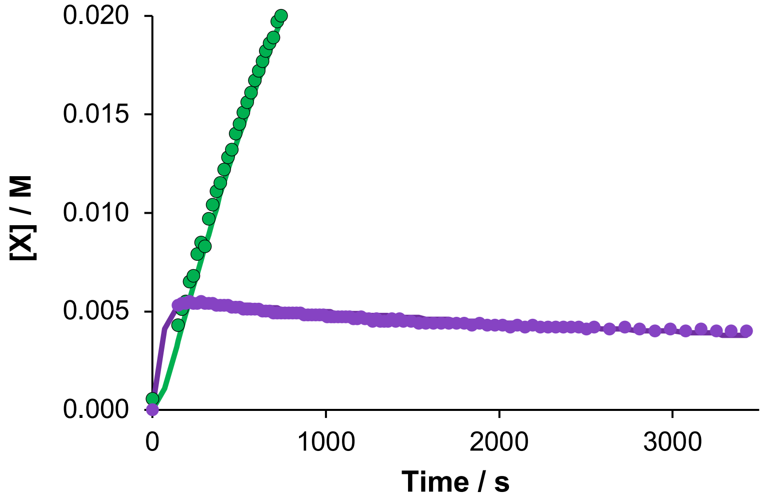

Figure S37: Experimental concentration-time profiles (points) for **1**, **2**, **5** and **4b$_{Ac}$** under regime III (*run 11*: [**1**]$_0$ = 0.10 M, [**2**]$_0$ = 0.10 M, [**3**]$_0$ = 0.075 M, [**4b$_H$**]$_0$ = 0.010 M; MeCN-d_3, 20 °C), and simulated profiles (lines) calculated from kinetic parameters obtained by global numerical fitting of all profiles – and those from the other 14 runs – to the telescoped kinetic model shown in Figure 6 and Scheme S3. Profiles obtained by *in situ* ^{1}H NMR monitoring.

Global numerical fitting: Run 12

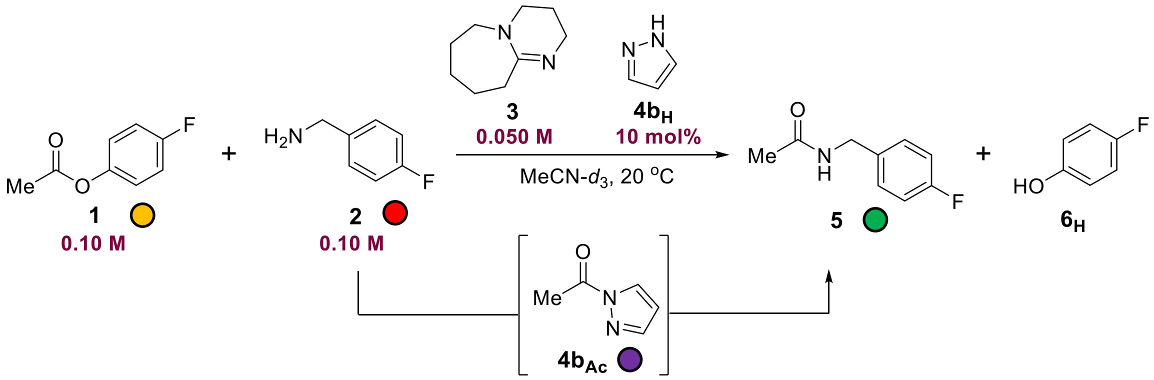

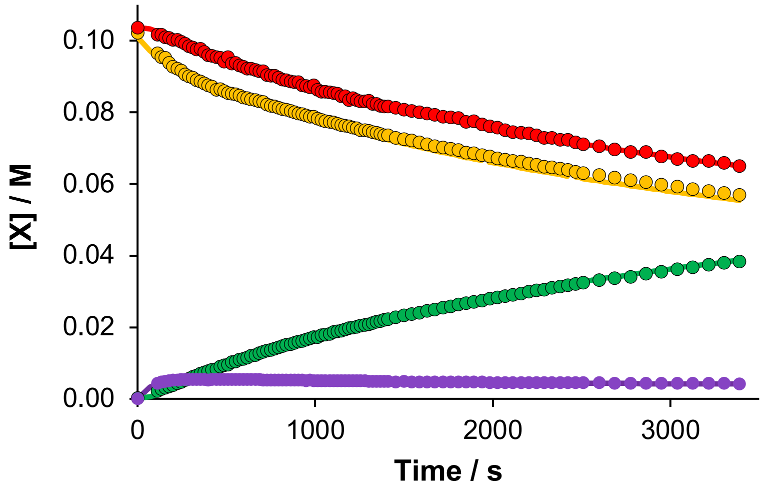

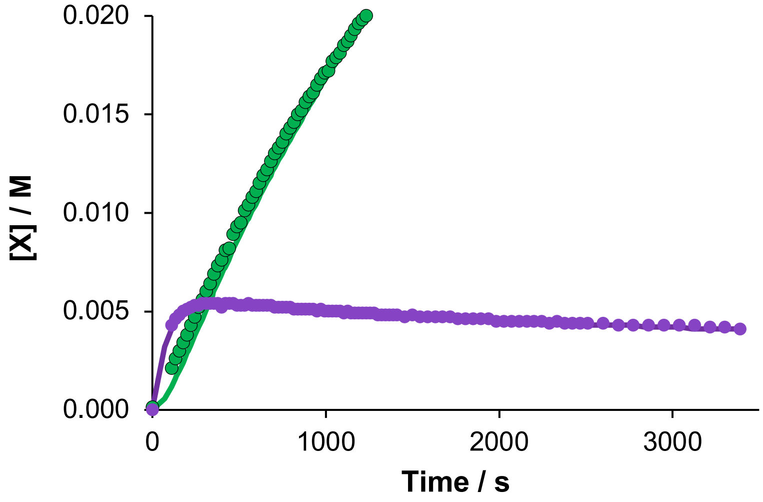

Figure S38: Experimental concentration-time profiles (points) for **1**, **2**, **5** and **4b$_{Ac}$** under regime III (*run 12*: [**1**]$_0$ = 0.10 M, [**2**]$_0$ = 0.10 M, [**3**]$_0$ = 0.050 M, [**4b$_H$**]$_0$ = 0.010 M; MeCN-d_3, 20 °C), and simulated profiles (lines) calculated from kinetic parameters obtained by global numerical fitting of all profiles – and those from the other 14 runs – to the telescoped kinetic model shown in Figure 6 and Scheme S3. Profiles obtained by *in situ* ^{1}H NMR monitoring.

Global numerical fitting: Run 13

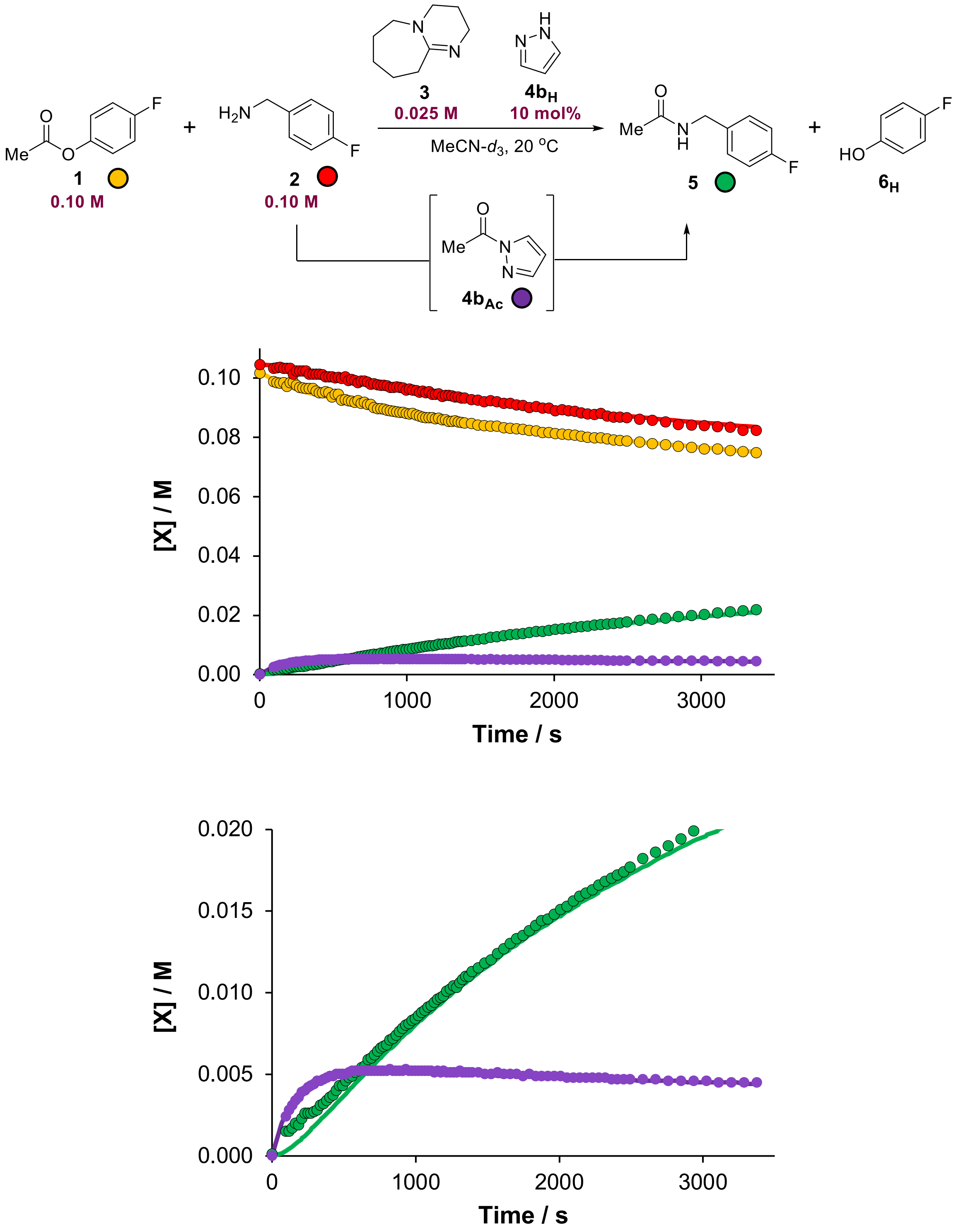

Figure S39: Experimental concentration-time profiles (points) for **1**, **2**, **5** and **4b$_{Ac}$** under regime III (*run 13*: [**1**]$_0$ = 0.10 M, [**2**]$_0$ = 0.10 M, [**3**]$_0$ = 0.025 M, [**4b$_H$**]$_0$ = 0.010 M; MeCN-d_3, 20 °C), and simulated profiles (lines) calculated from kinetic parameters obtained by global numerical fitting of all profiles – and those from the other 14 runs – to the telescoped kinetic model shown in Figure 6 and Scheme S3. Profiles obtained by *in situ* ^{1}H NMR monitoring.

Global numerical fitting: Run 14 (VR-SF-¹H NMR)

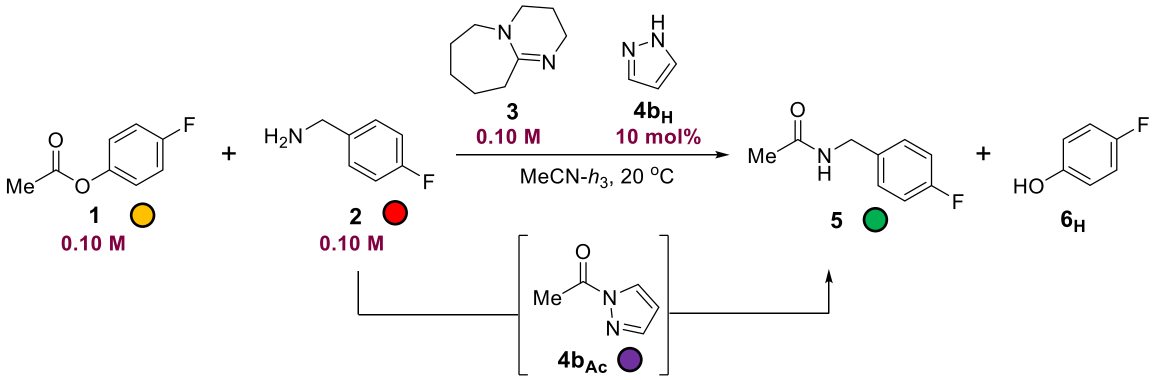

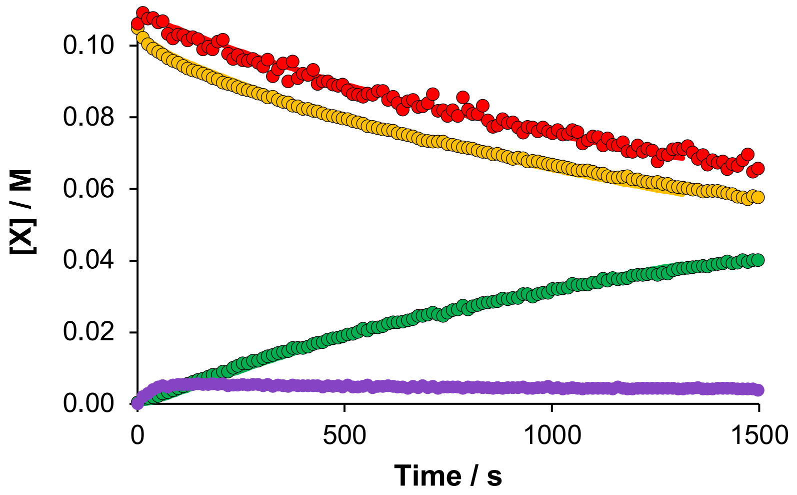

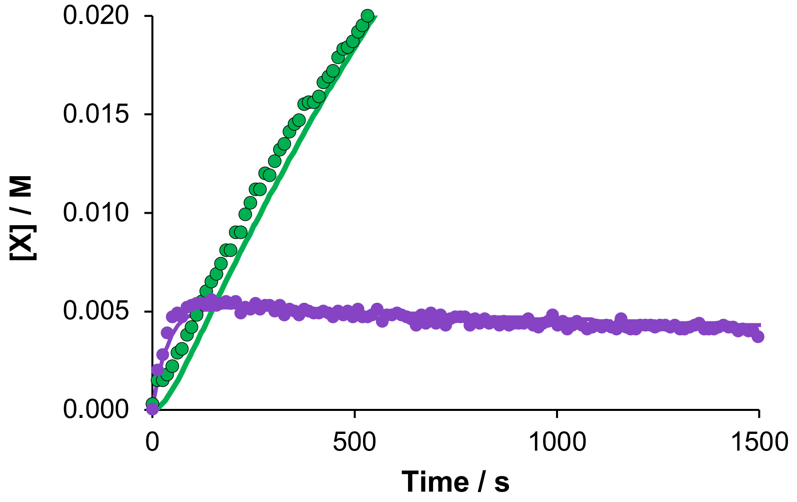

Figure S40: Experimental concentration-time profiles (points) for **1**, **2**, **5** and **4b$_{Ac}$** under regime III (*run 14*: [**1**]$_0$ = 0.10 M, [**2**]$_0$ = 0.10 M, [**3**]$_0$ = 0.10 M, [**4b$_H$**]$_0$ = 0.010 M; MeCN, 20 °C), and simulated profiles (lines) calculated from kinetic parameters obtained by global numerical fitting of all profiles – and those from the other 14 runs – to the telescoped kinetic model shown in Figure 6 and Scheme S3. Profiles obtained by VR-SF-¹H NMR monitoring.

Global numerical fitting: Run 15 (VR-SF-¹H NMR)

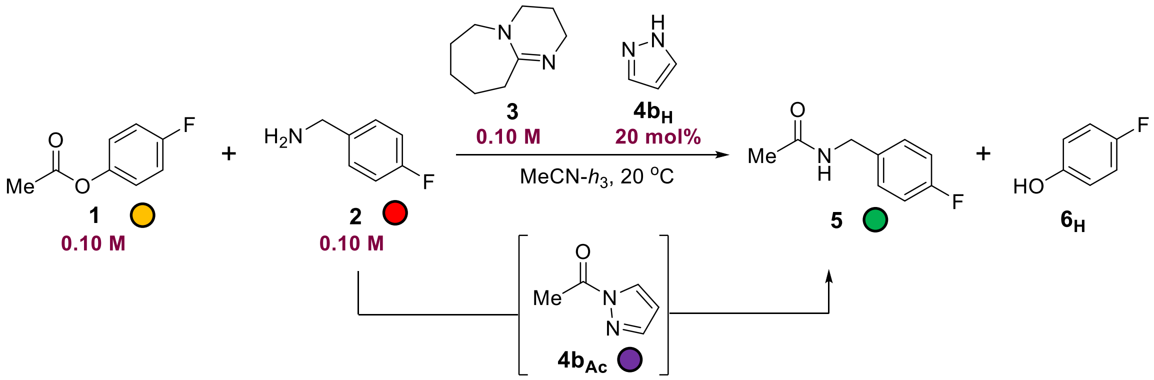

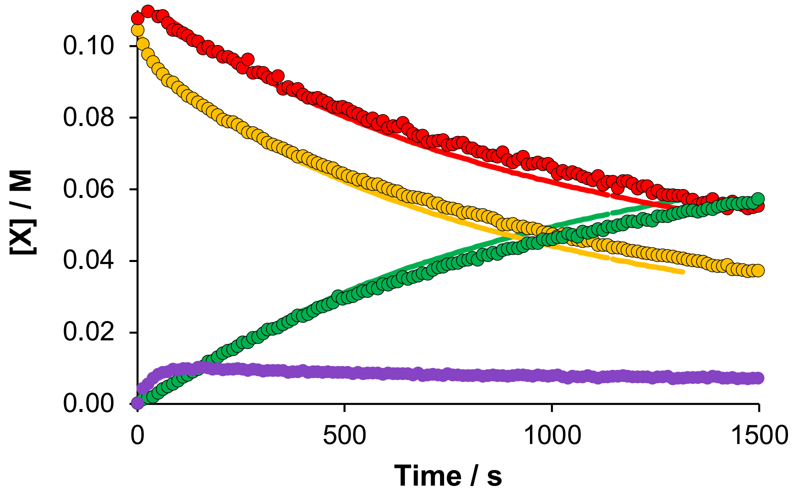

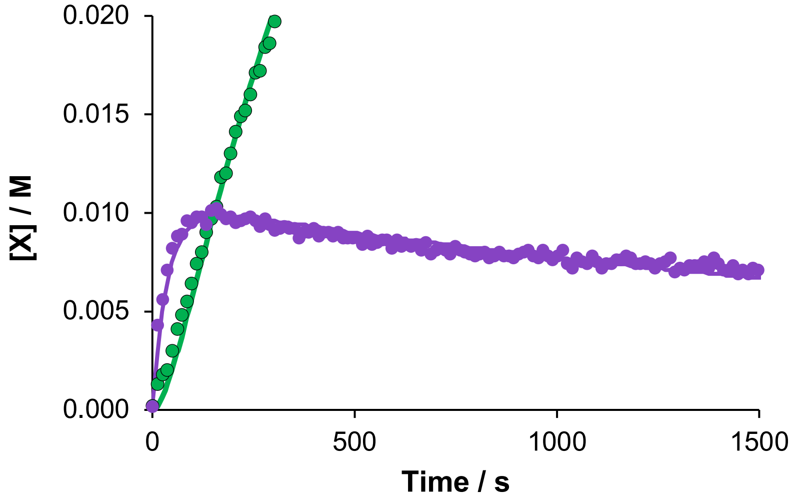

Figure S41: Experimental concentration-time profiles (points) for **1**, **2**, **5** and **4b$_{Ac}$** under regime III (*run 15*: [**1**]$_0$ = 0.10 M, [**2**]$_0$ = 0.10 M, [**3**]$_0$ = 0.10 M, [**4b$_H$**]$_0$ = 0.020 M; MeCN, 20 °C), and simulated profiles (lines) calculated from kinetic parameters obtained by global numerical fitting of all profiles – and those from the other 14 runs – to the telescoped kinetic model shown in Figure 6 and Scheme S3. Profiles obtained by VR-SF-¹H NMR monitoring.

S3.8.5 Regime IV Kinetics

Raw product evolution profiles

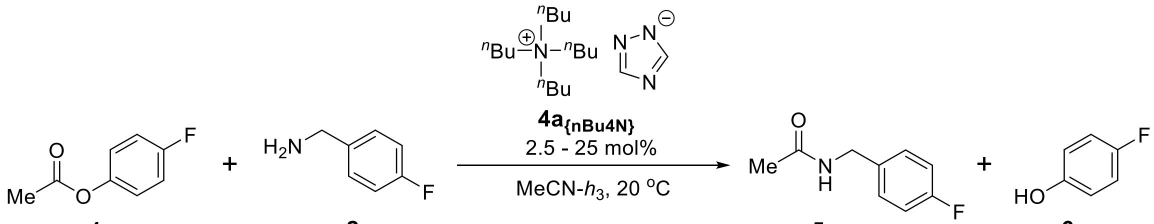

1 0.025 - 0.10 M; **2** 0.025 - 0.10 M; **5**; **6_H** 0 - 0.050 M

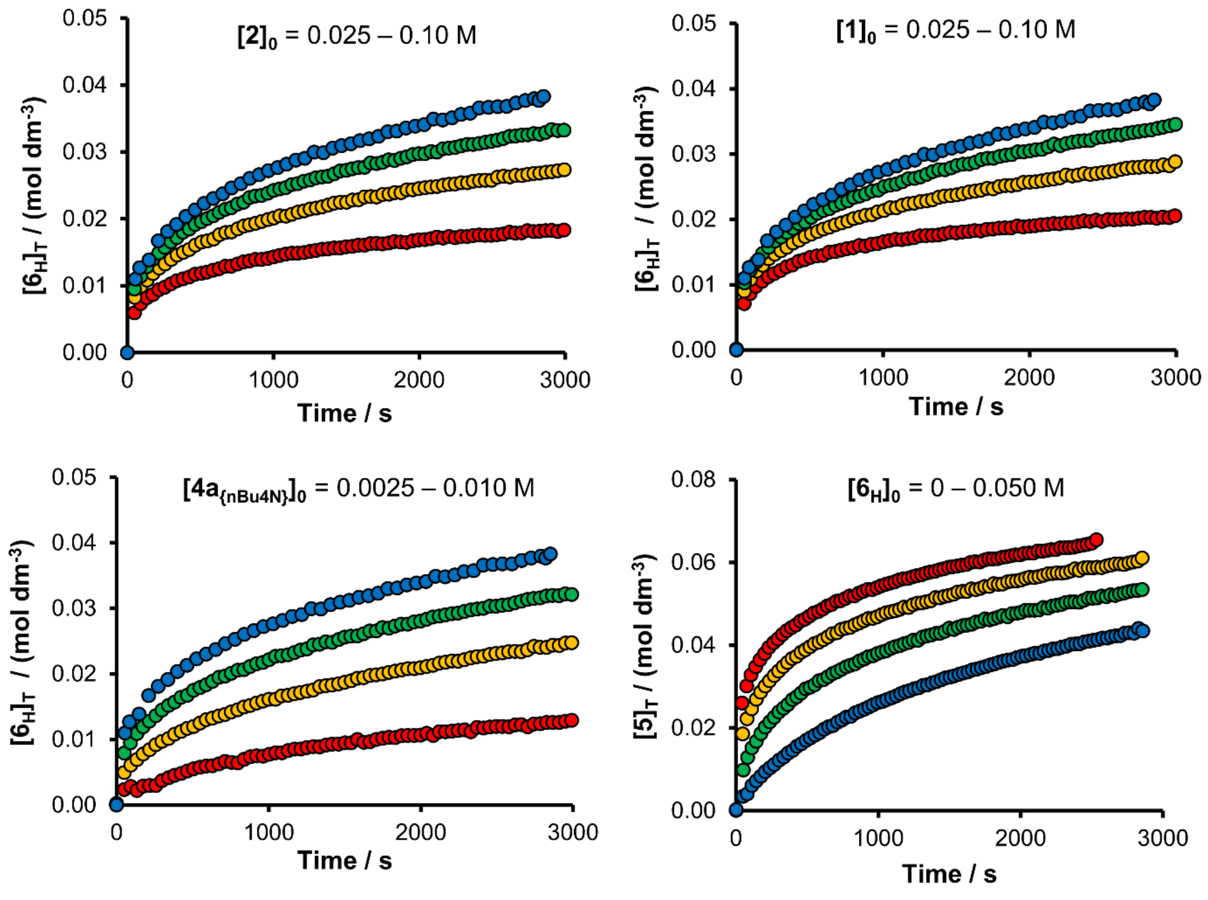

Figure S42: Product evolution profiles for the aminolysis of *p*-F-PhAc **1** with *p*-F-BnNH$_2$ **2** and *n*-tetrabutylammonium 1,2,4-triazolate **4a$_{\{nBu4N\}}$** in MeCN at 20 °C, *without* auxiliary DBU, obtained by *in situ* ^{19}F NMR spectroscopy. For kinetic runs initiated without exogenous *p*-F-PhOH **6$_H$** (A-C), the product evolution corresponds to the total concentration of liberated **6$_H$**; for kinetic runs initiated with the addition of exogenous **6$_H$**, the product evolution corresponds to the concentration of *p*-F-BnNHAc **5**. (A) Aminolysis of **1** (0.10 M) with variable **2** (0.025 – 0.10 M) and **4a$_{\{nBu4N\}}$** (10 mol%); (B) Aminolysis of variable **1** (0.025 - 0.10 M) with **2** (0.10 M), and **4a$_{\{nBu4N\}}$** (10 mol%); (C) Aminolysis of **1** (0.10 M) with **2** (0.10 M) and variable **4a$_{\{nBu4N\}}$** (2.5 - 10 mol%); (D) Aminolysis of **1** (0.10 M) with **2** (0.10 M), **4a$_{\{nBu4N\}}$** (25 mol%), and exogenous **6$_H$** (0 – 0.050 M).

Normalized product evolution profiles

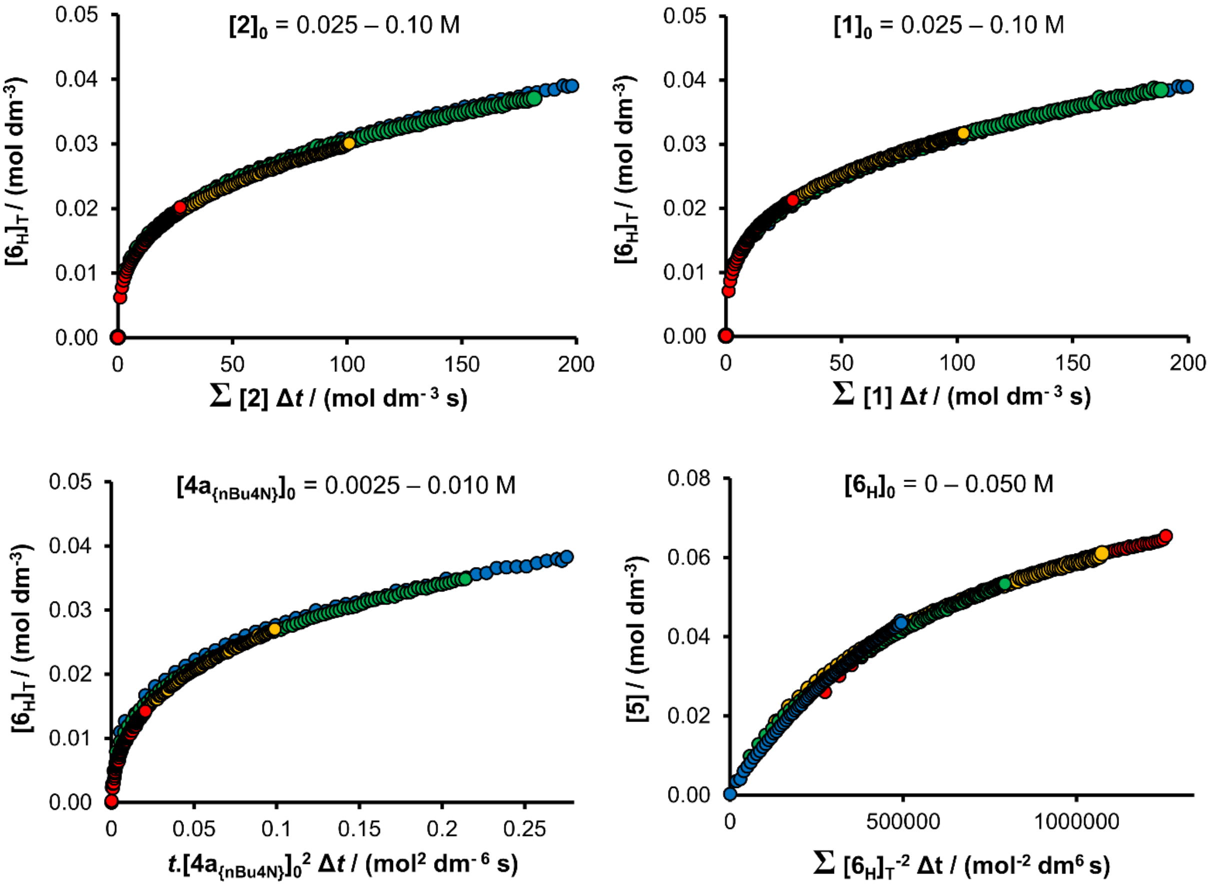

Figure S43: Single-component normalised product evolution profiles for each of the components involved in the aminolysis of *p*-F-PhAc **1** with *p*-F-BnNH$_2$ **2** and *n*-tetrabutylammonium 1,2,4-triazolate **4a**$_{\{nBu4N\}}$ in MeCN at 20 °C (raw kinetics: Figure S42). Whilst an inverse second-order dependence on exogenous *p*-F-PhOH **6**$_H$ was observed at certain concentrations (0.010 – 0.050 M; shown), more complex behaviour was observed during attempts to fully normalise product evolution profiles obtained in the absence of exogenous **6**$_H$.

S3.8.6 Isothermal kinetic studies of N-acetylated azoles

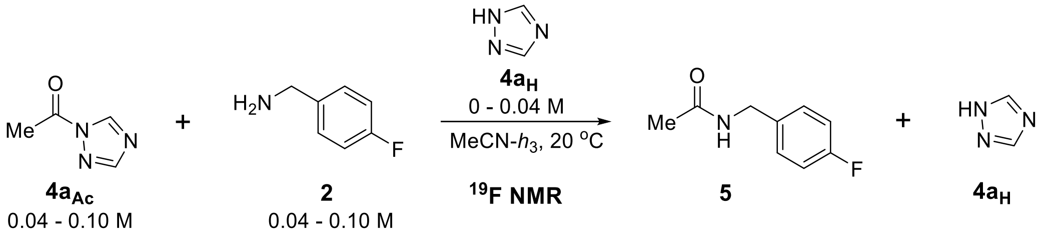

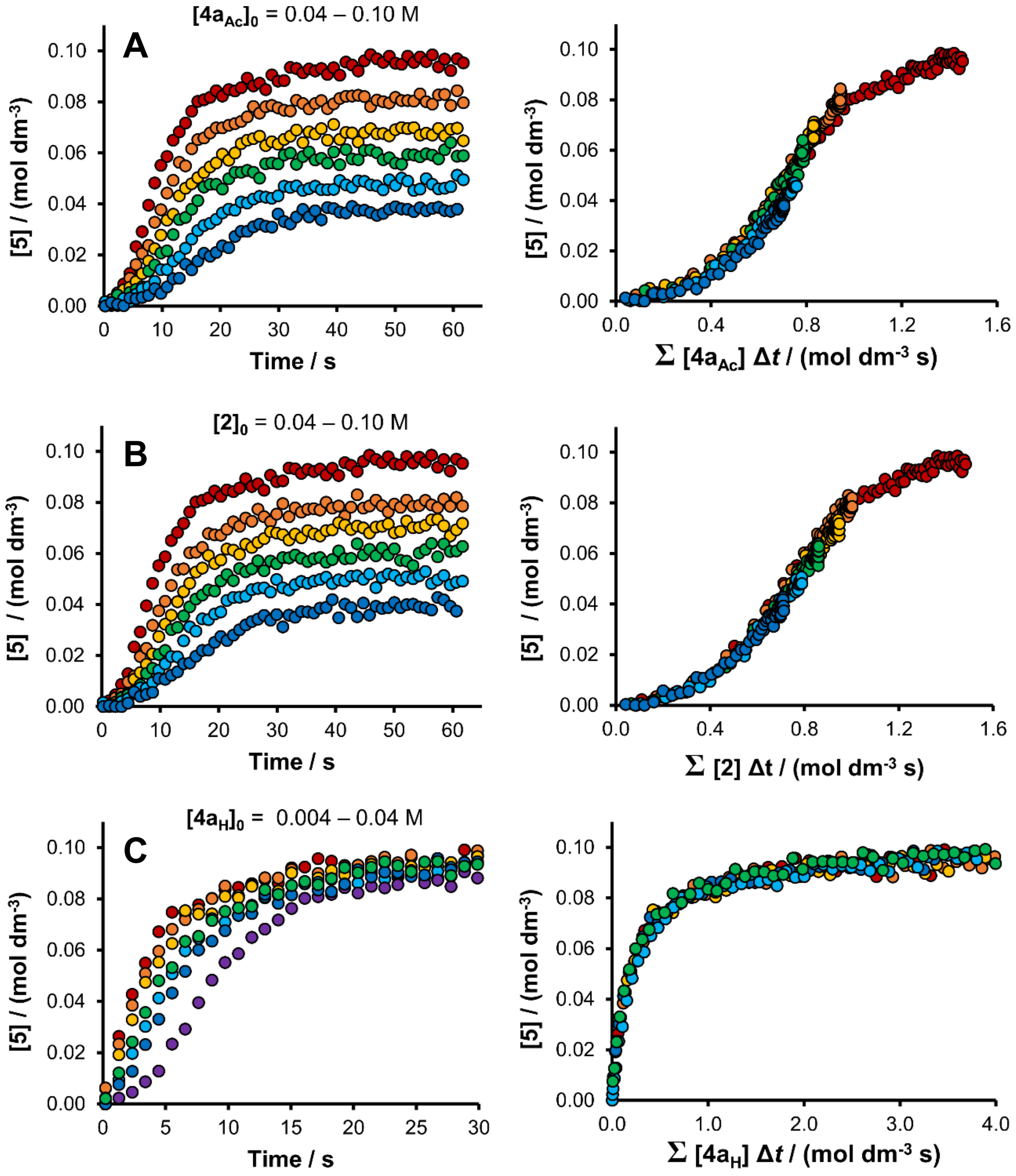

Figure S44: Concentration-time profiles and normalised profiles for *p*-F-BnNHAc **5** formed during the aminolysis of 1-acetyl-1,2,4-triazole **4a$_{Ac}$** with *p*-F-BnNH$_2$ **2** and 1,2,4-triazole **4a$_H$** in MeCN (20 °C). (A) Aminolysis of **4a$_{Ac}$** (0.04 – 0.10 M) with **2** (0.10 M). (B) Aminolysis of **4a$_{Ac}$** (0.10 M) with **2** (0.04 – 0.10 M). (C) Aminolysis of **4a$_{Ac}$** (0.10 M) with **2** (0.10 M) and exogenous **4a$_H$** (0.004 – 0.04 M). Analysed by VR-SF-^{19}F NMR spectroscopy using 1-fluoronaphthalene (0.050 M) as an internal integration standard.

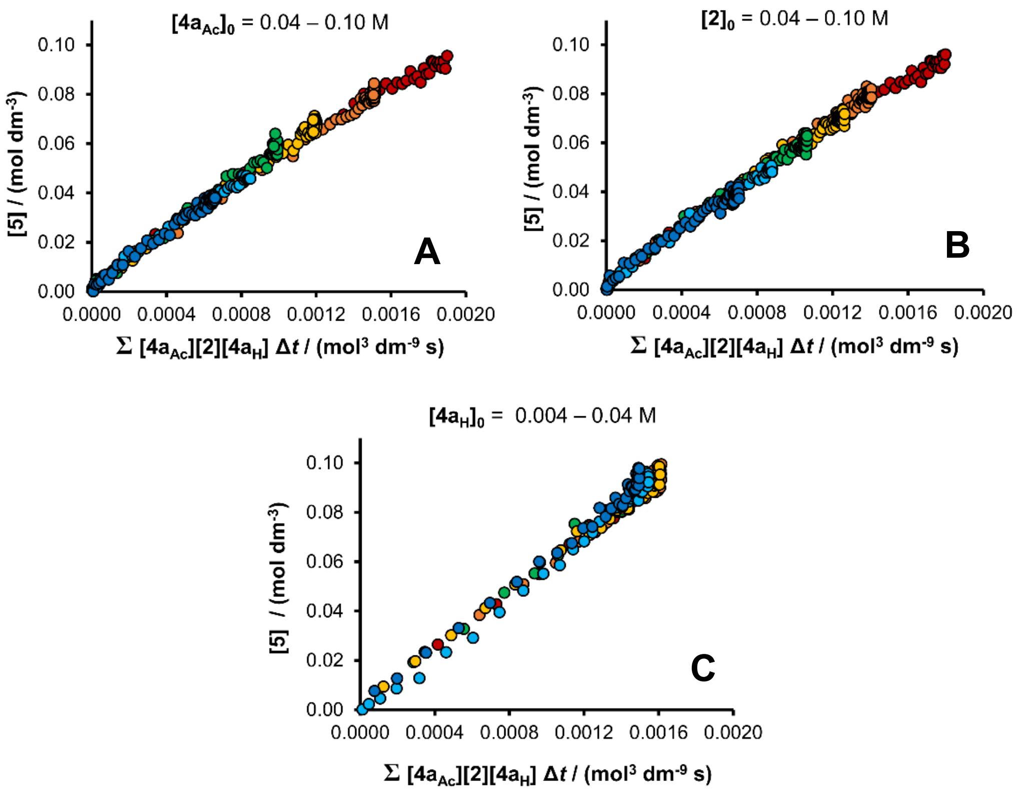

Figure S45: Fully normalised kinetic profiles for *p*-F-BnNHAc **5** formed during the aminolysis of 1-acetyl-1,2,4-triazole **4a**$_{\mathbf{Ac}}$ with *p*-F-BnNH$_2$ **2** and 1,2,4-triazole **4a**$_{\mathbf{H}}$ (0 – 0.04 M) in MeCN (20 °C) (raw kinetics: Figure S44). (**A**) Aminolysis of variable **4a**$_{\mathbf{Ac}}$ (0.04 – 0.10 M) with **2** (0.10 M). (**B**) Aminolysis of **4a**$_{\mathbf{Ac}}$ (0.10 M) with variable **2** (0.04 – 0.10 M). (**C**) Aminolysis of **4a**$_{\mathbf{Ac}}$ (0.10 M) with **2** (0.10 M) and exogenous **4a**$_{\mathbf{H}}$ (0.004 – 0.04 M). Reactions assembled by variable-ratio stopped-flow apparatus and concentrations measured by ^{19}F NMR spectroscopy, using 1-fluoronaphthalene (0.050 M) as an internal integration standard. Kinetic consistent with autocatalytic termolecular rate law $v = k_{obs}$[**4a**$_{\mathbf{Ac}}$][**2**][**4a**$_{\mathbf{H}}$].

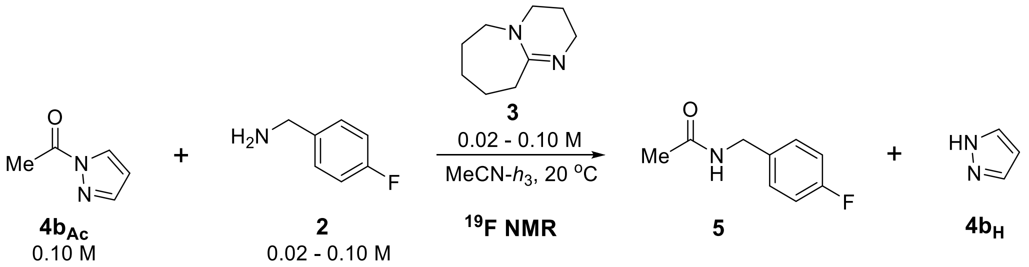

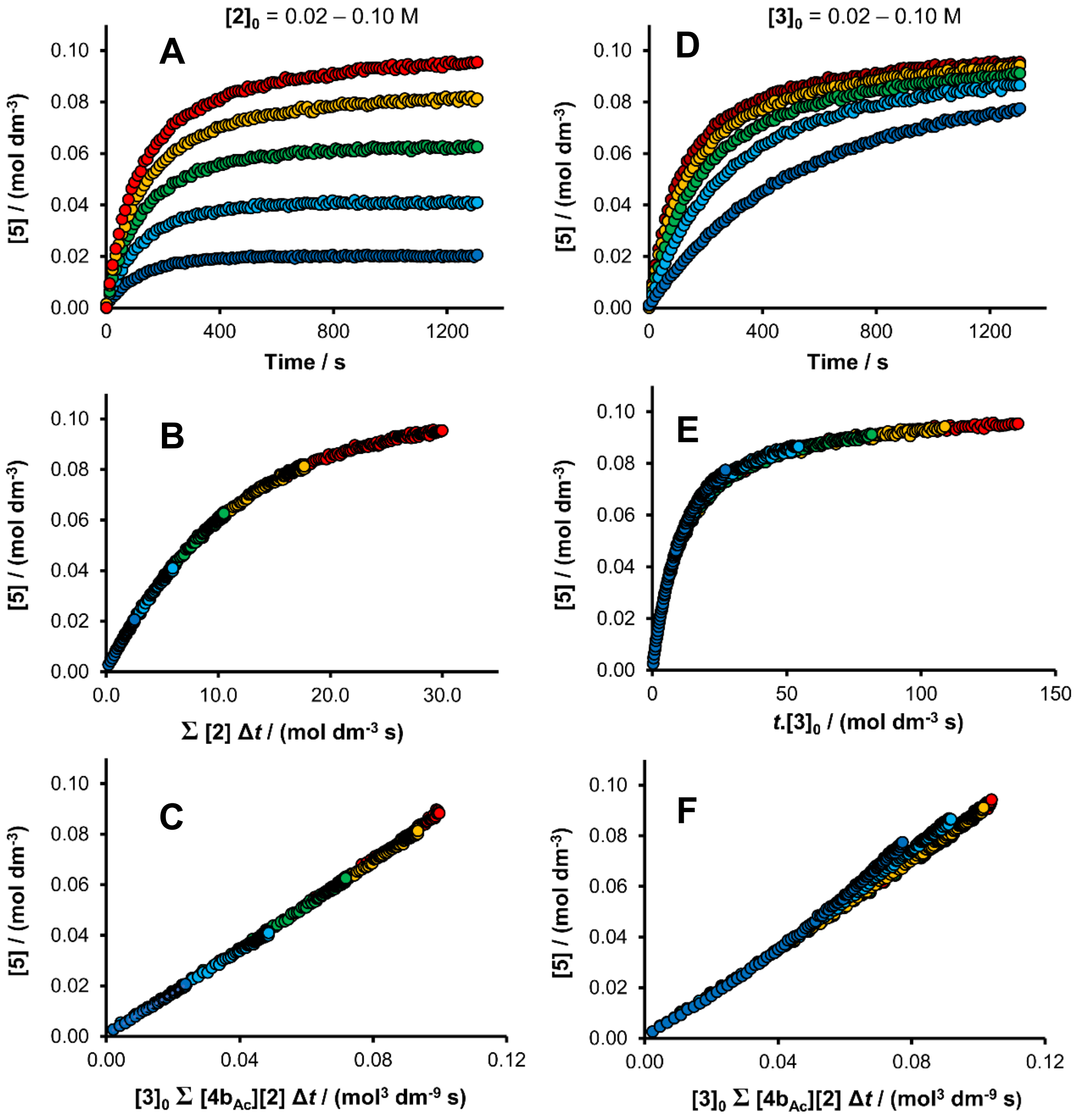

Figure S46: Temporal-concentration profiles and normalised profiles for *p*-F-BnNHAc **5** formed during the aminolysis of N-acetyl pyrazole **4b$_{Ac}$** with *p*-F-BnNH$_2$ **2** and DBU **3** in MeCN (20 °C). (**A, B, C**) Aminolysis of **4b$_{Ac}$** (0.10 M) with variable **2** (0.04 – 0.10 M) and **3** (0.10 M). (**D, E, F**) Aminolysis of **4b$_{Ac}$** (0.10 M) with **2** (0.10 M) and variable **3** (0.02 – 0.10 M). Reactions assembled by variable-ratio stopped-flow apparatus and concentraitons measured by ^{19}F NMR spectroscopy, using 1-fluoronaphthalene (0.050 M) as an internal integration standard.

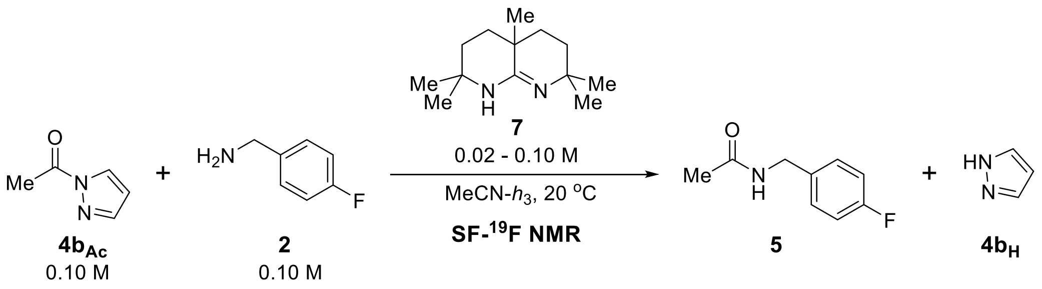

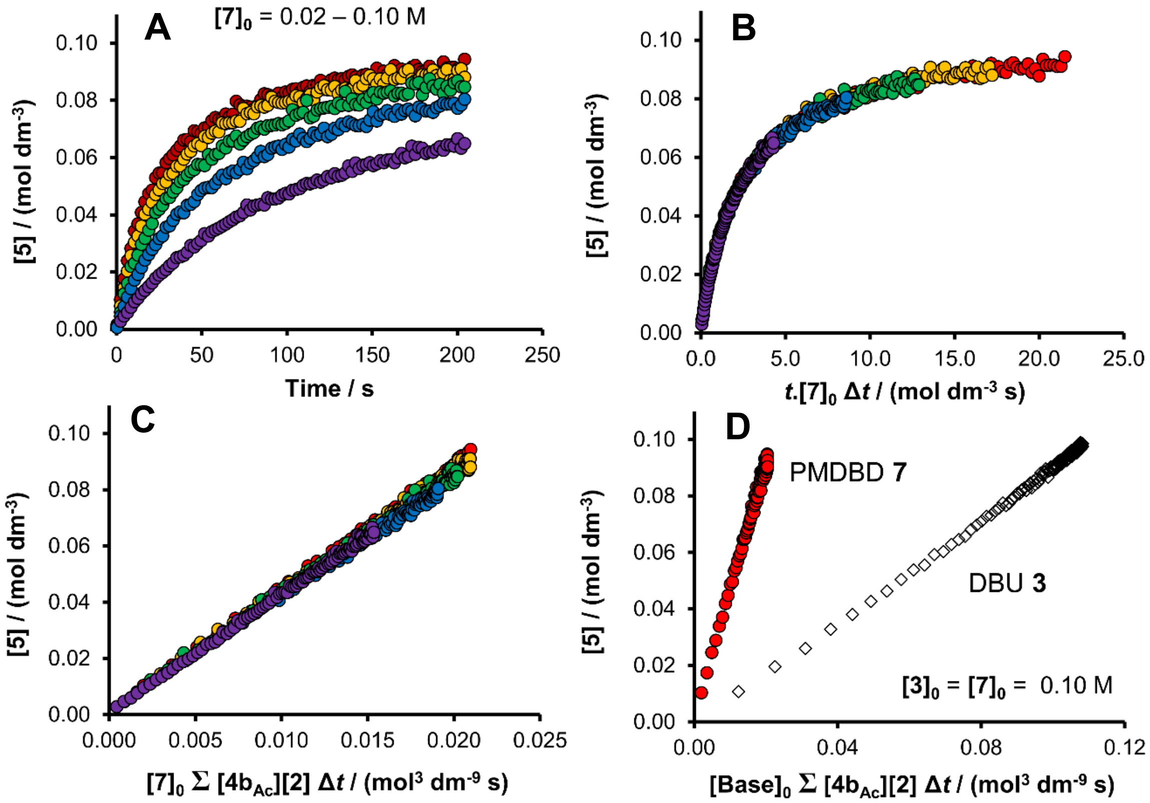

Figure S47: Product evolution profiles, single-component normalised profiles and fully normalised profiles for the base-catalysed aminolysis of N-acetyl pyrazole **4b$_{Ac}$** (0.10 M) with p-F-BnNH$_2$ **2** (0.10 M) in MeCN (20 °C). (**A**) Temporal concentration profiles of p-F-BnNHAc **5** in the aminolysis of **4b$_{Ac}$** (0.10 M) with **2** (0.10 M) and variable loadings of PMDBD **7** (0.020 – 0.10 M). (**B**) Kinetic profiles normalised assuming a constant catalyst concentration of [**7**]$_0$ = 0.020 – 0.10 M. (**C**) Fully normalised kinetic profiles for variable **7**, calculated according to v = $k_{obs}^{(20\ °C)}$[**4b$_{Ac}$**][**2**][**7**]$_0$. (**D**) Comparison of $k_{obs}^{(T)}$ obtained with **7** (0.10 M; $k_{obs}^{(20\ °C)}$ = 4.2 M^{-2} s^{-1}) and DBU **3** (0.10 M; $k_{obs}^{(20\ °C)}$ = 0.9 M^{-2} s^{-1}) under otherwise identical conditions ([**4b$_{Ac}$**]$_0$ = 0.10 M; [**2**]$_0$ = 0.10 M; MeCN, 20 °C). All kinetic profiles obtained by VR-SF-^{19}F NMR spectroscopy using 1-F-naphthalene 1-F-Nap as an internal integration standard (0.050 M).

S3.8.7 Variable-temperature kinetic studies

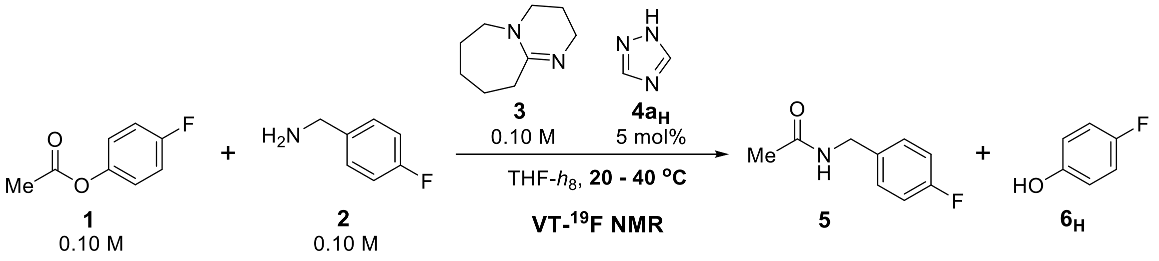

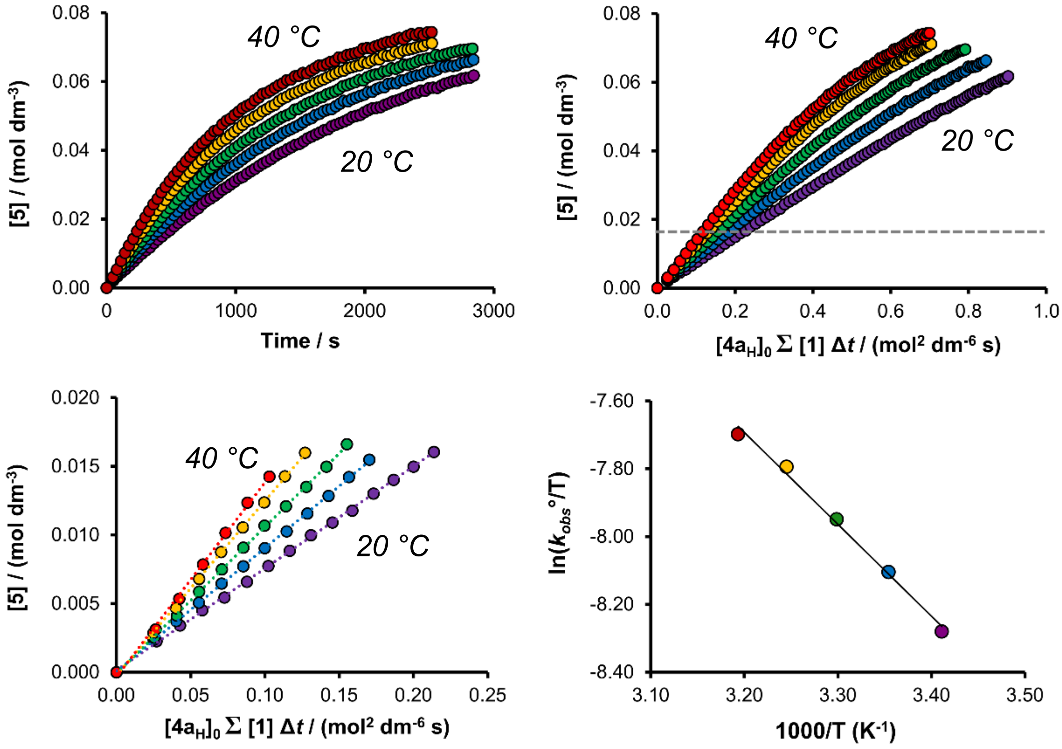

Figure S48: Variable temperature analysis (20 – 40 °C; 5°C increments) of the aminolysis of *p*-F-PhAc **1** (0.10 M) with *p*-F-BnNH$_2$ **2** (0.10 M), stoichiometric DBU **3** (0.10 M) and catalytic 1,2,4-triazole **4a$_H$** (5 mM, 5 mol%) in THF-*h*$_8$. Reactions assembled manually and product evolution profiles obtained by *in situ* ^{19}F NMR spectroscopy, using 1-F-naphthalene as an internal integration standard (0.050 M). Product evolution profiles normalised according to the empirical rate law $v = k_{obs}^{(T)}$[**1**][**4a$_H$**]$_0$, assuming a constant anion concentration of [**4a$_{\{DBUH\}}$**] ≈ [**4a$_H$**]$_0$ = 5 mM at early conversions (F < 20 %). $k_{obs}^{(20°C)}$ = 0.074 M^{-1} s^{-1}, $k_{obs}^{(25°C)}$ = 0.090 M^{-1} s^{-1}, $k_{obs}^{(30°C)}$ = 0.107 M^{-1} s^{-1}, $k_{obs}^{(35°C)}$ = 0.127 M^{-1} s^{-1}, $k_{obs}^{(40°C)}$ = 0.142 M^{-1} s^{-1}. $\Delta^{\ddagger}H$ = 23 kJ mol^{-1}, $\Delta^{\ddagger}S$ = -189 J K^{-1} mol^{-1}, $\Delta^{\ddagger}G_{293K}$ = 78 kJ mol^{-1}. $k° = k_{obs}^{(T)}.c°$, $c°$ = 1 M.

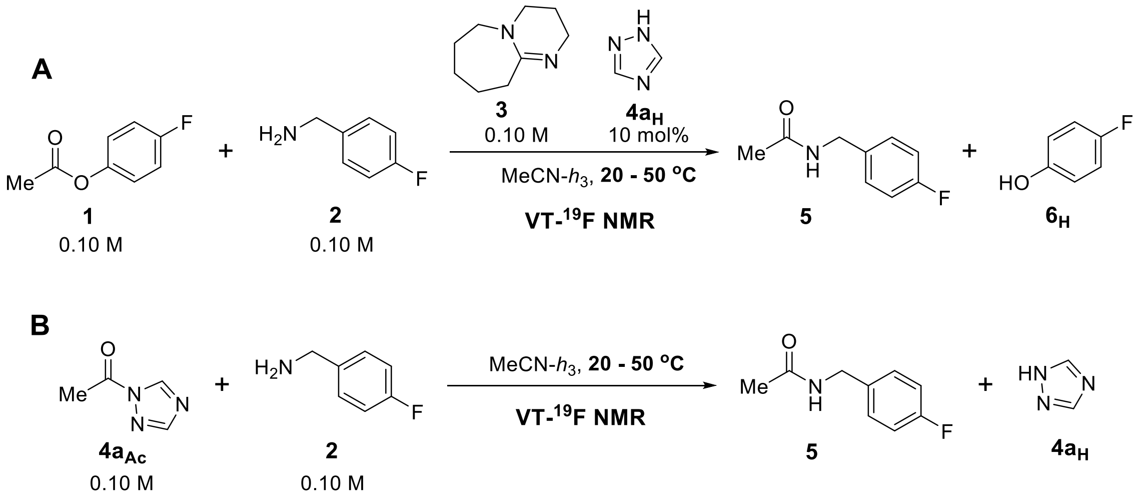

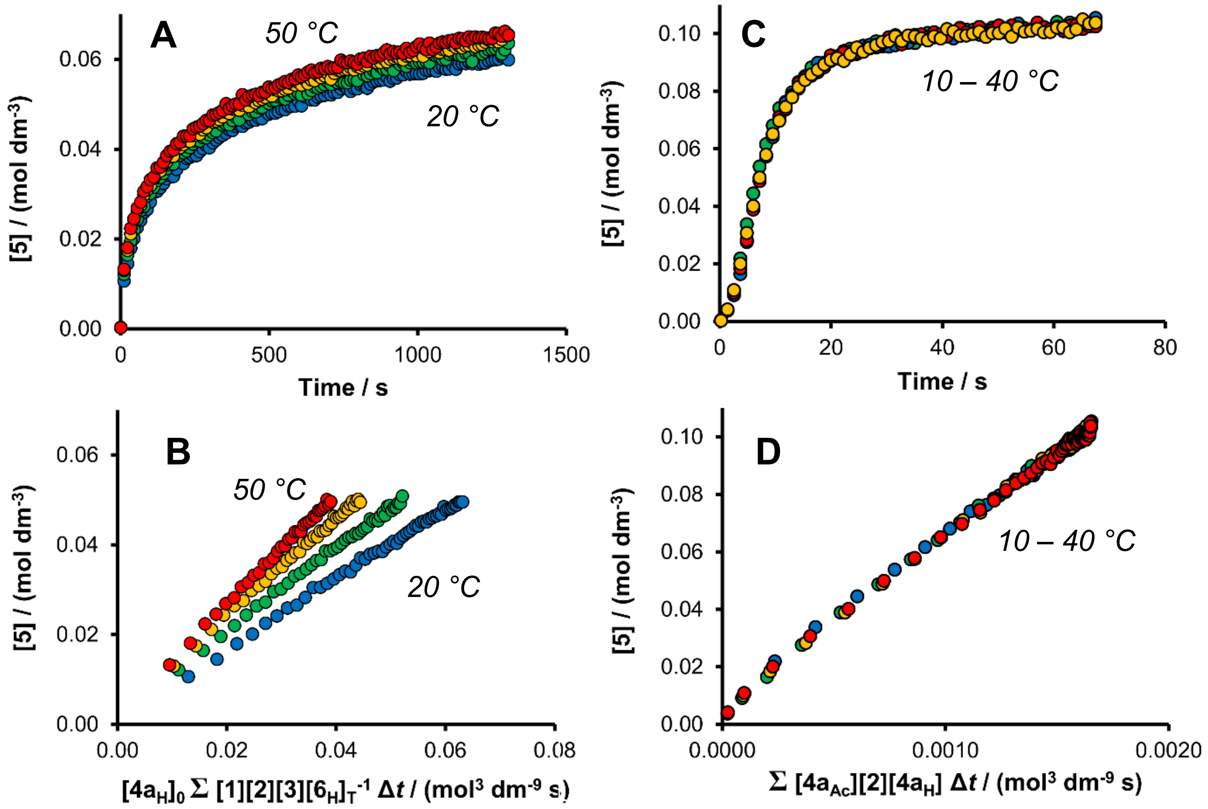

Figure S49: (**A**, **B**) Variable temperature kinetic profiles (20 – 50 °C; 10°C increments) for the aminolysis of *p*-F-PhAc **1** (0.10 M) with *p*-F-BnNH$_2$ **2** (0.10 M), stoichiometric DBU **3** (0.10 M) and catalytic 1,2,4-triazole **4a$_H$** (10 mM, 10 mol%) in MeCN. Product evolution profiles normalised according to the empirical rate law $v = k_{obs}^{(I)}$[**1**][**2**][**3**][**4a$_H$**]$_0$[**6$_H$**]$_T^{-1}$, assuming a constant catalyst concentration [**4a$_H$**]$_0$ = 10 mM and [**3**] = [**3**]$_0$ - 0.5[**6$_H$**]$_T$; normalized data truncated to ca 50 % conversion to account for this assumption (**C**, **D**) Variable temperature kinetic profiles (10 – 40 °C; 10°C increments) for the aminolysis of 1-acetyl-1,2,4-triazole **4a$_{Ac}$** (0.10 M) with **2** (0.10 M) in MeCN. Product evolution profiles normalised according to the empirical rate law $v = k_{obs}^{(T)}$[**4a$_{Ac}$**][**2**][**4a$_H$**]. Both reactions assembled and monitored by VR-SF-^{19}F NMR spectroscopy, using 1-F-naphthalene as an internal integration standard (0.050 M).

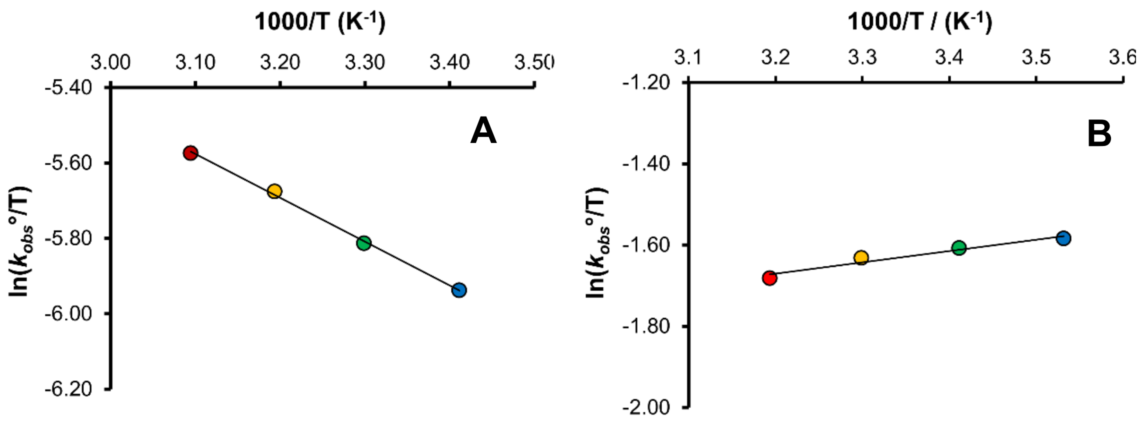

Figure S50: Eyring analyses of kinetic data in Figure S49. (**A**) Aminolysis of *p*-F-PhAc **1** (0.10 M) with *p*-F-BnNH$_2$ **2** (0.10 M), stoichiometric DBU **3** (0.10 M) and catalytic 1,2,4-triazole **4a$_H$**. $k_{obs}^{(20°C)}$ = 0.77 M^{-2} s^{-1}, $k_{obs}^{(30°C)}$ = 0.91 M^{-2} s^{-1}, $k_{obs}^{(40°C)}$ = 1.07 M^{-2} s^{-1}, $k_{obs}^{(50°C)}$ = 1.23 M^{-2} s^{-1}. $\Delta^{‡}H$ = 10 kJ mol^{-1}, $\Delta^{‡}S$ = -214 J K^{-1} mol^{-1}, $\Delta^{‡}G_{293K}$ = 72 kJ mol^{-1}. (**B**) Aminolysis of 1-acetyl-1,2,4-triazole **4a$_{Ac}$** (0.10 M) with **2** (0.10 M) in MeCN. $k_{obs}^{(10°C)}$ = 58.1 M^{-2} s^{-1}, $k_{obs}^{(20°C)}$ = 58.7 M^{-2} s^{-1}, $k_{obs}^{(30°C)}$ = 59.3 M^{-2} s^{-1}, $k_{obs}^{(40°C)}$ = 58.3 M^{-2} s^{-1}. $\Delta^{‡}H$ = -2 kJ mol^{-1}, $\Delta^{‡}S$ = -219 J K^{-1} mol^{-1}, $\Delta^{‡}G_{293K}$ = 62 kJ mol^{-1}. $k_{obs}°$ = $k_{obs}^{(T)}.c°^2$, $c°$ = 1 M.

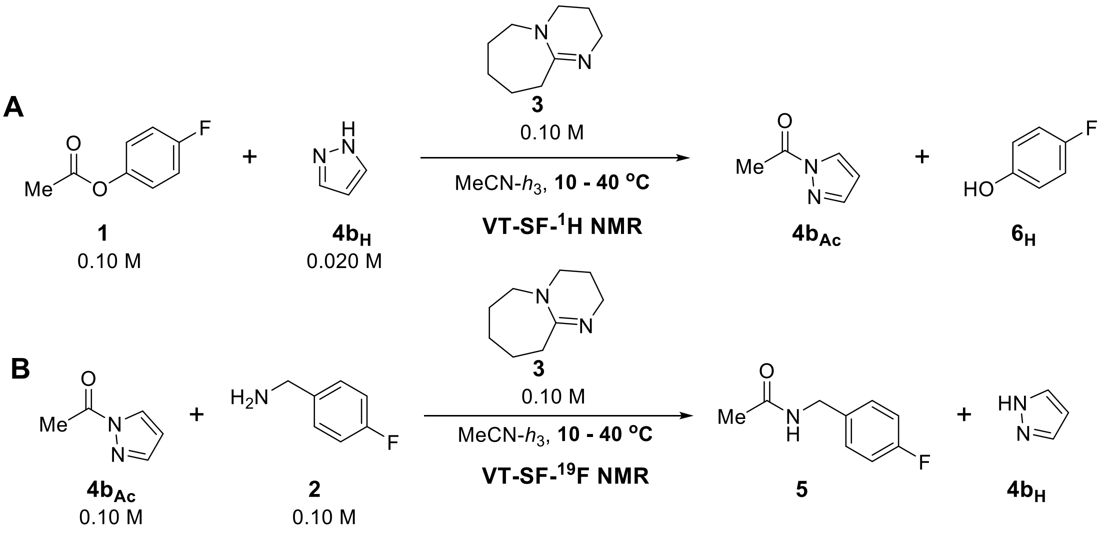

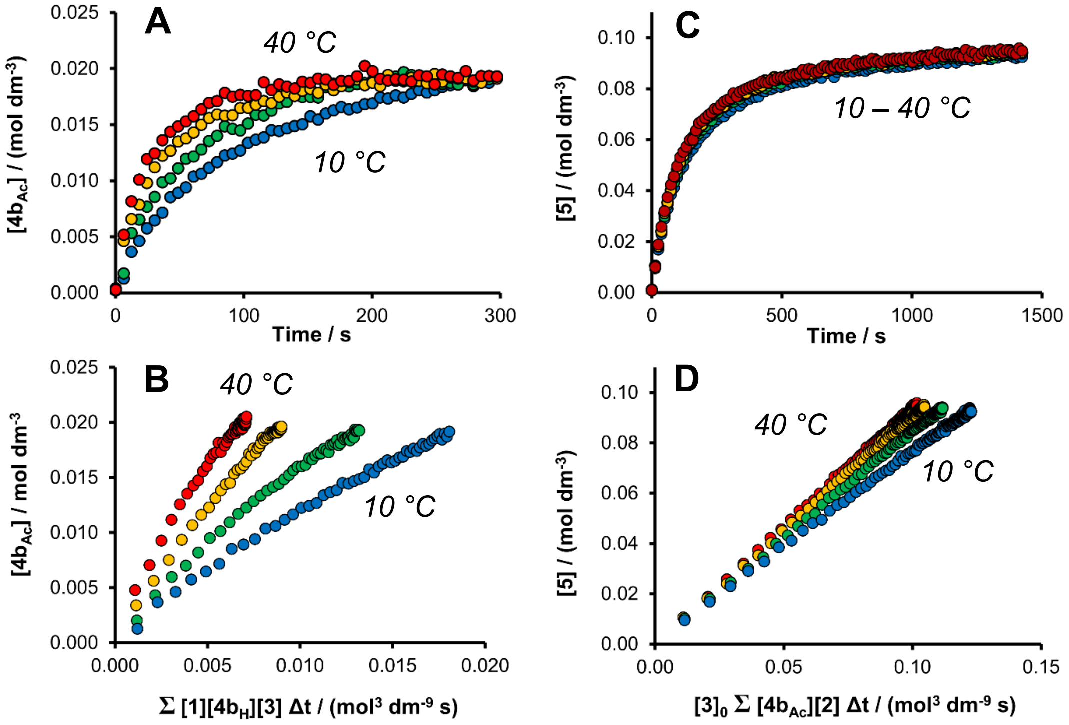

Figure S51: (**A**, **B**): Variable temperature kinetic profiles (10 – 40 °C; 10°C increments) for the acetylation of pyrazole (**4b$_H$**; 0.020 M) with *p*-F-PhAc **1** (0.10 M) and excess DBU **3** (0.10 M) in MeCN. Reactions assembled and monitored by SF-^{1}H NMR spectroscopy, using 1,3,5-trimethoxybenzene as an internal integration standard (0.033 M). Product evolution profiles normalised according to the empirical rate law $k_{obs}^{(T)}$[**1**][**4b$_H$**][**3**], assuming [**3**] = [**3**]$_0$ – 0.5.[**6$_H$**]$_T$. $k_{obs}^{(10°C)}$ = 1.09 M^{-2} s^{-1}, $k_{obs}^{(20°C)}$ = 1.57 M^{-2} s^{-1}, $k_{obs}^{(30°C)}$ = 2.22 M^{-2} s^{-1}, $k_{obs}^{(40°C)}$ = 3.01 M^{-2} s^{-1}. $\Delta^‡H$ = 23 kJ mol^{-1}, $\Delta^‡S$ = -164 J K^{-1} mol^{-1}, $\Delta^‡G_{293K}$ = 71 kJ mol^{-1}. (**C**, **D**) Variable temperature kinetic profiles (10 – 40 °C; 10°C increments) for the aminolysis of N-acetyl pyrazole **4b$_{Ac}$** (0.10 M) with *p*-F-BnNH$_2$ **2** (0.10 M) and DBU **3** (0.10 M) in MeCN. Reactions assembled and monitored by VT-SF-^{19}F NMR spectroscopy, using 1-F-naphthalene as an internal integration standard (0.050 M). Product evolution profiles normalised according to the empirical rate law $k_{obs}^{(T)}$[**4b$_{Ac}$**][**2**][**3**]$_0$, assuming a constant base concentration of [**3**]$_0$ = 0.10 M. $k_{obs}^{(10°C)}$ = 0.77 M^{-2} s^{-1}, $k_{obs}^{(20°C)}$ = 0.86 M^{-2} s^{-1}, $k_{obs}^{(30°C)}$ = 0.92 M^{-2} s^{-1}, $k_{obs}^{(40°C)}$ = 0.95 M^{-2} s^{-1}. $\Delta^‡H$ = 3 kJ mol^{-1}, $\Delta^‡S$ = -237 J K^{-1} mol^{-1}, $\Delta^‡G_{293K}$ = 72 kJ mol^{-1}. $k° = k_{obs}^{(T)}.c°^2$, $c°$ = 1 M.

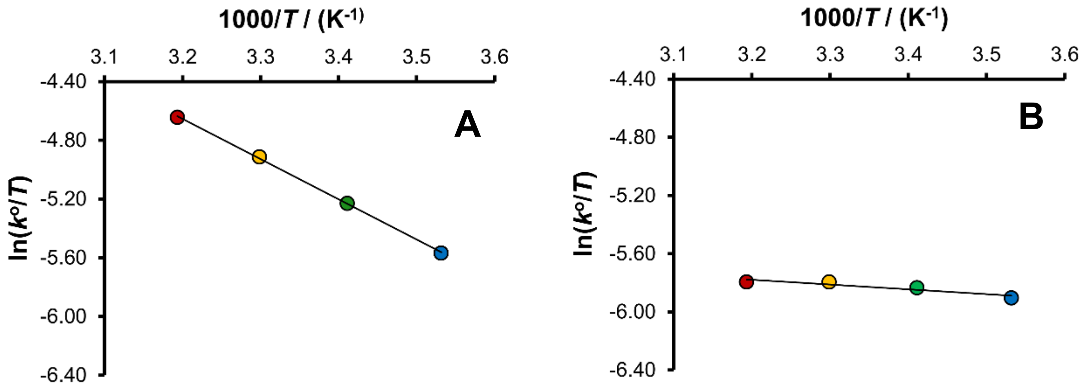

Figure S52: Eyring analyses of kinetic data in Figure S51. A) N-acetylation of pyrazole **$4b_H$** with *p*-F-PhAc **1** and DBU **3**. $k_{obs}^{(10°C)}$ = 1.09 M^{-2} s^{-1}, $k_{obs}^{(20°C)}$ = 1.57 M^{-2} s^{-1}, $k_{obs}^{(30°C)}$ = 2.22 M^{-2} s^{-1}, $k_{obs}^{(40°C)}$ = 3.01 M^{-2} s^{-1}. $\Delta^{‡}H$ = 23 kJ mol^{-1}, $\Delta^{‡}S$ = -164 J K^{-1} mol^{-1}, $\Delta^{‡}G_{293K}$ = 71 kJ mol^{-1}. B) Aminolysis of N-acetyl pyrazole **$4b_{Ac}$** (0.10 M) with *p*-F-BnNH$_2$ **2** (0.10 M) and DBU **3** (0.10 M) in MeCN-*h_3*. $k_{obs}^{(10°C)}$ = 0.77 M^{-2} s^{-1}, $k_{obs}^{(20°C)}$ = 0.86 M^{-2} s^{-1}, $k_{obs}^{(30°C)}$ = 0.92 M^{-2} s^{-1}, $k_{obs}^{(40°C)}$ = 0.95 M^{-2} s^{-1}. $\Delta^{‡}H$ = 3 kJ mol^{-1}, $\Delta^{‡}S$ = -237 J K^{-1} mol^{-1}, $\Delta^{‡}G_{293K}$ = 72 kJ mol^{-1}. $k° = k_{obs}^{(T)}.c°^{2}$, $c°$ = 1 M. Rate coefficients and activation parameters differ slightly from those obtained from catalytic data because of the different approach to analysis (numerical simulation and fitting for catalytic data; simple graphical analysis (i.e., VTNA) for stoichiometric data).

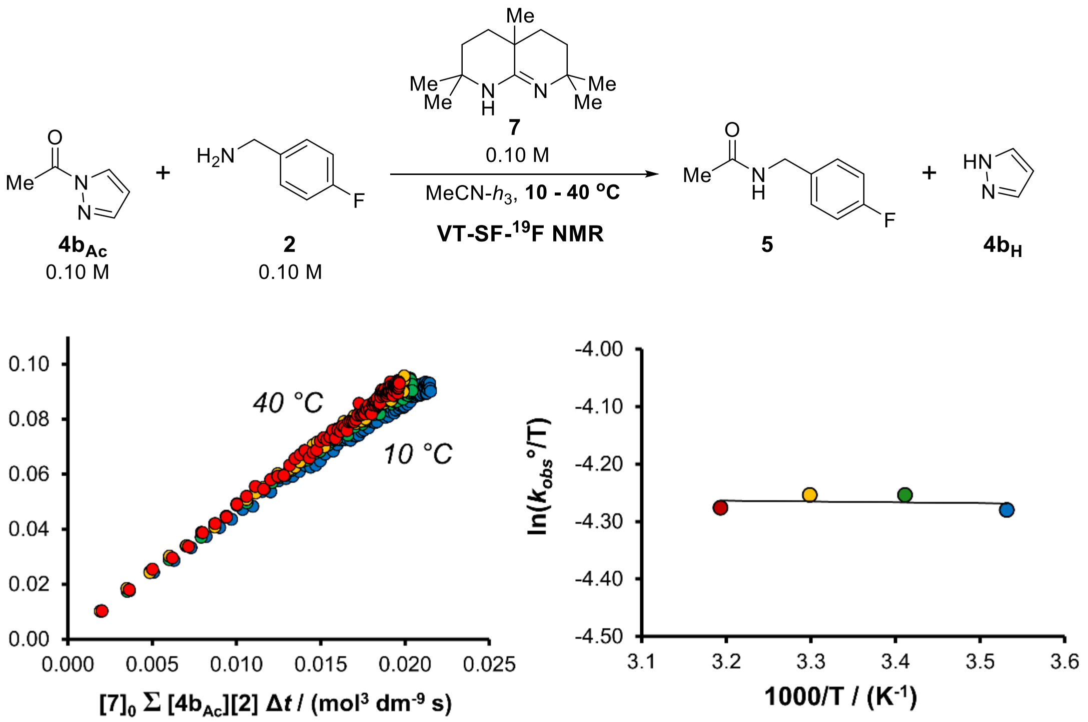

Figure S53: Variable temperature analysis (10 – 40 °C; 10°C increments) for the aminolysis of *N*-acetyl pyrazole **4b$_{Ac}$** (0.10 M) with *p*-F-BnNH$_2$ **2** (0.10 M) and PMDBD **7** (0.10 M) in MeCN. Reactions assembled and monitored by VT-SF-^{19}F NMR spectroscopy, with product evolution profiles normalised, as above, according to the empirical rate law $k_{obs}^{(T)}$[**4b$_{Ac}$**][**2**][**7**]$_0$, assuming a constant catalyst concentration of [**7**]$_0$ = 0.10 M. $k_{obs}^{(10°C)}$ = 3.92 M^{-2} s^{-1}, $k_{obs}^{(20°C)}$ = 4.17 M^{-2} s^{-1}, $k_{obs}^{(30°C)}$ = 4.31 M^{-2} s^{-1}, $k_{obs}^{(40°C)}$ = 4.35 M^{-2} s^{-1}. $\Delta^{\ddagger}H$ = 0 kJ mol^{-1}, $\Delta^{\ddagger}S$ = -233 J K^{-1} mol^{-1}, $\Delta^{\ddagger}G_{293K}$ = 68 kJ mol^{-1}. $k° = k_{obs}^{(T)}.c°^2$, $c°$ = 1 M.

S3.8.8 Structure-activity relationships: kinetic data

Tabulated data

Azole	$pK_a^{(MeCN)}$	$t_{1/2}$ / min	$\log_{10}(t_{1/2}$ / min)	$t_{1/2}$(ref)/ min
**4c**$_H$	22.2	1061[a]	3.03	258
**4d**$_H$	22.4	136[a]	2.13	-
**4e**$_H$	23.7	96	1.98	40
**4a**$_H$	24.6	8.4	0.92	8
**4f**$_H$	25.9	1.9	0.29	-
**4g**$_H$	26.9	1.8	0.25	-
**4h**$_H$	27.0	1.7	0.24	-
**4i**$_H$	27.7	2.2	0.35	-
**4j**$_H$	28.2	3.0	0.47	-
**4k**$_H$	28.7	5.2	0.72	-
**4l**$_H$	29.1*	25	1.40	540
**4m**$_H$	29.1	10	1.00	-
**4n**$_H$	29.0	9.2	0.96	-
**4o**$_H$	29.3	6.9	0.84	-
**4p**$_H$	29.5	18	1.25	-
**4q**$_H$	29.8	26	1.42	-
**4b**$_H$	30.1	27	1.44	132
**4r**$_H$	31.2	106[a]	2.02	-

Table S6: Phenomenological catalytic activities of various azoles (10 mol%) in the aminolysis of *p*-F-PhAc **1** (0.10 M) with *p*-F-BnNH$_2$ **2** (0.10 M) and DBU **3** (0.10 M) in MeCN at 20 °C. $t_{1/2}$ pertains to the time taken for the consumption of half the initial concentration of **1**. Reference half-lives taken from the literature.[S41] Acidities ($pK_a^{(MeCN)}$) obtained by calibrated KS-DFT/DLPNO-CCSD(T) computations, using pK_a(**4l**$_H$, MeCN) = 29.1 (imidazole *) as an anchor. [a]Half-lives determined by numerical fitting and extrapolation of kinetic profiles.

Azole	k'_1 / (M^{-2} s^{-1})	k_{-1} / (M^{-2} s^{-1})	k_2 / (M^{-2} s^{-1})	K'_1
**4f**$_H$	-	-	46	0.0070
**4g**$_H$	-	-	9.6	0.0880
**4h**$_H$	-	-	9.2	0.092
**4i**$_H$	-	-	6.9	0.14
**4j**$_H$	-	-	5.0	0.16
**4k**$_H$	13	49	2.6	0.26
**4l**$_H$	7.6	280	1.2	0.027
**4m**$_H$	8.9	43	1.6	0.21
**4n**$_H$	9.7	36	1.8	0.27
**4o**$_H$	8.1	35	1.7	0.24
**4p**$_H$	3.8	14	0.94	0.27
**4q**$_H$	3.7	13	0.48	0.27
**4b**$_H$	1.7	30	0.82	0.056
**4r**$_H$	0.57	5.7	0.18	0.10

Table S7: Experimental rate coefficients (k'_1, k_{-1}, k_2) and equilibrium constant (K'_1) for the catalysed aminolysis of *p*-F-PhAc **1** (0.10 M) with *p*-F-BnNH$_2$ **2** (0.10 M), DBU **3** (0.10 M) and class β azoles (10 mol%; pK_a(MeCN) > 25.9) in MeCN-d_3 at 20 °C. All constants extracted by numerical fitting of temporal concentration profiles for **1**, **2** and *p*-F-BnNHAc **5**, obtained by *in situ* ^{1}H NMR spectroscopy, to the telescoped kinetic model in the main text (Figure 6; Scheme S3). Competing hydrolysis was generally very minor for all azoles, and accounted for during numerical fitting by inclusion of a formally termolecular, DBU-catalysed reaction of adventitious water with the acetylated

intermediate $\mathbf{4_{Ac}}$. Absent entries for k'_1 and k_{-1} indicative of a rapid pre-equilibrium between **1** and the corresponding acetylated azole $\mathbf{4_{Ac}}$; in such cases only the equilibrium constant K'_1 could be determined with any significance.

Azole	$\Delta^{\ddagger}G'^{(1)}_{293K}$ (kJ mol^{-1})	$\Delta^{\ddagger}G^{(-1)}_{293K}$ (kJ mol^{-1})	$\Delta^{\ddagger}G^{(2)}_{293K}$ (kJ mol^{-1})	$\Delta G'^{(1)}_{293K}$ (kJ mol^{-1})
$\mathbf{4f_H}$	-	-	62	12
$\mathbf{4g_H}$	-	-	66	5.9
$\mathbf{4h_H}$	-	-	66	5.8
$\mathbf{4i_H}$	-	-	67	4.8
$\mathbf{4j_H}$	-	-	68	4.5
$\mathbf{4k_H}$	66	62	69	3.3
$\mathbf{4l_H}$	67	58	71	8.8
$\mathbf{4m_H}$	66	63	71	3.8
$\mathbf{4n_H}$	66	63	70	3.2
$\mathbf{4o_H}$	67	63	70	3.5
$\mathbf{4p_H}$	68	65	72	3.2
$\mathbf{4q_H}$	69	65	74	3.2
$\mathbf{4b_H}$	70	63	72	7.0
$\mathbf{4r_H}$	73	68	76	5.6

Table S8: Experimental free energies of activation ($\Delta^{\ddagger}G'^{(1)}_{293K}$, $\Delta^{\ddagger}G^{(-1)}_{293K}$, $\Delta^{\ddagger}G^{(2)}_{293K}$) and free energy of intermediate formation ($\Delta G'^{(1)}_{293K}$) for the catalytic aminolysis of *p*-F-PhAc **1** with *p*-F-BnNH$_2$ **2**, DBU **3** and various azoles (10 mol%) in MeCN-d_3 at 20 °C, calculated in accordance with rate coefficients extracted by numerical fitting **(**Table S7**)** of experimental data to telescoped kinetic model in Scheme S3. $\Delta G'^{(1)}_{293K} = -RT\ln(K'_1)$). $\Delta^{\ddagger}G^{(i)}_{293K} = -RT\ln(hk_i c^{\circ 2}/k_B T)$, c° = 1 M.

Azole	K_1k_2 / (M^{-1} s^{-1})	($\Delta G^{(1)}_{293K} + \Delta^{\ddagger}G^{(2)}_{293K}$) / (kJ mol^{-1})
$\mathbf{4c_H}$	2.84×10^{-5}	97
$\mathbf{4d_H}$	2.05×10^{-4}	92
$\mathbf{4e_H}$	3.12×10^{-4}	91
$\mathbf{4a_H}$	4.04×10^{-3}	85

Table S9: Experimental rate coefficients (K_1k_2) for the catalysed aminolysis of *p*-F-PhAc **1** (0.10 M) with *p*-F-BnNH$_2$ **2** (0.10 M), DBU **3** (0.10 M) and various class α azoles (10 mol%; pK_a(MeCN) < 25) in MeCN-d_3 at T = 20 °C. Nominal values for the underlying coefficients (k_1, k_{-1}, k_2) were determined by numerical fitting of kinetic profiles of **1**, **2** and **5** in each run to the telescoped kinetic model in Scheme S4, with the rate coefficient for reverse phenolysis fixed to an arbitrarily large value of $k_{-1} = 10^7$ M^{-2} s^{-1}. Given the absence of any detectable intermediate $\mathbf{4_{Ac}}$, the fitted values of k_1 and k_2 are highly correlated and thus insignificant in their own right; however, fitted values for the product K_1k_2 were found to be robust. $\{\Delta G^{(1)}_{293K} + \Delta^{\ddagger}G^{(2)}_{293K}\} = -RT\ln(hK_1k_2c^{\circ}/k_BT)$, c° = 1 M.

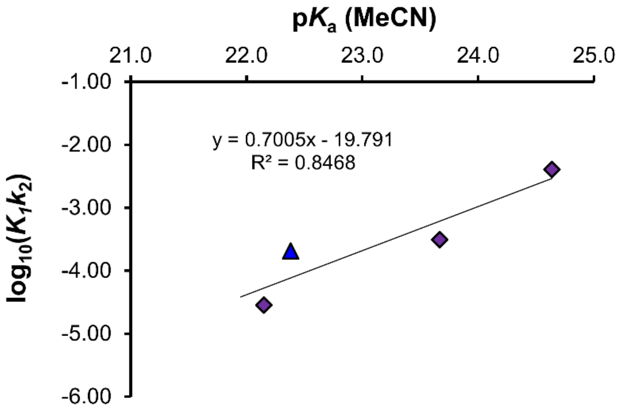

Figure S54. Structure-reactivity relationship between azole acidity (pK_a(MeCN)) and catalytic activity ($\log_{10}(K_1k_2)$) for the azole-catalysed (10 mol%) aminolysis of **1** (0.10 M) with **2** (0.10 M), **3** (0.10 M) and class α azoles (pK_a(MeCN) < 25) in MeCN (20 °C). Homoconjugation of **6**$_H$ assumed to be unaffected by azole identity. Purple diamonds: triazoles (**4a**$_H$, **4c**$_H$, **4e**$_H$). Blue triangle: 4-nitropyrazole (**4d**$_H$).

Numerical kinetic fits: ***4c*** $_{\mathbf{H}}$

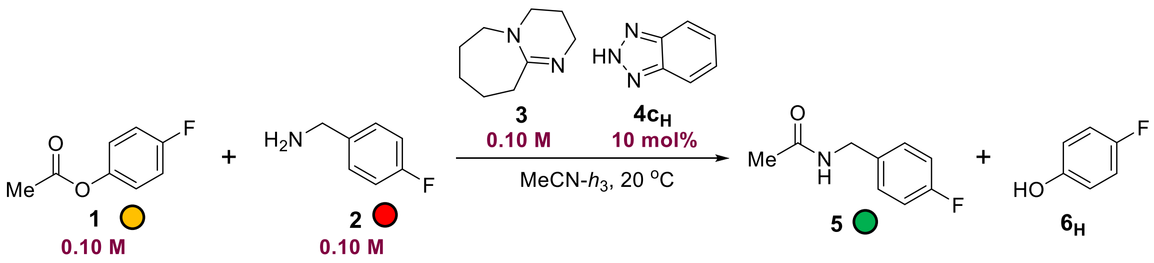

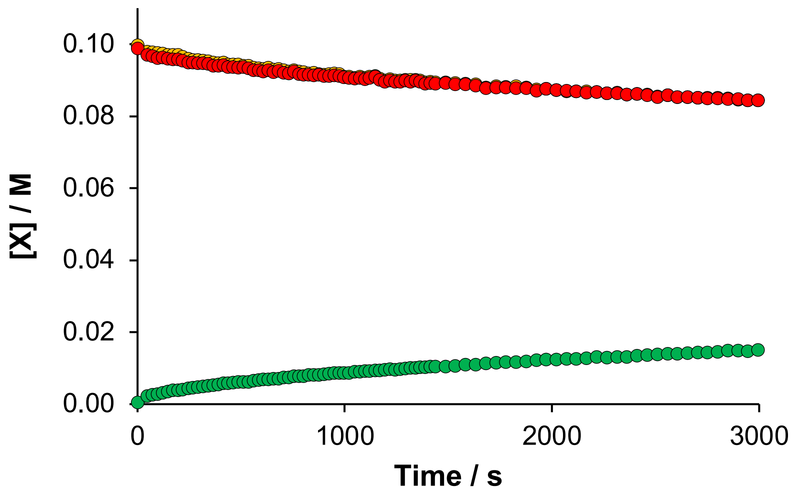

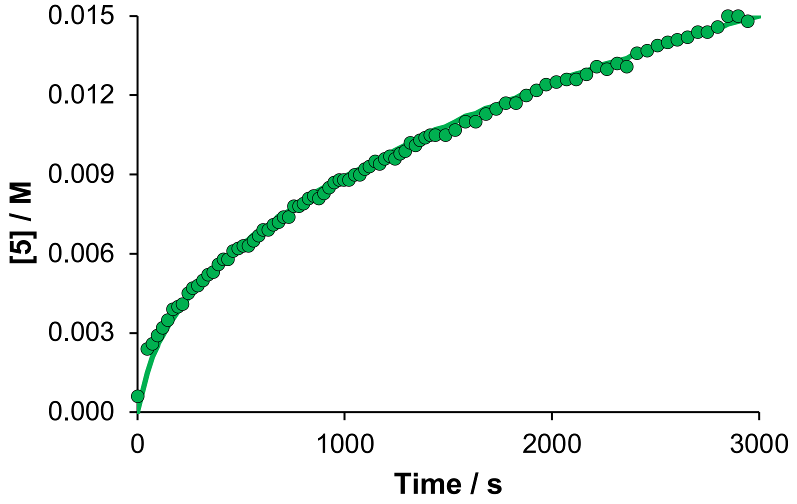

Figure S55: Experimental concentration-time profiles (points) for **1**, **2**, and **5** under standard catalytic conditions in MeCN ([**1**]$_0$ = 0.10 M, [**2**]$_0$ = 0.10 M, [**3**]$_0$ = 0.10 M, [**4c$_H$**]$_0$ = 0.010 M; MeCN, 20 °C), and simulated profiles (lines) calculated from kinetic parameters obtained by numerical fitting of [**1**], [**2**], and [**5**] to the telescoped kinetic model shown in Scheme S4. Profiles obtained by *in situ* ^{19}F NMR monitoring. k_{-1} = 10^7 M^{-2} s^{-1} assumed arbitrarily.

*Numerical kinetic fits: **4dH***

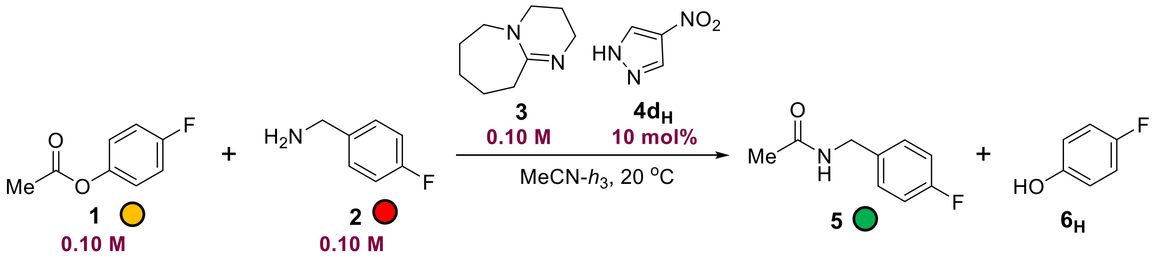

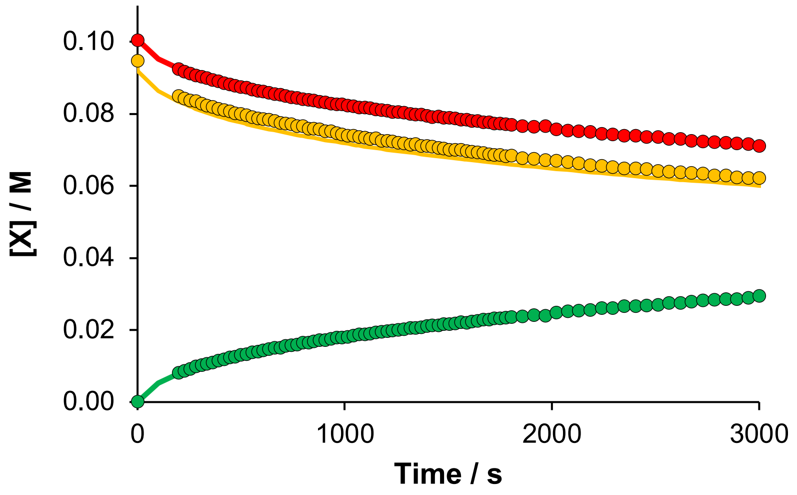

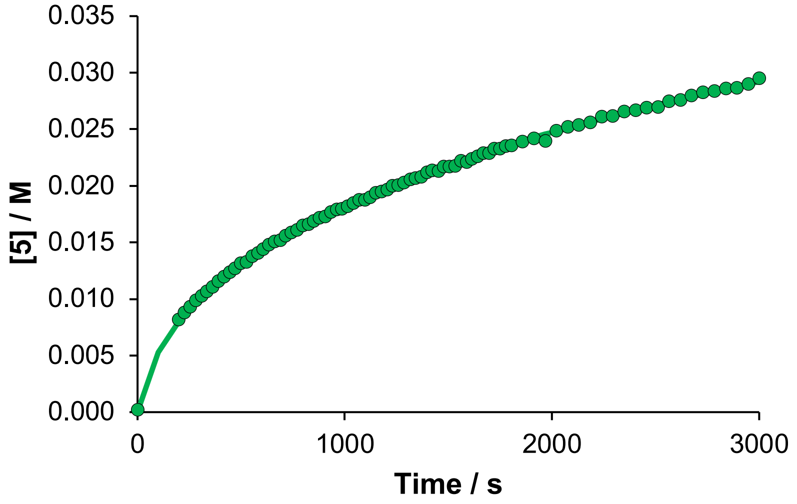

Figure S56: Experimental concentration-time profiles (points) for **1**, **2**, and **5** under standard catalytic conditions in MeCN ([**1**]$_0$ = 0.10 M, [**2**]$_0$ = 0.10 M, [**3**]$_0$ = 0.10 M, [**4d$_H$**]$_0$ = 0.010 M; MeCN, 20 °C), and simulated profiles (lines) calculated from kinetic parameters obtained by numerical fitting of [**1**], [**2**], and [**5**] to the telescoped kinetic model shown in Scheme S4. Profiles obtained by *in situ* ^{19}F NMR monitoring. k_{-1} = 10^7 M^{-2} s^{-1} assumed arbitrarily.

*Numerical kinetic fits: **4e**$_H$*

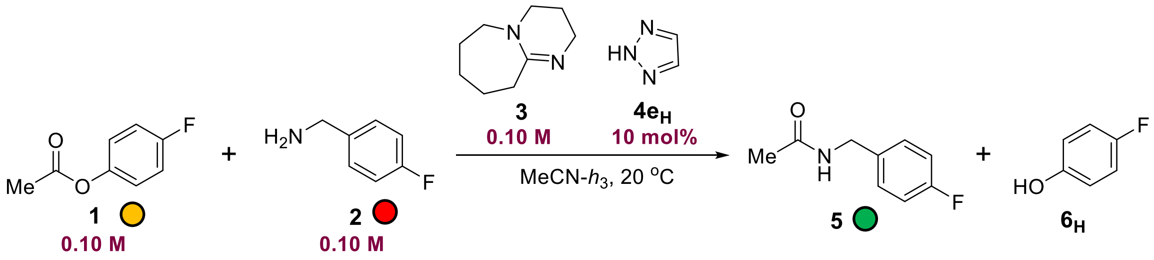

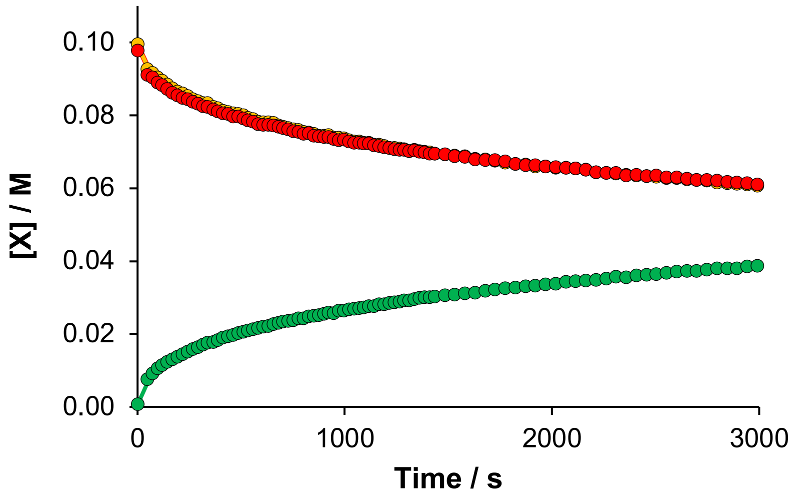

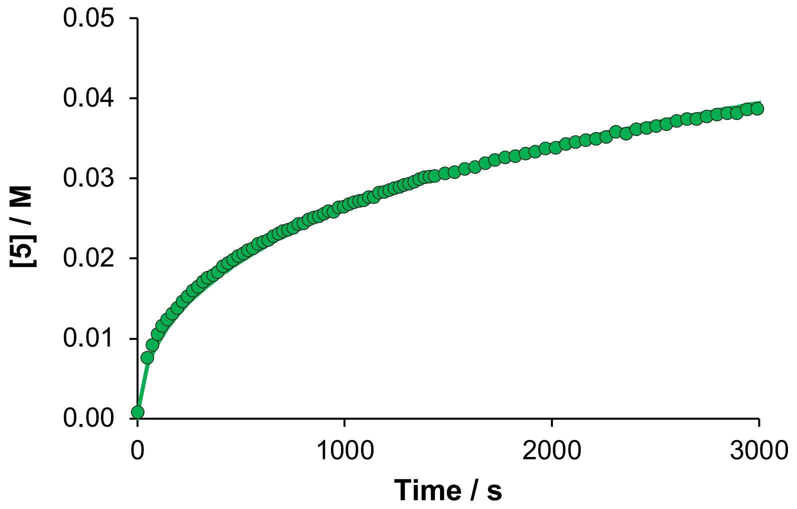

Figure S57: Experimental concentration-time profiles (points) for **1**, **2**, and **5** under standard catalytic conditions in MeCN ([**1**]$_0$ = 0.10 M, [**2**]$_0$ = 0.10 M, [**3**]$_0$ = 0.10 M, [**4e**$_H$]$_0$ = 0.010 M; MeCN, 20 °C), and simulated profiles (lines) calculated from kinetic parameters obtained by numerical fitting of [**1**], [**2**], and [**5**] to the telescoped kinetic model shown in Scheme S4. Profiles obtained by *in situ* ^{19}F NMR monitoring. k_{-1} = 10^7 M^{-2} s^{-1} assumed arbitrarily.

*Numerical kinetic fits: **4a**H*

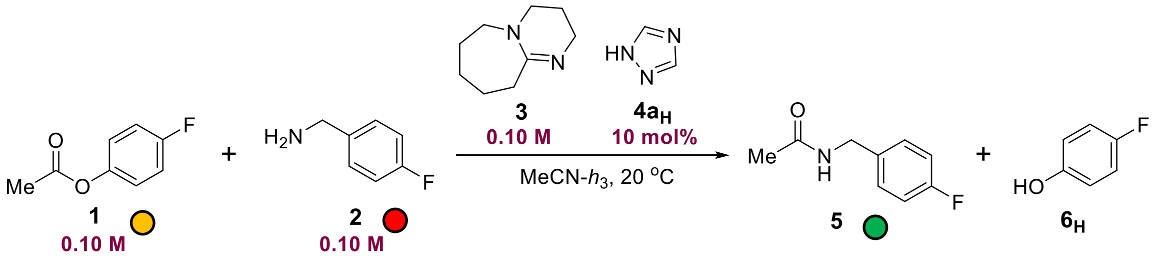

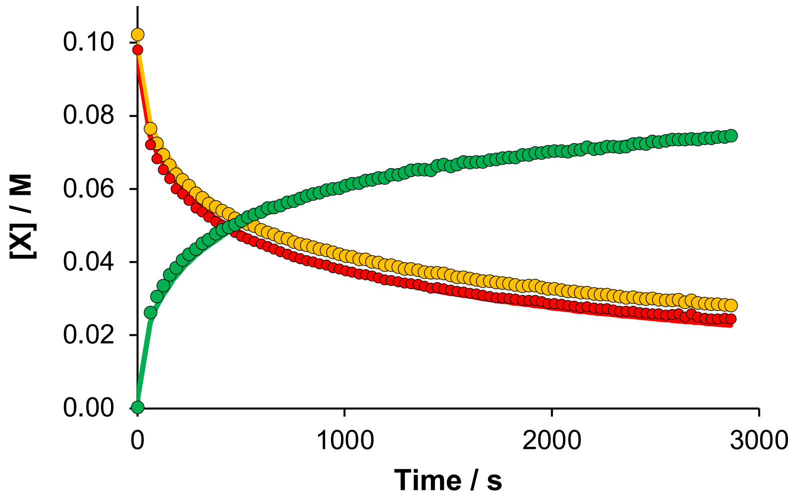

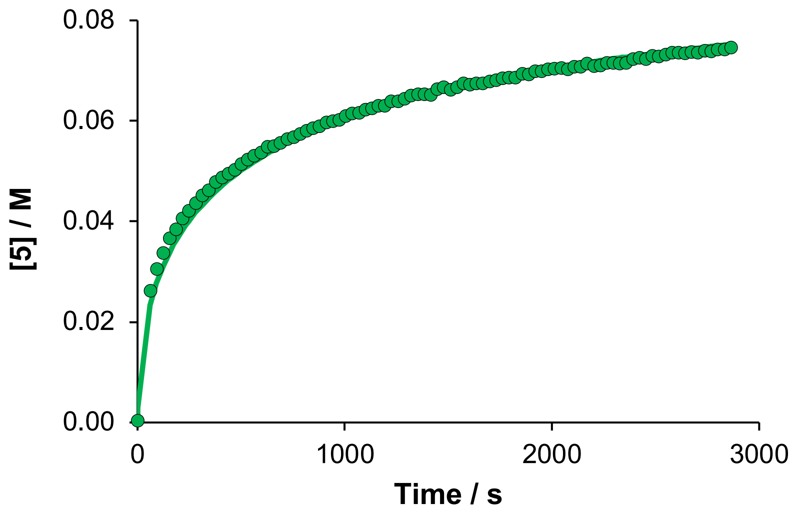

Figure S58: Experimental concentration-time profiles (points) for **1**, **2**, and **5** under standard catalytic conditions in MeCN ([**1**]$_0$ = 0.10 M, [**2**]$_0$ = 0.10 M, [**3**]$_0$ = 0.10 M, [**4a$_H$**]$_0$ = 0.010 M; MeCN, 20 °C), and simulated profiles (lines) calculated from kinetic parameters obtained by numerical fitting of [**1**], [**2**], and [**5**] to the telescoped kinetic model shown in Scheme S4. Profiles obtained by *in situ* ^{19}F NMR monitoring. k_{-1} = 10^7 M^{-2} s^{-1} assumed arbitrarily.

*Numerical kinetic fits: **4f**H*

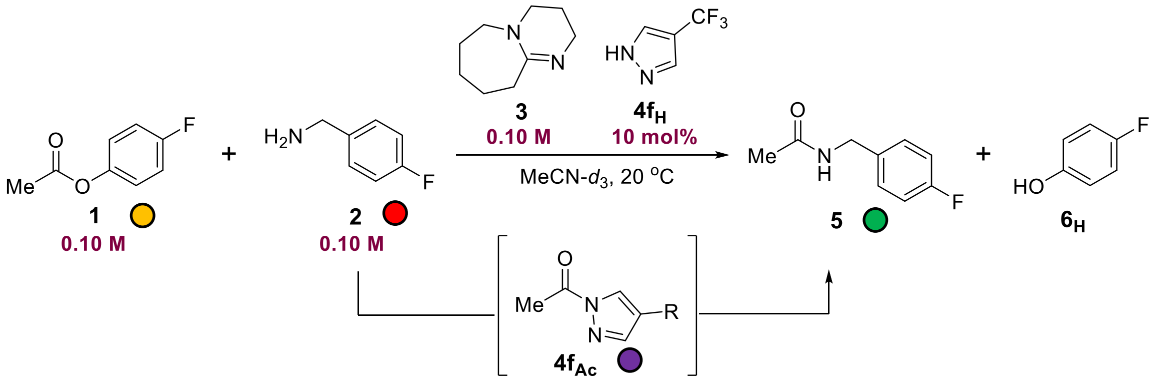

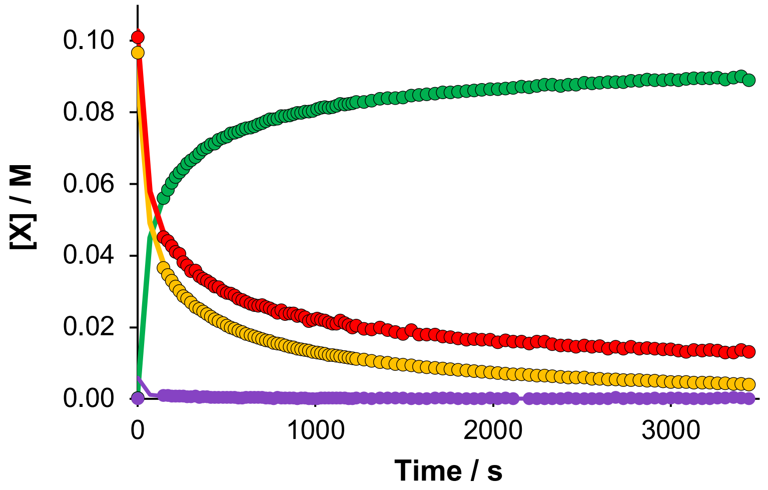

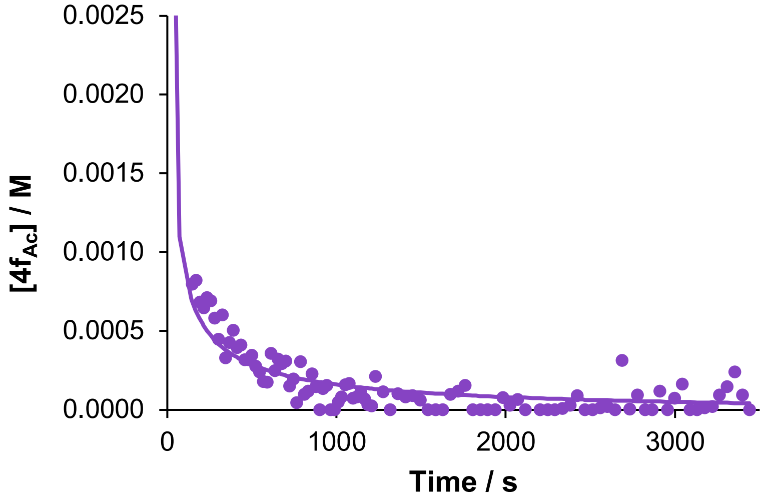

Figure S59: Experimental concentration-time profiles (points) for **1**, **2**, **5** and **4f$_{Ac}$** under standard catalytic conditions in MeCN ([**1**]$_0$ = 0.10 M, [**2**]$_0$ = 0.10 M, [**3**]$_0$ = 0.10 M, [**4f$_H$**]$_0$ = 0.010 M; MeCN-d_3, 20 °C), and simulated profiles (lines) calculated from kinetic parameters obtained by numerical fitting of [**1**], [**2**], and [**5**] to the telescoped kinetic model shown in Scheme S3; [**4f$_{Ac}$**] not included in fitting. Profiles obtained by *in situ* ^{1}H NMR monitoring.

Numerical kinetic fits: 4g$_H$

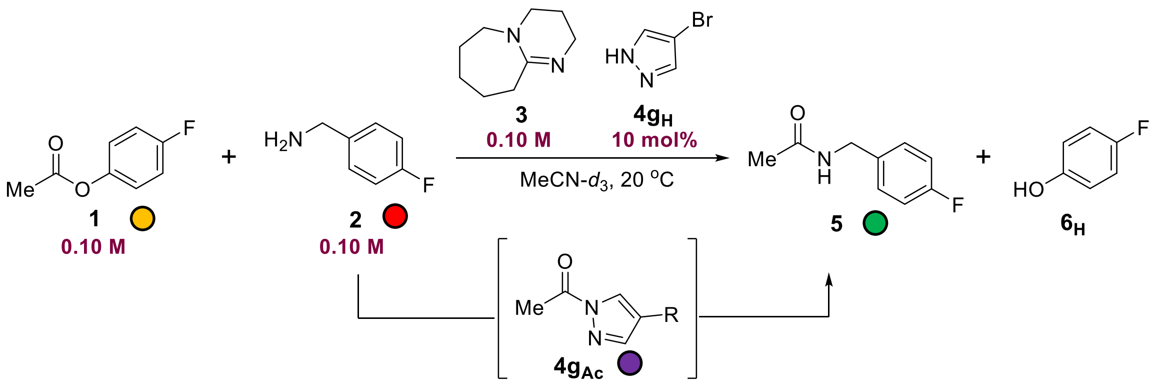

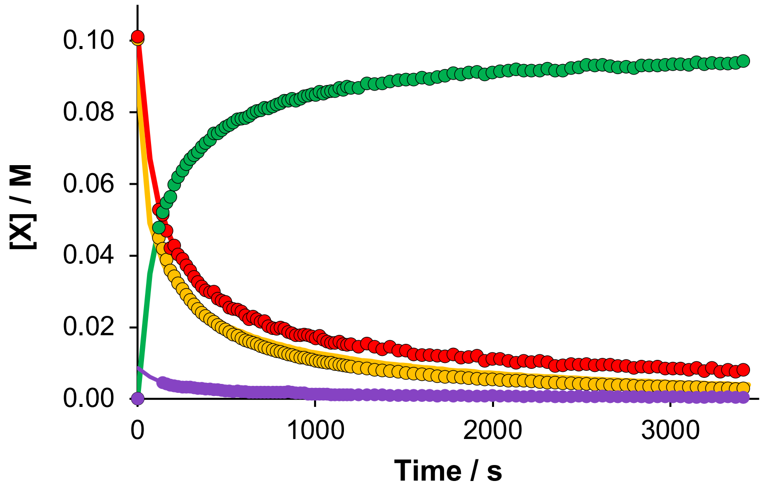

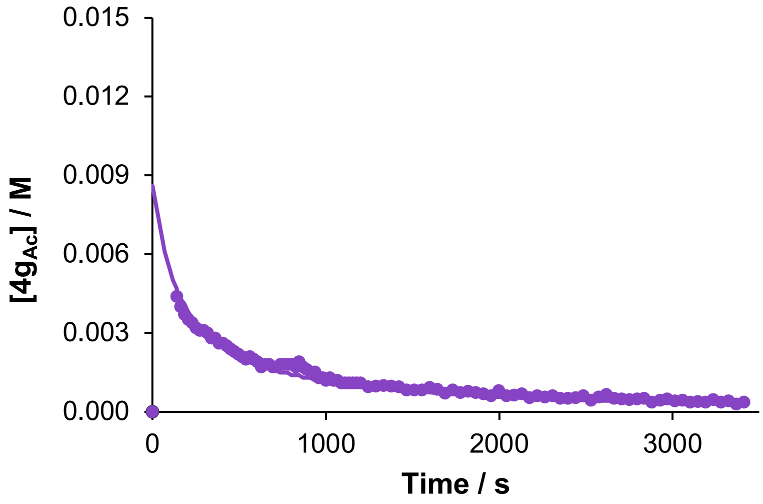

Figure S60: Experimental concentration-time profiles (points) for **1**, **2**, **5** and **4g$_{Ac}$** under standard catalytic conditions in MeCN ([**1**]$_0$ = 0.10 M, [**2**]$_0$ = 0.10 M, [**3**]$_0$ = 0.10 M, [**4g$_H$**]$_0$ = 0.010 M; MeCN-d_3, 20 °C), and simulated profiles (lines) calculated from kinetic parameters obtained by numerical fitting of [**1**], [**2**], and [**5**] to the telescoped kinetic model shown in Scheme S3; [**4g$_{Ac}$**] not included in fitting. Profiles obtained by *in situ* ^{1}H NMR monitoring.

*Numerical kinetic fits: **4h**$_H$*

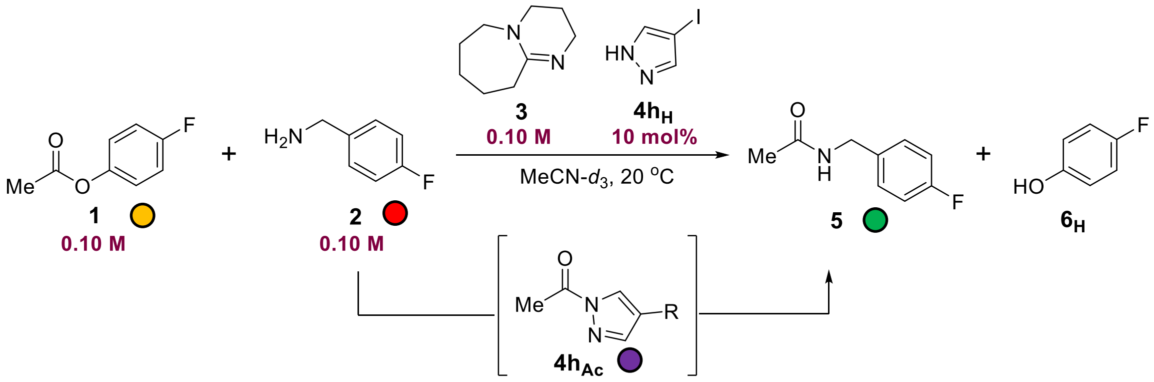

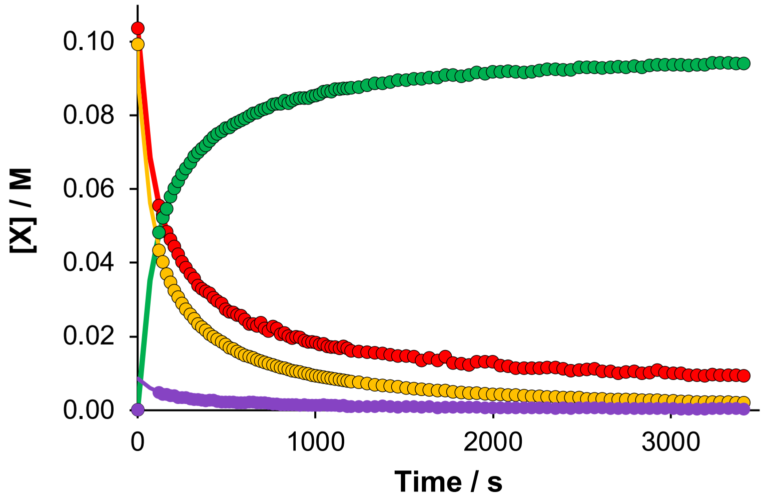

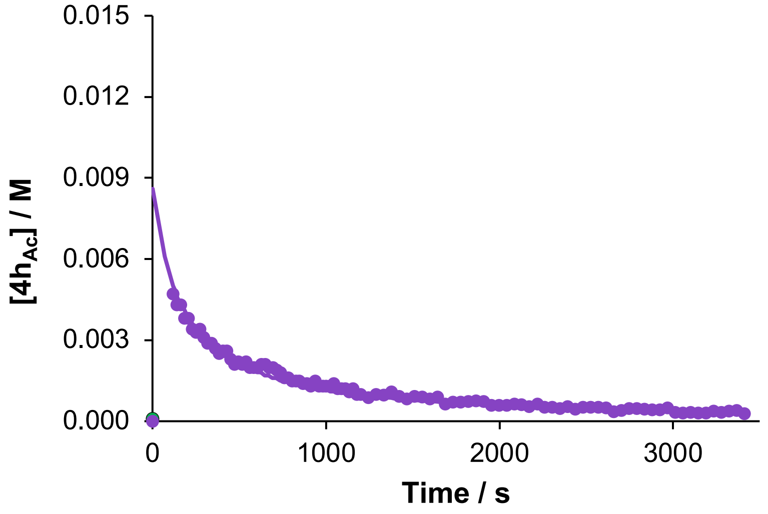

Figure S61: Experimental concentration-time profiles (points) for **1**, **2**, **5** and **4h**$_{Ac}$ under standard catalytic conditions in MeCN ([**1**]$_0$ = 0.10 M, [**2**]$_0$ = 0.10 M, [**3**]$_0$ = 0.10 M, [**4h**$_H$]$_0$ = 0.010 M; MeCN-d_3, 20 °C), and simulated profiles (lines) calculated from kinetic parameters obtained by numerical fitting of [**1**], [**2**], and [**5**] to the telescoped kinetic model shown in Scheme S3**;** [**4h**$_{Ac}$] not included in fitting. Profiles obtained by *in situ* ^{1}H NMR monitoring.

*Numerical kinetic fits: **4i**$_H$*

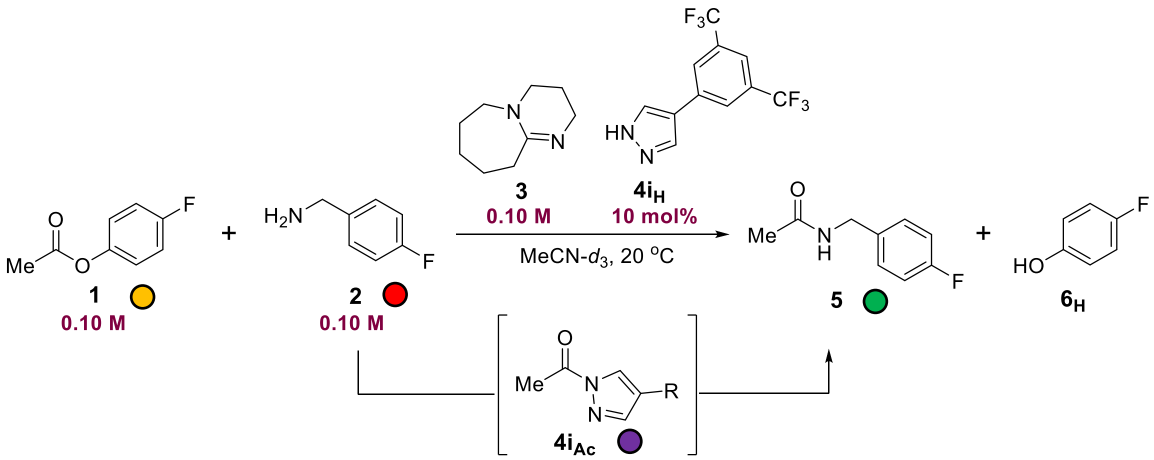

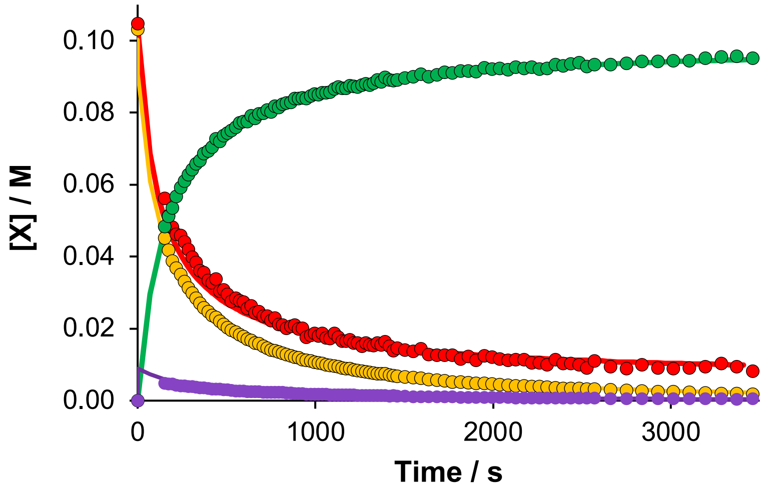

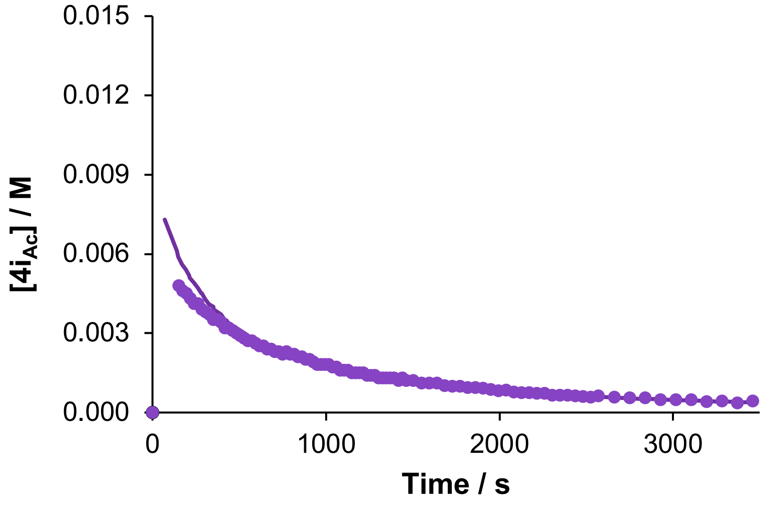

Figure S62: Experimental concentration-time profiles (points) for **1**, **2**, **5** and **4i**$_{Ac}$ under standard catalytic conditions in MeCN ([**1**]$_0$ = 0.10 M, [**2**]$_0$ = 0.10 M, [**3**]$_0$ = 0.10 M, [**4i**$_H$]$_0$ = 0.010 M; MeCN-d_3, 20 °C), and simulated profiles (lines) calculated from kinetic parameters obtained by numerical fitting of [**1**], [**2**], and [**5**] to the telescoped kinetic model shown in Scheme S3**;** [**4i**$_{Ac}$] not included in fitting. Profiles obtained by *in situ* ^{1}H NMR monitoring.

*Numerical kinetic fits: **4j$_H$***

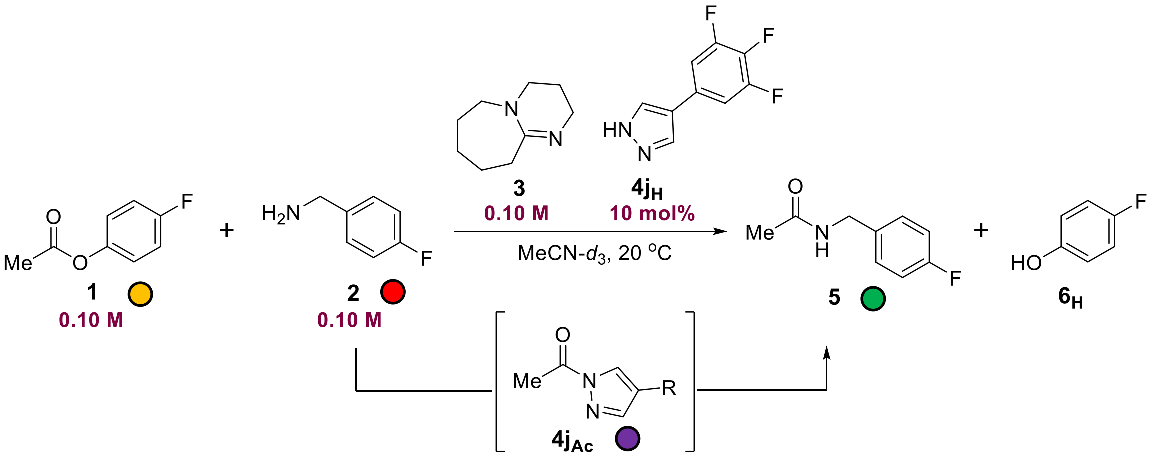

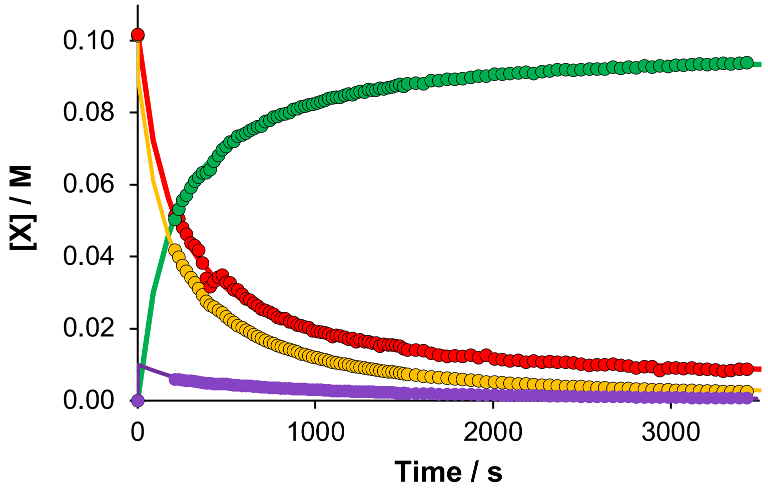

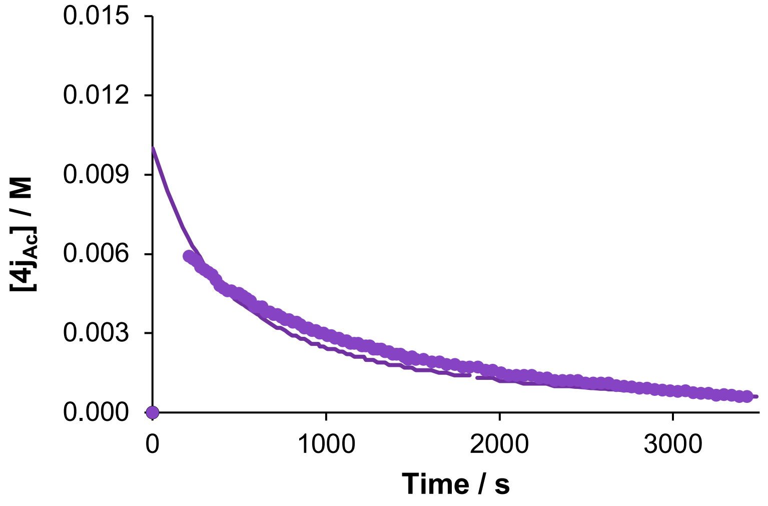

Figure S63: Experimental concentration-time profiles (points) for **1**, **2**, **5** and **4j$_{Ac}$** under standard catalytic conditions in MeCN ([**1**]$_0$ = 0.10 M, [**2**]$_0$ = 0.10 M, [**3**]$_0$ = 0.10 M, [**4j$_H$**]$_0$ = 0.010 M; MeCN-d_3, 20 °C), and simulated profiles (lines) calculated from kinetic parameters obtained by numerical fitting of [**1**], [**2**], and [**5**] to the telescoped kinetic model shown in Scheme S3**;** [**4j$_{Ac}$**] not included in fitting. Profiles obtained by *in situ* ^{1}H NMR monitoring.

*Numerical kinetic fits: **4k***

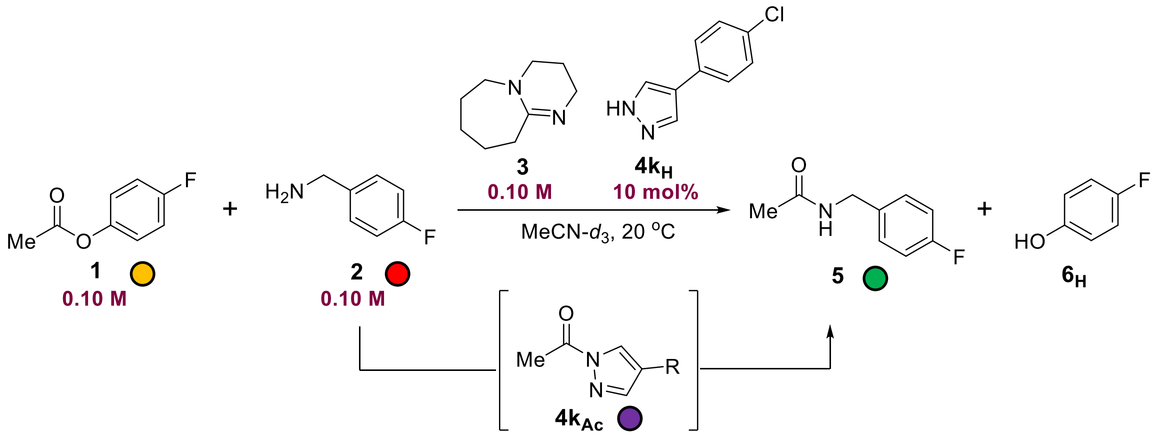

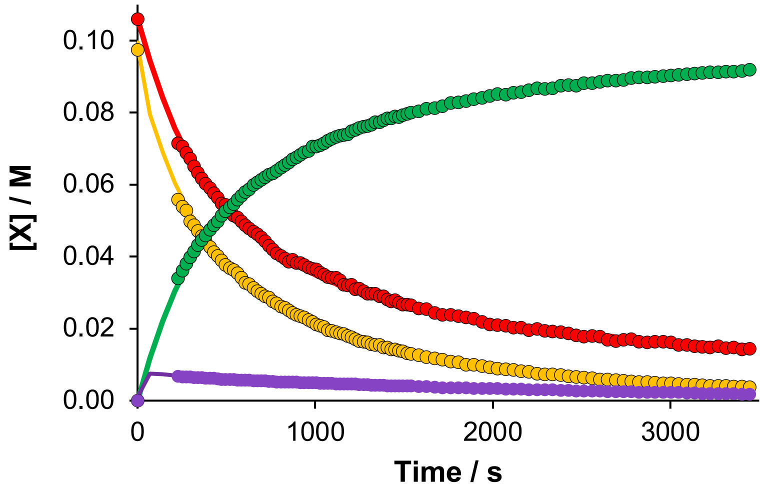

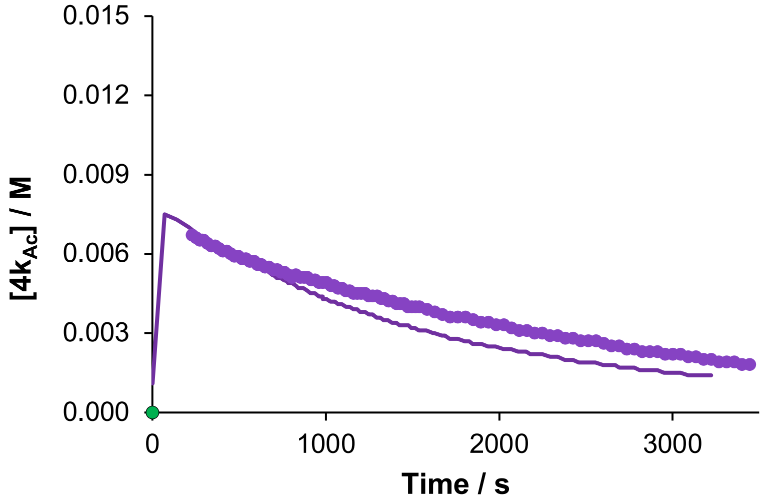

Figure S64: Experimental concentration-time profiles (points) for **1**, **2**, **5** and **4k**$_{\mathbf{Ac}}$ under standard catalytic conditions in MeCN ([**1**]$_0$ = 0.10 M, [**2**]$_0$ = 0.10 M, [**3**]$_0$ = 0.10 M, [**4k**$_{\mathbf{H}}$]$_0$ = 0.010 M; MeCN-*d*$_3$, 20 °C), and simulated profiles (lines) calculated from kinetic parameters obtained by numerical fitting of [**1**], [**2**], and [**5**] to the telescoped kinetic model shown in Scheme S3**;** [**4k**$_{\mathbf{Ac}}$] not included in fitting. Profiles obtained by *in situ* ^{1}H NMR monitoring.

*Numerical kinetic fits: **4l**$_H$*

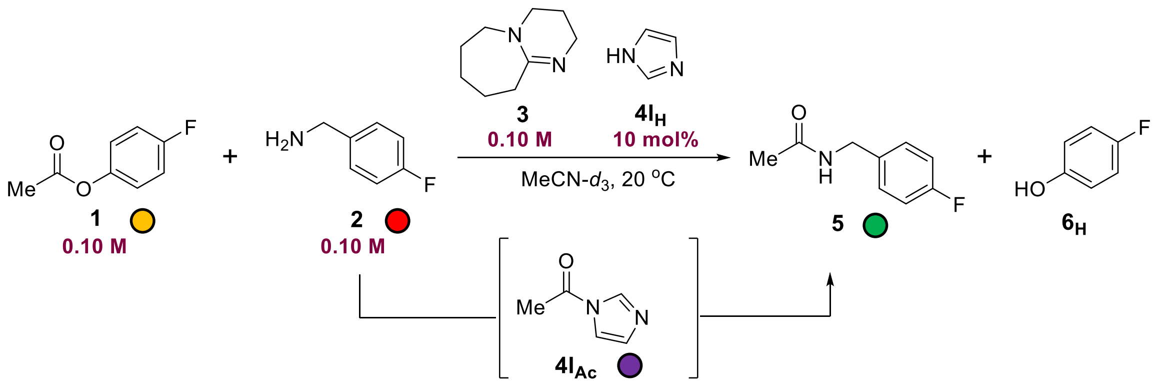

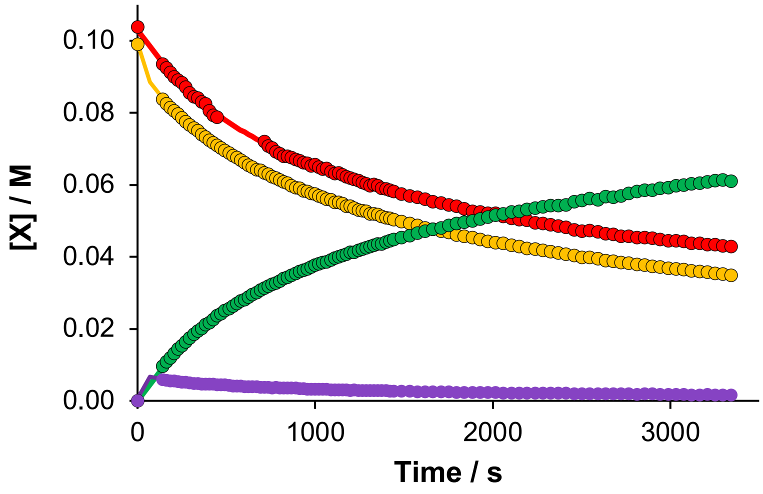

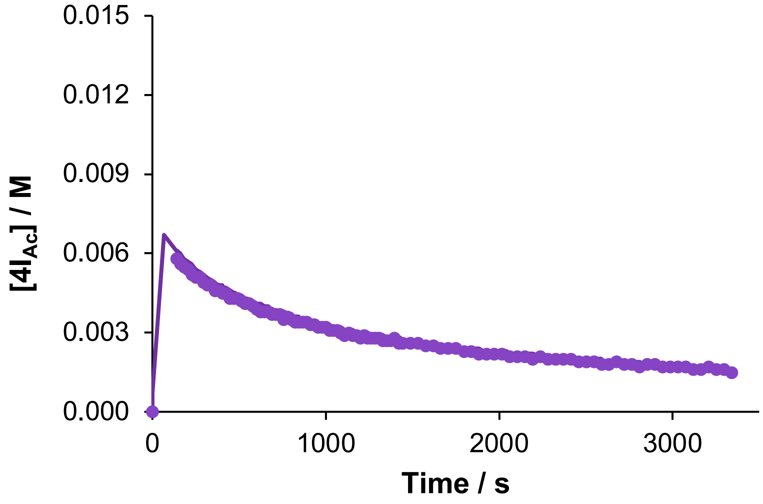

Figure S65: Experimental concentration-time profiles (points) for **1**, **2**, **5** and **4l**$_{Ac}$ under standard catalytic conditions in MeCN ([**1**]$_0$ = 0.10 M, [**2**]$_0$ = 0.10 M, [**3**]$_0$ = 0.10 M, [**4l**$_H$]$_0$ = 0.010 M; MeCN-d_3, 20 °C), and simulated profiles (lines) calculated from kinetic parameters obtained by numerical fitting of [**1**], [**2**], and [**5**] to the telescoped kinetic model shown in Scheme S3**;** [**4l**$_{Ac}$] not included in fitting. Profiles obtained by *in situ* ^{1}H NMR monitoring.

Numerical kinetic fits: **4m$_H$**

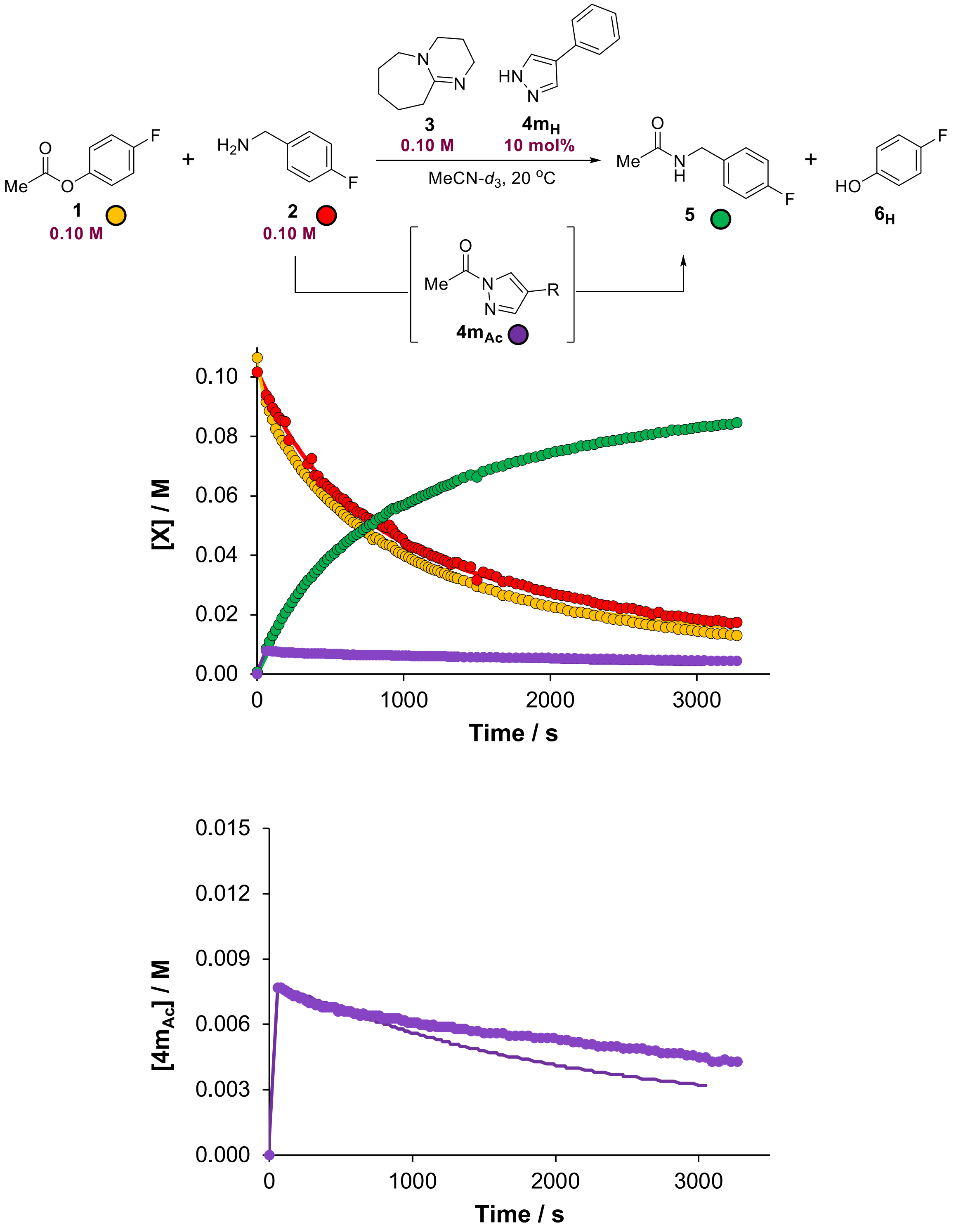

Figure S66: Experimental concentration-time profiles (points) for **1**, **2**, **5** and **4m$_{Ac}$** under standard catalytic conditions in MeCN ([**1**]$_0$ = 0.10 M, [**2**]$_0$ = 0.10 M, [**3**]$_0$ = 0.10 M, [**4m$_H$**]$_0$ = 0.010 M; MeCN-d_3, 20 °C), and simulated profiles (lines) calculated from kinetic parameters obtained by numerical fitting of [**1**], [**2**], and [**5**] to the telescoped kinetic model shown in Scheme S3**;** [**4m$_{Ac}$**] not included in fitting. Profiles obtained by *in situ* ^{1}H NMR monitoring.

Numerical kinetic fits: **$4n_H$**

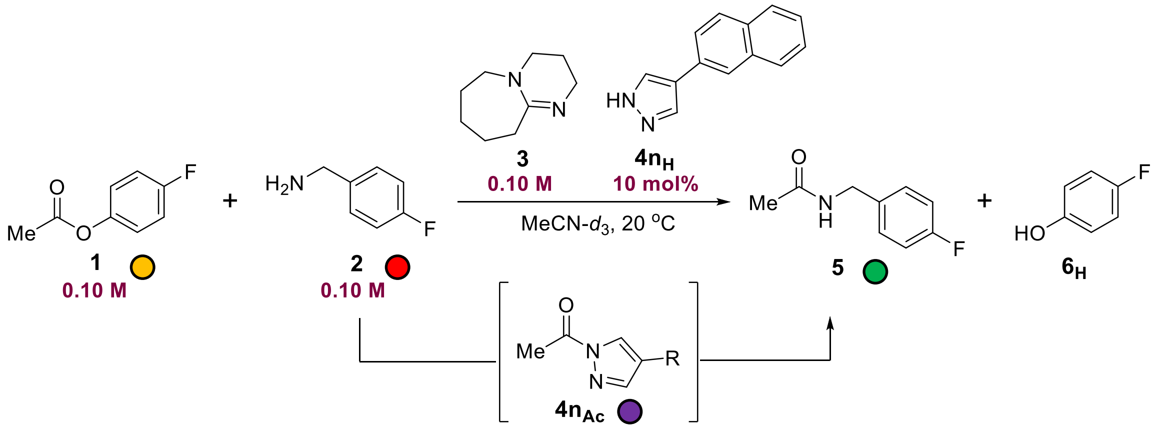

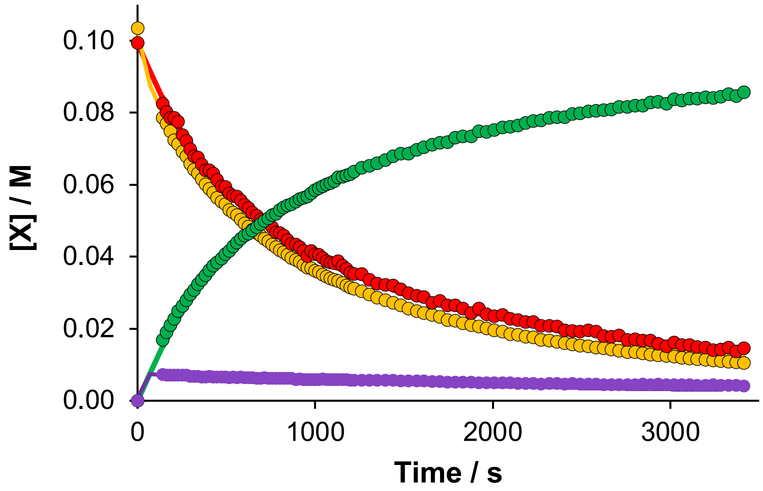

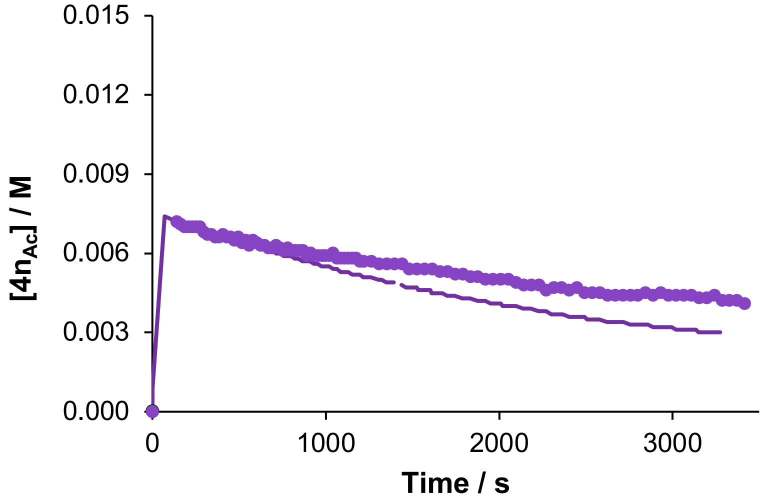

Figure S67: Experimental concentration-time profiles (points) for **1**, **2**, **5** and **$4n_{Ac}$** under standard catalytic conditions in MeCN ([**1**]$_0$ = 0.10 M, [**2**]$_0$ = 0.10 M, [**3**]$_0$ = 0.10 M, [**$4n_H$**]$_0$ = 0.010 M; MeCN-d_3, 20 °C), and simulated profiles (lines) calculated from kinetic parameters obtained by numerical fitting of [**1**], [**2**], and [**5**] to the telescoped kinetic model shown in Scheme S3**;** [**$4n_{Ac}$**] not included in fitting. Profiles obtained by *in situ* ^{1}H NMR monitoring.

Numerical kinetic fits: **4o_H**

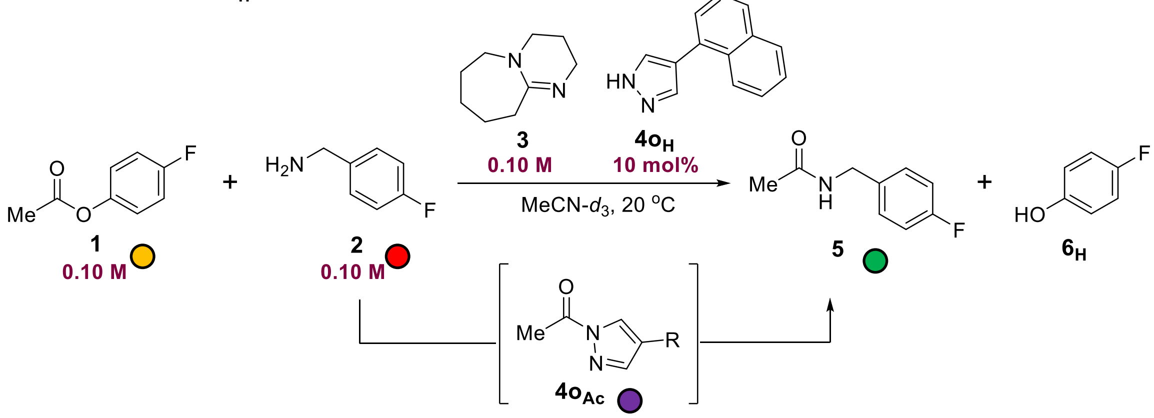

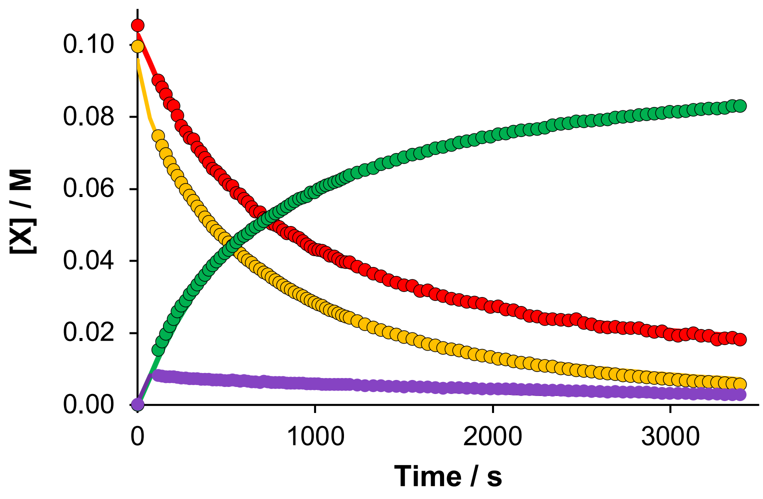

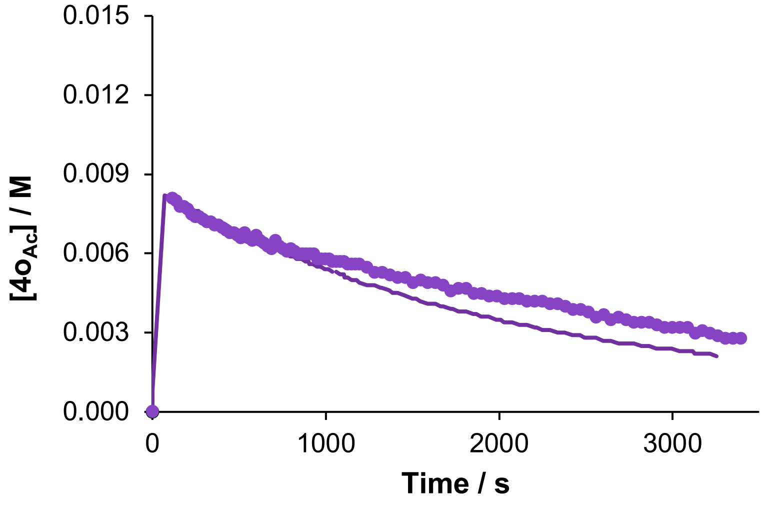

Figure S68: Experimental concentration-time profiles (points) for **1**, **2**, **5** and **4o_Ac** under standard catalytic conditions in MeCN ([**1**]$_0$ = 0.10 M, [**2**]$_0$ = 0.10 M, [**3**]$_0$ = 0.10 M, [**4o_H**]$_0$ = 0.010 M; MeCN-d_3, 20 °C), and simulated profiles (lines) calculated from kinetic parameters obtained by numerical fitting of [**1**], [**2**], and [**5**] to the telescoped kinetic model shown in Scheme S3**;** [**4o_Ac**] not included in fitting. Profiles obtained by *in situ* ^{1}H NMR monitoring.

Numerical kinetic fits: **4p$_H$**

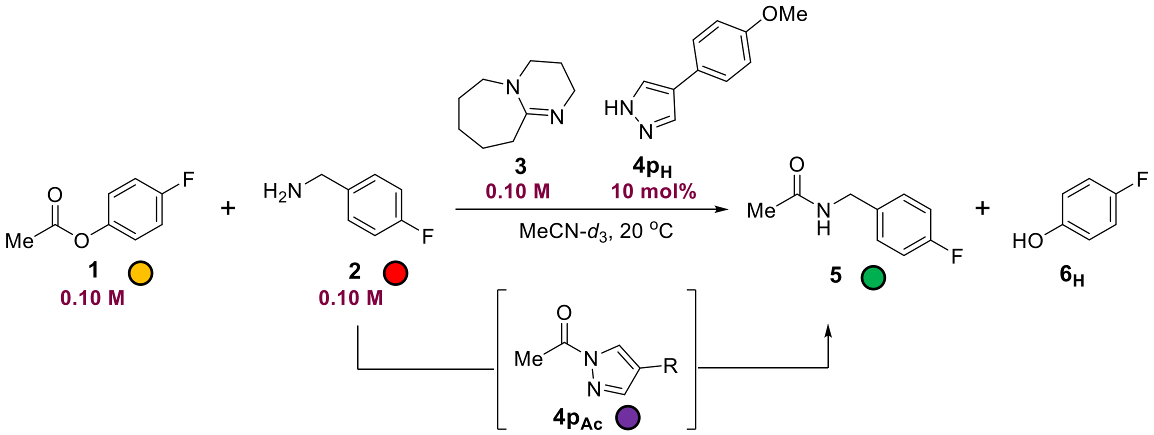

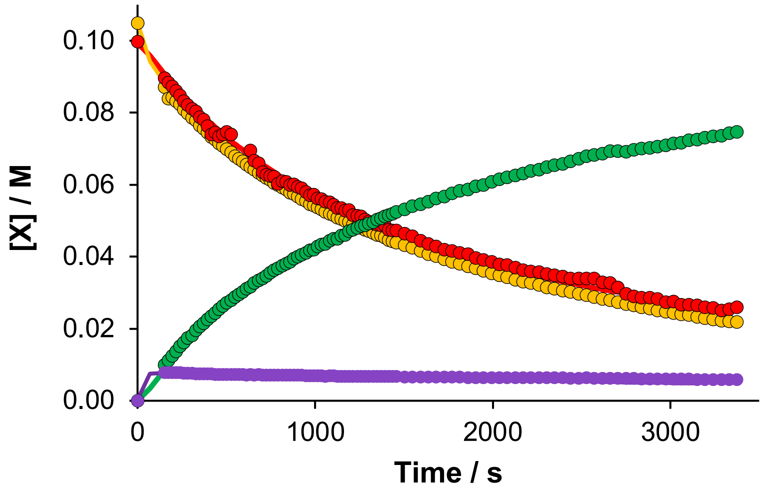

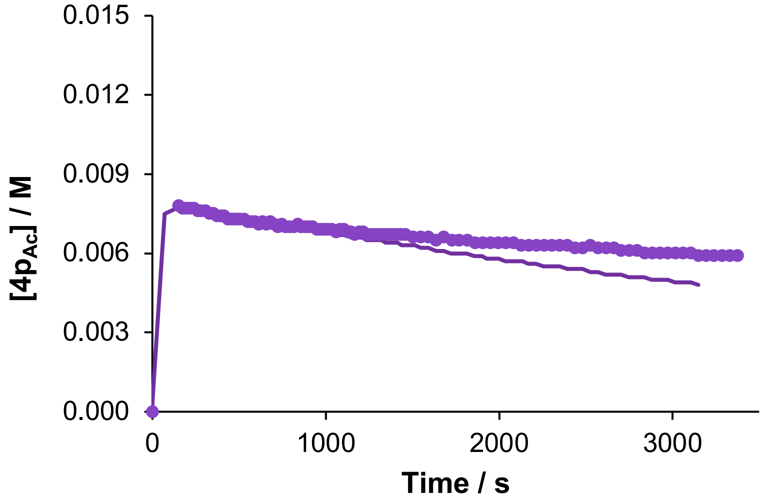

Figure S69: Experimental concentration-time profiles (points) for **1**, **2**, **5** and **4p$_{Ac}$** under standard catalytic conditions in MeCN ([**1**]$_0$ = 0.10 M, [**2**]$_0$ = 0.10 M, [**3**]$_0$ = 0.10 M, [**4p$_H$**]$_0$ = 0.010 M; MeCN-d_3, 20 °C), and simulated profiles (lines) calculated from kinetic parameters obtained by numerical fitting of [**1**], [**2**], and [**5**] to the telescoped kinetic model shown in Scheme S3**;** [**4p$_{Ac}$**] not included in fitting. Profiles obtained by *in situ* ^{1}H NMR monitoring.

Numerical kinetic fits: **4q$_H$**

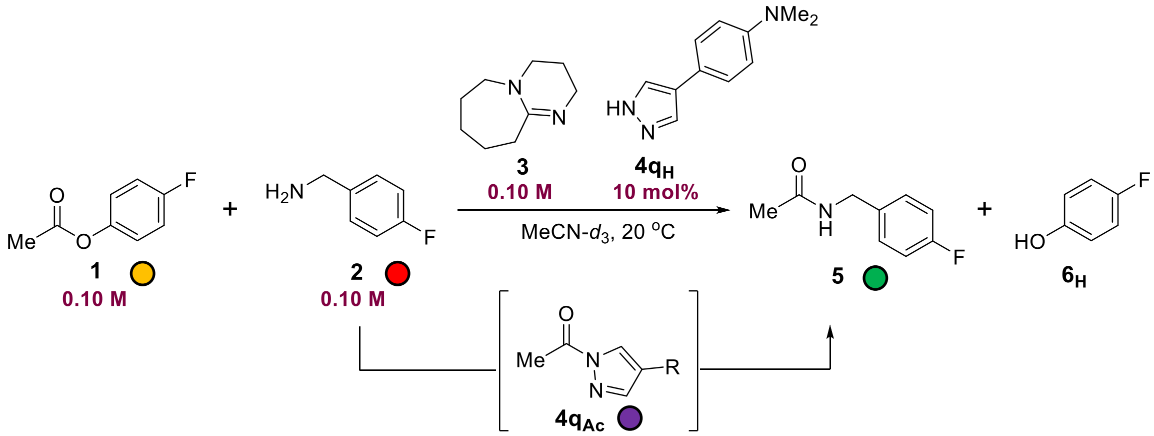

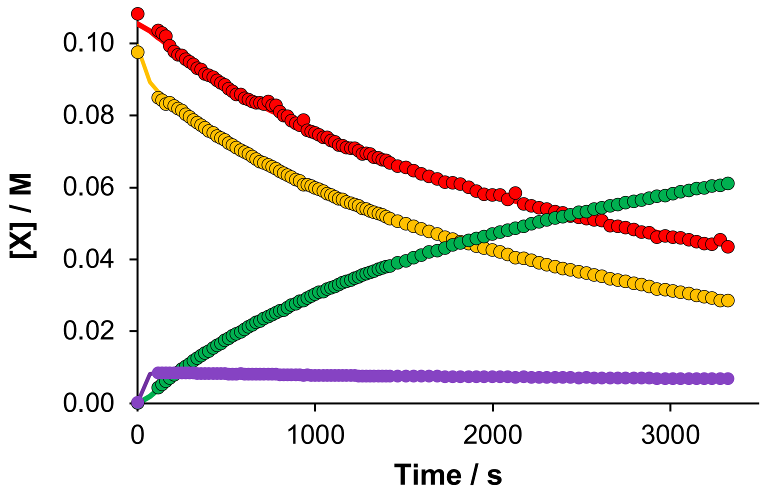

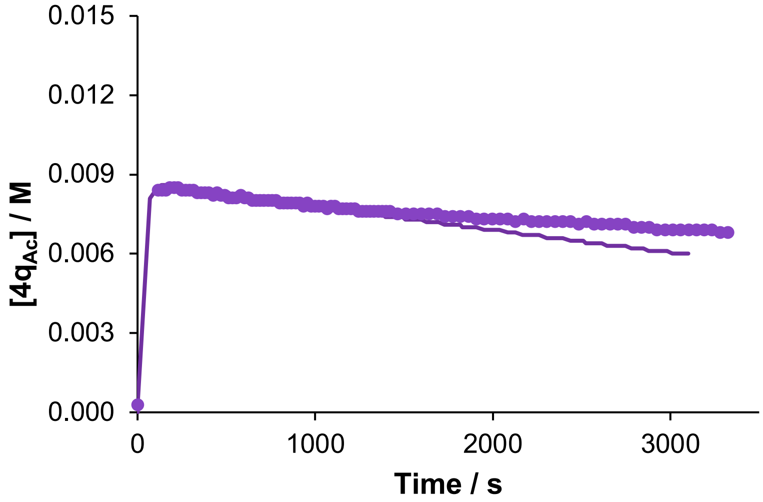

Figure S70: Experimental concentration-time profiles (points) for **1**, **2**, **5** and **4q$_{Ac}$** under standard catalytic conditions in MeCN ([**1**]$_0$ = 0.10 M, [**2**]$_0$ = 0.10 M, [**3**]$_0$ = 0.10 M, [**4q$_H$**]$_0$ = 0.010 M; MeCN-d_3, 20 °C), and simulated profiles (lines) calculated from kinetic parameters obtained by numerical fitting of [**1**], [**2**], and [**5**] to the telescoped kinetic model shown in Scheme S3**;** [**4q$_{Ac}$**] not included in fitting. Profiles obtained by *in situ* ^{1}H NMR monitoring.

Numerical kinetic fits: **4b$_H$**

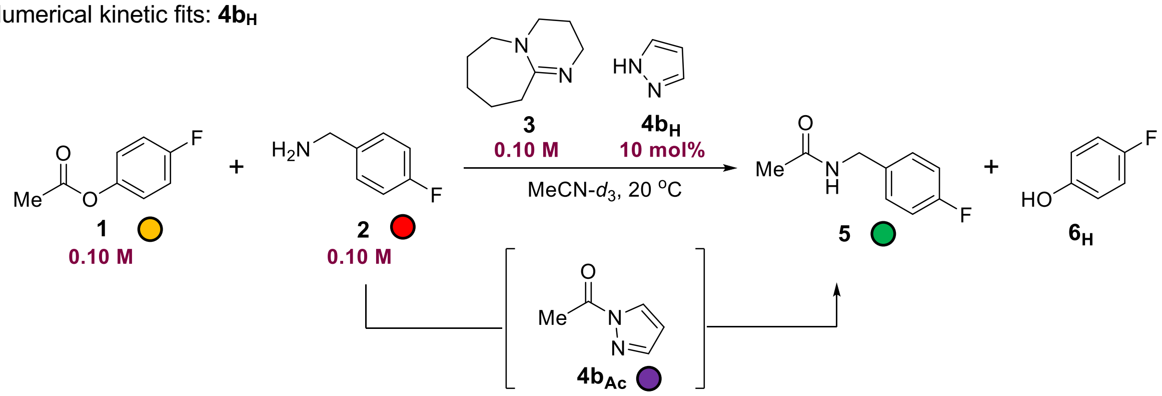

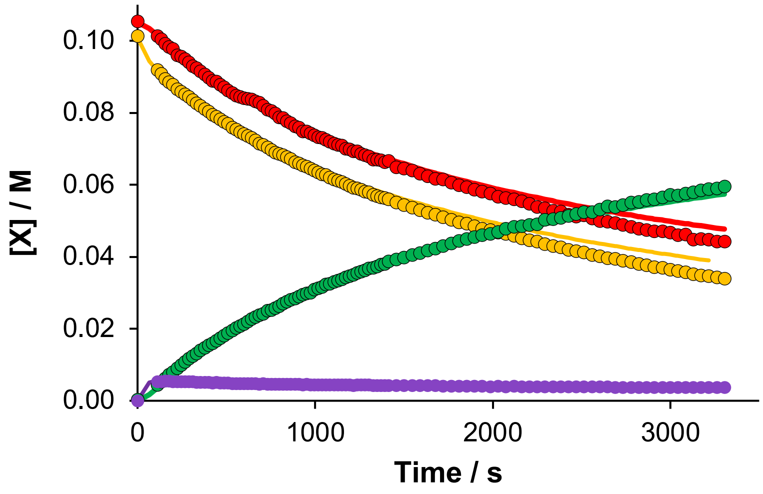

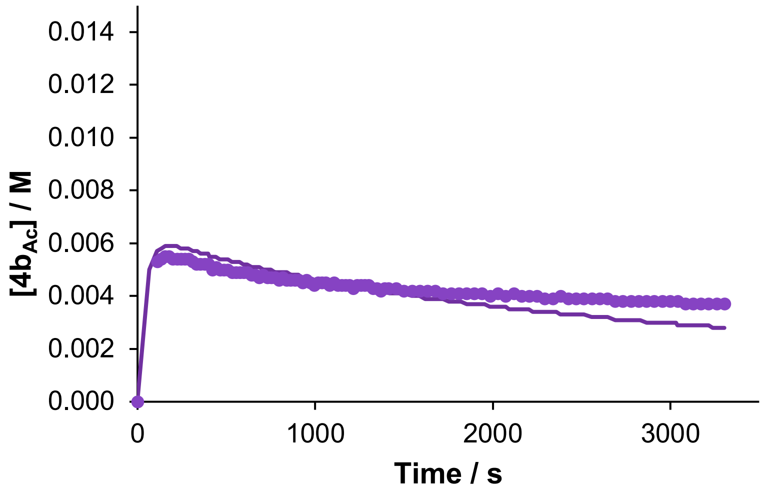

Figure S71: Experimental concentration-time profiles (points) for **1**, **2**, **5** and **4b$_{Ac}$** under standard catalytic conditions in MeCN ([**1**]$_0$ = 0.10 M, [**2**]$_0$ = 0.10 M, [**3**]$_0$ = 0.10 M, [**4b$_H$**]$_0$ = 0.010 M; MeCN-d_3, 20 °C), and simulated profiles (lines) calculated from kinetic parameters obtained by numerical fitting of [**1**], [**2**], and [**5**] to the telescoped kinetic model shown in Scheme S3**;** [**4b$_{Ac}$**] not included in fitting. Profiles obtained by *in situ* ^{1}H NMR monitoring.

*Numerical kinetic fits: **4r**H*

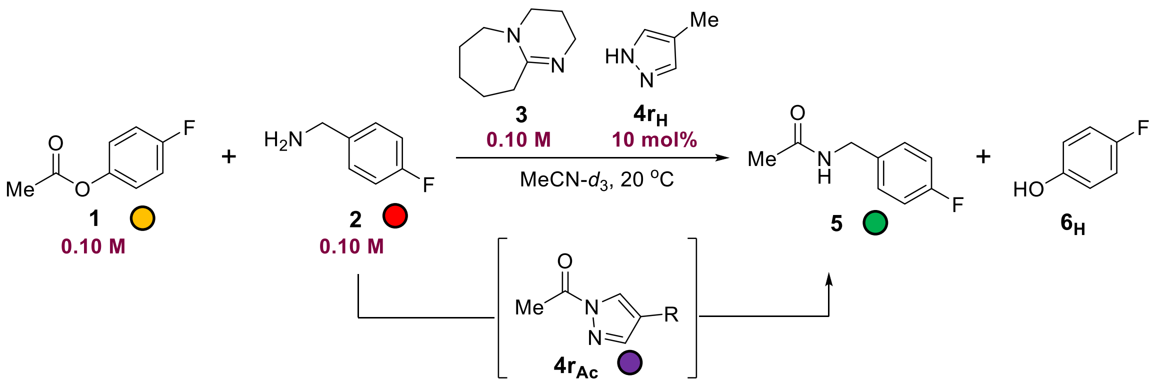

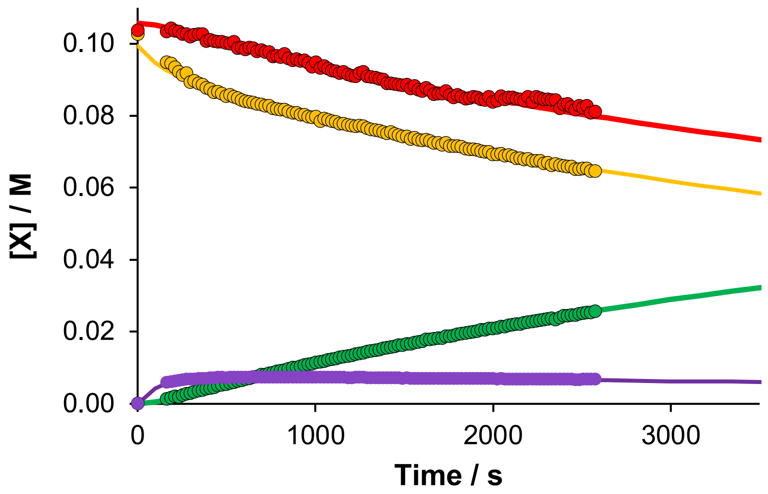

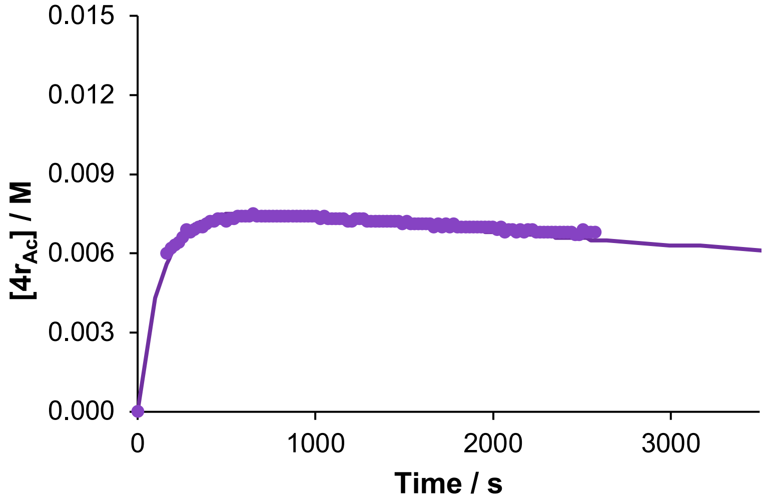

Figure S72: Experimental concentration-time profiles (points) for **1**, **2**, **5** and **4r**$_{\mathbf{Ac}}$ under standard catalytic conditions in MeCN ([**1**]$_0$ = 0.10 M, [**2**]$_0$ = 0.10 M, [**3**]$_0$ = 0.10 M, [**4r**$_{\mathbf{H}}$]$_0$ = 0.010 M; MeCN-d_3, 20 °C), and simulated profiles (lines) calculated from kinetic parameters obtained by numerical fitting of [**1**], [**2**], and [**5**] to the telescoped kinetic model shown in Scheme S3**;** [**4r**$_{\mathbf{Ac}}$] not included in fitting. Profiles obtained by *in situ* ^{1}H NMR monitoring.

S4. Intermolecular Competitions

S4.1. Competitive Hammett relationships

Overview

Linear free energy relationships (LFERs) for the acyl acceptor (**2-X**) were determined by intermolecular competition experiments under standard catalytic conditions in both MeCN-h_3 (regime **I**) and THF-h_8 (regime **II**).

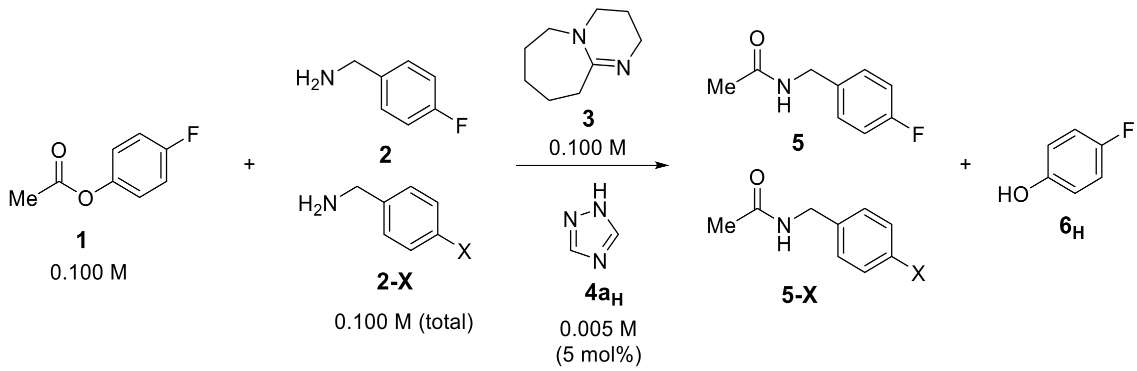

Scheme S5: General procedure for intermolecular competitions between amines.

In each experiment (Scheme S5), the reference amine **2** was assembled with an equimolar quantity, or two-fold excess, of the appropriately *para*-substituted amine **2-X** (i.e., [**2**]$_0$ = [**2-X**]$_0$, or 2.[**2**]$_0$ = [**2-X**]$_0$), in addition to appropriate quantities of auxiliary base (DBU **3**), catalyst (1,2,4-triazole **4a$_H$**; 5 mol%) and 1-fluoronaphthalene as an internal integration standard. The same *total initial concentration* of amine (i.e., [**2**]$_0$ + [**2-X**]$_0$ = 0.10 M) was used for all competition experiments.

Following initiation with *p*-F-PhAc **1** (*vide supra*), each competition reaction was monitored *in situ* by ^{19}F NMR spectroscopy, with the concentration of residual **2** calculated by direct (sum) integration and normalisation against 1-F-naphthalene; the concentrations of **1**, **5** and **6** (total) were calculated likewise. The concentration of the competing amine was either calculated directly (if fluorinated, e.g., for **2-CF$_3$**), or indirectly, in the latter case using approximation that [**2-X**] = [**2-X**]$_0$ – ([**6$_H$**]$_T$ - [**5**]); under standard catalytic conditions, hydrolysis is typically very minor, whilst the steady-state concentration of 1-acetyl-1,2,4-triazole **4a$_{Ac}$** was found to be below the detection limit of both ^{1}H/^{19}F NMR. For non-fluorinated amines, the initial concentration [**2-X**]$_0$ was calculated by a preliminary ^{1}H NMR analysis of a pre-reactive solution, prior to the addition of *p*-F-PhAc **1** (*vide infra*). Both the direct and indirect method of quantitation were used for the competition reaction between **2** and **2-CF$_3$** to confirm the validity of the indirect approach.

All competition reactions were initiated in the same manner (*vide infra*), with relative rate coefficients (k_X/k_F) obtained *via* non-linear regression using the mathematical formalisms developed by Bigeleisen and Wolfsberg (for first-order competitions).

Typical procedure

All solutions were prepared from a parent stock solution of 1-F-naphthalene (0.050 M) in MeCN-h_3 (or THF-h_8, as appropriate (see monitoring section for further detail on standard reaction assembly methods).

A pre-reactive stock solution of **2** (0.0666 M), **2-X** (0.0666 M), DBU **3**(0.133 M) and 1,2,4-triazole **4a$_H$** (0.0067 M, 5 mol%) was first prepared, in the appropriate solvent, using volumetric glassware, and transferred to a 5mm borosilicate NMR tube (600 µL; solution 1). This pre-reactive sample was loaded into the NMR probe (T_{NMR} = 293.1 K; 20.0°C), automatically tuned and matched to ^{1}H, and subjected to gradient shimming with the lock disabled; the receiver gain was optimised automatically, and a ^{1}H spectrum acquired (without solvent suppression) using standard acquisition parameters (*vide supra)* to determine the initial concentration ratio R_0 = [**2**]$_0$/[**2-X**]$_0$. The probe was then retuned and matched to ^{19}F, the receiver gain optimised, and a standard ^{19}F NMR spectrum acquired.

This pre-reactive sample (solution 1) was subsequently ejected from the spectrometer and transferred to a proximate fume hood (T_{IRM} = 20.0 °C $\pm$ 2.0 ºC), whereupon it was uncapped and the reaction initiated by injection of **1** (0.40 M, 200 µL, solution 2) with a glass microsyringe. The fully assembled reaction (800 µL; [**1**]$_0$ = 0.10 M, solution 3) was rapidly mixed by inverting the capped sample (three times) and shaking vigorously (for 5 s), and then swiftly transferred back to the spectrometer, after which monitoring was commenced immediately. ^{19}F spectra were acquired continuously until the reaction attained > 60 % conversion (F > 0.60; total amine), with each spectrum acquired using one transient per spectrum (ns = 1), a relaxation delay of t_{D1} = 10 s, and an acquisition time of t_{AQ} = 3.5 s (an additional, minimum inter-spectrum delay of t_{MD} > 10 s was used for all experiments).

Bigeleisen-Wolfsberg analysis

Relative rate coefficients (k_X/k_F) were obtained *via* non-linear regression using the mathematical formalisms developed by Bigeleisen and Wolfsberg.[S11] Assuming underlying or empirical first-order behaviour in the amine, the relative reactivities of the two competing amines k_X/k_F in each experiment were obtained from the substrate-focused Bigeleisen-Wolfsberg (B-W) equation:

$$\frac{k_X}{k_F} = \frac{\ln\left((1-F)\left(\frac{1+R_0}{1+R_F}\right)\right)}{\ln\left((1-F)\left(\frac{R_F}{R_0}\right)\left(\frac{1+R_0}{1+R_F}\right)\right)} \qquad (S20)$$

where F denotes the overall fractional conversion of both amines, R_F = [**2**]$_F$/[**2-X**]$_F$ is the instantaneous ratio of the two amines after a certain F, and R_0 denotes the corresponding ratio at the start of the reaction (F = 0).

$$F = 1 - \frac{[\mathbf{2-X}]_t + [\mathbf{2}]_t}{[\mathbf{2-X}]_0 + [\mathbf{2}]_0} \qquad (S21)$$

For all competitive LFERs reported in this work, relative rates were determined by non-linear regression with a rearranged form of the B-W equation:

$$F = 1 - \left(\frac{1 + R_F}{1 + R_0}\right)\left(\frac{R_F}{R_0}\right)^{\frac{\frac{k_X}{k_F}}{1-\frac{k_X}{k_F}}} \qquad (S22)$$

with the fractional conversions F and corresponding amine ratios R_F determined experimentally in regular intervals, using concentrations measured by *in situ* ^{19}F NMR spectroscopy (*vide supra*).

Non-linear regressions

Relative reactivities were determined under standard catalytic conditions in both MeCN-h_3 and THF-h_8, with the results of each non-linear regression summarised below. Note that R_F is defined inversely to the relative rate k_X/k_F, such that amines more reactive than the reference (**2**) lead to an increase in R_F over the course of the reaction, and *vice versa*. All reactions in MeCN-h_3 were taken to > 60 % conversion (F > 0.60); reactions in THF-h_8, meanwhile, were taken to > 50 % conversion (F > 0.50).

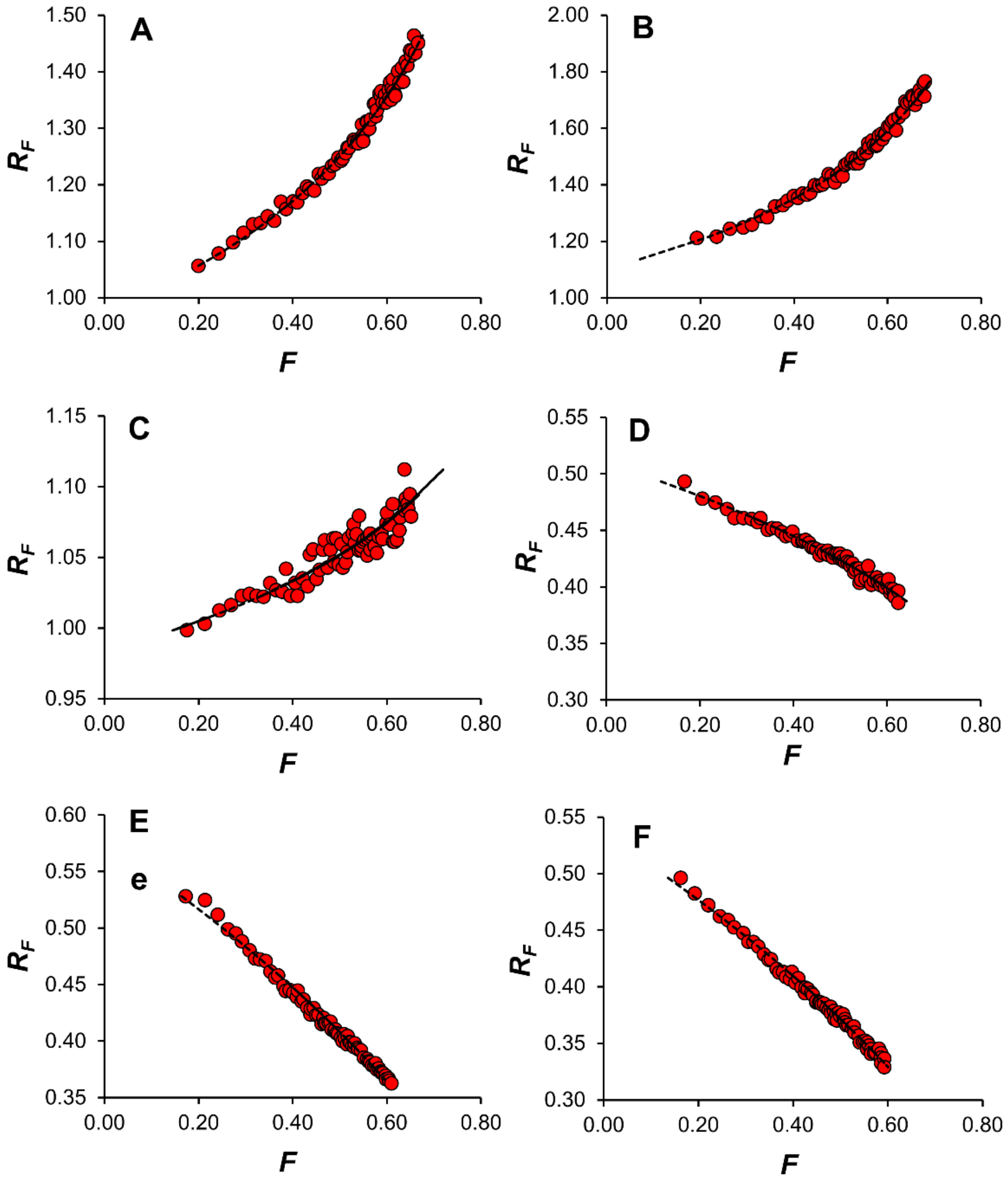

Figure S73: Determination of k_X/k_F by non-linear regression for six intermolecular competition reactions between **2** (reference) and **2-X** under standard catalytic conditions in MeCN (X = OMe, ^{t}Bu, H, Cl, CF_3). For each reaction $[\mathbf{1}]_0 = [\mathbf{3}]_0 = 0.10$ M; $[\mathbf{2}]_0 + [\mathbf{2\text{-}X}]_0 = 0.10$ M; $[\mathbf{4a_H}]_0 = 0.0050$ M (5 mol%). Approximately equimolar amine loadings (0.05 M : 0.05 M) were used for X = OMe, ^{t}Bu and H; a twofold excess of the competitor (0.067 M : 0.033 M) was used for X = Cl and CF_3. All concentrations determined by *in situ* ^{19}F NMR spectroscopy. A) X = OMe, $R_0 = 0.98$, $k_{OMe}/k_F = 1.43$. B) X = ^{t}Bu, $R_0 = 1.10$, $k_{tBu}/k_F = 1.48$. C) X = H, $R_0 = 0.98$, $k_H/k_F = 1.10$. D) X = Cl, $R_0 = 0.51$, $k_{Cl}/k_F = 0.77$. E) X = CF_3 (direct) $R_0 = 0.58$, $k_{CF3}/k_F = 0.63$. F) X = CF_3 (indirect), $R_0 = 0.54$, $k_{CF3}/k_F = 0.61$.

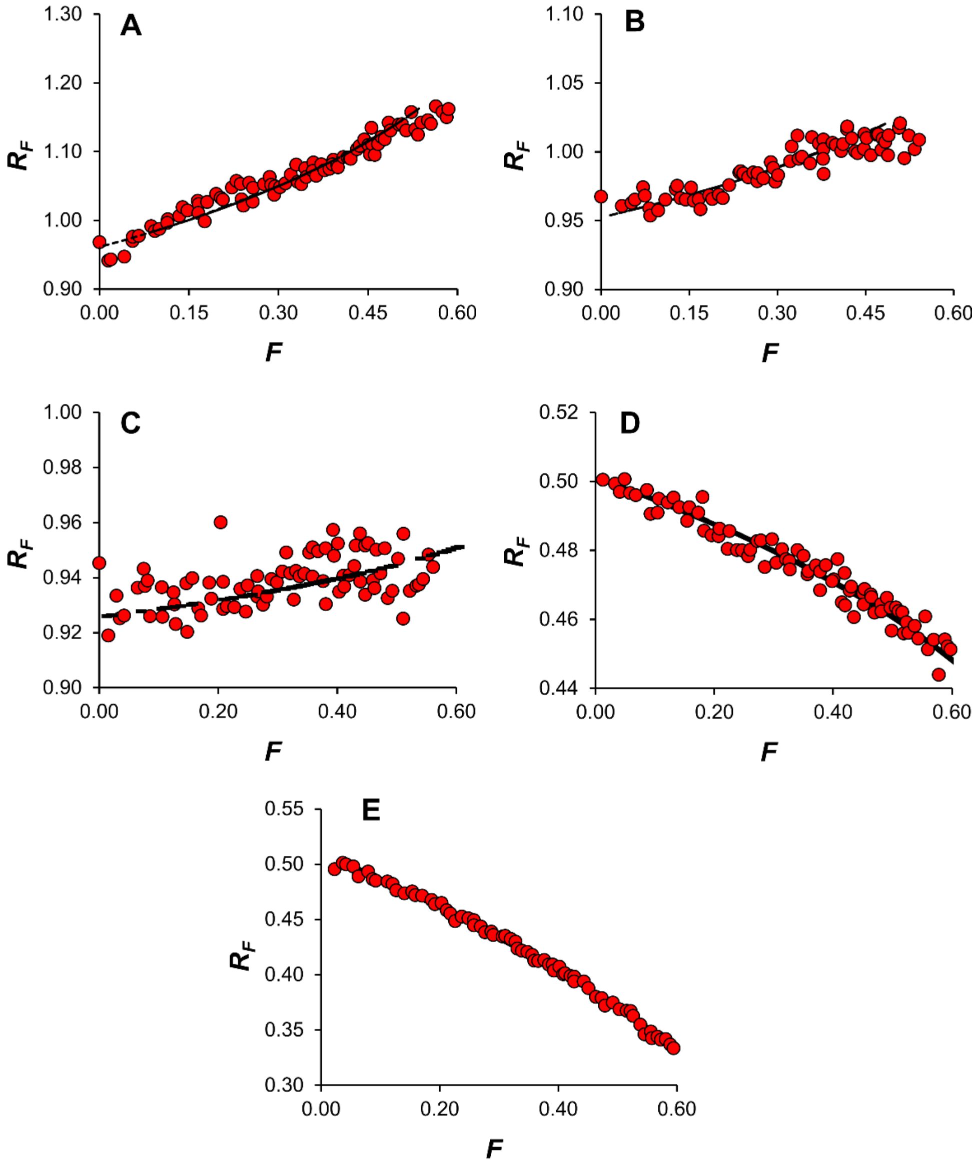

Figure S74: Determination of k_X/k_F by non-linear regression for six intermolecular competition reactions between **2** (reference) and **2-X** under standard catalytic conditions in THF (X = OMe, ^{t}Bu, H, Cl, CF_3). For each reaction [**1**]$_0$ = [**3**]$_0$ = 0.10 M; [**2**]$_0$ + [**2-X**]$_0$ = 0.10 M; [**4a$_H$**]$_0$ = 0.0050 M (5 mol%). Approximately equimolar amine loadings (0.05 M : 0.05 M) were used for X = OMe, ^{t}Bu and H; a twofold excess of the competitor (0.067 M : 0.033 M) was used for X = Cl and CF_3. All concentrations determined by *in situ* ^{19}F NMR spectroscopy. A) X = OMe, R_0 = 0.96, k_{OMe}/k_F = 1.28. B) X = ^{t}Bu, R_0 = 0.95, k_{tBu}/k_F = 1.11. C) X = H, R_0 = 0.93, k_H/k_F = 1.03. D) X = Cl, R_0 = 0.50, k_{Cl}/k_F = 0.89. E) X = CF_3 (direct) R_0 = 0.51, k_{CF3}/k_F = 0.65.

Hammett parameters and plots

X (MeCN-h_3)	σ_p	k_X/k_F	$\log_{10}(k_X/k_F)$
OMe	-0.268	1.43	0.155
^{t}Bu	-0.197	1.48	0.170
H	0	1.10	0.042
F	*0.062*	*1.00*	*0.000*
Cl	0.227	0.77	-0.111
CF_3	0.540	0.61	-0.212
		**ρ**	**-0.50**

Table S10: Hammett substituent constants (σ_p), relative rates (k_X/k_F) and reaction constant (ρ) determined by intermolecular competition for the **4a$_H$**-catalysed aminolysis of **1** by **2**/**2-X** (X = OMe, tBu, H, Cl, CF_3) and DBU **3** in MeCN. [**4a$_H$**]$_0$ = 0.005 M (5 mol%); [**1**]$_0$ = [**3**]$_0$ = 0.10 M. [**2**]$_0$ + [**2-X**]$_0$ = 0.10 M.

X (THF-h_8)	σ_p	k_X/k_F	$\log_{10}(k_X/k_F)$
OMe	-0.268	1.28	0.108
^{t}Bu	-0.197	1.12	0.049
H	0	1.03	0.013
F	*0.062*	1.00	0.000
Cl	0.227	0.89	-0.052
CF_3	0.540	0.65	-0.187
		**ρ**	**-0.34**

Table S11: Hammett substituent constants (σ_p), relative rates (k_X/k_F) and reaction constant (ρ) determined by intermolecular competition for the **4a$_H$**-catalysed aminolysis of **1** by **2**/**2-X** (X = OMe, tBu, H, Cl, CF_3) and DBU **3** in THF. [**4a$_H$**]$_0$ = 0.005 M (5 mol%); [**1**]$_0$ = [**3**]$_0$ = 0.10 M. [**2**]$_0$ + [**2-X**]$_0$ = 0.10 M.

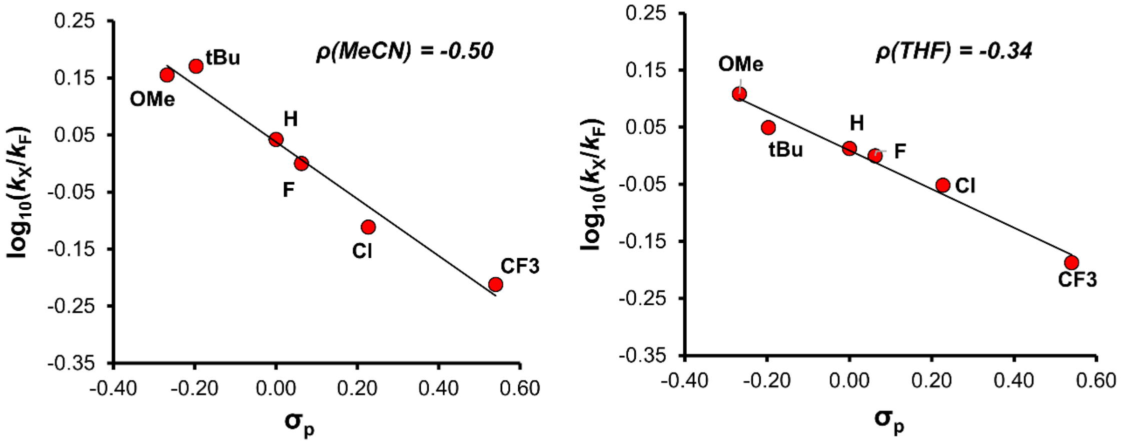

Figure S75: Hammett relationships determined by intermolecular competition for the **4a$_H$**-catalysed aminolysis of *p*-F-PhAc **1** in MeCN and THF under standard catalytic conditions (regimes I and II).

S4.2. $^{12}C/^{13}C$ KIE

Overview

The $^{12}C/^{13}C$ KIE for the carbonyl carbon in **1** was determined using isotopically labelled substrates. Specifically, the carbonyl $^{12}C/^{13}C$ KIE was determined through an intermolecular competition between [$^{13}CH_3$]-**1** (> 98% ^{13}C) and [^{13}CO]-**1** (> 99 % ^{13}C), using the large direct ^{13}C-^{1}H coupling ($^{1}J_{C1\text{-}H} \approx 130$ Hz) in the former to distinguish its CH_3 resonance from that of the carbonyl ^{13}C-labelled isotopologue ($^{2}J_{C2\text{-}H} \approx 7$ Hz) (Scheme S6). To determine the KIE, the competing isotopologous substrates were monitored continuously under standard catalytic conditions of regime I (**2**, **3**, **4a$_H$**; T_{NMR} = 293.1 K) by *in situ* ^{1}H NMR spectroscopy, with the KIE extracted by non-linear regression using the full substrate-focused Bigeleisen-Wolfsberg equation (*vide supra*). Due to intractable spectral congestion in THF-d_8, the KIE was determined in this manner for the reaction in MeCN-d_3 only.

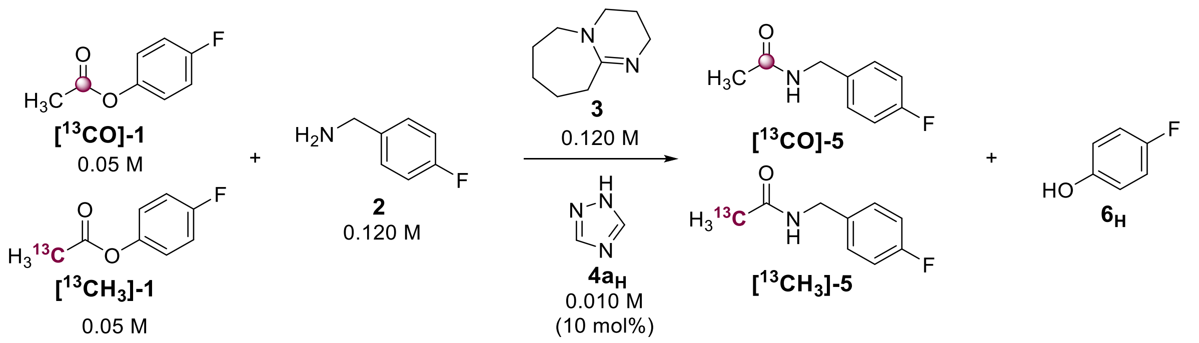

Scheme S6: Conditions for the measurement of the carbonyl $^{12}C/^{13}C$ KIE.

Attempts to directly monitor the competing substrates by $^{13}C\{^{1}H\}_{IG}$ NMR spectroscopy were ultimately unsuccessful: to accommodate the significant disparity in the rates of longitudinal relaxation between the ^{13}CO and $^{13}CH_3$ nuclei ($T_1(^{13}CO) \approx 37$ s; and $T_1(^{13}CH_3) \approx 10$ s), very large relaxation delays were required during monitoring, in turn precluding the acquisition of spectra with sufficient signal-to-noise. Alternative strategies for measuring the $^{12}C/^{13}C$ KIE by ^{19}F NMR, in a manner analogous to the determination of the amine $^{14}N/^{15}N$ KIE (*vide infra*), were precluded by kinetically-competitive self-exchange between **1** and **6$_H$** (usually inconsequential, but fundamentally problematic when relying on remote isotopic labels on the phenol ring to distinguish the competing isotopologues of **1**).

Reaction assembly

A stock solution of **2** (0.12 M), **4a$_H$** (0.010 M) and 1-F-naphthalene (0.025 M) in MeCN-d_3 was first dispensed to a 5 mm borosilicate NMR tube (Solution1; 700 μL); this sample was prepared < 2 h in advance of monitoring. Immediately prior to monitoring, [$^{13}CH_3$]-**1** (4.5 μL, 0.035 mmol; 0.05 M) and [^{13}CO]-**1** (4.5 μL, 0.035 mmol; 0.05 M) were then injected directly into this sample as neat liquids to afford a metastable solution (Solution 2); immediately thereafter, the sample was loaded into the spectrometer, tuned and matched to ^{1}H, locked to the ^{2}H signal of MeCN-d_3, subjected to gradient shimming, the receiver gain optimised automatically, and a ^{1}H NMR spectrum recorded to assess the initial ratio of the competing isotopologues, *R$_0$*. In the absence of DBU **3**, and at the concentrations in question, aminolysis of **1** was negligible during this time (< 5 min).

Having acquired the initial ^{1}H spectrum, the sample was quickly ejected from the spectrometer and uncapped in a proximate fume hood, whereupon catalysis was initiated with the addition of a slight excess of **3** (12.5 μL; 0.084 mmol; 0.12 M) as a neat liquid. On account of the rapid initial turnover in MeCN-d_3, as soon as **3** was added the NMR tube was capped, rapidly inverted and shaken, loaded into the NMR probe, locked, rapidly re-shimmed, and monitored continuously thereafter. Although this sequence was undertaken rapidly and without delay, care was nevertheless taken to re-shim the sample sufficiently well to ensure high resolution and good line shape during monitoring (for accurate quantitation, it proved important to avoid even relatively modest overlap between the CH_3 resonances of the competing substrates with the myriad other upfield ^{1}H resonances). During monitoring, ^{1}H spectra were acquired back-to-back using a standard pulse-acquire sequence and a specially configured set of acquisition parameters, including: (i) a 30° excitation pulse; (ii) a single transient per spectrum (ns = 1); (iii) a relaxation delay of t_{D1} = 60 s; and (iv) an extended acquisition time of t_{AQ} = 8.0 s (65536 points). For the production run, ^{1}H spectra were acquired continuously from 50 % conversion (F = 0.5) to 85 % (F = 0.85); despite several attempts, the first half of the reaction evolution was too rapid to be captured following manual reaction assembly (the requirement to obtain high resolution, and thereby to reshim the sample following initiation with DBU, ultimately imposed a minimum bound on the dead time).

Data analysis

^{1}H spectra obtained over the course of monitoring were then processed together as a stack, with zero-filling (128k points), and phasing (zeroth order only) and baseline (first-order Bernstein polynomial) corrections, applied consistently to all spectra. To avoid other, nearby resonances compromising the quantitation of the two isotopologous substrates, no exponential weighting functions were applied to any of the FIDs.

Concentrations of the competing isotopologues at time each point were quantified by direct (sum) integration and normalisation against 1-F-naphthalene, using constant integration ranges for all spectra, and the ratio R_F and the fractional conversion F were determined according to standard methods (*vide supra*).

$$F = 1 - \left(\frac{1 + R_F}{1 + R_0}\right)\left(\frac{R_F}{R_0}\right)^{\frac{\frac{k_{13CH3}}{k_{13CO}}}{1-\frac{k_{13CH3}}{k_{13CO}}}} \qquad (S23)$$

Non-linear regression of F, Figure S76, in accordance with equation S23, in turn afforded a relative rate of k_{13CH3}/k_{13CO} = 1.041(2) (see main text). Assuming a secondary ^{12}C/^{13}C KIE of unity at the methyl carbon in **1** (i.e, k_{12CH3}/k_{13CH3} = 1.0000), this value corresponds to the intrinsic KIE at the carbonyl carbon, i.e., k_{12CO}/k_{13CO} = 1.041(2).

S4.3. ^{14}N/^{15}N KIE

Overview

Amine ^{14}N/^{15}N KIEs were determined through intermolecular competition between [Ar-d_1]-**2** (> 99% D) and [^{15}N]-**2** (> 98 % ^{15}N), with the secondary isotope shift $^3\Delta$F(D) in the used to distinguish the *para*-^{19}F

resonances of the two isotopologues ($^3\Delta F(D) \approx 0.27$ ppm) (Scheme S7). In each experiment the competing substrates were subjected to standard catalytic conditions in MeCN-h_3 (**1**, **3**; **4a$_H$**, regime I or **4b$_H$**, regime III; T_{NMR} = 293.1 K) and monitored *in situ* by $^{19}F\{^1H\}$ NMR spectroscopy with inverse-gated ^1H decoupling (elimination of ^{19}F-^1H couplings lead to a useful enhancement in signal-to-noise). For each catalyst, a control intermolecular competition, between [Ar-d_1]-**2** (> 99% D) and **2**, was conducted to account for any KIE arising from mono-deuteration of aryl ring; this was essentially negligible in all cases (i.e., 1.000 – 1.005), as expected.

Reaction assembly

Intermolecular competition reactions were assembled in the standard manner. A stock solution of [Ar-d_1]-**2** (0.066 M), **2** *or* [^{15}N]-**2** (0.066 M), DBU **3** (0.16 M), 1,2,4-triazole **4a$_H$**/pyrazole **4b$_H$** (0.0133 M) and 1-F-naphthalene (0.025 M) in MeCN-h_3 was first prepared and dispensed to a 5 mm borosilicate NMR tube (Solution 1; 700 µL); this sample was prepared < 2 h in advance of monitoring. At leisure, this sample was loaded into the spectrometer, tuned and matched to ^1H, subjected to gradient shimming with the lock off (topshimfindsolvent), the receiver gain optimised automatically, and a ^1H NMR spectrum (without solvent suppression) recorded to assess the purity of the sample. Thereafter, the probe was retuned and matched to ^{19}F, the receiver gain optimised automatically, and a $^{19}F\{^1H\}$ spectrum acquired with inverse-gated ^1H coupling to assess the initial ratio of the isotopologues, R_0. Finally, the probe was retuned and matched to ^1H. At this point, the pre-reactive sample was ejected from the spectrometer and uncapped in a proximate fume hood, whereupon catalysis was initiated with the addition of a modest excess of **1** (200 uL, 0.48 M; Solution 2) as a solution in the appropriate solvent. Immediately after initiation, the NMR tube was capped, rapidly inverted and shaken, loaded into the NMR probe, rapidly re-shimmed, retuned and matched to ^{19}F, and monitored continuously. Although this sequence was undertaken rapidly and without delay, care was nevertheless taken to re-shim the sample sufficiently well to ensure high resolution and good line shape during monitoring.

During monitoring, $^{19}F\{^1H\}$ spectra were acquired back-to-back using a specially configured set of acquisition parameters, including: (i) a 30° excitation pulse; (ii) a four transients per spectrum (ns = 4); (iii) a relaxation delay of t_{D1} = 10 s between transients; and (iv) an acquisition time of t_{AQ} = 3.5 s (131072 points). For self-consistency, all reactions were monitored to roughly the same level of conversion ($F \approx 0.83$); higher conversions were avoided on account of insufficient signal-to-noise.

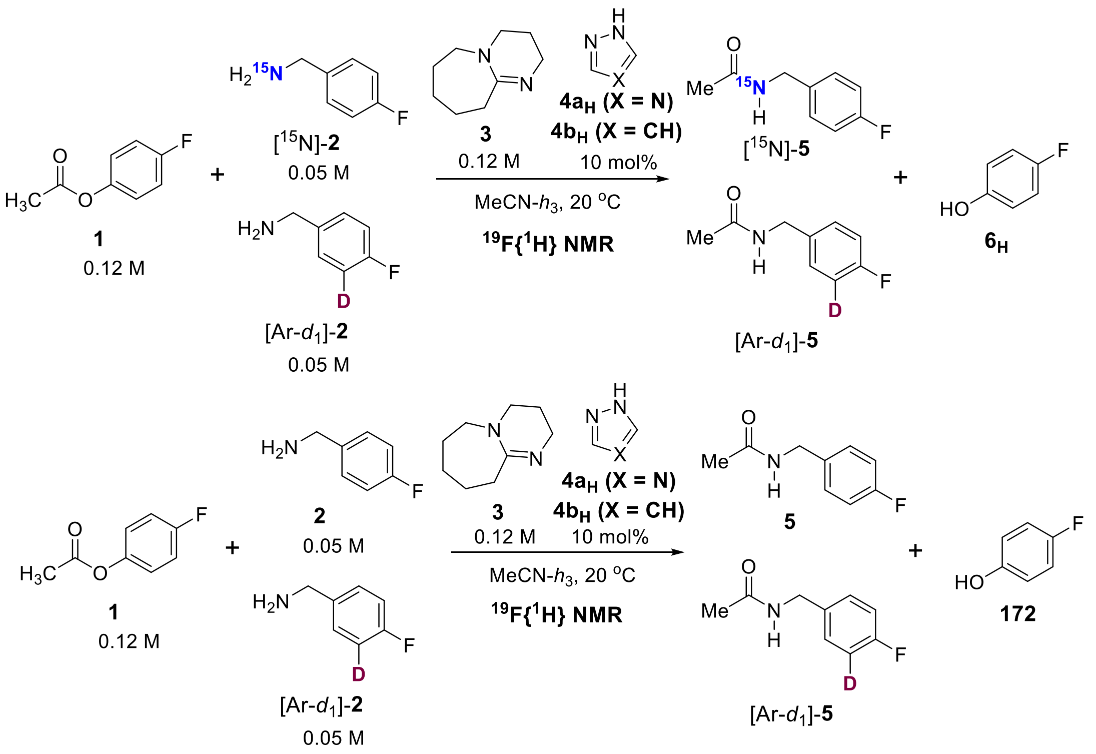

Scheme S7: Conditions for the measurement of the amine $^{14}N/^{15}N$ KIE in the **4a**$_H$- and **4b**$_H$-catalysed aminolysis of **1** with **2** and **3** in MeCN (regimes I and III, respectively).

Data analysis

$^{19}F\{^1H\}$ spectra obtained over the course of each reaction were processed together as a stack, with zero-filling (256k points), exponential line broadening (0.4 Hz), and both phasing (zeroth and first order) and baseline (third-order Bernstein polynomial) corrections applied consistently to all spectra. Concentrations were quantified by direct (sum) integration and normalisation against 1-F-naphthalene, using constant integration ranges for all spectra; the isotopologue ratio R_F and fractional conversion F were calculated according to standard definitions. As all $^{14}N/^{15}N$ KIEs were found to be very small, each KIE was determined using a simplified version of the substrate-focused B-W equation (essentially exact for heavy atom KIEs 0.97 – 1.03). Specifically, each $^{14}N/^{15}N$ KIE was determined by non-linear regression of R_F according to equation S 24. For simplicity, the uncertainty in each KIE, ε_{KIE}, was estimated from the standard error, ε_m, in the gradient m of the linear regression of ln(R_F), via equations S25 and S26.

$$\frac{R_F}{R_0} = (1-F)^{\frac{1}{\frac{k_{14N}}{k_{15N}}}-1} \qquad (S24)$$

$$\ln R_F = \left(\frac{1}{\frac{k_{14N}}{k_{15N}}} - 1\right)\ln(1-F) + \ln R_0\,, \qquad m = \frac{1}{\frac{k_{14N}}{k_{15N}}} - 1 \qquad (S25)$$

$$\varepsilon_{KIE} = \varepsilon_m \left(\frac{k_{14N}}{k_{15N}}\right)^2 \qquad (S26)$$

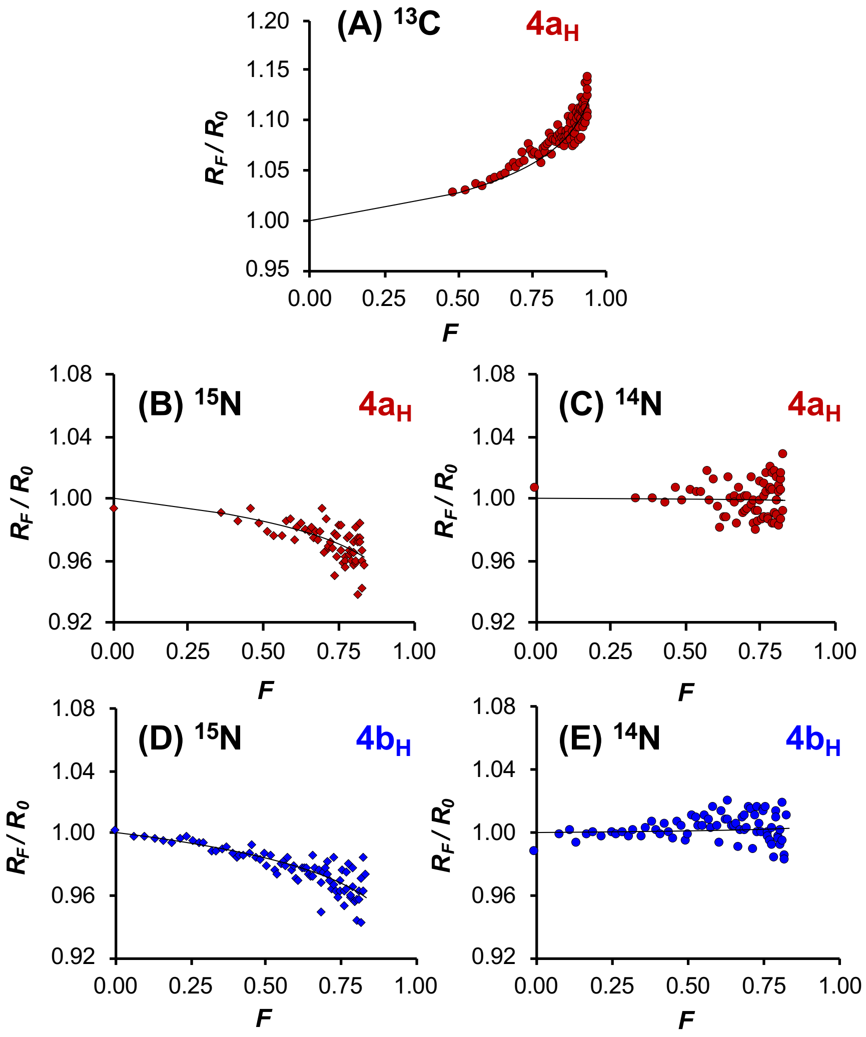

Figure S76: Fractionation profiles (R_F/R_0 vs F) for intermolecular competitions to determine heavy atom KIEs. (**A**): competition of [^{13}CO]-**1** (0.05 M) and [^{13}CH$_3$]-**1** (0.05 M) under near-standard regime I conditions ([**2**]$_0$ **=** [**3**]$_0$ **=** 0.12 M; [**4a$_H$**] = 0.010 M, 10 mol%; MeCN-d_3, 20 °C), obtained by *in situ* ^{1}H NMR monitoring. Relative rate k_{13CH3}/k_{13CO} = 1.041(2) determined by non-linear regression using the full substrate-focused Bigeleisen-Wolfsberg equation. R_F = [[^{13}CO]-**1**]/[[^{13}CH$_3$]-**1**] R_0 = [[^{13}CO]-**1**]$_0$/[[^{13}CH$_3$]-**1**]$_0$. 1 – F = ([[^{13}CO]-**1**] + [[^{13}CH$_3$]-**1**])/([[^{13}CO]-**1**]$_0$ + [[^{13}CH$_3$]-**1**]$_0$). (**B**) **4a$_H$**-catalysed intermolecular competition of [Ar-d_1]-**2** (0.05 M; "[Ar-d_1]") and [^{15}N]-**2** (0.05 M; "[^{15}N]"), for which R_F = [^{15}N]/[Ar-d_1], R_0 = [^{15}N]$_0$/[Ar-d_1]$_0$, (1 – F) = ([^{15}N] + [Ar-d_1])/([^{15}N]$_0$ + [Ar-d_1]$_0$) and $k_{14N,d1}/k_{15N}$ = 0.979(3). (**C**) **4a$_H$**-catalysed intermolecular competition of [Ar-d_1]-**2** (0.05 M; "[Ar-d_1]") and **2** (0.05 M; "[^{14}N]"), for which R_F = [^{14}N]/[Ar-d_1], R_0 = [^{14}N]$_0$/[Ar-d_1]$_0$, (1 – F) = ([^{14}N] + [Ar-d_1])/([^{14}N]$_0$ + [Ar-d_1]$_0$) and $k_{14N,d1}/k_{14N}$ = 0.999(4). (**D**) Analogous fractionation profile for the **4b$_H$**-catalysed intermolecular competition of [Ar-d_1]-**2** (0.05 M) and [^{15}N]-**2** (0.05 M), for which $k_{14N,d1}/k_{15N}$ = 0.977(2). (**E**) Analogous fractionation profile for the **4b$_H$**-catalysed intermolecular competition of [Ar-d_1]-**2** (0.05 M) and **2** (0.05 M), for which $k_{14N,d1}/k_{14N}$ = 1.001(2).

S5. ^{1}H DOSY NMR

S5.1. Overview

^{1}H-detected diffusion-ordered NMR spectroscopy (^{1}H DOSY NMR) was used to probe the homo- and heteroconjugation of **6**$_\mathbf{H}$ in MeCN under a range of basic conditions. To accommodate solutions of differing compositions and viscosities – especially those generated during titrations with strong bases, leading to solutions of variable ionic strengths – all DOSY measurements were undertaken with *internal calibration*. In contrast to measurements of absolute diffusion coefficients, internally calibrated DOSY NMR provides a means of solution-phase molecular weight determination that hinges primarily upon the *relative mobilities* – i.e., the microscopic structures – of solutes, and not the global properties of the solution *per se*. Internally-calibrated molecular weight measurements are thus much less sensitive to inter-experiment fluctuations in temperature, viscosity, and convection currents, yet retain the ability to impart information about the temperature-sensitivity of microscopic structure (i.e., intermolecular association). The measurement of molecular weights by internally-calibrated DOSY NMR also circumvents the need for exacting instrumental calibrations of, e.g., the maximum gradient strength of the probe.

To achieve internal calibration, all samples subjected to ^{1}H DOSY NMR measurements were prepared using a common suite of five internal diffusion standards, comprising: Et_2O **12** (0.025 M, M_w = 74.1 g mol^{-1}), cyclohexane **13** (CyH; 0.015 M, 84.2 g mol^{-1}), tetramethylbutane **14** (TMBu; 0.010 M, 114.1 g mol^{-1}), 1,3,5-trimethoxybenzene **15** (1,3,5-TMB; 0.015 M, 168.2 g mol^{-1}) and 1,3,5-triiropropylbenzene **16** (1,3,5-TIB; 0.015 M, 204.4 g mol^{-1}). Fluorobenzene **17** and CH_2Cl_2 were also typically added to solutions as a ^{19}F shift and heavy atom diffusion references, respectively, but were not used in diffusion calibrations. These five internal standards – all non-protic, poor hydrogen bond acceptors, and weakly polar – were selected in a deliberate attempt to: (i) minimise spectral congestion; (ii) ensure chemical compatibility, both with each other and with likely analytes (i.e., strong organic bases, N-heterocycles, organic anions and cations); (iii) avoid confounding intermolecular interactions, either between standards or between a standard and analyte; (iv) provide geometric, and density, consistency with **6**$_\mathbf{H}$ and its aggregates (i.e., dissipated ellipsoids, or compact spheres of 100 < M_W < 150 g mol^{-1}, were favoured; first-row elements only);[S12] and (v) provide a pertinent range of molecular weights for accurate linear regression. All five internal diffusion standards were deployed at consistent concentrations across all samples, with the concentration of each standard adjusted qualitatively to obtain signals of similar intensity to the other four (i.e., concentrations were varied to account for differences in the number of spins contributing to, and extent of homonuclear coupling in, the various ^{1}H resonances of the five standards).

In accordance with standard practice, logarithmic calibration curves ($\log_{10}(M_w)$ vs $\log_{10}(D_T)$) were constructed independently for each DOSY experiment, with the unknown molecular weight of the analyte(s) in turn determined by inter-/extrapolation.

S5.2. Data acquisition

Translational self-diffusion coefficients (D_T) for internal standards and analytes were measured using ^{1}H-detected diffusion-ordered NMR spectroscopy (^{1}H DOSY NMR), with all studies performed at T_{NMR} = 293.1 K (20.0 °C) on a Bruker Ascend 400 MHz NMR spectrometer fitted with a broadband direct-detect CryoProbe Prodigy unit (maximum gradient strength of g_{max} = 53.5 G cm^{-1}). All samples were prepared in anhydrous MeCN-d_3 (3 Å MS), and dispensed to borosilicate NMR tubes (5mm, Norrell; 600 – 800 μL sample volume). The ambient temperature of the instrument room was controlled to within ± 2 °C of the specified probe temperature (T_{IRM} = 20.0 ± 2.0 °C), and an elevated nitrogen gas flow rate of 535 L h^{-1} was set on the spectrometer to minimise the temperature gradient across the sample once in the probe; higher flow rates, and sample spinning, were avoided to ensure sample stability.

All ^{1}H DOSY experiments were performed using a longitudinal eddy current delay sequence with bipolar gradient pulses and two spoil gradient pulses (ledbpgp2s), with sinusoidal gradient shapes (SINE.100) used throughout. The bipolar gradient pulse duration (p30 = δ/2 = 550 μs), Eddy current delay (d21 = 5 ms) and gradient recovery delay (d16 = 0.20 ms) were fixed for all experiments, whilst the diffusion delay (d20 = Δ) was optimised for each sample; typically d20 = 60–100 ms. Production quality ^{1}H DOSY NMR spectra were obtained by collecting 16 gradient increments in the diffusion (F1) direction, with quadratic ramping between initial and final gradient amplitudes of 5 % (f_Z = 0.05) and 95 % (f_Z = 0.95), respectively. Each spectrum along the diffusion (F1) direction was acquired with 16 transients (ns = 16), four dummy transients (ds = 4), an acquisition time of t_{AQ} = 2.0 s and a relaxation delay of t_{D1} = 10 s between successive transients, giving a total experiment time of approximately 53 min. No apparent improvements in data quality at T_{NMR} = 293.1 K were observed with Bruker's convection-compensated double stimulated echo pulse sequence (dstebpgp3s), or with 3 mm NMR tubes.

Each experiment was processed initially as a stack of 1D 1H spectra, with each FID subjected to exponential apodization (4.0 Hz), manual phasing (zeroth and first order) and Whittaker smoother baseline corrections. Pseudo two-dimensional ^{1}H DOSY spectra, in turn, were generated in MNova (Version 11) using the Bayesian DOSY transform, one repetition, a resolution factor of 1.0 and 256 points in the diffusion dimension.

S5.3. Relevant theory

For ^{1}H DOSY experiments performed with bipolar gradient pulse sequences, the signal attenuation I/I_0 observed following the application of a longitudinal field gradient ($g_z = f_z\, g_{max}$), relative to the absence of such a gradient (I_0), is given by:[S13]

$$\ln\left(\frac{I}{I_0}\right) = -\,(\gamma_H \delta \sigma f_z g_{\max})^2 \Delta_{ef}\, D_T \qquad (S27)$$

$$\Delta_{ef} = \left(\Delta + \frac{(2\kappa - 2\lambda - 1)\delta}{4} - \frac{\tau}{2}\right) \qquad (S28)$$

where $\gamma_H = 2.6752 \times 10^4$ rad s^{-1} G^{-1} is the gyromagnetic ratio of the proton; Δ (s) is the diffusion delay; κ, λ and σ are dimensionless parameters that depend upon the exact shape of the gradient pulse; δ (s) is the total duration of the gradient pulses (twice the length of each individual bipolar pulse); τ is the total delay between the two bipolar gradient pulses, comprising both the gradient recovery delay and the duration of the intervening 180° pulse; g_{max} (G cm^{-1}) is the maximum gradient strength of the probe; f_z is the fraction of the maximum gradient strength applied to the sample; and D_T (cm^2 s^{-1}) is the translational self-diffusion coefficient. Δ_{ef} is the *effective* diffusion time, accounting for the particulars of the pulse sequence and gradient pulse shape.[13] For sine bell gradient pulses (e.g., Bruker's SINE.100), κ = 3/8, λ = 1/2, and σ = 2/π, leading to:

$$\ln\left(\frac{I}{I_0}\right) = -\frac{4}{\pi^2}(\gamma \delta f_z G_{\max})^2 \left(\Delta - \frac{5\delta}{16} - \frac{\tau}{2}\right) D_T \qquad (S29)$$

Given that δ is about two orders of magnitude smaller than Δ, and τ shorter still, this equation is often simplified (including, apparently, by MNova) to the form:

$$\ln\left(\frac{I}{I_0}\right) \approx -\frac{4}{\pi^2}(\gamma \delta f_z G_{\max})^2 \left(\Delta - \frac{\delta}{3}\right) D_T \qquad (S30)$$

During manual analysis, the diffusion coefficient D_T of each component can then be obtained by linear regression of the signal attenuation observed in a corresponding resonance as a function of applied gradient strength.

S5.4. Data analysis

For each individual DOSY experiment, the measured diffusion coefficients of the five diffusion standards (Et_2O **12**, CyH **13**, TMBu **14**; 1,3,5-TMB **15**; 1,3,5-TIPB **16**) were used to construct a unique *internal calibration curve* ($\log_{10}(D)$ vs $\log_{10}(M_w)$), allowing the molecular weight of unknown analytes in the same sample to be estimated by linear interpolation/extrapolation via equation S32 (α and β were treated as empirical parameters). Translational self-diffusion constants themselves were typically extracted directly from pseudo-2D DOSY spectra, obtained following an automatic Bayesian transformation of the 1D diffusion data in MNova.

$$D = \beta M_w^{\alpha} \qquad (S31)$$

$$\log_{10}(D) = \alpha \log_{10} M_w + \log_{10} \beta \qquad (S32)$$

S5.5. Data analysis - validation

To test the reproducibility of the procedure outlined above, the robustness of the automated DOSY processing in MNova, and the direct extraction of diffusion coefficients from pseudo 2D 1H DOSY spectra, extensive validation experiments, and analysis, were undertaken for a reference sample (MeCN-d_3) comprising the five internal diffusion standards in their typical concentrations (in addition to PhF).

Several key figures pertaining to this comparative analysis are shown below, including: (i) raw diffusion data obtained for this sample, in the form of 16 stacked diffusion-ordered 1D spectra, in Figure S77; (ii) linearised Stejskal-Tanner plots for each of the key five components (Et_2O **12**, CyH **13**, TMBu **14**, 1,3,5-TMB **15**, 1,3,5-TIB **16**) in Figure S78; the Bayesian-transformed pseudo-2D ^{1}H DOSY spectrum, generated with MNova, in Figure S79; and (iv) logarithmic calibration curves constructed from diffusion coefficients extracted by manual (1D) and automatic (2D) analysis (Figure S80). Crucially, the logarithmic calibration curves obtained in each case (1D, 2D) were found to afford similarly accurate estimates of the molecular weights of the five standards, displaying excellent room-mean square errors (RMSE) of 1.5 g mol^{-1} and 1.4 g mol^{-1}, respectively (Table S12). On this basis all further analysis was conducted automatically, using diffusion coefficients extracted directly from pseudo-2D ^{1}H DOSY spectra.

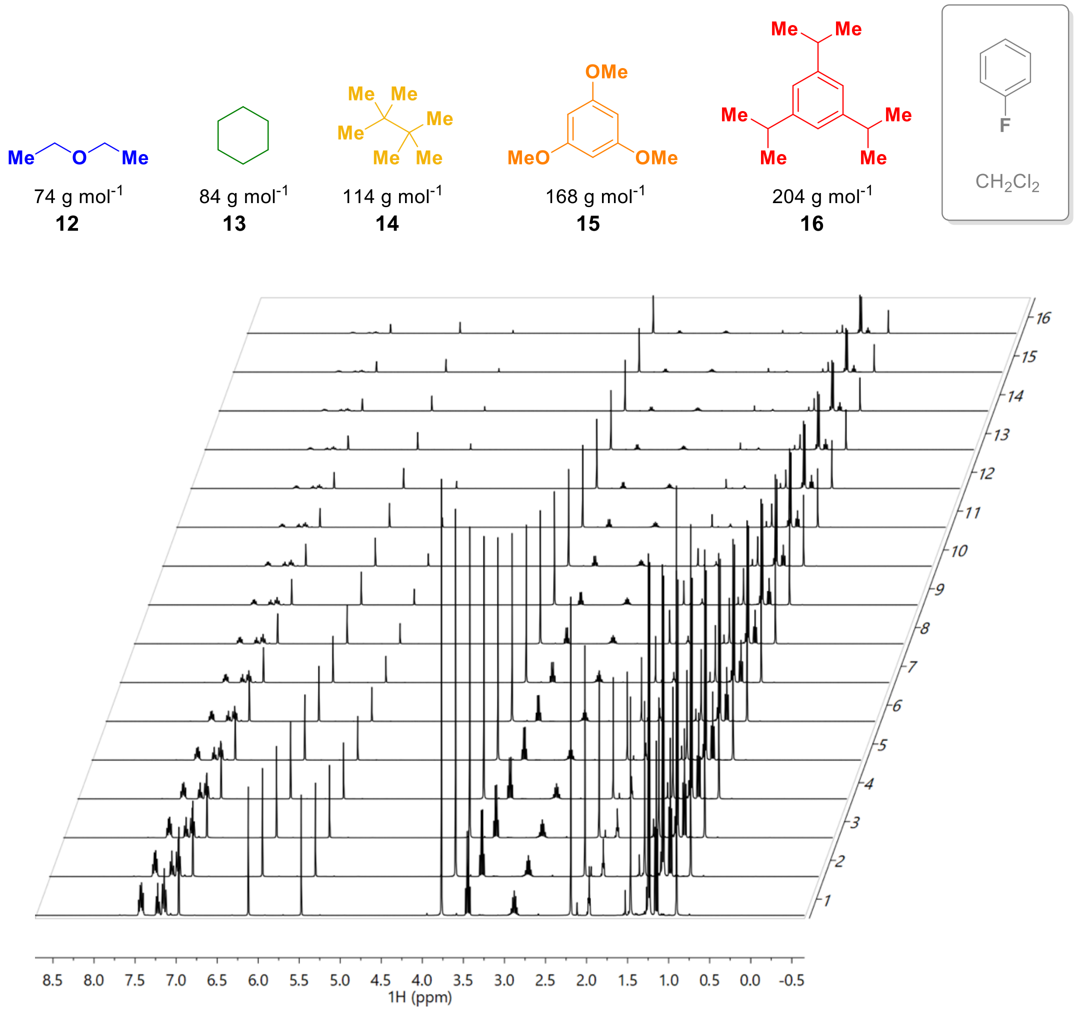

Figure S77: Stacked diffusion data obtained by ^{1}H DOSY NMR on a sample of Et_2O **12** (0.025 M), CyH **13** (0.015 M), TMBu **14** (0.010 M), 1,3,5-TMB **15** (0.015 M), 1,3,5-TIB **16** (0.015 M) in MeCN-d_3. Fluorobenzene (PhF; 0.050 M) was added for self-consistency with ^{1}H DOSY titrations involving *p*-F-PhOH $\mathbf{6_H}$, which required a ^{19}F chemical shift internal standard, and CH_2Cl_2 was added as a heavy-atom benchmark. ^{1}H spectra obtained in 16 increments along the diffusion dimension, with quadratic (q) ramping between 5 % (f_z = 0.05) and 95 % (f_z = 0.95) of the maximum gradient strength of the probe, using Bruker's ledbpgp2s pulse sequence, a diffusion delay of Δ = 75 ms, bipolar pulse duration of $\delta/2$ = 550 µs, Eddy current delay of 5 ms, gradient recovery delay of 0.20 ms, and sine bell-shaped shaped gradient pulses.

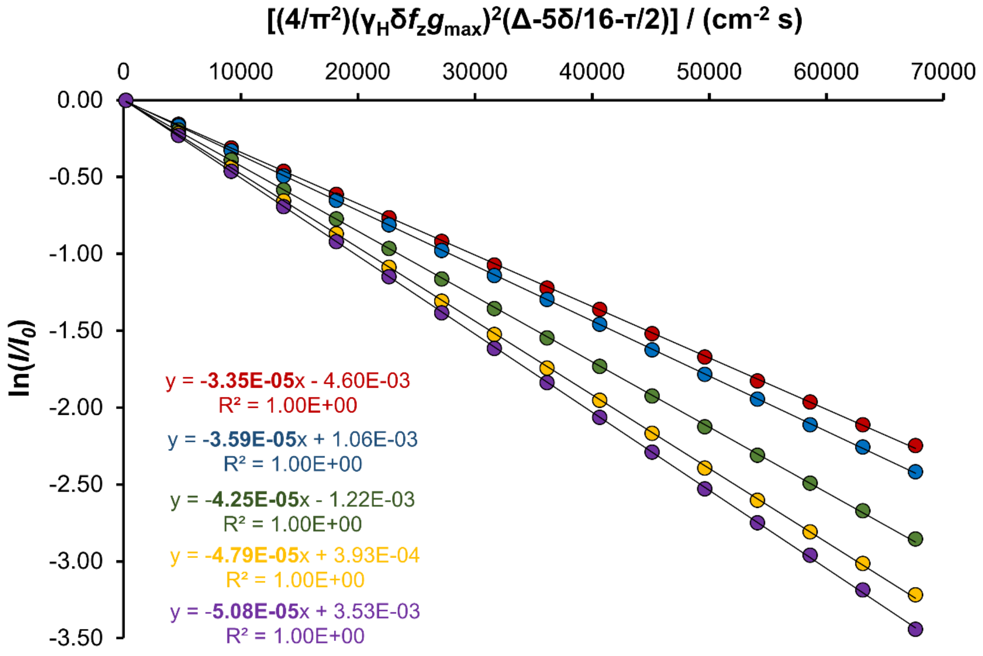

Figure S78: Linearised Stejskal-Tanner plots calculated from the stacked diffusion data shown in Figure S77, using integrals of resonances at δ_H = 0.90 ppm (TMBu **14**, s), 1.46 ppm (CyH **13**, s), 3.44 (Et_2O **12**, q), 3.77 ppm (1,3,5-TMB **15**, s) and 6.97 ppm (1,3,5-TIB **16**, s). The gradient of each trendline (bold) equals the (negative) translational self-diffusion coefficient of the corresponding internal diffusion standard (i.e., $-D_T$, in cm^2 s^{-1}). Excellent linearity (R^2 > 0.999) was observed for all resonances, indicating that diffusion was not perturbed significantly by convection currents.

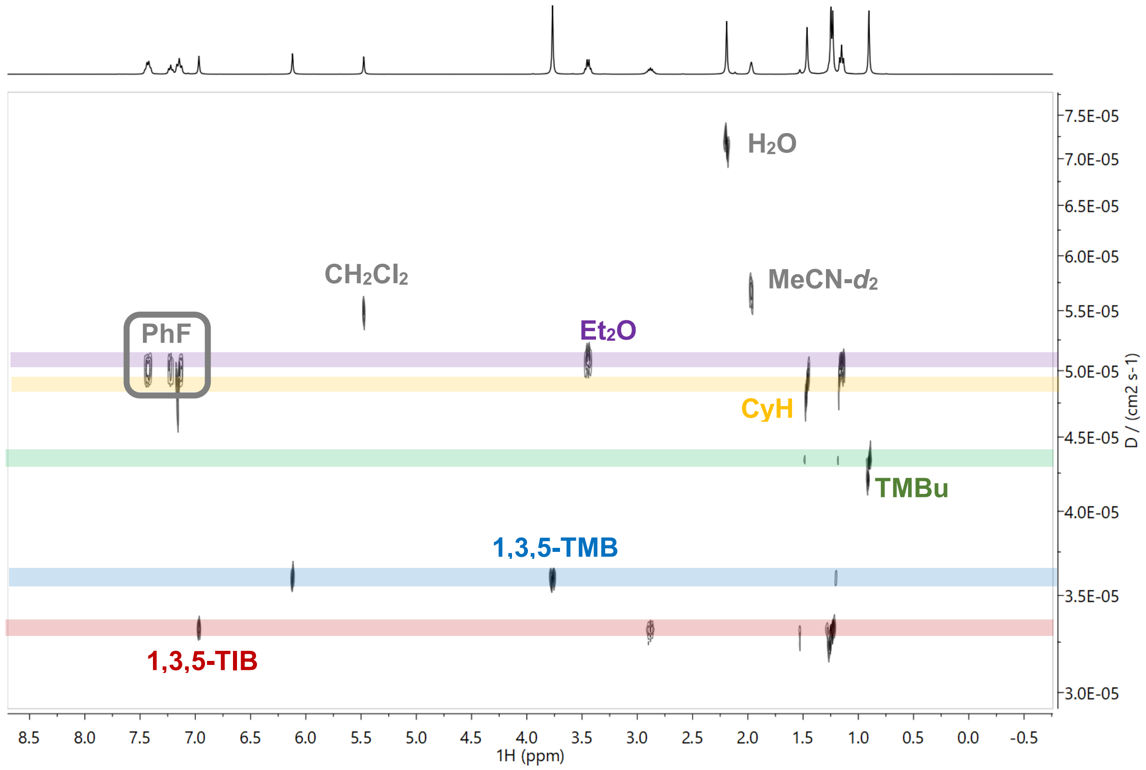

Figure S79: Pseudo-2D ^{1}H DOSY spectrum obtained following Bayesian transformation of the stacked 1D spectra in Figure S77. Key resonances for the five internal diffusion standards highlighted, alongside signals of other components (PhF, CH_2Cl_2), solvent (MeCN-d_2) and residual moisture.

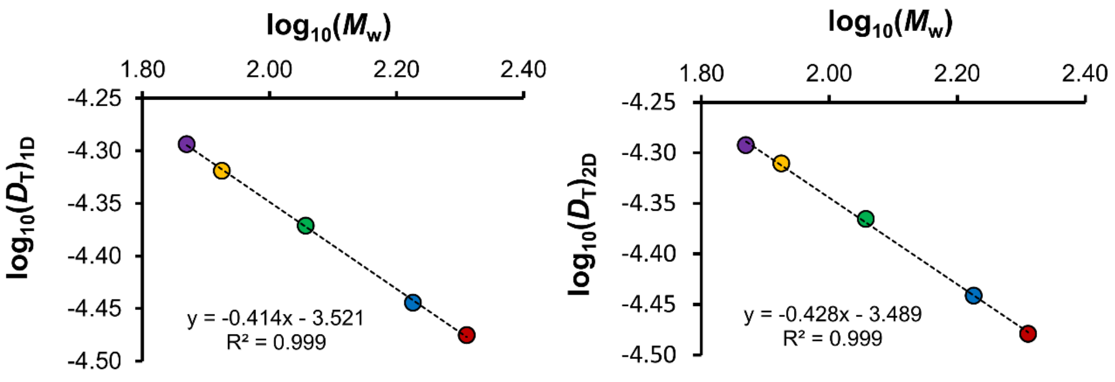

Figure S80: Logarithmic calibration curves calculated using diffusion coefficients (D_T) obtained by manual (1D; **Figure S78**) and automatic (2D; **Figure S79**) processing of diffusion data.

	M_w / (g mol^{-1})	$M_{w,1D}$ / (g mol^{-1})	$M_{w,2D}$ / (g mol^{-1})
Et_2O **12**	74.1	73.7	75.6
CyH **13**	84.2	84.8	83.4
TMBu **14**	114.1	113.4	112.0
1,3,5-TMB **15**	168.2	170.6	168.4
1,3,5-TIB **16**	204.4	202.4	206.1
	**RMSE / (g mol^{-1})**	1.5	1.4

Table S12: Calculated molecular weights for the five internal diffusion standards in MeCN-d_3 (Et_2O **12** (0.025 M), CyH **13** (0.015 M), TMBu **14** (0.010 M), 1,3,5-TMB **15** (0.015 M), 1,3,5-TIB **16** (0.015 M)) obtained following linear regression of $\log_{10}(D_T)$ vs $\log_{10}(M_w)$ (Figure S80). Diffusion coefficients extracted either manually (1D), via linearised Stejskal-Tanner plots, or automatically (2D), from pseudo-2D transformed ^{1}H DOSY spectra. First column shows actual molecular weights. RMSE = Root-mean-square error.

To assess the reproducibility of the internally calibrated DOSY experiments, and to explore their sensitivity towards key technical parameters, the reference sample was next reanalysed a total of six times, using the standard ledbpgp2s sequence, a diffusion delay of Δ = 75 ms, and variable number of increments in the diffusion dimension (n = 8, 16, 32) and ramping functions (linear, l; quadratic, q; exponential, exp). The calibration curves from all six experiments are shown overlaid in Figure S81, whilst the molecular weight estimates for the standards (denoted $M_{w[n,l/q/exp]}$) obtained in each case are summarised across Tables S13 – S15.

The average and standard deviations of the molecular weight estimates over all six experiments, summarised in Table S15, shows that internal calibration leads to molecular weight estimates that are indeed reproducible, and independent of common experimental parameters. With these findings in mind, all production-level ^{1}H DOSY experiments were undertaken, essentially arbitrarily, with quadratic ramping and 16 increments in the diffusion direction, affording equally spaced data points in linearised Stejskal-Tanner plots.

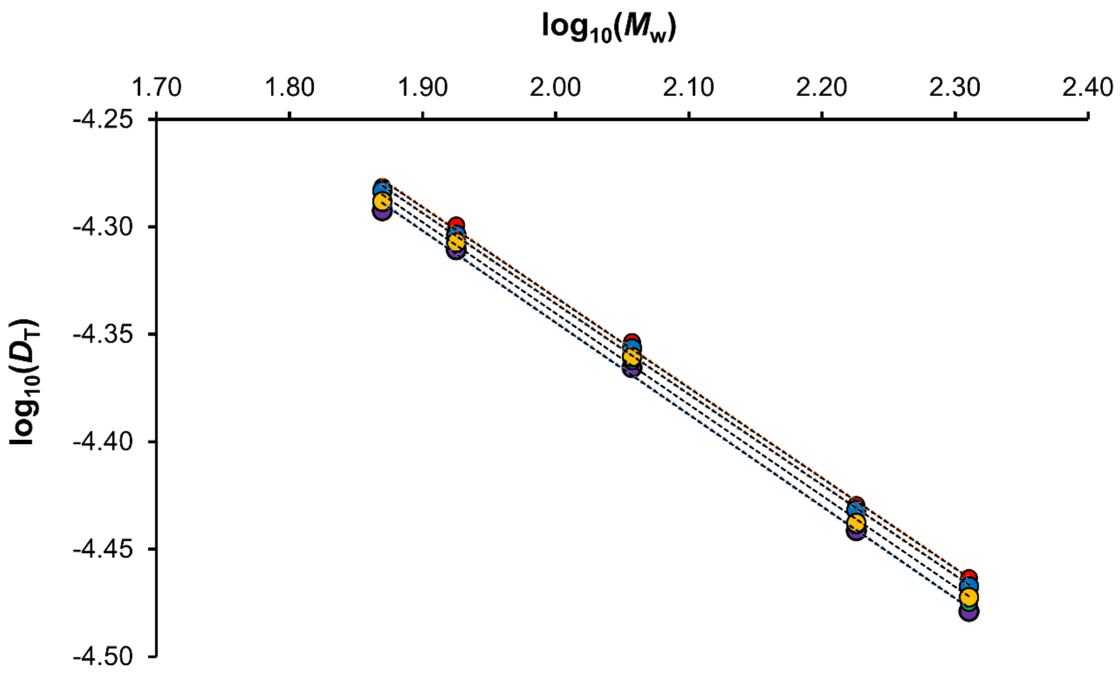

Figure S81: Six overlaid linear regressions of $\log_{10}(D_T)$ vs $\log_{10}(M_w)$, obtained from six independent ^{1}H DOSY analyses of a sample of Et_2O **12** (0.025 M), CyH **13** (0.015 M), TMBu **14** (0.010 M), 1,3,5-TMB **15** (0.015 M) and 1,3,5-TIB **16** (0.015 M) in MeCN-d_3. Diffusion coefficients in each case were extracted from pseudo-2D ^{1}H DOSY spectra and measured using a different gradient ramping function (l, q, exp; 16 increments) or number of diffusion increments (8, 16, 32; quadratic ramping), but otherwise identical acquisition parameters (ledbpgp2s; Δ = 75 ms, δ/2 = 550 μs, Eddy current delay of 5 ms, gradient recovery delay of 0.20 ms; Tables S13-S14).

	M_w / (g mol^{-1})	$M_{w[16,q]}$ / (g mol^{-1})	$M_{w[16,l]}$ / (g mol^{-1})	$M_{w[16,exp]}$ / (g mol^{-1})
Et_2O **12**	74.1	75.6	75.4	75.4
CyH **13**	84.2	83.4	83.5	83.6
TMBu **14**	114.1	112.0	112.0	111.7
1,3,5-TMB **15**	168.2	168.4	169.1	169.8
1,3,5-TIB **16**	204.4	206.1	205.4	204.9
	**RMSE / (g mol^{-1})**	1.4	1.3	1.5

Table 13: Calculated molecular weights for five internal diffusion standards in MeCN-d_3 (Et_2O **12** (0.025 M), CyH **13** (0.015 M), TMBu **14** (0.010 M), 1,3,5-TMB **15** (0.015 M), 1,3,5-TIB **16** (0.015 M)) obtained following three independent ^{1}H DOSY analyses of a single sample. Molecular weights determined by linear regression of $\log_{10}(D_T)$ vs $\log_{10}(M_w)$ in each case, with ^{1}H spectra acquired in 16 increments along the diffusion dimension with linear (l), quadratic (q) or exponential (exp) ramping between 5 % (f_z = 0.05) and 95 % (f_z = 0.95) of the maximum gradient strength of the probe. All other acquisition parameters were identical. First column shows actual molecular weights. RMSE = Root-mean-square error.

	M_w / (g mol^{-1})	$M_{w[16,q]}$ / (g mol^{-1})	$M_{w[32,q]}$ / (g mol^{-1})	$M_{w[8,q]}$ / (g mol^{-1})
Et_2O **12**	74.1	75.6	75.5	75.0
CyH **13**	84.2	83.4	83.2	83.9
TMBu **14**	114.1	112.0	112.1	112.1
1,3,5-TMB **15**	168.2	168.4	169.9	169.1
1,3,5-TIB **16**	204.4	206.1	204.6	205.2
	**RMSE / (g mol^{-1})**	1.4	1.4	1.1

Table S14: Calculated molecular weights for five internal diffusion standards in MeCN-d_3 (Et_2O **12** (0.025 M), CyH **13** (0.015 M), TMBu **14** (0.010 M), 1,3,5-TMB **15** (0.015 M), 1,3,5-TIB **16** (0.015 M)) obtained following three independent ^{1}H DOSY analyses of a single sample. Molecular weights determined by linear regression of $\log_{10}(D_T)$ vs $\log_{10}(M_w)$ in each case, with ^{1}H spectra acquired in 8, 16 or 32 increments along the diffusion dimension with quadratic ramping between 5 % (f_z = 0.05) and 95 % (f_z = 0.95) of the maximum gradient strength of the probe. All other acquisition parameters were identical. First column shows actual molecular weights. RMSE = Root-mean-square error.

	M_w / (g mol^{-1})	$M_{w,av}$ / (g mol^{-1})	σ_{Mw} / (g mol^{-1})
Et_2O **12**	74.1	75.2	0.5
CyH **13**	84.2	83.6	0.4
TMBu **14**	114.1	112.3	0.9
1,3,5-TMB **15**	168.2	169.1	0.7
1,3,5-TIB **16**	204.4	205.1	0.6

Table S15: Average molecular weights and standard deviations (σ_{Mw}) calculated for the five internal diffusion standards in MeCN-d_3 (Et_2O **12** (0.025 M), CyH **13** (0.015 M), TMBu **14** (0.010 M), 1,3,5-TMB **15** (0.015 M), 1,3,5-TIB **16** (0.015 M)). Averages and standard deviations calculated from six independent measurements of diffusion coefficients on a single sample using different ramping functions (l, q, exp) or diffusion increments (8, 16, 32) but otherwise identical acquisition parameters (Tables S13, S14).

S5.6. Discussion and figures

Single component measurements

Having established the validity of the ^{1}H DOSY NMR methodology, the diffusion coefficients of key species were measured *individually* at catalytically pertinent concentrations (0.050 M: 1,2,4-triazole **4a$_{H}$**, *p*-F-PhOH **6$_{H}$**, DBU **3**; 0.025 M: [$^{n}Bu_4N^+PF_6$], [$DBUH^+BPh_4^-$]), and their molecular weights estimated by internal calibration (Table S16). The measured molecular weights of **6$_{H}$** ($\delta M_w/M_w$ = + 7 %) and DBU **3** ($\delta M_w/M_w$ = + 1 %) were found to be in excellent agreement with the expected masses of the unassociated monomers, confirming the absence of any significant self-association or interference from the internal diffusion standards in MeCN.

In contrast, the molecular weights of both the tetra-*n*-butylammonium $^{n}Bu_4N^+$ ($\delta M_w/M_w$ = + 12 %) and $DBUH^+$ **3$_{H}^{+}$** cations ($\delta M_w/M_w$ = + 28 %) were moderately overestimated, for which several explanations might be proposed. One such argument may be that the effective molecular weights of the cations were increased by virtue of counteranion association (PF_6^-, BPh_4^-), yet this is seemingly inconsistent with the observations that: (i) the molecular weight of the tetraphenylborate anion BPh_4^- was moderately *underestimated* ($\delta M_w/M_w$ = - 12 %), and in any case independent of the countercation (**3$_{H}^{+}$,** $^{n}Bu_4N^+$); and (ii) the observed molecular weight of $DBUH^+$ was essentially independent of the counteranion (PF_6^-, BPh_4^-). Given that the linearity of the calibration curve was entirely unaffected by the presence of $^{n}Bu_4N^+$ and $DBUH^+$ **3$_{H}^{+}$**, a similar line of reasoning eliminates the possibility of intermolecular association between the cations and any of the internal standards. Furthermore, the high accuracy with which the molecular weight of un-ionised DBU **3** was estimated appears to exclude the possibility that a significant mismatch with the *geometries* of the internal standards is responsible for the overestimated molecular weight of $DBUH^+$ **3$_{H}^{+}$** (in any case, Stalke and co-workers have shown that diffusion coefficients of organic molecules with M_w = 120 – 200 g mol^{-1} diffuse at a rate that is largely independent of molecular geometry).[S12] No such comparison was possible for $^{n}Bu_4N^+$, making it difficult to rule out geometric mismatch as the cause of the molecular weight overestimation in this case.

Interestingly, the error in the molecular weight estimates of both $^{n}Bu_4N^+$ and $DBUH^+$ **3$_{H}^{+}$** is diminished *substantially* if both are treated explicitly as strongly monosolvated cations (i.e., $^{n}Bu_4N^+_{\{MeCN\}}$ and **3$_{H}^{+}$**$_{\{MeCN\}}$; Table S16), suggesting that strong solvation MeCN has a significant influence on the diffusion properties of these cations. Indeed, treating the $DBUH^+$ **3$_{H}^{+}$** cation in this way leads to almost quantitative agreement with experiment, as observed for neutral DBU **3**. Whilst it is difficult to completely discount a role for adventitious water in the apparent inflation of the cation, it is notable that the addition of excess H_2O (5 equiv., 0.125 M) to both [**3$_{H}^{+}$**PF_6^-] and [**3$_{H}^{+}$**BPh_4^-] had no discernible effect on the measured molecular weight of **3$_{H}^{+}$.**

Species	*M*w / (g mol-1)	*M*w (calc.) / (g mol-1)	Error / %
1,2,4-Triazole **4aH**	69.1	100.7 (74.4)	46 (8)
p-F-PhOH **6H**	112.1	120.4	7
DBU **3**	152.2	153.4	1
$^{n}Bu_4N^+$	242.5	271.0	12
$^{n}Bu_4N^+_{\{MeCN\}}$	283.6	"	-4
DBUH+ $\mathbf{3_H}^+$	153.2	194.4	28
$\mathbf{3_H}^+_{\{MeCN\}}$	193.3	"	1
BPh_4^-	319.2	298.2	-12

Table S16: Molecular weights of key neutral molecules (0.050 M) and ions (0.025 M) estimated by internally calibrated ^{1}H DOSY NMR in MeCN-d_3 solution (T = 293 K). Each mass was calculated on the basis of diffusion data obtained on *independent* samples, with the analyte present in each case alongside the five standard diffusion standards. $^{n}Bu_4N^+$ introduced as the hexafluorophosphate salt ([$^{n}Bu_4N^+$][PF_6^-] (0.025 M), BPh_4^- as [$\mathbf{3_H}^+$][BPh_4^-] (0.025 M), and $\mathbf{3_H}^+$ as both [$\mathbf{3_H}^+$][BPh_4^-] (0.025 M) and [$\mathbf{3_H}^+$][PF_6^-] (0.025 M). The predicted mass of $\mathbf{3_H}^+$ was found to be essentially independent of the counteranion (BPh_4^-, PF_6^-). The molecular weight of 1,2,4-triazole **4aH** was estimated in two ways: (i) using the five diffusion standards and full internal calibration; and (ii) the residual solvent signal (MeCN-d_2) as a single-point diffusion standard (bracketed). Error = $\{(M_w^{(calc)} - M_w)/M_w\}$ x 100 %.

Much larger errors were encountered in calculating the molecular weight of 1,2,4-triazole **4aH** by internal calibration: unlike **6H** and DBU **3**, the molecular weight of **4aH** was *significantly* overestimated ($\delta M_w/M_w$ = + 46 %), suggesting it diffuses *disproportionately* slower than the internal calibrants. Stalke and co-workers have highlighted similarly substantial discrepancies in estimating the molecular weights of other extremely small molecules (e.g., cyclopentane, THF, MTBE, TMS) by internally calibrated ^{1}H DOSY NMR, suggesting that this deviation is not a consequence of intermolecular association but of a fundamental disparity between the diffusion properties of the calibrants **12 – 16** (all dissipated spheres/ellipsoids, DSE; $\alpha_{DSE} \approx -0.6$) and **4aH** (presumably a compact sphere, CS; $\alpha_{CS} \approx -0.5$).[12] To account for the geometric differences between 1,2,4-triazole **4aH** and the internal calibrants – and for the fact that **4aH**, unlike the calibrants, is comparable in size to the solvent – the molecular weight was re-evaluated by direct comparison to the residual signal of MeCN-d_2. Assuming both MeCN and **4aH** diffuse as compact spheres ($\alpha_{CS} \approx -0.5$), that $D_{MeCN} = D$(MeCN-d_2) and $M_{MeCN} = M_w$(MeCN-h_3) = 41.1 g mol^{-1} (deuteration has a minimal effect on molecular volume), the molecular weight of 1,2,4-triazole **4aH** may then be estimated in accordance with

$$M_{TrzH} = M_{MeCN}\left(\frac{D_{TrzH}}{D_{MeCN}}\right)^{\frac{1}{\alpha_{CS}}} \qquad (S33)$$

By this approach, the deviation from the expected molecular weight of **4aH** was reduced *substantially* ($\delta M_w/M_w$ = + 8 %; Table S16), suggesting that **4aH** does not undergo any substantial self-association in MeCN (0.050 M).

^{1}H DOSY and homoconjugation

Having established the robustness of internally calibrated ^{1}H DOSY NMR for measuring the molecular weights of individual components in MeCN, further experiments were conducted to probe the interaction between multiple different components. The equilibrium between DBU **3** and *p*-F-PhOH $\mathbf{6_H}$ was studied first, by virtue of a ^{1}H DOSY NMR titration at fixed [$\mathbf{6_H}$]$_0$ (0.050 M); the evolution of M_w(**6**) as a function of [**3**] obtained from this titration is shown in Figure S82. In accordance with 1D ^{19}F NMR titration data, vide infra, the calculated molecular weight of **6** increased steadily following the addition of increasing quantities of **3**, stabilising at M_w(obs) ~ 230 g mol^{-1} after the addition of approximately 0.75 equivalents of **3** ([**3**]$_0$/[$\mathbf{6_H}$]$_0$ = 0.75); both the sub-stoichiometric saturation and limiting M_w(obs) are consistent with the formation of the first-order homoconjugate {**6-6**$_\mathbf{H}$}$^-$ (M_w = 223 g mol^{-1}; $\delta M_w/M_w \approx 3$ %). Inverse titrations with a fixed concentration of [**3**]$_0$ = 0.025 M and variable [$\mathbf{6_H}$]$_0$ displayed analogous behaviour, with the observed molecular weight of **3** increasing up to 2.0 equivs of $\mathbf{6_H}$ and apparently stabilising thereafter.

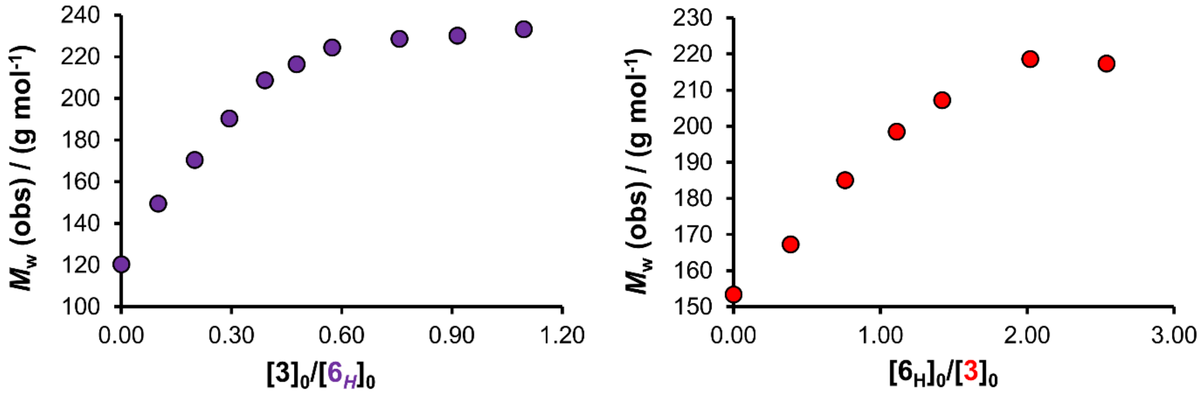

Figure S82: ^{1}H DOSY NMR titrations (T = 293 K) of: (A) $\mathbf{6_H}$ (0.050 M; fixed) with DBU **3** (0.005 – 0.050 M) in MeCN-d_3; and (B) DBU **3** (0.025 M; fixed) with $\mathbf{6_H}$ (0.005 – 0.063 M) in MeCN-d_3. In each case the molecular weight of the component at *constant concentration* was calculated by standard internal calibration.

Considering several conflicting observations, discerning the extent of ion-pairing between DBUH$^+$ $\mathbf{3_H^+}$ and the homoconjugate {**6-6**$_\mathbf{H}$}$^-$ by proved difficult. On the one hand, it is clear that the presence of super-stoichiometric $\mathbf{6_H}$ leads to molecular weight estimates for DBU **3**/$\mathbf{3_H^+}$ that are modestly but reproducibly greater than $\mathbf{3_H^+}$ salts with non-coordinating anions (*i.e.,* [$\mathbf{3_H^+}$][BPh$_4^-$]; [$\mathbf{3_H^+}$][PF$_6^-$], *vide supra),* suggesting some degree of ion-pairing with the homoconjugate (i.e., {**6-6**$_\mathbf{H}$}$^-_{\{\mathbf{DBUH}\}}$; that the measured masses of both components (i.e., **3** and **6**) at saturation ([**3**]$_0$/[$\mathbf{6_H}$]$_0$ > 0.50) are *substantially* lower than expected for the strongly ion-paired homoconjugate (M_w = 376 g mol^{-1}), however, indicates that ion-pairing is modest, with the speciation of the homoconjugate dominated by the free anion {**6-6**$_\mathbf{H}$}$^-$**.** However, this conclusion is in direct contrast to both experimental (*vide supra*) and computed (*vide infra*) ^{19}F chemical shifts, which suggest that DBUH$^+$ $\mathbf{3_H^+}$ complexation with the homoconjugate is strong, and that the ion-paired species ought to predominate at saturation.

This dichotomy was investigated more closely by subjecting solutions of: (i) independently synthesized $\{\mathbf{6\text{-}6_H}\}^-_{\{nBu4N\}}$ (0.025 M); and (ii) **3** (0.025 M) + $\mathbf{6_H}$ (0.050 M) to 1D ^{19}F NMR and ^{1}H DOSY NMR analysis under otherwise identical conditions (MeCN-*d*3, 20 ˚C). The measured molecular weights of the components in each solution are summarised in Table S17. Intriguingly, the molecular weight estimation for **6** was almost identical in both solutions – although slightly higher in $\{\mathbf{6\text{-}6_H}\}^-_{\{nBu4N\}}$, presumably due to the fact that 1:2 **3**:$\mathbf{6_H}$ does not lead to complete homoconjugation – indicating that the diffusion of the phenol homoconjugate $\{\mathbf{6\text{-}6_H}\}^-$ is apparently *independent* of the countercation. Both the molecular weights and phenolic chemical shift observed for solution (ii) were reproduced quantitatively upon addition of 1 equivalent of $[\mathbf{3_H}^+][PF_6^-]$ (0.025 M) to $\{\mathbf{6\text{-}6_H}\}^-_{\{nBu4N\}}$ (0.025 M) (solution i); independent addition of **3** (1 equiv, 0.025 M) to a separate sample of solution $\{\mathbf{6\text{-}6_H}\}^-_{\{nBu4N\}}$ (0.025 M), however, led to no change by either ^{19}F NMR or ^{1}H DOSY NMR (including the molecular weight of **3**), confirming the stability of the homoconjugate $\{\mathbf{6\text{-}6_H}\}^-$ towards further deprotonation. The addition of stoichiometric $\mathbf{6_H}$ (0.025 M) to $\{\mathbf{6\text{-}6_H}\}^-_{\{nBu4N\}}$ (0.025 M), which, barring second-order association, ought to lower the observed molecular weight significantly, in fact had the opposite effect, affording a material increase in the population-weighted molecular weight.

**Solution**	***Mw (calc.) / (g mol^{-1})***	***δF / ppm***
$\{\mathbf{6\text{-}6_H}\}^-_{\underline{\{nBu4N\}}}$	283	-
$\underline{\{\mathbf{6\text{-}6_H}\}^-}_{\{nBu4N\}}$	232	-134.9
$\underline{\mathbf{3}}$ + $\mathbf{6_H}$ (1 : 2)	222	-
**3** + $\underline{\mathbf{6_H}}$ (1 : 2)	223	-130.8
$\{\mathbf{6\text{-}6_H}\}^-_{\underline{\{nBu4N\}}}$ + $[\mathbf{3_H}^+][PF_6^-]$ (1 : 1)	274	-
$\underline{\{\mathbf{6\text{-}6_H}\}^-}_{\{nBu4N\}}$ + $[\mathbf{3_H}^+][PF_6^-]$ (1 : 1)	222	-130.9
$\{\mathbf{6\text{-}6_H}\}^-_{\{nBu4N\}}$ + $\underline{[\mathbf{3_H}^+]}[PF_6^-]$ (1 : 1)	217	-
$\{\mathbf{6\text{-}6_H}\}^-_{\underline{\{nBu4N\}}}$ + **3** (1 : 1)	274	-
$\underline{\{\mathbf{6\text{-}6_H}\}^-}_{\{nBu4N\}}$ + **3** (1 : 1)	226	-134.7
$\{\mathbf{6\text{-}6_H}\}^-_{\{nBu4N\}}$ + $\underline{\mathbf{3}}$ (1 : 1)	149	-
$\underline{\{\mathbf{6\text{-}6_H}\}^-}_{\{nBu4N\}}$ + $\underline{\mathbf{6_H}}$ (1 : 1)	246	-132.0

Table S17: Molecular weights of key species (underlined) estimated by internally calibrated ^{1}H DOSY NMR in MeCN-*d*3 solution (*T* = 293 K). Data obtained from a total of five independent solutions, including: (i) $\{\mathbf{6\text{-}6_H}\}^-_{\{nBu4N\}}$ (0.025 M); (ii) **3** (0.025 M) + $\mathbf{6_H}$ (0.050 M); (iii) $\{\mathbf{6\text{-}6_H}\}^-_{\{nBu4N\}}$ (0.025 M) + $[\mathbf{3_H}^+][PF_6^-]$ (0.025 M); (iv) $\{\mathbf{6\text{-}6_H}\}^-_{\{nBu4N\}}$ (0.025 M) + **3** (0.025 M); and (v) $\{\mathbf{6\text{-}6_H}\}^-_{\{nBu4N\}}$ (0.025 M) + $\mathbf{6_H}$ (0.025 M). Limiting shift of **6** (0.050 M) in the presence of excess **3** is $\delta_F \approx$ -131.7 ppm, is considerably downfield of the same shift for $\{\mathbf{6\text{-}6_H}\}^-_{\{nBu4N\}}$ ($\delta_F \approx$ -134.9 ppm). 19F shifts reference to 1-F-naphthalene (δ_F = -125.20 ppm)

In a further attempt to probe the degree of ion-pairing between DBUH$^+$ **3**$_{\mathbf{H}}$$^+$ and the homoconjugate {**6-6**$_{\mathbf{H}}$}$^-$, a stock solution of {**6-6**$_{\mathbf{H}}$}$^-_{\mathbf{\{nBu4N\}}}$ + [**3**$_{\mathbf{H}}$$^+$][PF$_6$$^-$] (1:1, 0.025 M, MeCN-$d_3$) was prepared, with the appropriate internal diffusion standards, and serially diluted to afford a series of solutions of equal stoichiometries but varying global concentrations; each solution was in turn subjected to molecular weight analysis by internally calibrated ^{1}H DOSY NMR, leading to Figure S83 (M_w(obs) vs [**6**$_{\mathbf{H}}$]$_0$). Significant reductions in the molecular weights of both DBU **3** and **6** were observed upon dilution, as expected for an associative equilibrium. Critically, whilst the molecular weights of DBU **3** and **6** almost identical for the highest concentration solution ([**6**$_{\mathbf{H}}$]$_0$ = 0.050 M), serial dilution led to disproportionate decreases in these estimates, such that for [**6**$_{\mathbf{H}}$]$_0$ < 0.015 M significantly different molecular weights were measured for the two species. The *convergence* of the two molecular weights in this way is typically interpreted as a signature of ion-pairing, and suggests that the two components effectively diffuse as a single species at [**6**$_{\mathbf{H}}$]$_0$ > 0.050 M (i.e. the speciation of both components is dominated by the ion-pair). It is not yet clear, however, how this might be reconciled with the gross disparity between the limiting molecular weight observed by ^{1}H DOSY and the theoretical molecular weight of a strongly associated complex {**6-6**$_{\mathbf{H}}$}$^-_{\mathbf{\{DBUH+\}}}$ (M_w = 376 g mol^{-1}).

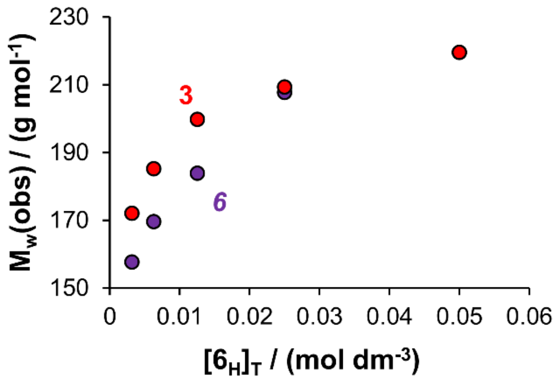

Figure S83: Internally calibrated ^{1}H DOSY NMR analysis of a sequentially diluted solution of {**6-6**$_{\mathbf{H}}$}$^-_{\mathbf{\{nBu4N\}}}$ (0.025 M) + [**3**$_{\mathbf{H}}$$^+$][PF$_6$$^-$] (1:1, 0.025 M) in MeCN-d_3 (T = 293 K).

S6. $^{1}H/^{19}F$ NMR Titrations

S6.1. Overview

To explore the various dynamic equilibria established in solution with different azoles, bases and **6$_H$**, a number of acid-base titrations were undertaken in MeCN and THF. Titrations were monitored by ^{1}H or ^{19}F NMR spectroscopy, and deliberately conducted at catalytically pertinent concentrations (i.e., 5 – 100 mM) for comparison to kinetic data. In each titration, small aliquots of a neutral organic base – typically, but not invariably, DBU **3** (*vide infra*) – were dispensed sequentially to a larger, more dilute solution of a neutral organic acid, in the form of *p*-F-PhOH **6$_H$** or **4a$_H$**. In all cases a single time-averaged set of resonances was observed for both the acid and the base, with the position of each resonance reflecting the speciation-weighted average of all exchanging species. Phenomenological equilibrium constants (K^c) in each case were determined by monitoring the chemical shift of a key ^{1}H (MeCN-d_3/THF-d_8) or ^{19}F (MeCN-h_3/THF-h_8) resonance from the *acid* and fitting to an appropriate equilibrium model. By virtue of the method of solution preparation, the concentration of the acid was held constant throughout each titration (typically ≈ 0.050 M).

S6.2. Typical titration

Data acquisition

A solution of *p*-F-PhOH **6$_H$** (0.0280 g, 0.250 mmol, 0.0500 M) and 1-F-naphthalene (16.2 µL, 0.125 mmol, 0.0250 M) was prepared in anhydrous MeCN (5 mL) under ambient conditions, using volumetric glassware (Solution 1) and an analytical balance (0.01 mg). A portion of this stock solution (500 µL) was transferred to a 5 mm borosilicate NMR tube. To ensure constant concentrations of *p*-F-PhOH **6$_H$** and 1-F-naphthalene throughout the titration, solution 1 was in turn used to prepare a solution of DBU **3** (74.6 µL, 0.500 mmol, 0.50 M, 1 mL; solution 2) in a separate volumetric flask (i.e., [**6$_H$**]$_T$ = 0.050 M). Both stock solutions were prepared immediately (< 1 h) prior to the titration.

Prior to the addition of any DBU **3**, the NMR tube containing solution 1 was loaded into the NMR probehead (T_{NMR} = 20.0 °C; T_{IRM} = 20 ± 2 °C), after which the probe was tuned and matched to ^{1}H, the sample shimmed with the lock off (lock was used for titrations in MeCN-d_3), the receiver gain optimised automatically, and a quantitative ^{1}H NMR spectrum acquired without solvent suppression (30° excitation pulse, ns = 4, t_{AQ} = 4.0 s, t_{D1} = 10 s). The probe was subsequently retuned and matched to ^{19}F, the receiver gain optimised automatically, and a quantitative ^{19}F spectrum acquired in a single transient. After the acquisition of the ^{19}F spectrum, the sample was ejected from the probe, uncapped in a proximate fumehood, and a small aliquot of solution 2 (5 µL) was added, whereafter the tube was re-capped, inverted three times, shaken vigorously for 10 s, loaded back into the NMR probehead, and analysed as previously (^{1}H *and* ^{19}F analysis). This sequence was repeated for a further 11 aliquots of the same volume (5 µL), six aliquots of 10 µL, and 3 aliquots of 20 µL. In each case a single time-

averaged ^{19}F signal was observed from **6**. To ensure optimal lineshapes and accurate chemical shifts, the sample was reshimmed following the addition of each aliquot.

At each titration point, the speciation-weighted ^{19}F ($\delta_{F,obs}$) shift of **6** was measured relative to that of the internal standard ($\delta_{F,IS}$ = -125.20 ppm), 1-F-naphthalene, whilst the total concentrations of **6** (constant) and DBU **3** ([**3**]$_T$) were determined by direct (sum) integration and normalisation against 1-F-naphthalene (c_{IS} = 0.0250 M) in the ^{1}H spectrum. The ^{19}F chemical shift of un-ionised **6$_H$** (δ_F[**6$_H$**]) was determined in the first ^{19}F NMR spectrum of the titration, in the absence of any **3** (solution 1).

Data analysis

The resulting isotherm, of δ_F[obs] as a function of [**3**]$_T$/[**6$_H$**]$_T$, was subjected to least-squares fitting in accordance with an appropriate equilibrium model in Excel, with the underlying equilibrium constant(s) optimised by minimising the sum of squares (δ_F[obs] – δ_F[calc])^2 across all titration points.

S6.3. Equilibrium models

For each titration point, δ_F[**calc**] was calculated either analytically or numerically, *vide infra*, in accordance with a specified *equilibrium model*, using experimentally measured values of [Base]$_T$ and [Acid]$_T$ and a given equilibrium constant (or pair of constants). A range of equilibrium models, including but not limited to elementary (1:1) proton transfer, were considered for any given isotherm.

For each such model, equations for the acid speciation – either **4a$_H$** or **6$_H$**, depending on the titration – were derived by hand. Solving these equations – either analytically or numerically – in turn afforded the simulated concentrations of each acid-derived species at a given titration point, for given a set of initial conditions ([Acid]$_T$, [Base]$_T$) and phenomenological equilibrium constants ({$K^C{}_i$}). With simulated concentrations for each acid-derived species in hand, δ_F[calc] was then calculated by mole-fraction weighting, using limiting chemical shifts measured either by experiment or computation.

Various equilibrium models, including pairs of coupled equilibria to handle homoconjugation, were considered in fitting experimental isotherms. These models are summarised below, with the acid in general denoted by **AH** and the base by **B**, alongside key mathematical derivations.

Titration model 1:

$$\mathbf{AH} + \mathbf{B} \underset{}{\overset{K_{PT}}{\rightleftharpoons}} \mathbf{A}^{\ominus} + \mathbf{BH}^{\oplus}$$

The phenomenological concentration equilibrium constant, K^{C}_{PT}, for an elementary proton transfer reaction is:

$$K_{PT}^{C} = \frac{[\mathrm{A}^{-}][\mathrm{BH}^{+}]}{[\mathrm{AH}][\mathrm{B}]} \qquad (S34)$$

For a total acid concentration $[\mathrm{AH}]_{\mathrm{T}}$ and total base concentration $[\mathrm{B}]_{\mathrm{T}}$, the equilibrium concentrations [AH], $[\mathrm{A}^{-}]$, [B], and $[\mathrm{BH}^{+}]$ are defined by mass balance and electroneutrality considerations in accordance with:

$$[\mathrm{B}]_{\mathrm{T}} = [\mathrm{B}] + [\mathrm{BH}^{+}] \qquad (S35)$$

$$[\mathrm{AH}]_{\mathrm{T}} = [\mathrm{AH}] + [\mathrm{A}^{-}] \qquad (S36)$$

$$[\mathrm{A}^{-}] = [\mathrm{BH}^{+}] \qquad (S37)$$

Substitution and rearrangement affords a quadratic equation in $[\mathrm{A}^{-}]$ ($[\mathrm{AH}]_{\mathrm{T}}$ was kept constant throughout each titration, whilst $[\mathrm{B}]_{\mathrm{T}}$ increased between each successive titration point):

$$K_{PT}^{C} = \frac{[\mathrm{A}^{-}]^{2}}{([\mathrm{AH}]_{\mathrm{T}} - [\mathrm{A}^{-}])([\mathrm{B}]_{\mathrm{T}} - [\mathrm{A}^{-}])} \qquad (S38)$$

$$(1 - K_{PT}^{C})[\mathrm{A}^{-}]^{2} + K_{PT}^{C}([\mathrm{B}]_{\mathrm{T}} + [\mathrm{AH}]_{\mathrm{T}})[\mathrm{A}^{-}] - K_{PT}^{C}[\mathrm{B}]_{\mathrm{T}}[\mathrm{AH}]_{\mathrm{T}} = 0 \qquad (S39)$$

which can in turn be solved analytically, yielding:

$$[\mathrm{A}^{-}] = \frac{-K_{PT}^{C}([\mathrm{B}]_{\mathrm{T}} + [\mathrm{AH}]_{\mathrm{T}}) \pm \sqrt{(K_{PT}^{C})^{2}([\mathrm{B}]_{\mathrm{T}} + [\mathrm{AH}]_{\mathrm{T}})^{2} + 4(K_{PT}^{C})^{2}(1 - K_{PT}^{C})[\mathrm{B}]_{\mathrm{T}}[\mathrm{AH}]_{\mathrm{T}}}}{2(1 - K_{PT}^{C})} \qquad (S40)$$

The observed chemical shift of the acid under a fast exchange regime, $\delta^{(A)}_{calc}$, will be the mole-fraction weighted average of the intrinsic shifts δ_{AH} and δ_{A-}, such that:

$$\delta_{calc}^{(A)} = (1 - x_{A^{-}})\delta_{AH} + x_{A^{-}}\delta_{A-} \qquad (S41)$$

$$[\mathrm{A}^{-}] = \left(\frac{\delta_{calc}^{(A)} - \delta_{AH}}{\delta_{A^{-}} - \delta_{AH}}\right)[\mathrm{AH}]_{\mathrm{T}} \qquad (S42)$$

Titration model 2:

$$\mathbf{AH} + \mathbf{B} \xrightleftharpoons{K_{PTIP}} \mathbf{A}^{\ominus}\ \mathbf{BH}^{\oplus}$$

The phenomenological concentration equilibrium constant, K^{C}_{PTIP}, for an elementary proton transfer reaction affording an intimate ion-pair directly is:

$$K^{C}_{PTIP} = \frac{[\mathrm{BH^+:A^-}]}{[\mathrm{AH}][\mathrm{B}]} \qquad (S43)$$

For a total acid concentration $[\mathrm{AH}]_\mathrm{T}$ and total base concentration $[\mathrm{B}]_\mathrm{T}$, the equilibrium concentrations [AH], [B], and $[\mathrm{BH^+:A^-}]$ are defined by mass balance considerations:

$$[\mathrm{B}]_\mathrm{T} = [\mathrm{B}] + [\mathrm{BH^+:A^-}] \qquad (S44)$$

$$[\mathrm{AH}]_\mathrm{T} = [\mathrm{AH}] + [\mathrm{BH^+:A^-}] \qquad (S45)$$

Substitution and rearrangement affords a quadratic equation in $[\mathrm{BH^+:A^-}]$ ($[\mathrm{AH}]_\mathrm{T}$ was kept constant throughout each titration, whilst $[\mathrm{B}]_\mathrm{T}$ increased between each successive titration point):

$$K^{C}_{PTIP} = \frac{[\mathrm{BH^+:A^-}]}{([\mathrm{B}]_\mathrm{T} - [\mathrm{BH^+:A^-}])([\mathrm{AH}]_\mathrm{T} - [\mathrm{BH^+:A^-}])} \qquad (S46)$$

$$K^{C}_{PTIP}[\mathrm{BH^+:A^-}]^2 - (1 + K^{C}_{PTIP}[\mathrm{B}]_\mathrm{T} + K^{C}_{PTIP}[\mathrm{AH}]_\mathrm{T})[\mathrm{BH^+:A^-}] + K^{C}_{PTIP}[\mathrm{B}]_\mathrm{T}[\mathrm{AH}]_\mathrm{T} = 0 \qquad (S47)$$

which can in turn be solved analytically, yielding:

$$[\mathbf{BH^+:A^-}] = \frac{\left(1 + K^{C}_{PTIP}[\mathrm{B}]_\mathrm{T} + K^{C}_{PTIP}[\mathrm{AH}]_\mathrm{T}\right) \pm \sqrt{(1 + K^{C}_{PTIP}[\mathrm{B}]_\mathrm{T} + K^{C}_{PTIP}[\mathrm{AH}]_\mathrm{T})^2 - 4\left({K^{C}_{PTIP}}^2\right)[\mathrm{B}]_\mathrm{T}[\mathrm{AH}]_\mathrm{T}}}{2K^{C}_{PTIP}} \qquad (S48)$$

The observed chemical shift of the acid under a fast exchange regime, $\delta^{(A)}_{calc}$, will be the mole-fraction weighted average of the intrinsic shifts $\delta_{\{AH\}}$ and $\delta_{\{BH^+:A^-\}}$, such that:

$$\delta^{(A)}_{calc} = \left(1 - x_{\{BH^+:A^-\}}\right)\delta_{AH} + x_{\{BH^+:A^-\}}\delta_{\{BH^+:A^-\}} \qquad (S49)$$

$$[\mathrm{BH^+:A^-}] = \left(\frac{\delta^{(A)}_{calc} - \delta_{AH}}{\delta_{\{BH^+:A^-\}} - \delta_{AH}}\right)[\mathrm{AH}]_\mathrm{T} \qquad (S50)$$

Titration model 3:

$$2\,\mathbf{AH} + \mathbf{B} \xrightleftharpoons{K_{PTHC}} \mathbf{AH\text{--}A}^{\ominus} + \mathbf{BH}^{\oplus}$$

The phenomenological concentration equilibrium constant, K^{C}_{PTHC}, for the formally concerted formation of an unbound anionic homoconjugate is:

$$K^{C}_{PTHC} = \frac{[\mathrm{AH{:}A^-}][\mathrm{BH^+}]}{[\mathrm{AH}]^2[\mathrm{B}]} \tag{S51}$$

For a total acid concentration $[\mathrm{AH}]_\mathrm{T}$ and total base concentration $[\mathrm{B}]_\mathrm{T}$, the equilibrium concentrations [AH], $[\mathrm{AH{:}A^-}]$, [B], and $[\mathrm{BH^+}]$ are defined by mass balance and electroneutrality considerations:

$$[\mathrm{B}]_\mathrm{T} = [\mathrm{B}] + [\mathrm{BH^+}] \tag{S52}$$

$$[\mathrm{AH}]_\mathrm{T} = [\mathrm{AH}] + 2[\mathrm{AH{:}A^-}] \tag{S53}$$

$$[\mathrm{BH^+}] = [\mathrm{AH{:}A^-}] \tag{S54}$$

Substitution and rearrangement affords a cubic equation in $[\mathrm{AH{:}A^-}]$ ($[\mathrm{AH}]_\mathrm{T}$ was kept constant throughout each titration, whilst $[\mathrm{B}]_\mathrm{T}$ increased between each successive titration point):

$$K^{C}_{PTHC} = \frac{[\mathrm{AH{:}A^-}]^2}{([B]_T - [\mathrm{AH{:}A^-}])([\mathrm{AH}]_\mathrm{T} - 2[\mathrm{AH{:}A^-}])^2} \tag{S55}$$

$$A[\mathrm{AH{:}A^-}]^3 + B[\mathrm{AH{:}A^-}]^2 + C[\mathrm{AH{:}A^-}] + D = 0 \tag{S56}$$

with the polynomial coefficients A, B, C and D. The cubic equation can be solved analytically or numerically.

$$A = 4K^{C}_{PTHC} \qquad B = 1 - 4K^{C}_{PTHC}[\mathrm{B}]_\mathrm{T} - 4K^{C}_{PTHC}[\mathrm{AH}]_\mathrm{T} \tag{S57}$$

$$C = K^{C}_{PTHC}[\mathrm{AH}]_\mathrm{T}(4[\mathrm{B}]_\mathrm{T} + [\mathrm{AH}]_\mathrm{T}) \qquad D = -K^{C}_{PTHC}[\mathrm{B}]_\mathrm{T}[\mathrm{AH}]_\mathrm{T}^2$$

The observed chemical shift of the acid under a fast exchange regime, $\delta^{(A)}_{calc}$, will be the mole-fraction weighted average of the intrinsic shifts $\delta_{\{AH\}}$ and $\delta_{\{AH:A^-\}}$, such that:

$$\delta^{(A)}_{calc} = \delta_{AH}x_{AH} + 2\delta_{\{\mathrm{AH{:}A^-}\}}x_{\{\mathrm{AH{:}A^-}\}}, \quad x_i = \frac{[\mathrm{i}]}{[\mathrm{AH}]_\mathrm{T}} \tag{S58}$$

$$x_{AH} + 2x_{\{\mathrm{AH{:}A^-}\}} = 1 \tag{S59}$$

$$[\mathrm{AH{:}A^-}] = \left(\frac{1}{2}\right)\left(\frac{\delta^{(A)}_{calc} - \delta_{AH}}{\delta_{\{\mathrm{AH{:}A}\}^-} - \delta_{AH}}\right)[\mathrm{AH}]_\mathrm{T} \tag{S60}$$

Titration model 4:

$$2\,\mathbf{AH} + \mathbf{B} \xrightleftharpoons{K_{PTHCIP}} \mathbf{AH{-}{-}A^{\ominus}\ BH^{\oplus}}$$

The phenomenological concentration equilibrium constant, K^{C}_{PTHCIP}, for the formally concerted formation of an ion-paired anionic homoconjugate is:

$$K^{C}_{PTHCIP} = \frac{[\mathrm{AH{:}A^-{:}BH^+}]}{[\mathrm{AH}]^2[\mathrm{B}]} \qquad (S61)$$

For a total acid concentration $[\mathrm{AH}]_{\mathrm{T}}$ and total base concentration $[\mathrm{B}]_{\mathrm{T}}$, the equilibrium concentrations [AH], $[\mathrm{AH{:}A^{-}{:}BH^{+}}]$, and [B] are defined by mass balance considerations:

$$[\mathrm{B}]_{\mathrm{T}} = [\mathrm{B}] + [\mathrm{AH{:}A^-{:}BH^+}] \qquad (S62)$$

$$[\mathrm{AH}]_{\mathrm{T}} = [\mathrm{AH}] + 2[\mathrm{AH{:}A^-{:}BH^+}] \qquad (S63)$$

Substitution and rearrangement affords a cubic equation in $[\mathrm{AH{:}A^{-}{:}BH^{+}}]$ ($[\mathrm{AH}]_{\mathrm{T}}$ was kept constant throughout each titration, whilst $[\mathrm{B}]_{\mathrm{T}}$ increased between each successive titration point):

$$K^{C}_{PTHCIP} = \frac{[\mathrm{AH{:}A^-{:}BH^+}]}{([B]_T - [\mathrm{AH{:}A^-{:}BH^+}])([\mathrm{AH}]_{\mathrm{T}} - 2[\mathrm{AH{:}A^-{:}BH^+}])^2} \qquad (S64)$$

$$A[\mathrm{AH{:}A^-{:}BH^+}]^3 + B[\mathrm{AH{:}A^-{:}BH^+}]^2 + C[\mathrm{AH{:}A^-{:}BH^+}] + D = 0 \qquad (S65)$$

with the polynomial coefficients A, B, C and D. The cubic equation can be solved analytically or numerically.

$$A = 4K^{C}_{PTHCIP} \qquad B = -4K^{C}_{PTHCIP}([\mathrm{B}]_{\mathrm{T}} + [\mathrm{AH}]_{\mathrm{T}}) \qquad (S66)$$

$$C = 1 + K^{C}_{PTHCIP}[\mathrm{AH}]_{\mathrm{T}}(4[\mathrm{B}]_{\mathrm{T}} + [\mathrm{AH}]_{\mathrm{T}}) \qquad D = -K^{C}_{PTHCIP}[\mathrm{B}]_{\mathrm{T}}[\mathrm{AH}]^2_{\mathrm{T}}$$

The observed chemical shift of the acid under a fast exchange regime, $\delta^{(A)}_{\mathrm{calc}}$, will be the mole-fraction weighted average of the intrinsic shifts $\delta_{\{\mathrm{AH}\}}$ and $\delta_{\{\mathrm{AH{:}A\text{-}{:}BH+}\}}$, such that:

$$\delta^{(A)}_{calc} = \delta_{AH}x_{AH} + 2\delta_{\{\mathrm{AH{:}A^-{:}BH^+}\}}x_{\{\mathrm{AH{:}A^-}{:}BH^+\}}, \quad x_i = \frac{[\mathrm{i}]}{[\mathrm{AH}]_{\mathrm{T}}} \qquad (S67)$$

$$x_{AH} + 2x_{\{\mathrm{AH{:}A^-}{:}BH^+\}} = 1 \qquad (S68)$$

$$[\mathrm{AH{:}A^-{:}BH^+}] = \left(\frac{1}{2}\right)\left(\frac{\delta^{(A)}_{calc} - \delta_{AH}}{\delta_{\{\mathrm{AH{:}A^-}{:}BH^+\}} - \delta_{AH}}\right)[\mathrm{AH}]_{\mathrm{T}} \qquad (S69)$$

Titration model 5:

$$\mathbf{AH} + \mathbf{B} \underset{}{\overset{K_{\mathbf{PTIP}}}{\rightleftharpoons}} \mathbf{A}^{\ominus}\ \mathbf{BH}^{\oplus}$$

$$\mathbf{AH} + \mathbf{A}^{\ominus}\ \mathbf{BH}^{\oplus} \overset{K_{\mathbf{HC1s}}}{\rightleftharpoons} \mathbf{AH{-}{-}A}^{\ominus} + \mathbf{BH}^{\oplus}$$

If ion-pairing and homoconjugation are competitive, and the speciation of AH is finely balanced, simulations of experimental isotherms require models with multiple, coupled equilibria. In the model above, there are two phenomenological concentration equilibrium constants: K^{C}_{PTIP}, for the formally concerted formation of the ion-pair; and (ii) K^{C}_{HC1s}, for the formally substitutive first-order homoconjugation of A^-, in which AH competes with BH^+ for hydrogen-bonding with A^-.

$$K^{C}_{PTIP} = \frac{[\mathrm{BH^+}{:}\,\mathrm{A^-}]}{[\mathrm{AH}][\mathrm{B}]} \tag{S70}$$

$$K^{C}_{HC1s} = \frac{[\mathrm{AH}{:}\,\mathrm{A^-}][\mathrm{BH^+}]}{[\mathrm{AH}][\mathrm{BH^+}{:}\,\mathrm{A^-}]} \tag{S71}$$

For a total acid concentration $[\mathrm{AH}]_\mathrm{T}$ and total base concentration $[\mathrm{B}]_\mathrm{T}$, the equilibrium concentrations [AH], $[\mathrm{AH{:}A^-}]$, $[\mathrm{BH^+{:}A^-}]$, $[\mathrm{BH^+}]$ and [B] are defined by mass balance and electroneutrality considerations:

$$[\mathrm{B}]_\mathrm{T} = [\mathrm{B}] + [\mathrm{BH^+}] + [\mathrm{BH^+}{:}\,\mathrm{A^-}] \tag{S72}$$

$$[\mathrm{AH}]_\mathrm{T} = [\mathrm{AH}] + [\mathrm{BH^+}{:}\,\mathrm{A^-}] + 2[\mathrm{AH}{:}\,\mathrm{A^-}] \tag{S73}$$

$$[\mathrm{BH^+}] = [\mathrm{AH}{:}\,\mathrm{A^-}] \tag{S74}$$

Substitution affords a pair of simultaneous equations in [AH] and $[\mathrm{AH{:}A^-}]$:

$$K^{C}_{PTIP} = \frac{[\mathrm{AH}]_\mathrm{T} - [\mathrm{AH}] - 2[\mathrm{AH}{:}\,\mathrm{A^-}]}{[\mathrm{AH}]([\mathrm{B}]_\mathrm{T} - [\mathrm{AH}]_\mathrm{T} + [\mathrm{AH}] + [\mathrm{AH}{:}\,\mathrm{A^-}]\ )} \tag{S75}$$

$$K^{C}_{HC1s} = \frac{[\mathrm{AH}{:}\,\mathrm{A^-}]^2}{[\mathrm{AH}]([\mathrm{AH}]_\mathrm{T} - [\mathrm{AH}] - 2[\mathrm{AH}{:}\,\mathrm{A^-}])} \tag{S76}$$

Extensive rearrangement in turn gives a quartic equation in [AH] ($[\mathrm{AH}]_\mathrm{T}$ was kept constant throughout each titration, whilst $[\mathrm{B}]_\mathrm{T}$ increased between each successive titration point):

$$A[\mathrm{AH}]^4 + B[\mathrm{AH}]^3 + C[\mathrm{AH}]^2 + D[\mathrm{AH}] + E = 0 \tag{S77}$$

with the polynomial coefficients A, B, C, D and E. The quartic equation can be solved analytically or numerically.

For notational simplicity, $K^{C}_{PTIP} = K_1$ and $K^{C}_{HC1s} = K_2$ in the expressions below:

$$A = {K_1}^2(1 - K_2) \quad (S78)$$

$$B = K_1(2 + 2K_1[\mathrm{B}]_\mathrm{T} - 2K_1[\mathrm{AH}]_\mathrm{T} - 2K_1K_2[\mathrm{B}]_\mathrm{T} + K_1K_2[\mathrm{AH}]_\mathrm{T} - 2K_2) \quad (S79)$$

$$C = {K_1}^2{[\mathrm{AH}]_\mathrm{T}}^2 - 2{K_1}^2[\mathrm{AH}]_\mathrm{T}[\mathrm{B}]_\mathrm{T} + {K_1}^2{[\mathrm{B}]_\mathrm{T}}^2 - 4K_1[\mathrm{AH}]_\mathrm{T} + 2K_1[\mathrm{B}]_\mathrm{T} + 1 - 4K_1K_2[\mathrm{B}]_\mathrm{T} + 2K_1K_2[\mathrm{AH}]_\mathrm{T} \quad (S80)$$

$$D = 2[\mathrm{AH}]_\mathrm{T}(K_1[\mathrm{AH}]_\mathrm{T} - K_1[\mathrm{B}]_\mathrm{T} - 1) \quad (S81)$$

$$E = {[\mathrm{AH}]_\mathrm{T}}^2 \quad (S82)$$

The observed chemical shift of the acid under a fast exchange regime, $\delta^{(A)}_{calc}$, will be the mole-fraction weighted average of the intrinsic shifts $\delta_{\{AH\}}$, $\delta_{\{AH:A-\}}$, and $\delta_{\{BH+:A-\}}$, such that:

$$\delta^{(A)}_{calc} = \delta_{AH}x_{AH} + \delta_{\{\mathrm{BH^+:A^-}\}}x_{\{\mathrm{BH^+:A^-}\}} + 2\delta_{\{\mathrm{AH:A^-}\}}x_{\{\mathrm{AH:A^-}\}}, \quad x_i = \frac{[i]}{[AH]_T} \quad (S83)$$

$$x_{AH} + x_{\{\mathrm{BH^+:A^-}\}} + 2x_{\{\mathrm{AH:A^-}\}} = 1 \quad (S84)$$

Assuming the intrinsic shift $\delta_{\{AH:A-\}}$ to be a weighted average of the other two intrinsic shifts, with a single weighting coefficient *w* (*vide supra*), then:

$$\delta_{\{\mathrm{AH:A^-}\}} = w\delta_{AH} + (1 - w)\delta_{\{\mathrm{BH^+:A^-}\}} \quad (S85)$$

$$w = \frac{\delta_{\{\mathrm{AH:A^-}\}} - \delta_{\{\mathrm{BH^+:A^-}\}}}{\delta_{AH} - \delta_{\{\mathrm{BH^+:A^-}\}}} \quad (S86)$$

$$\delta^{(A)}_{obs} = \delta_{AH}\left(x_{AH} + 2wx_{\{\mathrm{AH:A^-}\}}\right) + \delta_{\{\mathrm{BH^+:A^-}\}}\left(x_{\{\mathrm{BH^+:A^-}\}} + 2(1 - w)x_{\{\mathrm{AH:A^-}\}}\right) \quad (S87)$$

Titration model 6:

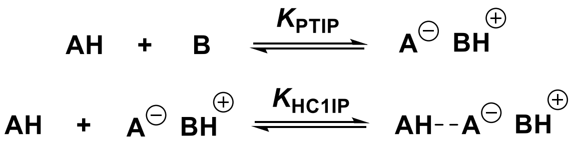

In contrast to titration model 5, the homoconjugate AH:A$^-$ may itself be ion-paired. Such a scenario is adequately modelled by the coupled two equilibria above, including the phenomenological equilibrium constants: (i) K^{C}_{PTIP}, for the formally concerted formation of the primary ion-pair BH$^+$:A$^-$; and (ii) K^{C}_{HC1IP}, for the formally associative first-order homoconjugation of BH$^+$:A$^-$, leading to a doubly hydrogen-bonded complex of A$^-$ in which AH coexists alongside BH$^+$ (AH:A$^-$:BH$^+$). Such a scenario appears plausible for both phenolates (two lone pairs, single site) and ambident azoles (two lone pairs, different sites).

$$K^{C}_{PTIP} = \frac{[\mathrm{BH^+:A^-}]}{[\mathrm{AH}][\mathrm{B}]} \qquad (S88)$$

$$K^{C}_{HC1IP} = \frac{[\mathrm{AH:A^-}:BH^+]}{[\mathrm{AH}][\mathrm{BH^+:A^-}]} \qquad (S89)$$

For a total acid concentration [AH]$_T$ and total base concentration [B]$_T$, the equilibrium concentrations [AH], [BH$^+$:A$^-$], [B], and [AH:A$^-$:BH$^+$] are defined by mass balance considerations:

$$[\mathrm{B}]_\mathrm{T} = [\mathrm{B}] + [\mathrm{BH^+:A^-}] + [\mathrm{AH:A^-:BH^+}] \qquad (S90)$$

$$[\mathrm{AH}]_\mathrm{T} = [\mathrm{AH}] + [\mathrm{BH^+:A^-}] + 2[\mathrm{AH:A^-:BH^+}] \qquad (S91)$$

Substitution affords a pair of simultaneous equations in [AH] and [AH:A$^-$:BH$^+$]:

$$K^{C}_{PTIP} = \frac{[\mathrm{BH^+:A^-}]}{[\mathrm{AH}][\mathrm{B}]} = \frac{[\mathrm{AH}]_\mathrm{T} - [\mathrm{AH}] - 2[\mathrm{AH:A^-}:BH^+]}{([\mathrm{B}]_\mathrm{T} - [\mathrm{AH}]_\mathrm{T} + [\mathrm{AH}] + [\mathrm{AH:A^-}:BH^+]\,)[\mathrm{AH}]} \qquad (S92)$$

$$K^{C}_{HC1IP} = \frac{[\mathrm{AH:A^-}:BH^+]}{[\mathrm{AH}][\mathrm{BH^+:A^-}]} = \frac{[\mathrm{AH:A^-}:BH^+]}{([\mathrm{AH}]_0 - [\mathrm{AH}] - 2[\mathrm{AH:A^-:BH^+}]])[\mathrm{AH}]} \qquad (S93)$$

Rearrangement in turn gives a cubic equation in [AH] ([AH]$_T$ was kept constant throughout each titration, whilst [B]$_T$ increased between each successive titration point):

$$A[\mathrm{AH}]^3 + B[\mathrm{AH}]^2 + C[\mathrm{AH}] + D = 0 \qquad (S94)$$

with the polynomial coefficients A, B, C, and D. The cubic equation can be solved analytically or numerically.

For notational simplicity, $K^{C}_{PTIP} = K_1$ and $K^{C}_{HC1IP} = K_2$ in the expressions below:

$$A = K_1K_2 \qquad \mathrm{B} = K_1(1 - K_2[\mathrm{AH}]_\mathrm{T} + 2K_2[\mathrm{B}]_\mathrm{T}) \tag{S95}$$

$$C = K_1[\mathrm{B}]_\mathrm{T} - K_1[\mathrm{AH}]_\mathrm{T} + 1 \qquad D = -[\mathrm{AH}]_\mathrm{T}$$

The observed chemical shift of the acid under a fast exchange regime, $\delta^{(A)}_{calc}$, will be the mole-fraction weighted average of the intrinsic shifts $\delta_{\{AH\}}$, $\delta_{\{AH:A-\}}$, and $\delta_{\{BH+:A-\}}$, such that:

$$\delta^{(A)}_{calc} = \delta_{AH}x_{AH} + \delta_{\{\mathrm{BH^+:A^-}\}}x_{\{\mathrm{BH^+:A^-}\}} + 2\delta_{\{\mathrm{AH:A^-}:BH^+\}}x_{\{\mathrm{AH:A^-}:BH^+\}}, \quad x_i = \frac{[i]}{[AH]_T} \tag{S96}$$

$$x_{AH} + x_{\{\mathrm{BH^+:A^-}\}} + 2x_{\{\mathrm{AH:A^-}:BH^+\}} = 1 \tag{S97}$$

Assuming the intrinsic shift $\delta_{\{AH:A-:BH+\}}$ to be a weighted average of the other two intrinsic shifts, with a single weighting coefficient *w* (*vide supra*), then:

$$\delta_{\{\mathrm{AH:A^-}:BH^+\}} = w\delta_{AH} + (1 - w)\delta_{\{\mathrm{BH^+:A^-}\}} \tag{S98}$$

$$w = \frac{\delta_{\{\mathrm{AH:A^-}:BH^+\}} - \delta_{\{\mathrm{BH^+:A^-}\}}}{\delta_{AH} - \delta_{\{\mathrm{BH^+:A^-}\}}} \tag{S99}$$

$$\delta^{(A)}_{calc} = \delta_{AH}\left(x_{AH} + 2wx_{\{\mathrm{AH:A^-}:BH^+\}}\right) + \delta_{\{\mathrm{BH^+:A^-}\}}\left(x_{\{\mathrm{BH^+:A^-}\}} + 2(1 - w)x_{\{\mathrm{AH:A^-}:BH^+\}}\right) \tag{S100}$$

S6.4. Fitted isotherms

Titrations with 6_H

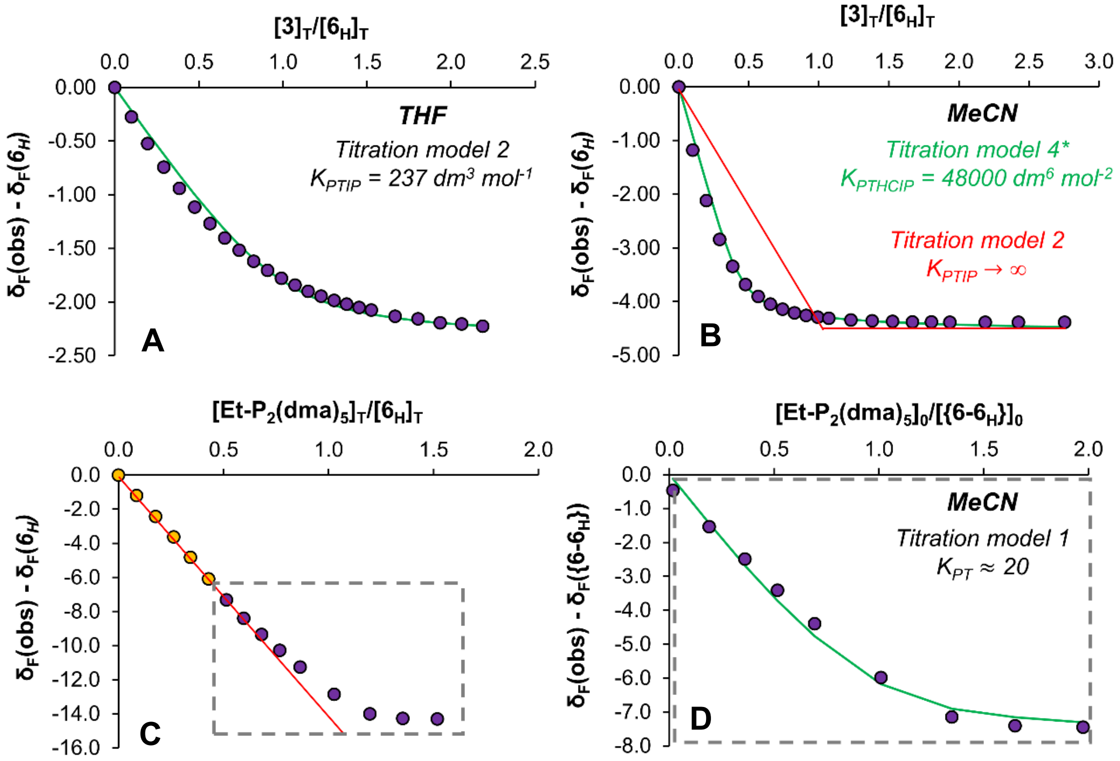

Figure S84: (**A**) Isotherm obtained from the ^{19}F NMR titration (T = 20 °C) of $\mathbf{6_H}$ (0.050 M) with DBU **3** (0.005 – 0.11 M) in THF, with data fit to a simple model for salt formation (titration model 2; K_{PTIP} = 237 M^{-1}, $\delta_F(\mathbf{6_{\{DBUH\}}})$ = -130.14 ppm, $\delta_F(\mathbf{6_H})$ = -127.74 ppm). The chemical shift evolutions in both titrations were nominally attributed to salt formation (i.e., $\mathbf{4a_{\{DBUH\}}}$, $\mathbf{6_{\{DBUH\}}}$), but the formation of stable hydrogen-bonded complexes would be kinetically indistinguishable. (**B**) Isotherm obtained from the ^{19}F NMR titration (T = 20 °C) of of $\mathbf{6_H}$ (0.050 M) with DBU **3** (0.005 – 0.14 M) in MeCN, with data fit to a global model for homoconjugate $\mathbf{\{6\text{-}6_H\}_{\{DBUH\}}}$ formation (titration model 4; K_{PTHCIP} = 48,000 M^{-2}, $\delta_F(\mathbf{\{6\text{-}6_H\}_{\{DBUH\}}})$ = -131.74 ppm, $\delta_F(\mathbf{6_H})$ = -127.07 ppm). Essentially identical fits were obtained with titration model 3 (K_{PTHC} = 745 M^{-1}). This suggests negligible ion-pairing between $\mathbf{3_H}^+$ and the homoconjugate $\mathbf{\{6\text{-}6_H\}}^-$ under the given conditions ($[\mathbf{6}]_T$ = 0.050 M, MeCN, 20°C), but there is an inconsistency with the chemical shifts: with excess **3**, the observed shift of the homoconjugate (δ_F(obs) = -131.7 ppm) was notably downfield of independently synthesised $\mathbf{\{6\text{-}6_H\}}_{\{nBu4N+\}}$ (0.025 M, δ_F(obs) = -135.0 ppm), and the *in situ* measured shift of $\mathbf{6_H}$ (0.050 M) following titration with ca 0.5 equiv. Et-P$_2$(dma)$_5$ (δ_F(obs) = -134.4 ppm). The reason for this inconsistency is not clear. Given the difference in the intrinsic acidities of (the conjugate acid of) **3** (pK_{aH}(MeCN) = 24.3) and $\mathbf{6_H}$ (pK_a(MeCN) = 27.2), it was assumed that the concentration of p-fluorophenoxide salt $\mathbf{6_{\{DBUH\}}}$ remained vanishingly low throughout titrations with **3**. (**C**) Isotherm obtained from the ^{19}F NMR titration (T = 20 °C) of $\mathbf{6_H}$ (0.050 M) with Et-P$_2$(dma)$_5$ **17** (0.005 – 0.075 M) in MeCN. Yellow data points: 0 – 0.5 equivs **17**; purple data points: > 0.5 equivs **17**. (**D**) Reformulation of data in (C) in terms of **17** equivalents relative to the first-order homoconjugate $\mathbf{\{6\text{-}6_H\}}$, with the data fit to a simple model for proton transfer (titration model 1; $K_{PT\approx}$ 20, $\delta_F(\mathbf{6}^-)$ = -141.62 ppm, $\delta_F(\mathbf{6_H})$ = -127.07 ppm). All shifts referenced to the ^{19}F signal of 1-fluoronaphthalene (-125.20 ppm).

Titrations with $4a_H$

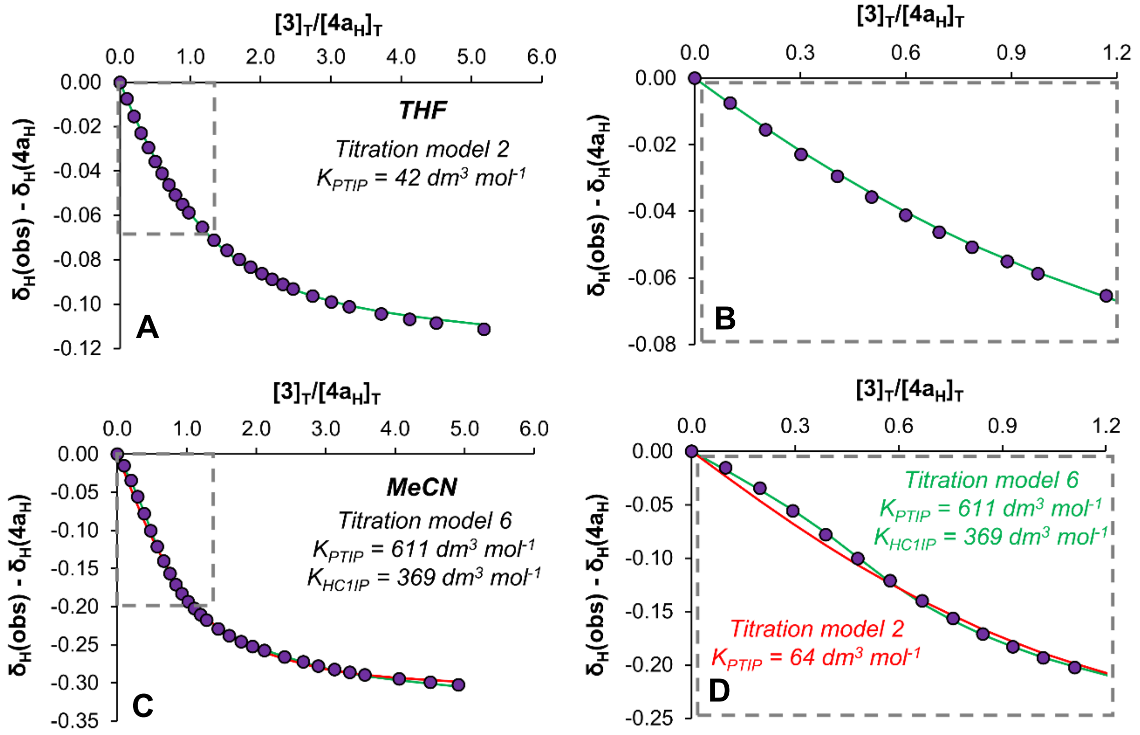

Figure S85: (**A**) Isotherm obtained from the ^{1}H NMR titration of 1,2,4-triazole $\mathbf{4a_H}$ (0.050 M) with DBU **3** (0.005 – 0.25 M) in THF at T = 20°C, and least-squares fit to a simple equilibrium for ion-pair formation (titration model 2; K_{PTIP} = 42 M^{-1}, δ_H($\mathbf{4a_{\{DBUH\}}}$) = 7.956 ppm, δ_H($\mathbf{4a_H}$) = 8.078 ppm). (**B**) Zoomed in portion of isotherm in (A). (**C**) Isotherm obtained from the ^{1}H NMR titration of 1,2,4-triazole $\mathbf{4a_H}$ (0.050 M) with DBU **3** (0.005 – 0.25 M) in MeCN-d_3 at T = 20°C, and least-squares fits to either: (i) ion pair model (titration model 2; K_{PTIP} = 64 M^{-1}, δ_H($\mathbf{4a_{\{DBUH\}}}$) = 7.821 ppm, δ_H($\mathbf{4a_H}$) = 8.145 ppm); or (ii) coupled ion-pair formation and homoconjugation (titration model 6; K_{PTIP} = 611 M^{-1}, K_{HC1IP} = 369 M^{-1}, δ_H($\mathbf{4a_{\{DBUH\}}}$) = 7.821 ppm, δ_H($\mathbf{\{4a\text{-}4a_H\}_{\{DBUH\}}}$) = 8.070 ppm, δ_H($\mathbf{4a_H}$) = 8.145 ppm). (**D**) Zoomed in portion of (C), highlighting the deficiency of titration model 2. All shifts referenced to the residual solvent signals (MeCN-d_3:1.970 ppm; THF-d_8 3.617 ppm).

S7. Computations

S7.1. General considerations

Geometry Optimisations

Optimised geometries and Cartesian force constants for all species were computed with restricted KS-DFT (RKS-DFT) using an ultrafine integration grid and tight optimisation criteria, as implemented in *Gaussian09*.[S14] Unless otherwise stated, the hybrid variant of the Perdew-Burke-Ernzerhof functional [PBE0][S15] was used alongside Grimme's D3 empirical dispersion correction [GD3BJ][S16-S18] (with Becke-Johnson damping) and a moderately augmented, polarised triple-zeta basis set [6-311+G(d,p) (5d,7f)]. Implicit solvation, using the integral-equation formalism of the polarisable continuum model (IEFPCM) and universal force-field (UFF) radii, was implemented during all optimisations and subsequent force constant calculations, using the parameters for MeCN as appropriate. Optimised geometries were routinely characterised as minimum-energy structures or transition states in accordance with a harmonic frequency analysis, with the latter characterised by a Hessian matrix with a single negative eigenvalue (a single imaginary frequency).

Overall, this method corresponds to PBE0+GD3BJ/6-311+G(d,p)/IEFPCM(UFF)/Ultrafine (Method 1-PCM), and potential energies calculated at this level are denoted $E_S^{(\mathbf{1\text{-}PCM})}$. Gas phase potential energies calculated at the same level, but without implicit solvation, are denoted $E_g^{(\mathbf{1})}$; the solvation free energy of any given structure was approximated by $\Delta G_{\text{solv}} \approx E_S^{(\mathbf{1\text{-}PCM})} - E_g^{(\mathbf{1})}$, with both $E_S^{(\mathbf{1\text{-}PCM})}$ and $E_g^{(\mathbf{1})}$ calculated with respect to the same (solution-phase) geometry in each case.

Thermochemistry

Molecular partition functions and thermochemical data were computed at T = 298.15 K (25.0 °C) and p = 1 atm (0.0409 M) using the quasi rigid-rotor harmonic-oscillator approximation, as implemented in the *Goodvibes* program developed by Paton and co-workers.[S19] Molecular masses and rotational constants calculated in *Gaussian09* were used directly for the calculation of translational and rotational enthalpies and entropies; prior to the calculation of vibrational enthalpies, however, all harmonic frequencies obtained from *Gaussian09* were linearly scaled (f_s = 0.95), whilst small imaginary frequencies that could not be eliminated by repeated re-optimisation, or by the imposition of stricter geometry convergence criteria, were inverted to real, positive values. Vibrational entropies were calculated from the scaled harmonic frequencies in accordance with a modification of the harmonic-oscillator approximation outlined by Grimme,[S31] in which contributions to the vibrational entropy from very low harmonic frequencies – those below a certain cutoff (ω_c) – are calculated by interpolation between expressions for the entropy of a harmonic oscillator and a rigid rotor. In this work, all vibrational entropies were calculated using a cutoff frequency of ω_c = 50 cm^{-1}. Free energies calculated in this way, using KS-DFT and solution-phase vibrational frequencies, are denoted [$G_S^{(\mathbf{1\text{-}PCM})}$].

Deficiencies in the KS-DFT surfaces, and accordingly the free energies $G_S^{\mathbf{(1\text{-}PCM)}}$, were corrected by subjecting all structures to supplementary DLPNO-CCSD(T) calculations. Unless otherwise stated, all DLPNO-CCSD(T) calculations were conducted using the minimally augmented but extensively polarised ma-def2-TZVPP basis set (with def2-TZVPP/C), using tight SCF convergence criteria (TightSCF) and the default suite of tight cutoff thresholds (TightPNO) specified in ORCA 4.0.0.2,[S20-S21] without any implicit solvation or RI approximations for either the Coulomb or HF exchange integrals. Potential energies calculated in this way are denoted $E_g^{(\mathrm{DLPNO})}$.Composite free energies, $G_S^{(\mathrm{DLPNO})}$, incorporating DLPNO-CCSD(T) corrections to the potential energy surface, and appropriate standard state corrections (*vide infra*), were then calculated in accordance with:

$$G_S^{(DLPNO)} = E_g^{(DLPNO)} + \left(E_S^{(1-PCM)} - E_g^{(1)}\right) + \left(G_S^{(1-PCM)} - E_S^{(1-PCM)}\right) + RT\ln\left(\frac{c^\circ}{c_{1atm}}\right) \qquad (S101)$$

which includes: (i) the accurate gas-phase potential energy, $E_g^{(\mathrm{DLPNO})}$; (ii) the solvation free energy, approximated by $E_S^{\mathbf{(1\text{-}PCM)}}$ - $E_g^{\mathbf{(1)}}$; (iii) the thermal free energy correction, given by $G_S^{\mathbf{(1\text{-}PCM)}}$ – $E_S^{\mathbf{(1\text{-}PCM)}}$; and (iv) a standard state correction to the translational free energy, $RT\ \ln(c°/c_{1\mathrm{atm}})$, where $c°$ corresponds to the appropriate standard state concentration ($c°$ = 1.00 M for each solute molecule; $c°$ = 19.2 M for MeCN, when modelled explicitly) and $c_{1\mathrm{atm}}$ = 0.0409 M is the concentration of an ideal gas at a pressure of 1 atm (*Gaussian09* outputs free energies at $c_{1\mathrm{atm}}$ by default). At T = 298.15 K, the standard state correction increases the free energy of each molecule by +7.9 kJ mol^{-1}, and a molecule of (solvent) MeCN by +15.2 kJ mol^{-1}, thereby favouring associative processes relative to the gas-phase standard state of $c_{1\mathrm{atm}}$ (especially solute-solvent associations). Equivalently, as all geometries and harmonic frequencies were calculated with implicit solvation, the composite solution-phase free energies $G_S^{(\mathrm{DLPNO})}$ may be expressed as:

$$G_S^{(DLPNO)} = G_S^{(1-PCM)} + \left(E_g^{(DLPNO)} - E_g^{(1)}\right) + RT\ln\left(\frac{c^\circ}{c_{1atm}}\right) \qquad (S102)$$

S7.2. Heavy atom kinetic isotope effects

Bigeleisen-Mayer equation

Theoretical heavy atom kinetic isotope effects (KIEs; k_L/k_H) for the **4a**$_\mathbf{H}$- and **4b**$_\mathbf{H}$-catalysed aminolysis of *p*-F-PhAc **1** with *p*-F-$BnNH_2$ **2** and DBU **3** were computed using the Bigeleisen-Mayer equation (T = 293.1 K) in conjunction with the one-dimensional Bell correction for quantum tunnelling, as implemented in *PyQuiver*.[S22]

For each KIE calculation, isotope-independent Cartesian force constants for the reactant (A: **1** or **2**) and transition state (‡: TS1a/b – TS10a/b) of interest were supplied by preluding electronic structure calculations (RKS-DFT) in *Gaussian09* (Rev E.01). Isotope-dependent harmonic frequencies for each species (e.g., $\{u^{(A)}_{L,i}\}$ for the light isotopologue of A), including the imaginary frequencies for the isotopologous transition states ($iu^{(‡)}_{L,TS}$; $iu^{(‡)}_{H,TS}$), were in turn calculated from these force constants through the use of *PyQuiver*, using appropriate isotope masses (m_{12C} = 12.0000; m_{13C} = 13.003355;

m_{14N} = 14.003074; m_{15N} = 15.000109) and linear scaling as required (*vide infra*), with the appropriate number of translational and rotational modes removed.

$$\frac{k_L}{k_H} = \frac{\kappa_L(T)}{\kappa_H(T)}\left(\frac{\left[\left(\frac{\sigma_L}{\sigma_H}\right)f\right]_{\ddagger}}{\left[\left(\frac{\sigma_L}{\sigma_H}\right)f\right]_{A}}\right), \qquad \frac{\kappa_L(T)}{\kappa_H(T)} = \frac{v^{\ddagger}_{L,TS}}{v^{\ddagger}_{H,TS}}\frac{\sin\left(\frac{u^{\ddagger}_{H,TS}}{2}\right)}{\sin\left(\frac{u^{\ddagger}_{L,TS}}{2}\right)}, \qquad u_j = \frac{hv_j}{k_BT} \tag{S103}$$

$$\left[\left(\frac{\sigma_L}{\sigma_H}\right)f\right]_{A} = \left[\prod_{j}^{3N^{(A)}-6}\left(\frac{u_{L,j}{}^{(A)}}{u_{H,j}{}^{(A)}}\right)\right]\left[\prod_{j}^{3N^{(A)}-6}\frac{1-e^{-u_{H,j}{}^{(A)}}}{1-e^{-u_{L,j}{}^{(A)}}}\right]\left[\prod_{j}^{3N^{(A)}-6}e^{\left(\frac{u_{H,j}{}^{(A)}-u_{L,j}{}^{(A)}}{2}\right)}\right] \tag{S104}$$

$$\left[\left(\frac{\sigma_L}{\sigma_H}\right)f\right]_{\ddagger} = \left(\frac{v_{L,TS}{}^{(\ddagger)}}{v_{H,TS}{}^{(\ddagger)}}\right)\left[\prod_{j}^{3N^{(\ddagger)}-7}\left(\frac{u_{L,j}{}^{(\ddagger)}}{u_{H,j}{}^{(\ddagger)}}\right)\right]\left[\prod_{j}^{3N^{(\ddagger)}-7}\frac{1-e^{-u_{H,j}{}^{(\ddagger)}}}{1-e^{-u_{L,j}{}^{(\ddagger)}}}\right]\left[\prod_{j}^{3N^{(\ddagger)}-7}e^{\left(\frac{u_{H,j}{}^{(\ddagger)}-u_{L,j}{}^{(\ddagger)}}{2}\right)}\right] \tag{S105}$$

Force constant calculations

Default method

Unless otherwise stated, optimised geometries and Cartesian force constants for reactants (A) and transition states (‡) were calculated at the standard optimisation level of theory (1-PCM), as described above. Small imaginary frequencies unrelated to transition vectors were vigorously eliminated by the use of tight convergence criteria and an ultrafine integration grid; persistent small imaginary frequencies (< 50i cm^{-1}) that could not be eliminated in this way were neglected in KIE calculations (these were present in only a handful of structures). Prior to the calculation of KIEs, all harmonic frequencies were scaled linearly, using a ZPVE scaling factor of f_s = 0.980.[S23]

Conformational searching

Cartesian force constants were calculated using the geometries of the lowest energy conformers located for both substrates and transition states. No attempt was made to calculate Boltzmann-weighted reduced isotopic partition function ratios from multiple conformers.

Appropriate transition state conformers were located on the basis of conformational searching of structurally analogous tetrahedral intermediates; these searches were conducted manually, and conformer stabilities assessed at the optimisation level of theory (i.e., $G^{(1)}_{PCM}$). Typical conformational preferences of these intermediates – including the zwitterionic intermediate **4$_{T-ZW}$**, neutral hemiaminal **4$_{T-HA}$**, and alkoxide intermediate **4$_{T-AL}$** – are summarised in Figure S86. In general, the lowest energy conformers of the various tetrahedral intermediates feature extensive intramolecular hydrogen bonding between the amino fragment, carbonyl oxygen and azole catalyst. The lowest energy conformations of tetrahedral species **4$_{T-HA}$** and **4$_{T-AL}$**, with pyramidal nitrogen centres, were generally characterised by an antiperiplanar alignment of the amino lone pair (n_N) and the antibonding σ^*_{C-N} orbital between the tetrahedral centre and the azole fragment. In all tetrahedral species (**4$_{T-ZW}$**, **4$_{T-HA}$**, **4$_{T-AL}$**), the preferred orientation of the azole fragment is apparently dictated by intramolecular hydrogen bonding between

the polarised C-H[5] bond of the azole and the tetrahedral oxygen. These conformational preferences appear to be common to 1,2,4,-triazole $\mathbf{4a_H}$ and pyrazole $\mathbf{4b_H}$.

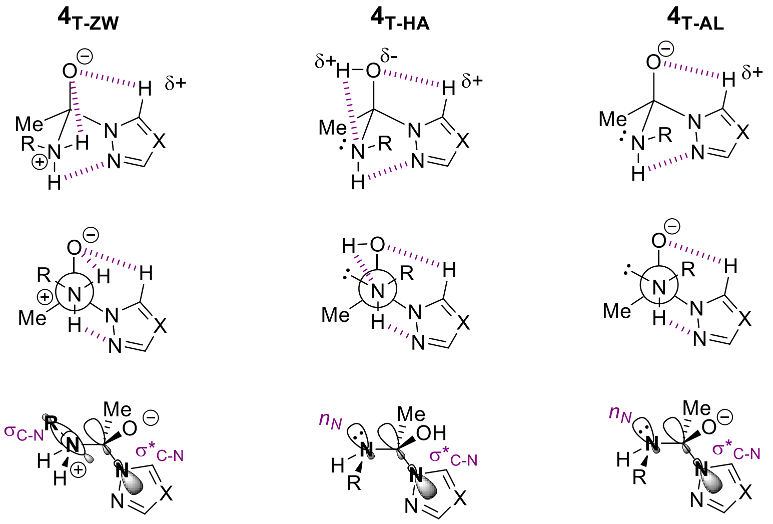

Figure S86: General conformational preferences for key intermediates $\mathbf{4_{T\text{-}ZW}}$, $\mathbf{4T_{\text{-}HA}}$ and $\mathbf{4_{T\text{-}AL}}$ (see main text) involved in the aminolysis of N-acetylated azoles $\mathbf{4_{Ac}}$ with **2**.

The lowest energy conformations of DBU **3** and DBUH$^+$ $\mathbf{3_H}^+$ were determined independently, in the absence of intermolecular complexation with the amine **2** or any tetrahedral intermediate, and these same conformations were used without distortion for all transition states featuring $\mathbf{3/3_H}^+$ explicitly. The conformational spaces of intermolecular complexes were explored by perturbing the orientation and/or binding site of DBU/DBUH$^+$ with respect to the tetrahedral intermediate, but *without* perturbing the (independently refined) internal conformations of either fragment (geometry optimisations of the complexes did not perturb the internal conformations of each fragment). As kinetic isotope effects depend overwhelmingly on local vibrational modes – essentially those in which the labelled atom undergo significant displacement during vibration – it is unlikely that small conformational adjustments to **3** could have any material influence on either the $^{14}N/^{15}N$ amine or carbonyl $^{12}C/^{13}C$ KIEs.

Heavy atom KIEs: $4a_H$-catalysed aminolysis

To provide a reliable benchmark for experiment, the sensitivity of heavy atom KIE calculations to the underlying potential energy surface was explicitly explored for a complement of plausibly rate-determining transition state models (saddle-point structures) involved in the **$4a_H$**-catalysed aminolysis of *p*-F-PhAc **1** with *p*-F-BnNH$_2$ **2** and DBU **3**. For each transition state model, absolute $^{12}C/^{13}C$ KIEs were calculated with respect to the carbonyl (CO; $^{12/13}k_{CO}$) and methyl (CH$_3$; $^{12/13}k_{CH3}$) carbons in *p*-F-PhAc **1**, and $^{14}N/^{15}N$ KIEs with respect to the amino nitrogen (NH$_2$; $^{14/15}k_{NH2}$) in *p*-F-BnNH$_2$ **2**, using the Bigeleisen-Mayer equation (T = 293.1 K) and Cartesian force constants obtained on range of RKS-DFT surfaces. The normalised carbonyl $^{12}C/^{13}C$ KIE ($^{12/13}k_{CO}/^{12/13}k_{CH3}$) was also calculated for each transition state model to provide a direct comparison to the experimental $^{12}C/^{13}C$ KIE, determined by intermolecular competition between [^{13}CO]-**1** and [^{13}CH$_3$]-**1**.

Tables S18 – S26 summarise the average KIEs and standard deviations for each transition state model across a total of eight different surfaces, all of which differ from the default KIE surface (1-PCM) in a single respect. Changes to the exchange-correlation functional (ωB97XD, M06-2X; 1-PCM = PBE0+GD3BJ), solvation model (IEFPCM vs SMD; MeCN), and basis set (6-31+G(d,p), 6-311++G(2d,p), def2-TZVP, cc-pVTZ; 1-PCM = 6-311+G(d,p)) were all explored, with the former having the greatest influence. The re-optimisation of each saddle-point structure with each method began from the corresponding optimised structure on the PBE0/6-311+G(d,p)/IEFPCM(MeCN)/Ultrafine surface.

In general, all $^{12}C/^{13}C$ KIEs were either normal or else inverse but very close to unity; $^{14}N/^{15}N$ KIEs for all transition state models were inverse. Strikingly, calculations on the vast majority of transition state models, even charged transition states or those leading to charge separation (i.e., to the formation of a zwitterionic intermediate), afforded both $^{12}C/^{13}C$ and $^{14}N/^{15}N$ KIEs that were essentially insensitive to the underlying PES, with standard deviations typically less than, or comparable to, experimental uncertainties.

Heavy atom KIEs quoted in the main text for transition states based on **$4a_H$** correspond to average KIEs; those for **$4b_H$**-catalysed aminolysis were calculated only at the standard level of theory, although the uncertainties in these values are likely to be almost identical to those estimated for **$4a_H$**-catalysed aminolysis.

Transition state models: ***TS1-a***

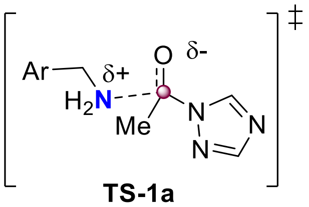

TS-1a

C	2.43964	2.00511	-0.92424
C	2.17902	1.06527	0.23180
H	2.34965	1.51707	-1.89491
H	3.45994	2.38297	-0.81574
H	1.75310	2.84986	-0.86038
O	2.09731	1.47392	1.39748
C	3.77956	-1.91130	-0.77177
C	3.63308	-0.84298	1.02157
N	2.93879	-0.20310	0.06970
N	4.18662	-1.93541	0.52228
N	3.02329	-0.87953	-1.09212
H	4.04507	-2.66952	-1.49501
H	3.70513	-0.47539	2.03294
N	0.53774	0.34302	-0.23536
H	0.56334	-0.07858	-1.16166
C	-0.55251	1.31826	-0.09072
H	0.42679	-0.41141	0.43909
C	-1.90653	0.66745	-0.04104
C	-2.46265	0.28748	1.18032
H	-1.92999	0.49542	2.10331
C	-3.69509	-0.35193	1.23633
H	-4.13900	-0.64837	2.17946
C	-4.35837	-0.60117	0.04915
F	-5.55606	-1.21862	0.09290
C	-3.84333	-0.23653	-1.18147
H	-4.40072	-0.44520	-2.08712
C	-2.60957	0.40072	-1.21604
H	-2.19235	0.69696	-2.17354
H	-0.48942	2.01499	-0.92785
H	-0.34997	1.87021	0.83077

Method change	$^{12/13}k_{CO}$	$^{12/13}k_{CH3}$	$^{14/15}k_{NH2}$	$^{12/13}k_{CO}/^{12/13}k_{CH3}$
None	1.0446	0.9999	0.9918	1.0447
6-31+G(d,p)	1.0442	0.9995	0.9945	1.0447
6-311++G(2d,p)	1.0452	0.9995	0.9921	1.0457
def2-TZVP	1.0427	1.0002	0.9887	1.0425
cc-pVTZ	-	-	-	-
SMD(MeCN)	1.0448	0.9998	0.9937	1.0450
M06-2X	1.0470	0.9993	0.9930	1.0477
wB97XD	1.0448	0.9997	0.9906	1.0451
Average	1.0448	0.9997	**0.9921**	**1.0451**
**Std deviation**	0.0013	0.0003	**0.0020**	**0.0016**

Table S18: $^{12}C/^{13}C$ and $^{14}N/^{15}N$ KIEs computed for TS1-a using the Bigeleisen-Mayer equation (20 °C) and Bell's one-dimensional tunnelling correction. $^{12}C/^{13}C$ KIEs computed relative to *p*-F-PhAc **1** and $^{14}N/^{15}N$ KIEs relative *to* *p*-F-BnNH$_2$ **2**. Default force constants ("none") obtained at the PBE0+GD3BJ/6-311+G(d,p)/IEFPCM(MeCN,UFF) level.

*Transition state models: **4a**T-zw*

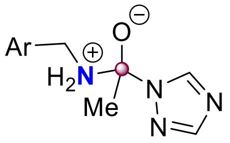

4a$_{T\text{-}zw}$

C	2.37362	1.90600	-1.06109
C	2.07002	1.04851	0.15848
H	2.26174	1.36461	-2.00250
H	3.40860	2.24335	-0.97247
H	1.72623	2.78394	-1.05520
O	2.06047	1.56615	1.30131
C	3.74088	-1.97503	-0.63969
C	3.70864	-0.69603	1.01701
N	2.88285	-0.21855	0.07910
N	4.27747	-1.81549	0.59453
N	2.89213	-1.02887	-0.99631
H	3.97712	-2.80442	-1.29156
H	3.85799	-0.20132	1.96359
N	0.55988	0.34865	-0.16576
H	0.59754	-0.21135	-1.01846
C	-0.54092	1.33536	-0.19281
H	0.42417	-0.29635	0.61237
C	-1.88420	0.67282	-0.09590
C	-2.44948	0.40689	1.15134
H	-1.93074	0.71064	2.05547
C	-3.67489	-0.23876	1.25510
H	-4.12697	-0.44815	2.21740
C	-4.32132	-0.60880	0.08993
F	-5.51184	-1.23288	0.18050
C	-3.79644	-0.35813	-1.16468
H	-4.34109	-0.65939	-2.05172
C	-2.56918	0.28699	-1.24782
H	-2.14377	0.49525	-2.22459
H	-0.45120	1.90492	-1.11785
H	-0.36350	2.00583	0.65045

Method change	$^{12/13}K_{CO}$	$^{12/13}K_{CH3}$	$^{14/15}K_{NH2}$	$^{12/13}K_{CO}/^{12/13}K_{CH3}$
None	1.0280	0.9983	0.9795	1.0298
6-31+G(d,p)	1.0291	0.9990	0.9786	1.0301
6-311++G(2d,p)	1.0304	0.9988	0.9786	1.0316
def2-TZVP	1.0310	0.9989	0.9814	1.0321
cc-pVTZ				
SMD(MeCN)	1.0310	0.9993	0.9762	1.0317
M06-2X	1.0318	0.9985	0.9783	1.0334
wB97XD	1.0308	0.9987	0.9784	1.0321
Average	1.0303	0.9988	**0.9787**	**1.0316**
**Std deviation**	0.0013	0.0003	**0.0016**	**0.0012**

Table S19: $^{12}C/^{13}C$ and $^{14}N/^{15}N$ EIEs computed for **4a**$_{\mathbf{T\text{-}ZW}}$ using the Bigeleisen-Mayer equation (20 °C) and Bell's one-dimensional tunnelling correction. $^{12}C/^{13}C$ KIEs computed relative to *p*-F-PhAc **1** and $^{14}N/^{15}N$ KIEs relative *to* *p*-F-BnNH$_2$ **2**. Default force constants ("none") obtained at the PBE0+GD3BJ/6-311+G(d,p)/IEFPCM(MeCN,UFF) level.

*Transition state models: **TS2-a***

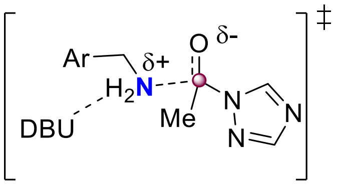

TS2-a

C	-4.20659	2.34427	0.55398
C	-3.71657	1.22013	-0.30526
H	-3.89075	2.24169	1.58956
H	-5.30194	2.33699	0.51750
H	-3.85274	3.28567	0.13511
O	-3.72121	1.23114	-1.52153
C	-3.66176	-1.57854	1.77912
C	-3.83504	-1.27397	-0.28285
N	-3.74441	-0.08234	0.33632
N	-3.79352	-2.24893	0.60216
N	-3.62956	-0.26821	1.66738
H	-3.59137	-2.07017	2.73912
H	-3.93921	-1.36585	-1.35215
N	-1.58406	1.49077	0.24371
H	-1.44289	1.64632	1.23674
C	-0.94468	2.53045	-0.55007
H	-1.18760	0.56526	0.00255
C	0.56181	2.49851	-0.46989
C	1.31511	1.83859	-1.43963
H	0.81478	1.38740	-2.29027
C	2.69761	1.73864	-1.33458
H	3.28898	1.22344	-2.08291
C	3.31377	2.31295	-0.23856
F	4.65703	2.21839	-0.12346
C	2.60712	2.98022	0.74506
H	3.13054	3.42025	1.58608
C	1.22577	3.06857	0.61720
H	0.65675	3.58879	1.38247
H	-1.31570	3.50591	-0.22045
H	-1.26482	2.39553	-1.58835
H	-0.12795	-3.22853	-3.20203
H	1.86818	-3.86201	-1.71176
C	1.47947	-2.84466	-1.83727
C	0.00605	-2.87945	-2.17489
H	2.05126	-2.38066	-2.65228
H	-0.51711	-3.57783	-1.51279
H	3.71672	-2.46446	-0.96484
H	3.08941	-3.38194	0.39419
C	3.07560	-2.38874	-0.07978
N	1.73806	-2.11174	-0.59996
C	-0.55733	-1.48606	-1.96994
H	-1.62676	-1.45483	-2.19647
H	-0.07027	-0.79104	-2.67214
H	4.76222	-1.46175	0.82161
C	0.75899	-1.35372	-0.02968

```
C       3.67405  -1.35215   0.85415
N      -0.35840  -1.04294  -0.60767
H       1.44344  -2.74449   2.15897
H       3.70514  -2.30039   2.79328
C       3.18814  -1.47370   2.29348
C       1.68108  -1.69334   2.36004
H       3.45674  -0.35681   0.45565
C       0.92540  -0.81078   1.37260
H       1.32152  -1.49074   3.37310
H      -0.09343  -0.63835   1.72196
H       3.45413  -0.56105   2.83898
H       1.39423   0.17945   1.31007
```

Method change	$^{12/13}k_{CO}$	$^{12/13}k_{CH3}$	$^{14/15}k_{NH2}$	$^{12/13}k_{CO}/^{12/13}k_{CH3}$
None	1.0310	1.0015	0.9951	1.0295
6-31+G(d,p)	1.0284	1.0014	0.9954	1.0270
6-311++G(2d,p)	1.0348	1.0010	0.9946	1.0338
def2-TZVP	1.0356	1.0009	0.9941	1.0347
cc-pVTZ	1.0353	1.0010	0.9948	1.0343
SMD(MeCN)	1.0351	1.0014	0.9948	1.0337
M06-2X	1.0371	1.0009	0.9978	1.0362
wB97XD	1.0412	1.0003	0.9965	1.0409
Average	1.0348	1.0011	**0.9954**	**1.0337**
**Std deviation**	0.0038	0.0004	**0.0012**	**0.0042**

Table S20: $^{12}C/^{13}C$ and $^{14}N/^{15}N$ KIEs computed for TS2-a using the Bigeleisen-Mayer equation (20 °C) and Bell's one-dimensional tunnelling correction. $^{12}C/^{13}C$ KIEs computed relative to *p*-F-PhAc **1** and $^{14}N/^{15}N$ KIEs relative *to p*-F-BnNH$_2$ **2**. Default force constants ("none") obtained at the PBE0+GD3BJ/6-311+G(d,p)/IEFPCM(MeCN,UFF) level.

*Transition state models: **TS3-a***

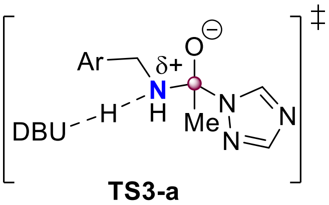

TS3-a

C	3.63766	-2.58804	0.59488
C	2.93650	-1.48147	-0.19832
H	3.36622	-2.58894	1.65411
H	4.71597	-2.43945	0.50675
H	3.38751	-3.55639	0.15584
O	3.21992	-1.38403	-1.44350
C	3.19739	1.32989	2.00406
C	3.79308	0.92626	0.03905
N	3.21824	-0.16332	0.54767
N	3.80648	1.90384	0.93998
N	2.82425	0.07599	1.81109
H	3.02305	1.84013	2.94147
H	4.17325	0.96095	-0.96910
N	1.41123	-1.57139	0.05727
H	1.25055	-1.64714	1.06075
C	0.74558	-2.67877	-0.64634
H	0.93506	-0.49705	-0.31382
C	-0.74572	-2.54596	-0.49903
C	-1.49703	-1.85574	-1.45007
H	-1.01096	-1.46358	-2.33705
C	-2.85955	-1.64780	-1.27505
H	-3.45055	-1.10920	-2.00673
C	-3.45697	-2.14468	-0.13072
F	-4.77761	-1.93929	0.05299
C	-2.75278	-2.84772	0.82913
H	-3.26158	-3.22799	1.70716
C	-1.39069	-3.04464	0.63275
H	-0.82296	-3.59123	1.37989
H	1.08791	-3.63936	-0.25080
H	1.04918	-2.61125	-1.69196
H	1.24752	3.03308	-3.04924
H	-0.46647	4.20070	-1.53641
C	-0.47544	3.13368	-1.78285
C	0.92291	2.64115	-2.08266
H	-1.12541	3.00689	-2.65760
H	1.62450	2.99726	-1.32128
H	-2.76182	3.49667	-1.07442
H	-2.04247	3.92769	0.46820
C	-2.30050	3.07601	-0.17575
N	-1.07579	2.42748	-0.64902
C	0.90248	1.12601	-2.06936
H	1.88907	0.68912	-2.23971
H	0.24013	0.75644	-2.86599
H	-4.29598	2.63033	0.41141
C	-0.51513	1.30283	-0.16266

```
C      -3.30889   2.16945   0.50862
N       0.43702   0.66248  -0.77946
H      -0.95054   2.60234   2.17441
H      -3.30785   2.78676   2.57685
C      -2.99589   1.92306   1.97991
C      -1.51133   1.66110   2.19738
H      -3.36011   1.22364  -0.04231
C      -0.93411   0.70609   1.16138
H      -1.35016   1.23856   3.19305
H      -0.02923   0.24503   1.56028
H      -3.58007   1.06666   2.33457
H      -1.63386  -0.11458   0.96406
```

Method change	$^{12/13}k_{CO}$	$^{12/13}k_{CH3}$	$^{14/15}k_{NH2}$	$^{12/13}k_{CO}/^{12/13}k_{CH3}$
None	1.0283	1.0001	0.9753	1.0282
6-31+G(d,p)	1.0271	1.0000	0.9754	1.0271
6-311++G(2d,p)	1.0283	0.9998	0.9751	1.0285
def2-TZVP	1.0286	1.0000	0.9761	1.0286
cc-pVTZ	1.0292	1.0001	0.9771	1.0291
SMD(MeCN)	1.0298	1.0006	0.9747	1.0292
M06-2X	1.0297	0.9993	0.9748	1.0304
wB97XD	1.0281	0.9997	0.9735	1.0284
Average	1.0286	1.0000	**0.9753**	**1.0287**
**Std deviation**	0.0009	0.0004	**0.0011**	**0.0009**

Table S21: $^{12}C/^{13}C$ and $^{14}N/^{15}N$ KIEs computed for TS3-a using the Bigeleisen-Mayer equation (20 °C) and Bell's one-dimensional tunnelling correction. $^{12}C/^{13}C$ KIEs computed relative to *p*-F-PhAc **1** and $^{14}N/^{15}N$ KIEs relative *to* *p*-F-BnNH$_2$ **2**. Default force constants ("none") obtained at the PBE0+GD3BJ/6-311+G(d,p)/IEFPCM(MeCN,UFF) level.

*Transition state models: **TS4-a***

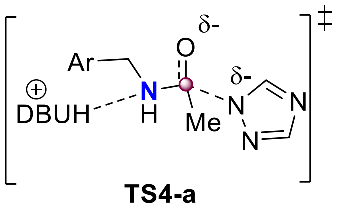

TS4-a

F	-2.76402	3.97372	-0.76001
H	-2.45309	2.01884	-2.42635
C	-1.64808	3.21834	-0.84420
C	-1.60509	2.19723	-1.77516
H	-0.66611	4.30850	0.71600
C	-0.59246	3.49421	0.00451
C	-0.45502	1.42007	-1.84542
H	-0.40260	0.61470	-2.57122
C	0.54768	2.70336	-0.08387
C	0.62990	1.65396	-1.00043
H	1.38634	2.90812	0.57519
C	1.83381	0.75523	-1.03124
H	1.90614	0.24235	-1.99123
H	2.75485	1.33716	-0.89460
N	1.72168	-0.27984	-0.00318
H	1.83397	0.13701	0.91793
H	1.61709	-2.89613	0.91585
C	2.67056	-1.36621	-0.13850
O	2.89552	-1.81007	-1.28574
H	2.51946	-1.80479	1.99406
C	2.54376	-2.32455	1.03344
N	4.21378	-0.45798	0.41820
N	4.23661	0.39193	1.46045
H	3.38638	-3.01656	1.02160
C	5.40895	-0.44731	-0.15711
H	5.64238	-1.04368	-1.02758
C	5.47458	0.87353	1.45596
H	5.81608	1.59659	2.18578
N	6.25481	0.38470	0.46811
H	-3.37502	-3.50074	-0.84155
H	-4.92815	-2.29582	0.54042
H	-4.19426	-2.18256	-1.68517
C	-3.25960	-2.47114	-1.19809
H	-2.18480	-3.13248	-2.92360
H	-5.19417	-1.38462	2.63836
C	-4.36259	-1.37262	0.68759
C	-2.10089	-2.35541	-2.16235
H	-0.70228	-3.50407	-0.98203
C	-4.24361	-1.10383	2.17670
N	-3.09837	-1.59432	-0.03099
H	-3.49378	-1.78271	2.59719
H	-4.92097	-0.57561	0.18227
C	-0.80666	-2.49228	-1.38948
H	-2.12866	-1.38475	-2.66647
C	-1.90770	-1.11425	0.30191

```
H      0.07118  -2.29021  -2.00514
N     -0.81369  -1.52354  -0.31002
C     -3.91498   0.34675   2.50975
H     -4.81751   0.96125   2.42965
H     -3.58162   0.40974   3.55097
H     -1.43687  -0.58866   2.29934
C     -1.69350  -0.07329   1.36632
H      0.08531  -1.03697  -0.10240
C     -2.84100   0.90483   1.58604
H     -3.27235   1.17545   0.61679
H     -0.79520   0.47095   1.06703
H     -2.43174   1.83028   1.99862
```

Method change	$^{12/13}k_{CO}$	$^{12/13}k_{CH3}$	$^{14/15}k_{NH2}$	$^{12/13}k_{CO}/^{12/13}k_{CH3}$
None	1.0458	1.0011	0.9813	1.0447
6-31+G(d,p)	1.0454	1.0010	0.9810	1.0444
6-311++G(2d,p)	1.0463	1.0008	0.9808	1.0455
def2-TZVP	1.0454	1.0007	0.9811	1.0447
cc-pVTZ	1.0448	1.0008	0.9818	1.0440
SMD(MeCN)	1.0477	1.0015	0.9812	1.0461
M06-2X	1.0467	1.0004	0.9810	1.0463
wB97XD	1.0458	1.0008	0.9800	1.0450
Average	1.0460	1.0009	**0.9810**	**1.0451**
**Std deviation**	0.0009	0.0003	**0.0005**	**0.0008**

Table S22: $^{12}C/^{13}C$ and $^{14}N/^{15}N$ KIEs computed for TS4-a using the Bigeleisen-Mayer equation (20 °C) and Bell's one-dimensional tunnelling correction. $^{12}C/^{13}C$ KIEs computed relative to *p*-F-PhAc **1** and $^{14}N/^{15}N$ KIEs relative *to* *p*-F-BnNH$_2$ **2**. Default force constants ("none") obtained at the PBE0+GD3BJ/6-311+G(d,p)/IEFPCM(MeCN,UFF) level.

*Transition state models: **TS5-a***

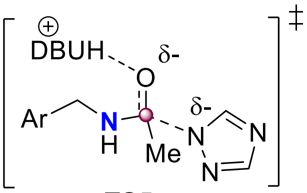

TS5-a

F	-3.51174	3.76048	-1.08823
H	-2.60965	1.92200	-2.67627
C	-2.33660	3.11433	-0.91590
C	-1.96488	2.15058	-1.83539
H	-1.88338	4.18964	0.87849
C	-1.55271	3.43354	0.17557
C	-0.75709	1.49052	-1.64323
H	-0.44849	0.73670	-2.36102
C	-0.35238	2.75317	0.35230
C	0.06300	1.77763	-0.55148
H	0.26008	2.97714	1.21856
C	1.38038	1.05868	-0.40024
H	1.26756	0.02872	-0.74170
H	2.12158	1.52048	-1.06778
N	1.88156	1.02577	0.96012
H	2.45386	1.83358	1.16936
H	2.46802	0.09971	3.49129
C	2.52816	-0.15522	1.37896
O	1.98726	-1.27359	1.12881
H	3.84889	0.92466	2.72657
C	3.22888	0.02587	2.70772
N	4.07114	-0.12526	0.25112
N	4.81501	0.97427	0.03302
H	3.85393	-0.84508	2.90349
C	4.48226	-1.07648	-0.57782
H	4.03118	-2.05830	-0.60649
C	5.65587	0.61071	-0.92931
H	6.39991	1.28610	-1.33266
N	5.49637	-0.66191	-1.35254
H	-2.74198	-3.66735	-1.50018
H	-4.49178	-2.42860	-0.41110
H	-3.20490	-2.17370	-2.32112
C	-2.45204	-2.62438	-1.66981
H	-1.03809	-3.22216	-3.15685
H	-5.20996	-1.81193	1.68505
C	-3.89687	-1.62514	0.02922
C	-1.08392	-2.53866	-2.30752
H	-0.10418	-3.94512	-0.99304
C	-4.15021	-1.59425	1.52569
N	-2.51628	-1.91603	-0.38525
H	-3.60089	-2.41997	1.99083
H	-4.22113	-0.69276	-0.44885
C	-0.03402	-2.88854	-1.27508
H	-0.90754	-1.52716	-2.68266

```
C      -1.41164  -1.61938   0.28874
H       0.97655  -2.70942  -1.64618
N      -0.23158  -2.05291  -0.10751
C      -3.79106  -0.26344   2.17605
H      -4.58697   0.46624   1.99375
H      -3.73051  -0.39882   3.26121
H      -1.42954  -1.46197   2.39605
C      -1.39235  -0.78064   1.53725
H       0.61835  -1.70762   0.40159
C      -2.47395   0.28571   1.64573
H      -2.61808   0.75067   0.66555
H      -0.40254  -0.31855   1.56765
H      -2.10928   1.08208   2.29857
```

Method change	$^{12/13}k_{CO}$	$^{12/13}k_{CH3}$	$^{14/15}k_{NH2}$	$^{12/13}k_{CO}/^{12/13}k_{CH3}$
None	1.0458	0.9998	0.9786	1.0460
6-31+G(d,p)	1.0454	0.9998	0.9784	1.0456
6-311++G(2d,p)	1.0460	0.9996	0.9788	1.0464
def2-TZVP	1.0461	0.9995	0.9784	1.0466
cc-pVTZ	1.0444	0.9996	0.9795	1.0448
SMD(MeCN)	1.0479	1.0001	0.9786	1.0478
M06-2X	1.0457	0.9994	0.9791	1.0463
wB97XD	1.0460	0.9997	0.9786	1.0463
Average	1.0459	0.9997	**0.9788**	**1.0462**
**Std deviation**	0.0010	0.0002	**0.0004**	**0.0009**

Table S23: $^{12}C/^{13}C$ and $^{14}N/^{15}N$ KIEs computed for TS5-a using the Bigeleisen-Mayer equation (20 °C) and Bell's one-dimensional tunnelling correction. $^{12}C/^{13}C$ KIEs computed relative to *p*-F-PhAc **1** and $^{14}N/^{15}N$ KIEs relative *to* *p*-F-BnNH$_2$ **2**. Default force constants ("none") obtained at the PBE0+GD3BJ/6-311+G(d,p)/IEFPCM(MeCN,UFF) level.

*Transition state models: **TS6-a***

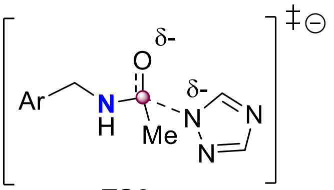

TS6-a

F	5.55920	-0.55806	0.42111
H	4.48189	1.78311	0.19021
C	4.25402	-0.34694	0.13601
C	3.79685	0.95632	0.04022
H	3.82295	-2.44157	0.03832
C	3.42675	-1.43629	-0.04818
C	2.45585	1.16489	-0.25256
H	2.07748	2.17917	-0.32868
C	2.08495	-1.20243	-0.33987
C	1.58231	0.09226	-0.44970
H	1.41862	-2.04753	-0.48421
C	0.13333	0.33437	-0.76757
H	0.03885	0.96194	-1.65762
H	-0.35312	-0.62562	-1.00098
N	-0.53295	1.03184	0.31823
H	-0.48909	0.47569	1.16635
H	-2.06680	3.02282	1.42431
C	-1.90090	1.39615	0.04550
O	-2.15210	1.91261	-1.08696
H	-2.35347	1.44081	2.18798
C	-2.51801	2.03598	1.28555
N	-2.70986	-0.17846	0.07804
N	-2.51110	-1.11342	1.02236
H	-3.59166	2.15605	1.13058
C	-3.54160	-0.66377	-0.83525
H	-3.84502	-0.09581	-1.70195
C	-3.25645	-2.13416	0.61754
H	-3.31462	-3.06142	1.17319
N	-3.92389	-1.91200	-0.53604

Method change	**$^{12/13}k_{CO}$**	**$^{12/13}k_{CH3}$**	**$^{14/15}k_{NH2}$**	**$^{12/13}k_{CO}/^{12/13}k_{CH3}$**
None	1.0492	1.0015	0.9822	1.0476
6-31+G(d,p)	1.0492	1.0013	0.9819	1.0478
6-311++G(2d,p)	1.0501	1.0011	0.9820	1.0489
def2-TZVP	1.0488	1.0012	0.9822	1.0475
cc-pVTZ	1.0475	1.0016	0.9825	1.0458
SMD(MeCN)	-	-	-	-
M06-2X	1.0521	1.0006	0.9825	1.0515
wB97XD	1.0501	1.0012	0.9819	1.0488
Average	1.0496	1.0012	**0.9822**	**1.0483**
**Std deviation**	0.0014	0.0003	**0.0003**	**0.0017**

Table S24: $^{12}C/^{13}C$ and $^{14}N/^{15}N$ KIEs computed for TS6-a using the Bigeleisen-Mayer equation (20 °C) and Bell's one-dimensional tunnelling correction. $^{12}C/^{13}C$ KIEs computed relative to *p*-F-PhAc **1** and $^{14}N/^{15}N$ KIEs relative *to p*-F-BnNH$_2$ **2**. Default force constants ("none") obtained at the PBE0+GD3BJ/6-311+G(d,p)/IEFPCM(MeCN,UFF) level.

Transition state models: ***TS7-a***

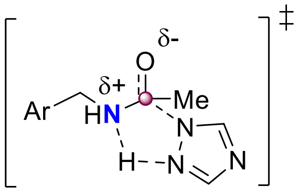

TS7-a

C	2.43961	-1.74783	1.31906
C	1.96993	-1.53324	-0.08803
H	2.22568	-0.89823	1.96718
H	3.51401	-1.92455	1.29446
H	1.95214	-2.64429	1.71655
O	2.24623	-2.23525	-1.03099
C	2.84438	2.45426	0.06038
C	4.04909	0.87556	-0.58664
N	2.86224	0.35772	-0.29578
N	4.09075	2.20213	-0.38136
N	2.07931	1.37061	0.12866
H	2.48663	3.43759	0.33447
H	4.87768	0.28230	-0.94817
N	0.62142	-0.80673	-0.17002
H	0.78642	0.19837	0.10554
C	-0.48506	-1.42919	0.61743
H	0.38054	-0.80525	-1.16249
C	-1.77692	-0.71419	0.35641
C	-2.63496	-1.15235	-0.65180
H	-2.38343	-2.03433	-1.23254
C	-3.81785	-0.47620	-0.92090
H	-4.49666	-0.80708	-1.69790
C	-4.12095	0.64089	-0.16437
F	-5.26614	1.30150	-0.41717
C	-3.29734	1.10305	0.84634
H	-3.57862	1.97870	1.41917
C	-2.11979	0.41387	1.10217
H	-1.46483	0.75845	1.89628
H	-0.20739	-1.36729	1.66927
H	-0.54371	-2.47978	0.32977

Method change	$^{12/13}k_{CO}$	$^{12/13}k_{CH3}$	$^{14/15}k_{NH2}$	$^{12/13}k_{CO}/^{12/13}k_{CH3}$
None	1.0361	1.0003	0.9718	1.0358
6-31+G(d,p)	1.0364	1.0002	0.9717	1.0362
6-311++G(2d,p)	1.0369	1.0001	0.9711	1.0368
def2-TZVP	1.0370	1.0001	0.9720	1.0369
cc-pVTZ	1.0373	1.0001	0.9728	1.0372
SMD(MeCN)	1.0372	1.0008	0.9705	1.0364
M06-2X	1.0376	1.0002	0.9711	1.0374
wB97XD	1.0414	1.0001	0.9703	1.0413
Average	1.0375	1.0002	**0.9714**	**1.0372**
**Std deviation**	0.0017	0.0002	**0.0008**	**0.0017**

Table S25: $^{12}C/^{13}C$ and $^{14}N/^{15}N$ KIEs computed for TS7-a using the Bigeleisen-Mayer equation (20 °C) and Bell's one-dimensional tunnelling correction. $^{12}C/^{13}C$ KIEs computed relative to *p*-F-PhAc **1** and $^{14}N/^{15}N$ KIEs relative *to* *p*-F-BnNH$_2$ **2**. Default force constants ("none") obtained at the PBE0+GD3BJ/6-311+G(d,p)/IEFPCM(MeCN,UFF) level.

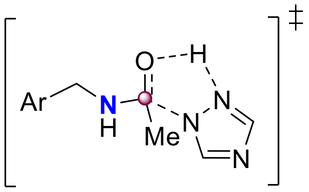

TS8-a

H	4.09595	1.99064	-0.55262
H	1.64499	1.59985	-0.36962
C	3.70153	1.00467	-0.33560
C	2.33586	0.77444	-0.23297
F	5.89284	0.15461	-0.24982
H	-0.05972	-0.81029	-0.89045
C	4.56398	-0.06166	-0.15388
C	1.84322	-0.49964	0.04840
C	0.35935	-0.75543	0.11596
H	-5.44961	-0.51250	-1.85769
N	-3.61574	0.11680	-1.00806
N	-0.35894	0.28829	0.82029
C	4.11518	-1.33736	0.12841
H	-0.27070	0.31259	1.82510
C	-4.75894	-0.55821	-1.02683
C	2.74390	-1.54616	0.23125
H	0.16563	-1.71471	0.60442
O	-1.31751	1.16033	-1.02189
C	-1.36113	0.98561	0.29366
N	-3.03842	-0.19479	0.16530
H	4.82427	-2.14431	0.27195
H	2.37513	-2.54122	0.46018
N	-4.95996	-1.29437	0.08218
C	-3.85629	-1.02684	0.79860
H	-1.28257	2.89318	1.19002
C	-1.97863	2.04951	1.14451
H	-2.17150	1.68561	2.15402
H	-3.64043	-1.43646	1.77550
H	-2.91186	2.38736	0.69739
H	-2.25182	1.03104	-1.37180

Method change	$^{12/13}k_{CO}$	$^{12/13}k_{CH3}$	$^{14/15}k_{NH2}$	$^{12/13}k_{CO}/^{12/13}k_{CH3}$
None	1.0294	0.9982	0.9698	1.0313
6-31+G(d,p)	1.0306	0.9983	0.9700	1.0324
6-311++G(2d,p)	1.0315	0.9981	0.9695	1.0335
def2-TZVP	1.0312	0.9980	0.9698	1.0333
cc-pVTZ	1.0311	0.9980	0.9707	1.0332
SMD(MeCN)	1.0329	0.9981	0.9681	1.0349
M06-2X	1.0310	0.9978	0.9693	1.0333
wB97XD	1.0341	0.9981	0.9691	1.0361
Average	1.0315	0.9981	**0.9695**	**1.0335**
**Std deviation**	0.0014	0.0001	**0.0008**	**0.0015**

Table S26: $^{12}C/^{13}C$ and $^{14}N/^{15}N$ KIEs computed for TS8-a using the Bigeleisen-Mayer equation (20 °C) and Bell's one-dimensional tunnelling correction. $^{12}C/^{13}C$ KIEs computed relative to *p*-F-PhAc **1** and $^{14}N/^{15}N$ KIEs relative *to* *p*-F-BnNH$_2$ **2**. Default force constants ("none") obtained at the PBE0+GD3BJ/6-311+G(d,p)/IEFPCM(MeCN,UFF) level.

Summary of Heavy atom KIEs: 4a$_H$ and 4b$_H$-catalysed aminolysis

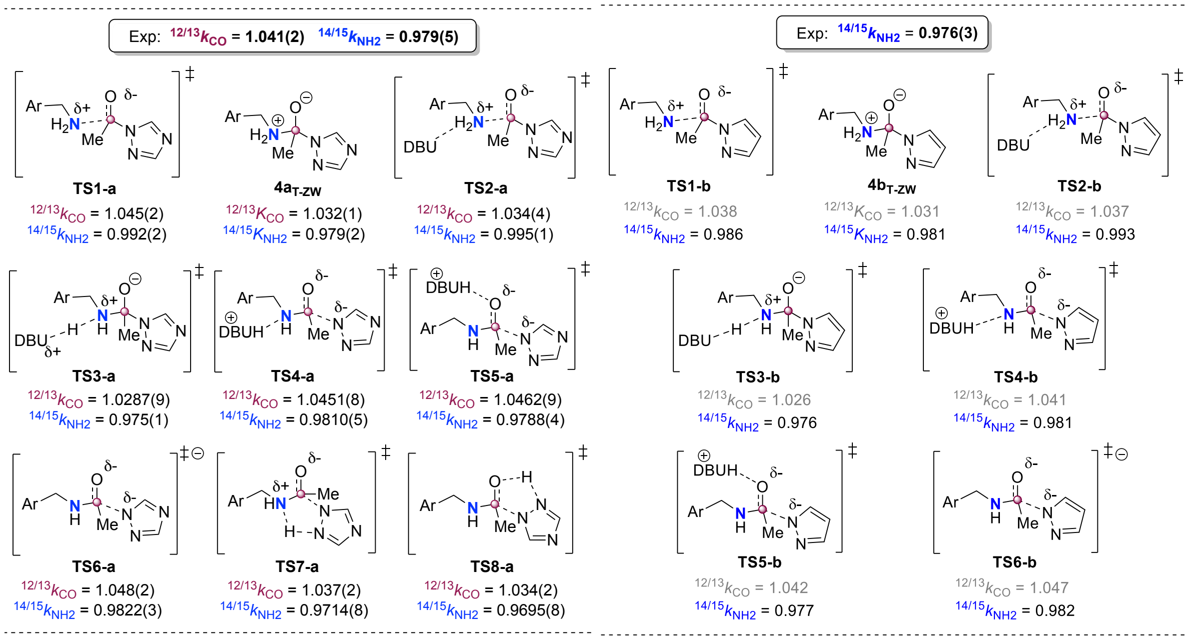

Figure S87: $^{12}C/^{13}C$ and $^{14}N/^{15}N$ KIEs computed for a range of key transition states in the **4aH** and **4b$_H$**-catalysed aminolysis of *p*-F-PhAc **1** with *p*-F-BnNH$_2$ **2** and **3** in MeCN (regimes I and III). $^{12}C/^{13}C$ KIEs computed relative to *p*-F-PhAc **1** and $^{14}N/^{15}N$ KIEs relative *to p*-F-BnNH$_2$ **2**. For **4b$_H$**-catalyssis, the force constants obtained at the PBE0+GD3BJ/6-311+G(d,p)/IEFPCM(MeCN,UFF) level. Saddle-point structures directly analogous to those obtained for **4a$_H$**-catalysed aminolysis,*vide supra*, and uncertainties also expected to be similar.

S7.3. *pKa calculations*

General methods

Logarithmic acid dissociation constants of neutral (pK_a) acids in MeCN, if unavailable in the literature, were computed by the isodesmic method,[S24] using composite DLPNO-CCSD(T) free energy computations, as described above, and experimental pK_a values of analogous acids as anchors. In general, pK_a^{calc} of acids of unknown acidity, A_i-H, were computed according to:

$$pK_a^{\text{calc}}(A_i - H) = pK_a^{\text{exp}}(A_{\text{Ref}} - H) + \frac{\Delta G_{S,PT}}{RT \ln 10} \quad (S106)$$

where $\Delta G_{S,PT}$ corresponds to the solution-phase Gibbs free energy change accompanying the transfer of a proton from an arbitrary acid A_i-H to the reference anion, A_{Ref}^-. For all theoretical pK_a values in this work, $\Delta G_{S,PT}$ was computed *ab initio* using composite solution-phase Gibbs free energies obtained in the standard manner (DLPNO-CCSD(T)/ma-def2-TZVPP/TightPNO//PBE0+GD3BJ/6-311+G(d,p)/MeCN), with geometries optimised using the IEFPCM solvation model (but single-points energies with both IEFPCM and SMD). In each case, the reference acid A_{Ref}-H was chosen so as to resemble the acid of interest A_i-H as closely as possible, both in structure and thermodynamic acidity.

When more extensive experimental data on closely related acids was available in the literature, uncorrected pK_a^{calc} values were refined by calibration. Specifically, calibrated pK_a values for acids of unknown acidity, pK_a^{calb}(A_i-H), were determined according to

$$pK_a^{\text{calb}}(A_i - H) = a\, pK_a^{\text{calc}}(A_i - H) + b \quad (S107)$$

where the calibration parameters (a, b) were determined independently by a linear regression of pK_a^{exp} vs pK_a^{calc} for a series of *structurally analogous* acids of known acidities.

Azoles (4H)

Uncorrected acidities pK_a^{calc}(4H)

The uncorrected, directly computed pK_a values of various neutral azoles in MeCN, pK_a^{calc}(**4H**), were computed relative to imidazole (pK_a^{exp}(**4IH**) = 29.3) according to:

$$pK_a^{\text{calc}}(\text{Az} - H) = pK_a^{\text{exp}}(\text{Im} - H) + \frac{\Delta G_{S,PT}(\text{Az} - H)}{RT \ln 10} \quad (S108)$$

where $\Delta G_{S,PT}$(**4H**) corresponds to the solution-phase Gibbs free energy change accompanying the transfer of a proton from an arbitrary azole **4H** to the imidazolate anion, **4I**$^-$.

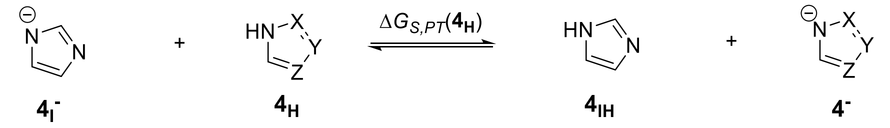

Paramaterisation

Calibrated acidities for all azoles, pK_a^{calb}(**4_H**), were in turn determined using parameters obtained from the linear regression of experimental and *ab initio* pK_a data for a range of electron-deficient indoles (**In^X-H**) in MeCN, including 2-, 3-, 4-, 5- and 7-nitroindole, 3-cyanoindole, and indole itself, spanning ten pK_a units.[S25] Theoretical acidities of the variously substituted indoles, pK_a^{calc}(**In^X-H**), were calculated from $\Delta G_{S,PT}$(**In^X-H**) in the standard manner, using composite DLPNO-CCSD(T) and KS-DFT computations, either the IEFPCM or SMD solvation model with appropriate parameters for MeCN, and imidazole (pK_a^{exp}(**$4I_H$**) = 29.3) as an experimental anchor.

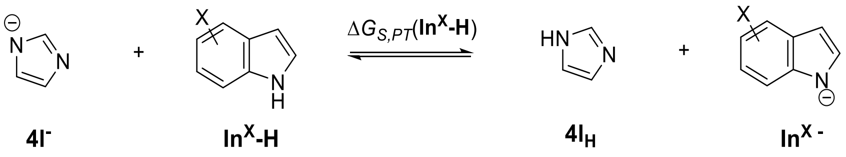

Linear regressions of pK_a^{exp}(**In^X-H**) and pK_a^{calc}(**In^X-H**), using theoretical acidities obtained with both the IEFPCM and SMD solvation models, afforded the results in Table S27 and Figure S88 and the calibration parameters $a_{(PCM)}$, $b_{(PCM)}$, $a_{(SMD)}$ and $b_{(SMD)}$. Directly computed acidities for the various indoles, pK_a^{calc}(**In^X-H**), were remarkably accurate when computed with the IEFPCM model (RMSE = 0.34 pK units); calibration reduced the error further, but only modestly, to 0.31 p*K* units. Directly computed acidities obtained with SMD, in contrast, were considerably poorer (RMSE = 1.11 pK units), but improved more significantly following calibration (RMSE = 0.71 pK units).

**Indole (In^X-H)**	**pK_a^{exp} (MeCN)**	**pK_a^{calc} (PCM)**	**pK_a^{calb} (PCM)**	**pK_a^{calc} (SMD)**	**pK_a^{calb} (SMD)**
3-NO_2-Indole	22.77	22.92	22.97	24.88	23.41
2-NO_2-Indole	23.64	23.36	23.40	24.62	23.12
3-CN-Indole	26.01	25.75	25.71	26.62	25.35
4-NO_2-Indole	27.89	28.06	27.94	29.18	28.20
5-NO_2-Indole	28.18	28.64	28.51	29.74	28.83
7-NO_2-Indole	29.99	30.59	30.39	31.43	30.72
Indole	32.57	32.36	32.11	32.06	31.42
	RMSE	0.34	0.31	1.11	0.71

Table S27: Experimental (pK_a^{exp}),[25] directly computed (pK_a^{calc}) and calibrated (pK_a^{calb}) acidities for seven variously substituted indoles (**In^X-H**) in MeCN. pK_a^{calc} determined using imidazole (pK_a^{exp}(**$4I_H$**) = 29.3) as an anchor, and proton transfer free energies ($\Delta G_{S,PT}$) computed at the DLPNO-CCSD(T)/ma-def2-TZVPP/TightPNO//PBE0+GD3BJ/6-311+G(d,p)/MeCN level, using either the IEFPCM or SMD solvation model and the quasi rigid-rotor harmonic-oscillator approximation of Grimme (ω_c = 50 cm^{-1}). Linear regression parameters: $a_{(PCM)}$ = 0.968, $b_{(PCM)}$ = 0.786. $a_{(SMD)}$ = 1.11; $b_{(SMD)}$ = - 4.32.

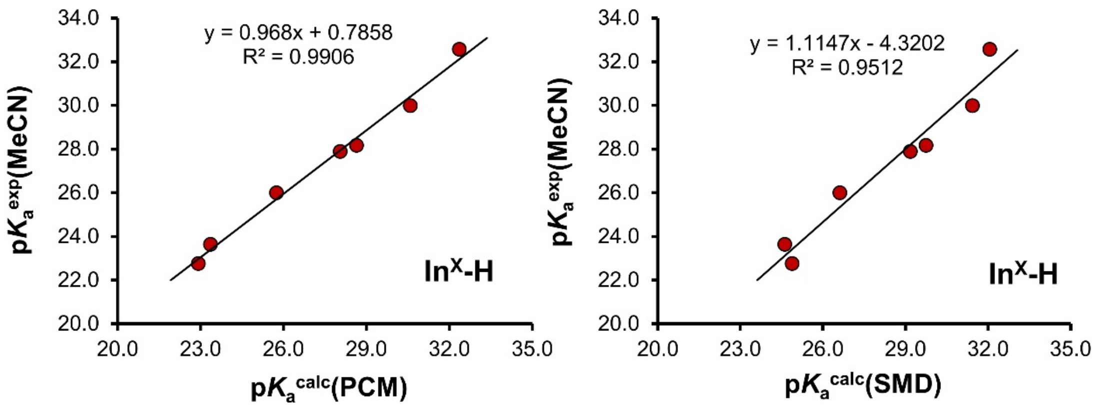

Figure S88: Linear regressions of experimental (pK_a^{exp})[25] and directly computed (pK_a^{calc}) acidities of seven mono-substituted indoles (**In^X-H**) in MeCN. pK_a^{calc} values determined using imidazole (pK_a^{exp}(**4I$_H$**) = 29.3) as an anchor, and proton transfer free energies ($\Delta G_{S,PT}$(**In^X-H**)) computed at the DLPNO-CCSD(T)/ma-def2-TZVPP/TightPNO//PBE0+GD3BJ/6-311+G(d,p)/MeCN level, using either the IEFPCM or SMD solvation model and the quasi rigid-rotor harmonic-oscillator approximation of Grimme (ω_c = 50 cm^{-1}). Linear regression parameters: $a_{(PCM)}$ = 0.968, $b_{(PCM)}$ = 0.786. $a_{(SMD)}$ = 1.11; $b_{(SMD)}$ = - 4.32.

Calibrated acidities pK_a^{calb}(Az-H)

Calibrated pK_a values (Table S28) of all the azoles shown in the main text, pK_a^{calb}(**4$_H$**), were determined by:

$$\mathrm{p}K_a^{\mathrm{calb}}(\mathrm{Az-H}) = a\ \mathrm{p}K_a^{\mathrm{calc}}(\mathrm{Az-H}) + b \qquad (S109)$$

where the directly computed acidities, pK_a^{calc}(**4$_H$**), were determined as described above, and the parameters *a* and *b,* pertaining to either $a_{(PCM)}/b_{(PCM)}$ or $a_{(SMD)}/b_{(SMD)}$ (Figure S88), were determined by the linear regression of experimental and directly computed acidities of various indoles (*vide supra*).

On account of the lower intrinsic RMSE for the indole calibrants, pK_a's determined with the IEFPCM model were used for structure-activity analysis; however, using the SMD acidities had no significant effect on the qualitative nature of the relationships shown in the main text.

Experimental acidities in MeCN (pK_a^{exp}(MeCN)) have seemingly not been reported in the literature for the vast majority of azoles; Bordwell has reported the acidities of several key azoles in DMSO, however, and these are also shown in Table S28 for comparison.[S26]

**Azole (Az-H)**	**pK_a^{exp} (DMSO)**	**pK_a^{calb} (MeCN,PCM)**	**pK_a^{calb} (MeCN,SMD)**
**$4c_H$**	11.9	22.15	20.65
**$4d_H$**	-	22.38	22.38
**$4e_H$**	13.9	23.67	21.98
**$4a_H$**	14.8	24.64	23.60
**$4f_H$**	-	25.90	25.23
**$4g_H$**	-	26.91	26.35
**$4h_H$**	-	27.04	26.77
**$4i_H$**	-	27.68	27.42
**$4j_H$**	-	28.15	27.69
**$4k_H$**	-	28.73	28.11
**$4l_H$**	18.6	29.15	28.34
**$4m_H$**	-	29.13	28.38
**$4n_H$**	-	29.05	28.43
**$4o_H$**	-	29.32	28.65
**$4p_H$**	-	29.51	28.75
**$4q_H$**	-	29.79	28.89
**$4b_H$**	19.8	30.08	29.03
**$4r_H$**	-	31.21	30.20

Table S28: Calibrated acidities (pK_a^{calb}) for various azoles (**4_H**) in MeCN, determined according to pK_a^{calb} = a.pK_a^{calc} + b and pK_a^{calc} = pK_a^{exp}(**$4l_H$**) + $\Delta G_{S,PT}$(**4_H**)/(RT.ln 10), where $a_{(PCM)}$ = 0.968, $b_{(PCM)}$ = 0.786, $a_{(SMD)}$ = 1.11, $b_{(SMD)}$ = -4.32, pK_a^{exp}(**$4l_H$**) = 29.3 is the reference pK_a of imidazole in MeCN, and $\Delta G_{S,PT}$(Az-H) was calculated *ab initio* for each azole using composite DLPNO-CCSD(T) and KS-DFT computations. pK_a^{calc} values for ambident azoles determined with respect to the most stable tautomer of the acid,[S27-S40] as determined by computation; the most favourable tautomers were found to be 2H-1,2,3-triazole (**$4d_H$**), 1H-benzotriazole (**$4c_H$**), and 1H-1,2,4-triazole (**$4a_H$**). 1H- and 2H-1,2,3-triazole are apparently nearly degenerate in MeCN.

S8. NMR spectra

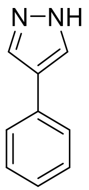

^{1}H NMR (400 MHz, CD_3CN)

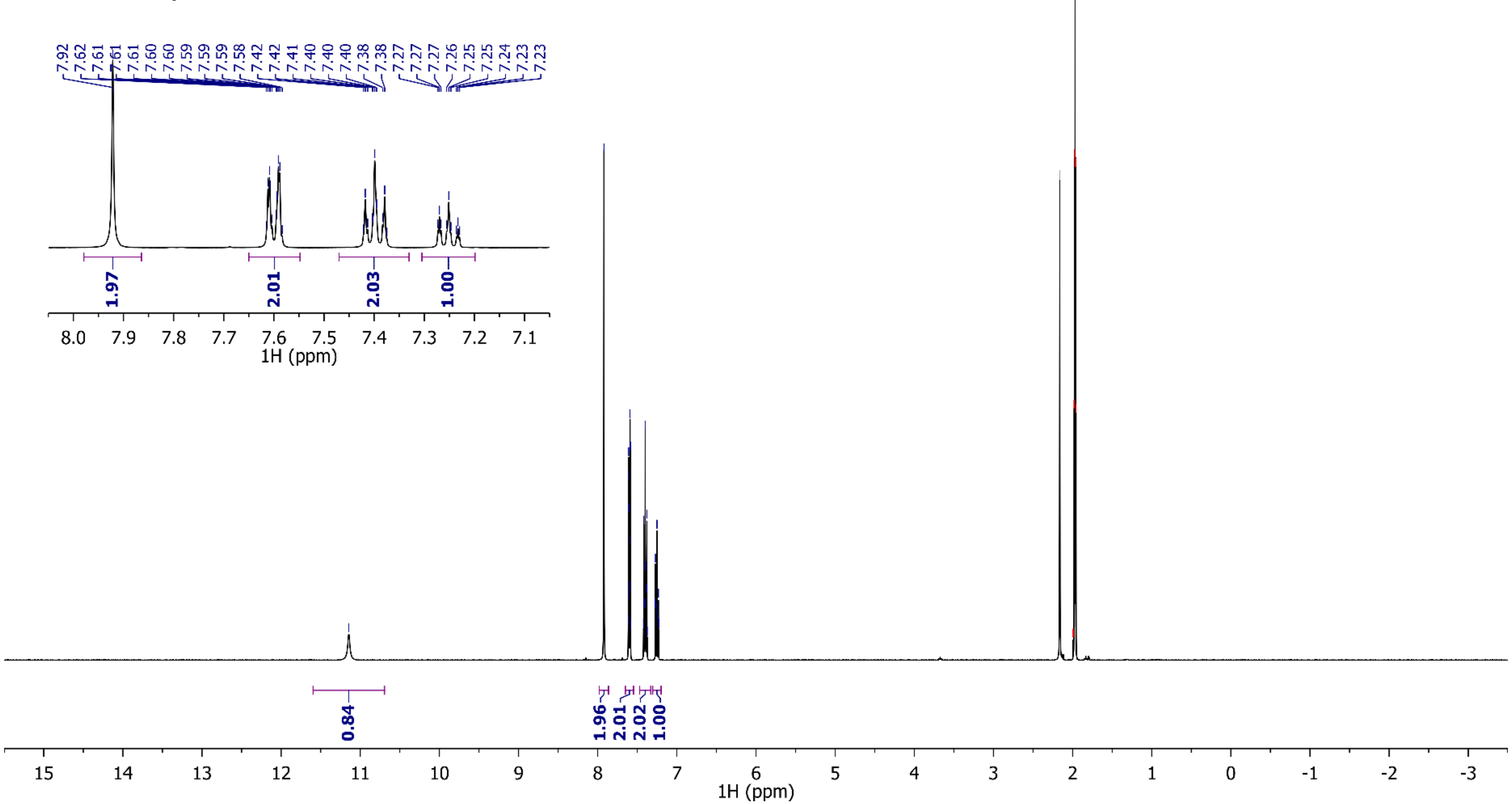

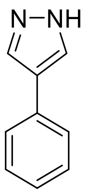

^{13}C NMR (101 MHz, CD_3CN)

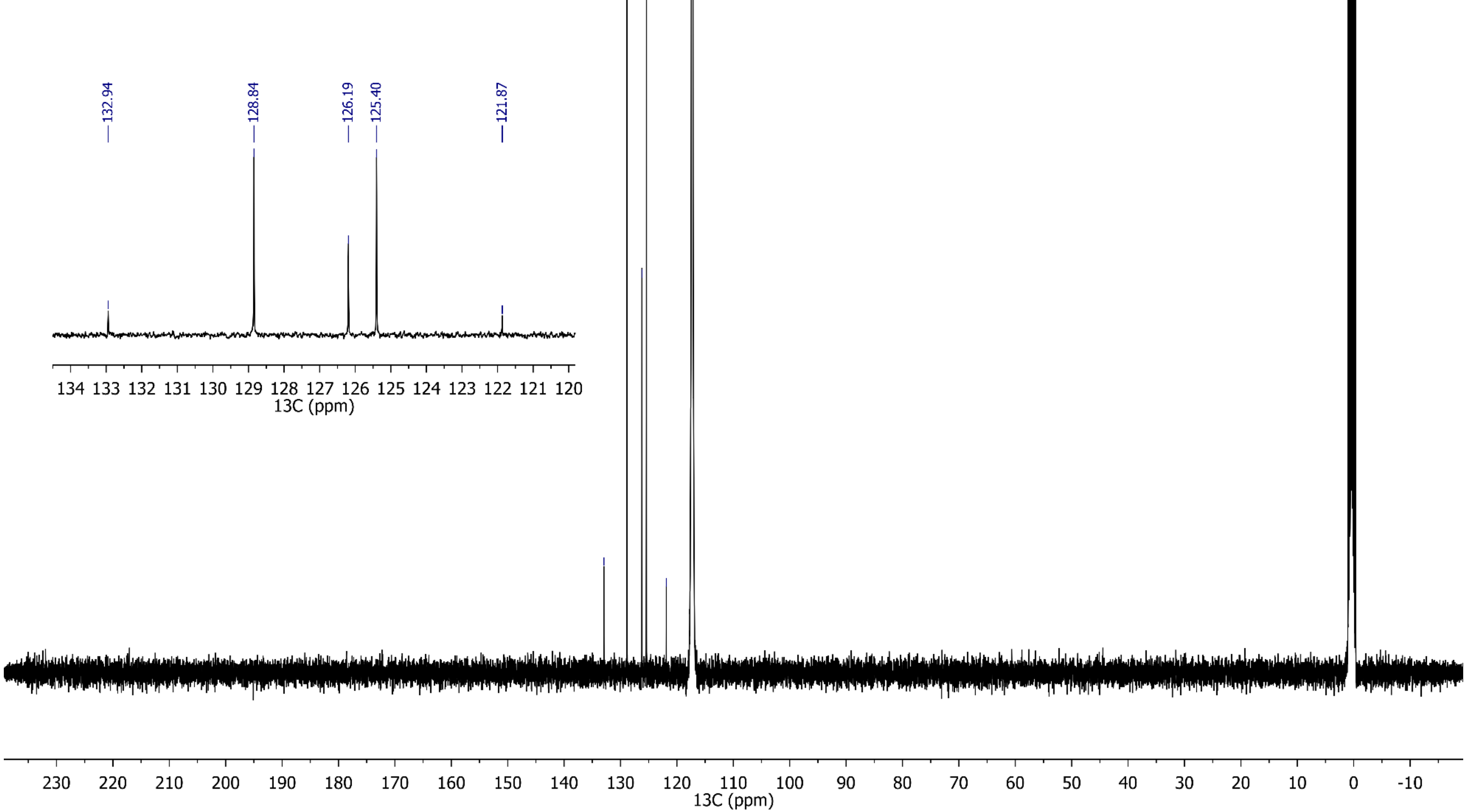

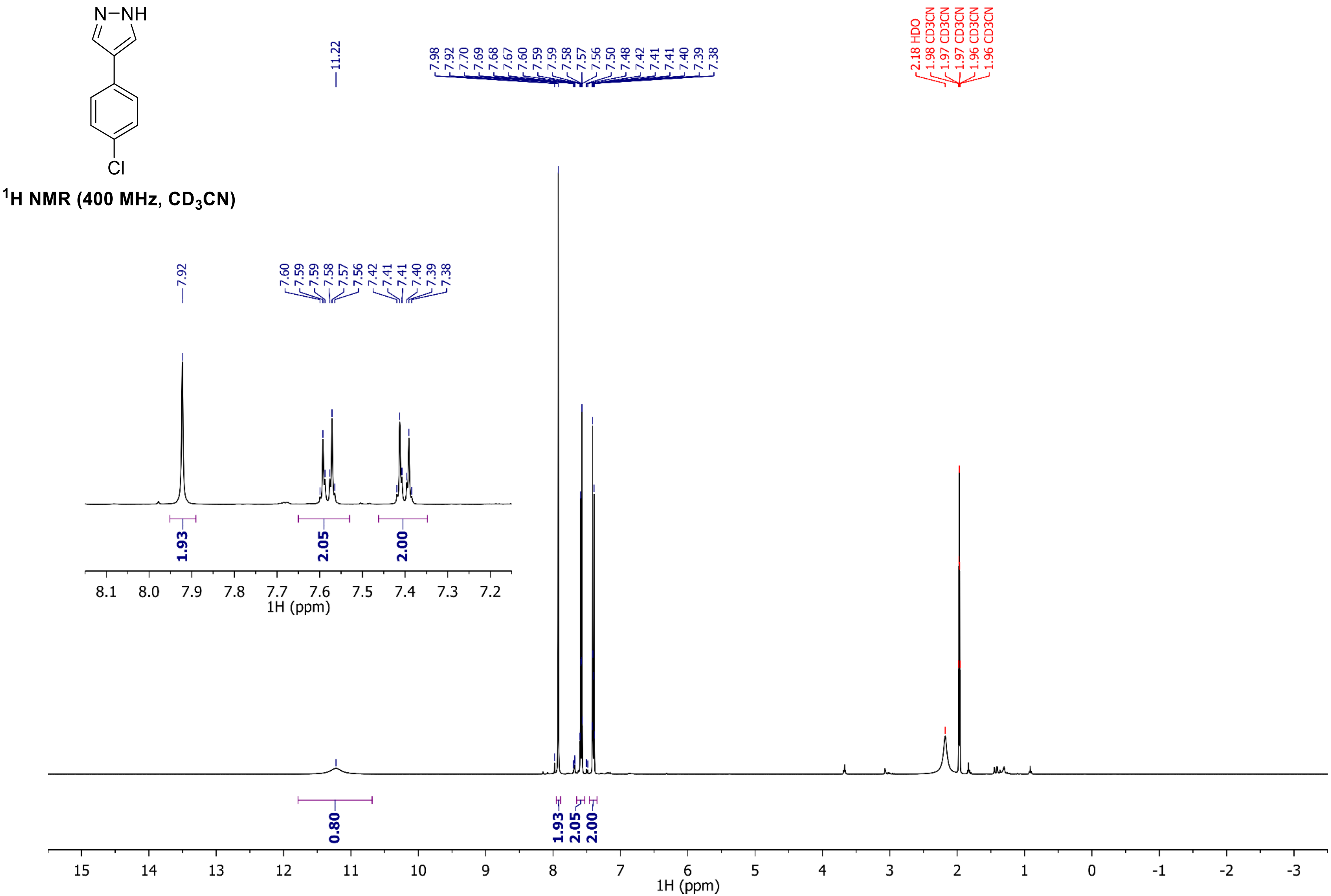

N–NH
Cl
^{1}H NMR (400 MHz, CD_3CN)
11.22
7.98 7.92 7.70 7.69 7.68 7.67 7.60 7.59 7.59 7.58 7.57 7.56 7.50 7.48 7.42 7.41 7.41 7.40 7.39 7.38
2.18 HDO
1.98 CD3CN
1.97 CD3CN
1.97 CD3CN
1.96 CD3CN
1.96 CD3CN
7.92
7.60 7.59 7.59 7.58 7.57 7.56 7.42 7.41 7.41 7.40 7.39 7.38
1.93
2.05
2.00
1H (ppm)
0.80
1.93
2.05
2.00
1H (ppm)

^{13}C NMR (101 MHz, CD_3CN)

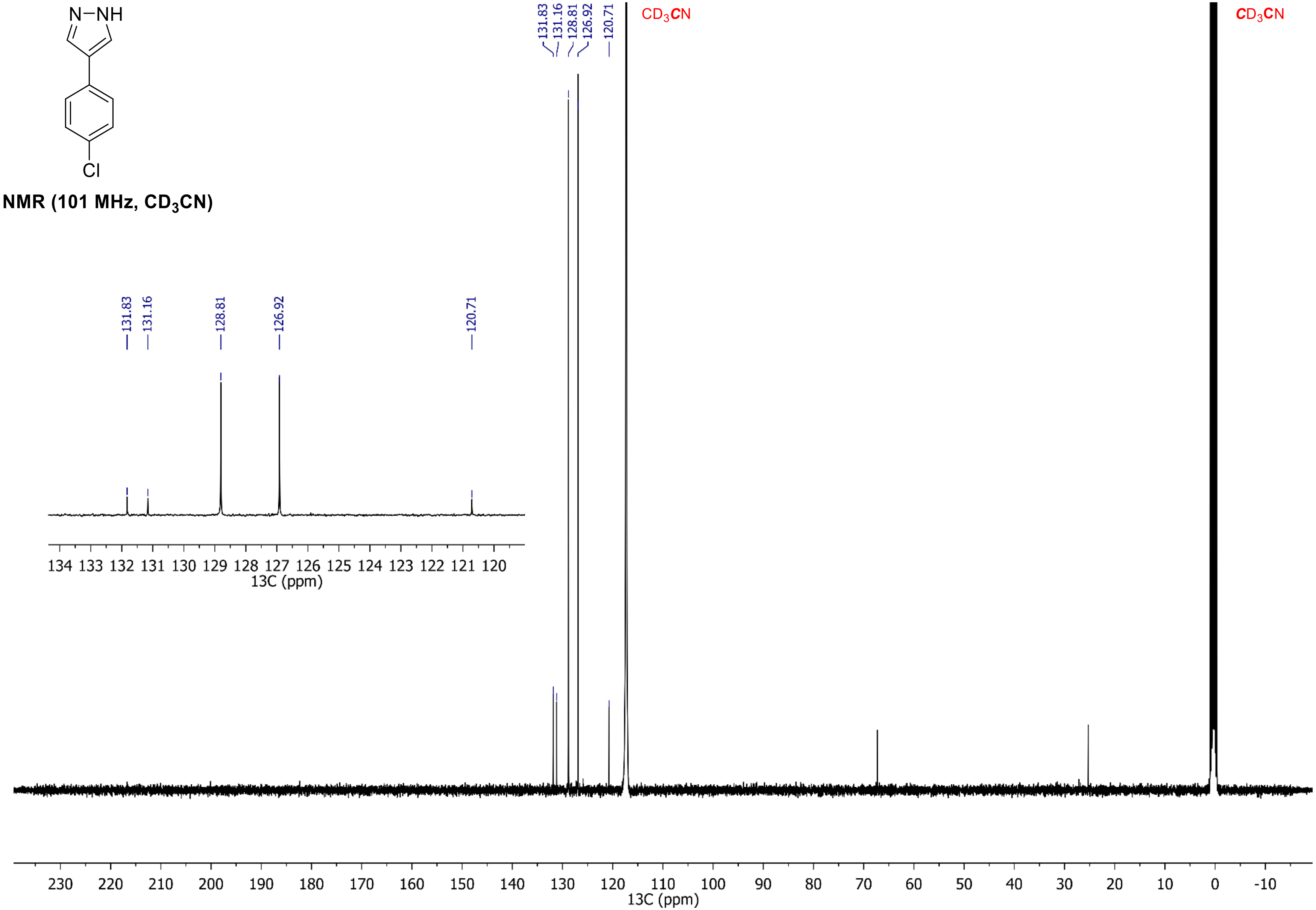

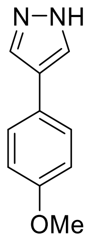

^{1}H NMR (400 MHz, CD_3CN)

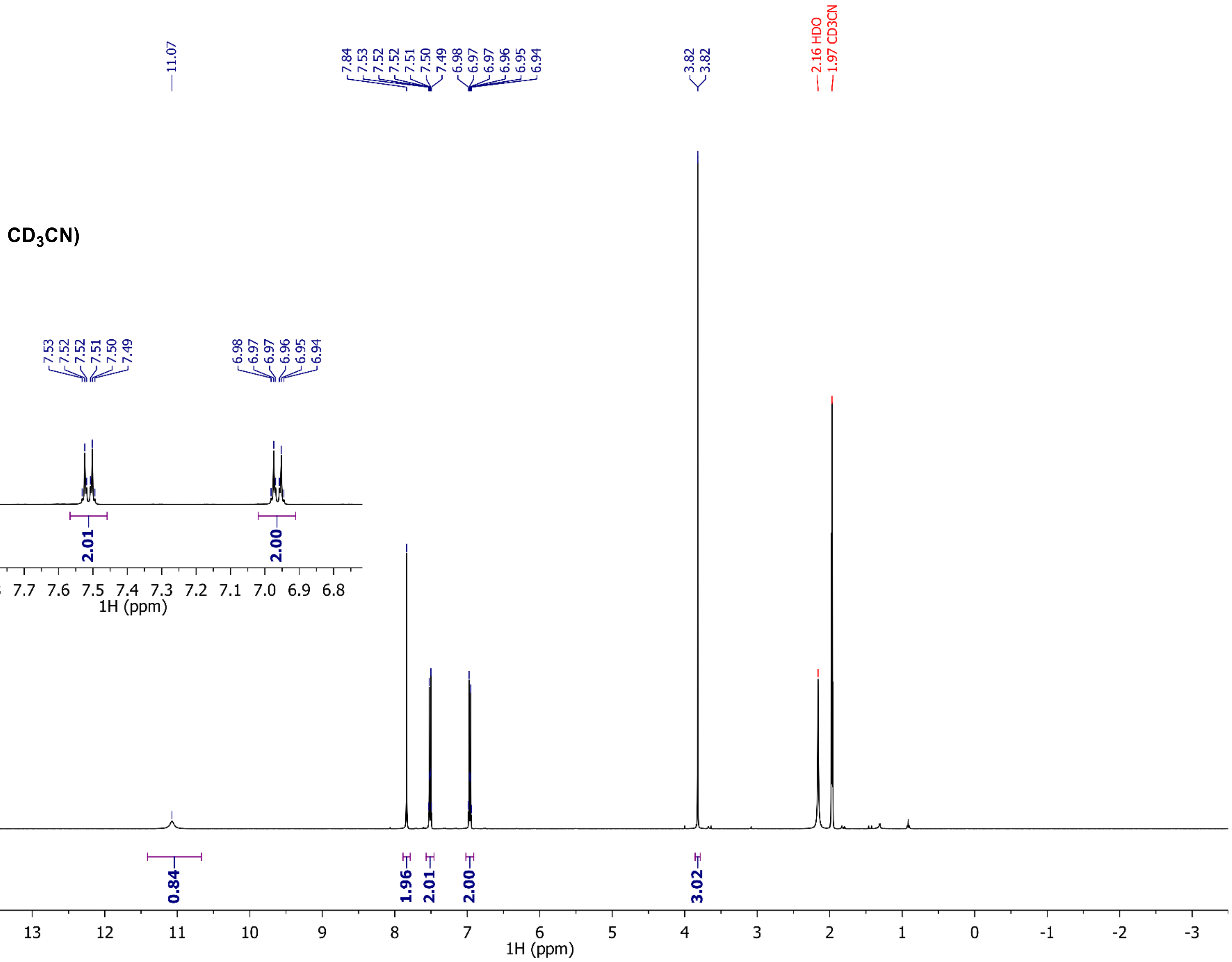

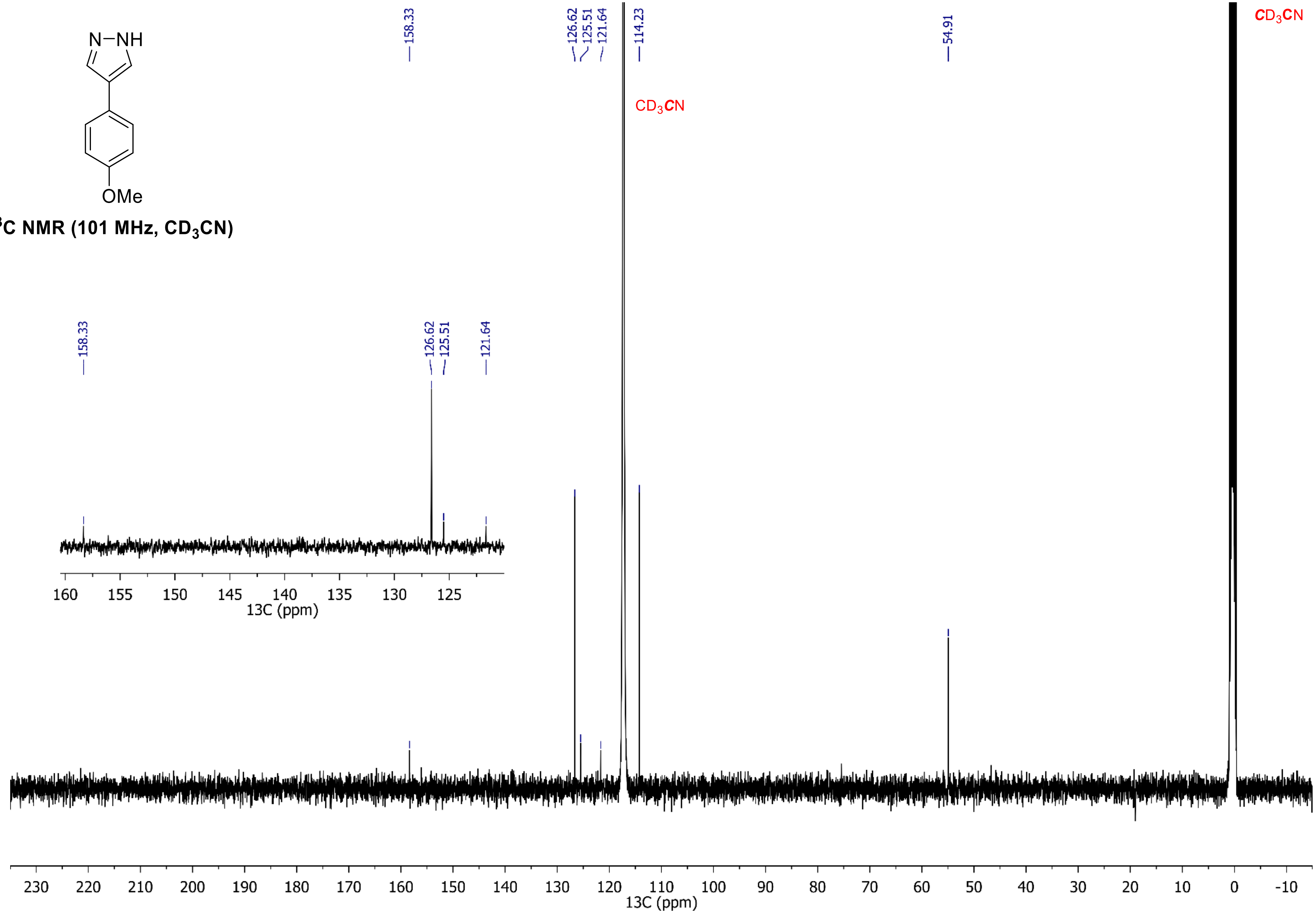

^{13}C NMR (101 MHz, CD_3CN)

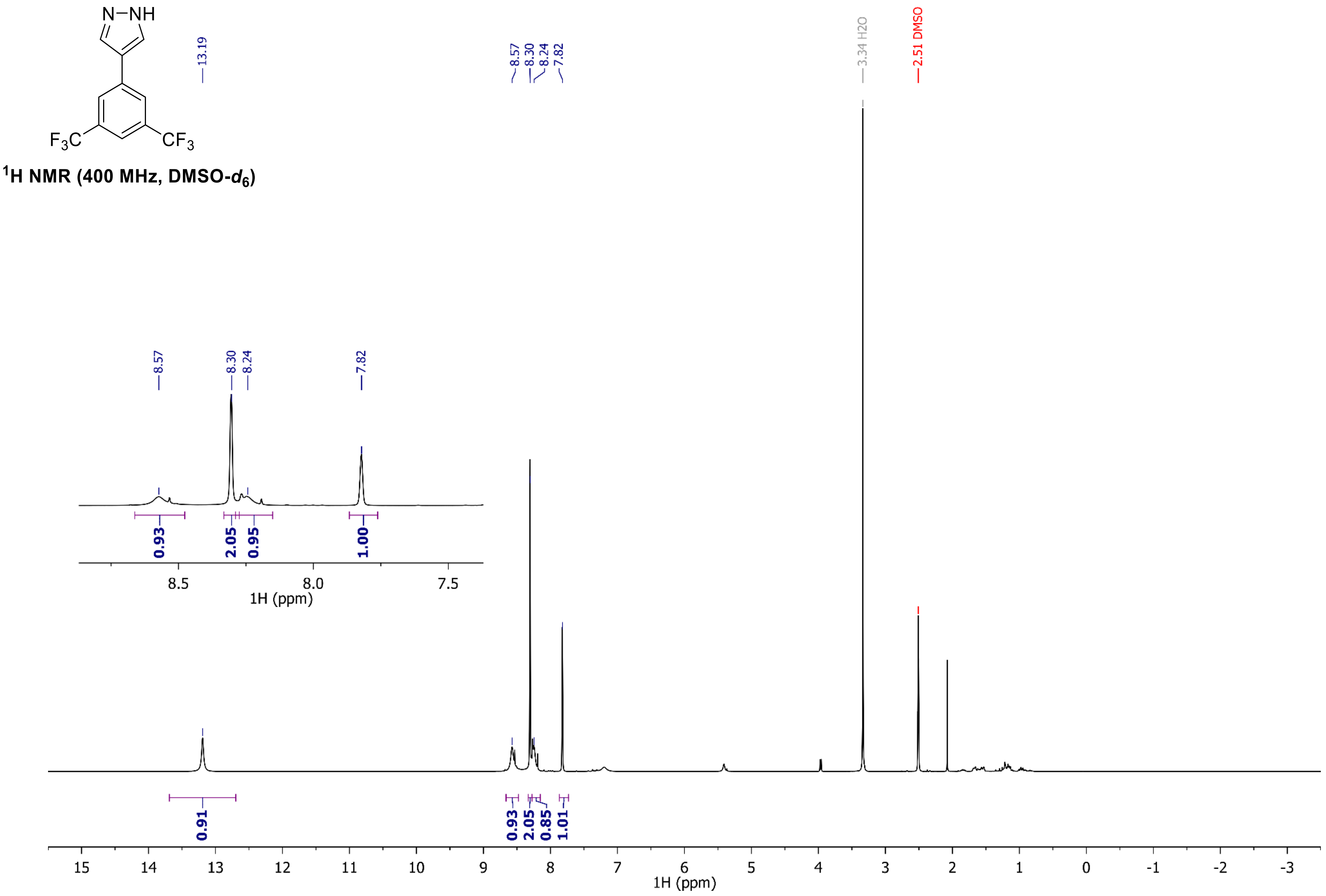
N–NH
F3C
CF3
1H NMR (400 MHz, DMSO-d6)
13.19
8.57
8.30
8.24
7.82
3.34 H2O
2.51 DMSO
0.93
2.05
0.95
1.00
1H (ppm)
0.91
0.85
1.01

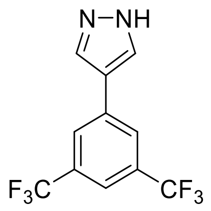

^{13}C NMR (101 MHz, DMSO-d_6)

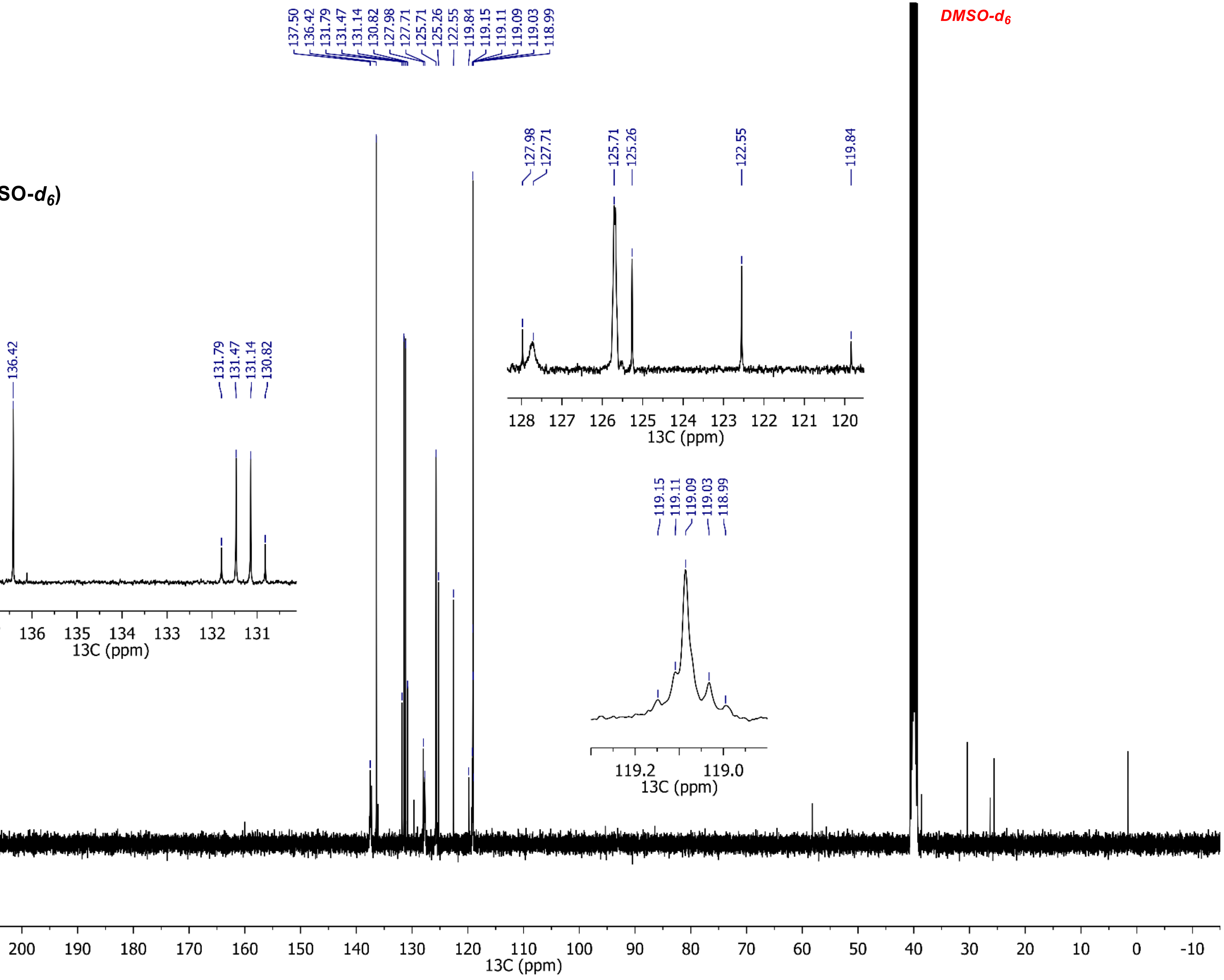

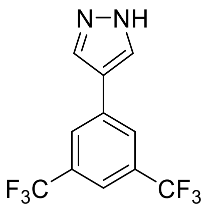

^{19}F NMR (377 MHz, DMSO-d_6)

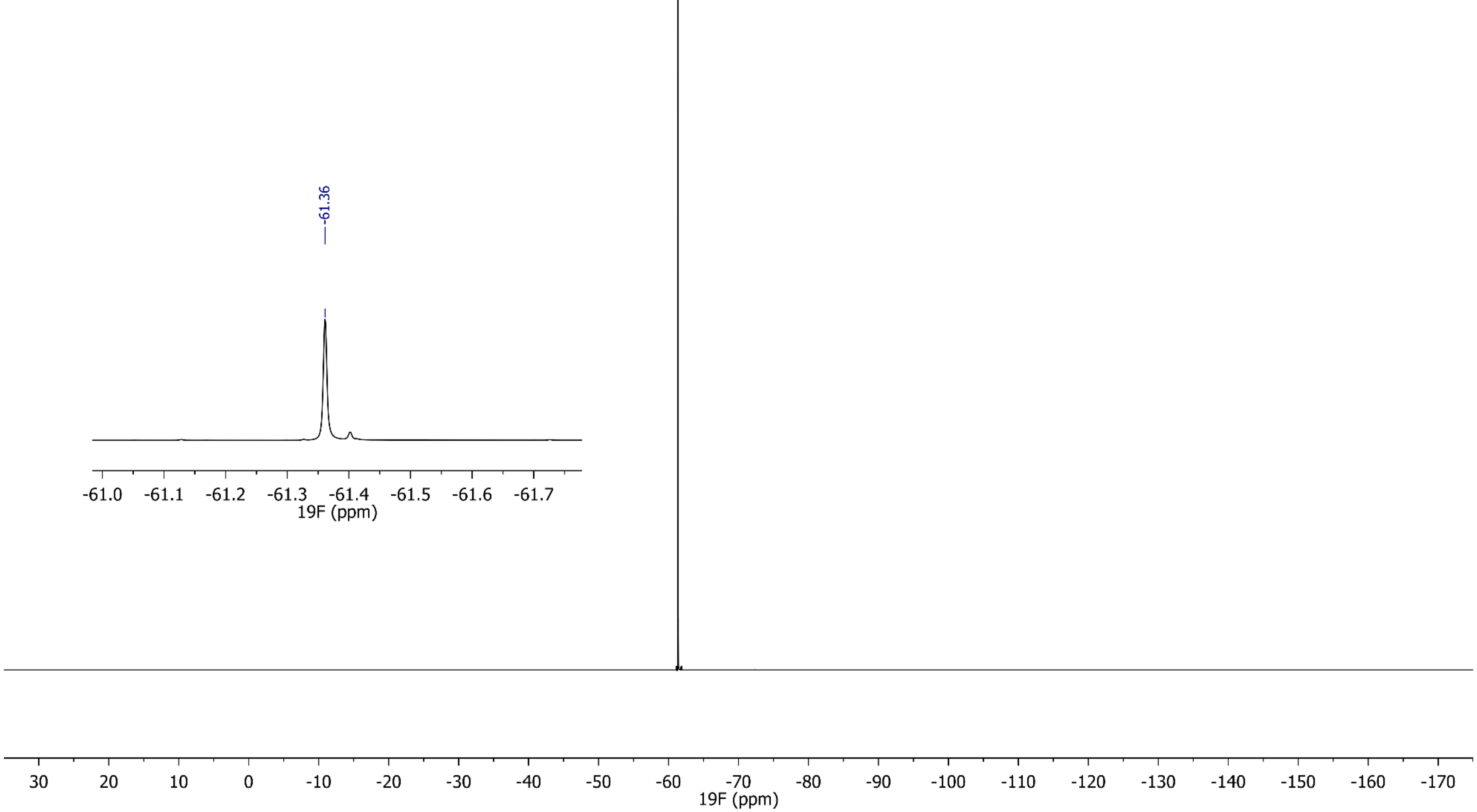

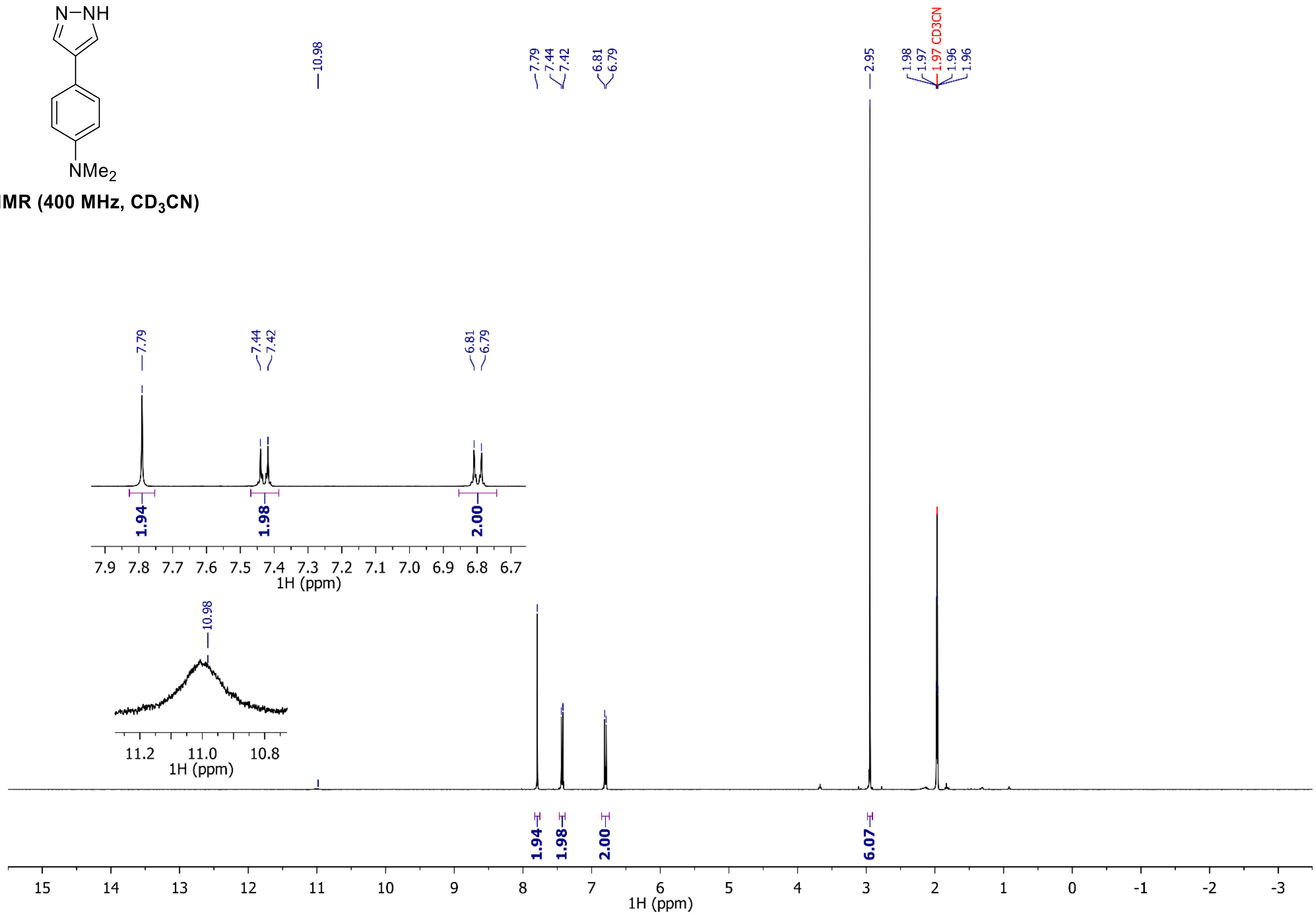

^{1}H NMR (400 MHz, CD_3CN)

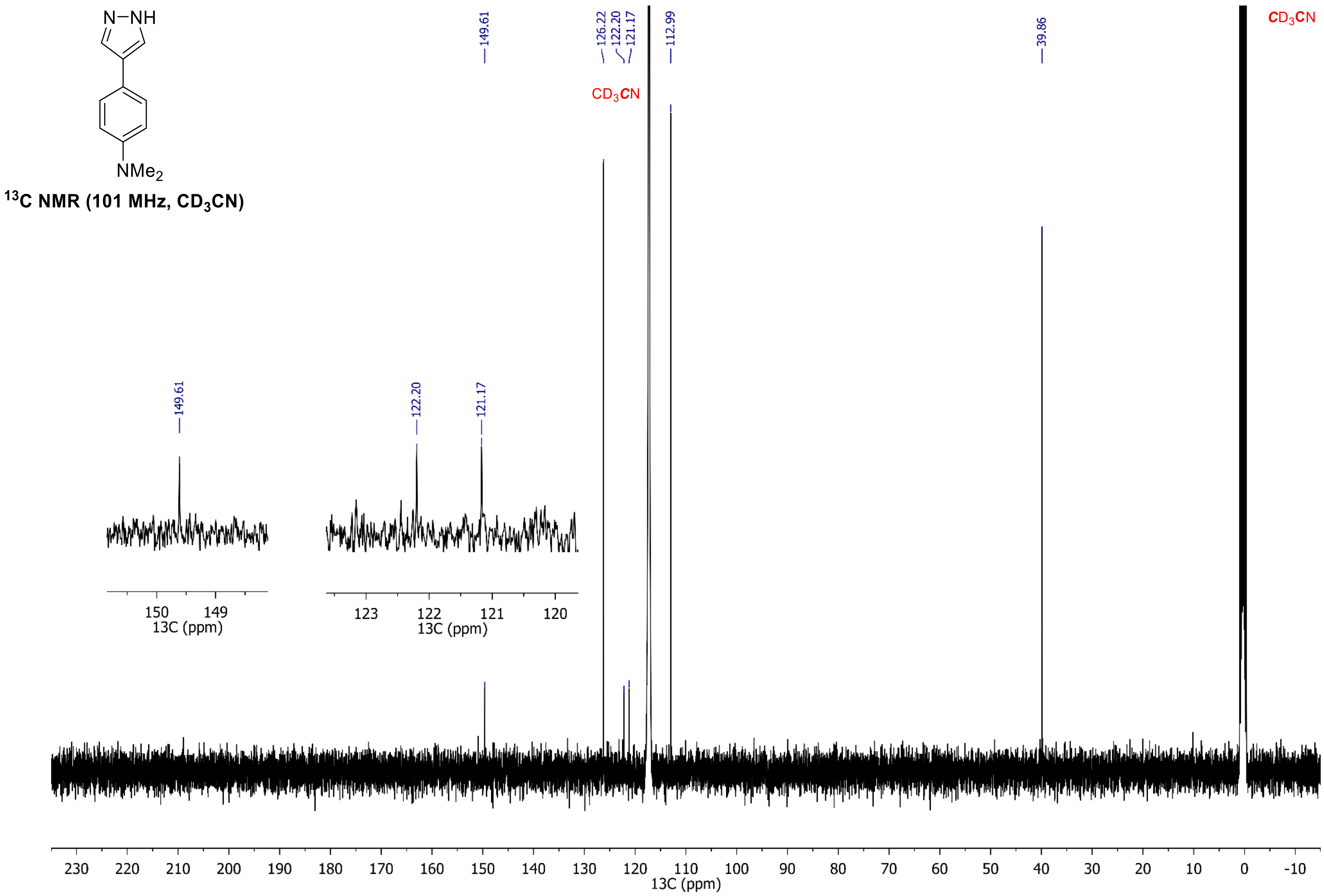
N–NH
NMe2
13C NMR (101 MHz, CD3CN)
149.61
126.22
122.20
121.17
112.99
39.86
CD3CN
CD3CN
150
149
13C (ppm)
123
122
121
120
13C (ppm)
230
220
210
200
190
180
170
160
150
140
130
120
110
100
90
80
70
60
50
40
30
20
10
0
-10
13C (ppm)

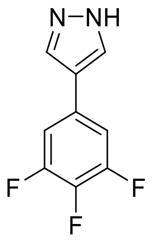

^1H NMR (400 MHz, CD_3CN)

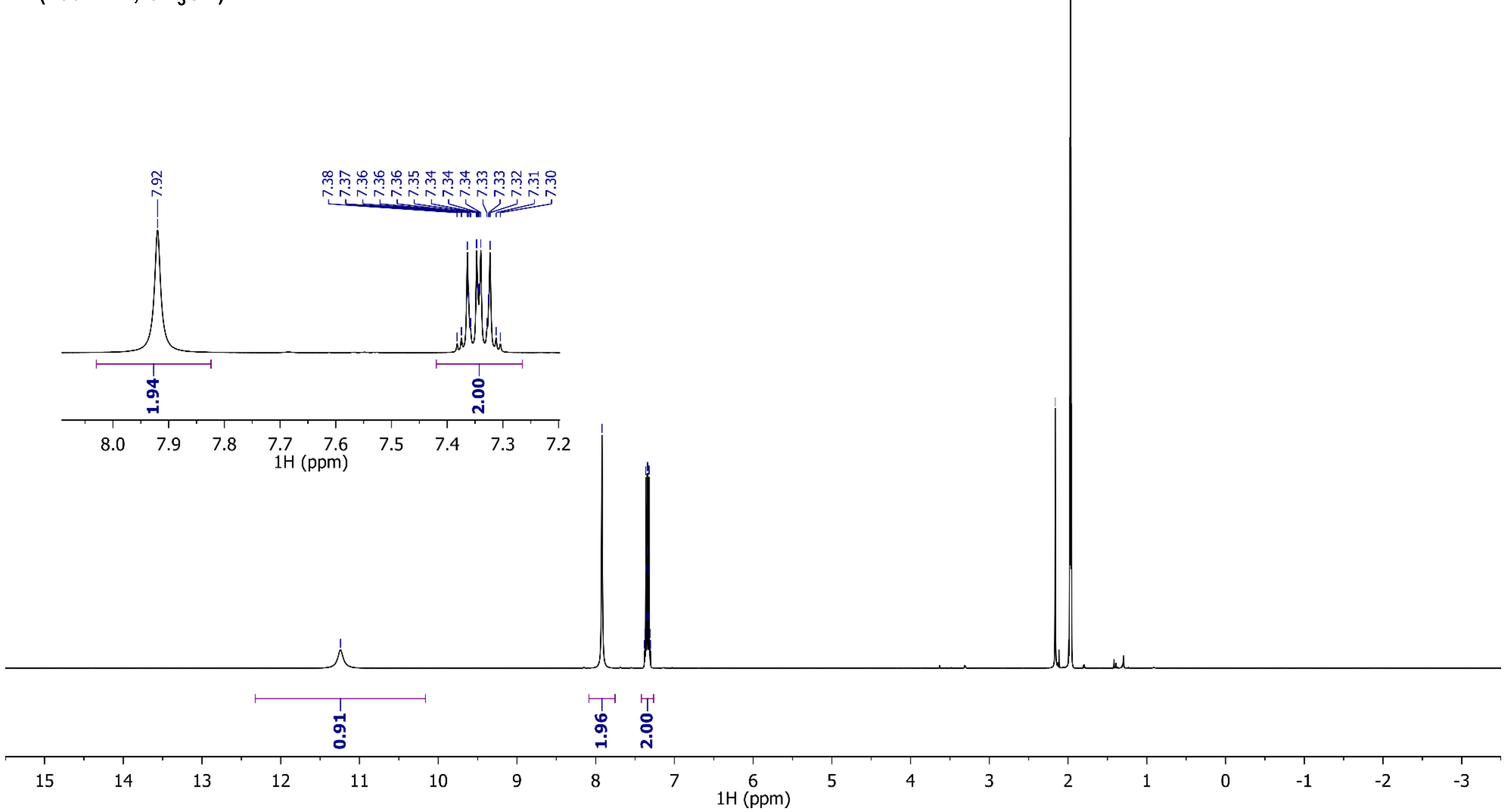

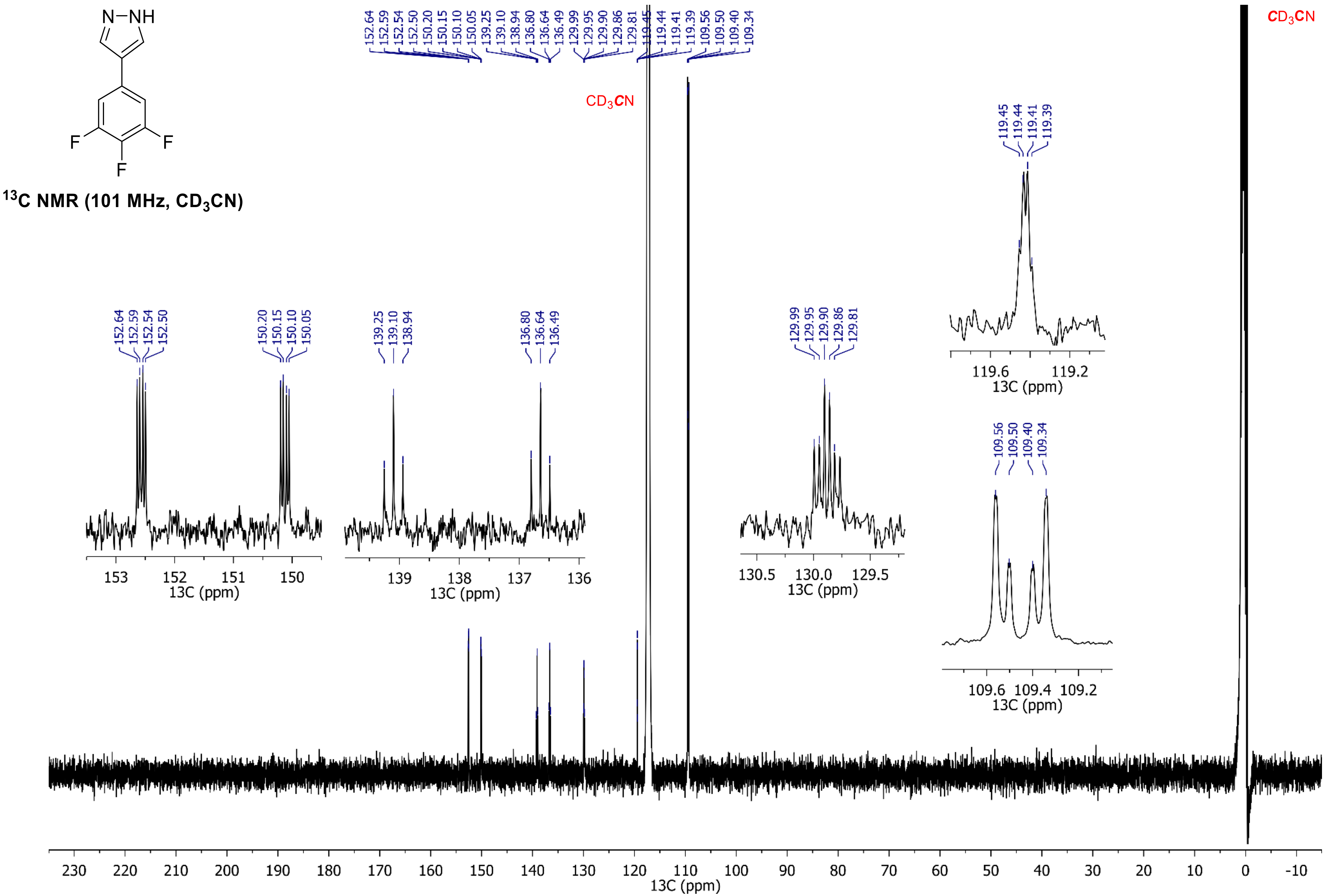
N–NH
F
F
F
13C NMR (101 MHz, CD3CN)
CD3CN
CD3CN
152.64
152.59
152.54
152.50
150.20
150.15
150.10
150.05
139.25
139.10
138.94
136.80
136.64
136.49
129.99
129.95
129.90
129.86
129.81
119.45
119.44
119.41
119.39
109.56
109.50
109.40
109.34
13C (ppm)

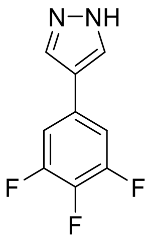

^{19}F NMR (377 MHz, CD_3CN)

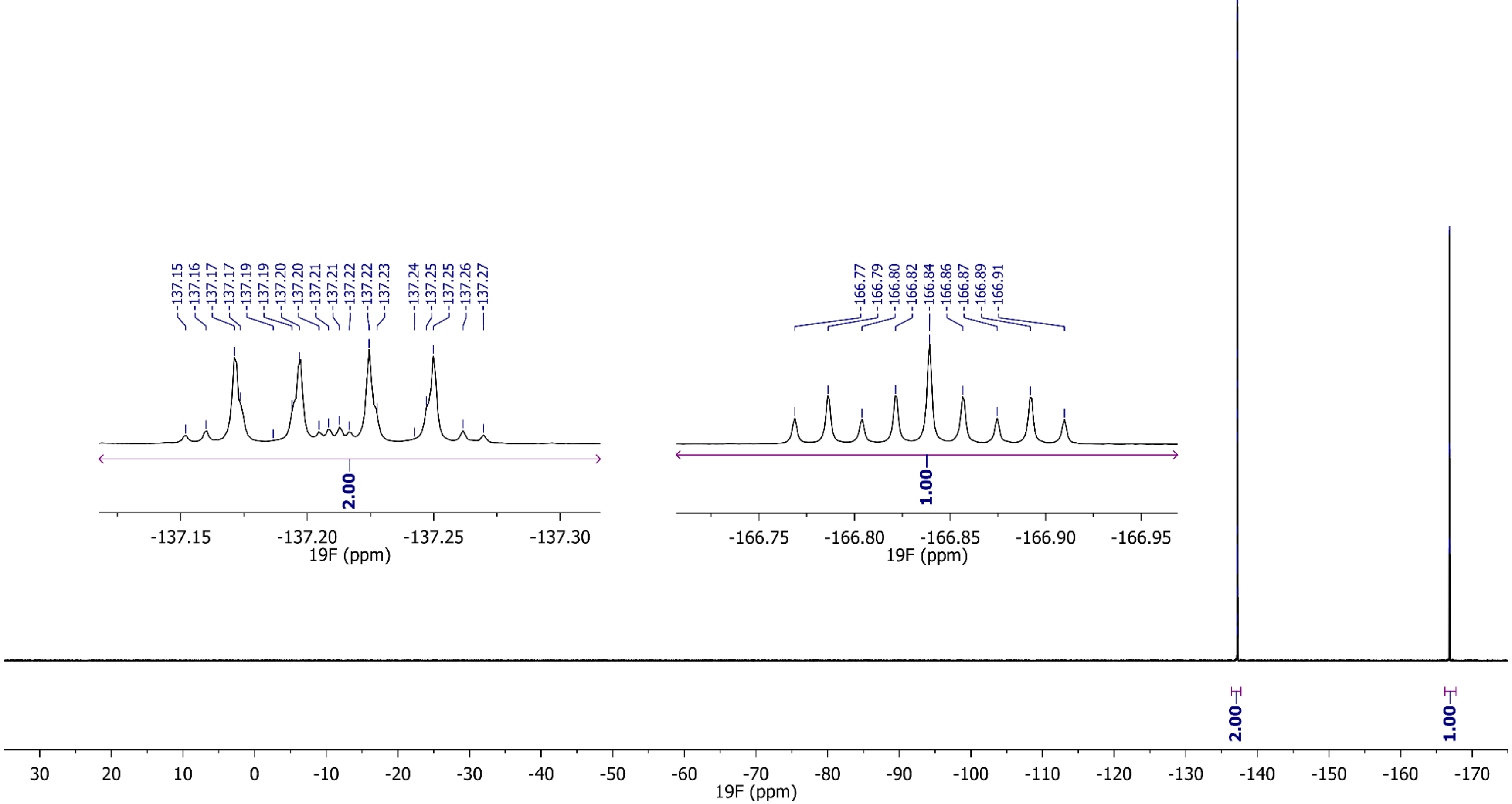

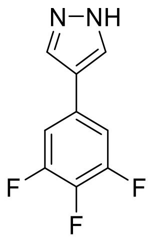

$^{19}F\{^{1}H\}$ NMR (377 MHz, CD_3CN)

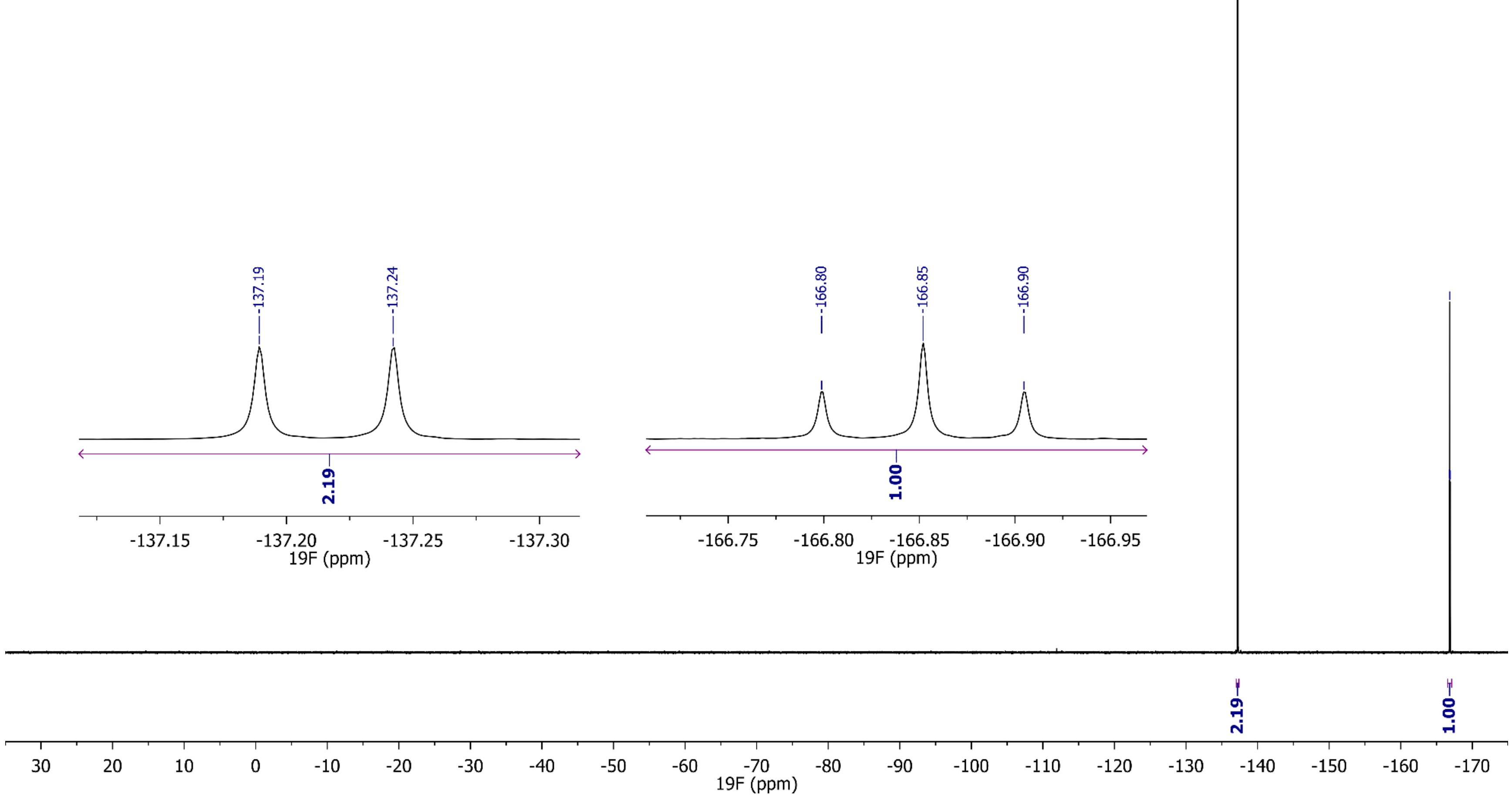

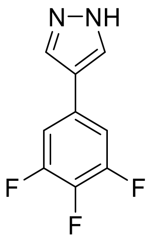

^1H NMR (400 MHz, DMSO-d_6)

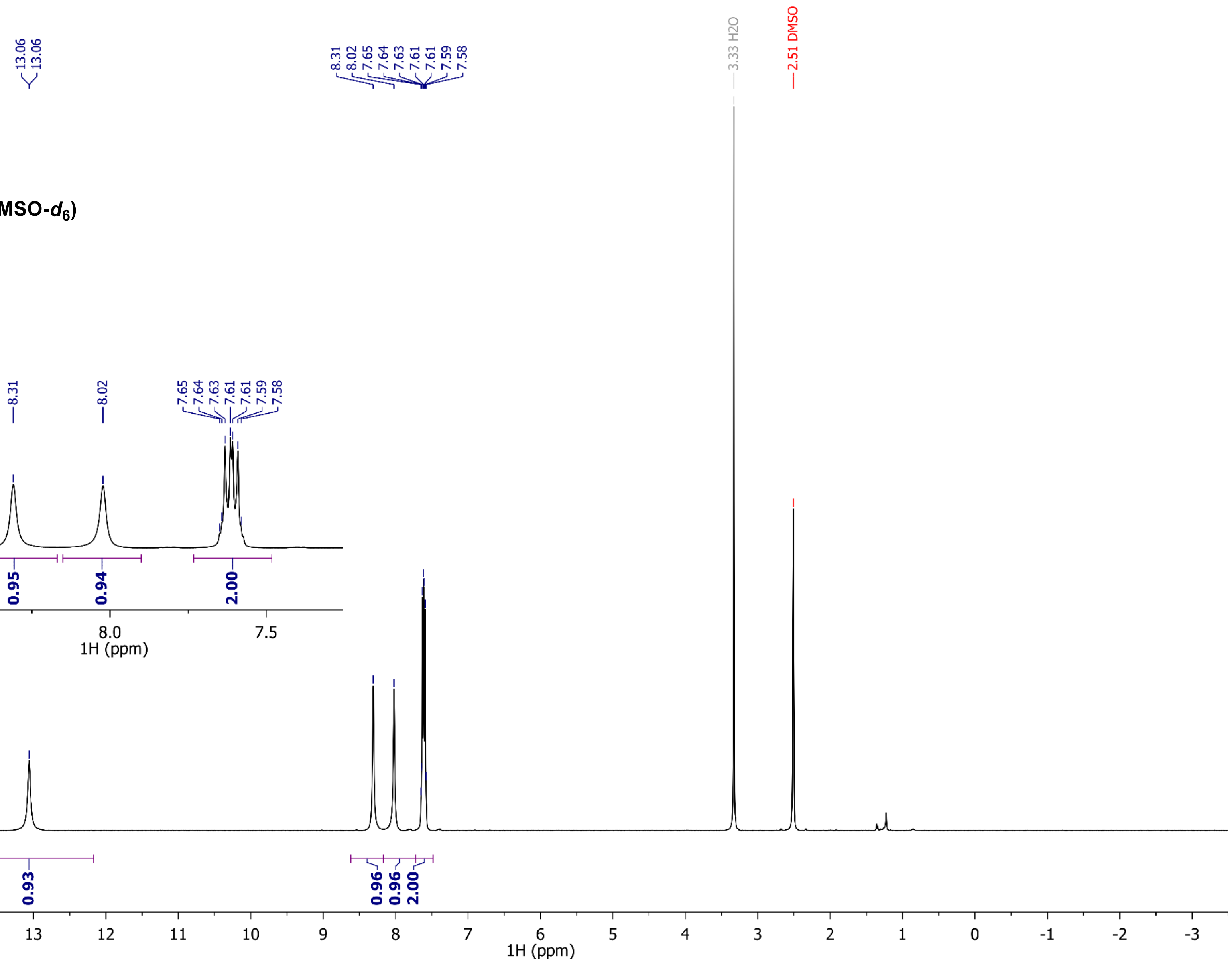

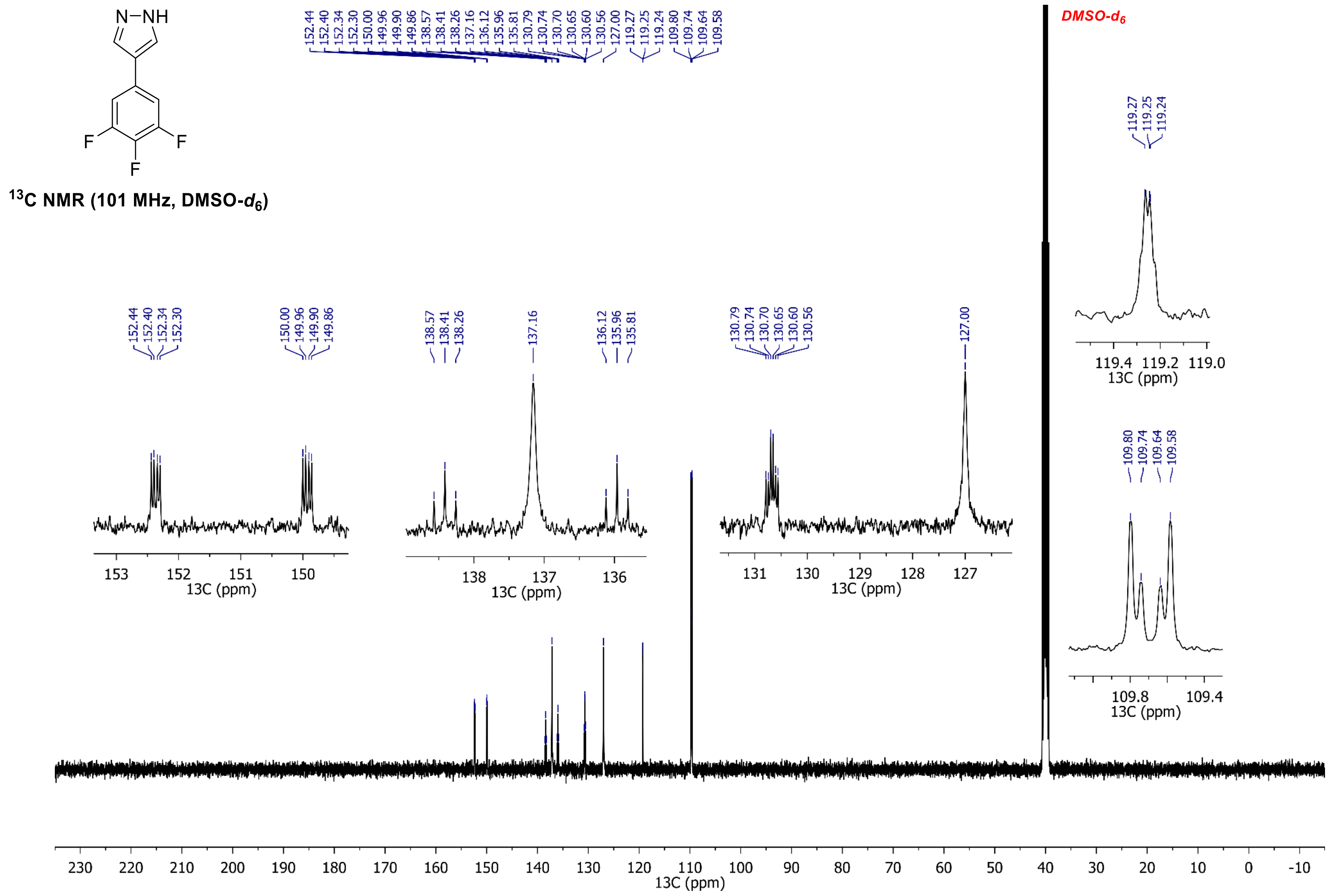

^{13}C NMR (101 MHz, DMSO-d_6)

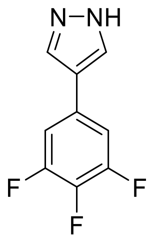

^{19}F NMR (377 MHz, DMSO-d_6)

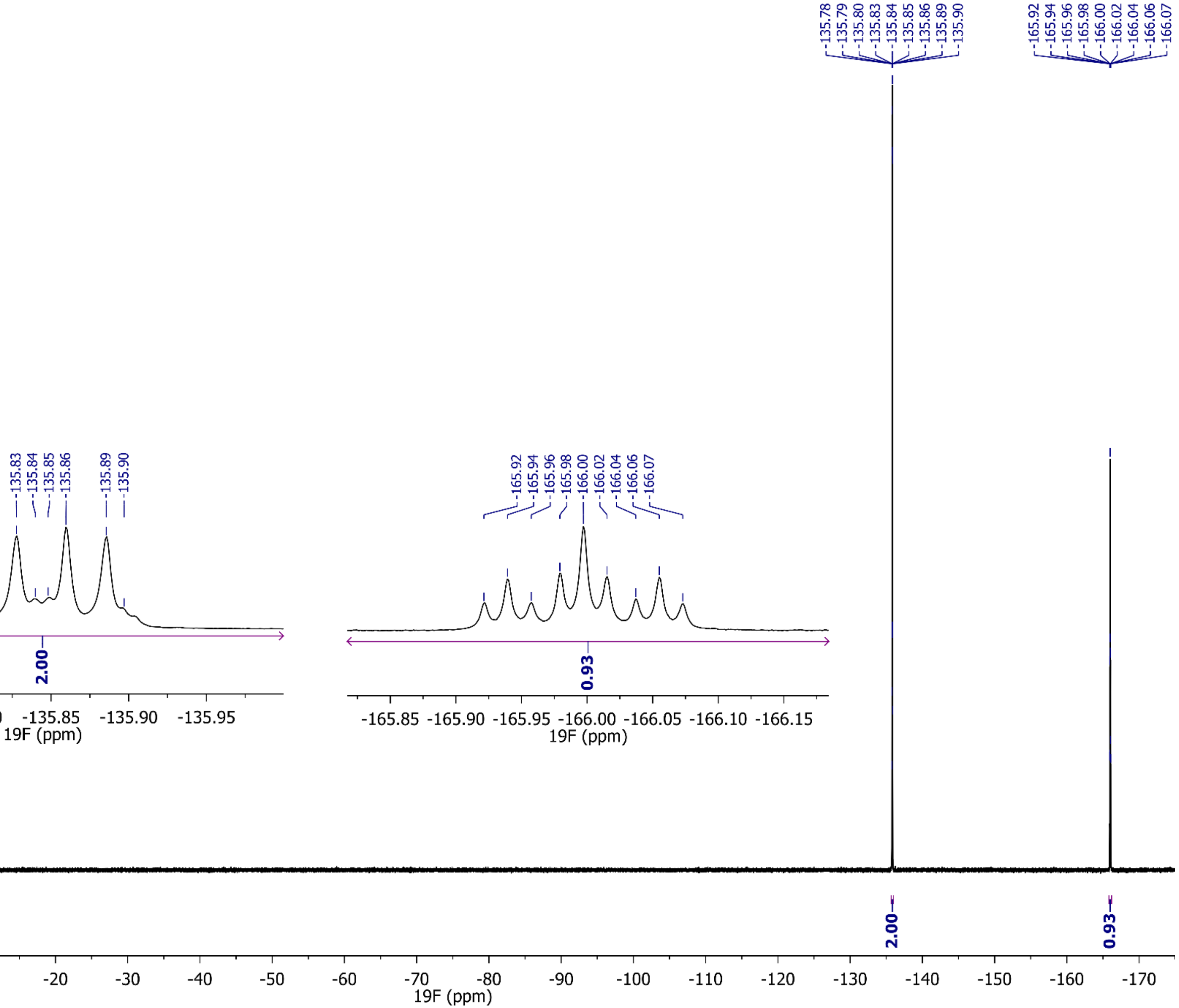

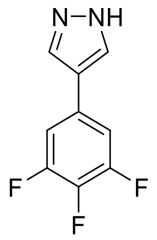

$^{19}F\{^{1}H\}$ NMR (377 MHz, DMSO-d_6)

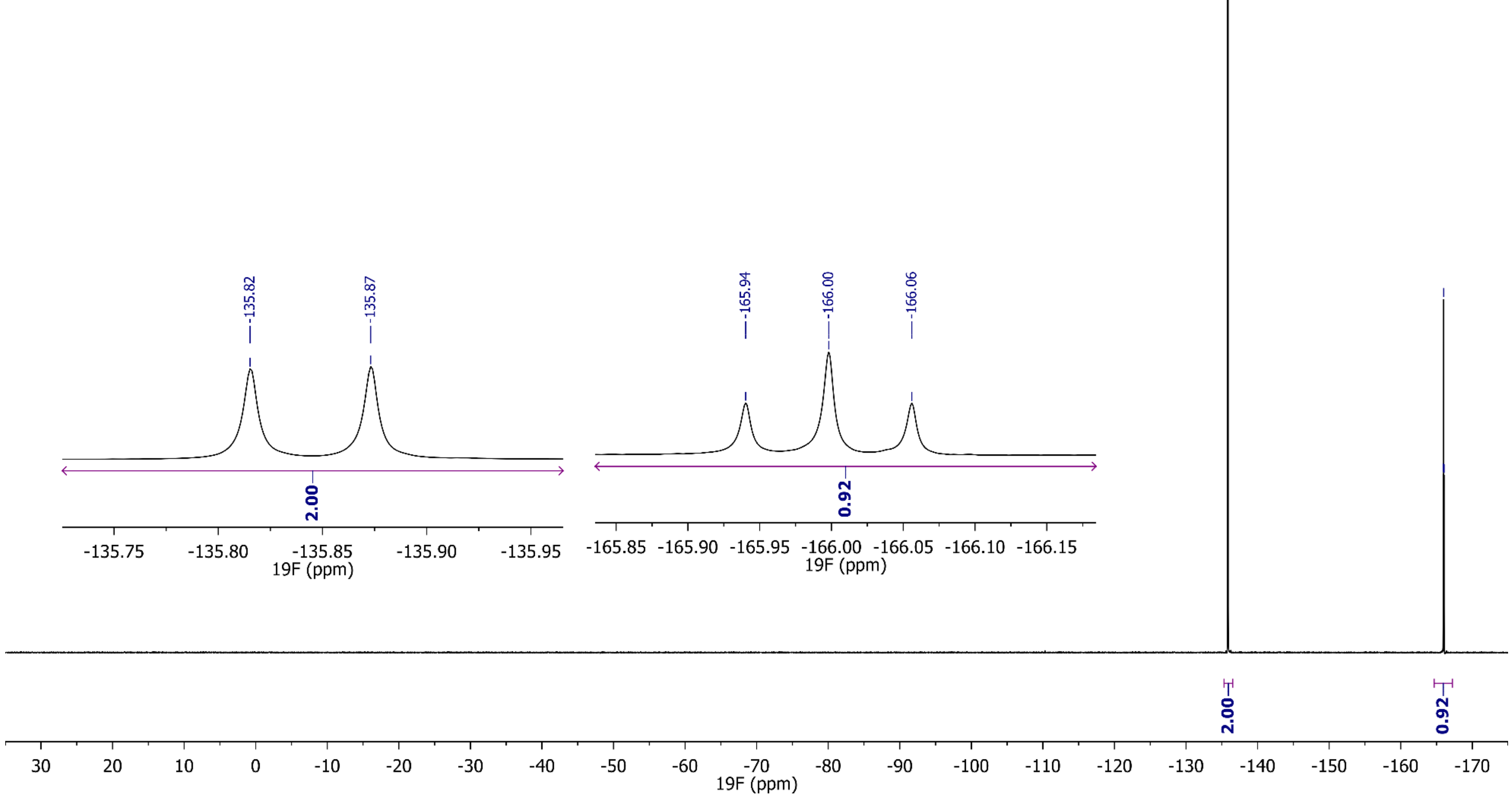

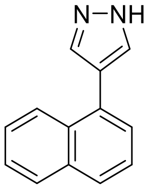

^1H NMR (400 MHz, DMSO-d_6)

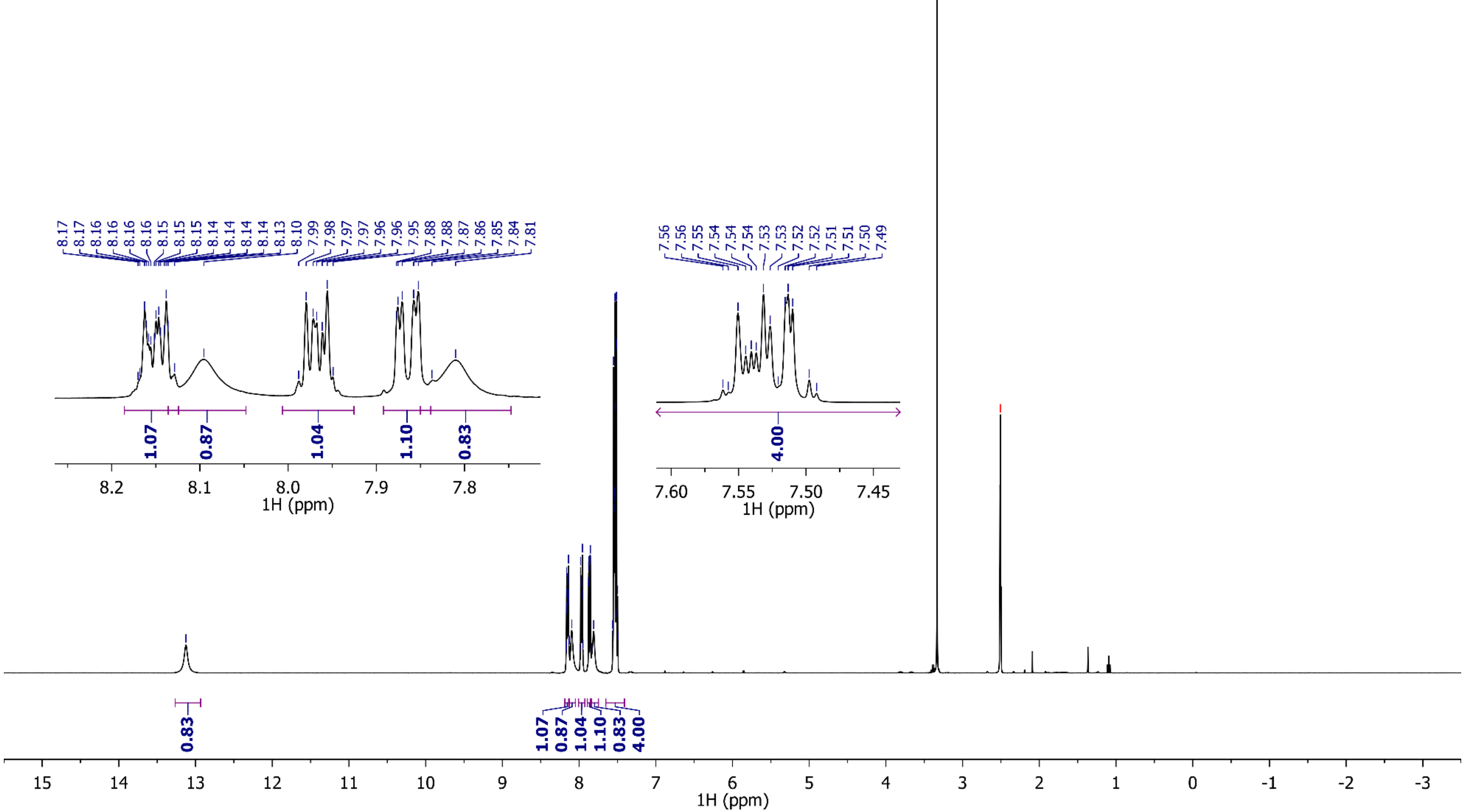

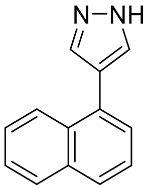

^{13}C NMR (101 MHz, DMSO-d_6))

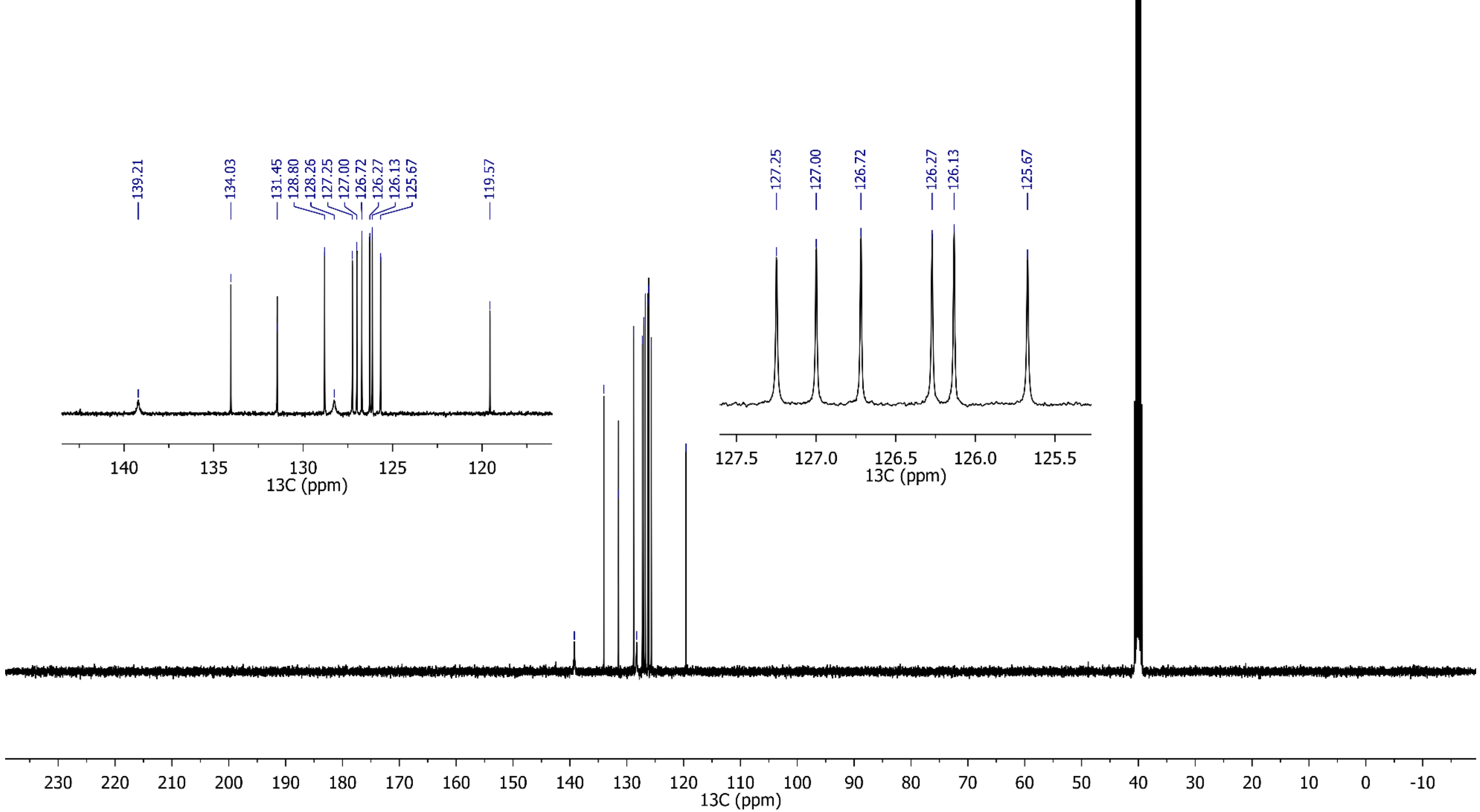

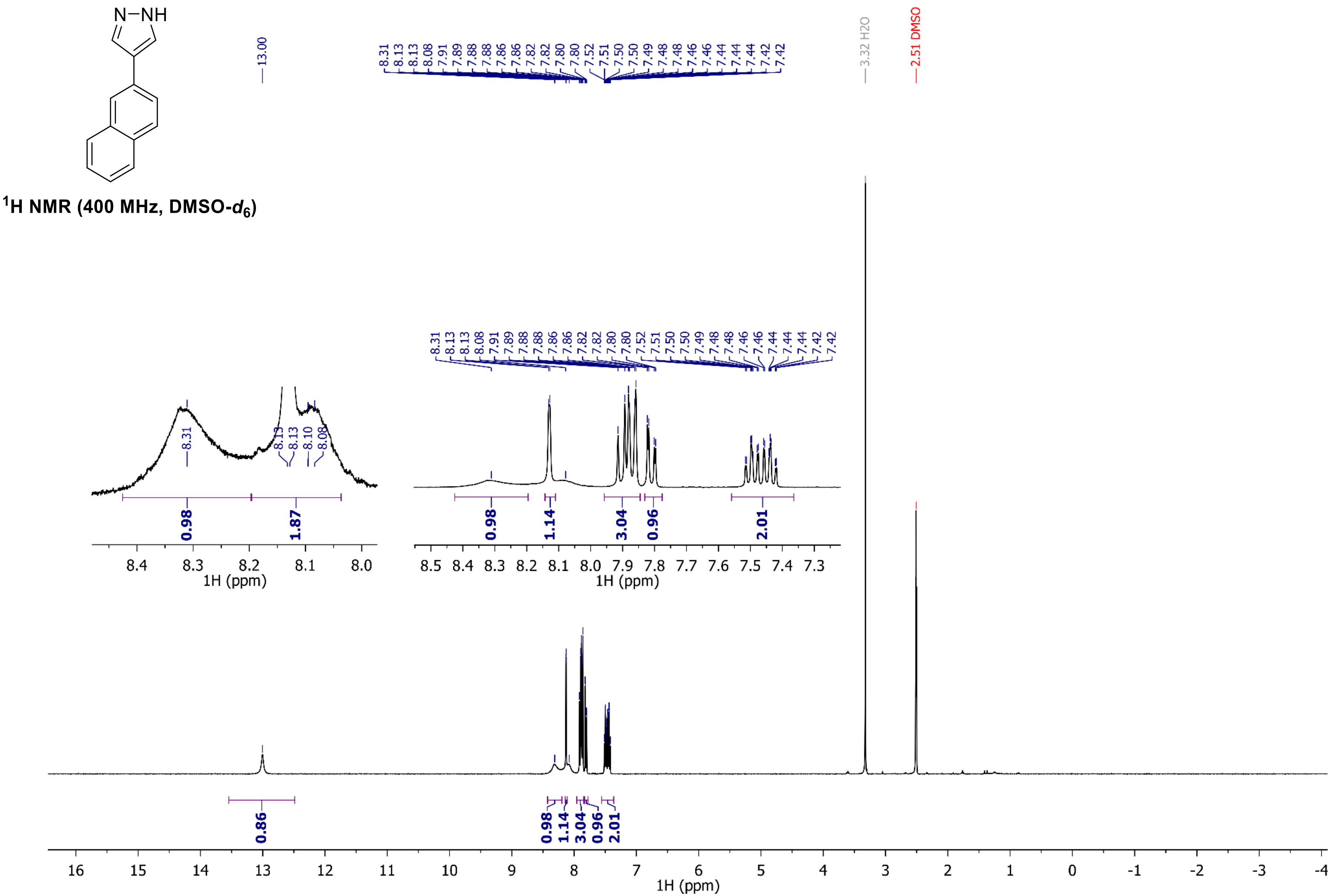
N–NH
1H NMR (400 MHz, DMSO-d6)
13.00
8.31 8.13 8.13 8.08 7.91 7.89 7.88 7.88 7.86 7.86 7.82 7.82 7.80 7.80 7.52 7.51 7.50 7.50 7.49 7.48 7.48 7.46 7.46 7.44 7.44 7.44 7.42 7.42
3.32 H2O
2.51 DMSO
0.86 0.98 1.14 3.04 0.96 2.01
1H (ppm)

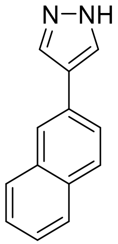

^{13}C NMR (101 MHz, DMSO-d_6)

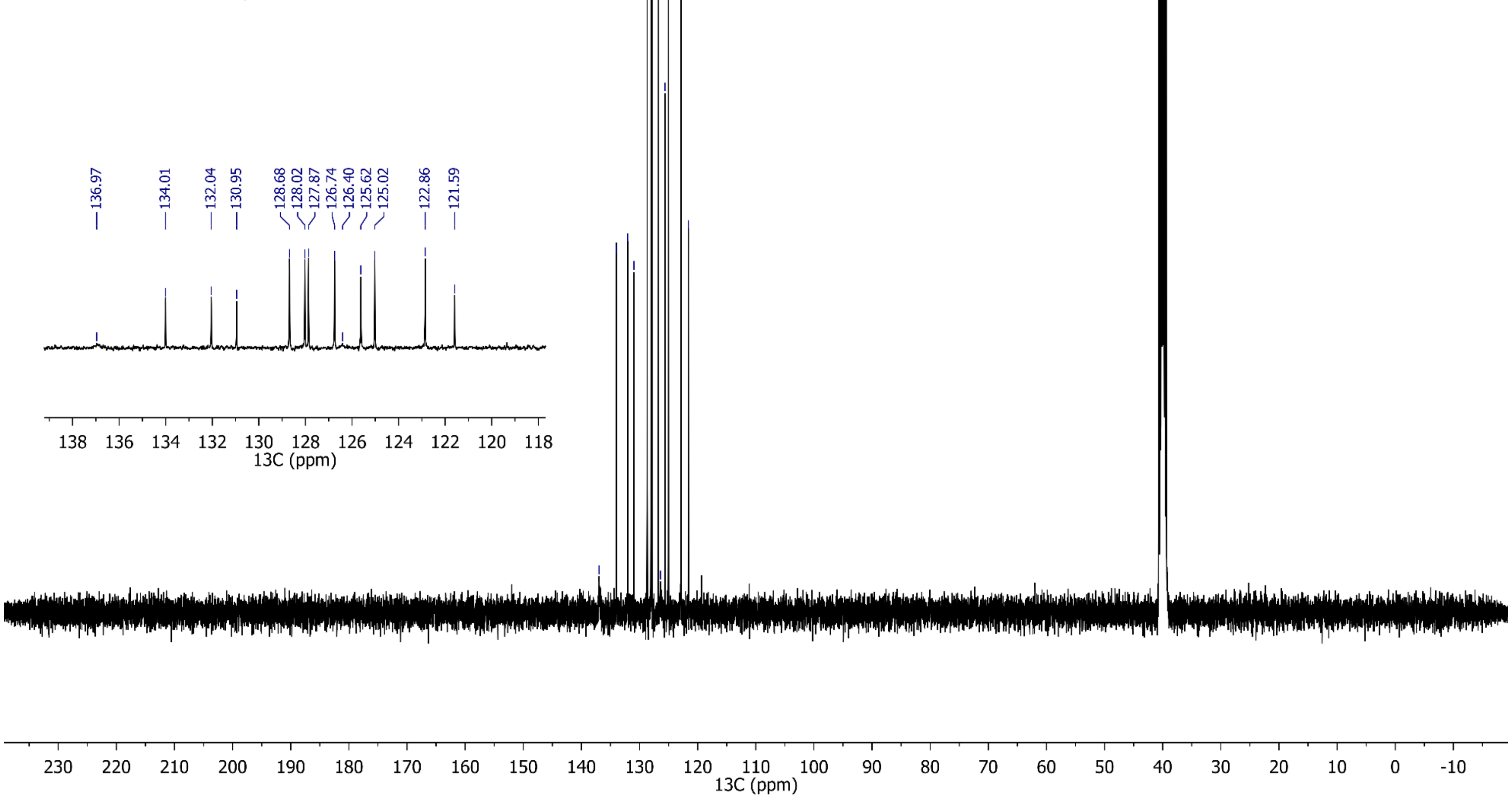

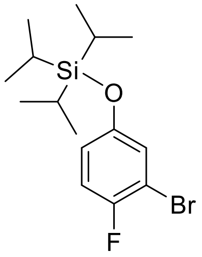

^{1}H NMR (400 MHz, $CDCl_3$)

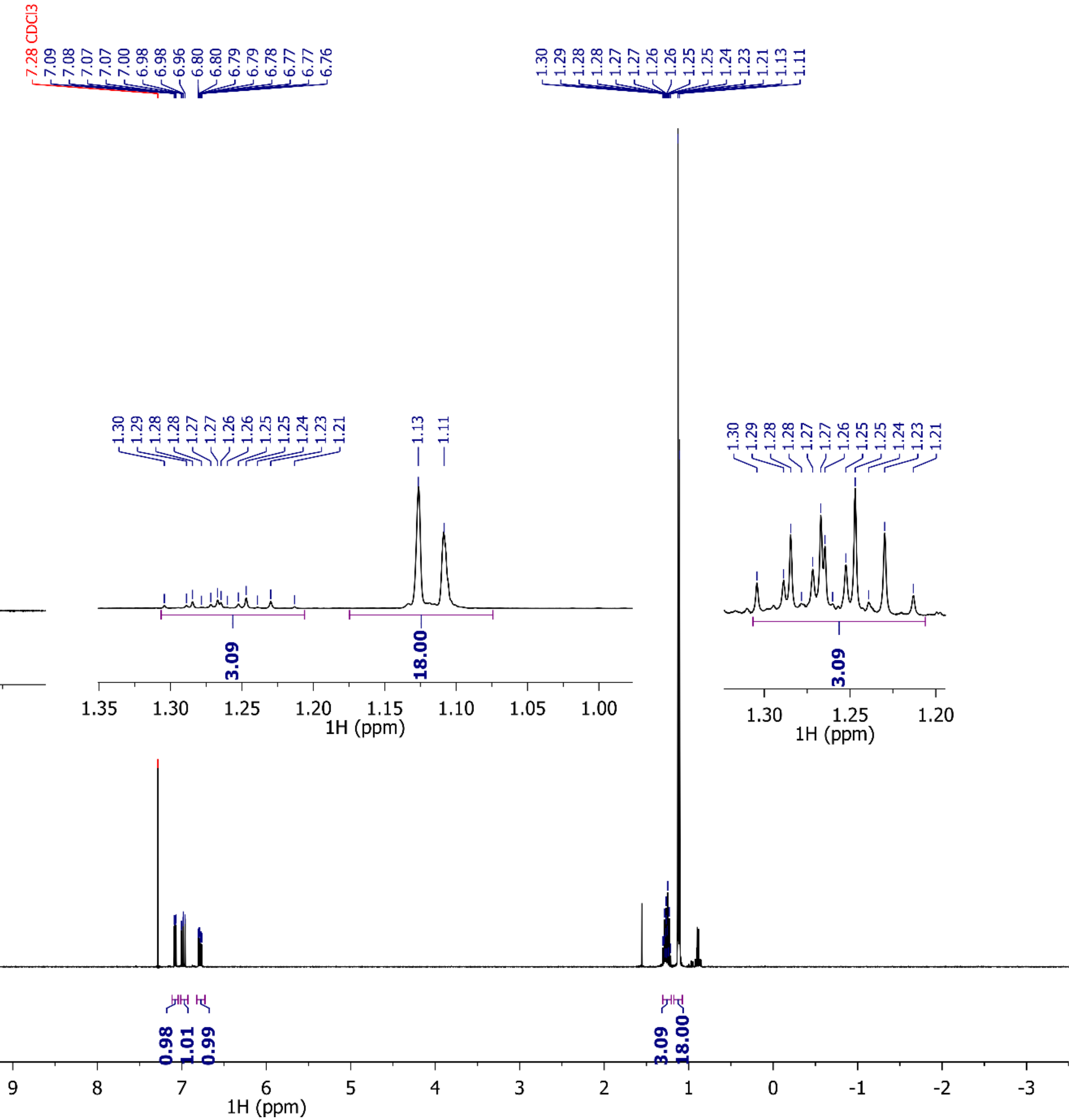

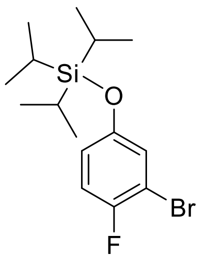

^{13}C NMR (101 MHz, $CDCl_3$)

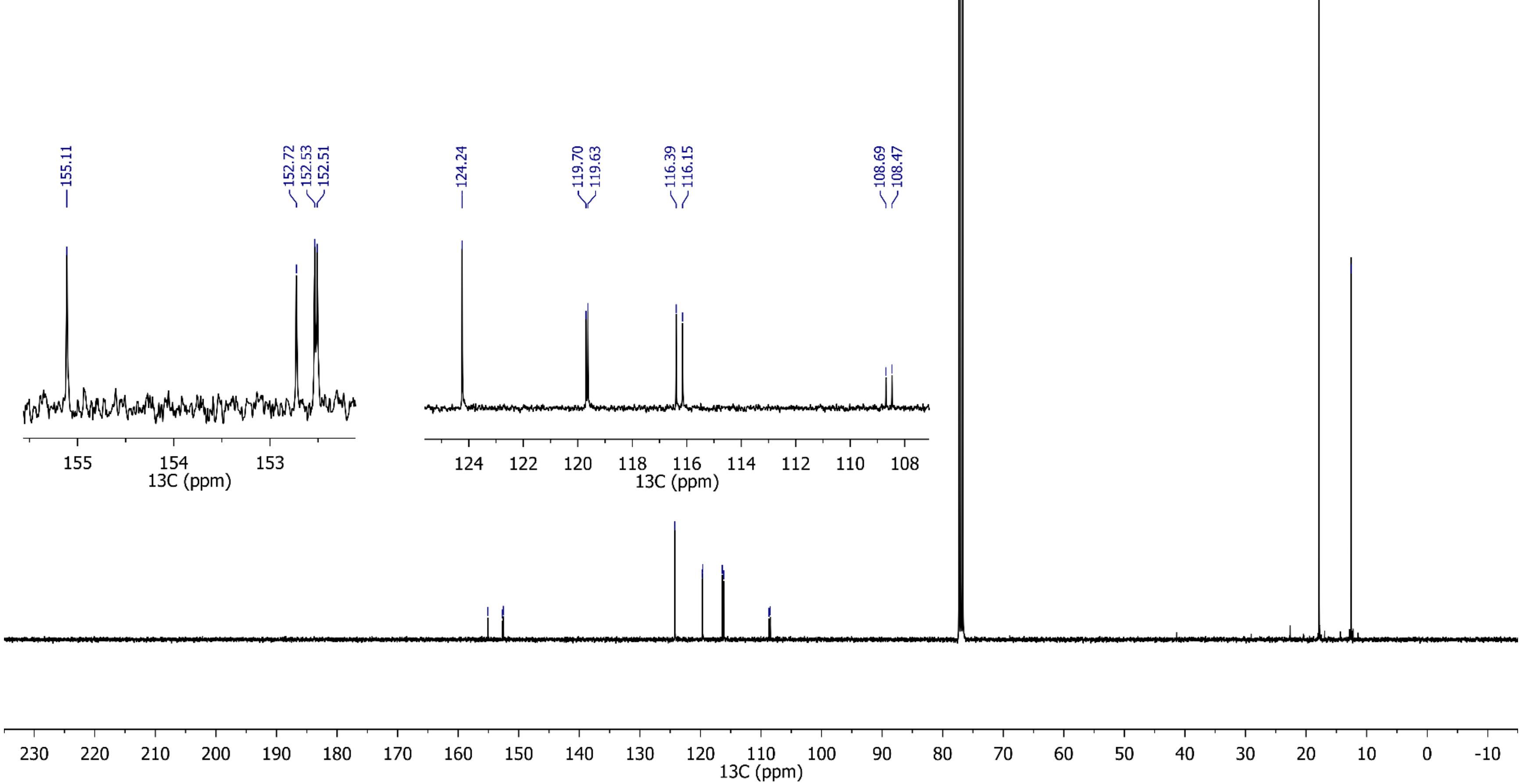

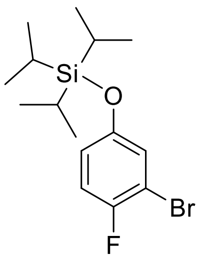

^{19}F NMR (377 MHz, CDCl$_3$)

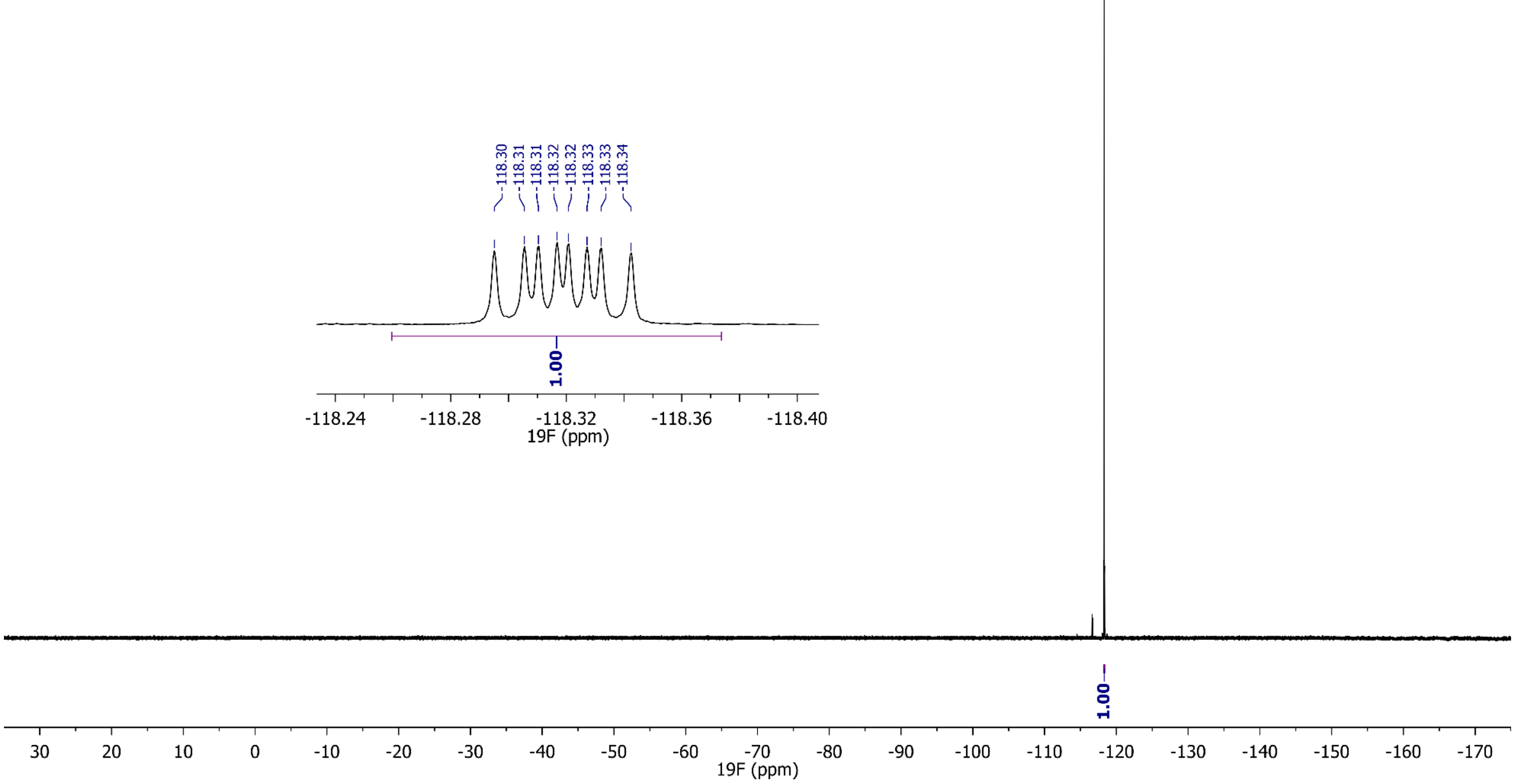

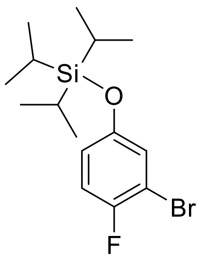

$^{29}Si\{^{1}H\}$ NMR (80 MHz, $CDCl_3$)

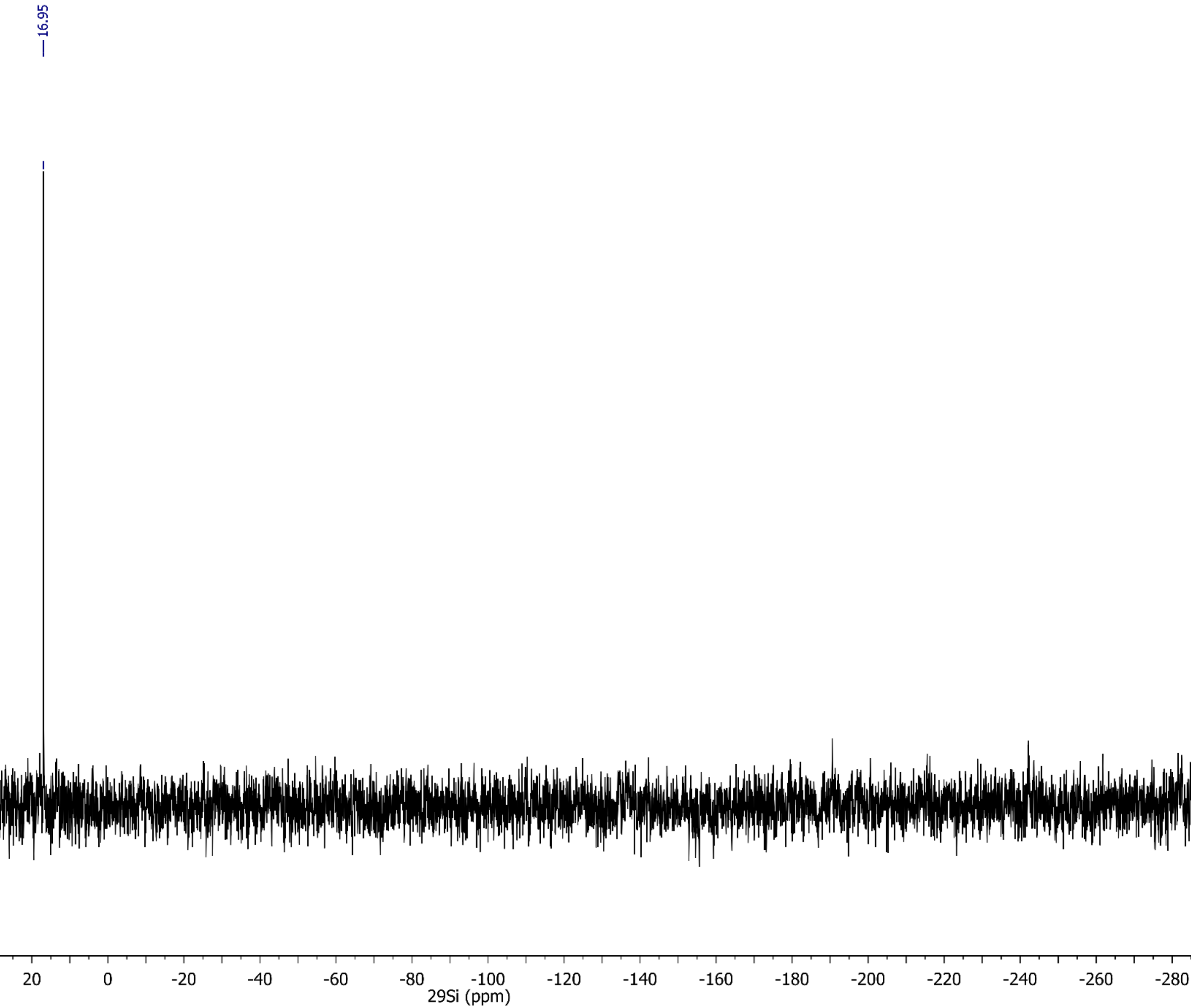

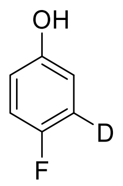

^{1}H NMR (400 MHz, $CDCl_3$)

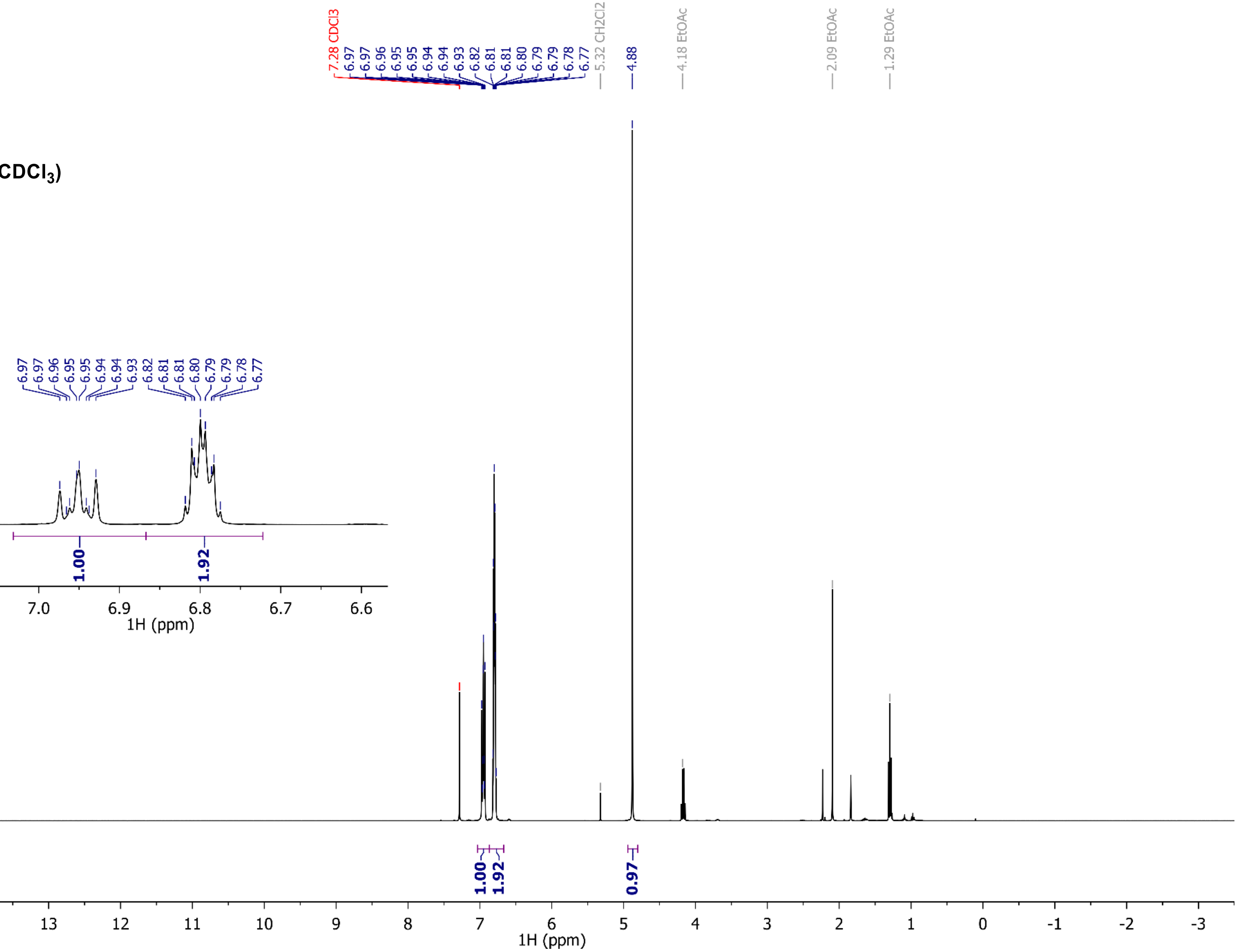

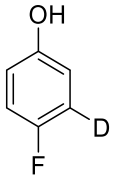

^{13}C NMR (101 MHz, $CDCl_3$)

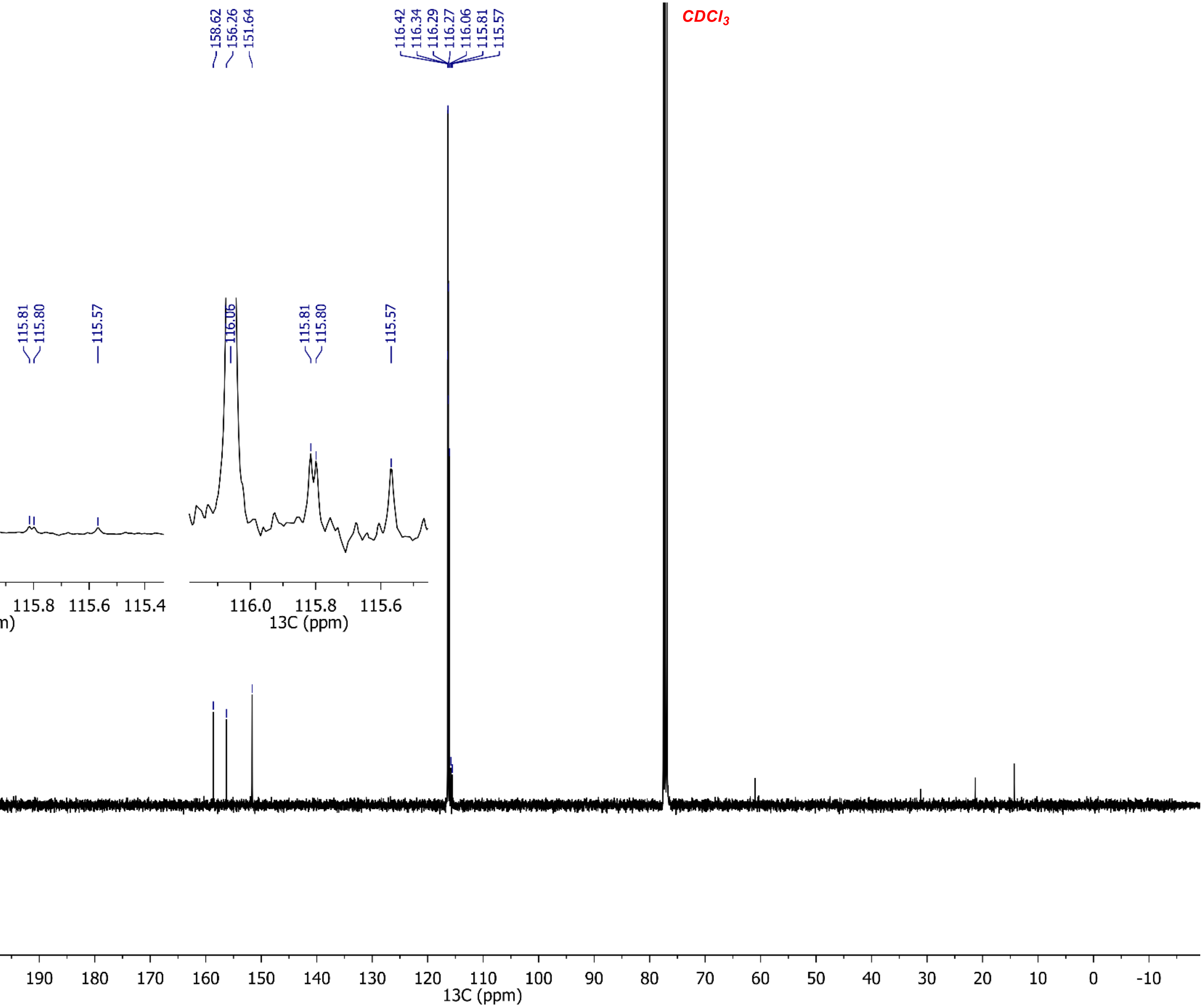

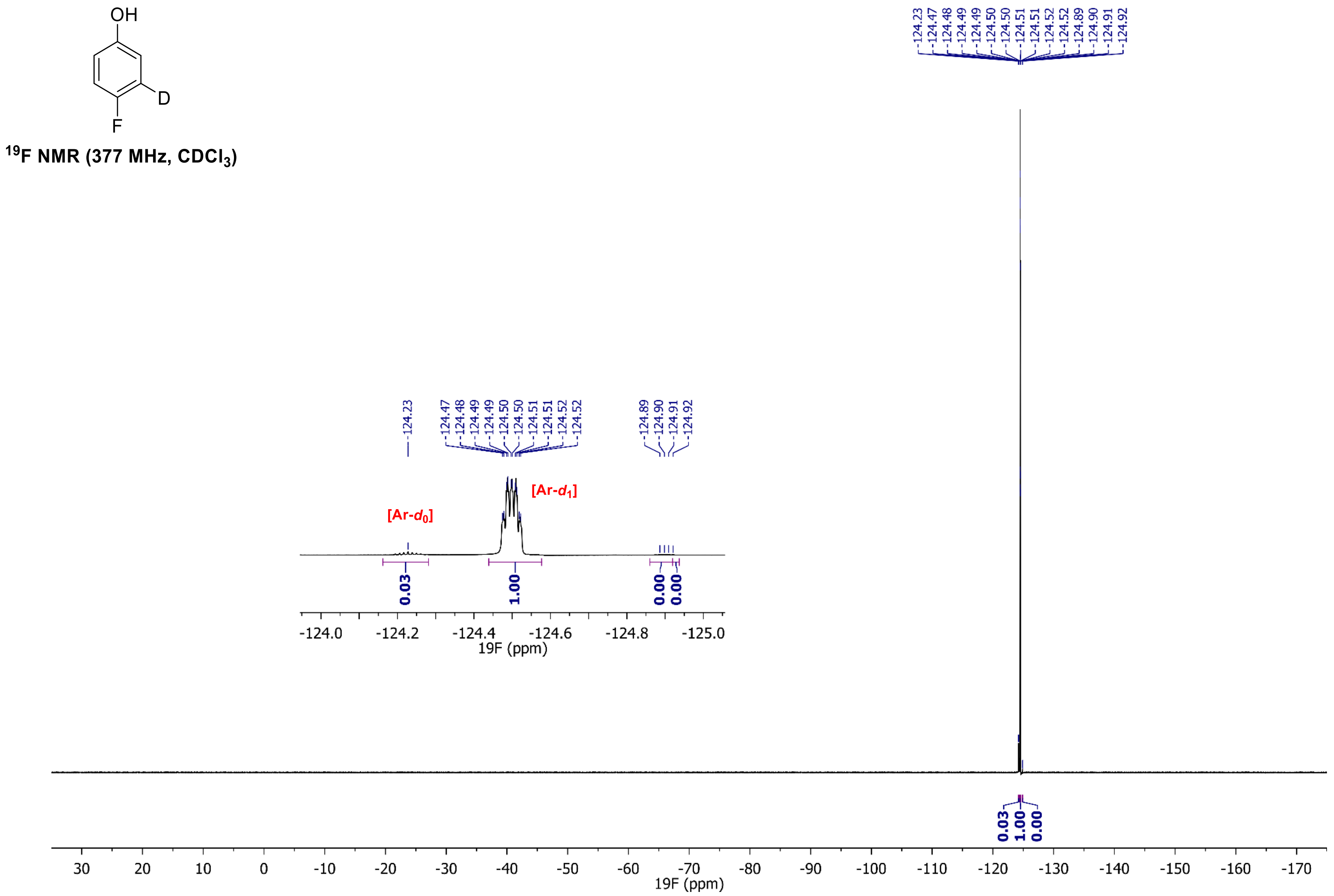

OH
D
F
^{19}F NMR (377 MHz, CDCl$_3$)
-124.23
-124.47
-124.48
-124.49
-124.49
-124.50
-124.50
-124.51
-124.51
-124.52
-124.52
-124.89
-124.90
-124.91
-124.92
[Ar-d_0]
[Ar-d_1]
0.03
1.00
0.00
0.00
-124.0
-124.2
-124.4
-124.6
-124.8
-125.0
19F (ppm)
0.03
1.00
0.00
30
20
10
0
-10
-20
-30
-40
-50
-60
-70
-80
-90
-100
-110
-120
-130
-140
-150
-160
-170
19F (ppm)

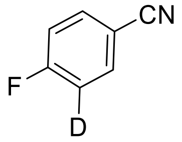

^{1}H NMR (400 MHz, $CDCl_3$)

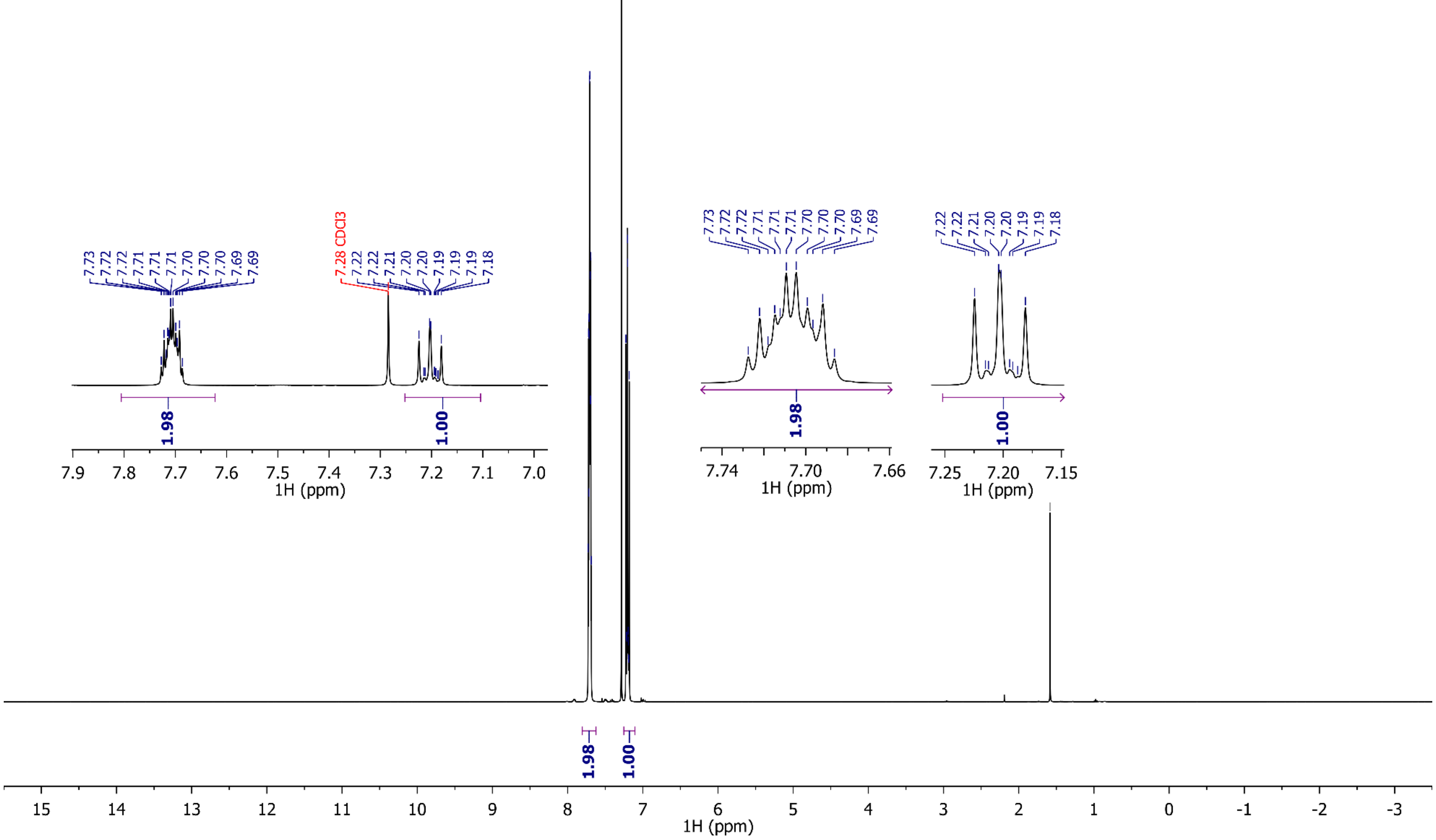

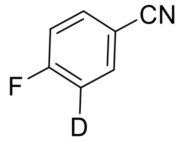

^{13}C NMR (101 MHz, $CDCl_3$)

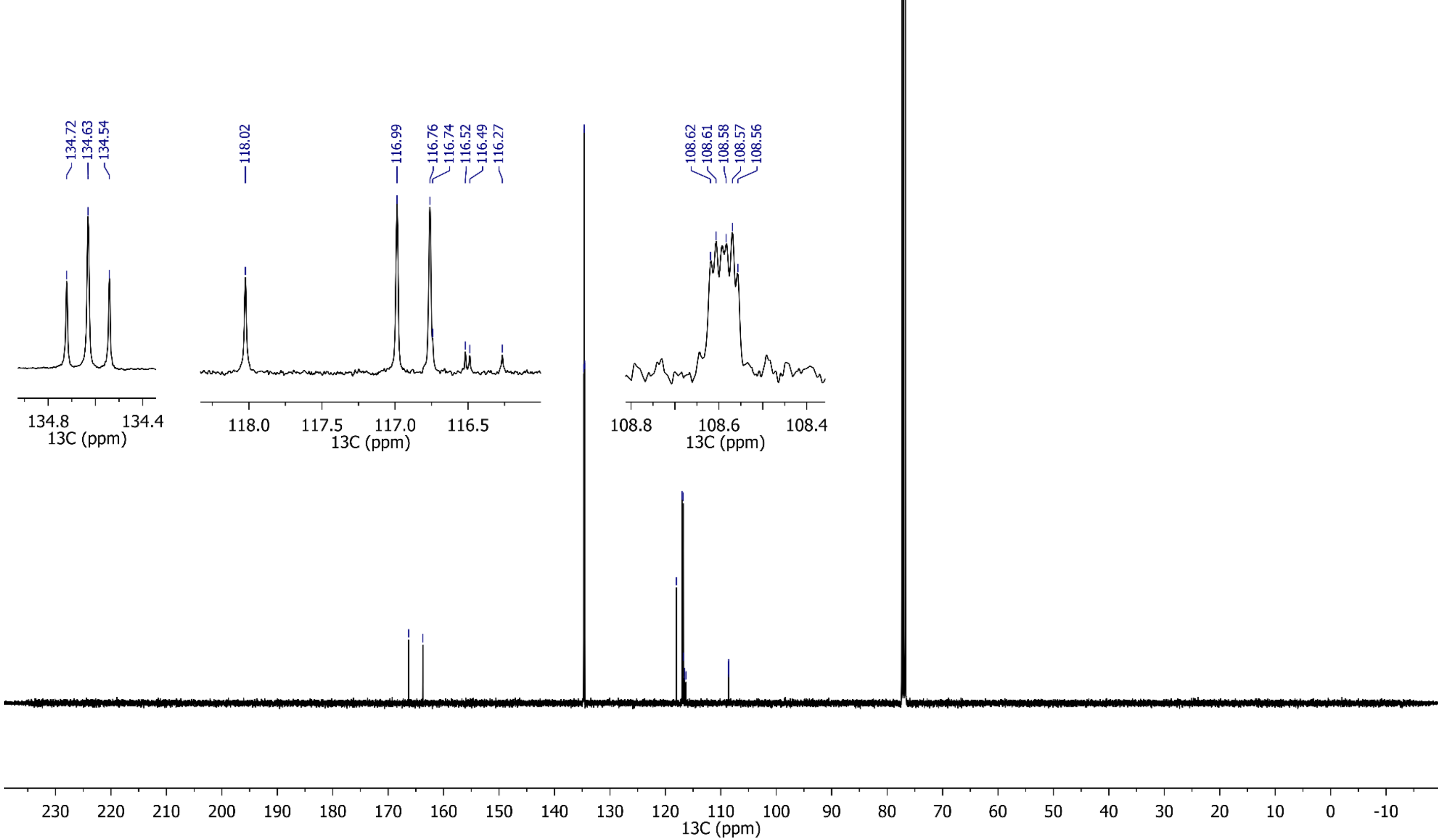

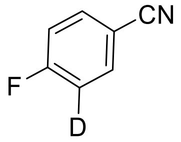

^{19}F NMR (377 MHz, $CDCl_3$)

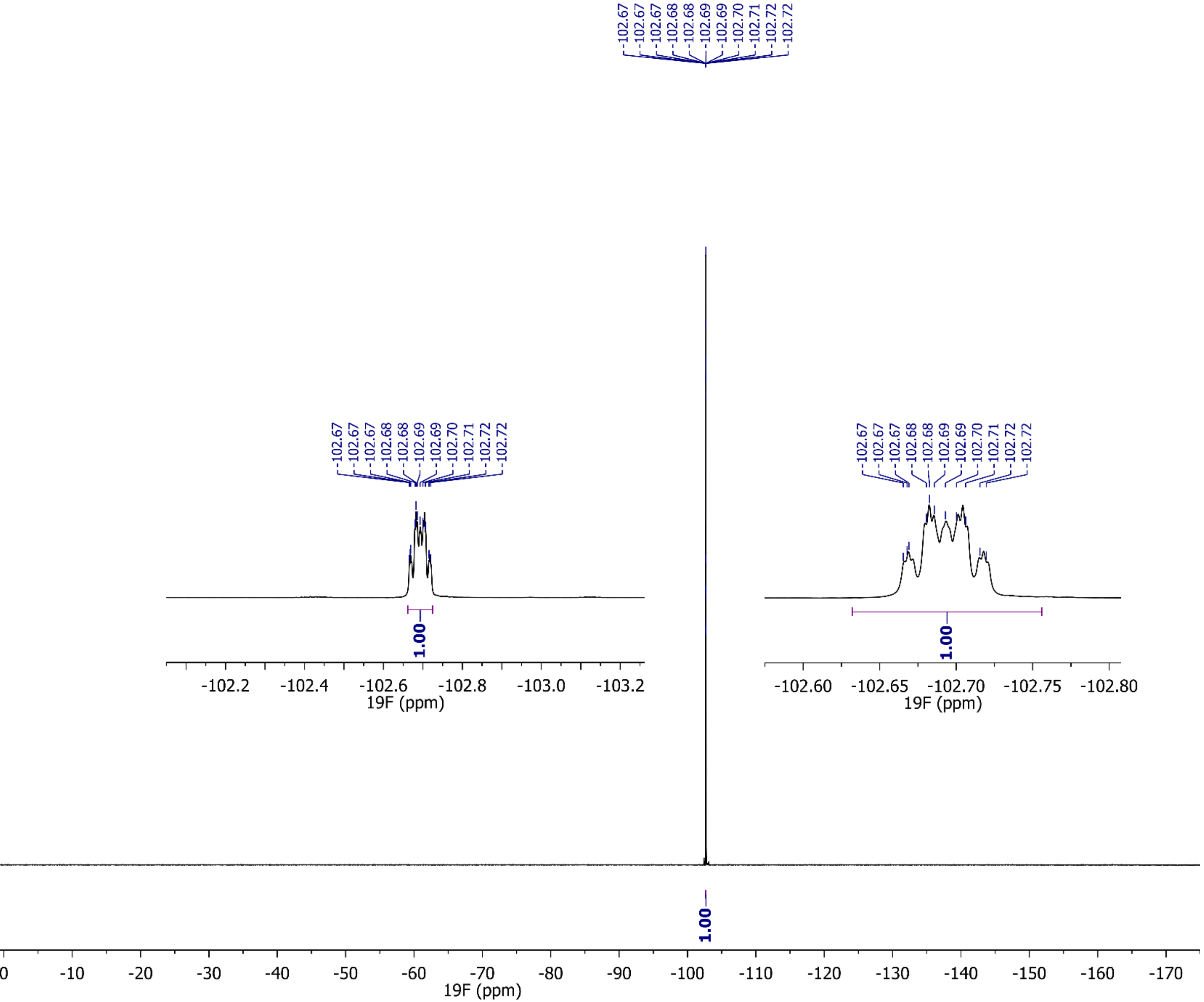

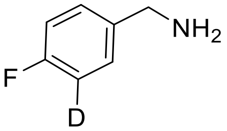

^{1}H NMR (400 MHz, CD_3CN)

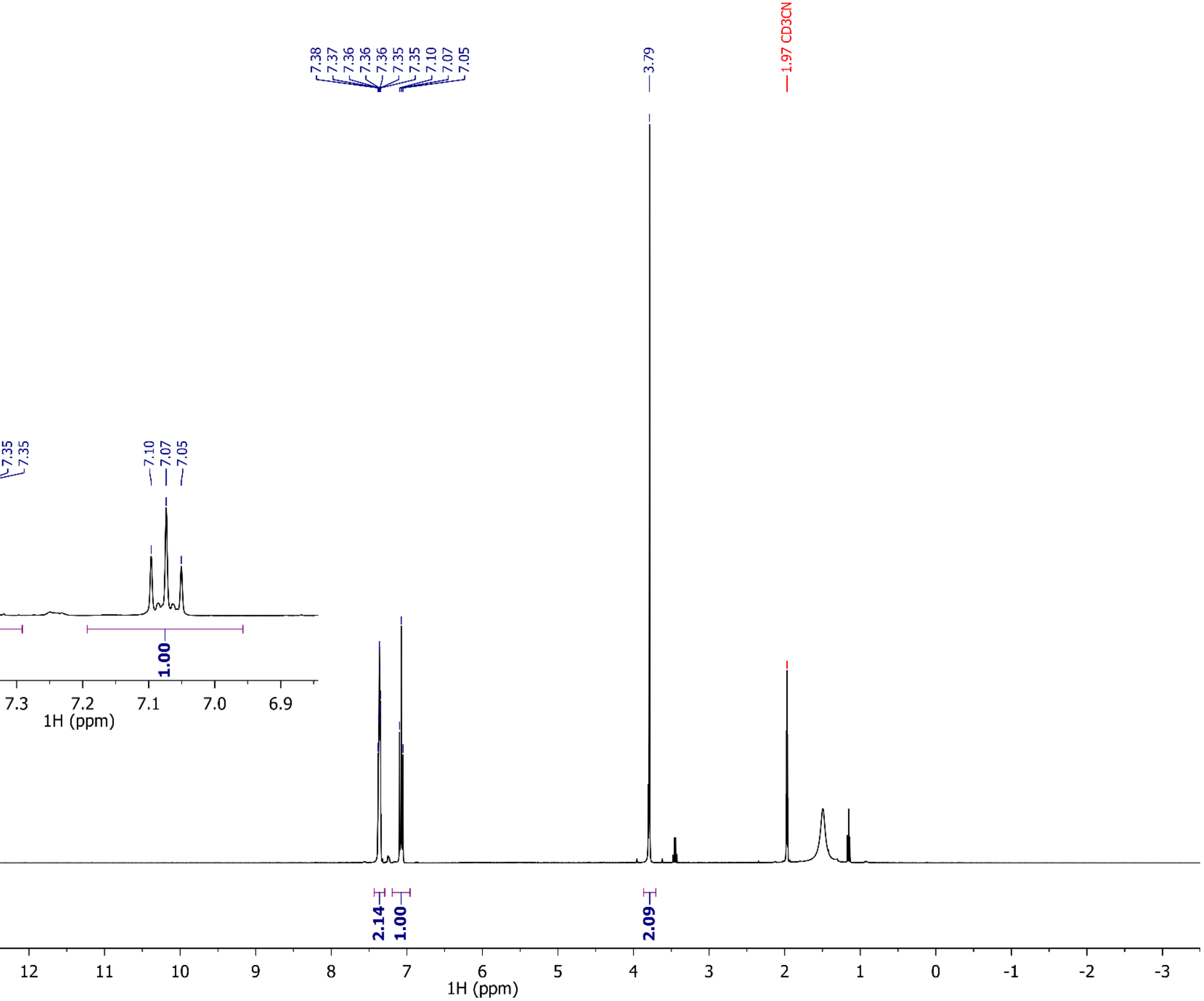

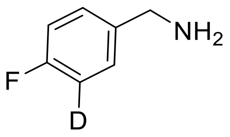

^{13}C NMR (101 MHz, CD_3CN)

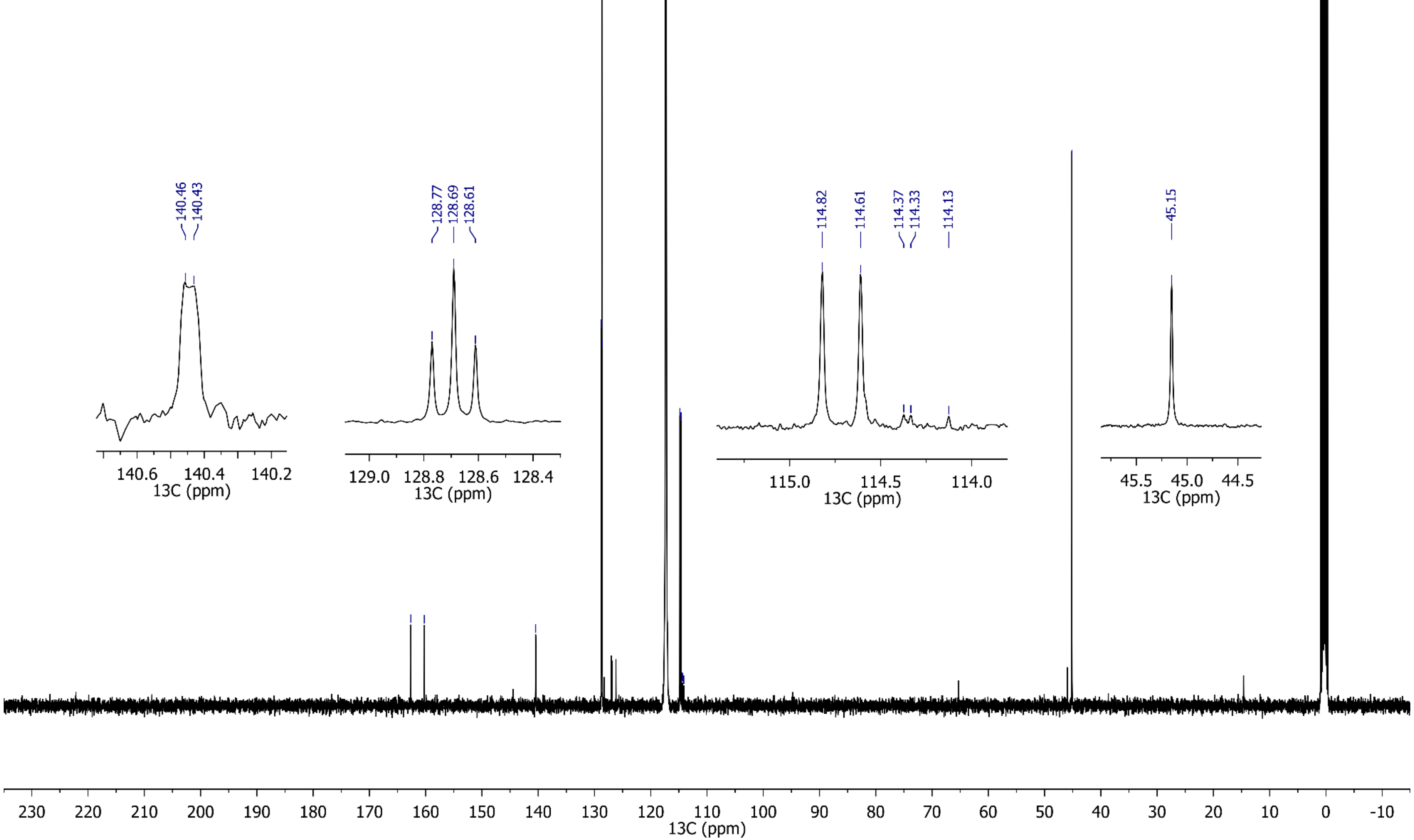

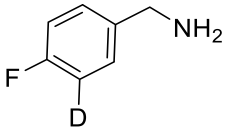

^{19}F NMR (377 MHz, CD_3CN)

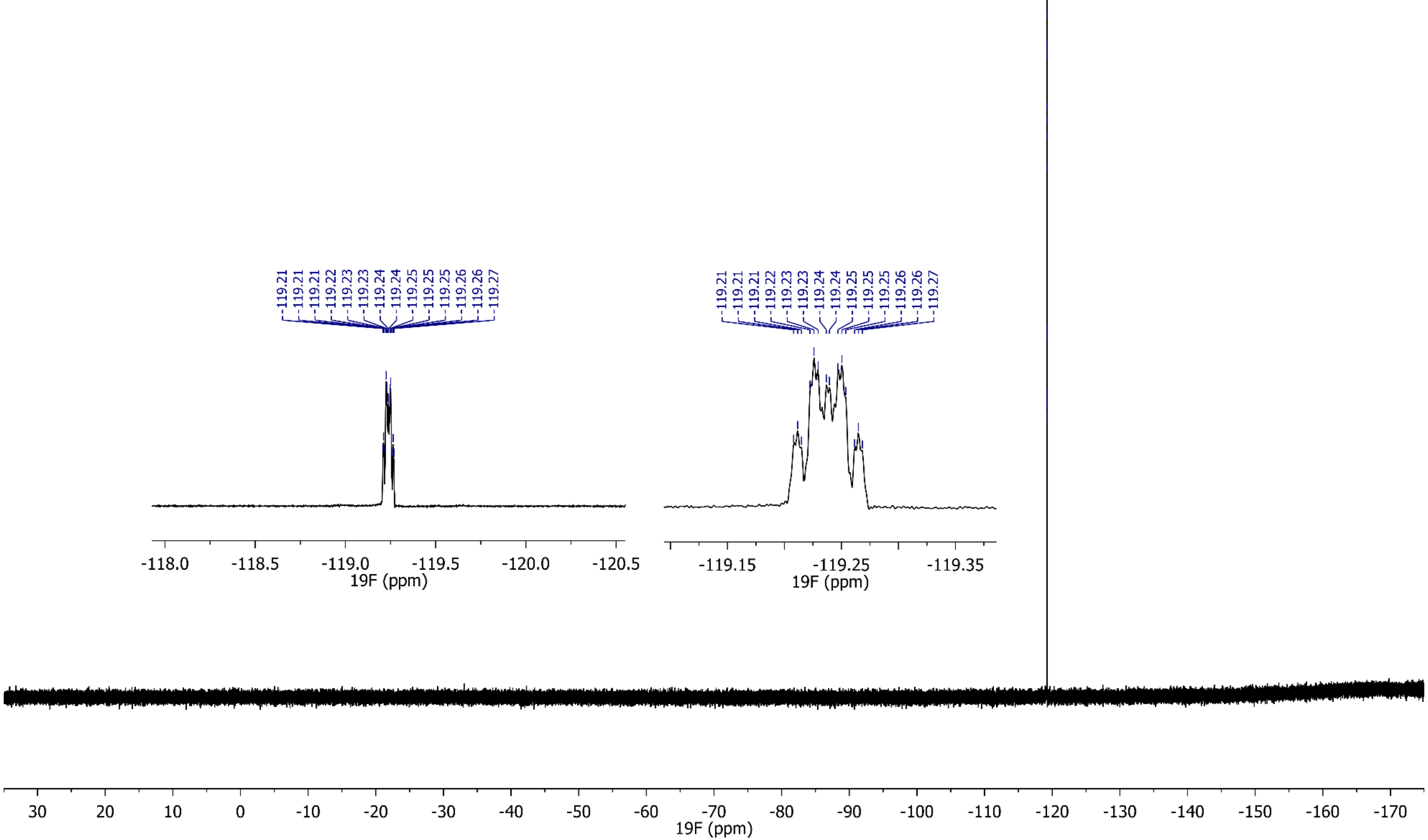

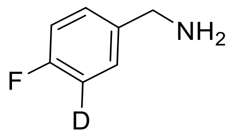

$^{19}F\{^1H\}$ NMR (377 MHz, CD_3CN)

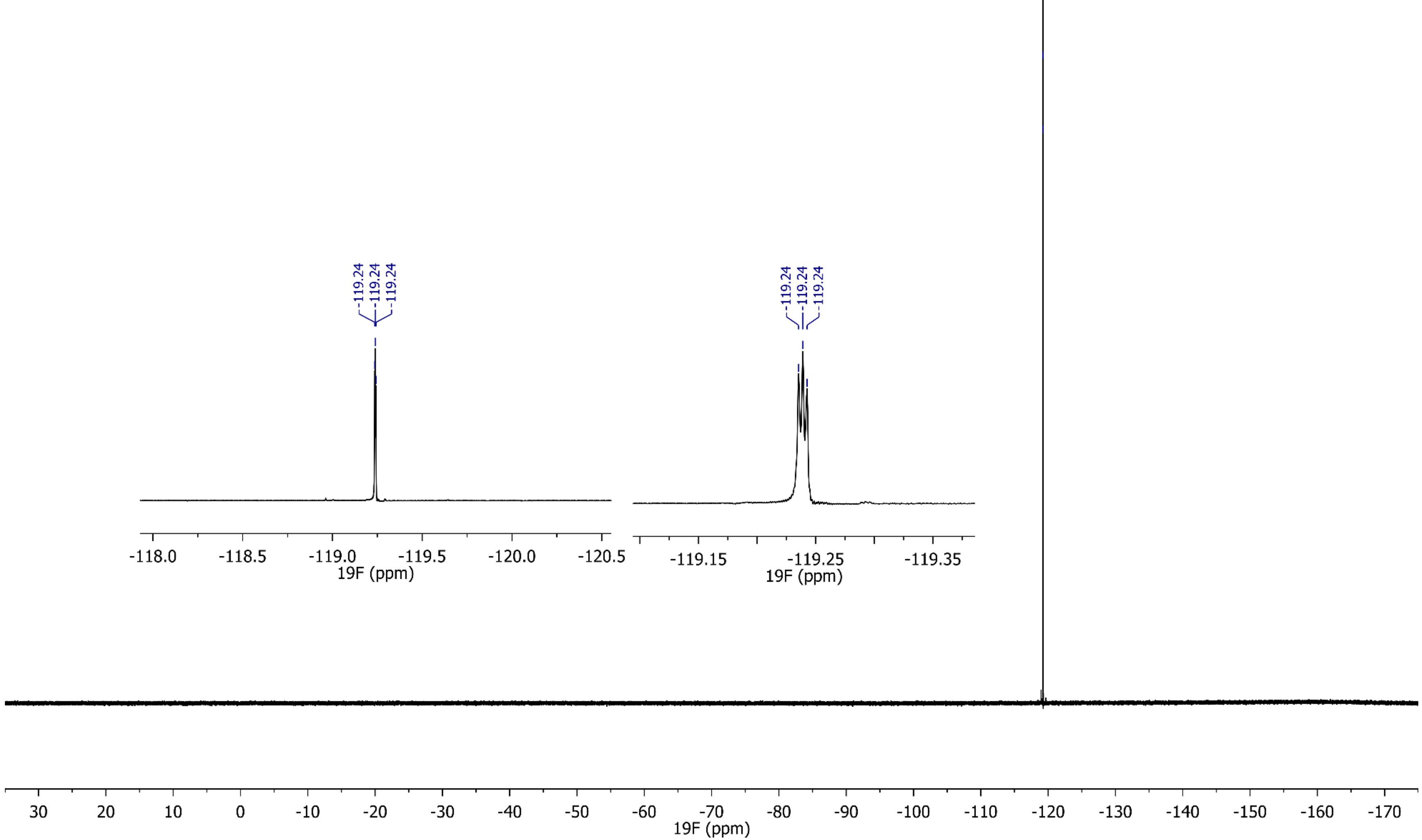

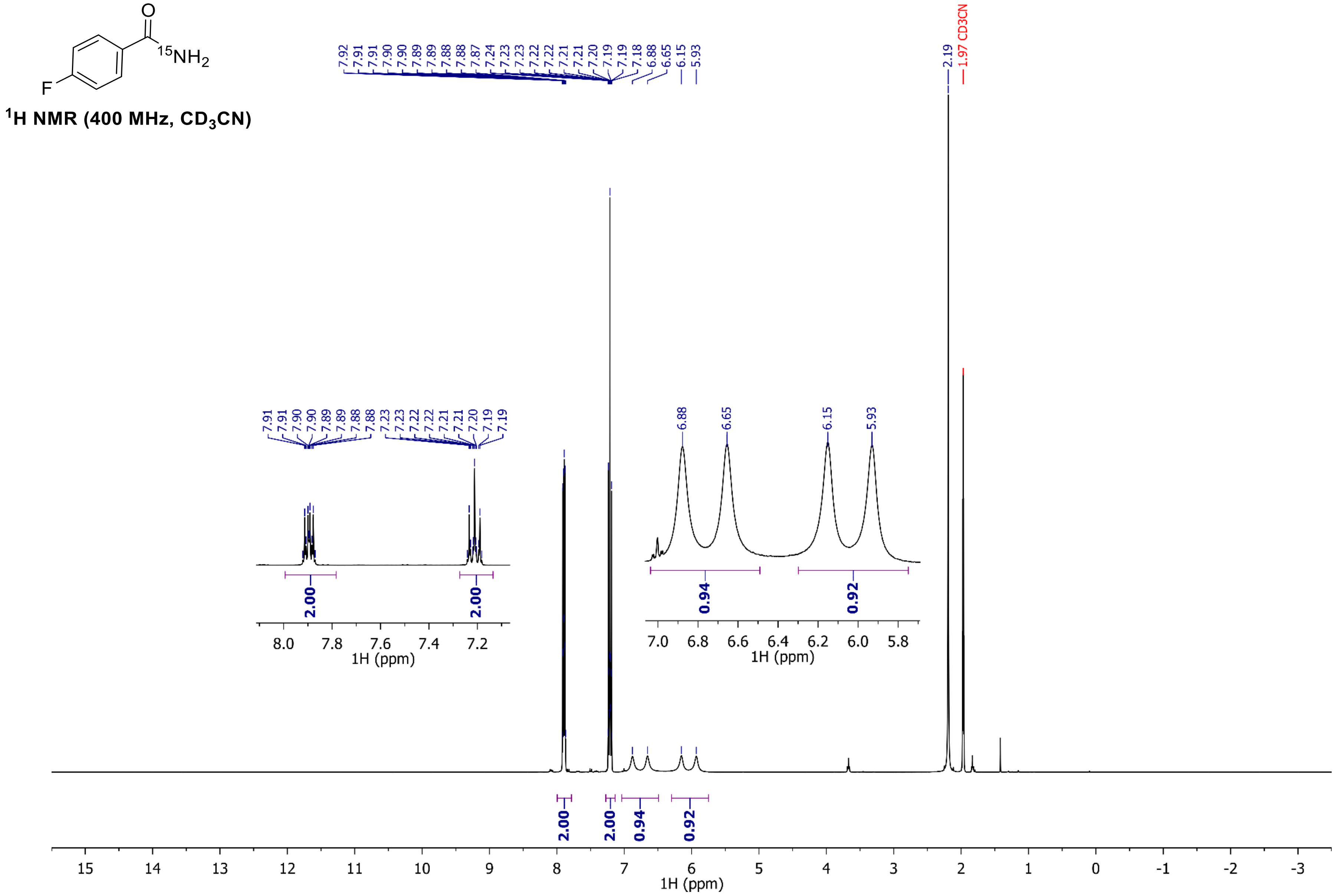

O
$^{15}NH_2$
F
^{1}H NMR (400 MHz, CD_3CN)
7.92 7.91 7.91 7.90 7.90 7.89 7.89 7.88 7.88 7.87 7.24 7.23 7.23 7.22 7.22 7.21 7.21 7.20 7.19 7.19 7.18 6.88 6.65 6.15 5.93
2.19
1.97 CD3CN
2.00 2.00 0.94 0.92
1H (ppm)

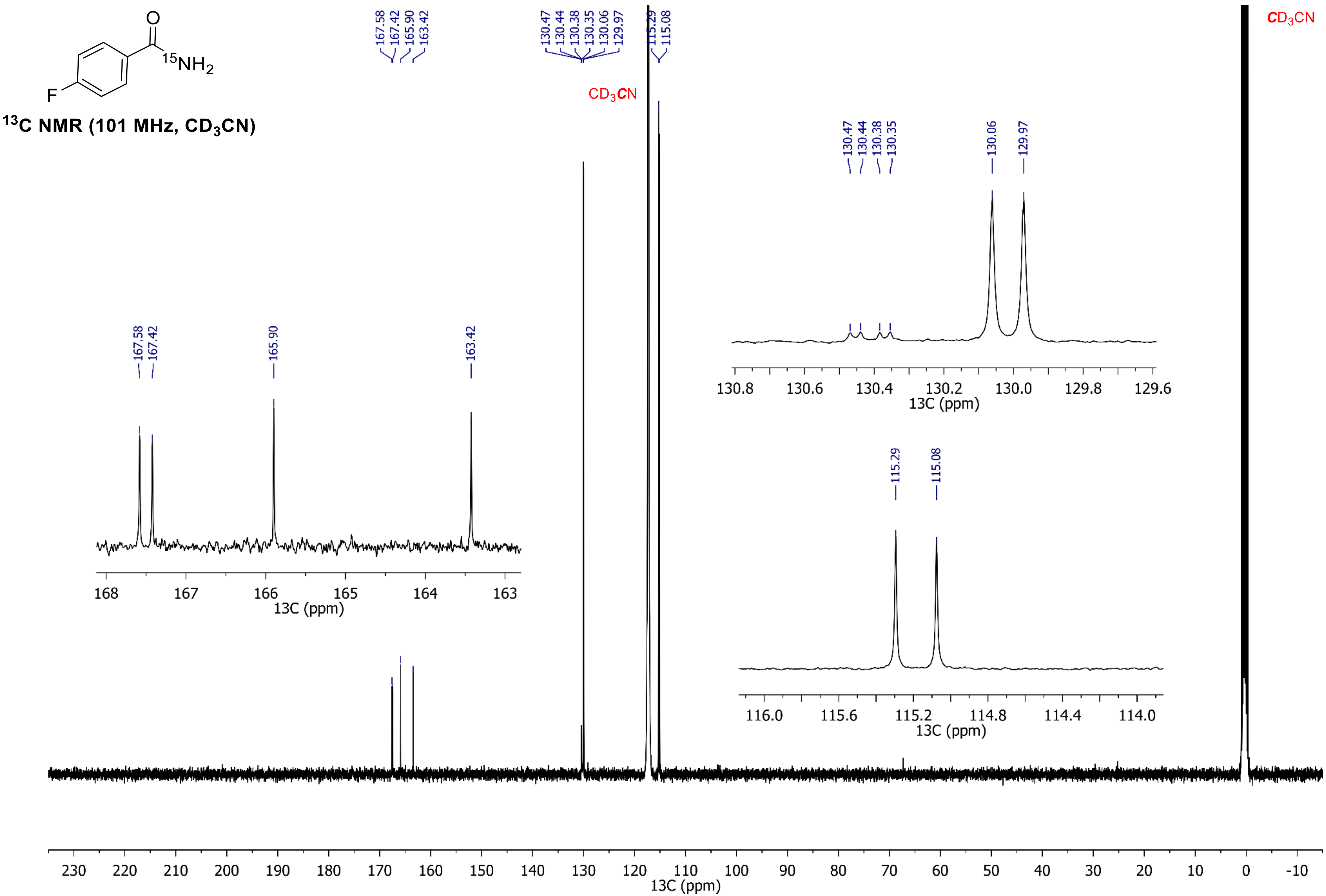

O
$^{15}NH_2$
F
^{13}C NMR (101 MHz, CD_3CN)
167.58
167.42
165.90
163.42
130.47
130.44
130.38
130.35
130.06
129.97
115.29
115.08
CD_3CN
CD_3CN
13C (ppm)

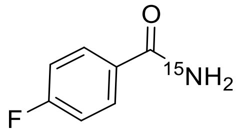

^{19}F NMR (377 MHz, CD_3CN)

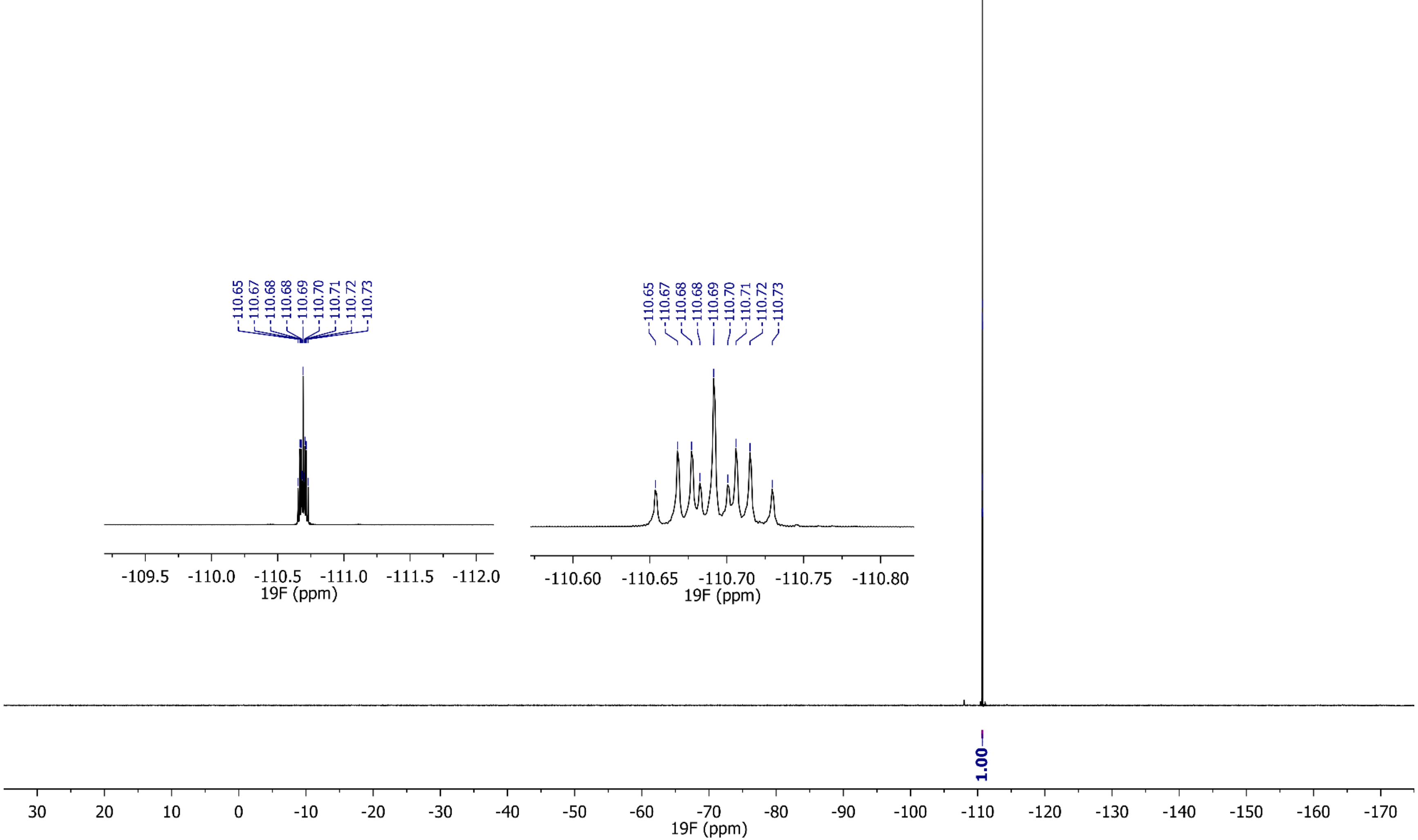

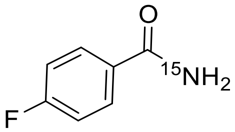

^{15}N NMR (41 MHz, CD_3CN)

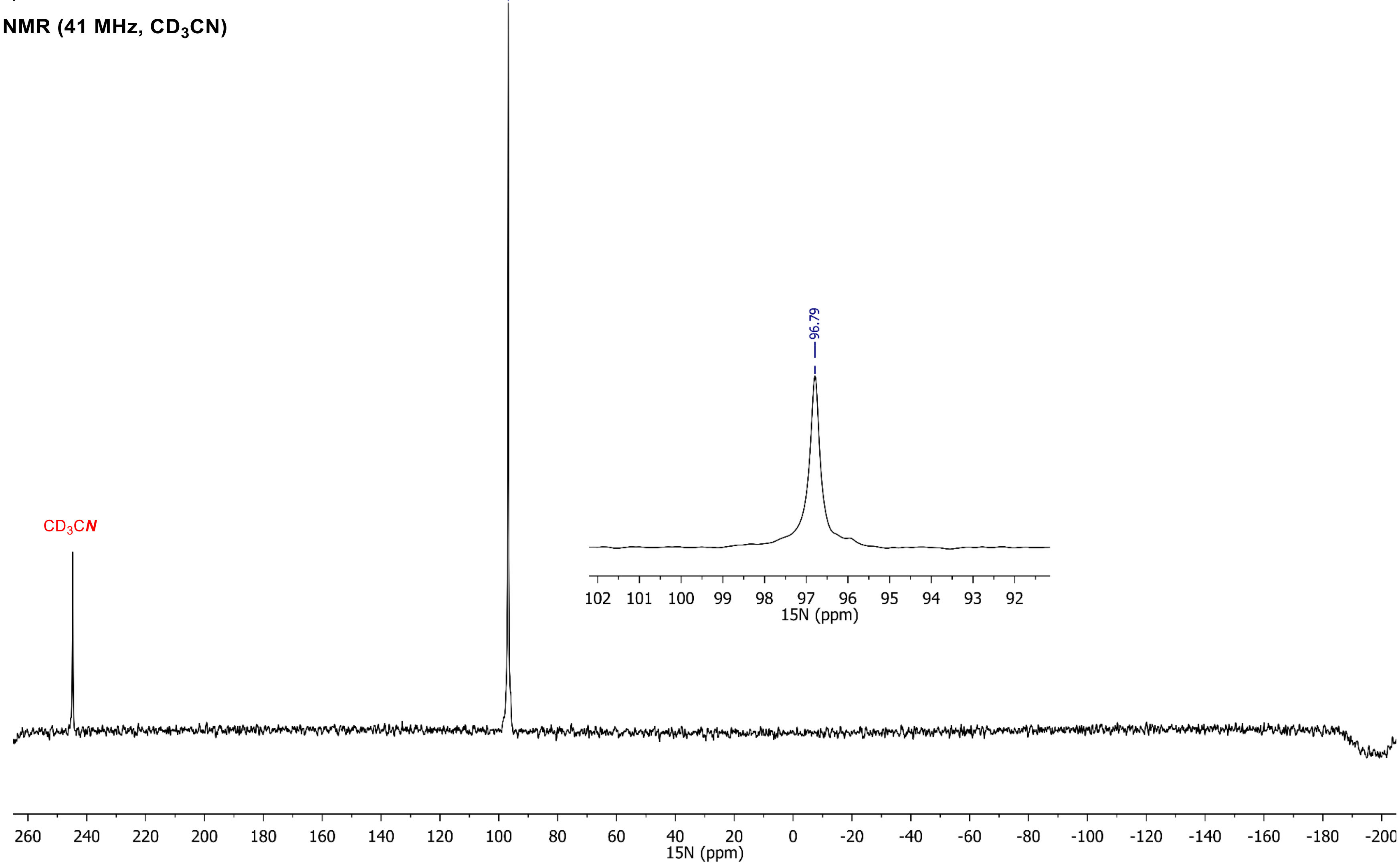

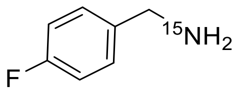

^1H NMR (400 MHz, CD_3CN)

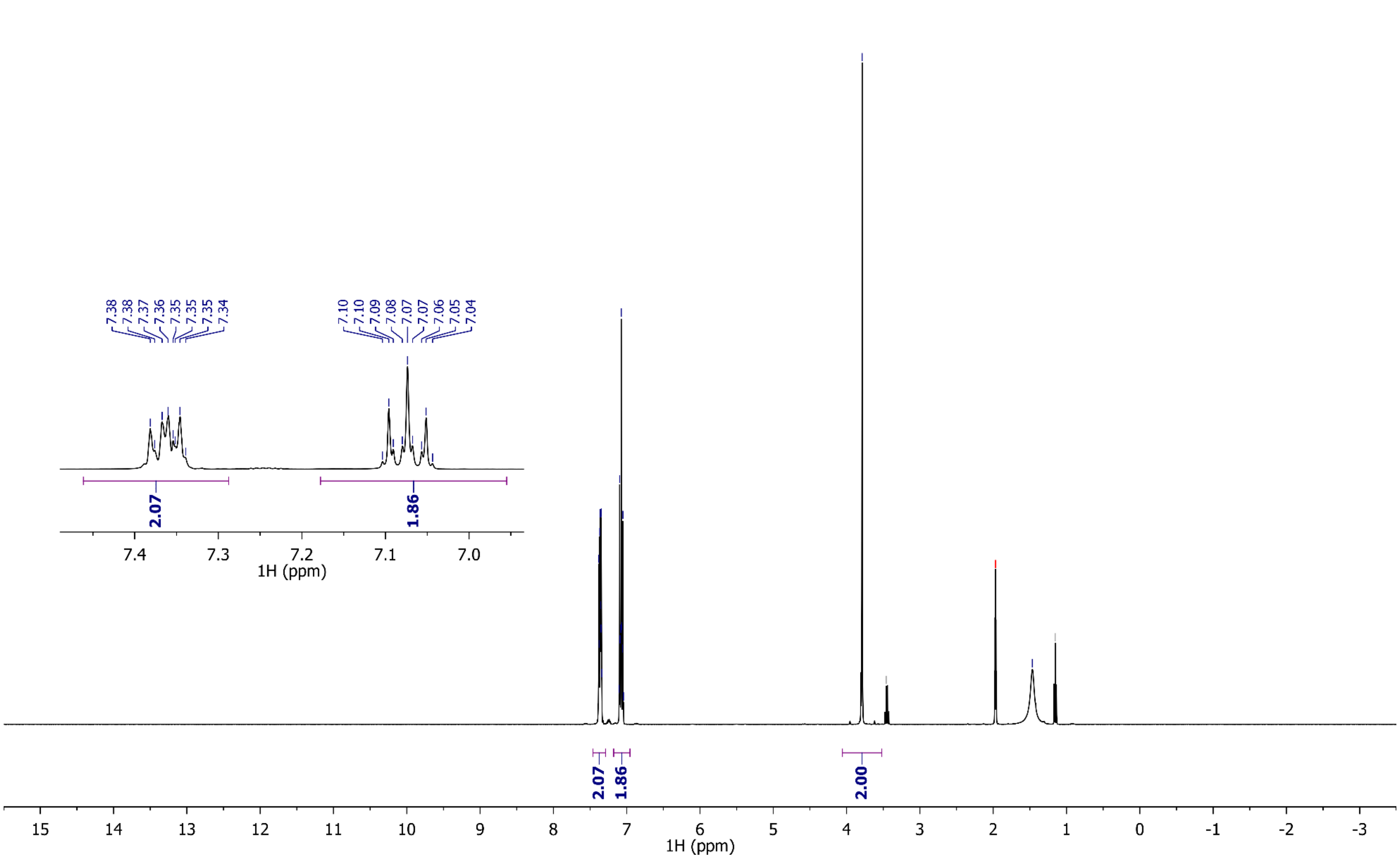

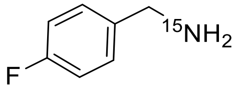

^{13}C NMR (101 MHz, CD_3CN)

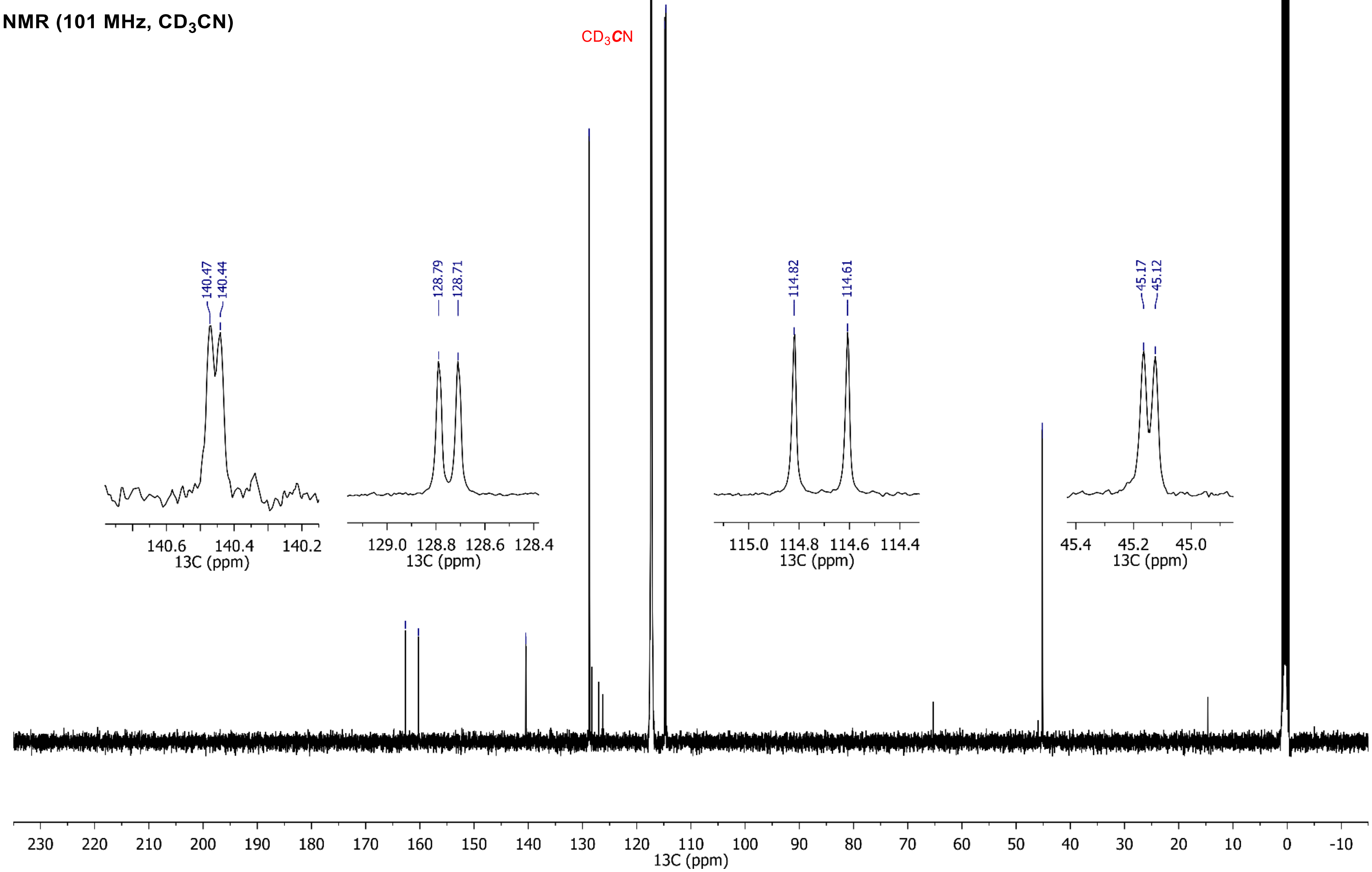

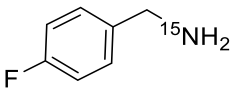

^{19}F NMR (377 MHz, CD_3CN)

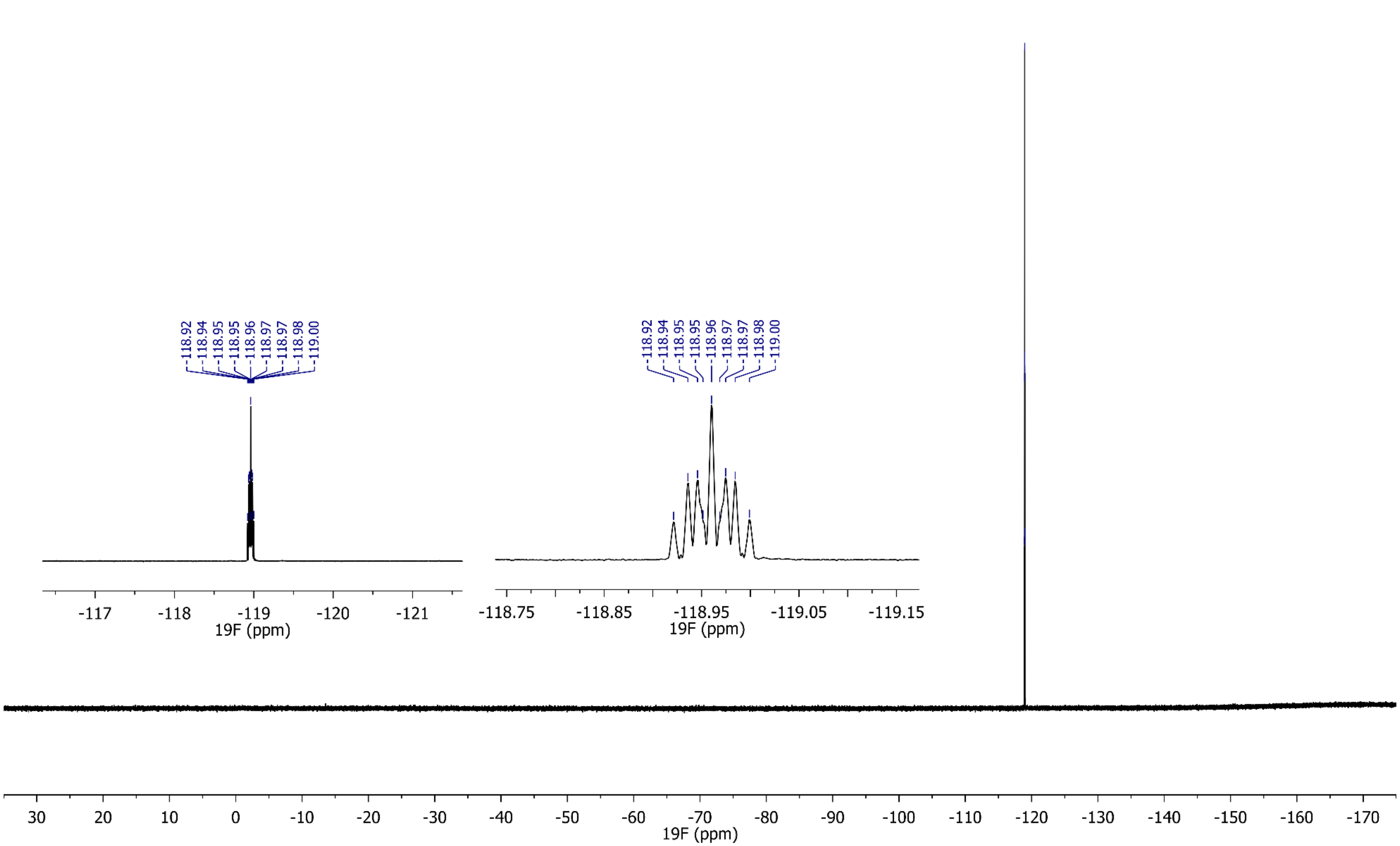

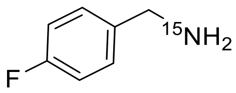

$^{19}F\{^{1}H\}$ NMR (377 MHz, CD_3CN)

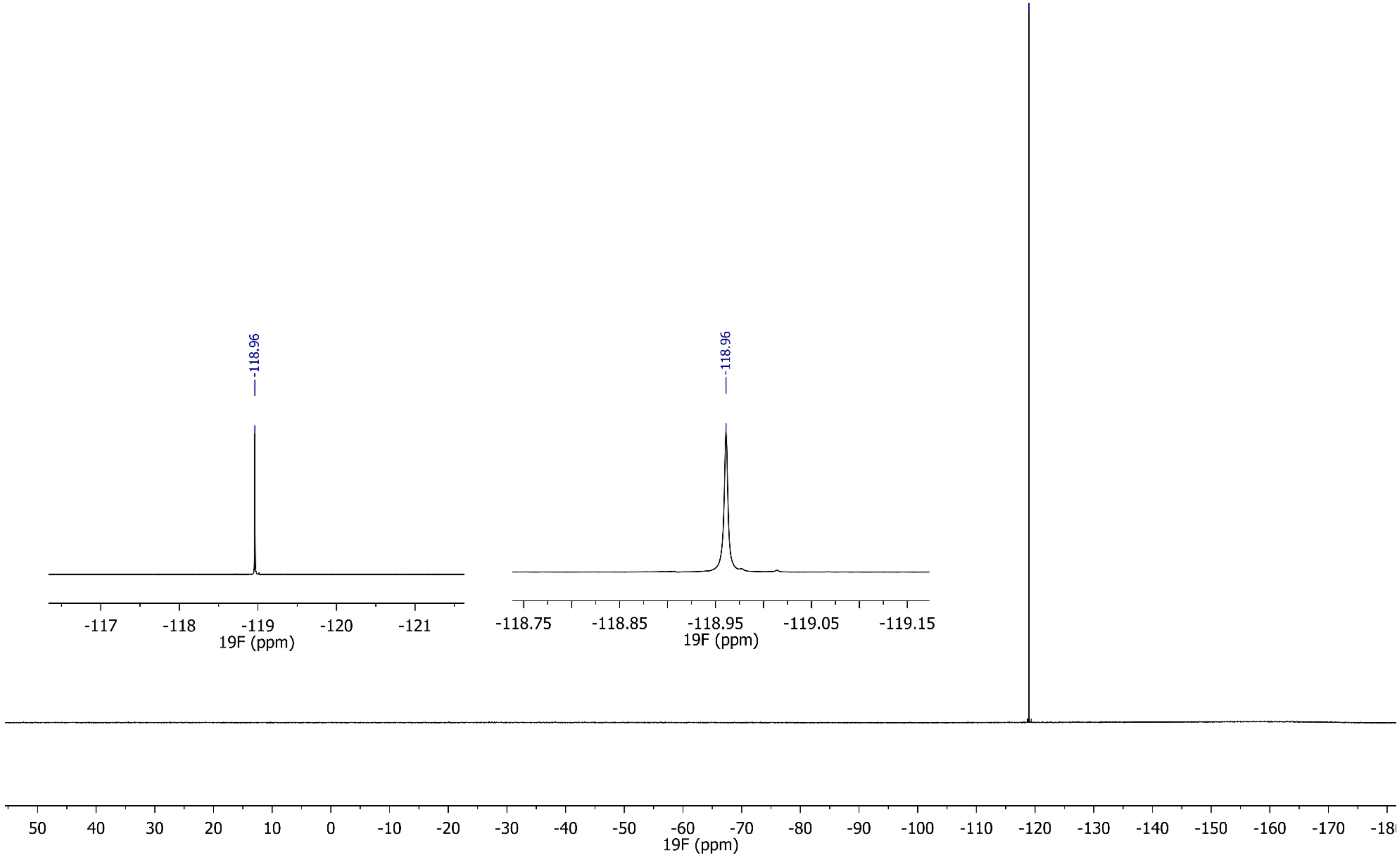

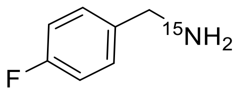

^{15}N NMR (41 MHz, CD_3CN)

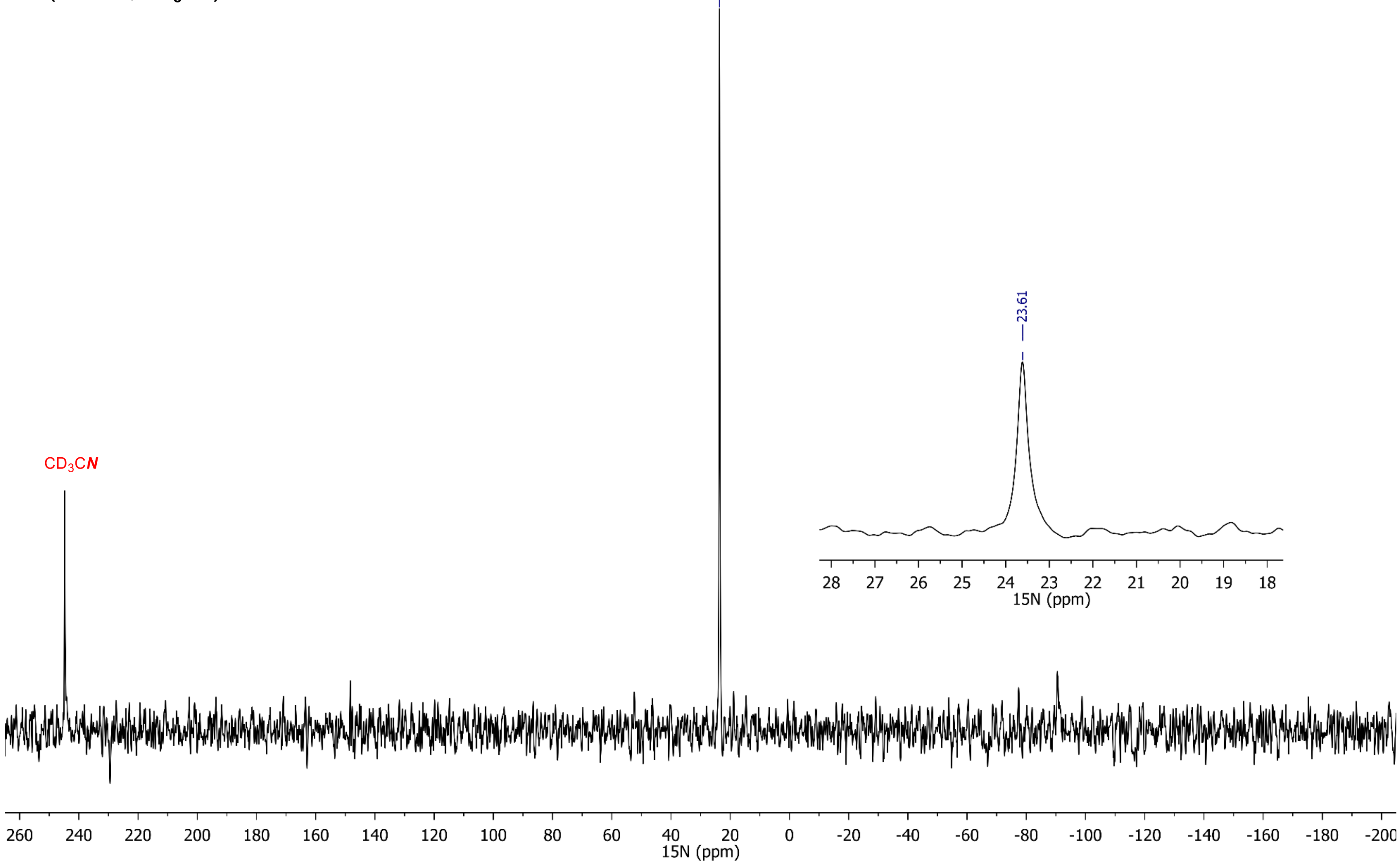

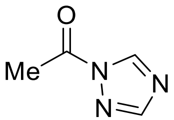

^{1}H NMR (400 MHz, CD_3CN)

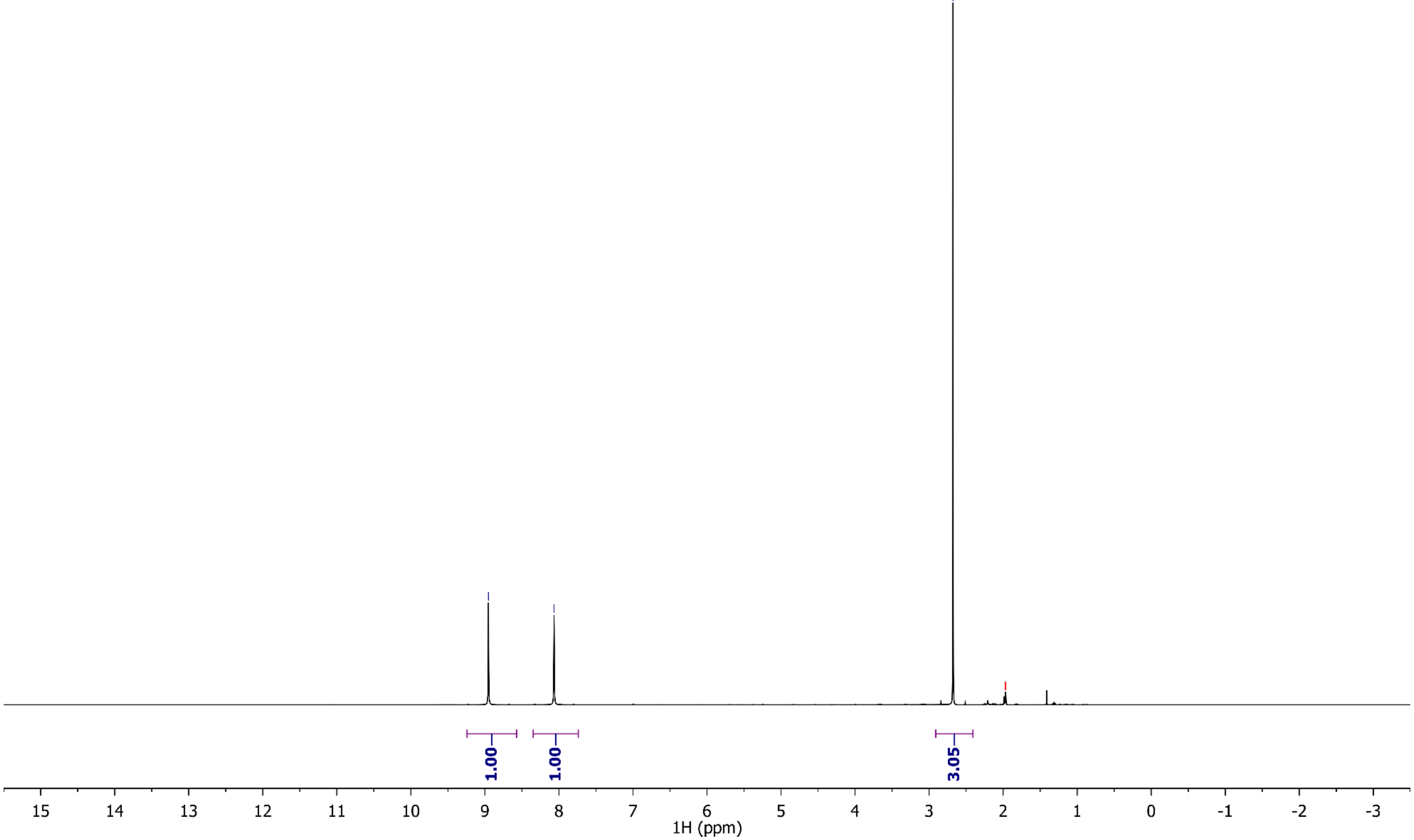

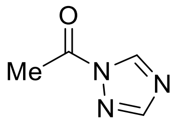

^{13}C NMR (101 MHz, CD_3CN)

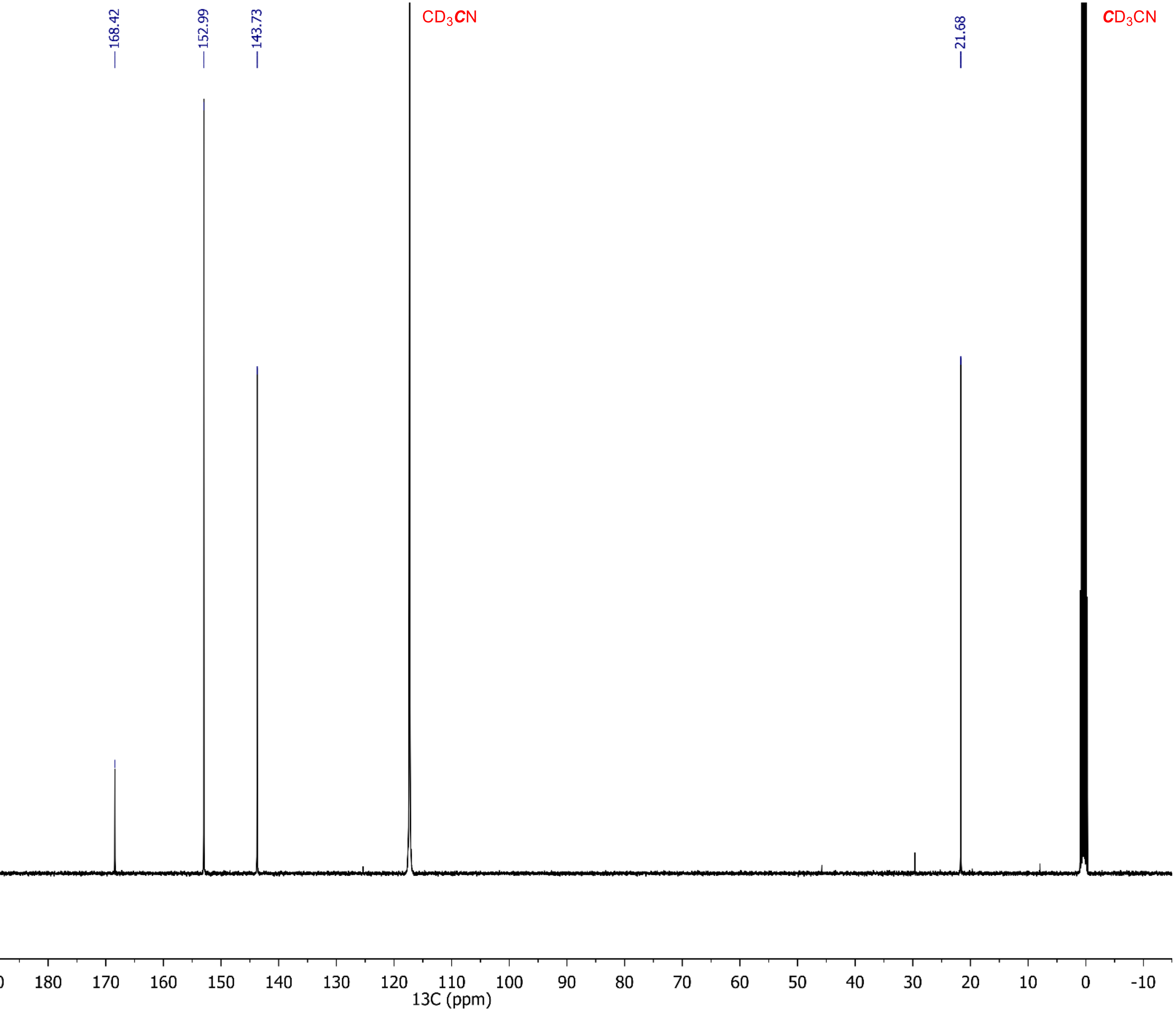

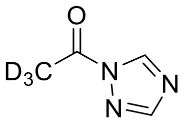

^{1}H NMR (400 MHz, CD_3CN)

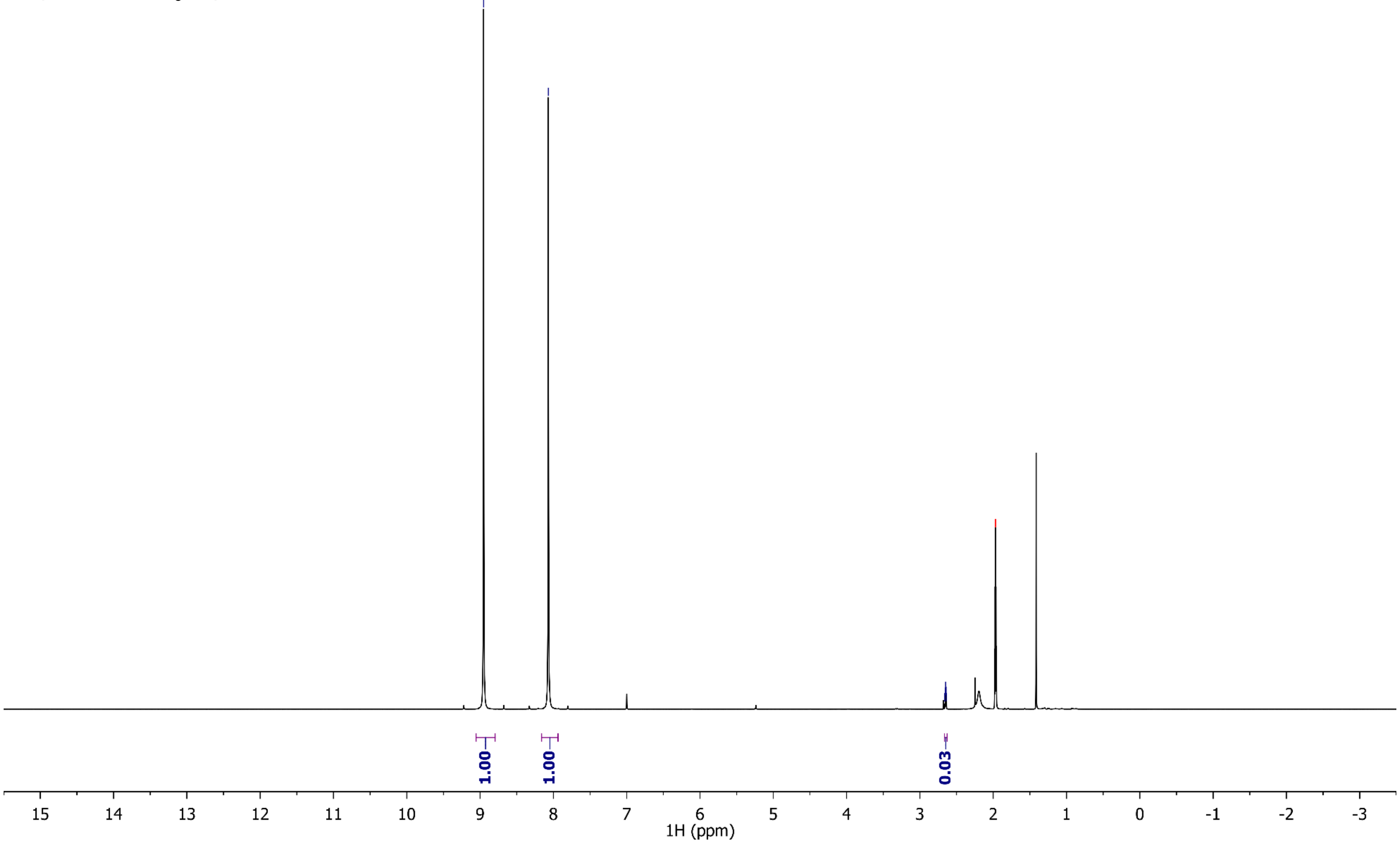

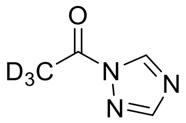

^{13}C NMR (101 MHz, CD_3CN)

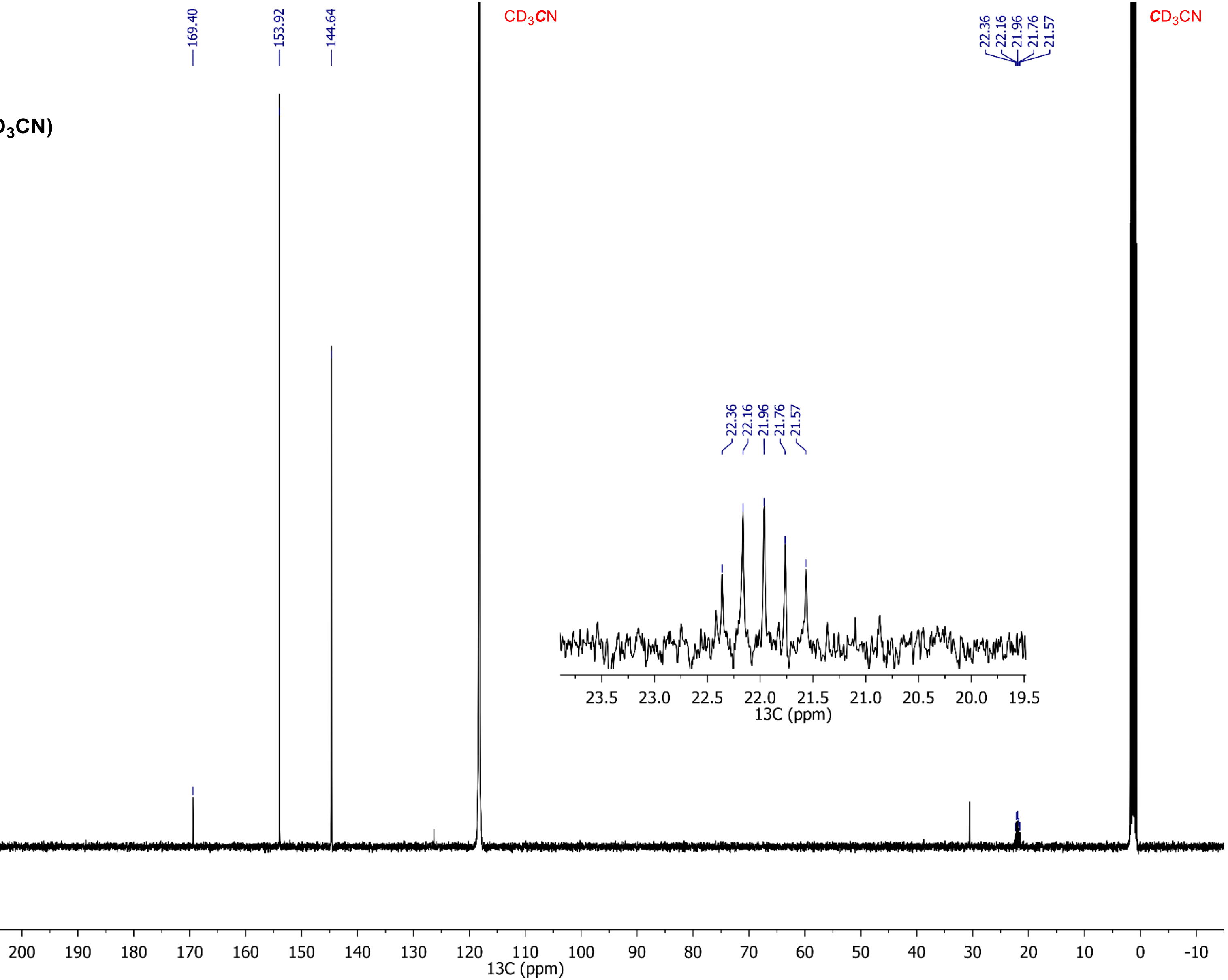

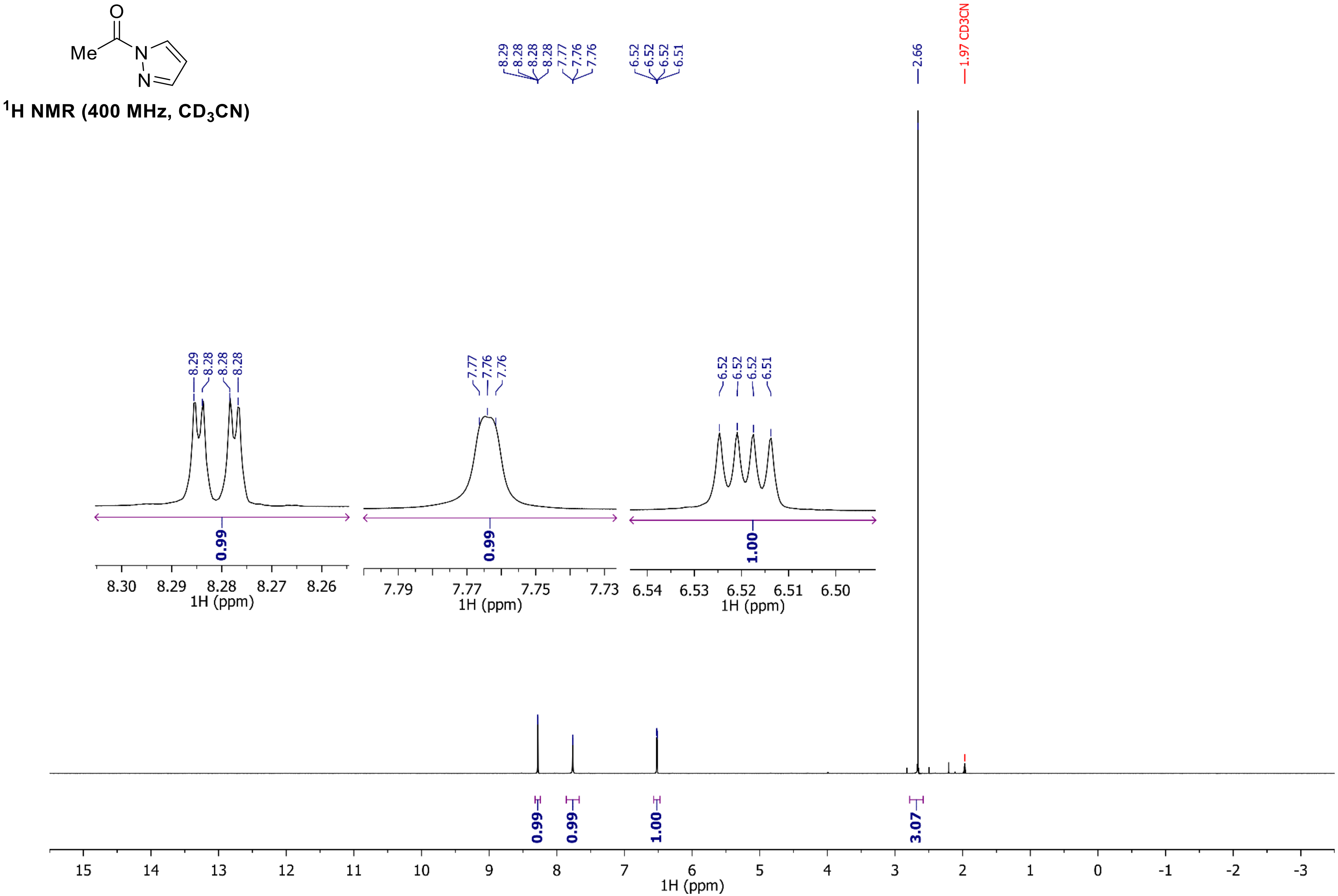
Me
N
N
O
^{1}H NMR (400 MHz, CD_3CN)
8.29
8.28
8.28
8.28
7.77
7.76
7.76
6.52
6.52
6.52
6.51
2.66
1.97 CD3CN
0.99
0.99
1.00
3.07
1H (ppm)

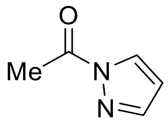

^{13}C NMR (101 MHz, CD_3CN)

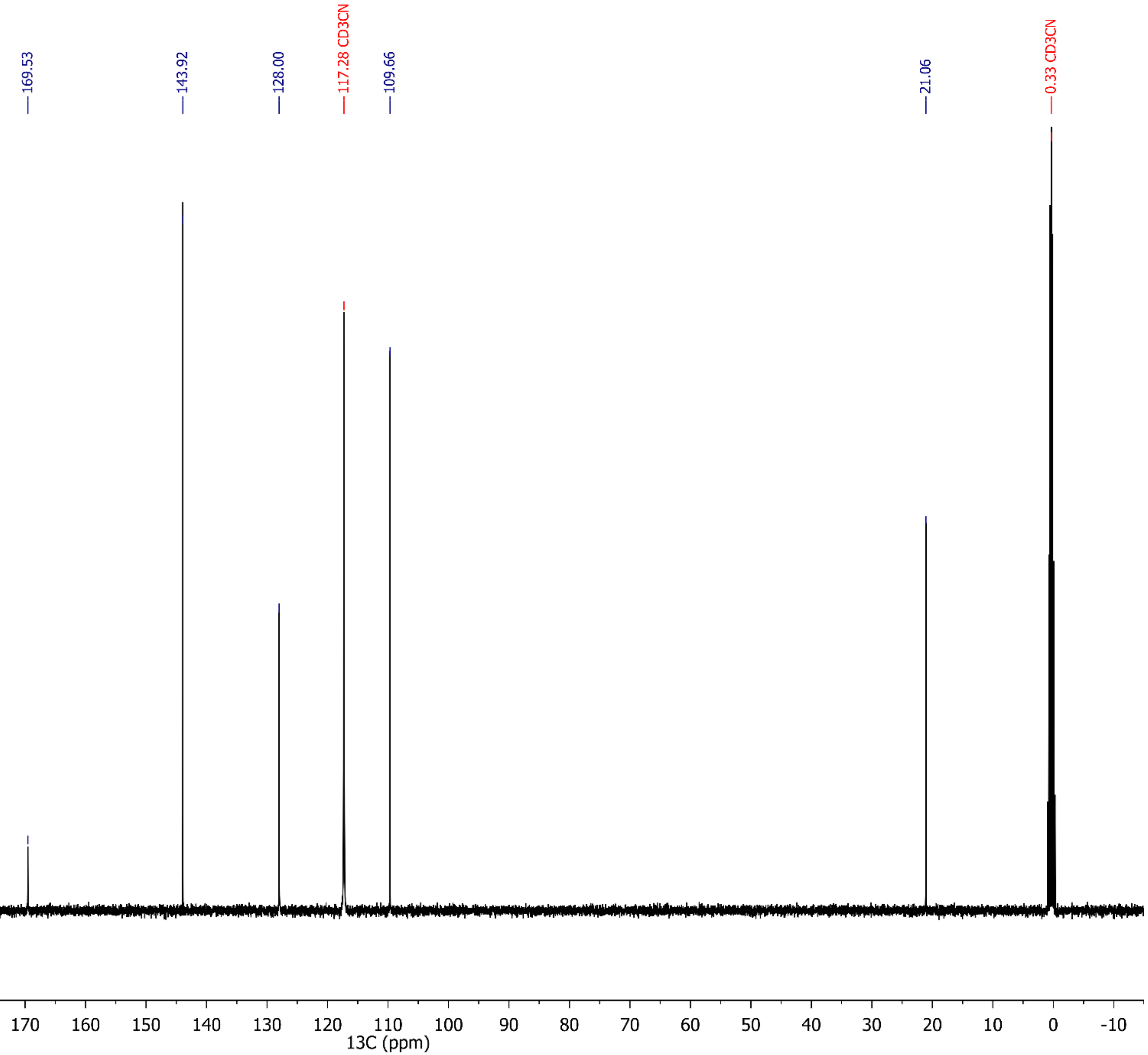

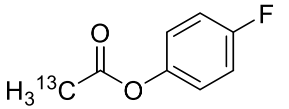

^{1}H NMR (400 MHz, CD_3CN)

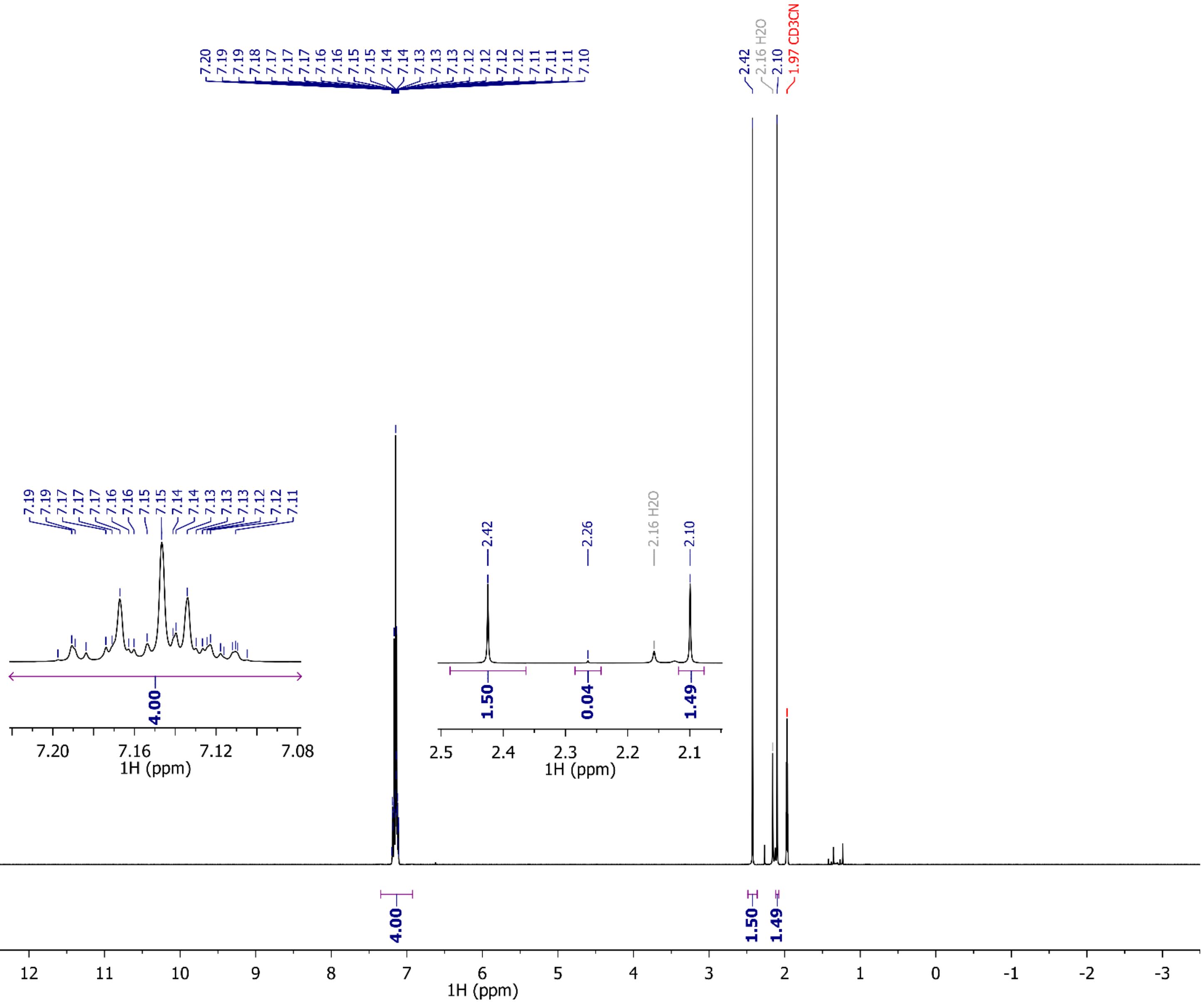

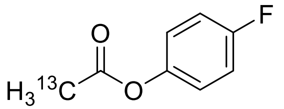

^{13}C NMR (101 MHz, CD_3CN)

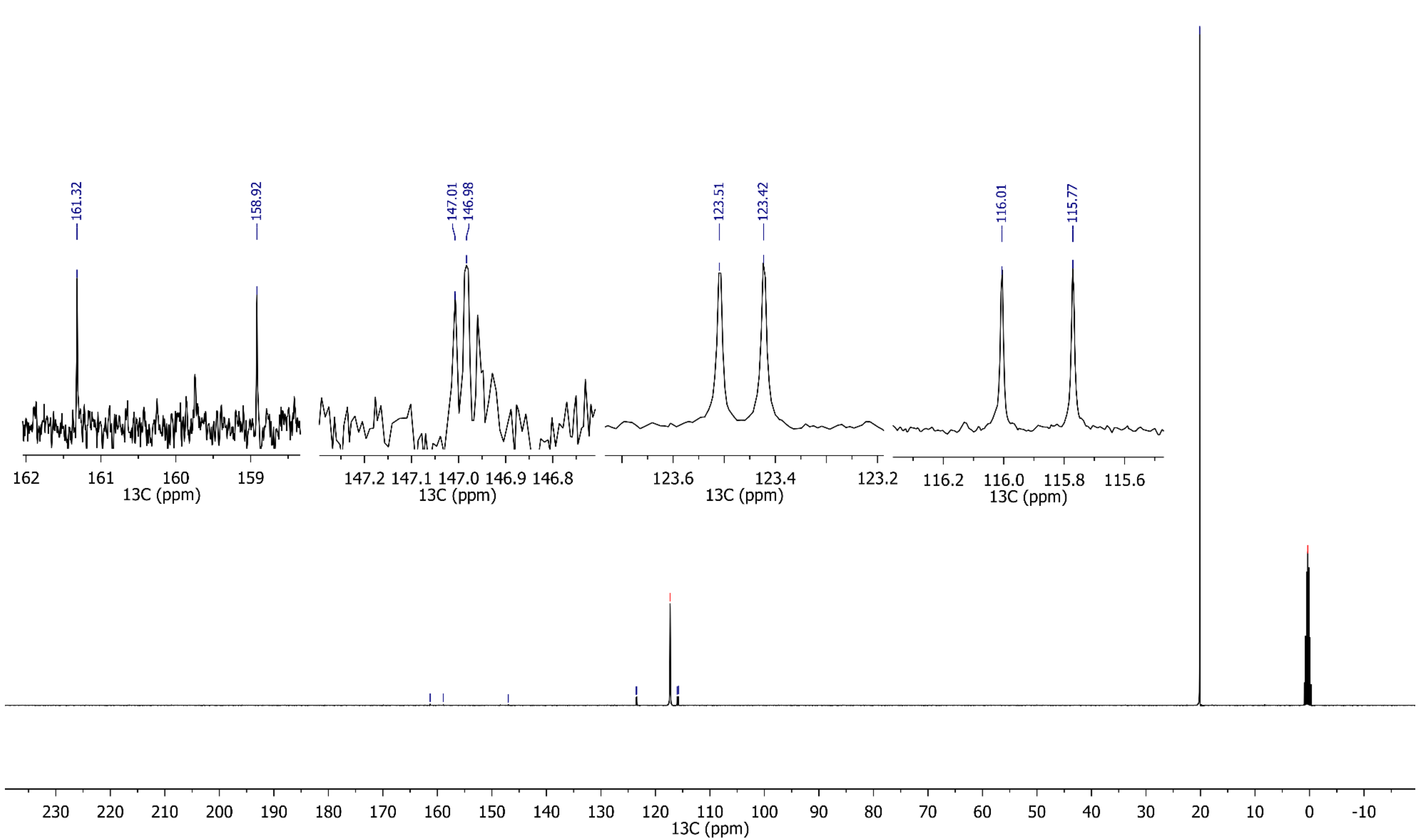

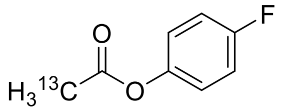

^{19}F NMR (377 MHz, CD_3CN)

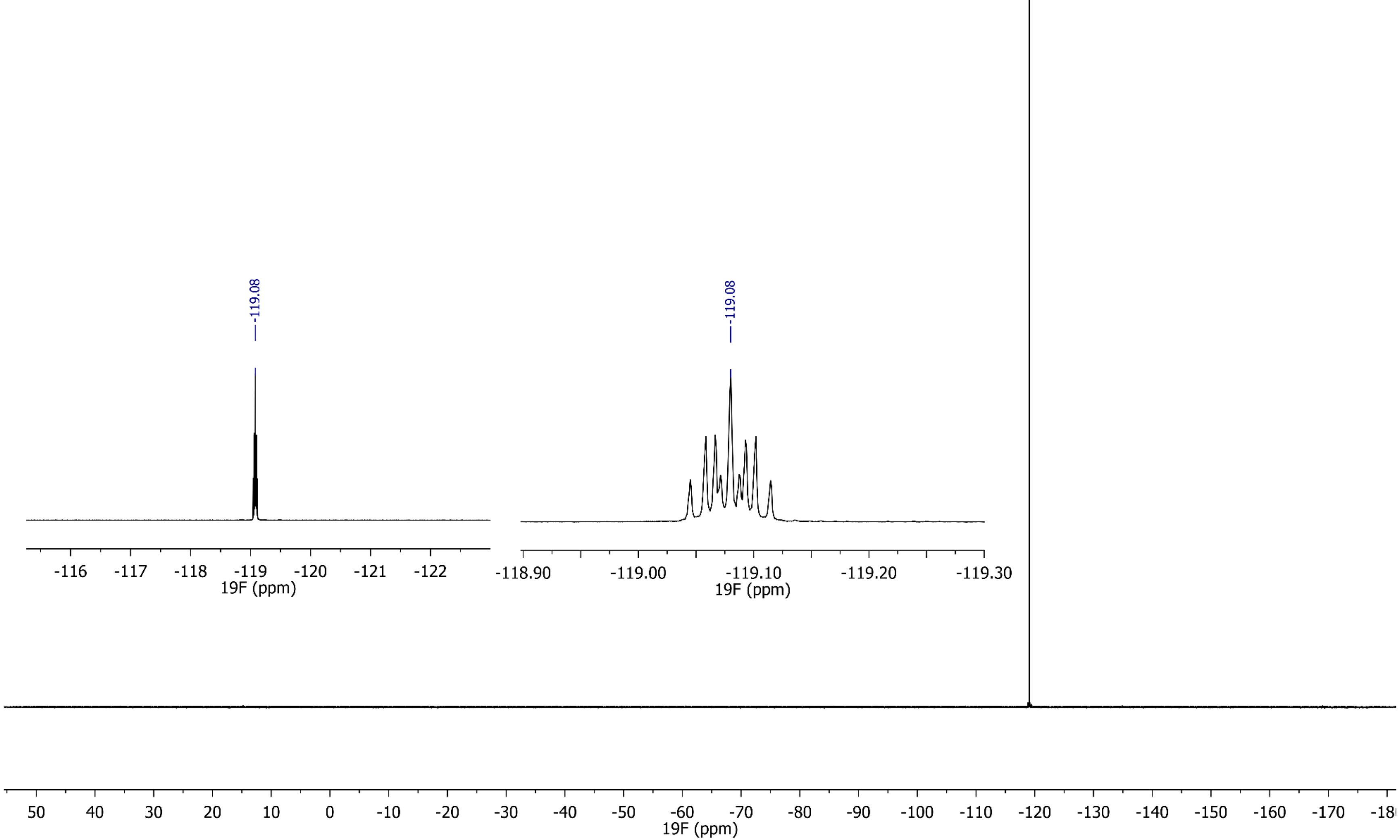

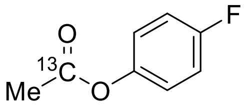

¹H NMR (400 MHz, CD_3CN)

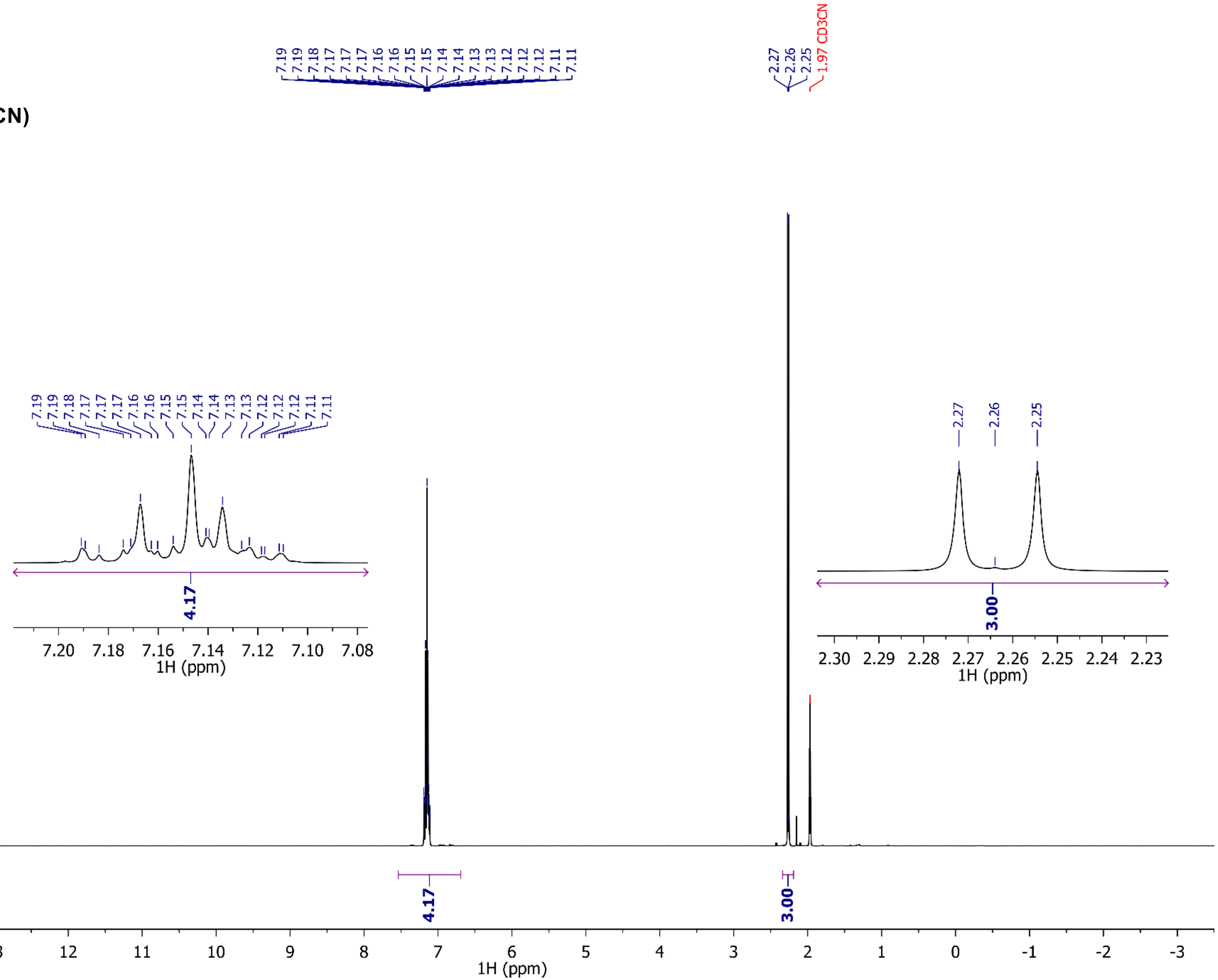

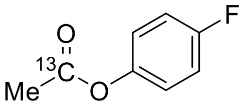

^{13}C NMR (101 MHz, CD_3CN)

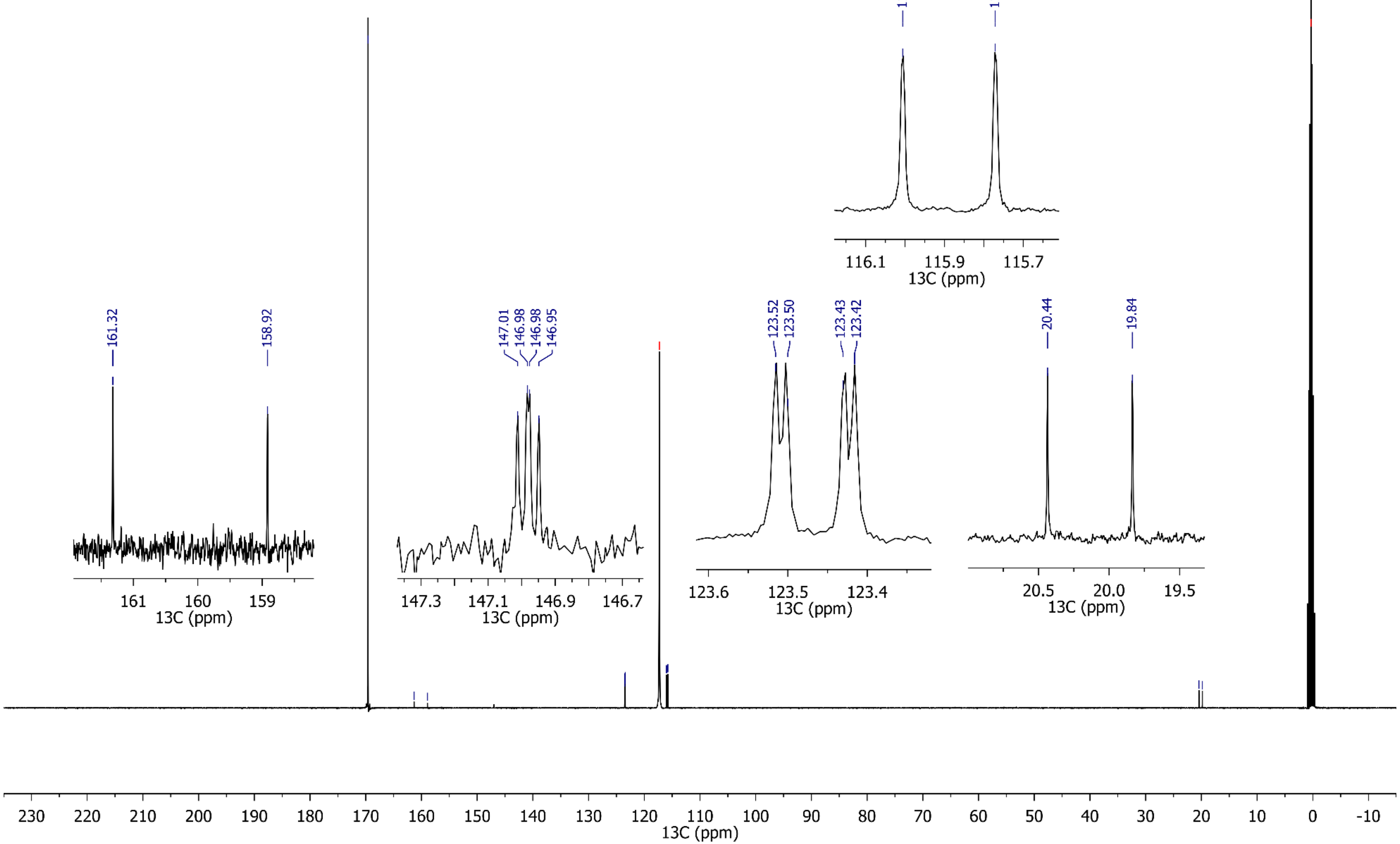

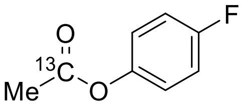

^{19}F NMR (377 MHz, CD_3CN)

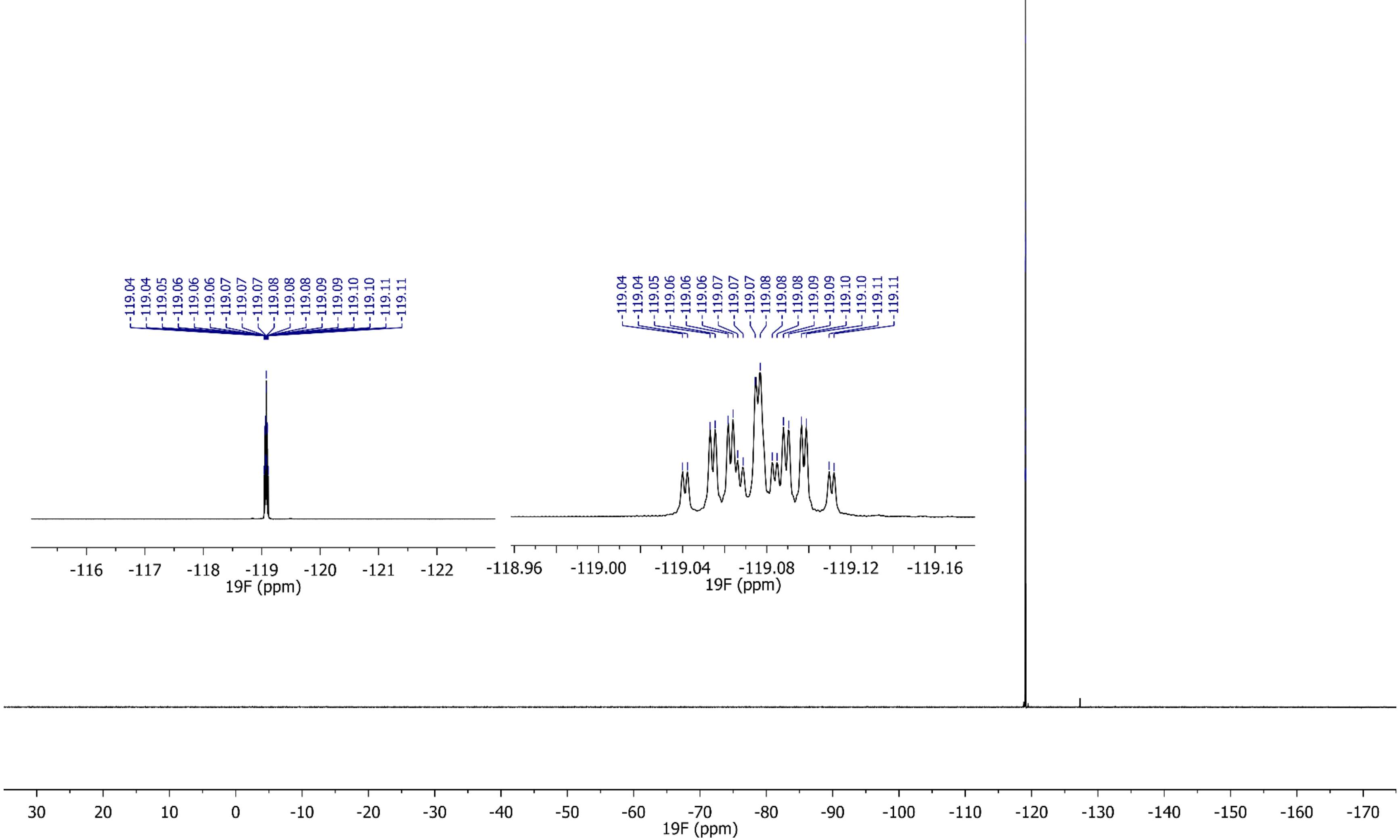

References

S1. Gondo, K.; Oyamada, J.; Kitamura, T., Palladium-Catalyzed Desilylative Acyloxylation of Silicon–Carbon Bonds on (Trimethylsilyl)arenes: Synthesis of Phenol Derivatives from Trimethylsilylarenes. *Org. Lett.* **2015,** *17*, 4778-4781.

S2. Olofson, R. A.; Kendall, R. V., Protection by acylation in the selective alkylation of heterocycles. *J. Org. Chem.* **1970,** *35*, 2246-2248.

S3. Murato, K.; Yatsunami, T.; Iwasaki, S., Photochemische reaktionen. 108. Mitteilung. Photochemistry of N-acylazoles. VI. Photoreactivities of 1-acyl-1,2,4-triazoles and of 2-acyltetrazoles. *Helv. Chim. Acta* **1980,** *63*, 588-605.

S4. Zhang, J.; Jia, R.-P.; Wang, D.-H., Copper-catalyzed C–N cross-coupling of arylboronic acids with N-acylpyrazoles. *Tetrahedron Lett.* **2016,** *57*, 3604-3607.

S5. (a) Johnston, C. P.; West, T. H.; Dooley, R. E.; Reid, M.; Jones, A. B.; King, E. J.; Leach, A. G.; Lloyd-Jones, G. C., Anion-Initiated Trifluoromethylation by TMSCF3: Deconvolution of the Siliconate–Carbanion Dichotomy by Stopped-Flow NMR/IR. *J. Am. Chem. Soc.* **2018,** *140*, 11112-11124; (b) Wei, R.; Hall, A. M. R.; Behrens, R.; Pritchard, M .S.; King, E. J.; Lloyd-Jones, G. C. Stopped-Flow ^{19}F NMR Spectroscopic Analysis of a Protodeboronation Proceeding at the Sub-Second Time-Scale. *Eur. J. Org. Chem.*, **2021**, *17*, 2332–2342.

S6. Wei, R.; Dickson, C. L.; Uhrín, D.; Lloyd-Jones, G. C., Rapid Estimation of T1 for Quantitative NMR. *J. Org. Chem.* **2021,** *86*, 9023-9029.

S7. Burés, J., Variable Time Normalization Analysis: General Graphical Elucidation of Reaction Orders from Concentration Profiles. *Angew. Chem. Int. Ed.* **2016,** *55*, 16084-16087.

S8. Nielsen, C. D. T.; Burés, J., Visual kinetic analysis. *Chem. Sci.* **2019,** *10*, 348-353.

S9. King, E. L.; Altman, C., A Schematic Method of Deriving the Rate Laws for Enzyme-Catalyzed Reactions. *J. Phys. Chem.* **1956,** *60*, 1375-1378.

S10. Sims, P. A., An "Aufbau" Approach To Understanding How the King–Altman Method of Deriving Rate Equations for Enzyme-Catalyzed Reactions Works. *J. Chem. Educ.* **2009,** *86*, 385.

S11. Bigeleisen, J.; Wolfsberg, M., Theoretical and Experimental Aspects of Isotope Effects in Chemical Kinetics. In *Advances in Chemical Physics*, 1957; pp 15-76.

S12. Neufeld, R.; Stalke, D., Accurate molecular weight determination of small molecules via DOSY-NMR by using external calibration curves with normalized diffusion coefficients. *Chem. Sci.* **2015,** *6*, 3354-3364.

S13. Sinnaeve, D., The Stejskal–Tanner equation generalized for any gradient shape—an overview of most pulse sequences measuring free diffusion. *Concepts Magn. Reson. A: Bridg. Educ. Res.* **2012,** *40A*, 39-65.

S14. M. J. Frisch, G. W. Trucks, H. B. Schlegel, G. E. Scuseria, M. A. Robb, J. R. Cheeseman, G. Scalmani, V. Barone, B. Mennucci, G. A. Petersson, H. Nakatsuji, M. Caricato, X. Li, H. P. Hratchian, A. F. Izmaylov, J. Bloino, G. Zheng, J. L. Sonnenberg, M. Hada, M. Ehara, K. Toyota, R. Fukuda, J. Hasegawa, M. Ishida, T. Nakajima, Y. Honda, O. Kitao, H. Nakai, T. Vreven, J. A. Montgomery, Jr., J. E. Peralta, F. Ogliaro, M. Bearpark, J. J. Heyd, E. Brothers, K. N. Kudin, V. N. Staroverov, R. Kobayashi, J. Normand, K. Raghavachari, A. Rendell, J. C. Burant, S. S. Iyengar, J. Tomasi, M. Cossi, N. Rega, J. M. Millam, M. Klene, J. E. Knox, J. B. Cross, V. Bakken, C. Adamo, J. Jaramillo, R. Gomperts, R. E. Stratmann, O. Yazyev, A. J. Austin, R. Cammi, C. Pomelli, J. W. Ochterski, R. L. Martin, K. Morokuma, V. G. Zakrzewski, G. A. Voth, P. Salvador, J. J. Dannenberg, S. Dapprich, A. D. Daniels, Ö. Farkas, J.

B. Foresman, J. V. Ortiz, J. Cioslowski, and D. J. Fox, Gaussian 09 (Gaussian, Inc., Wallingford CT, 2009).

S15. Adamo, C.; Barone, V., Toward reliable density functional methods without adjustable parameters: The PBE0 model. *J. Chem. Phys.* **1999,** *110*, 6158-6170.

S16. Grimme, S., Ehrlich, S., Goerigk, L., Effect of the Damping Function in Dispersion Corrected Density Functional Theory. *J. Comp. Chem.* **2011,** *32*, 1456-1465.

S17. Grimme, S., Supramolecular binding thermodynamics by dispersion-corrected density functional theory. *Chem. Eur. J.* **2012,** *18*, 9955-9964.

S18. Grimme, S.; Steinmetz, M., Effects of London dispersion correction in density functional theory on the structures of organic molecules in the gas phase. *Phys. Chem. Chem. Phys.* **2013,** *15*, 16031-16042.

S19. Luchini, G.; Alegre-Requena, J. V.; Funes-Ardoiz, I.; Paton, R. S. GoodVibes: Automated Thermochemistry for Heterogeneous Computational Chemistry Data. F1000Research, 2020, 9, 291 DOI: 10.12688/f1000research.22758.1

S20. Neese, F., The ORCA program system. *Wiley Interdiscip. Rev. Comput. Mol. Sci.* **2012,** *2*, 73-78.

S21. Neese, F., Software update: the ORCA program system, version 4.0. *Wiley Interdiscip. Rev. Comput. Mol. Sci.* **2018,** *8*, 4-9.

S22. Anderson, T.L.; Kwan, E.E. PyQuiver 2020, www.github.com/ekwan/PyQuiver

S23. Kesharwani, M. K.; Brauer, B.; Martin, J. M. L., Frequency and Zero-Point Vibrational Energy Scale Factors for Double-Hybrid Density Functionals (and Other Selected Methods): Can Anharmonic Force Fields Be Avoided? *J. Phys. Chem. A* **2015,** *119*, 1701-1714.

S24. Sastre, S.; Casasnovas, R.; Muñoz, F.; Frau, J., Isodesmic reaction for accurate theoretical pKa calculations of amino acids and peptides. *Phys. Chem. Chem. Phys.* **2016,** *18*, 11202-11212.

S25. Kütt, A.; Tshepelevitsh, S.; Saame, J.; Lõkov, M.; Kaljurand, I.; Selberg, S.; Leito, I., Strengths of Acids in Acetonitrile. *Eur. J. Org. Chem.* **2021,** *2021*, 1407-1419.

S26. Bordwell, F. G., Equilibrium Acidities in Dimethyl Sulfoxide Solution. *Acc. Chem. Res*. **1988,** *21*, 456-463.

S27. Koldobskii, G. I.; Ostrovskii, V. A.; Gidaspov, B. V., Tautomerism and acid-base properties of tetrazoles (review). *Chem. Heterocycl. Compounds* **1980,** *16*, 665-674.

S28. Albert, A.; Taylor, P. J., The tautomerism of 1,2,3-triazole in aqueous solution. *J. Chem. Soc., Perkin Transac. 2* **1989**, 1903-1905.

S29. Cox, J. R.; Woodcock, S.; Hillier, I. H.; Vincent, M. A., Tautomerism of 1,2,3- and 1,2,4-triazole in the gas phase and in aqueous solution: a combined ab initio quantum mechanics and free energy perturbation study. *J. Phys. Chem.* **1990,** *94*, 5499-5501.

S30. Wong, M. W.; Leung-Toung, R.; Wentrup, C., Tautomeric equilibrium and hydrogen shifts of tetrazole in the gas phase and in solution. *J. Am. Chem. Soc.* **1993,** *115*, 2465-2472.

S31. Davarski, K. A.; Khalachev, N. K.; Yankova, R. Z.; Raikov, S., Quantum chemical study of the tautomerism, geometry, and electronic structure of 1,2,3- and 1,2,4-triazoles. *Chem. Heterocycl. Compounds* **1998,** *34*, 568-574.

S32. Elguero, J.; Katritzky, A. R.; Denisko, O. V., Prototropic Tautomerism of Heterocycles: Heteroaromatic Tautomerism—General Overview and Methodology. In *Advances in Heterocyclic Chemistry*, Katritzky, A. R., Ed. Academic Press: 2000; Vol. 76, pp 1-84.

S33. Mazurek, A. P.; Sadlej-Sosnowska, N., Studies on tautomerism in tetrazole: comparison of Hartree–Fock and density functional theory quantum chemical methods. *Chem. Phys. Lett.* **2000,** *330*, 212-218.

S34. Minkin, V. I.; Garnovskii, A. D.; Elguero, J.; Katritzky, A. R.; Denisko, O. V., The Tautomerism of Heterocycles: Five-membered Rings with Two or More Heteroatoms. In *Advances in Heterocyclic Chemistry*, Katritzky, A. R., Ed. Academic Press: 2000; Vol. 76, pp 157-323.

S35. Murdock, S. E.; Lynden-Bell, R. M.; Kohanoff, J.; Margulis, C. J.; Sexton, G. J., Solvation effects on equilibria: Triazoles and N-methyl piperidinol. *Phys. Chem. Chem. Phys.* **2002,** *4*, 5281-5288.

S36. Satchell, J. F.; Smith, B. J., Calculation of aqueous dissociation constants of 1,2,4-triazole and tetrazole: A comparison of solvation models. *Phys. Chem. Chem. Phys.* **2002,** *4*, 4314-4318.

S37. Nagy, P. I.; Tejada, F. R.; Messer, W. S., Theoretical Studies of the Tautomeric Equilibria for Five-Member N-Heterocycles in the Gas Phase and in Solution. *J. Phys. Chem. B* **2005,** *109*, 22588-22602.

S38. Jiménez, V.; Alderete, J. B., Complete basis set calculations on the tautomerism and protonation of triazoles and tetrazole. *Journal of Molecular Structure: THEOCHEM* **2006,** *775*, 1-7.

S39. Balabin, R. M., Tautomeric equilibrium and hydrogen shifts in tetrazole and triazoles: Focal-point analysis and ab initio limit. *J. Chem. Phys.* **2009,** *131*, 154307.

S40. Oziminski, W. P., Theoretical study on the solvent influence on 1,2,3-triazole tautomeric equilibrium. A comparison of incremental microsolvation and continuum solvation model approaches. *Tetrahedron* **2013,** *69*, 3197-3205.

S41. Yang, X.; Bumbu, V. D.; Birman, V. B., Kinetic Resolution of β-Lactams via Enantioselective N-Acylation. Org. Lett. 2011, 13, 4755-4757.
